# Genome-wide identification and expression analysis of two-component system genes in sweet potato (*Ipomoea batatas* L.)

**DOI:** 10.3389/fpls.2022.1091620

**Published:** 2023-01-12

**Authors:** Ruxue Huo, Yanshu Zhao, Tianxu Liu, Meng Xu, Xiaohua Wang, Ping Xu, Shengjie Dai, Xiaoyu Cui, Yonghua Han, Zhenning Liu, Zongyun Li

**Affiliations:** ^1^ Jiangsu Key Laboratory of Phylogeny and Comparative Genomics, School of Life Sciences, Institute of Integrative Plant Biology, Jiangsu Normal University, Xuzhou, China; ^2^ College of Agriculture and Forestry Science, Linyi University, Linyi, China

**Keywords:** two-component system, sweet potato, phylogeny, evolution, expression profiles

## Abstract

Two-component system (TCS), which comprises histidine kinases (HKs), histidine phosphotransfer proteins (HPs), and response regulators (RRs), plays essential roles in regulating plant growth, development, and response to various environmental stimuli. TCS genes have been comprehensively identified in various plants, while studies on the genome-wide identification and analysis of TCS in sweet potato were still not reported. Therefore, in this study, a total of 90 TCS members consisting of 20 HK(L)s, 11 HPs, and 59 RRs were identified in the genome of *Ipomoea batatas*. Furthermore, their gene structures, conserved domains, and phylogenetic relationships were analyzed in detail. Additionally, the gene expression profiles in various organs were analyzed, and response patterns to adverse environmental stresses were investigated. The results showed that these 90 TCS genes were mapped on 15 chromosomes with a notably uneven distribution, and the expansion of TCS genes in sweet potato was attributed to both segmental and tandem duplications. The majority of the TCS genes showed distinct organ-specific expression profiles, especially in three types of roots (stem roots, fibrous roots, tuberous roots). Moreover, most of the TCS genes were either induced or suppressed upon treatment with abiotic stresses (drought, salinity, cold, heat) and exogenous phytohormone abscisic acid (ABA). In addition, the yeast-two hybrid system was used to reveal the HK-HP-RR protein-protein interactions. IbHP1, IbHP2, IbHP4, and IbHP5 could interact with three HKs (IbHK1a, IbHK1b, and IbHK5), and also interact with majority of the type-B RRs (IbRR20–IbRR28), while no interaction affinity was detected for IbHP3. Our systematic analyses could provide insights into the characterization of the TCS genes, and further the development of functional studies in sweet potato.

## 1 Introduction

The two-component system (TCS) mediated protein phosphorylation is a major signal transduction pathway in regulating the response to adverse environmental stimuli in bacteria and plants. This system was originally identified in *Escherichia coli* (Kamberov et al., 1994), which included a membrane-associated histidine protein kinase (HK) and a cytoplasmic response regulator (RR) with a receiver (REC) domain. When bacteria are exposed to stress conditions, the HK proteins primarily sense stress signals and autophosphorylation occurs in the histidine residue (H) of the HK domain. Thereafter, the phosphate is subsequently transferred to an aspartate residue (D) in the REC domain of the RR protein. RRs are transcription factors that can convert the external stimuli into internal signals by activating or suppressing downstream targets (Mizuno, 1997). In eukaryotes, such as yeast and plants, TCS have evolved a more complex multi-step TCS signaling system with additional His phosphotransfer (HP) proteins, which are responsible for the transfer of phosphates between HKs and RRs. The multi-step phosphorelay could provide more approaches for studying the signal transduction pathway, thereby further increasing the complexity and accuracy of its regulation (Huo et al., 2020).

To date, TCS genes have been identified at a genome-wide scale in several plant species, including Arabidopsis (Hwang et al., 2002), rice (Ito and Kurata, 2006; Du et al., 2007), maize (Chu et al., 2011), soybean (Mochida et al., 2010), Chinese cabbage (Liu et al., 2014), tomato (He et al., 2016a), cucumber (He et al., 2016b), and chickpea (Ahmad et al., 2020), among others. Classifications and characterizations of the TCS genes have been well established. The model plant Arabidopsis represents the most studied and widely understood TCS elements in plants. Genome-wide analysis revealed 55 TCS-related genes in Arabidopsis, including 16 AHKs, 6 AHPs, and 33 ARRs. The Arabidopsis HKs are comprised of three subfamilies: AHK, ethylene receptor, and phytochrome. Furthermore, the AHK subfamily contains three cytokinin receptors (AHK2, AHK3, and AHK4), AHK1, AHK5, and CKI1. AHPs (AHP1–AHP5) are characterized by a highly conserved XHQXKGSSXS motif with a His phosphorylation site. However, because AHP6 lacks the conserved His residue, it is known as a pseudo-HP protein. Based on conserved domains and phylogenetic analysis, ARRs could be further classified into four subgroups, type-A, type-B, type-C, and pseudo-RRs (Hwang et al., 2002; Huo et al., 2020).

The TCS signaling pathway plays essential roles in regulating plant growth, development, and response to various environmental stimuli. The cytokinin signaling pathway is a typical representative of TCS signaling system. Studies have shown that three cytokinin receptors act as positive regulators in cytokinin signal transduction. The *ahk2 ahk3 ahk4* triple mutant exhibits reduced sensitivity to exogenous cytokinin, retarded root and leaf growth, and induced reproductive developmental defects (Higuchi et al., 2004; Nishimura et al., 2004; Riefler et al., 2006; Tran et al., 2007; Kinoshita-Tsujimura and Kakimoto, 2014). CKI1 is essential for central cell specification in embryo sacs as well as for vascular patterning (Hejatko et al., 2003; Hejatko et al., 2009; Deng et al., 2010; Yuan et al., 2016; Zhu et al., 2022). AHK5 acts as a negative regulator to control root elongation in an ETR1-dependent ethylene and ABA signaling pathway (Iwama et al., 2007). Additionally, there has been an increasing amount of evidence demonstrating that the TCS signaling pathway is involved in various stress responses. Transcripts of *AHK2*, *AHK3*, and *AHK4* were all rapidly induced by dehydration, and the expression of *AHK2* was upregulated with NaCl and ABA treatments. The expression of *AHK3* was also induced under high salinity and cold stresses. Additional functional studies have revealed that these three cytokinin receptors act as negative regulators in the osmotic stress responses of Arabidopsis (Tran et al., 2007). AHK1 is a classical osmotic stress protein, which acts as positive regulators in drought and salt stress responses (Tran et al., 2007; Wohlbach et al., 2008; Lü et al., 2012). In addition, AHK5 positively regulates salt sensitivity and contributes to biotic pathogen resistance (Pham et al., 2012). Similarly, TCS genes in rice, soybean, and tomato have also been shown to participate in drought, high salinity, and extreme temperature stresses (Firon et al., 2012; Wen et al., 2015).

Sweet potato (*Ipomoea batatas* L.) is an economically important food crop that is widely cultivated. It is an important source of calories, proteins, vitamins, and minerals for humanity (Yan et al., 2022). However, studies on the TCS genes in sweet potato are still limited, and their respective functional roles have not yet been reported. In the present study, the putative TCS genes were identified in the sweet potato genome, and their gene structures, conserved domains, and phylogenetic relationships were analyzed in detail. Additionally, the gene expression profiles in various organs were analyzed, and the response patterns to adverse environmental stresses were investigated. Moreover, the gene duplications and evolutionary patterns of the TCS genes in *I. batatas* and its two wild relatives were determined, and the yeast-two hybrid assay was conducted to reveal the HK-HP-RR protein-protein interactions. Our systematic analyses could provide insight into the characterization of the TCS genes, and further the development of functional studies in sweet potato.

## 2 Materials and methods

### 2.1 Identification of the TCS genes in the sweet potato genome

To identify the TCS genes in the genome of sweet potato (*I. batatas*), database including 64295 whole protein sequences were downloaded primarily from the sweet potato genome website (https://sweetpotao.com/) (Jun et al., 2017). Then the local BLAST+ program in the R language was built based on the protein annotation database. Thereafter, *Arabidopsis* TCS protein sequences were used as seed sequences to conduct the BLAST P search with an expected e-value of 1×10^-3^ as the threshold. The protein sequences of the identified sweet potato TCS members were also used as queries to repeat the BLAST P search to ensure that no additional related genes were missing from the database. Finally, after removing the redundant sequences, all the retrieved TCS protein sequences were further analyzed using the Pfam database (http://pfam.janelia.org/) (Punta et al., 2012), the SMART database (http://smart.embl-heidelberg.de/) (Letunic et al., 2012), and the Conserved Domain Database of the NCBI (http://www.ncbi.nlm.nih.gov/Structure/cdd/wrpsb.cgi) (Marchler-Bauer et al., 2011). This was done to eliminate the sequences that did not contain the known conserved domains or motifs of the TCS elements, namely the HK, HATPase, REC, CHASE domain for cytokinin binding (CHASE), ethylene-binding domain (C2H4), and HPt domains. The TCS genes in the genome of two diploid wild relatives of cultivated sweet potato, *Ipomoea trifida* and *Ipomoea trilobal*, were identified using a BLAST P search against the databases of the Sweetpotato Genomics Resource (http://sweetpotato.uga.edu/) (Wu and Lau, 2018) with default expected values. The TCS genes in the genome of *Ipomoea nil* were identified using a BLAST P search against the NCBI databases. Protein sequences of the TCS members from Arabidopsis, rice, maize, tomato, and soybean were then obtained based on the published data.

### 2.2 Gene structure, motif recognition, multiple-sequence alignment, and phylogenetic analyses

The gene structure schematic with exon-intron organizations of the TCS genes was illustrated using the Gene Structure Display Server (http://gsds.gao-lab.org/) (Hu et al., 2015). Furthermore, the conserved motifs of the TCS protein sequences were identified using the online MEME program (https://meme-suite.org/meme/) (Bailey et al., 2015). The deduced amino acid sequences of the conserved HK, HATPase, REC, Myb, and CCT domains were aligned using the Clustal X program. For the phylogenetic relationships of HK, HP, and RR members, the identified HK, HP, and RR protein sequences were separately aligned with a gap opening penalty of 10 and a gap extension penalty of 0.2 using Clustal W in the MEGA7.0 software (http://www.megasoftware.net/) (Kumar et al., 2016). The phylogenetic trees were further constructed using the neighbor-joining (NJ) method, in which Poisson correction, pairwise deletion, and bootstrapping (1,000 replicates; random seeds) were considered as parameters (Liu et al., 2014).

### 2.3 Protein properties and putative promoter region predictions of TCS genes

The molecular weight (MW) and theoretical isoelectric point (pI) of the TCS proteins were calculated using the online tool ExPASy (http://web.expasy.org/compute_pi/) (Wilkins et al., 1999). The subcellular location was predicted with the WoLF PSORT (https://wolfpsort.hgc.jp/) (Horton et al., 2007). The upstream sequences (1,500 bp) of the transcriptional start site of each TCS gene were chosen as the putative promoter regions, and the PlantCARE website (http://bioinformatics.psb.ugent.be/webtools/plantcare/html/search_CARE.html) (Lescot et al., 2002) was used to identify the abiotic stress-related and phytohormone-related *cis*-regulatory elements.

### 2.4 Chromosomal localization, gene duplications, and evolutionary analysis of the TCS genes

The TCS genes were mapped to the corresponding chromosomes based on the sweet potato genome database using the MapInspect software. Tandem duplications were defined if a pair of genes had >40% amino acid sequence similarity and was separated by fewer than five intervening genes (Nuruzzaman et al., 2010). Segmental duplications were analyzed using the MCScanX software with default parameters. The synteny relationship of the TCS genes between *I. batatas* and its wild related species was generated using the Circos software. The occurrence of duplication events and homologous gene divergence, as well as selective pressure on duplicated genes were estimated by calculating synonymous (*Ks*) and non-synonymous substitutions (*Ka*) per site between the duplicated gene pairs using the Codeml procedure of the PAML program (Yang, 1997). The divergence time was calculated at a neutral substitution rate of 1.5 × 10^-8^ substitutions per site per year for the chalcone synthase gene (*Chs*) (Koch et al., 2000).

### 2.5 Plant growth and treatments

The *I. batatas* cultivar Taizhong 6 was used for expression analysis. The plants were grown on an experimental farm at Linyi University. The stem roots, fibrous roots, tuberous roots, stems, leaves, and flowers were separately sampled to analyze the tissue- or organ-specific expression of the TCS genes. All samples were taken from at least 10 plants.

The seedlings of the sweet potato cultivar Taizhong 6 were used for subsequent treatment. Seedlings of a uniform size were grown in Hoagland solution at 28/22°C under a photoperiod of 16 h light/8 h dark in a temperature-controlled greenhouse. About a week later, when the seedlings had 4–5 functional leaves and adventitious roots measuring 8 to 10 cm, they were subjected to stress treatment (Hou et al., 2021). For salt, drought, and ABA treatments, the adventitious roots of the seedlings were submerged in a solution containing 100 mM NaCl, 20% PEG 6000, and 100 µM ABA, respectively. For cold and heat treatments, the seedlings were placed under 10/4°C day/night and 35/35°C day/night conditions, respectively. All roots were separately collected at 0, 1, 6, 12, 24, and 48 h after treatment. All treatments were repeated three times, and each treatment contained at least 20 seedlings. After treatment, all samples were immediately frozen in liquid nitrogen and stored in a refrigerator at -80°C.

### 2.6 RNA extraction and qRT-PCR analysis

RNA extraction, reverse-transcription, and qRT-PCR reactions were performed as previously described (Xiao et al., 2012) using the primers listed in **Supplementary Table S1**. The primers were designed using the Primer (version 5.0) software. The specificity of the reactions was verified *via* melting curve analysis, and the products were further confirmed using agarose gel electrophoresis. qRT-PCR was performed using a Roche LightCycler^®^ 480 II system under the following conditions: 95°C for 15 s; followed by 40 cycles of 95°C for 15 s, 55°C for 15 s, and 72°C for 15 s. There were two biological replicates and three technical replicates for each sample, as well as a negative control using the SYBR Premix ExTaq kit (TOYOBO, Osaka, Japan). The *IbActin* gene was used as the reference gene to study different organs or tissues and developmental stages. Notably, the *IbARF* gene has been used in abiotic stress and plant hormone treatment studies (Sung-Chul et al., 2012). The comparative ΔΔ^CT^ method was used to calculate the relative expression levels of different genes, and a heat map was generated in TBtools using the relative expression data of each gene.

### 2.7 Yeast two-hybrid assays

The matchmaker GAL4 Two-Hybrid System (Clontech) was used to determine the self-activation and protein-protein interactions. The full coding sequences of *IbHK1a*, *IbHK1b*, *IbHK5*, *IbHP1–IbHP5*, and *IbRR20–IbRR28* were amplified using KOD DNA plomerase (TOYOBO) based on the gene sequences with primers listed in **Supplementary Table S2**. *pGADT7* (AD) and *pGBKT7* (BD) vectors were double digested with *Eco*RI and *Bam*HI to prepare the linearized carriers, and then the purified *IbHP1–IbHP5* fragments were inserted into a *pGBKT7* vector with the In-Fusion Cloning Kit (TRANSGEN). The remaining *IbHKs* and *IbRRs* were inserted into the *pGADT7* vertor. After the detection of bait vector self-activation, bait plasmids and prey plasmids were cotransformed into the yeast competent cell AH109 *via* the lithium acetate-mediated transformation method, according to the manufacturer’s instructions. The transformants were primarily cultured in SD/-Leu/-Trp and SD/-Ade/-Leu/-His/-Trp media, and the colonies were cultured in SD/-Ade/-Leu/-His/-Trp medium with X-*α*-gal. *pGBKT7-53* and *pGADT7-T* were used as positive controls, while *pGBKT7-Lam* and *pGADT7-T* were used as negative controls.

## 3 Results

### 3.1 Identification of the TCS genes in *I. batatas* and its wild relatives

BLAST P searches were performed against the local BLAST+ program with the *Arabidopsis* TCS proteins as query sequences. After removing the redundant sequences and conserved domain elimination, 90 TCS members consisting of 20 HK(L)s, 11 HPs, and 59 RRs were identified in the *I. batatas* genome (**Supplementary Table S3**). Similarly, TCS genes in the genome of two wild relatives of cultivated sweet potato, *I. trifida* and *I. trilobal*, were identified. Seventy-one TCS members consisting of 21 HK(L)s, 6 HPs, and 44 RRs were obtained in the *I. trifida* genome (**Supplementary Table S4**); also, 68 TCS members consisting of 21 HK(L)s, 6 HPs, and 41 RRs were obtained in the *I. trilobal* genome (**Supplementary Table S5**). Additionally, 75 TCS members consisting of 21 HK(L)s, 10 HPs, and 44 RRs were retrieved in the genome of *I. nil* (**Supplementary Table S6**), which is in the same genus as cultivated sweet potato.

To show a clear and systematic understanding of the TCS gene numbers in plant genomes, we summarized the TCS genes that have already been identified in several representative plants (**Table 1**). The number of TCS genes in the *I. batatas* genome was only fewer than that in the *Glycine max* genome, which was approximately 1.5 times than that in the *Arabidopsis thaliana* genome. Comparatively, the *I. batatas* genome possessed more pseudo-HPs, type-A RRs, type-C RRs, and pseudo-RRs than *A. thaliana*, but the same amount of HK(L)s and type-B RRs, indicating their preferential gene duplications. Compared with its wild relatives, the *I. batatas* genome possessed more HPs and RRs, but this was not the case for HK(L)s. Interestingly, the large numbers of pseudo-HPs even exceed that of any plant species already reported. Therefore, their evolution mechanisms and possible functions require further investigations.

**Table 1:** Summary of the TCS gene numbers identified in plants. HK(L), HP, and RR represent His-kinase or like, histidine phosphotransfer, and response regulator protein, respectively.

Species	HK(L)	HP (pseudo HP)	Type A RR	Type B RR	Type C RR	Pseudo RR	Total	Reference
*Arabidopsis thaliana*	17 (9)	6 (1)	10	12	2	9	56	Schaller et al., 2008
*Oryza sativa*	11 (3)	5 (3)	13	13	2	8	52	Pareek et al., 2006
*Lotus japonicus*	14	7	7	11	1	5	40	Ishida et al., 2009
*Glycine max*	36 (15)	13	18	15	3	13	98	Mochida et al., 2010
*Zea mays*	11 (3)	9 (2)	16	9	3	11	59	Chu et al., 2011
*Physcomitrella patens*	18	3	7	5	2	4	39	Ishida et al., 2010
*Brassica rapa*	20 (9)	8 (1)	21	17	4	15	85	Liu et al., 2014
*Triticum aestivum*	7	10	41	2	0	2	45	Gahlaut et al., 2014
*Solanum lycopersicum*	20 (11)	6 (2)	7	23	1	8	65	He et al., 2016a
*Cicer arietinum*	10 (8)	5 (2)	7	7	2	10	51	Ahmad et al., 2020
*Cucumis melo*	9 (8)	6 (3)	8	11	0	6	51	Liu et al., 2020
*Zizania latifolia*	21 (4)	5 (3)	14	14	2	6	69	He et al., 2020
*Sorghum bicolor*	13	5 (2)	3	7	2	7	37	Zameer et al., 2021
*Ipomoea batatas*	20 (10)	11 (6)	19	13	7	20	90	This work
*Ipomoea trifida*	21 (10)	6 (1)	13	10	6	15	71	This work
*Ipomoea triloba*	21 (10)	6 (1)	13	9	6	13	68	This work
*Ipomoea nil*	21 (10)	10 (3)	17	10	2	15	75	This work

### 3.2 HK proteins in *I. batatas*

Twenty HK(L) proteins identified in *I. batatas* were named according to the homologous genes in Arabidopsis. The conserved domains, ORF length, protein properties, and subcellular localization were predicted using bioinformatics. To further analyze their structural characteristics and conserved regions, we mapped the gene structures containing exons and introns, and also examined their conserved regions and motifs. Furthermore, their putative protein sequences were aligned and a phylogenetic tree was constructed (**Supplementary Figure S1**, **S2**).

The 20 HK(L) proteins identified in *I. batatas* could be categorized into 10 HKs and 10 HK-likes (HKLs) based on the presence of the conserved His-kinase transmitter (HK) domain. Among these, the 10 HKs were further classified into five subgroups: cytokinin receptor-like HKs, CKI1-like HKs, AHK1-like HKs, AHK5-like HKs, and ethylene receptor-like HKs (**Supplementary Table S3**). All these HKs possessed a conserved HK domain that contains five conserved signature motifs, namely, H, N, G1, F, and G2 (**Supplementary Figure S1**). The four cytokinin receptor-like HKs (IbHK2a, IbHK2b, IbHK3, and IbHK4) comprised HK, HATPase, REC, TM, and CHASE domains. Because the CHASE domain is specific for cytokinin receptor proteins, and necessary for cytokinin binding, this indicates that IbHK2a, IbHK2b, IbHK3, and IbHK4 might function as cytokinin receptors. Additionally, the presence of a TM domain in the cytokinin receptor proteins indicates the plasma membrane location of cytokinin-binding sites (Caesar et al., 2011; Wulfetange et al., 2011). The subcellular localization prediction showed that the four proteins mainly localize in the endoplasmic reticulum (ER) membrane and plasma membrane. The two AHK1-like HKs (IbHK1a, IbHK1b) contained HK, HATPase, REC, and TM domains, and were predicted to be localized in the plasma membrane. Both CKI1-like HK (IbCKI1) and AHK5-like HK (IbHK5) contained only one member with HK, HATPase, and REC; however, the IbHK5 lacked TM domain, and was deemed to possibly localize in the cytoplasm. Two ethylene receptor-like HKs (IbETR1 and IbERS1) contained the TM, C2H4, GAF, and HK domains. IbETR1 possessed the REC domain, while IbERS1 lacked the REC domain, which is consistent with those in other plants. C_2_H_4_ is an ethylene-binding domain, suggesting their potential function as ethylene receptors. Additionally, another 10 HKLs were divided into two subgroups: ethylene receptor-like HKLs and phytochrome-like HKLs, in which the H site of the HK domain is replaced by other amino acids. The five ethylene receptor-like HKLs (IbHKL1-5) were composed of TM, C2H4, GAF, HKL, and REC domains. Another five phytochrome-like HKLs (IbHKL6-10) contained HKL, HATPase, GAF, PHY, and PAS domains. However, all the phytochrome-like HKLs lacked the REC and TM domains, and were predicted to be localized in the cytoplasm and nucleus. The PHY domain is a chromophore-binding domain. The presence of chromophore-binding domains demonstrated that they were candidate proteins involved in phytochromes.

### 3.3 HP proteins in *I. batatas*

Eleven HP proteins were identified in sweet potato with a conserved His-containing phosphotransfer domain (HPt) corresponding to motif 1 (**Supplementary Figure S3**). The ORF lengths of the HP genes ranged from 339 bp to 961 bp, which encoded polypeptides of 113 aa to 287 aa with predicted molecular weights ranging from 12.8 kD to 32.8 kD. Furthermore, the theoretical pI ranged from 4.50 to 9.17. All the HP proteins were predicted to be localized in the cytoplasm and nucleus (**Supplementary Table S3**). Based on the phylogenetic analysis and protein sequence alignment results, these 11 HP proteins could be divided into two groups: HP and pseudo-HP. Five HP proteins (IbHP1-5) had a conserved HPt domain, whereas the other six HP proteins (IbHP6-11) had a pseudo-HPt that lacked the His phosphorylation site (**Supplementary Figure S4**).

### 3.4 RR proteins in *I. batatas*

Fifty-nine RR proteins, including 19 type-A RRs, 13 type-B RRs, 7 type-C RRs, and 20 pseudo-RRs were identified in *I. batatas* (**Table 1**). The type-A RRs (IbRR1-19) had relatively short ORF lengths with only one conserved REC domain corresponding to motifs 1, 2, and 3 as identified using MEME (**Supplementary Figure S5**). The type-B RRs are usually transcription factors characterized by one REC domain in the N terminal end and one Myb domain in the C terminal end. Eleven type-B RRs (IbRR20-30) possessed both the REC domain and Myb domain (motif 4), whereas IbRR31 and IbRR32 had only the REC domain. Seven type-C RRs (IbRR33-39) also possessed only one REC domain with shorter ORF lengths, which is similar to the structures of type-A RRs. In addition, 20 pseudo-RRs (PRRs) could be further divided into two subgroups: Clock PRR and type-B PRR. Compared with authentic RRs, PRRs had a pseudo-REC domain, in which the conserved D site was substituted with other amino acid residues (**Supplementary Figure S6**). Clock PRRs are featured with a CCT domain, which plays an important role in regulating circadian rhythm and controlling flowering time (Hayama and Coupland, 2003). Nine clock PRRs (IbPRR1-9), except for IbPRR1 and IbPRR4, contained the conserved CCT domain (motif 5) and pseudo-REC domain. Eleven pseudo-RRs (IbPRR10-20) were assigned to type-B PRRs. IbPRR10, 11, 12, 13, 16, and 17 possessed both one pseudo-REC domain and one Myb domain, which were predicted to have transcription factor functions. However, IbPRR14, 15, 18, 19, and 20 had only the pseudo-REC domain.

### 3.5 Phylogenetic analysis of plant TCS proteins

**Figure 1:**
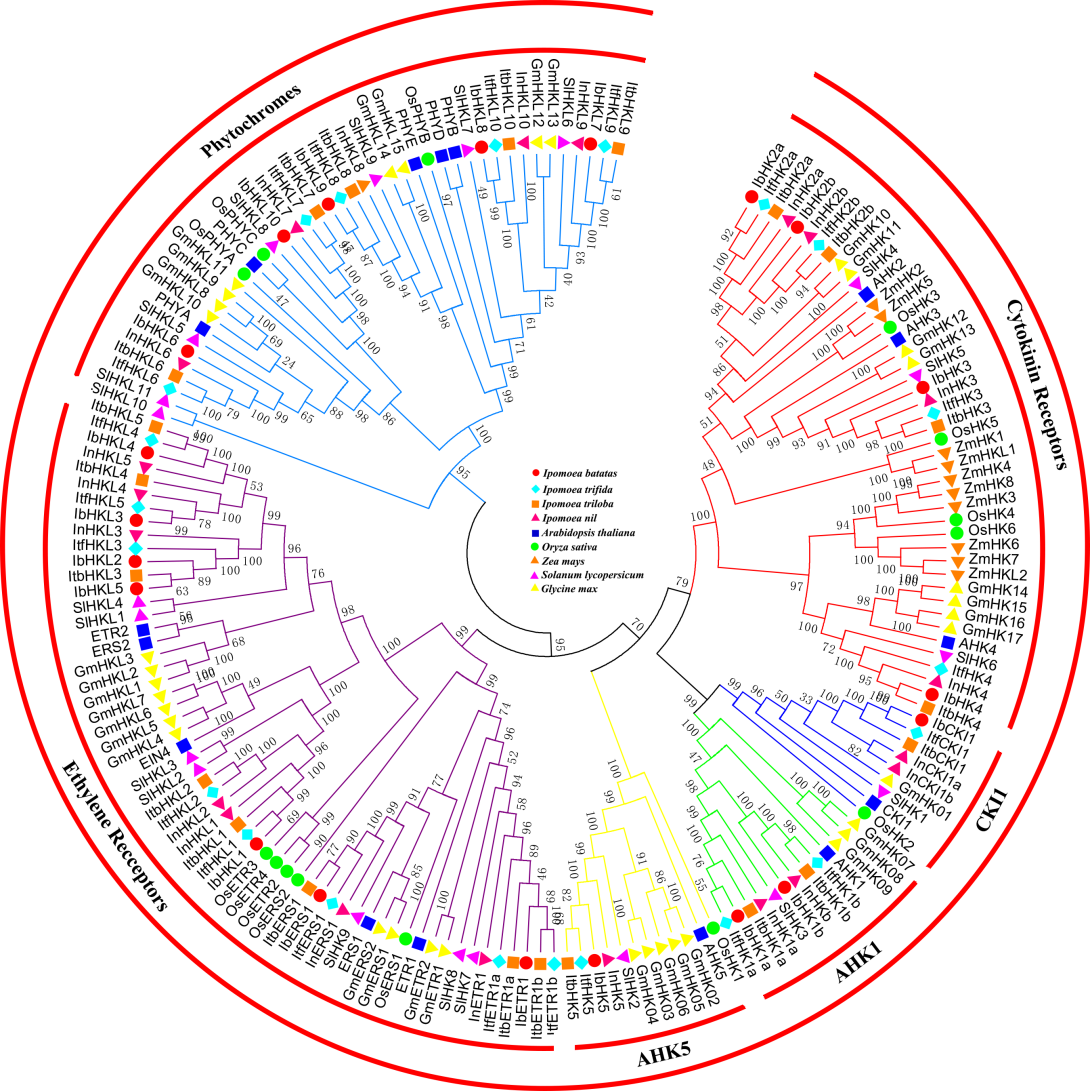
Phylogenetic relationships of HK(L)s in Arabidopsis, rice, maize, tomato, soybean and sweet potato. The phylogenetic tree was constructed using the neighbor-joining method with MEGA 7.0 software. HK(L)s in various species were marked with different labels and diverse subgroups of HK(L)s were highlighted by different colors.

To further reveal the phylogenetic relationships among TCS genes, 179 HK(L)s, 72 HPs, and 373 RRs from Arabidopsis (Hwang et al., 2002), rice (Pareek et al., 2006), maize (Chu et al., 2011), soybean (Mochida et al., 2010), tomato (He et al., 2016a), sweet potato, and sweet potato’s wild relatives were used to perform multiple alignments and construct phylogenetic trees. The 179 HK(L) proteins from the nine land plant species were perfectly divided into six distinct subfamilies (**Figure 1**), namely, cytokinin receptors, CKI1, AHK1, AHK5, ethylene receptors, and phytochromes. This is consistent with previously reported classification results (Hwang et al., 2002; Schaller et al., 2008; Huo et al., 2020). Each subfamily contained representative HK(L) proteins from the nine species, indicating that the subfamilies were evolutionarily conserved and already formed before the divergence of monocotyledon and dicotyledon. *I. batatas* have the nearest relationships with *I. trifida*, *I. trilobal*, and *I.nil*, their HK(L) proteins were clustered in a single clade among each distinct subfamily. Overall, the HK(L) proteins in the *Ipomoea* genus were more closely related to those in dicotyledons than those in monocotyledons.

**Figure 2:**
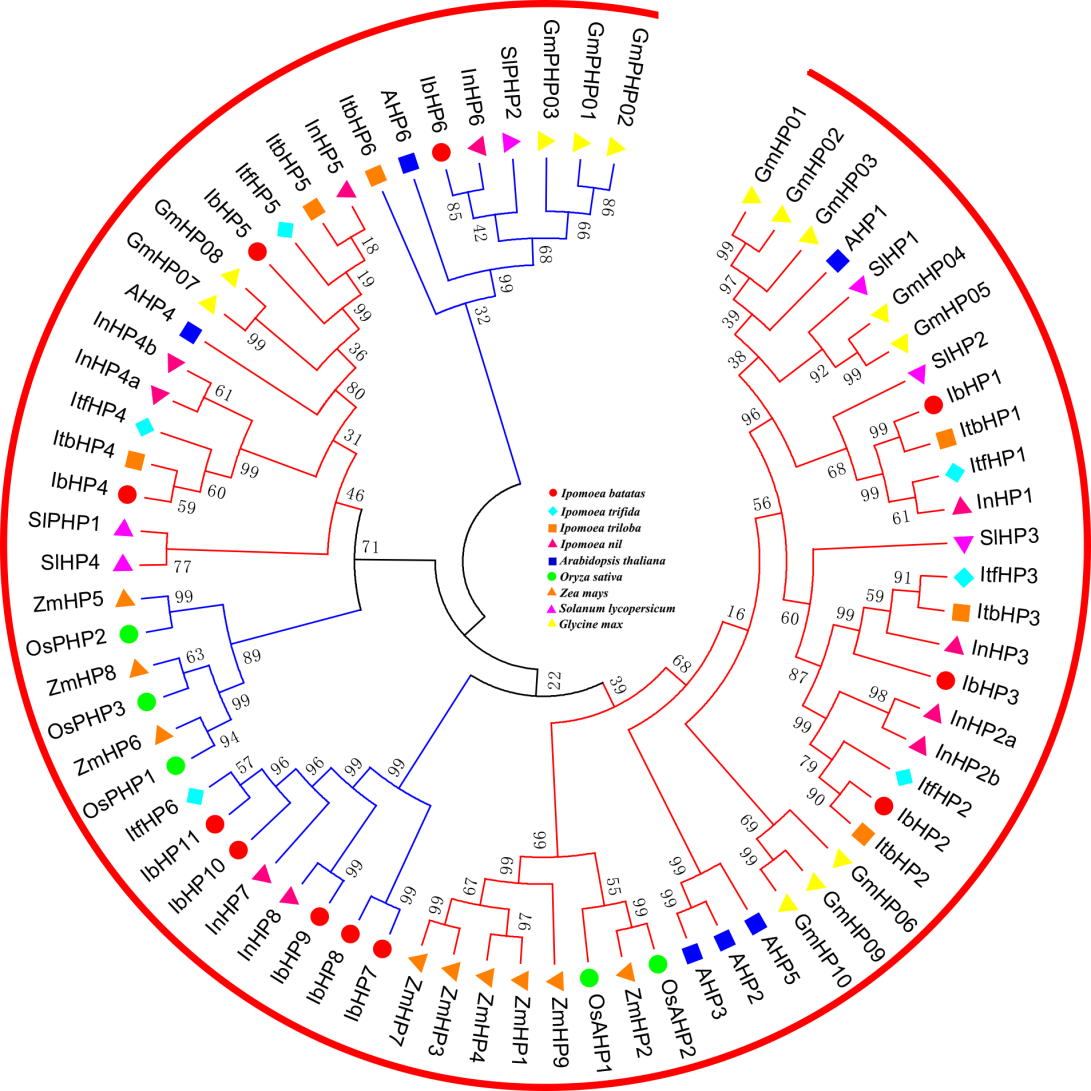
Phylogenetic relationships of HPs in Arabidopsis, rice, maize, tomato, soybean and sweet potato. For other details, see **Figure 1**.

All the authentic and pseudo-HP proteins from these nine species mentioned above were mainly divided into two subfamilies, which were labeled with red and blue lines in **Figure 2**. The authentic HP proteins of IbHP1-3 that were homologous to Arabidopsis (AHP1, AHP2, AHP3, and AHP5) occupied an independent clade, which may also act as positive regulators in cytokinin signaling (Hutchison et al., 2006). However, IbHP4 and IbHP5 were clustered into the same group with AHP4, which showed relatively distinct genetic relationships from the other authentic HP proteins. The pseudo-HP proteins showed more scattered distributions in the phylogenetic tree; specifically, the pseudo-HP proteins in the monocotyledon and dicotyledon plants occupied two different clades. Notably, IbHP7-11, together with ItfHP6, InHP7, and InHP8, occupied a single distinct clade, considering these extra members lacked the homologous counterparts in Arabidopsis, their evolutionary mechanisms and functional roles require further investigation.

**Figure 3:**
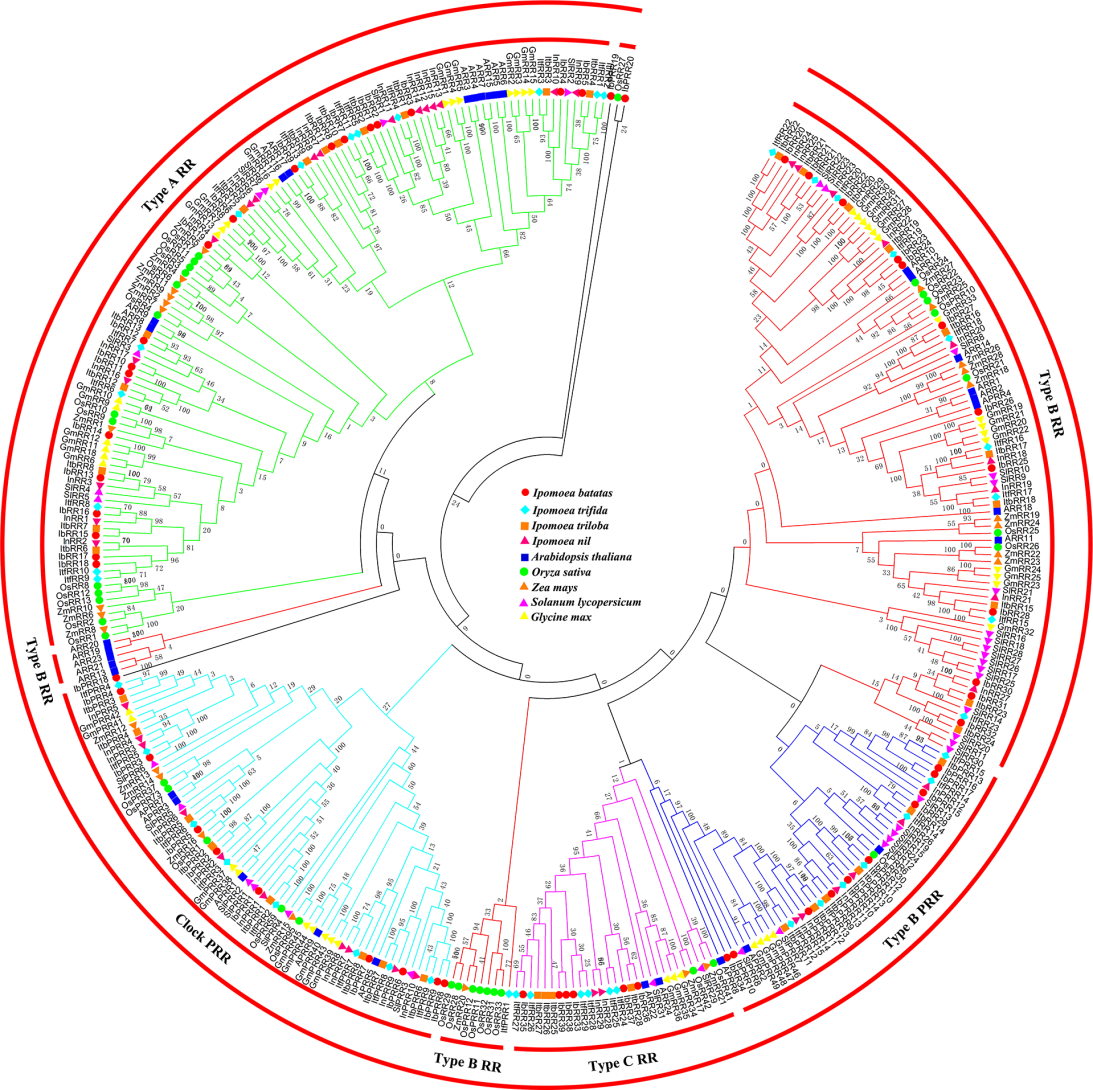
Phylogenetic relationships of RRs in Arabidopsis, rice, maize, tomato, soybean and sweet potato. For other details, see **Figure 1**.

All the RR proteins from these nine species were grouped into four subfamilies: type-A RR, type-B RR, type-C RR, and PRR (**Figure 3**). Type-A RR proteins occupied a single clade, with the majority of the members in a lager subclade and several members from monocotyledon plants in the other clade. Type-B RR proteins could be further subdivided into three subgroups. The type B-I RR subgroup was most predominant, and contained the RRs from all the nine species. The type B-II RR subgroup contained only five Arabidopsis members (ARR13, ARR19, ARR20, ARR21, and ARR23), and no homologs of other plants were clustered in this clade. Previously, we had analyzed the phylogenetic tree containing the species of the Chinese cabbage. While corresponding homologs were identified in Chinese cabbage, no members from rice and soybean were clustered in this subgroup, which was consistent with these results (Liu et al., 2014). We proposed that the type B-II RR subgroup members occurred only after the diversification of Cruciferae and other families. Instead, the type B-III RR subgroup contained several members from the monocotyledon plants, rice and maize, indicating that this group most likely occurred after the diversification of monocotyledon and dicotyledon. Type-C RRs shared a similar structure with type-A RRs, but their genetic distance was not closely related. Previous studies have suggested that type-C RRs might be the oldest RRs, and type-A RRs might have evolved from type-C RRs due to mutations in their promoters (Pils and Heyl, 2009). Pseudo-RR members were mainly divided into two groups, i.e., clock PRRs and type-B PRRs. These Pseudo-RR members had relatively closer phylogenetic relationships with type-B RR members. Similarly, the PRR subfamily contained members from all the nine species. It is important to note that IbPRR18, IbPRR19, and IbPRR20 were not grouped into any subfamily or clade, most likely due to the lower amino acid sequence similarity.

### 3.6 Genomic distribution and gene duplication in sweet potato and its wild relatives

**Figure 4:**
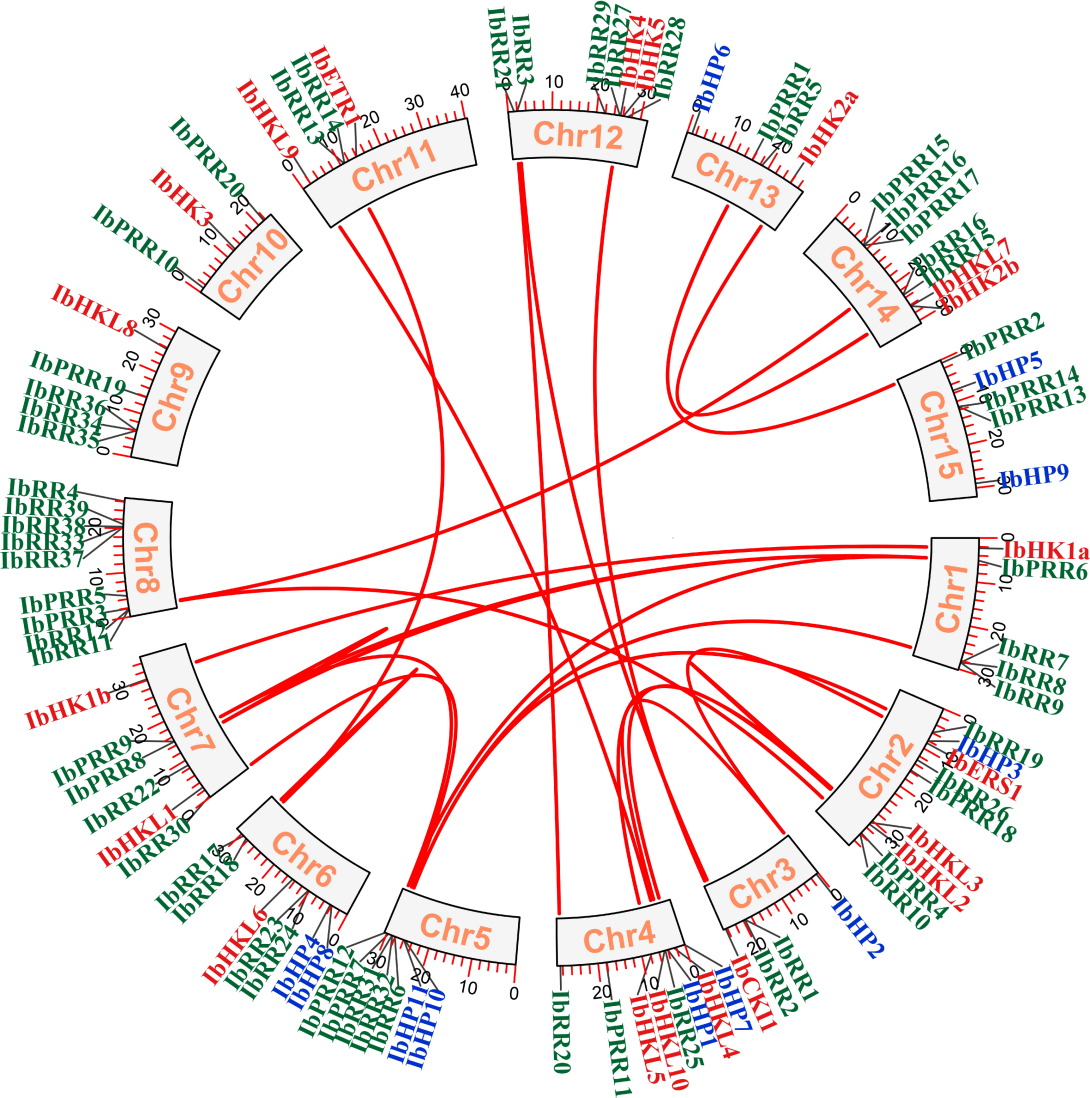
Synteny relationships of duplicated TCS genes in *Ipomoea batatas*. Lines between the chromosomes indicate duplicated gene pairs. Bars located in the chromosomes represent the chromosome length (cM). HK(L)s, HP and RR gene family members were highlighted with different color fonts.

The chromosomal location of each TCS gene was determined based on the genomic information of *I. batatas*, and 90 TCS genes were mapped on the 15 chromosomes with a notably uneven distribution (**Figure 4**, **Supplementary Figure S7**). Among which Chr02, Chr04, Chr05, Chr02, Chr06, Chr08, Chr12, and Chr14 possessed relatively more TCS genes. Gene duplication events play critical roles in gene family evolution, especially the segmental and tandem duplications are the two dominated approaches for gene family members expansion and novel gene functions generation in plants (Cannon et al., 2004; Kong et al., 2007). The gene duplication events were investigated in the sweet potato TCS gene family, and the results revealed 20 segmental duplication gene pairs involved 32 genes, including HK(L)s, HPs, and RRs (**Table 2**, **Supplementary Table S7**). Specifically, five segmental duplication gene pairs that involved nine genes were present in the HK(L) gene subfamily, with a proportion of 45%. Moreover, the percentage of gene numbers involved in segmental duplications for the HP, type-A RR, type-B RR, and pseudo-RR subfamilies were approximately 30%. However, no segmental duplicated genes were found in the type-C RR subfamily. In addition, nine tandem duplication gene pairs that involved 21 genes were also identified (**Supplementary Table S8**). The tandem duplicated genes were only present in the RR subfamily. Most notably, the percentage of gene numbers involved in tandem duplications for the type-A RR and type-C RR subfamilies were 47.4% and 42.9%, respectively. These results confirmed that the expansion of TCS genes in *I. batatas* was mainly attributed to both segmental and tandem duplications. In contrast, the expansion of HK(L)s and HPs mainly depended on segmental duplications, whereas the expansion of type-C RRs mainly depended on tandem duplications.

**Table 2:** Comparisons of segmental and tandem duplicated TCS genes in *Ipomoea batatas*.

Gene subfamily	Number of segmental duplicated gene pairs and involved genes	Percentage of segmental duplicated genes (%)	Number of tandem duplicated gene pairs and involved genes	Percentage of tandem duplicated genes (%)
***HK(L)***	5/9	45	0/0	0
***HP***	2/3	27.3	0/0	0
**Type-A *RR***	6/10	31.6	4/9	47.4
**Type-B *RR***	2/4	30.8	1/2	15.4
**Type-C *RR***	0/0	0	1/3	42.9
**Pseudo *RR***	5/6	30	3/7	35

The synonymous rate (*Ks*), non-synonymous rate (*Ka*), and *Ka/Ks* of these duplicates were calculated, and divergence time was determined using the *Ks* values (**Supplementary Table S7**). The *Ks* values ranged from 0.01278–3.422052, corresponding to the divergence time from 0.423–114.07 MYA (million years ago). The long time span suggests that TCS gene expansion and evolution occurred over millions of years, and not only at specific time points. Furthermore, the *Ka/Ks* values of the segmental duplications were less than 1, indicating that they all underwent purification selection.

**Figure 5:**
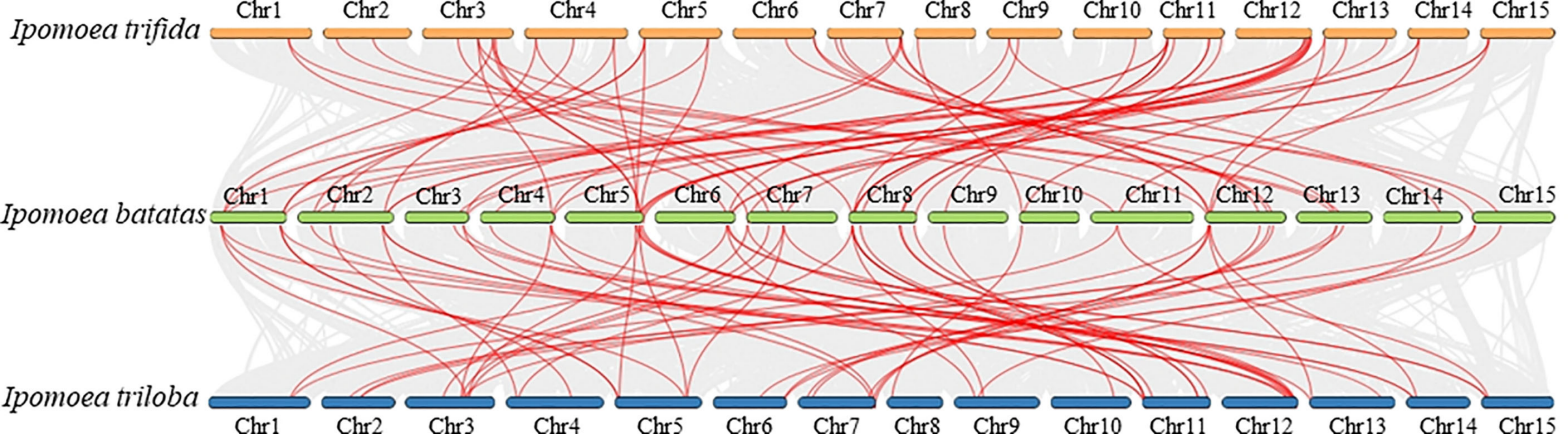
Synteny relationships of duplicated TCS genes in *Ipomoea batatas*, *Ipomoea trifida* and *Ipomoea trilobal*. Lines between the chromosomes indicate duplicated gene pairs.

To further reveal the TCS gene evolution in sweet potato and its two wild relatives, *I. trifida* and *I. trilobal*, the synteny relationships between their TCS genes were analyzed (**Figure 5**). In total, 73 segmental duplicated gene pairs were identified between *I. batatas* and *I. trifida*, and 83 segmental duplicated gene pairs were identified between *I. batatas* and *I. trilobal* (**Supplementary Table S9**, **S10**). This finding indicates their close relationships and conserved evolution. Likewise, the *Ks* and *Ka* modes for these segmental duplicated paralogs were also determined. Results showed the *Ka/Ks* values of the segmental duplications between *I. batatas* and its two wild relatives, *I. trifida* and *I. trilobal*, were all less than 1, indicating that these paralogs also underwent purification selection.

### 3.7 Analysis of *cis*-elements in the putative promoter regions of TCS genes in *I. batatas*

*Cis*-regulatory elements, which are located upstream of genes and act as binding sites for TFs, have essential functions in determining the tissue-specific or stress-responsive expression patterns of genes (Le et al., 2012). To further understand the transcriptional regulation and potential functions of these TCS genes, 1,500 bp regions upstream of the transcriptional start site were selected to identify *cis*-regulatory elements. A number of abiotic stress-related (e.g., drought, high salinity, low temperature, and wound) and hormone-related (e.g., ABA, auxin, ethylene, GA, MeJA, and SA) *cis*-elements were found in the putative promoters of TCS genes in *I. batatas*. These *cis*-elements are counted and classified in **Supplementary Table S11**. The occurrence of these *cis*-elements suggests that these TCS genes might have functions in abiotic stress adaptations and various hormone signaling processes.

### 3.8 Expression profiles of TCS genes in various organs

**Figure 6:**
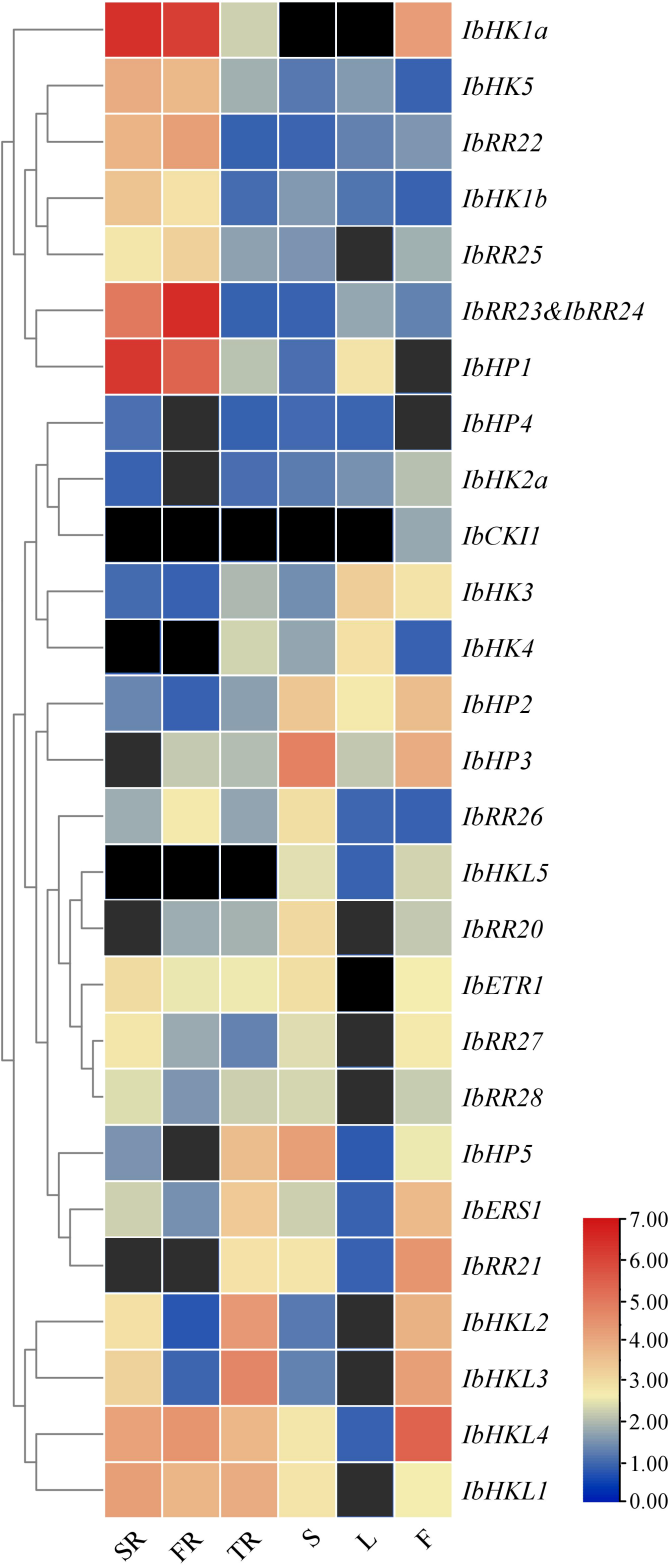
Heat map representation for the organ-specific TCS gene expression profiles in *Ipomoea batatas*. SR, stem roots, FR, fibrous roots, TR, tuberous roots, S, stems, L, leaves and F, flowers. The expression levels of genes are presented using fold-change values transformed to Log^2^ format. The Log^2^ (fold-change values) and the color scale are shown at the bottom of heat map. Genes that were not expressed were shown with black boxes.

To obtain first insight into the functions of TCS genes during the vegetative and reproductive developmental stages of the sweet potato, qRT-PCR was performed to analyze the expression patterns in various organs or tissues such as the stem roots, fibrous roots, tuberous roots, stems, leaves, and flowers (**Supplementary Figure S8**). This study only focused on the HKs, HPs, and type-B RRs. *IbRR23* and *IbRR24* were analyzed together as one gene because they shared high similarity and transcripts were not distinguished by qRT-PCR. The expression profiles of 27 TCS genes were clustered in a heat map, but the transcripts of *IbHK2b* and *IbRR29* were not detected and thus not included here. As shown in **Figure 6**, the majority of the TCS genes showed relatively high expression levels in three types of roots. In particular, the *IbHK1a*, *IbHK5*, *IbRR22*, *IbHK1b*, *IbRR25*, *IbRR23&24*, and *IbHP1* were grouped together and were mainly expressed in both the stem roots and fibrous roots. Consistently, Arabidopsis *HK1* and *HK5* were also mainly expressed in the roots, where functional studies revealed their essential roles in various abiotic stress responses (Tran et al., 2007; Wohlbach et al., 2008; Pham et al., 2012; Kumar et al., 2013). The corresponding Arabidopsis homologs in sweet potato (*IbHK1a*, *IbHK1b*, and *IbHK5*) were also most likely involved in stress adaptations. Moreover, *IbHP5*, *IbERS1*, *IbRR21*, *IbHKL1–IbHKL4* showed relatively higher expression levels in tuberous roots, indicating their potential functions in tuberous root expansion and yield formation. *IbHKL5*, *IbHP2*, *IbHP3*, *IbHP5*, and *IbRR22* were expressed abundantly in stems, while two cytokinin receptors (*IbHK3* and *IbHK4*) exhibited the highest expressions in leaves. *IbCKI1*, *IbERS1*, *IbRR21*, and *IbHKL2–IbHKL4* had relatively higher expressions in flowers, suggesting their potential roles in reproductive development. Notably, *IbCKI1* was exclusively expressed in flowers, and *IbCKI1* was homologous with Arabidopsis *CKI1*. Previous studies showed that *AtCKI1* was expressed almost exclusively in the ovules, and is involved in regulating female gametophyte development (Deng et al., 2010; Yuan et al., 2016). Similar expression profiles may suggest similar functions, thus *IbCKI1* was also proposed to regulate female gametophyte development in sweet potato.

### 3.9 Expression profiles of TCS genes in response to various abiotic stresses

**Figure 7:**
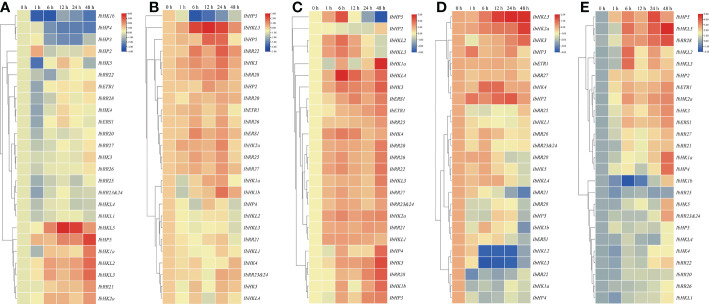
Heat map representation for the response patterns to drought **(A)**, salt stress **(B)**, heat **(C)**, cold stress **(D)**, and exogenous ABA **(E)** treatment TCS genes in *Ipomoea batatas*. The expression levels of genes are presented using fold-change values transformed to Log^2^ format compared to control.

Mounting evidence has shown that TCS is involved in the adaptation of plants to various environmental stresses. Because roots were generally considered as the primary organs involved in these stresses, the above mentioned 27 TCS genes examined were also used to study the response patterns to various abiotic stresses such as drought, high salinity, heat, cold, and ABA in the roots. In total, 25 root-expressed genes showing an abiotic stress response were clustered in a heat map with the exception of two unexpressed genes, *IbCKI1* and *IbHP1*. As shown in **Figure 7A**, seven genes (*IbHKL5*, *IbHP5*, *IbHK1a*, *IbHKL2*, *IbHKL3*, *IbRR21*, and *IbHK2a*) were clustered in a clade, and their expressions were clearly upregulated upon PEG treatment. Meanwhile, another clade contained three genes (*IbHK1b*, *IbHP4*, and *IbHP3*) whose expressions were continuously suppressed and remained at low levels at 48 h. For the high salinity treatment, the transcripts of *IbHP3* were consistently suppressed and even decreased to ~10% of the control level. The expressions of *IbHK1a* and *IbHK1b* were primarily downregulated at 1 h, then continuously upregulated and peaked at 24 h, before decreasing rapidly at 48 h. In contrast, *IbHKL5*, *IbHP5*, *IbRR22*, *IbHK3*, and *IbRR28* were primarily induced with upregulated expressions, then suppressed at 12 h or 24 h, and finally decreased to almost basal levels at 48 h (**Figure 7B**). For the high temperature treatment, most of the TCS genes under examination responded to heat treatment and exhibited upregulated expressions (**Figure 7C**), especially *IbHK1a*, *IbHKL4*, *IbHK3*, *IbHP4*, *IbHK5*, *IbRR28*, *IbHK1b*, and *IbHP3*. This suggests their positive functions under high temperature conditions. However, the *IbHP5* transcript was primarily induced, subsequently repressed after 6 h treatment, and almost undetectable at 48 h. Unlike those for the heat treatment, the response patterns to the cold treatment for the TCS genes seemed to be more diverse. As shown in **Figure 7D**, *IbHKL5*, *IbHK2a, IbHK3*, *IbHK4*, and *IbHP2* responded positively to the heat treatment with notably upregulated expressions. Contrarily, some representative genes, such as *IbRR21*, *IbHK1b*, *IbHKL2*, *IbHKL3*, and *IbRR22*, their transcripts were clearly suppressed with downregulated expressions. Interestingly, the response patterns to the heat and cold treatments of the TCS genes were generally opposite. For example, the transcripts of *IbHK1a*, *IbHK1b*, *IbHK5*, *IbERS1*, *IbHKL2*, *IbHKL3*, *IbHKL4*, *IbHP4*, *IbHP5*, and *IbRR28* were observably induced upon heat treatment but they were suppressed in cold treatment conditions. ABA is an important phytohormone involved in regulating various environmental stress responses. Studies have also suggested the presence of intensive interactions and crosstalk between cytokinins and ABA, as well as their signaling pathways (Ha et al., 2012). In light of this, we also examined the effects of exogenous ABA on the TCS gene expressions in sweet potato. As shown in **Figure 7E**, most of the examined TCS genes were generally upregulated upon ABA treatment, which was consistent with the results that ABA-responsive elements (ABRE) were widely identified in the putative promoters of TCS genes in sweet potato.

### 3.10 The protein-protein interaction network of TCS elements in *I. batatas*

**Figure 8:**
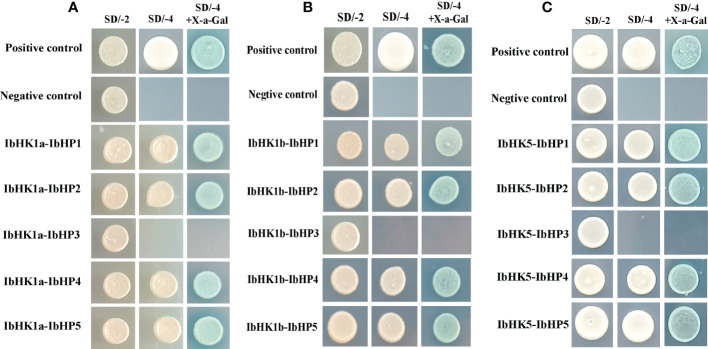
Protein-protein interaction studies among IbHKs and IbHP1~IbHP5 with yeast-two hybrid assays. **(A)** Interactions between IbHK1a and IbHP1~IbHP5, **(B)** Interactions between IbHK1b and IbHP1~IbHP5, **(C)** Interactions between IbHK5 and IbHP1~IbHP5.

**Figure 9:**
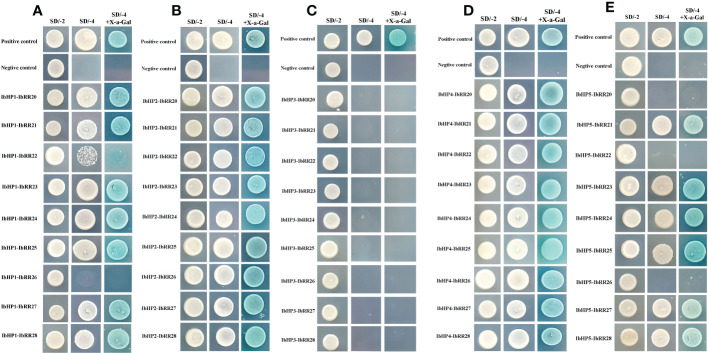
Protein-protein interaction studies among IbHP1~IbHP5 and IbRR20~IbRR28 with yeast-two hybrid assays. **(A)** Interactions between IbHP1 and IbRR20~IbRR28, **(B)** Interactions between IbHP2 and IbRR20~IbRR28, **(C)** Interactions between IbHP3 and IbRR20~IbRR28, **(D)** Interactions between IbHP4 and IbRR20~IbRR28, **(E)** Interactions between IbHP5 and IbRR20~IbRR28.

**Figure 10:**
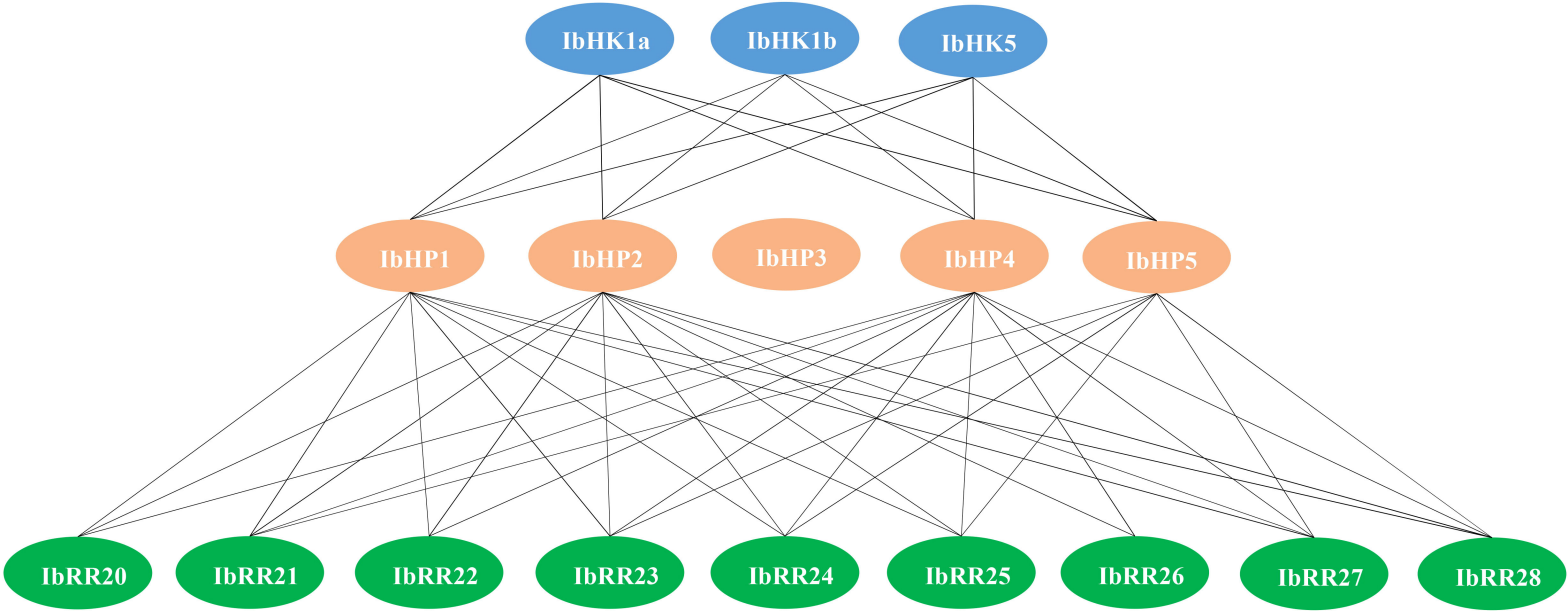
Schematic presentation of interacted TCS proteins in *Ipomoea batatas*. Lines indicate the direct protein-protein Interactions detected with yeast-two hybrid assays in this study.

Since the discovery of the two-component multi-step phosphorylation system in plants, the protein-protein interactions of the TCS elements were investigated to reveal the phosphorylation signal transduction network in Arabidopsis, rice, and polar (Dortay et al., 2008; Sharan et al., 2017; Héricourt et al., 2019). To gain first insight into the interaction network of the TCS proteins in sweet potato, three osmosensing HKs (IbHK1a, IbHK1b, IbHK5) involved in the abiotic stress response were chosen to identify the downstream HPs-RRs counterparts using yeast two hybrid assays. The bait vector self-activation was primarily detected but no self-activation was detected. HK-HP interactions were first studied, and the results showed that IbHK1a, IbHK1b, and IbHK5 could interact with IbHP1, IbHP2, IbHP4, and IbHP5; no interactions were detected for IbHP3 (**Figure 8**). The interaction assays between five HPs (IbHP1–IbHP5) and nine type-B RRs (IbRR20–IbRR28) were also conducted. As shown in **Figure 9**, IbHP1 could interact with IbRR20–IbRR25 and also IbRR27–IbRR28, even though only relatively weak interaction activities were detected between IbHP1 and IbRR22. However, no interactions were detected between IbHP1 and IbRR26. Moreover, no interaction was detected between IbHP5 and IbRR20, IbRR22, and IbRR26, but IbHP5 could interact well with the other six type-B RRs. Additionally, IbHP2 and IbHP4 could interact with all nine type-B RRs while showing strong interaction activities. Remarkably, interactions were never detected between IbHP3 and type-B RRs with repeated trials. Considering that no interaction activity was detected between both upstream HKs and downstream type-B RRs, we assumed that IbHP3 was most likely not involved in the TCS signal transduction system in sweet potato. Based on these results, a protein-protein interaction map was created to present a set of IbHK-IbHP-IbRR phosphorylation signal transduction pathways in sweet potato (**Figure 10**).

## 4 Discussions

In this study, 90 TCS members consisting of 20 HK(L)s, 11 HPs, and 59 RRs were identified in the sweet potato (*I. batatas*) genome. Furthermore, 71 and 68 TCS members were found in its two wild relatives, *I. trifida* and *I. trilobal*. In addition, 75 TCS members existed in the genome of *I. nil*. According to previous studies, the calculated genome size of *I. batatas*, *I. trifida*, *I. trilobal*, and *I. nil* is 851.4 Mb, 476.4 Mb, 495.9 Mb, and 734.8 Mb, respectively (Hirakawa et al., 2015; Hoshino et al., 2016; Yang and Moeinzadeh, 2017; Wu and Lau, 2018; Li et al., 2019; Yan et al., 2022). The *I. batatas* genome possessed relatively more genes than the other three *Ipomoea* genus relatives, which was consistent with their genome size. Comparatively, the *I. batatas* genome possessed more HPs, RRs, and particularly large numbers of pseudo-HPs, but not more HK(L)s.

To further reveal the mechanisms of TCS gene family members expansion in *I. batatas* genome, gene duplication events were investigated. Twenty segmental duplicated gene pairs involved 32 genes and nine tandem duplicated gene pairs involved 21 genes were identified, suggesting that the expansion of TCS genes in *I. batatas* was attributed to both segmental and tandem duplications. He et al. (2016a) analyzed the gene duplications among TCS genes in tomato, and also found that segmental duplication and tandem duplication events both contributed to the expansion of these TCS gene members (He et al., 2016a). However, 10 segmental duplicated gene pairs were identified in Arabidopsis, accounting for 35.71% of the total TCS genes. Furthermore, 66 out of the 98 TCS genes in soybean were involved in segmental duplication events, whereas the tandem duplication of TCS genes was not found in Arabidopsis or soybean (Mochida et al., 2010; Liu et al., 2014). Similarly, in Chinese cabbage, 61 of the 85 TCS genes were found to be segmental duplications, whereas only one tandem duplicated gene pair was found (Hirakawa et al., 2015). These results suggest that segmental duplication was the main mechanism of TCS gene expansion in Arabidopsis, Chinese cabbage, and soybean. Notably, only three pairs of tandem duplications and one pair of segmental duplications were identified in the Cucurbitaceae crops, cucumber and watermelon (He et al., 2016b), which indicates that mechanisms of TCS gene expansions in various plants may be quite distinctive.

*I. trifida* is assumed to be the diploid progenitor of *I. batatas*, after the B2 subgenome specialization at an estimated 1.3 MYA, *I. batatas* alone underwent two whole-genome duplication events at approximately 0.8 MYA and 0.5 MYA (Yang and Moeinzadeh, 2017). We analyzed the synteny relationships of TCS genes between *I. batatas* and *I. trifida*, and found 73 segmental duplicated gene pairs. This demonstrates that the evolution of TCS genes between these two species was fairly conserved; more importantly, the gene expansions mainly occurred before their specification.

The TCS signaling pathway plays essential roles in regulating plant growth, development, and response to various environmental stimuli. The expression profiles of TCS genes in stem roots, fibrous roots, tuberous roots, stems, leaves, and flowers of the sweet potato were analyzed, and their response patterns under drought, high salinity, heat, cold, and ABA in roots were also conducted. The majority of TCS genes showed relatively high expression levels in roots, which was consistent with TCS gene expression profiles in other plants, such as Arabidopsis (Huo et al., 2020), tomato (He et al., 2016a), soybean (Mochida et al., 2010), and Chinese cabbage (Liu et al., 2014). This is most likely due to the fact that cytokinins are mainly synthesized in the roots. Roots are believed to be the most important primary organs in the response to drought and high salinity stresses. *IbHK1a*, *IbHK5*, *IbRR22*, *IbHK1b*, *IbRR25*, *IbRR23&24*, and *IbHP1* were grouped together and were mainly expressed in the stem roots and fibrous roots. In fact, Arabidopsis *HK1* and *HK5* were also mainly expressed in roots, and functional studies revealed their essential roles in various abiotic stress responses (Tran et al., 2007; Wohlbach et al., 2008; Pham et al., 2012; Kumar et al., 2013). Coincidentally, transcripts of homologous genes in sweet potato could also be induced or suppressed with PEG or NaCl treatment, suggesting their potential functional roles in environmental stress adaptations. Our expression analysis results could provide valuable information to further elucidate the functional roles of sweet potato TCS genes under abiotic stress conditions.

Information on the protein-protein interactions of TCS elements was indispensable for the correct or precise phosphorylation signal transduction, protein interaction map was already depicted in Arabidopsis, rice, and polar (Dortay et al., 2008; Sharan et al., 2017; Héricourt et al., 2019). To gain first insight into the protein-protein interaction network of the TCS signal transduction pathway in sweet potato, three osmosensing proteins (IbHK1a, IbHK1b, and IbHK5) were chosen to identify the downstream HPs-RRs counterparts using yeast two hybrid assays. The protein-protein interaction network was schematically presented. The results showed that IbHK1a, IbHK1b, and IbHK5 could interact with IbHP1, IbHP2, IbHP4, and IbHP5, but no interactions were detected for IbHP3. Moreover, interactions were never detected between IbHP3 and nine type-B RRs. Still it remains to be resolved whether IbHP3 could interact with other HK proteins or type-A RRs. Similarly, AHP4 could not interact with any of the AHKs, and is a non-functional protein in Arabidopsis (Dortay et al., 2008). Thus, we proposed that it is likely that IbHP3 was not involved in the TCS signal transduction in sweet potato. In this study, only three osmosensing proteins were chosen for the HK-HP interaction assays in sweet potato, more efforts are needed to include other HKs and type-A RRs in the protein interaction network.

## 5 Conclusion

This study found 90 putative members of the TCS family in the genome of *I. batatas*, including 20 HK(L)s, 11 HPs, and 59 RRs. Additionally, 71, 68, and 75 TCS genes were found in *I. trifida*, *I. trilobal*, and *I. nil*, respectively. A comparative analysis of diverse plant species revealed that the evolution of TCS members was highly conserved. Twenty segmental duplication gene pairs and nine tandem duplication gene pairs were identified, suggesting that both segmental and tandem duplications contributed to the expansion of TCS genes in *I. batatas*, and this expansion occurred before the specification of *I. batatas* and its diploid progenitor *I. trifida*. The majority of TCS genes were specifically or preferentially expressed in certain organs, especially in the three types of roots (stem roots, fibrous roots, tuberous roots). The promoter regions of TCS genes exhibited numerous abiotic stress-related and hormone-related *cis*-elements, indicating their potential roles in abiotic stress adaptations and various hormone signaling processes. Moreover, their response patterns to PEG, NaCl, cold, heat, and exogenous ABA were verified. Protein-protein interactions were conducted among three osmosensing proteins (IbHK1a, IbHK1b, IbHK5), five HPs (IbHP1–IbHP5), and nine type-B RRs (IbRR20–IbRR28); thereafter, the interaction network was preliminary established. These results could provide insight into the characterization of the TCS genes in sweet potato, and further the development of functional studies on these TCS genes under abiotic stress conditions.

## Data availability statement

The original contributions presented in the study are included in the article/**Supplementary Material**. Further inquiries can be directed to the corresponding authors.

## Author contributions

RH performed the experiments, analyzed the data, and drafted the manuscript. YZ and TL participated in qRT-PCR experiments and data analysis. MX, XW, and PX contributed to bioinformatic analysis, XC and SD participated in abiotic stress treatments, ZNL, ZYL, and YH conceived the study and its design and assisted with revisions to the manuscript. All authors contributed to the article and approved the submitted version.
