# Supplementary material for: Genome-wide identification and expression analysis of two-component system genes in sweet potato (Ipomoea batatas L.)
Source: Front Plant Sci. 2023 Jan 12;13:1091620. doi: 10.3389/fpls.2022.1091620 (PMC9878860; doi:10.3389/fpls.2022.1091620)
Supplement: Supplementary file 1 [file DataSheet_1.zip › Supplementary Table S10. Segmental duplicated TCS genes Ipomoea batatas and Ipomoea triloba.docx]

Table S10. Segmental duplicated TCS genes *Ipomoea batatas* and *Ipomoea trilobal*.

| **Number** | **Gene I** | **Gene II** | ***Ka*** | ***Ks*** | ***Ka/Ks*** |
| --- | --- | --- | --- | --- | --- |
| 1 | ItbHK1b | IbHK1a | 0.103633 | 0.638911 | 0.162202 |
| 2 | ItbHK1a | IbHK1a | 0.00469 | 0.049549 | 0.094647 |
| 3 | ItbHK1b | IbHK1b | 0.001858 | 0.039424 | 0.047138 |
| 4 | ItbHK1a | IbHK1b | 0.102578 | 0.667563 | 0.153661 |
| 5 | ItbHK2b | IbHK2a | 0.140802 | 0.587327 | 0.239733 |
| 6 | ItbHK2b | IbHK2b | 0.034755 | 0.07626 | 0.455749 |
| 7 | ItbHK3 | IbHK3 | 0.011652 | 0.037203 | 0.313192 |
| 8 | ItbHK4 | IbHK4 | 0.013718 | 0.094082 | 0.145809 |
| 9 | ItbHK5 | IbHK5 | 0.003481 | 0.02463 | 0.141322 |
| 10 | ItbCKI1 | IbCKI1 | 0.011975 | 0.026597 | 0.450248 |
| 11 | ItbETR1b | IbETR1 | 0.194266 | 0.353608 | 0.549382 |
| 12 | ItbERS1 | IbERS1 | 0.021122 | 0.065802 | 0.320988 |
| 13 | ItbHKL1 | IbHKL1 | 0.004735 | 0.082159 | 0.057634 |
| 14 | ItbHKL3 | IbHKL2 | 0.004009 | 0.053137 | 0.07545 |
| 15 | ItbHKL3 | IbHKL3 | 0.005974 | 0.049248 | 0.12131 |
| 16 | ItbHKL3 | IbHKL5 | 0.166264 | 0.890493 | 0.18671 |
| 17 | ItbHKL6 | IbHKL6 | 0.020835 | 0.045048 | 0.462505 |
| 18 | ItbHKL8 | IbHKL8 | 0.002291 | 0.052683 | 0.043479 |
| 19 | ItbHKL9 | IbHKL9 | 0.00153 | 0.025079 | 0.061015 |
| 20 | ItbHKL10 | IbHKL9 | 0.091344 | 0.704943 | 0.129577 |
| 21 | ItbHKL10 | IbHKL10 | 0.008036 | 0.034194 | 0.235021 |
| 22 | ItbHKL9 | IbHKL10 | 0.091796 | 0.687759 | 0.133471 |
| 23 | ItbHP1 | IbHP1 | 0.050326 | 0.048248 | 1.043067 |
| 24 | ItbHP2 | IbHP1 | 0.234149 | 2.142353 | 0.109295 |
| 25 | ItbHP1 | IbHP2 | 0.231364 | 1.890393 | 0.122389 |
| 26 | ItbHP2 | IbHP2 | 0 | 0.041106 | 0 |
| 27 | ItbHP3 | IbHP2 | 0.095461 | 0.779829 | 0.122413 |
| 28 | ItbHP2 | IbHP3 | 0.083447 | 0.48416 | 0.172354 |
| 29 | ItbHP3 | IbHP3 | 0.003755 | 0.109 | 0.034447 |
| 30 | ItbHP4 | IbHP4 | 0.01049 | 0.014019 | 0.748242 |
| 31 | ItbHP4 | IbHP5 | 0.256951 | 2.727202 | 0.094218 |
| 32 | ItbHP5 | IbHP5 | 0 | 0.021054 | 0 |
| 33 | ItbRR1 | IbRR1 | 0.154677 | 1.037512 | 0.149085 |
| 34 | ItbRR2 | IbRR1 | 0 | 0.037625 | 0 |
| 35 | ItbRR2 | IbRR2 | 0.005753 | 0.057171 | 0.100623 |
| 36 | ItbRR2 | IbRR3 | 0.188301 | 1.007732 | 0.186856 |
| 37 | ItbRR1 | IbRR3 | 0.062034 | 0.165444 | 0.374957 |
| 38 | ItbRR3 | IbRR4 | 0.020193 | 0.043923 | 0.459741 |
| 39 | ItbRR4 | IbRR5 | 0.054964 | 0.05457 | 1.007225 |
| 40 | ItbRR5 | IbRR6 | 0 | 0.042414 | 0 |
| 41 | ItbRR9 | IbRR6 | 0.232342 | 0.940671 | 0.246996 |
| 42 | ItbRR5 | IbRR7 | 0.236946 | 1.285839 | 0.184274 |
| 43 | ItbRR11 | IbRR7 | 0.082367 | 0.109371 | 0.753099 |
| 44 | ItbRR12 | IbRR10 | 0.231915 | 1.894098 | 0.122441 |
| 45 | ItbRR12 | IbRR11 | 0.017267 | 0.085641 | 0.201621 |
| 46 | ItbRR13 | IbRR12 | 0 | 0.060334 | 0 |
| 47 | ItbRR8 | IbRR14 | 0.079447 | 0.132091 | 0.601456 |
| 48 | ItbRR6 | IbRR14 | 0.313314 | 0.955877 | 0.327776 |
| 49 | ItbRR7 | IbRR15 | 0.036521 | 0.11735 | 0.31121 |
| 50 | ItbRR6 | IbRR17 | 0.007244 | 0.019089 | 0.379498 |
| 51 | ItbRR7 | IbRR18 | 0.154191 | 0.619481 | 0.248903 |
| 52 | ItbRR6 | IbRR18 | 0.009197 | 0.019232 | 0.478214 |
| 53 | ItbRR8 | IbRR18 | 0.213864 | 0.651903 | 0.32806 |
| 54 | ItbRR5 | IbRR19 | 0.235227 | 1.349606 | 0.174293 |
| 55 | ItbRR20 | IbRR20 | 0.229715 | 0.793704 | 0.289421 |
| 56 | ItbRR20 | IbRR21 | 0.227683 | 0.755033 | 0.301554 |
| 57 | ItbRR20 | IbRR22 | 0.013486 | 0.02895 | 0.465829 |
| 58 | ItbRR15 | IbRR28 | 0.005469 | 0.033872 | 0.161453 |
| 59 | ItbRR14 | IbRR29 | 0.065746 | 0.098664 | 0.666358 |
| 60 | ItbRR24 | IbRR30 | 0.651004 | 2.199277 | 0.296008 |
| 61 | ItbRR24 | IbRR32 | 0.053031 | 0.090966 | 0.582975 |
| 62 | ItbRR26 | IbRR35 | 0.077992 | 0.190844 | 0.40867 |
| 63 | ItbRR28 | IbRR38 | 0.118597 | 0.341585 | 0.347197 |
| 64 | ItbRR28 | IbRR38 | 0.118597 | 0.341585 | 0.347197 |
| 65 | ItbRR28 | IbRR39 | 0.257176 | 0.364868 | 0.704846 |
| 66 | ItbPRR1 | IbPRR1 | 0 | 0.022317 | 0 |
| 67 | ItbPRR2 | IbPRR1 | 0.435153 | 3.255252 | 0.133677 |
| 68 | ItbPRR1 | IbPRR2 | 0.430754 | 2.779934 | 0.154951 |
| 69 | ItbPRR2 | IbPRR2 | 0.002919 | 0.029488 | 0.09899 |
| 70 | ItbPRR4 | IbPRR3 | 0.026858 | 0.063852 | 0.420627 |
| 71 | ItbPRR4 | IbPRR4 | 0.262567 | 1.004876 | 0.261293 |
| 72 | ItbPRR5 | IbPRR5 | 0.018674 | 0.049568 | 0.376744 |
| 73 | ItbPRR6 | IbPRR6 | 0.210322 | 1.000322 | 0.210254 |
| 74 | ItbPRR8 | IbPRR6 | 0.238354 | 0.8506 | 0.280219 |
| 75 | ItbPRR6 | IbPRR7 | 0.258269 | 0.759054 | 0.340252 |
| 76 | ItbPRR7 | IbPRR7 | 0.24192 | 0.834014 | 0.290067 |
| 77 | ItbPRR8 | IbPRR7 | 0.043132 | 0.091185 | 0.473011 |
| 78 | ItbPRR6 | IbPRR8 | 0.01396 | 0.003459 | 4.035506 |
| 79 | ItbPRR7 | IbPRR8 | 0.229921 | 0.973679 | 0.236136 |
| 80 | ItbPRR8 | IbPRR8 | 0.270791 | 0.811815 | 0.333562 |
| 81 | ItbPRR11 | IbPRR10 | 0.017126 | 0.075287 | 0.22747 |
| 82 | ItbPRR10 | IbPRR12 | 0.020497 | 0.027317 | 0.750347 |
| 83 | ItbPRR9 | IbPRR14 | 0.162157 | 0.230738 | 0.702777 |
