# Supplementary material for: Genome-wide identification and expression analysis of two-component system genes in sweet potato (Ipomoea batatas L.)
Source: Front Plant Sci. 2023 Jan 12;13:1091620. doi: 10.3389/fpls.2022.1091620 (PMC9878860; doi:10.3389/fpls.2022.1091620)
Supplement: Supplementary file 1 [file DataSheet_1.zip › Supplementary Table S3. TCS proteins in Ipomoea batatas.docx]

Table S3. TCS proteins in *Ipomoea batatas* genome.

| **Gene name** | **Gene locus** | **Domains** | **Chr** | **position** | **ORF length (bp)** | **Deduced polypeptide** | | | **Subcellular**  **localization** |
| --- | --- | --- | --- | --- | --- | --- | --- | --- | --- |
|  |  |  |  |  |  | Length  (aa) | MW  (kDa) | PI |  |
| **HKs** | | | | | | | | | |
| IbHK1a | g423 | HK, Rec | Chr01 | 2305130-2310710 (+) | 3624 | 1208 | 135.2 | 6.32 | Plasma membrane |
| IbHK1b | g29410 | HK, Rec | Chr07 | 30276506-30281997 (+) | 3504 | 1168 | 130.1 | 6.48 | Plasma membrane |
| IbHK2a | g54785 | CHASE, HK, Rec | Chr13 | 25905124-25912718 (+) | 3891 | 1297 | 146.1 | 7.03 | Endoplasmic Reticulum and Membrane |
| IbHK2b | g59045 | CHASE, HK, Rec | Chr14 | 26255134-26263998 (-) | 4587 | 1529 | 173.0 | 6.89 | Endoplasmic Reticulum and Membrane |
| IbHK3 | g39874 | CHASE, HK, Rec | Chr10 | 12469737-12475142 (+) | 2844 | 948 | 106.8 | 6.14 | Endoplasmic Reticulum and Membrane |
| IbHK4 | g50268 | CHASE, HK, Rec | Chr12 | 25439409-25448111 (-) | 3912 | 1304 | 143.1 | 6.12 | Endoplasmic Reticulum and Membrane |
| IbHK5 | g50349 | HK, Rec | Chr12 | 26079456-26086040 (-) | 3075 | 1025 | 115.0 | 5.21 | Cytoplasm and Membrane |
| IbCKI1 | g12703 | HK, Rec | Chr03 | 24400978-24403728 (-) | 2262 | 754 | 82.8 | 6.35 | Endoplasmic Reticulum |
| IbETR1 | g43417 | GAF, HK, Rec | Chr11 | 13971506-13975052 (+) | 2019 | 673 | 75.8 | 6.44 | Endoplasmic Reticulum |
| IbERS1 | g5501 | GAF, HK | Chr02 | 8596515-8599246 (-) | 1824 | 608 | 68.0 | 6.59 | Endoplasmic Reticulum |
| IbHKL1 | g25719 | GAF, HKL, Rec | Chr07 | 2780274-2784463 (+) | 2199 | 733 | 81.8 | 8.73 | Endoplasmic Reticulum |
| IbHKL2 | g8401 | GAF, HKL, Rec | Chr02 | 31037444-31039793 (-) | 2208 | 736 | 84.4 | 8.45 | Endoplasmic Reticulum |
| IbHKL3 | g8333 | GAF, HKL, Rec | Chr02 | 30589913-31037370 (+) | 2187 | 729 | 80.7 | 8.56 | Endoplasmic Reticulum |
| IbHKL4 | g13146 | GAF, HKL, Rec | Chr04 | 2542127-2574883 (-) | 2232 | 744 | 83.1 | 7.77 | Endoplasmic Reticulum |
| IbHKL5 | g13882 | GAF, HKL, Rec | Chr04 | 7798882-7802074 (+) | 2493 | 831 | 91.7 | 8.69 | Endoplasmic Reticulum |
| IbHKL6 | g22825 | PHY, HKL | Chr06 | 14640878-14645847 (+) | 3141 | 1047 | 115.9 | 5.81 | Cytoplasm and Nucleus |
| IbHKL7 | g59038 | PHY, HKL | Chr14 | 26221257-26226194 (+) | 3108 | 1036 | 114.9 | 5.76 | Cytoplasm and Nucleus |
| IbHKL8 | g37417 | PHY, HKL | Chr09 | 25659784-25659926 (+) | 3393 | 1131 | 126.0 | 5.85 | Cytoplasm and Nucleus |
| IbHKL9 | g41655 | PHY, HKL | Chr11 | 2006035-2010315 (-) | 3390 | 1130 | 125.3 | 5.68 | Cytoplasm and Nucleus |
| IbHKL10 | g13495 | PHY, HKL | Chr04 | 4726314-4730969 (+) | 3408 | 1136 | 125.7 | 6.69 | Cytoplasm and Nucleus |
| **HPs** | | | | | | | | | |
| IbHP1 | g13370 | HPt | Chr04 | 3993354-3995422 (+) | 528 | 176 | 19.7 | 5.25 | Cytoplasm and Nucleus |
| IbHP2 | g9509 | HPt | Chr03 | 498980-501508 (-) | 459 | 153 | 17.2 | 4.85 | Cytoplasm and Nucleus |
| IbHP3 | g5431 | HPt | Chr02 | 8107648-8108472 (+) | 339 | 113 | 12.8 | 5.02 | Cytoplasm and Nucleus |
| IbHP4 | g21441 | HPt | Chr06 | 4359215-4360236 (+) | 363 | 121 | 13.9 | 4.66 | Cytoplasm and Nucleus |
| IbHP5 | g61029 | HPt | Chr15 | 7409452-7410972 (-) | 453 | 151 | 17.5 | 8.27 | Cytoplasm and Nucleus |
| IbHP6 | g51309 | Pseudo-HPt | Chr13 | 1151891-1155630 (+) | 861 | 287 | 32.8 | 9.17 | Cytoplasm and Nucleus |
| IbHP7 | g12777 | Pseudo-HPt | Chr04 | 39655-47058 (-) | 537 | 179 | 20.1 | 4.50 | Cytoplasm and Nucleus |
| IbHP8 | g34846 | Pseudo-HPt | Chr06 | 4028412-4029465 (+) | 537 | 179 | 20.2 | 4.59 | Cytoplasm and Nucleus |
| IbHP9 | g63864 | Pseudo-HPt | Chr15 | 28980014-28982476 (-) | 441 | 147 | 16.6 | 5.35 | Cytoplasm and Nucleus |
| IbHP10 | g20059 | Pseudo-HPt | Chr05 | 25610291-25611911 (+) | 441 | 147 | 16.7 | 6.29 | Cytoplasm and Nucleus |
| IbHP11 | g20101 | Pseudo-HPt | Chr05 | 25877932-25879642 (-) | 441 | 147 | 16.7 | 6.12 | Cytoplasm and Nucleus |
| **Type A RRs** | | | | | | | | | |
| IbRR1 | g12608 | Rec | Chr03 | 19589858-19594234 (+) | 1332 | 444 | 49.0 | 8.52 | Nucleus |
| IbRR2 | g12082 | Rec | Chr03 | 19590810-19594233 (+) | 894 | 298 | 32.5 | 5.16 | Nucleus |
| IbRR3 | g47182 | Rec | Chr12 | 2042957-2045570 (+) | 897 | 299 | 33.0 | 6.15 | Nucleus |
| IbRR4 | g33858 | Rec | Chr08 | 26080908-26084506 (+) | 1281 | 427 | 47.0 | 7.27 | Nucleus |
| IbRR5 | g53645 | Rec | Chr13 | 18448713-18450504 (+) | 705 | 235 | 25.5 | 4.65 | Nucleus |
| IbRR6 | g20447 | Rec | Chr05 | 28055410-28056402 (+) | 441 | 147 | 16.3 | 5.93 | Nucleus |
| IbRR7 | g3932 | Rec | Chr01 | 28441552-28443015 (+) | 417 | 139 | 15.0 | 4.88 | Nucleus |
| IbRR8 | g3934 | Rec | Chr01 | 28449193-28450769 (+) | 417 | 139 | 15.2 | 5.36 | Nucleus |
| IbRR9 | g3935 | Rec | Chr01 | 28453457-28458640 (+) | 1905 | 635 | 68.9 | 5.36 | Nucleus |
| IbRR10 | g8841 | Rec | Chr02 | 34068284-34068740 (+) | 456 | 152 | 16.3 | 5.40 | Nucleus |
| IbRR11 | g30555 | Rec | Chr08 | 1733351-1734444 (-) | 549 | 183 | 20.1 | 4.81 | Nucleus |
| IbRR12 | g30557 | Rec | Chr08 | 1740589-1741302 (-) | 492 | 164 | 18.3 | 9.06 | Nucleus |
| IbRR13 | g42973 | Rec | Chr11 | 10822581-10824109 (+) | 822 | 274 | 31.0 | 5.46 | Nucleus |
| IbRR14 | g42966 | Rec | Chr11 | 10918510-10920252 (+) | 765 | 255 | 28.8 | 5.11 | Nucleus |
| IbRR15 | g58574 | Rec | Chr14 | 23247738-23248916 (+) | 642 | 214 | 23.9 | 6.91 | Nucleus |
| IbRR16 | g55375 | Rec | Chr14 | 23148339-23149875 (+) | 666 | 222 | 24.9 | 9.34 | Nucleus |
| IbRR17 | g24894 | Rec | Chr06 | 29387376-29389212 (+) | 717 | 239 | 26.5 | 5.22 | Nucleus |
| IbRR18 | g24753 | Rec | Chr06 | 28547430-28552102 (+) | 1590 | 530 | 58.9 | 6.36 | Cytoplasm |
| IbRR19 | g5095 | Rec | Chr02 | 5948342-5949272 (+) | 492 | 164 | 17.8 | 8.69 | Nucleus |
| **Type B RRs** | | | | | | | | | |
| IbRR20 | g16498 | Rec, Myb | Chr04 | 28532514-28536149 (+) | 2034 | 678 | 74.0 | 6.54 | Nucleus |
| IbRR21 | g47091 | Rec, Myb | Chr12 | 1349974-1352928 (+) | 1971 | 657 | 71.8 | 5.90 | Nucleus |
| IbRR22 | g26505 | Rec, Myb | Chr07 | 8751245-8754594 (+) | 1995 | 665 | 72.8 | 5.55 | Cytoplasm |
| IbRR23 | g22427 | Rec, Myb | Chr06 | 10269123-10272556 (-) | 1887 | 629 | 69.8 | 5.45 | Nucleus |
| IbRR24 | g22235 | Rec, Myb | Chr06 | 10249137-10261658 (-) | 1740 | 580 | 65.0 | 5.42 | Cytoplasm |
| IbRR25 | g13465 | Rec, Myb | Chr04 | 4552390-4556175 (+) | 2037 | 679 | 73.8 | 5.83 | Nucleus |
| IbRR26 | g6082 | Rec, Myb | Chr02 | 12975173-12978761 (-) | 2004 | 668 | 73.5 | 5.69 | Nucleus |
| IbRR27 | g50246 | Rec, Myb | Chr12 | 25274804-25278994 (+) | 1857 | 619 | 67.5 | 5.62 | Nucleus |
| IbRR28 | g50506 | Rec, Myb | Chr12 | 27049847-27053161 (+) | 1719 | 573 | 63.7 | 6.11 | Cytoplasm |
| IbRR29 | g49840 | Rec, Myb | Chr12 | 21899420-21908026 (+) | 2793 | 931 | 104.6 | 9.51 | Cytoplasm |
| IbRR30 | g25313 | Rec, Myb | Chr07 | 310054-317169 (-) | 2709 | 903 | 100.0 | 8.45 | Cytoplasm |
| IbRR31 | g20553 | Rec | Chr05 | 28733640-28735920 (-)- | 525 | 175 | 20.0 | 8.33 | Cytoplasm |
| IbRR32 | g20548 | Rec | Chr05 | 28668709-28671582 (+) | 1614 | 538 | 57.8 | 5.01 | Cytoplasm |
| **Type C RRs** | | | | | | | | | |
| IbRR33 | g33222 | Rec | Chr08 | 20372592-20373060 (+) | 363 | 121 | 13.7 | 9.10 | Cytoplasm |
| IbRR34 | g34928 | Rec | Chr09 | 6076930-6078562 (+) | 528 | 175 | 18.4 | 5.19 | Cytoplasm |
| IbRR35 | g34926 | Rec | Chr09 | 6068742-6072296 (-) | 795 | 265 | 29.0 | 9.54 | Cytoplasm |
| IbRR36 | g34929 | Rec | Chr09 | 6078940-6082015 (-) | 720 | 240 | 25.8 | 5.41 | Cytoplasm |
| IbRR37 | g33149 | Rec | Chr08 | 20286385-20287049 (+) | 321 | 107 | 11.8 | 7.82 | Nucleus |
| IbRR38 | g33158 | Rec | Chr08 | 20372592-20373065 (+) | 396 | 132 | 14.6 | 6.90 | Cytoplasm |
| IbRR39 | g33229 | Rec | Chr08 | 20901194-20906010 (+) | 978 | 326 | 36.4 | 8.63 | Cytoplasm |
| **Pseudo RRs** | | | | | | | | | |
| IbPRR1 | g53330 | Pseudo-Rec, CCT | Chr13 | 15906066-15910380 (-) | 1743 | 581 | 65.5 | 5.82 | Nucleus |
| IbPRR2 | g59988 | Pseudo-Rec | Chr15 | 713050-713660 (+) | 450 | 150 | 16.4 | 8.63 | Nucleus |
| IbPRR3 | g30584 | Pseudo-Rec, CCT | Chr08 | 1863739-1874809 (-) | 3570 | 1190 | 129.8 | 9.15 | Cytoplasm |
| IbPRR4 | g8789 | Pseudo-Rec | Chr02 | 33719520-33720500 (+) | 612 | 204 | 22.8 | 5.26 | Nucleus |
| IbPRR5 | g31109 | Pseudo-Rec, CCT | Chr08 | 4725905-4731778 (+) | 2418 | 806 | 88.4 | 6.63 | Nucleus |
| IbPRR6 | g871 | Pseudo-Rec, CCT | Chr01 | 5087616-5090400 (-) | 1878 | 626 | 69.0 | 6.61 | Nucleus |
| IbPRR7 | g20699 | Pseudo-Rec, CCT | Chr05 | 29589949-29592720 (+) | 1656 | 552 | 62.1 | 8.85 | Nucleus |
| IbPRR8 | g27243 | Pseudo-Rec, CCT | Chr07 | 14879775-14884127 (+) | 1308 | 436 | 48.5 | 6.00 | Nucleus |
| IbPRR9 | g27036 | Pseudo-Rec, CCT | Chr07 | 14879780-14884126 (+) | 1308 | 436 | 47.9 | 5.69 | Nucleus |
| IbPRR10 | g38332 | Pseudo-Rec, Myb | Chr10 | 1158944-1167350 (+) | 5226 | 1742 | 194.7 | 6.82 | Cytoplasm |
| IbPRR11 | g15212 | Pseudo-Rec, Myb | Chr04 | 18158893-18163850 (+) | 1737 | 579 | 64.6 | 6.61 | Nucleus |
| IbPRR12 | g20841 | Pseudo-Rec, Myb | Chr05 | 30389900-30393020 (+) | 1476 | 492 | 55.2 | 5.79 | Cytoplasm |
| IbPRR13 | g61530 | Pseudo-Rec, Myb | Chr15 | 11255736-11246946 (-) | 1659 | 553 | 60.1 | 7.22 | Cytoplasm |
| IbPRR14 | g61529 | Pseudo-Rec | Chr15 | 11245830-11247718 (-) | 486 | 161 | 17.9 | 4.77 | Cytoplasm |
| IbPRR15 | g56632 | Pseudo-Rec | Chr14 | 8920960-8921418 (-) | 306 | 102 | 11.4 | 5.84 | Cytoplasm |
| IbPRR16 | g56635 | Pseudo-Rec, Myb | Chr14 | 8941180-8942172 (+) | 669 | 223 | 25.4 | 7.08 | Cytoplasm |
| IbPRR17 | g56637 | Pseudo-Rec, Myb | Chr14 | 8950855-8951897 (+) | 684 | 228 | 25.9 | 6.46 | Nucleus |
| IbPRR18 | g6428 | Pseudo-Rec | Chr02 | 15719110-15488004 (+) | 522 | 174 | 19.6 | 8.63 | Cytoplasm |
| IbPRR19 | g35948 | Pseudo-Rec | Chr09 | 13854054-13854580 (-) | 384 | 128 | 14.3 | 5.85 | Cytoplasm |
| IbPRR20 | g41064 | Pseudo-Rec | Chr10 | 21569283-21569815 (-) | 432 | 144 | 15.9 | 10.7 | Cytoplasm |
