# Supplementary material for: Genome-wide identification and expression analysis of two-component system genes in sweet potato (Ipomoea batatas L.)
Source: Front Plant Sci. 2023 Jan 12;13:1091620. doi: 10.3389/fpls.2022.1091620 (PMC9878860; doi:10.3389/fpls.2022.1091620)
Supplement: Supplementary file 1 [file DataSheet_1.zip › Supplementary Table S4. TCS proteins in Ipomoea trifida.docx]

Table S4. TCS proteins in *Ipomoea trifida* genome.

| **Gene name** | **Gene locus** | **Domains** | **Chr** | **position** | **ORF length (bp)** | **Deduced polypeptide** | | | **Subcellular**  **localization** |
| --- | --- | --- | --- | --- | --- | --- | --- | --- | --- |
|  |  |  |  |  |  | Length  (aa) | MW  (kDa) | PI |  |
| **HKs** | | | | | | | | | |
| ItfHK1a | itf05g23770 | HK, Rec | Chr05 | 23828349-23822272 | 3618 | 1205 | 134.9 | 6.15 | Plasma membrane |
| ItfHK1b | itf03g06760 | HK, Rec | Chr03 | 4324046-4318372 | 3585 | 1194 | 133.2 | 6.58 | Plasma membrane |
| ItfHK2a | itf02g10570 | CHASE, HK, Rec | Chr02 | 9201334-9192362 | 3756 | 1251 | 140.7 | 6.90 | Endoplasmic Reticulum and Membrane |
| ItfHK2b | itf09g07800 | CHASE, HK, Rec | Chr09 | 4184084-4191169 | 3591 | 1196 | 134.4 | 6.57 | Endoplasmic Reticulum and Membrane |
| ItfHK3 | itf08g10860 | CHASE, HK, Rec | Chr08 | 9443184-9436844 | 3093 | 1030 | 115.2 | 6.56 | Endoplasmic Reticulum and Membrane |
| ItfHK4 | itf07g06180 | CHASE, HK, Rec | Chr07 | 4150452-4155275 | 2415 | 804 | 88.0 | 6.15 | Endoplasmic Reticulum and Membrane |
| ItfHK5 | itf07g05390 | HK, Rec | Chr07 | 3545113-3553224 | 3066 | 1021 | 114.7 | 5.25 | Cytoplasm and Membrane |
| ItfCKI1 | itf14g00150 | HK, Rec | Chr14 | 116620-119414 | 2292 | 763 | 83.9 | 6.20 | Endoplasmic Reticulum |
| ItfETR1a | itf01g21090 | GAF, HK, Rec | Chr01 | 22113639-22107469 | 2223 | 740 | 82.7 | 7.13 | Endoplasmic Reticulum |
| ItfETR1b | itf01g21080 | GAF, HK, Rec | Chr01 | 22098695-22097260 | 483 | 160 | 17.0 | 9.16 | Endoplasmic Reticulum |
| ItfERS1 | itf04g24110 | GAF, HK | Chr04 | 25654534-25658346 | 1908 | 635 | 70.9 | 6.75 | Endoplasmic Reticulum |
| ItfHKL1 | itf03g16670 | GAF, HKL, Rec | Chr03 | 13387113-13392642 | 2292 | 763 | 85.0 | 6.93 | Endoplasmic Reticulum |
| ItfHKL2 | itf05g05210 | GAF, HKL, Rec | Chr05 | 4873049-4869665 | 2286 | 761 | 85.0 | 8.61 | Endoplasmic Reticulum |
| ItfHKL3 | itf04g07670 | GAF, HKL, Rec | Chr04 | 4762145-4765768 | 2280 | 759 | 84.3 | 8.45 | Endoplasmic Reticulum |
| ItfHKL4 | itf13g20890 | GAF, HKL, Rec | Chr13 | 21554794-21558998 | 2337 | 778 | 87.0 | 8.12 | Endoplasmic Reticulum |
| ItfHKL5 | itf13g15110 | GAF, HKL, Rec | Chr13 | 17463107-17459455 | 2346 | 781 | 86.9 | 8.13 | Endoplasmic Reticulum |
| ItfHKL6 | itf15g14890 | GAF, PHY, HKL | Chr15 | 12255116-12247829 | 3384 | 1127 | 124.9 | 5.89 | Cytoplasm and Nucleus |
| ItfHKL7 | itf09g07850 | GAF, PHY, HKL | Chr09 | 4221474-4214893 | 3366 | 1121 | 124.5 | 5.78 | Cytoplasm and Nucleus |
| ItfHKL8 | itf01g35930 | GAF, PHY, HKL | Chr01 | 32008533-32013639 | 3390 | 1129 | 125.4 | 5.65 | Cytoplasm and Nucleus |
| ItfHKL9 | itf13g18050 | GAF, PHY, HKL | Chr13 | 19903145-19897353 | 3408 | 1135 | 125.8 | 6.48 | Cytoplasm and Nucleus |
| ItfHKL10 | itf10g03700 | GAF, PHY, HKL | Chr10 | 3192299-3186211 | 3393 | 1130 | 125.9 | 5.81 | Cytoplasm and Nucleus |
| **HPs** | | | | | | | | | |
| ItfHP1 | itf13g19130 | HPt | Chr13 | 20552583-20550378 | 456 | 151 | 17.2 | 4.83 | Cytoplasm and Nucleus |
| ItfHP2 | itf14g19090 | HPt | Chr14 | 18474353-18477332 | 459 | 152 | 17.3 | 4.97 | Cytoplasm and Nucleus |
| ItfHP3 | itf04g24780 | HPt | Chr04 | 26150668-26148411 | 459 | 152 | 17.3 | 5.78 | Cytoplasm and Nucleus |
| ItfHP4 | itf15g21110 | HPt | Chr15 | 20211709-20208066 | 411 | 136 | 15.7 | 5.19 | Cytoplasm and Nucleus |
| ItfHP5 | itf06g17660 | HPt | Chr06 | 20310218-20312004 | 453 | 150 | 17.5 | 8.27 | Cytoplasm and Nucleus |
| ItfHP6 | itf12g21020 | Pseudo-HPt | Chr12 | 20125785-20127271 | 414 | 137 | 15.5 | 5.59 | Cytoplasm and Nucleus |
| **Type A RRs** | | | | | | | | | |
| ItfRR1 | itf02g19770 | Rec | Chr02 | 15763455-15761436 | 612 | 203 | 22.0 | 5.23 | Nucleus |
| ItfRR2 | itf01g24600 | Rec | Chr01 | 24701147-24698996 | 813 | 270 | 52.7 | 5.44 | Nucleus |
| ItfRR3 | itf11g21120 | Rec | Chr11 | 18388795-18391251 | 669 | 222 | 24.0 | 6.53 | Nucleus |
| ItfRR4 | itf07g22870 | Rec | Chr07 | 23029948-23026803 | 807 | 268 | 28.8 | 6.68 | Nucleus |
| ItfRR5 | itf14g04090 | Rec | Chr14 | 3418479-3416250 | 693 | 230 | 25.0 | 5.51 | Nucleus |
| ItfRR6 | itf11g02520 | Rec | Chr11 | 1267541-1266462 | 549 | 182 | 20.00 | 4.81 | Nucleus |
| ItfRR7 | itf11g02530 | Rec | Chr11 | 1279103-1277634 | 705 | 234 | 26.2 | 9.32 | Nucleus |
| ItfRR8 | itf09g11500 | Rec | Chr09 | 6622259-6620426 | 645 | 214 | 23.8 | 5.52 | Nucleus |
| ItfRR9 | itf15g04350 | Rec | Chr15 | 2552046-2549267 | 711 | 236 | 26.3 | 5.13 | Nucleus |
| ItfRR10 | itf04g27950 | Rec | Chr04 | 28103783-28102235 | 492 | 163 | 44.1 | 5.93 | Nucleus |
| ItfRR11 | itf05g02210 | Rec | Chr05 | 1687098-1685474 | 417 | 138 | 15.0 | 4.88 | Nucleus |
| ItfRR12 | itf12g24050 | Rec | Chr12 | 21985655-21986736 | 363 | 120 | 13.3 | 5.59 | Nucleus |
| ItfRR13 | itf05g02200 | Rec | Chr05 | 1674990-1673579 | 375 | 124 | 13.4 | 4.28 | Nucleus |
| **Type B RRs** | | | | | | | | | |
| ItfRR14 | itf07g19070 | Rec, Myb | Chr07 | 18942149-18944832 | 1287 | 428 | 47.6 | 8.88 | Nucleus |
| ItfRR15 | itf07g04050 | Rec, Myb | Chr07 | 2637648-2633681 | 1650 | 549 | 61.4 | 5.70 | Nucleus |
| ItfRR16 | itf13g18290 | Rec, Myb | Chr13 | 20071511-20066918 | 2091 | 696 | 75.7 | 6.07 | Nucleus |
| ItfRR17 | itf04g16470 | Rec, Myb | Chr04 | 15187238-15191631 | 1989 | 662 | 72.7 | 5.66 | Nucleus |
| ItfRR18 | itf07g06370 | Rec, Myb | Chr07 | 4290454-4284965 | 1974 | 657 | 71.7 | 6.20 | Nucleus |
| ItfRR19 | itf15g17550 | Rec, Myb | Chr15 | 16371060-16375199 | 1755 | 584 | 64.7 | 5.92 | Nucleus |
| ItfRR20 | itf03g22130 | Rec, Myb | Chr03 | 17570552-17574207 | 1992 | 663 | 72.6 | 5.55 | Cytoplasm |
| ItfRR21 | itf07g23710 | Rec, Myb | Chr07 | 23687116-23683865 | 1971 | 656 | 71.9 | 5.91 | Nucleus |
| ItfRR22 | itf13g00740 | Rec, Myb | Chr13 | 518203-515043 | 1917 | 638 | 69.2 | 6.00 | Nucleus |
| ItfRR23 | itf12g25040 | Rec, Myb | Chr12 | 22603298-22606170 | 1386 | 461 | 49.4 | 4.88 | Cytoplasm |
| **Type C RRs** | | | | | | | | | |
| ItfRR24 | itf11g17110 | Rec | Chr11 | 14020872-14021575 | 360 | 119 | 13.4 | 6.74 | Nucleus |
| ItfRR25 | itf00g12530 | Rec | Chr00 | 21333708-21334410 | 360 | 119 | 13.3 | 6.11 | Nucleus |
| ItfRR26 | itf10g17630 | Rec | Chr10 | 19706294-19708439 | 642 | 213 | 23.3 | 5.55 | Nucleus |
| ItfRR27 | itf10g17580 | Rec | Chr10 | 19694032-19694783 | 390 | 129 | 14.0 | 6.41 | Nucleus |
| ItfRR28 | itf11g17570 | Rec | Chr11 | 14763326-14761179 | 414 | 137 | 15.0 | 6.82 | Nucleus |
| ItfRR29 | itf11g17560 | Rec | Chr11 | 14743561-14742244 | 375 | 124 | 13.5 | 4.96 | Nucleus |
| **Pseudo RRs** | | | | | | | | | |
| ItfPRR1 | itf00g10880 | Pseudo-Rec | Chr00 | 18370246-18362641 | 1134 | 377 | 41.8 | 6.27 | Nucleus |
| ItfPRR2 | itf02g03390 | Pseudo-Rec, CCT | Chr02 | 4390672-4395829 | 1647 | 548 | 61.8 | 5.74 | Nucleus |
| ItfPRR3 | itf06g26040 | Pseudo-Rec | Chr06 | 25546685-25545597 | 450 | 149 | 16.4 | 8.63 | Nucleus |
| ItfPRR4 | itf04g05990 | Pseudo-Rec | Chr04 | 3516896-3515922 | 612 | 203 | 22.7 | 5.16 | Nucleus |
| ItfPRR5 | itf11g02700 | Pseudo-Rec, CCT | Chr11 | 1392708-1386190 | 2178 | 725 | 79.3 | 7.07 | Nucleus |
| ItfPRR6 | itf11g06930 | Pseudo-Rec, CCT | Chr11 | 3711243-3718588 | 2385 | 794 | 87.2 | 6.67 | Nucleus |
| ItfPRR7 | itf12g26610 | Pseudo-Rec, CCT | Chr12 | 23500913-23503505 | 1650 | 549 | 61.3 | 5.59 | Nucleus |
| ItfPRR8 | itf05g20000 | Pseudo-Rec, CCT | Chr05 | 21543486-21546985 | 1974 | 657 | 72.4 | 6.67 | Nucleus |
| ItfPRR9 | itf03g26830 | Pseudo-Rec, CCT | Chr03 | 23076934-23071096 | 1425 | 474 | 52.6 | 5.71 | Nucleus |
| ItfPRR10 | itf06g13800 | Pseudo-Rec, Myb | Chr06 | 16945977-16955278 | 2862 | 953 | 102.9 | 8.93 | Cytoplasm |
| ItfPRR11 | itf12g27250 | Pseudo-Rec, Myb | Chr12 | 23787426-23784236 | 1521 | 506 | 56.9 | 5.77 | Cytoplasm |
| ItfPRR12 | itf08g01660 | Pseudo-Rec, Myb | Chr08 | 1137968-1143704 | 1668 | 555 | 61.7 | 6.27 | Nucleus |
| ItfPRR13 | itf13g06280 | Pseudo-Rec, Myb | Chr13 | 7025447-7019715 | 1668 | 555 | 62.2 | 6.24 | Nucleus |
| ItfPRR14 | itf09g21410 | Pseudo-Rec, Myb | Chr09 | 18197569-18193041 | 1452 | 483 | 54.2 | 5.99 | Cytoplasm |
| ItfPRR15 | itf09g21430 | Pseudo-Rec, Myb | Chr09 | 18207793-18206793 | 669 | 222 | 25.3 | 6.46 | Cytoplasm |
