# Supplementary material for: Genome-wide identification and expression analysis of two-component system genes in sweet potato (Ipomoea batatas L.)
Source: Front Plant Sci. 2023 Jan 12;13:1091620. doi: 10.3389/fpls.2022.1091620 (PMC9878860; doi:10.3389/fpls.2022.1091620)
Supplement: Supplementary file 1 [file DataSheet_1.zip › Supplementary Table S5. TCS proteins in Ipomoea triloba.docx]

Table S5. TCS proteins in *Ipomoea triloba* genome.

| **Gene name** | **Gene locus** | **Domains** | **Chr** | **position** | **ORF length (bp)** | **Deduced polypeptide** | | | **Subcellular**  **localization** |
| --- | --- | --- | --- | --- | --- | --- | --- | --- | --- |
|  |  |  |  |  |  | Length  (aa) | MW  (kDa) | PI |  |
| **HKs** | | | | | | | | | |
| ItbHK1a | itb05g24510 | HK, Rec | Chr05 | 29279114-29272694 | 3618 | 1205 | 135.0 | 6.32 | Plasma membrane |
| ItbHK1b | itb03g06800 | HK, Rec | Chr03 | 4922980-4916792 | 3621 | 1206 | 134.7 | 6.49 | Plasma membrane |
| ItbHK2a | itb02g05980 | CHASE, HK, Rec | Chr02 | 3723386-3715010 | 3756 | 1251 | 140.6 | 6.68 | Endoplasmic Reticulum and Membrane |
| ItbHK2b | itb09g08440 | CHASE, HK, Rec | Chr09 | 5050366-5057038 | 3591 | 1196 | 134.3 | 6.44 | Endoplasmic Reticulum and Membrane |
| ItbHK3 | itb08g12200 | CHASE, HK, Rec | Chr08 | 12297049-12303694 | 3093 | 1030 | 115.3 | 6.56 | Endoplasmic Reticulum and Membrane |
| ItbHK4 | itb07g06230 | CHASE, HK, Rec | Chr07 | 4389710-4394835 | 2985 | 994 | 110.0 | 6.67 | Endoplasmic Reticulum and Membrane |
| ItbHK5 | itb07g05460 | HK, Rec | Chr07 | 3761209-3769429 | 3066 | 1021 | 114.7 | 5.29 | Cytoplasm and Membrane |
| ItbCKI1 | itb14g00190 | HK, Rec | Chr14 | 134224-137058 | 2193 | 730 | 80.8 | 6.22 | Plasma membrane |
| ItbETR1a | itb01g20480 | GAF, HK, Rec | Chr01 | 26736835-26730435 | 2223 | 740 | 82.8 | 7.38 | Endoplasmic reticulum |
| ItbETR1b | itb01g20470 | GAF, HK, Rec | Chr01 | 26722212-26717005 | 1122 | 373 | 42.2 | 9.95 | Endoplasmic reticulum |
| ItbERS1 | itb04g23530 | GAF, HK | Chr04 | 28556225-28560205 | 1908 | 635 | 70.9 | 6.75 | Endoplasmic reticulum |
| ItbHKL1 | itb03g17470 | GAF, HKL, Rec | Chr03 | 16085198-16090655 | 2292 | 763 | 85.1 | 7.20 | Endoplasmic reticulum |
| ItbHKL2 | itb05g04560 | GAF, HKL, Rec | Chr05 | 4164061-4159351 | 2286 | 761 | 85.0 | 8.61 | Endoplasmic reticulum |
| ItbHKL3 | itb04g07980 | GAF, HKL, Rec | Chr04 | 5553486-5557525 | 2280 | 759 | 84.3 | 8.26 | Endoplasmic reticulum |
| ItbHKL4 | itb13g18370 | GAF, HKL, Rec | Chr13 | 25337689-25333105 | 2349 | 782 | 174.7 | 6.97 | Cytoplasm |
| ItbHKL5 | itb13g24440 | GAF, HKL, Rec | Chr13 | 30110357-30114656 | 2337 | 778 | 87.0 | 8.12 | Endoplasmic reticulum |
| ItbHKL6 | itb15g15470 | GAF, PHY, HKL | Chr15 | 14404572-14399616 | 3384 | 1127 | 124.9 | 5.89 | Cytoplasm and Nucleus |
| ItbHKL7 | itb09g08530 | GAF, PHY, HKL | Chr09 | 5088084-5081983 | 3366 | 1121 | 124.5 | 5.74 | Cytoplasm and Nucleus |
| ItbHKL8 | itb10g04130 | GAF, PHY, HKL | Chr10 | 3899141-3893445 | 3396 | 1131 | 126.1 | 5.78 | Cytoplasm and Nucleus |
| ItbHKL9 | itb01g35800 | GAF, PHY, HKL | Chr01 | 37804879-37809975 | 3390 | 1129 | 125.4 | 5.65 | Cytoplasm and Nucleus |
| ItbHKL10 | itb13g21550 | GAF, PHY, HKL | Chr13 | 28140282-28134954 | 3408 | 1135 | 125.7 | 6.42 | Cytoplasm and Nucleus |
| **HPs** | | | | | | | | | |
| ItbHP1 | itb13g22750 | HPt | Chr13 | 28990604-28988927 | 438 | 145 | 16.8 | 5.15 | Cytoplasm and Nucleus |
| ItbHP2 | itb14g20770 | HPt | Chr14 | 23025699-23028658 | 459 | 152 | 17.2 | 4.85 | Cytoplasm and Nucleus |
| ItbHP3 | itb04g24370 | HPt | Chr04 | 29084464-29082087 | 459 | 152 | 17.3 | 6.07 | Cytoplasm and Nucleus |
| ItbHP4 | itb15g21560 | HPt | Chr15 | 24267059-24263607 | 411 | 136 | 15.7 | 5.04 | Cytoplasm and Nucleus |
| ItbHP5 | itb06g16060 | HPt | Chr06 | 20272788-20274515 | 453 | 150 | 17.5 | 8.27 | Cytoplasm and Nucleus |
| ItbHP6 | itb12g20130 | Pseudo-HPt | Chr12 | 22619134-22621134 | 435 | 144 | 16.8 | 5.22 | Cytoplasm and Nucleus |
| **Type A RRs** | | | | | | | | | |
| ItbRR1 | itb07g23570 | Rec | Chr07 | 27947535-27944519 | 807 | 268 | 28.8 | 4.98 | Nucleus |
| ItbRR2 | itb14g04320 | Rec | Chr14 | 3892084-3889733 | 690 | 229 | 25.0 | 5.15 | Nucleus |
| ItbRR3 | itb11g22970 | Rec | Chr11 | 24863394-24865792 | 672 | 223 | 24.2 | 6.53 | Nucleus |
| ItbRR4 | itb02g12560 | Rec | Chr02 | 8659111-8661280 | 582 | 193 | 21.0 | 5.38 | Nucleus |
| ItbRR5 | itb12g24390 | Rec | Chr12 | 25934623-25936076 | 438 | 145 | 16.2 | 5.93 | Nucleus |
| ItbRR6 | itb15g04580 | Rec | Chr15 | 2902768-2900089 | 723 | 240 | 26.7 | 5.21 | Nucleus |
| ItbRR7 | itb09g12560 | Rec | Chr09 | 7940859-7938623 | 645 | 214 | 23.8 | 5.36 | Nucleus |
| ItbRR8 | itb01g24350 | Rec | Chr01 | 30004185-30002021 | 807 | 268 | 30.5 | 5.46 | Nucleus |
| ItbRR9 | itb05g01870 | Rec | Chr05 | 1463380-1461048 | 453 | 150 | 16.4 | 4.76 | Nucleus |
| ItbRR10 | itb05g01890 | Rec | Chr05 | 1473215-1471511 | 417 | 138 | 15.1 | 6.57 | Nucleus |
| ItbRR11 | itb05g01860 | Rec | Chr05 | 1452971-1451299 | 381 | 126 | 13.6 | 4.74 | Nucleus |
| ItbRR12 | itb11g02460 | Rec | Chr11 | 1232158-1231104 | 558 | 185 | 20.2 | 4.75 | Nucleus |
| ItbRR13 | itb11g02470 | Rec | Chr11 | 1236102-1235379 | 279 | 92 | 10.5 | 10.3 | Nucleus |
| **Type B RRs** | | | | | | | | | |
| ItbRR14 | itb07g21330 | Rec, Myb | Chr07 | 25726280-25727972 | 984 | 327 | 36.8 | 9.24 | Nucleus |
| ItbRR15 | itb07g04180 | Rec, Myb | Chr07 | 2800151-2796443 | 1650 | 549 | 61.4 | 6.01 | Nucleus |
| ItbRR16 | itb07g06400 | Rec, Myb | Chr07 | 4557624-4551988 | 1995 | 664 | 72.5 | 6.39 | Nucleus |
| ItbRR17 | itb13g21840 | Rec, Myb | Chr13 | 28350954-28346141 | 2094 | 697 | 75.9 | 6.03 | Nucleus |
| ItbRR18 | itb04g20180 | Rec, Myb | Chr04 | 24716019-24720557 | 1989 | 662 | 72.7 | 5.64 | Nucleus |
| ItbRR19 | itb15g18490 | Rec, Myb | Chr15 | 20161124-20166262 | 1833 | 610 | 67.8 | 5.77 | Nucleus |
| ItbRR20 | itb03g22710 | Rec, Myb | Chr03 | 20781137-20784730 | 1977 | 658 | 71.9 | 5.76 | Cytoplasm |
| ItbRR21 | itb07g24410 | Rec, Myb | Chr07 | 28705009-28701598 | 1971 | 656 | 71.9 | 5.88 | Nucleus |
| ItbRR22 | itb13g00930 | Rec, Myb | Chr13 | 829120-824969 | 2127 | 708 | 77.0 | 6.19 | Nucleus |
| **Type C RRs** | | | | | | | | | |
| ItbRR23 | itb12g25460 | Rec | Chr12 | 26712386-26707242 | 960 | 319 | 36.8 | 6.56 | Cytoplasm |
| ItbRR24 | itb12g25380 | Rec | Chr12 | 26612961-26615315 | 1437 | 478 | 51.8 | 5.47 | Cytoplasm |
| ItbRR25 | itb10g17820 | Rec | Chr10 | 23929402-23930039 | 390 | 129 | 14.1 | 4.81 | Nucleus |
| ItbRR26 | itb10g17780 | Rec | Chr10 | 23907812-23908461 | 294 | 97 | 10.8 | 4.93 | Nucleus |
| ItbRR27 | itb10g17770 | Rec | Chr10 | 23903579-23907073 | 654 | 217 | 23.7 | 5.28 | Cytoplasm |
| ItbRR28 | itb11g20500 | Rec | Chr11 | 21626136-21626886 | 339 | 112 | 12.3 | 8.79 | Nucleus |
| **Pseudo RRs** | | | | | | | | | |
| ItbPRR1 | itb02g16680 | Pseudo-Rec, CCT | Chr02 | 12645458-12650915 | 1647 | 548 | 61.8 | 5.74 | Nucleus |
| ItbPRR2 | itb06g24850 | Pseudo-Rec | Chr06 | 26247521-26246548 | 450 | 149 | 16.3 | 8.63 | Nucleus |
| ItbPRR3 | itb04g06300 | Pseudo-Rec | Chr04 | 4084532-4083559 | 591 | 196 | 21.8 | 5.04 | Nucleus |
| ItbPRR4 | itb11g02680 | Pseudo-Rec, CCT | Chr11 | 1371752-1365430 | 2178 | 725 | 79.4 | 6.91 | Nucleus |
| ItbPRR5 | itb11g07200 | Pseudo-Rec, CCT | Chr11 | 4395078-4401843 | 2379 | 792 | 87.0 | 6.76 | Nucleus |
| ItbPRR6 | itb03g24570 | Pseudo-Rec, CCT | Chr03 | 23294358-23299429 | 1305 | 434 | 48.2 | 5.84 | Nucleus |
| ItbPRR7 | itb05g20630 | Pseudo-Rec, CCT | Chr05 | 26659826-26663430 | 1974 | 657 | 72.5 | 6.57 | Nucleus |
| ItbPRR8 | itb12g27020 | Pseudo-Rec, CCT | Chr12 | 27638959-27641684 | 1578 | 525 | 58.5 | 6.24 | Nucleus |
| ItbPRR9 | itb06g11820 | Pseudo-Rec, Myb | Chr06 | 16372730-16377656 | 1575 | 524 | 57.0 | 7.20 | Cytoplasm |
| ItbPRR10 | itb12g27680 | Pseudo-Rec, Myb | Chr12 | 27938139-27934967 | 1602 | 533 | 59.6 | 5.55 | Cytoplasm |
| ItbPRR11 | itb08g01680 | Pseudo-Rec, Myb | Chr08 | 1357893-1363589 | 1671 | 556 | 61.8 | 6.12 | Nucleus |
| ItbPRR12 | itb13g12000 | Pseudo-Rec, Myb | Chr13 | 17809043-17815053 | 1668 | 555 | 62.1 | 6.51 | Nucleus |
| ItbPRR13 | itb09g23880 | Pseudo-Rec, Myb | Chr09 | 23494163-23500652 | 1608 | 535 | 59.2 | 4.91 | Cytoplasm |
