# Supplementary material for: Genome-wide identification and expression analysis of two-component system genes in sweet potato (Ipomoea batatas L.)
Source: Front Plant Sci. 2023 Jan 12;13:1091620. doi: 10.3389/fpls.2022.1091620 (PMC9878860; doi:10.3389/fpls.2022.1091620)
Supplement: Supplementary file 1 [file DataSheet_1.zip › Supplementary Table S6. TCS proteins in Ipomoea nil.docx]

Table S6. TCS proteins in *Ipomoea nil* genome.

| **Gene name** | **Gene locus** | **Domains** | **Deduced polypeptide** | | | **Subcellular localization** |
| --- | --- | --- | --- | --- | --- | --- |
|  |  |  | Length  (aa) | MW  (kDa) | PI |  |
| **HKs** | | | | | | |
| InHK1a | XP_019177443 | HK, Rec | 1208 | 135.4 | 6.64 | Plasma membrane |
| InHK1b | XP_019172390 | HK, Rec | 1208 | 135.0 | 6.40 | Plasma membrane |
| InHK2a | XP_019193391 | CHASE, HK, Rec | 1218 | 136.5 | 6.88 | Endoplasmic Reticulum and Membrane |
| InHK2b | XP_019162776 | CHASE, HK, Rec | 1223 | 137.7 | 7.36 | Endoplasmic Reticulum and Membrane |
| InHK3 | XP_019155744 | CHASE, HK, Rec | 1031 | 115.4 | 6.51 | Endoplasmic Reticulum and Membrane |
| InHK4 | XP_019176525 | CHASE, HK, Rec | 990 | 110.0 | 6.52 | Endoplasmic Reticulum and Membrane |
| InHK5 | XP_019176804 | HK, Rec | 1021 | 114.7 | 5.23 | Cytoplasm and Membrane |
| InCKI1a | XP_019189130 | HK, Rec | 366 | 40.5 | 6.36 | Endoplasmic Reticulum |
| InCKI1b | XP_019171259 | HK, Rec | 693 | 76.6 | 5.80 | Endoplasmic Reticulum |
| InETR1 | XP_019154004 | GAF, HK, Rec | 740 | 82.8 | 7.15 | Endoplasmic Reticulum |
| InERS1 | XP_019163846 | GAF, HK | 635 | 71.0 | 6.75 | Endoplasmic Reticulum |
| InHKL1 | XP_019192407 | GAF, HKL, Rec | 762 | 85.0 | 7.87 | Endoplasmic Reticulum |
| InHKL2 | XP_019192791 | GAF, HKL, Rec | 688 | 77.0 | 9.13 | Endoplasmic Reticulum |
| InHKL3 | XP_019191425 | GAF, HKL, Rec | 756 | 83.8 | 8.00 | Endoplasmic Reticulum |
| InHKL4 | XP_019181054 | GAF, HKL, Rec | 780 | 86.4 | 7.95 | Endoplasmic Reticulum |
| InHKL5 | XP_019180505 | GAF, HKL, Rec | 765 | 85.5 | 7.99 | Endoplasmic Reticulum |
| InHKL6 | XP_019191093 | PHY, HKL | 1127 | 125.2 | 6.06 | Cytoplasm and Nucleus |
| InHKL7 | XP_019162785 | PHY, HKL | 1121 | 124.4 | 5.73 | Cytoplasm and Nucleus |
| InHKL8 | XP_019178187 | PHY, HKL | 11131 | 126.2 | 5.91 | Cytoplasm and Nucleus |
| InHKL9 | XP_019174791 | PHY, HKL | 1131 | 125.6 | 5.71 | Cytoplasm and Nucleus |
| InHKL10 | XP_019180751 | PHY, HKL | 1134 | 125.9 | 6.62 | Cytoplasm and Nucleus |
| **HPs** | | | | | | |
| InHP1 | XP_019181218 | HPt | 151 | 17.19 | 4.83 | Cytoplasm and Nucleus |
| InHP2a | XP_019197548 | HPt | 152 | 17.19 | 5.31 | Cytoplasm and Nucleus |
| InHP2b | XP_019197494 | HPt | 152 | 17.28 | 5.33 | Cytoplasm and Nucleus |
| InHP3 | XP_019164476 | HPt | 152 | 17.23 | 5.78 | Cytoplasm and Nucleus |
| InHP4a | XP_019184827 | HPt | 136 | 15.66 | 5.18 | Cytoplasm and Nucleus |
| InHP4b | XP_019184817 | HPt | 136 | 15.73 | 5.42 | Cytoplasm and Nucleus |
| InHP5 | XP_019196386 | HPt | 150 | 17.54 | 8.59 | Cytoplasm and Nucleus |
| InHP6 | XP_019189371 | Pseudo-HPt | 160 | 18.47 | 7.01 | Cytoplasm and Nucleus |
| InHP7 | XP_019151109 | Pseudo-HPt | 146 | 16.39 | 5.02 | Cytoplasm and Nucleus |
| InHP8 | XP_019194240 | Pseudo-HPt | 146 | 16.75 | 5.22 | Cytoplasm and Nucleus |
| **Type A RRs** | | | | | | |
| InRR1 | XP_019162256 | Rec | 219 | 24.3 | 5.37 | Nucleus |
| InRR2 | XP_019186161 | Rec | 239 | 26.6 | 5.22 | Nucleus |
| InRR3 | XP_019166242 | Rec | 262 | 29.6 | 5.51 | Nucleus |
| InRR4 | XP_019163017 | Rec | 96 | 10.8 | 9.71 | Nucleus |
| InRR5 | XP_019200349 | Rec | 150 | 16.8 | 5.64 | Nucleus |
| InRR6 | XP_019157245 | Rec | 103 | 11.9 | 8.50 | Nucleus |
| InRR7 | XP_019184350 | Rec | 147 | 16.2 | 5.11 | Nucleus |
| InRR8 | XP_019184347 | Rec | 156 | 17.1 | 5.60 | Nucleus |
| InRR9 | XP_019197301 | Rec | 198 | 21.4 | 5.11 | Nucleus |
| InRR10 | XP_019154212 | Rec | 227 | 24.6 | 7.01 | Nucleus |
| InRR11 | XP_019170920 | Rec | 299 | 32.7 | 4.79 | Nucleus |
| InRR12 | XP_019164573 | Rec | 223 | 24.4 | 5.15 | Nucleus |
| InRR13 | XP_019157385 | Rec | 95 | 10.6 | 5.86 | Nucleus |
| InRR14 | XP_019184797 | Rec | 212 | 24.5 | 9.51 | Nucleus |
| InRR15 | XP_019183655 | Rec | 138 | 15.5 | 9.47 | Nucleus |
| InRR16 | XP_019159132 | Rec | 191 | 20.8 | 4.81 | Nucleus |
| InRR17 | XP_019185509 | Rec | 151 | 16.3 | 5.05 | Nucleus |
| **Type B RRs** | | | | | |  |
| InRR18 | XP_019180835 | Rec, Myb | 680 | 74.0 | 6.00 | Nucleus |
| InRR19 | XP_019177684 | Rec, Myb | 650 | 71.5 | 5.74 | Nucleus |
| InRR20 | XP_019176516 | Rec, Myb | 624 | 68.3 | 5.91 | Nucleus |
| InRR21 | XP_019176687 | Rec, Myb | 549 | 61.3 | 5.45 | Nucleus |
| InRR22 | XP_019173759 | Rec, Myb | 576 | 64.4 | 5.56 | Nucleus |
| InRR23 | XP_019197965 | Rec, Myb | 640 | 70.4 | 5.51 | Nucleus |
| InRR24 | XP_019181977 | Rec, Myb | 684 | 74.4 | 6.04 | Nucleus |
| InRR25 | XP_019170922 | Rec, Myb | 608 | 67.0 | 5.84 | Nucleus |
| InRR26 | XP_019188286 | Rec | 375 | 41.9 | 7.21 | Nucleus |
| InRR27 | XP_019191752 | Rec, Myb | 630 | 68.4 | 5.53 | Cytoplasm |
| **Type C RRs** | | | | | |  |
| InRR28 | XP_019169115 | Rec | 147 | 16.0 | 6.08 | Nucleus |
| InRR29 | XP_019178923 | Rec | 127 | 13.6 | 6.83 | Cytoplasm |
| **PRRs** | | | | | | |
| InPRR1 | XP_019199944 | Pseudo-Rec, CCT | 543 | 61.0 | 6.12 | Nucleus |
| InPRR2 | XP_019152769 | Pseudo-Rec | 153 | 16.8 | 8.72 | Cytoplasm |
| InPRR3 | XP_019159397 | Pseudo-Rec, CCT | 720 | 79.1 | 8.24 | Nucleus |
| InPRR4 | XP_019159402 | Pseudo-Rec, CCT | 632 | 69.4 | 9.02 | Nucleus |
| InPRR5 | XP_019184959 | Pseudo-Rec | 227 | 25.3 | 5.27 | Nucleus |
| InPRR6 | XP_019198690 | Pseudo-Rec, CCT | 787 | 85.6 | 6.53 | Nucleus |
| InPRR7 | XP_019200416 | Pseudo-Rec, CCT | 498 | 55.6 | 6.23 | Nucleus |
| InPRR8 | XP_019200419 | Pseudo-Rec, CCT | 472 | 52.7 | 6.11 | Nucleus |
| InPRR9 | XP_019195113 | Pseudo-Rec, CCT | 642 | 71.5 | 6.67 | Nucleus |
| InPRR10 | XP_019179827 | Pseudo-Rec, CCT | 438 | 48.1 | 5.84 | Nucleus |
| InPRR11 | XP_019176858 | Pseudo-Rec | 540 | 59.0 | 6.91 | Cytoplasm |
| InPRR12 | XP_019160991 | Pseudo-Rec, Myb | 280 | 31.5 | 6.17 | Cytoplasm |
| InPRR13 | XP_019200236 | Pseudo-Rec | 362 | 41.2 | 5.74 | Nucleus |
| InPRR14 | XP_019190940 | Pseudo-Rec | 499 | 56.0 | 6.48 | Nucleus |
| InPRR15 | XP_019156488 | Pseudo-Rec | 557 | 62.3 | 5.97 | Nucleus |
