# Supplementary material for: Genome-wide identification and expression analysis of two-component system genes in sweet potato (Ipomoea batatas L.)
Source: Front Plant Sci. 2023 Jan 12;13:1091620. doi: 10.3389/fpls.2022.1091620 (PMC9878860; doi:10.3389/fpls.2022.1091620)
Supplement: Supplementary file 1 [file DataSheet_1.zip › Supplementary Table S7. Ka, Ks calculation and divergent time of the segmental duplicated TCS gene pairs in Ipomoea batatas.docx]

Table S7. Ka, Ks calculation and divergent time of the segmental duplicated TCS gene pairs in *Ipomoea batatas*.

| Number | Gene I | Gene II | *Ka* | *Ks* | *Ka/Ks* | Divergence time  (MYA) |
| --- | --- | --- | --- | --- | --- | --- |
| 1 | *IbHK1a* | *IbHK1b* | 0.102486 | 0.668741 | 0.153252 | 22.29 |
| 2 | *IbHK2a* | *IbHK2b* | 0.185574 | 0.69412 | 0.267351 | 23.14 |
| 3 | *IbHKL2* | *IbHKL3* | 0.006575 | 0.047114 | 0.139555 | 1.58 |
| 4 | *IbHKL3* | *IbHKL5* | 0.175628 | 0.972313 | 0.18063 | 32.41 |
| 5 | *IbHKL9* | *IbHKL10* | 0.092211 | 0.682011 | 0.135204 | 22.73 |
| 6 | *IbHP1* | *IbHP2* | 0.234149 | 1.981019 | 0.118196 | 66.03 |
| 7 | *IbHP2* | *IbHP3* | 0.083447 | 0.511877 | 0.163021 | 17.06 |
| 8 | *IbRR1* | *IbRR3* | 0.18719 | 0.899632 | 0.208074 | 29.99 |
| 9 | *IbRR7* | *IbRR6* | 0.239217 | 1.149942 | 0.208025 | 38.33 |
| 10 | *IbRR6* | *IbRR19* | 0.233165 | 1.381245 | 0.168808 | 46.04 |
| 11 | *IbRR10* | *IbRR11* | 0.250481 | 1.664841 | 0.150454 | 55.49 |
| 12 | *IbRR14* | *IbRR18* | 0.304704 | 1.011041 | 0.301377 | 33.70 |
| 13 | *IbRR17* | *IbRR18* | 0.007346 | 0.01278 | 0.57483 | 0.423 |
| 14 | *IbRR20* | *IbRR21* | 0.174484 | 0.76873 | 0.226976 | 25.62 |
| 15 | *IbRR30* | *IbRR32* | 0.622701 | 3.422052 | 0.181967 | 114.07 |
| 16 | *IbPRR1* | *IbPRR2* | 0.430754 | 2.779934 | 0.154951 | 92.66 |
| 17 | *IbPRR6* | *IbPRR7* | 0.242255 | 0.876562 | 0.27637 | 29.22 |
| 18 | *IbPRR6* | *IbPRR8* | 0.219283 | 0.9719 | 0.225623 | 32.40 |
| 19 | *IbPRR6* | *IbPRR9* | 0.23993 | 0.992284 | 0.241796 | 33.08 |
| 20 | *IbPRR7* | *IbPRR8* | 0.260831 | 0.848751 | 0.307311 | 28.29 |
