# Supplementary material for: Genome-wide identification and expression analysis of two-component system genes in sweet potato (Ipomoea batatas L.)
Source: Front Plant Sci. 2023 Jan 12;13:1091620. doi: 10.3389/fpls.2022.1091620 (PMC9878860; doi:10.3389/fpls.2022.1091620)
Supplement: Supplementary file 1 [file DataSheet_1.zip › Supplementary Table S8. Distribution of tandem duplicated TCS gene pairs in Ipomoea batatas.docx]

Table S8. Distribution of tandem duplicated TCS gene pairs in Ipomoea batatas.

| **Gene subfamily** | **Tandem duplications** |
| --- | --- |
| *HK(L)* | none |
| *HP* | none |
| Type-A *RR* | IbRR1/ IbRR2、IbRR7/ IbRR8/ IbRR9、IbRR11/ IbRR12、  IbRR13/ IbRR14 |
| Type-B *RR* | IbRR31/ IbRR32 |
| Type-C *RR* | IbRR34/ IbRR35/ IbRR36 |
| Pseudo *RR* | IbPRR8/ IbRR9、IbPRR13/ IbRR14、IbPRR15/ IbRR16/ IbRR17 |
