# Supplementary material for: Genome-wide identification and expression analysis of two-component system genes in sweet potato (Ipomoea batatas L.)
Source: Front Plant Sci. 2023 Jan 12;13:1091620. doi: 10.3389/fpls.2022.1091620 (PMC9878860; doi:10.3389/fpls.2022.1091620)
Supplement: Supplementary file 1 [file DataSheet_1.zip › Supplementary Table S9. Segmental duplicated TCS genes Ipomoea batatas and Ipomoea trifida.docx]

Table S9. Segmental duplicated TCS genes *Ipomoea batatas* and *Ipomoea trifida*.

| **Number** | **Gene I** | **Gene II** | ***Ka*** | ***Ks*** | ***Ka/Ks*** |
| --- | --- | --- | --- | --- | --- |
| 1 | *IbHK1a* | *ItfHK1a* | 0.002519 | 0.040763 | 0.061802 |
| 2 | *IbHK1a* | *ItfHK1b* | 0.10069 | 0.654034 | 0.153952 |
| 3 | *IbHK1b* | *ItfHK1b* | 0.001486 | 0.029044 | 0.051179 |
| 4 | *IbHK1b* | *ItfHK1a* | 0.103616 | 0.672066 | 0.154176 |
| 5 | *IbHK3* | *ItfHK3* | 0.012131 | 0.044014 | 0.275617 |
| 6 | *IbHK4* | *ItfHK4* | 0.016062 | 0.101935 | 0.157574 |
| 7 | *IbHK5* | *ItfHK5* | 0.001738 | 0.021515 | 0.080789 |
| 8 | *IbCKI1* | *ItfCKI1* | 0.005198 | 0.011658 | 0.445892 |
| 9 | *IbETR1* | *ItfETR1b* | 0.134573 | 0.17912 | 0.751301 |
| 10 | *IbERS1* | *ItfERS1* | 0.022596 | 0.060762 | 0.371885 |
| 11 | *IbHKL1* | *ItfHKL1* | 0.004142 | 0.051593 | 0.080286 |
| 12 | *IbHKL2* | *ItfHKL3* | 0.00516 | 0.067495 | 0.076448 |
| 13 | *IbHKL3* | *ItfHKL3* | 0.009584 | 0.072885 | 0.131498 |
| 14 | *IbHKL5* | *ItfHKL3* | 0.167349 | 0.912341 | 0.183428 |
| 15 | *IbHKL7* | *ItfHKL7* | 0.031988 | 0.052313 | 0.611485 |
| 16 | *IbHKL8* | *ItfHKL10* | 7.63E-04 | 0.036103 | 0.021122 |
| 17 | *IbHKL9* | *ItfHKL8* | 7.65E-04 | 0.031831 | 0.024022 |
| 18 | *IbHKL10* | *ItfHKL8* | 0.091354 | 0.701246 | 0.130273 |
| 19 | *IbHP1* | *ItfHP1* | 0.05892 | 0.086183 | 0.68366 |
| 20 | *IbHP1* | *ItfHP2* | 0.237601 | 2.033642 | 0.116835 |
| 21 | *IbHP2* | *ItfHP3* | 0.092287 | 0.698669 | 0.132089 |
| 22 | *IbHP2* | *ItfHP1* | 0.201198 | 1.86368 | 0.107957 |
| 23 | *IbHP2* | *ItfHP2* | 0.005631 | 0.062864 | 0.089577 |
| 24 | *IbHP3* | *ItfHP2* | 0.085438 | 0.44732 | 0.191 |
| 25 | *IbHP3* | *ItfHP3* | 0 | 0.060334 | 0 |
| 26 | *IbHP4* | *ItfHP4* | 0.024745 | 0.072433 | 0.341621 |
| 27 | *IbHP5* | *ItfHP5* | 0 | 0.010453 | 0 |
| 28 | *IbHP5* | *ItfHP4* | 0.263589 | 2.029558 | 0.129875 |
| 29 | *IbHP10* | *ItfHP6* | 0.019029 | 0.044957 | 0.423258 |
| 30 | *IbRR1* | *ItfRR4* | 0.155071 | 1.107329 | 0.14004 |
| 31 | *IbRR3* | *ItfRR4* | 0.059808 | 0.157438 | 0.379879 |
| 32 | *IbRR5* | *ItfRR1* | 0.055266 | 0.07433 | 0.743517 |
| 33 | *IbRR4* | *ItfRR3* | 0.018226 | 0.077405 | 0.235459 |
| 34 | *IbRR6* | *ItfRR10* | 0.229809 | 1.506075 | 0.152588 |
| 35 | *IbRR6* | *ItfRR13* | 0.228008 | 0.999213 | 0.228188 |
| 36 | *IbRR6* | *ItfRR12* | 0 | 0.051193 | 0 |
| 37 | *IbRR7* | *ItfRR13* | 0.199999 | 0.256438 | 0.779911 |
| 38 | *IbRR7* | *ItfRR12* | 0.261399 | 1.307999 | 0.199846 |
| 39 | *IbRR10* | *ItfRR6* | 0.225232 | 1.672271 | 0.134687 |
| 40 | *IbRR11* | *ItfRR6* | 0.007341 | 0.086032 | 0.085329 |
| 41 | *IbRR12* | *ItfRR7* | 0.005423 | 0.079839 | 0.067918 |
| 42 | *IbRR14* | *ItfRR2* | 0.067057 | 0.112776 | 0.594607 |
| 43 | *IbRR14* | *ItfRR9* | 0.266655 | 0.78395 | 0.340143 |
| 44 | *IbRR15* | *ItfRR8* | 0.024531 | 0.079923 | 0.306933 |
| 45 | *IbRR17* | *ItfRR9* | 0.007342 | 0.012807 | 0.573255 |
| 46 | *IbRR18* | *ItfRR2* | 0.207543 | 0.590256 | 0.351615 |
| 47 | *IbRR18* | *ItfRR8* | 0.135826 | 0.592878 | 0.229097 |
| 48 | *IbRR18* | *ItfRR9* | 0.003661 | 0.012821 | 0.285534 |
| 49 | *IbRR19* | *ItfRR10* | 0.005361 | 0.073285 | 0.07315 |
| 50 | *IbRR19* | *ItfRR12* | 0.288856 | 2.277244 | 0.126845 |
| 51 | *IbRR20* | *ItfRR20* | 0.229798 | 0.816697 | 0.281374 |
| 52 | *IbRR20* | *ItfRR22* | 0.014771 | 0.047997 | 0.307747 |
| 53 | *IbRR21* | *ItfRR20* | 0.222001 | 0.769833 | 0.288375 |
| 54 | *IbRR21* | *ItfRR22* | 0.183173 | 0.799851 | 0.229009 |
| 55 | *IbRR22* | *ItfRR20* | 0.00325 | 0.011342 | 0.286529 |
| 56 | *IbRR26* | *ItfRR17* | 0.003485 | 0.024254 | 0.143691 |
| 57 | *IbRR29* | *ItfRR14* | 0.030574 | 0.059992 | 0.509643 |
| 58 | *IbRR30* | *ItfRR23* | 0.668869 | 2.198 | 0.304308 |
| 59 | *IbRR32* | *ItfRR23* | 0.020397 | 0.03699 | 0.551416 |
| 60 | *IbRR35* | *ItfRR27* | 0.010268 | 0.055892 | 0.183717 |
| 61 | *IbPRR1* | *ItfPRR2* | 7.82E-04 | 0.022322 | 0.03501 |
| 62 | *IbPRR3* | *ItfPRR4* | 0.247872 | 1.034609 | 0.23958 |
| 63 | *IbPRR3* | *ItfPRR5* | 0.016995 | 0.04038 | 0.420871 |
| 64 | *IbPRR4* | *ItfPRR4* | 0.011399 | 0.03756 | 0.303493 |
| 65 | *IbPRR4* | *ItfPRR5* | 0.268364 | 1.070804 | 0.250619 |
| 66 | *IbPRR6* | *ItfPRR8* | 0.006574 | 0.040286 | 0.163193 |
| 67 | *IbPRR6* | *ItfPRR7* | 0.238919 | 0.841133 | 0.284044 |
| 68 | *IbPRR7* | *ItfPRR9* | 0.264971 | 0.765994 | 0.345918 |
| 69 | *IbPRR7* | *ItfPRR8* | 0.238753 | 0.87004 | 0.274417 |
| 70 | *IbPRR7* | *ItfPRR7* | 0.043572 | 0.086532 | 0.503532 |
| 71 | *IbPRR8* | *ItfPRR7* | 0.254698 | 0.89984 | 0.283048 |
| 72 | *IbPRR12* | *ItfPRR11* | 0.008839 | 0.018128 | 0.487607 |
| 73 | *IbPRR14* | *ItfPRR10* | 0.119235 | 0.176521 | 0.675471 |
