# Supplementary material for: Genome-wide identification and expression analysis of two-component system genes in sweet potato (Ipomoea batatas L.)
Source: Front Plant Sci. 2023 Jan 12;13:1091620. doi: 10.3389/fpls.2022.1091620 (PMC9878860; doi:10.3389/fpls.2022.1091620)
Supplement: Supplementary file 2 [file DataSheet_2.zip › Supplementary Dataset 2. Protein sequences of TCS members in Ipomoea trifida.docx]

**Protein sequences**

>ItfHK1a

MAVGSNTSPVSSESLSPSITPKGSFLERILCRMFSSGMFCTSNQSPSSRRNFSRDVEEEEFQDASTLCLSSYYSVFVVRLAIMVMLAILIGLLTLLTWHFTRVYTTRSLNTLAFGLRHELLQRPILRMWNILNSTVEIATAQVKLSEYVIKRYSKPVNQAQQAELYEVMRDVTWALFASRKALNSITISYKNGFVQAFHRDHRSNNTFYIYSDLSNYSISGTYDVSMLTSRQGWNDQSIHNNTTAIWYRETLDPLTGVRVGRKSQIPPDELINIAGISQVPDGAATWHVAVSKFSDSPLLSSALPVWDASNESIVAVVGVTTALYSVGQFMKEIVEFHSGHIYLTSQEGWLLATSTSTPLLRNSTTRPELIMAVDSEDPVIKAGAQCLQKEYGNKFPPNNEVHIENAKLGDQMYYIDSFFLNLKRLPMVGVIIIPRKYIMGKVDERAFKTFVILISASICILFIGCVCIFILTNGVSKEMKLRAELIRQLDARRKAEASSNYKSQFLANMSHELRTPMAAVIGLLDILIYDDCLTNEQYATITQIRKCSTALLRLLNNILDISKVESGKLVLEETEFDLTRELEGLIDMFSVQCINHNVETVLDLSDEMPKLVKGDSGRVVQIFANLISNSLKFTTSGYIVLRGWCESLNDLTNSRNFFFNQKDSWSAPKVKLKRAERRPFKKDSKTVLWFEVEDTGCGIDPNKWESVFENFEQADPSTTRLHGGTGLGLCIVRSLVNKMGGEIKVVKKNGPGTLMRLYLLLNAPTDSAEQHSPPTLAEQTTTVLLALNGRMGRLIMSKWLEKNGLHTCEAADWNELTQMLQGDFGSKSSLQDSGCEHFSDNSSTLLIVVIDIGLLNLSTNIWKEQLNFLDKYSERAKFAWVLYHDTSNSIKSELRKRGHLMMVNRPLYKGKMIQILEAAFTKDKNLELQSAENTAIQVNMHECHHEIDASHSCLTSPDDSDKSETGNVRPVRTFLAEEKPNKHFRNVSSSSIYATLNNYFVDITQPNLGEDDASREDDRREKRNRSEEHSGSTRCVELSTVSSSKTANEQKSLSGLQILLAEDTPVLQRVATIMLEKMGATVVVVGDGQQAVDALKFCRNGPNGSSQEDDTSPTSPTEGFCSPPYDLILMDCQMPKMDGYEATKAIRRSEMETGTHIPIVALTAHAMSSDEAKCLEVGMDAYLTKPIDSKLMVSTILSLTTRKN

>ItfHK1b

MAYRANRTPSISSESSSTPTTPVGSLPERILHKMFGFGNLYRRNQSPTRRRIFRRDVEEEEEEFQYASTLCLSSYYSVFVVRLAIMVMLAILIGLLTLLTWHFTRVYTKRSLNTLAFGLRHELLQRPILRMWNILNSTVEIATAQVKMSEFVMRRYSKAINQEQQVELYEAMKDVTWALFASRKALNSLTINYRNGFVQAFHRDHRSNNTFYIYSDLSNYSISGTYDAGMLSSRDGWNDQSIHGNTSAIWYREPLDPLSGVRIGKQSQIQPDELINIAGISQVPDGAASWHVAVSKYSDSPLLSAALPVWDPSNKSIVAVVGVTTALYSVGQLMKEIVEFHSGHIYLTSQEGWLLATSTNTPLLVNSTTRPELIMAIESEDPVIQAGAQCLQKEYGNKIPPGHEVHIENAKLGNQLYYIDSFFLNLRRLPMVGVIIIPRKYIMGKVDERAFKTLVILISASVCILIIGCVCIFILTNGVSKEMKLRAELISQLDARRKAEASSNYKSQFLANMSHELRTPMAAVIGLLDILICDDCLTNEQFATITQIRKCSTALLRLLNNILDLSKVESGKLVLEETEFDLSRELEGLVDMFSVQCINHNVETVLDLSDDMPKLVKGDSGRVVQIFANLLSNSLKFTSSGYIILRGWCENPNTLANSRKFSVNQKDSWSAPKVKLKPHGNHARRPSKKDNNKTVLWFEVDDTGCGIDTSKWESVFESFEQADPSTTRLHGGTGLGLCIVRTLVNKMGGEIKVVKKNGSGTLMQLCLLLNTPIDVTGQHGHLNFREQTMTVLLALNGRMGRLIMSQWLEKNGVHTCEASEWNELTQMLQRLSKTKTNSQGAGNANTSLFVIVIDIGLLDLSTNIWEEQLNFLDKYCGKAKFAWILYHDTANTIKSELRRRGHLLMVNRPLYKGKMIQILEAIVKENSLELQSAVNTTEENLHECHEIDANHSCIASPDDSDNSENGKDKAVNAFRAEERGNEHFAKASSTSQYGTLNNYFVDFTQTNLEDNTSPEDQPRQARNRSVECLGSPHPRESTVSSSNETNQQKSLAGLTILLAEDTPVLQRVATIMLEKLGAKVVVVGDGQQAVDALKSREEGSSTTTQTEGPCSMAFDLILMDCQMPKMDGYEATKAIRRSEVATGSHIPIVALTAHAMSSDQAKCLEVGMDAYLTKPIDSKLMVSTILSLTKSLQA

>ItfHK2a

MIPPQHTKITSFGLACLSFLLPLCSTKRMSMNCKVHGMKGGFSSKFRLKKARESQHGPSRWRRQLLFLWLFFVAIGFIWLLISSSYGRLGRKVEAPPHLDGDTTNFLLQHFNVSREEIHSLASNFLDTDQISLLKCSGSPRYESSVLKSENQVYEKKCKLGEKIEAYGQCPVSDENTFRNIDSVLQQTSTPFLSHCAPSSISSDHQFCEKETLQVRALGDQCKDIAFCFTKIFWWILLGIAVSWKLRWLRAESGRNEQQKLVSQQEFGQQPQLLEHLQQQQAHVASRVSRKLWEKLLVAFVLSGVIASIWFFWYLNEDIMFWRKETLASMCDERARMLQDQFNVSMNHVHALAILVSTFHHGKQPSAIDQRTFEEYTERTAFERPLTSGVAYALRVLHSERENFERQHGWAIKKMESEDQSLAQEYMPGNLDRAPDKDEYAPVIFSQQTVSHIVSIDMMSGKDDRENILRARASGKGVLTSPFKLLKSNNLGVVLTFAVYNTHLAPDATPDQRINATVGYIGASYDVPSLVEKLLHQLASKHTIVVNVYDTTNTHSPIKMYGADETETELLHVSNLDFGDPARKHEMHCRFKQKPPPPWIAIGASIGVLVITLLVGHIFHAAIARIAKFEHDYQKMMNLKHRAEAADIAKSQFLATVSHEIRTPMNGVLGMLQMLMDTNLDATQRDFAQTAHASGKDLISLINEVLDQAKIDSGRLELEAVPFDLRAVLDNVLSLSSGRSHEKGIELAVYVSDQVPKMVVGDPGRFRQIIANLVGNSIKFTKNKEGHVFVTVHLADEVRCPLDVKDEVLRESLSLVQDQTNRSFNTLSGFPVVDRWRSWQNFKKLSEEESDKIKLLVTVEDTGVGISLEAQGRIFTPFMQADSSTSRTYGGTGIGLSISKHLVDLMGGEIGFFSEPGTGSTFSFTAAFSRDQRGSVEAKWQQYDTGVLDFHGLRALVIDGKRIRAEVTRYHLQRLGLNVKITSTVDHACSYLSTCSKTSEPEHLVIMIFIDKDNWDTENSFALRNIVKDLRPYGSTTLNGATPKLFLLATEMSSTECNQLKSDGLVDNVLIKPIRLSVLASCLQEATGFTYKRQVTMPKPSTLGNLLKEKQILVVDDNIVNRRVAEGALKKYGAIVTCVDGGKAALALLKPPHNFDACFMDLQMPEMDGFEATRQIRKLENEYKETINSGEILVDAPGKLAHWRLPILAMTADVIRASNEECMRCGMDDYVSKPFDEGQLYSALARFFESG

>ItfHK4

MQEDRENILRARATGKAVLTSPFRLLGSNHLGVVLTFPVYKSMLQANPSQQDRIEATAGYLGGAFDVESLVENLLGQLAGNQAIVVNVYDITNSSDPLVMYGQPGEEGDLSLTHVSKLDFGDPFRKHEMICRYLQKAPTAWAAVTTAFFIFVIGFLVGYMIYGAGIHIIKVEDDFHEMEALKVKAEAADIAKSQFLATVSHEIRTPMNGILGMLALLLDTDLSSTQRDYAQTAQACGKALITLINEVLDRAKIEAGKLELEIVPFDLRSILDDVLSLFSEKSRKKGVELAVFVSDKVPEIVFGDPGRFRQVITNLVGNSVKFTERGHVFVQVSLAEEAKAKSEACLNGGSERFIPSSGYHCETLSGYEVADNRNTWDSFKHVIPDEPLYYRAANKLMTDDASQNVTLMVSVEDTGIGIPLHAQDRVFTPFMQADSSTSRNYGGTGIGLSISKCLVELMGGQINFISRPDVGSTFSFTVNFQRYETNGSVDLKKGLSDDLPMSFKGLRAIVVDGKPVRASVTKYHLKRLGILVEVVNSIKKAAAVFGKNGSLISKGQLQPDMILVEKDVWISEDGGGLSLQIPNLKPNGHTYKVPKMILLAVDISSAEFEKAKAAGFADTIIMKPLRASMVGACLHQVLGMGKKTQGKDACNKSTLRGLLCGKRILVVDDNRVNRRVAAGALKKFGADVECAESGAAALALLQLPHNFDACFMDIQMPEMDGFEATRRIRKMENEANERVNGGLEGEGRHKWHVPILAMTADVIHATLDKCLKIGMDGYVSKPFEEENLYKAVAKFFESKPMPDV

>ItfHK2b

MSWNCKNLGMKGSLSSNFRLRKLLSGGWRWRRKYMILWLIFVAIGLIGLLISLNNGLMRRKVEAPDLDEDSTNLLLEHFNVSKERIQVLSAENVVYQKQYELAIEKLEANGQCPVPDENTLTNLDIVVQQIPLPISHCASLATSSDHQFCEKEPLQGRALGDQCKDAAFYFTKVCWWILLGIAISWKLCWLCGESGGNDRQKQVQQQELPQQPQLLQHLQQQQAQTSSRIARKWWEKLLVISVSVGVMGSIWLFSHLNEEFTVRRKETIASMCDERARMLQDQFNVSMNHVHAWAFLVSTFHHGKQPSAIDQKTFEEYADRTAFERPLTSGVAYAIKLCHSERENFEKQQGWTIKKMESEDQSLAQEYISGNLDPAPIQDEYAPVIFSQQTISHIVSIDMMSGKEDRENILRARASGKGVLTSPFKLLKSNNVGVILTFAVYNTDLPPDATPEQRINATLGYFGAAYDFPSLVEKLLHQLASKHTIVVNVYDTTNASAPIRMYGMEEADLDETDRELVHVINLDFGDPARRHEMHCRFKQKRPPPWTAIAASIGVLVITLLLGHIFHAAINRIAKFERDYQKMMDLKHRAEAADIAKSQFLATVSHEIRTPMNGVLGMLQMLMDTNLDATQLEYAQTAHASGKDLISLINEVLDQAKIESGRLELEAVAFDLRAVLDKVLSLCSGRSHEKRIELAVYVSDQIPEVVIGDPGRFRQIITNLVGNSIKFTKEKGHVFVSVHLADEVKSPNDVKDEVLRQSLTLVQDRPNTCFNTLSGFPIVDRWRSWQNFKKLSEEKTENIKLLVTVEDAGVGIPLEAQGRIFMPFMQADSSTSRTYGGTGIGLSISKRLVELMGGEIGFFSEPGTGSTFSFTAAFARAEEGLLESKRQRNDPSVSELRGLRALVIDDKSIRAEVTRYHLQRLGLNVKIISKMDCSCSHLSTCLEASPLEHLALIFIDKDNWDDETSITLSKILKELRANSSNVVSGVIPKFVLLATNMSATNRNELRSAGLVDSILIKPLRLSALVSCIQETTGFMNKRHITRRKPSSLGSLLKGKRILVVDDNVVNRRVAEGAIRKYGAIVSCVDSGKAALALLKPPHKFDACFMDLQMPEMDGFEATRQIRCLESKYNENINSGEVLIEMHGKVSHWHTPILATTADVIQATNEKCLQCGMDDYISKPFDEWQLYSAVARFFESS

>ItfHK3

MSLLHVIGFGLKLGNLLLTLCSLVVTLISMNWLSNGGVMTTKTLLDDGEEILTKLWGKISENISKIQHSYSQYIGSKKVRKNWWGLLVIWLGFGAVLAFCAFWCLSTQAMEKRKETLASMCDERARMLQDQFNVSMNHVQAMSILISTFHHGKNPSVIDQRTFARYTERTAFERPLTSGVAYAVRVLHPEREQFEREQDWTIKRMDPQFHENEYNVDNLEPSPIQEEYAPVIFAQDTIAHVISVDMLSGKEDRENVLRARASGKGVLTAPFKLLKTNRLGVILTFAVYKKDLPSNATPNERIEATYGYLGGVFDIESLVEKLLQQLASKQTILVNVYDTTNLSDPISMYGTNVSIDDLEHVSSLNFGDPFRKHEMHCRFKQKPPWPWLAIITSFGIITIVLLLGHIFHATINRIAKVEDDYHEMMELKKRAEAADVAKSEFLATVSHEIRTPMNGVLGMLHMLMDTELDVTQQDYVRTAQASGKALVSLINEVLDQAKIESGKLELEAVSFDPRAILDDVLSLFSGKSQEKGVELAVYISDKIPKLLIGDPGRFRQIITNLMGNSIKFTEKGHIFVTVHLAEEVVVEHESSYALSGFSIEHESSSTLSGFLVADRRQSWKKFKAFQEGFSSFKLTSDQINLIVSVEDTGVGIPFEAQSRVFTPFMQVGPSIARIHGGTGIGLSISKCLVHLMKGEIGFVSLPKTGSTFTFTAVFANGSFSSNELKGQHINDESNSVFSEFKGMRALVVDPRPVRAQVSKYHIQRLGIYVKVVPDLNHGYTCISTEKTNINIVLVEQEVWDMDSGMATEFVEKLRSYDISCSPKLFVLANCASATRANASNFGVSTPFVIMKPLRASMLAASLQRALGVNNRGNYRNGGLSGVPLSELLHKRKILVVDDNPVNLRVANAALRKYGADVVCIDSGEQAISHLRPPHRFDACFMDIQMPKMDGFEATKRIRELERQANSQNEHGELLVNASNWHVPILAMTADVIHATNEQCLKCGMDGYVSKPFEPEQLYREVSRFFHVKSN

>ItfCKI1

MVNFVRSLKTMWPVYLALTLCLAGLGIAGWLISVLVAIQHHTTQGGAKVEKRDVKLGMILLGINICITVTSVVVLTWWRSRVMMREMCVKAALIKQKEATEEAERKSMSKSVAVANASHEVRTALAGITGLIQMCRADADASAAHSELNDNLRHMESCTNDLYSLLNSILDASRIEAGKMQVEEDEFDLQELLEDVVDLYYPVGMKKGVDVILDPCDESVEKFRRVRGDRGKLKQVLSNLLFNAIKFTDEGYVALRVWARKPSPRPPSQSPPKPKRASSSSPIAILKGFVATFCGVPAKTGGGEEEEEEVNDSVLERKDGGVEYIFKVVDTGKGIPKEKRNSVFENYSQVKDMGRGKKHQLGHGLGLGIAQSLVRLMGGEIGIEDKETGERGTCFKFNIVLDNIVILESSSSSHNNNINTYSSGHHVVVFMHCEERGKIIGRFLENRGIKVSLVQKGHQQLSRKLKKIKRGALNLPRSTTTPLPSYYSSSSSSKEELEDETMPLHTNTCTVLIIIDTSAAGEALFPEVIKAVSEFHRDLQPGCVRVLWIDTTALGRGVDNNFQLPSTDLIISKPLQGSRLHSVLGLLPDFASSSQLGEIQVVIEKDKEEEDEDNGGGSSSSEKKALTGKRILVVEDNPTLRKICTTLVSSLGALTYACTNGEEALQLVSSGLQDHHHQPPFDYILMDCEMPIMDGFEATKRIKEEGKAMGIWIPIIALTAHTGKEDMDKVTEAGMDYYLSKPINAATLLTAIHFLDKSTTHLL

>ItfERS1

MESCDCVEILLPTDELLVKYQYISDFFIAFAYFSIPLELIYFVHKSAFFPYRWVLMQFGAFIVLCGATHLINLWTFSSHSKTVAIVMTIAKISTAIVSCVTALMLVHIIPDLLSVKTRELFLKTRAEELDREMGLIIKQEETGRHVRMLTHEIRSTLDRHTILRTTLVELGRTLDLAECALWMPTQRGMVLQLSHTLNNLIPVGSTVPINLGIINDIFNSSGAILIPHSCELAKMRSTNTGRHVPPEVAAVRVPLIHLSNFQINDWPELSAKSYAVMVLILPMNGIRKWREHELELVQVVADQVAVALSHAAILEESMRAHDQLMQQNIALDLARQEAEMAIHARNDFLAVMNHEMRTPMHSVIALCSLLLETDLNPEQRVMMETILKSSNLLATLINDVLDLSRLEDGSLELENVTFNLHGVFREVVNMIKPIAAVKKLSTTLSLALDVPIHAVGDAKRLTQIMLNVAGNAVKFTKEGQISIEASVAKPDYIRGSRQGEFYPPSTEGHFYLRMQVKDSGCGISPQDIPLIFTKFTEARSASNRSNSGAGLGLAICRRFVQLMGGHIWIESEGLGKGTTVTFIVKLGSCNYPNAPAIVAPRGRANQGSDDLFKYRQYHRADGSMYAPVARYQRSL

>ItfETR1a

MESCNCIDPQWPADELLMKYQYISDFFIALAYFSIPVELIYFVKKSAVFPYRWVLVQFGAFIILCGATHFINLWTFGMHTRTVAIVMTTAKLLTALVSCVTALMLVHIIPDLLSVKTRELFLKNKAAELDREMGLIRTQEETGRHVRMLTHEIRSTLDRHTILKTTLVELGRTLGLEECALWMPTRTGLELQLSYTLRHQNPVGFTVPIHLPVISQVFHTNRAVKISPNSPVARLRPAGKYIPGEVVAIRVPLLHLSNFQINDWPELSTKRYALMVLMLPSDSARQWHVHELELVEVVADQVAVALSHAAILEESMRARDLLVEQNIALDLARREAETAVRARNDFLAVMNHEMRTPMHAIIALSSLLQETKLTPEQRLMVETILKSSNLLATLINDVLDLSRLEDGSLQLEIGTFNLQALFWEVHNLIKPIASVKKLSVALSLSSDLPEYAIGDEKRLMQVLLNVVGNAVKFSKEGSISVSAFVAKSEFLRDPQAPDFFPVITENHFYLRVQVKDTGVGINPLDIPKIFSKFAQNQSLATKNSGGSGLGLAICKRFVNLMEGHIWIESEGLGKGATAIFIVKLGIPGLSNELKPTLVPKLPANHIHTIFLGLKVLLMDDNSMSRMVTKGLLAHLGFDVTTANSGDECLRVVNQEHKVVIIDVSMAVVDGYKLSNQIHEKFSKCHERPFIVGLIGTTDRAMKEKCLRAGMDGVILKPISVEKMRNVLTELFEHGVVLDAQ

>ItfHKL6

MSTSRPSQSSSNSARSKHSARIIAQTSIDAKLHAEFEESGDSFDYSSSVRVTSVDAGVQKPRSDKVTTAYLHQIQKAKYIQPFGCLLALDEKTFKVIAFSENAPEMLTMVSHAVPSVGDHPVLGIGTDIRTIFTSPSAAALQKALGFGEVSLLNPILVHCKTSGKPFYAIIHRVTGSLIVDFEPVKPYEVPMTAAGALQSYKLAAKAIARLQSLPSGSMERLCDTMVQEVFELTGYDRVMIYKFHDDDHGEVVSEITKPGLEPYLGLHYPATDIPQAARFLFMKNKVRMICDCRAKHVRVVQDEKLSIDLTLCGSTLRAPHSCHLQYMENMNSIASLVMAVVVNDGDDEGEASESGRIQKRKRLWGLVVCHNTTPRFVPFPLRYACEFLAQVFAIHVNKELELENQIVEKNILRTQTLLCDMLMRDAPLGIVSQSPNIMDLIKCDGAALLYKSKVHRLGITPTDFQLHDIVSWLSEYHMDSTGLSTDSLYDAGFQGALALGDAICGMASVRISDKDWLFWFRSHTAAEVRWGGAKHEPDEKDDGRKMHPRSSFKAFLEVVKTRSLPWKDYEMDAIHSLQLILRNAFGKEADTMDTKANANAIHSKLNDLRIDGMQELEAVTSEMVRLIETATVPILAVDVDGLVNGWNTKIAELTGLTVDEAIGKHFLTLVEDSSIHNVRKMLSLALQGKEEKNVQFEIKTHGQRSESGPISLIVNACASRDVQESVVGVCFIAQDITGQKTIMDKFTRIEGDYRAIIQNPNPLIPPIFGTDEFGWCSEWNSAMTNLSGWCRDEVMDKMLLGEVFGTQKACCRLKNQEAFVNLGVVLNNAITGQVSEKTRFGFFARNGKYVECLLSVSKRLDQEGAVTGLFCFLQLASQELQQALHFQKLSEQTAMKRLKVLAYIRRQVKNPLSGIMFSRKMLEGTELGKDQKSILHTSAQCQQQLSKVLDDTDLDCIIEGYLDLEMVEFKLDEVLQASISQVMTKSNGKSLRIINDIADNILCETLYGDSLRLQQILSEFLSVAVNFTPSGGQLALSSKLTKDNLGESIQLAHLEFRLTHTGGGVPEELLTQMFGSEADASEDGISLLISRKLVKLMNGDVQYLREAGRSTFIISVELAVASKPSS

>ItfHKL4

MKWIKECRTIGTMLRMSVLKLLVLSVHIAIAAADNGFARCNCEYEGFWSIENILECQKVSDFLIAVAYFSIPIELIYFISCSNVPFKLVLFEFIAFIVLCGMTHLLNGWTYYGQHSFQLMLALTVFKVLTAMVSFATAITLISLIPLLLKVKVRELMLKKKAWDLGREVGLIKKQREAGWHVRMLTQEIRKSLDRDTILETTLSELSKTLGLHNCAIWMPNQDRTVMNLTHEVRERNFSDVNDFLIPILDTDVQEIKASDEVKLLEPSSPLAAASSGRSSEPGCVAAIRMPMLRVANFKGGTPELVPACYAILVLVLPSGQGRSWGSQEIEIVKVVANQVTVAISHAAVLEESQHMRDKLAEQNRELQQAQQGALRANQARNAFQMVMSNGMRRPMHSIFGLLSILQEDENLNSEQHLLINATVKTSNVISNLITDVMDCSTKDNRKFPLETRCFELHSMIKEAVCVAKCICAYKGYEFSVEVDKSLPNHVMGDERRAFQVILHVVGNLLKNSNGGCLKFHVVPERSSQGGNDLGWRTWRSNSSRENVFVRFEIGVHGNNSQPEHTTSKVLNPNQKYCGKDFEGSLSFSVCKKLVQLMQGDIWVSPNPMGFDQQVMAVVLGFQLRPSVVIGISEYGDSSNRTHSDSLFPGLNVLLADYDDVNRAVTRRMLEKLGCIVSSVSSGYECLGCLGTTISPFQIVLLDLHLPDLDGFEVTMRIRKFKSRNWPLIVALTSNNDASIRGRCFQVGMNGVICKPLFLQGIADELQKVMLIASRTLS

>ItfHKL3

MSKILALRVLVWVSLVALTAGDNGFFRCNCDYDGFWSIETIMEWQKVGDFLIAVAYFSIPIELLYFVSCSNAPFKLILVEFIAFIVLCGMTHLLMGWTYYGQHSFHLMLALTIFKVLTALVSFATAITLVTLIPLLLKVKVREFMLKKKTWDLGREVGMIKKQKEAGWHVRMLTREIRKSLDRHTILYTTLIELSKTLDLHNCAIWMPNEEKTEMDLTHEVRGRSFVDGHNFPIPVLDPVVQEIKQSVEVKLLDPDTPLAVASSGGVCEPGSVAAIRMPMLRVANFKGGTPELVPQCYAILVLVIPAGQGRCWGNQEMGIVKVVADQVAVAISHAAVLEEVQNMRDKLEEQNRALHQAQQDALRASQARNSFQMVMSNGMRRPMHSILGLLSVLQDEQLNCEQKLLRDTLAKTSNVLSTLINDAMDTSTKQNRRFQLEMRSFQLHSMIKEAICLAKCLCTFKGYEFVVEVDKSLPNHVIGNEIRVFQVILHMVGNLLKSSGGGCIKFSVTREKDGQGGKDLGWRTKSSSEHVHVRFEIGIAGNCSKPEGVYKASHCSEAYGRREVEEVLSFTVCKKLVQLMQGNISVVPNPKGFHQSMAVVLGFQLGPSTSGMSGCSESSSLTHPSSLLAGLKVLLADHDGINRGVTRRLLEKLGCNVSAVSTGYECLGALGPAACPFQVILLDLHLPELDGFEVTMRIRKFRSRSWPLIIALTANDDEDASDRCIQVGMNGIIRKPVILQGIADELTRVLLLKSRNIA

>ItfHKL1

MGAAMLRWLFLGLLVSSIFSAVSAIDYLCCDDEGLFSVSNILFMQKVGDVLIAVAYFSIPIELLYFISCSNIPFKWVLVQFIAFIVLCGLTHLLNVWTINTQPSFQMIMSLTVAKILTALVSCATAITLLTLIPLLLKFKVRELFLRQNVLELDQEVGMMKKQKEASMHVRMLTLEIRKSLDKHTILYTTLVELSKTLNLQNCAVWMPSGNRAEMNLTHELNPCSAREHHSLSINDPDVLEITKNEGVRLLKQDSVLAAASSGGSGQPGAVAAIRMPLLRGSNFKGGTPELIETCFAILVLVFPSVNDGDSSYDELEIVEVVADQVAVALSHATVLEESQSMQEKLKERNRVLQQAKEDAMKASQARNSFQKVMNNGMRRPMHSILGLLSILQDDNLKPEQKIVVDTLVKTSTVLSTLISDAMEISAKDDGKFPVEMRPFQLHSLIREASCLVKCFAIYKGFDFSTDVPSSLPNQVMGDEKRTFQVILHMVGHLFNVSDGNGSVIFRVASESGTEDGNNKVWNTRKPSSSDDNVTIKFEIEVAIGDSQSGTSVSVVPSGRKRHNSKDVTEGLSFTMCKKLVQLMQGNIWVSSNSRGRGQGMTLILRYQKQSSIRRRIFEYRNPSEQPLPSTMFEGLQVLLADDDDVNRMVTKKLLEKLRCQVSTVSTGFECLSALGPSATSFQVIILDLHMPEMDGFEVAMRVRKFRSRNWPLIIALTASSEDHMWERCLQVGMNGLIRKPVLLQRLAEELQRVLQRAGSEVM

>ItfHKL2

MLRWLFLALFIASVFVSVSAIDCHCDEEGVWGIESILECQKVSDFLIAVAYFSIPLELLYFISCANIPFKWVLVQFIVFIVLCGLTHLLNGWTFSAQPSFQLIVSLTVVKILTALVSCATAITLLTLFPLILKIKVREIFLRQNVLELDQEVDMMKRQKEASLHVRMLTREIRKSIDKHTILYTTLVELSKTLNLQNCAVWMPNEKGAEINLTHELNPGAAARKKCSLSINDRDVLEIKKIKGVRILRQDSVLAAASSGGTGEPGAVAAIRMPLLQVSNFKGGTPEIFSPRYAILVLVLPSTSDHSVWGNNEVEIVEVVADQVAVALSHATVLEESQSMREKLKERNHVLQRAKEDAMKASHARDSFQKVMNNGMRRPMHSILGLLSILQDDNINPEQRIIVDTMVKASTVLSTLMSDAMEITAKHNGKFLVEIRLFHLHSLIMEASSIVKCMSVYKGFGFLADIPNSLPNQVMGDEKRTFQVLLHMVGHLLNVSDGKGSVIFRVVQESGTEEGNNKVWNTRKPSPADDWVTIKFEIEVSVEGSRPDSSVSTIHFGAGRHNCKDVKKGLSFNICKKLVQMMQGNIWMSSDSQGRAQSMTLILRFQKQSSYRRRVFEFKNPREKQLSSSTLEGIQVLLADDDDVNRMVTKKLLGKLGCEVFAVSTGFQCLSALAPSGASFQVIILDLHMPEMDGFEVATRVRNSFRGRGSRPVIIALTASSEEHMWEKCNQVGMNGLIQKPVLLQRLADELQRVLHSAREGP

>ItfHKL5

MMEFWVFILNCEFGFPGMMLKLLASGLFISSFLIVLAAADNGVRCNCDDIEGVWSIESILECQKVSDFLIAVAYFSIPIELLYFISCSNIPLKLVLFEFIAFIVLCGMTHLLSGWTYYGQHPFQLMLALTVFKVLTAMVSFATAITLITFIPLLLKVKVREIMLKKKAQDLGREVGMIKKQKEAGWHVRMLTQEIRKSLDRHTILYTTLIELSKTLDLCNCAIWMPNVGKTEMNLTHEVRGKDFSNLYNYSIPILDPDVQEIKKSIEVKLLDPKSALADASSGGTSEPGGVAAIRMPMLRVANFKGGTPELVPACYAILVLVIPAGQGRCWGNQEIEILKVVADQVAVAISHAAVLEESQHMREKLVEQNRSLQQAQKDALRANLARNGFQMVMSNGMRRPMHSISGLLSILQDEKLNREQKLLADAMAKTSNVLSNLINDVMDTSSKENGKFPLEFRSFQLHSMIKEAACLIKCLCALKGNDFAVEVDRSLPNRVMGDERRVFQVILHVVGNLLKISGGGCLKFRVVPEKASQGGNDFRWKTWRSNSSSENVYIRLEIGICSYKSRTEGATSNVSSQKYGSREIEDGLSFSLCRKLVKLMQGEIWMVPNSKGFDQSVAIILPFQLKPSIVLDISGESSNHTNPYFLFEGLEVLLADYDDLNRAVTCRLLEKLGCIVSTVSSGYDCLGALGNGVSSFQVVLLELNLPDLDGFELTMRIRKFQSRGFPLIIALTASSDVDVIGRCLQVGMNGIIRKPVLLQGIADELQRVLLLTNRIISPRE

>ItfETR1b

MGVCAVQAIILCGATHFCGAAVLVCMPKVYNLMKPIASVKKLSVTLSLSSDLPEYAIGDEKRLMQVLLNVVGNAVKFSKEGSVSVSAFVAKSEFLRDPQAPDFFSVITVKDTGVGINPLDIPKIFSKFAQNQSLATKNSGGSGLGHAICKRYALITSNLN

>ItfHK5

MVSEMENAHTEEMDIEVLSSMWPEDINEAGKQFNIEQPGADLDMLEEVTINEEATTIVDFQRLMELTDYSDKGSSQLAYLVKNWEYKQANAVRLLREELDYLSKQQQESELKKLEILEQHRFEEERYGGDKRPVSILDEDLKYIYQDIPRRKKDVVVQHEKLEIEAEYDSIIYWKQRALHLQKLLAASIERENVLLEKLQESIEKLERQSSPVEELSQVLKRADNYLHFVLQTAPIVIGHQDKELRYRFIYNHFPSLREEDIIGKTDVEIFSGSGVKESQDFKKEVLERGLPAKREITFETELFGSKTFLIYVEPVFSKAGETIGVNYMGMEVTDQVRKREKMAKLREEIAVQKAKETELNRTIHITEETMRAKQMLATMSHEIRSPLSGVVSMTEILATTKLEKDQRQLVNVMLSSGDLVLQLINDILDLSKVESGVMKLEATKFRPREVVKHVLQTAAASLQKLLTLEGFVAEDVPTEVIGDVLRIRQILTNLISNAIKFTHEGKVGIKLYVVPEPSLGAKQGSHQKQSLDSLKSSSNNWKEDRCLSASHGKHDRTASFSYKDGEGTFENQMHKDGSNHSVSSGALDDDLDAHPDQEEKTVWICCDVYDTGIGIPENALPTLFKKYMQVGADTARKYGGTGLGLAICKQLVELMGGHLTVSSKEHHGSTFTFVLPHKVSPLCESSDENDEMSDMGSHDTSTDANEDDANSGFFQFQPRTLGSLFSSHGSGRAQKLSPNTFGFNTLQSCNGLPKNSYTFPANSVMLKDMGSVEDACSVIDVDILSDPESSFRQSSHSDNPSTLERDKHAHSGSNGQCHHHSSYSTDSTSTRKDEDVKTAVQEKRQPEGNSPCSSDNNQEVSKSAPKPRILLVEDNKINVMVTQSMMKQLGHQIDIVNNGIEAVRAVQRSCYDLILMDVCMPVMDGLQATRLIRSFEETGNWDAARTAGVEEVPSSSLSLKRSDSKSSNGRIPIIAMTANALSESADECFANGMDSFVSKPVTFQKLKECLQQYLPQRHRL

>ItfHKL7

MSSRSGTIRTNCSMSSSARSRHDARVVAQTSIDAKLHVEFEESEEQFDYSTSVNLSNSTSNIPSSTVSAYLQKMQRGSLIQPFGCLIAIDEHNFSVLGFSENAPEMLDLAPHAVPSIEQQEALTFGTNVRTLFRSTGAAALEKAASFEEVSLINPILVHCKNSGKPFYAILHRIDVGLVIDLEPVNPADVPVTAAGALKSYKLAAKAISKLQSLPSGDISLLCDVLVREVRDLTGYDRVMVYKFHEDEHGEVVAECRKPDLEPYLGLHYPATDIPQASRFLFMKNKVRMICDCLAPSVKVIQDKTLAQPLSLCGSTLRAPHGCHAQYMANMGSIASLAMSVTINEDDDEMDSDQQKGRKLWGLVVCHHSSPRFVPFPLRYACEFLVQVFSVQINKEVELAAQRLEKHILRTQTVLCDMLLRESPVGIVTKSPNIMDLVRCDGAALYYRNKFWLLGATPTEPQIRDIAQWLLDSHSSSTGLSTDSLMEAGYPNASVLGDSVCGMAAVKITAKDFLFWFRSHTAKAIKWGGAKHDPGDKDDGRKMHPRSSFKAFLEVVKRSLPWEDVEMDAIHSLQLILRGSLQDEVVDNSKMIVNVPAVDTSIQRVDELRIVTTEMVRLIETASIPILAVDTSGCINGWNIKVAELTGLVVQEAIGAPLVDLVVSEAVSTIKNVLSLALQGKEEKNVEIKLKKFGSQENNDPVILVANACSSRDVKGNIIGVCFVGQDVTGQKLIMDKYNRIQGDYVGILRSPSALIPPIFLMDEHGRCLEWNDAMQKLTGLKRAEAIDQMILGEVFTVSSFGCKVKDSDTLTKLRILLNGVIAGQDSEDLLFGFFDKQNKYVEALISANKRTDVVGRITGVLCFLHVPSPELQYAIHVQKLSEQAAANSLKKLAYVRREVRNPLNGIKCIQNLMKSSDLSKDQMQLLKTSTMCQEQLAKIIDDTDIESIEESYMEMNCCEFSLGEAIKAVVNQAMIPSRERQVQIMCDLPVEASSLYLFGDNLRIQQVLSDFLTTAVLFTPHFEESSVLFRIIPRREQIGAKMHVVHLEFRITHPAPGIPEELIQEMFNYSQSMSREGLGLYISQKLIKIMNGTVQYLREAERSSFIILVEFPASLRSEHQ

>ItfHKL9

MASGSRSKNVQQNQAQSSGTSNVNYRDSVSKAVAQYTVDARLHAVFEQSGESGKSFDYSQSVKTITQNVPEKQITAYLSKIQRGGHIQPFGCMIAVDEPSFRVIGYSENAREMLGLTPQSVPSLERPEILAIGTDVRTLFTPSSSVLLERAFGAREITLLNPIWIHSKNSGKPFYAILHRIDVGIVIDLEPARTEDPALSIAGAVQSQKLAVRAISHLQSLPGGDIKLLCDTVVESVRELTGYDRVMVYKFHEDEHGEVVAESKRPDLEPYIGLHYPATDIPQASRFLFKQNRVRMIVDCNATPVQVIQDESLMQPLCLVGSTLRAPHGCHAQYMANMGSIASLTLAVVINGSDEEAVGGRNSMRLWGLVVGHHTSARCIPFPLRYACEFLMQAFGLQLNMELQLASQLSEKHVLRTQTLLCDMLLRDAPTGIITQSPSIMDLVKCDGAALYYQGKYYPLGVTPNEAQIKEIVDWLLTYHGDSTGLSTDSLGDAGYPGAASLGDAVCGMAVAYITSRDFLFWFRSHTAKEIKWGGAKHHPEDKDDGQRMHPRSSFKAFLEVVKSRSLLWENAEMDAIHSLQLILRDSFKDAEASNSKAVVRAPPGELELQGMDELSSVAREMVRLIETATAPIFAVDVEGRINGWNAKVAELVGLSVEEAMGKLLIQDLVHKESQETTEKLLFNALRGEEDKNVEIKLRTFGTEEDKKAIFLVVNACSSKDYTNNIVGVCFVGQDVTGQKIVMDKFIHIQGDYKAIVHSPNPLIPPIFASDENTSCSEWNTAMEKLTGWSRGETIGKLLVGEVFGSCCRLRGPDAMTKFMIILHNAIGGQDTDRFPFSFFDRNGKYVQALLTANKRANMDGQIIGAFCFLQIASPELQQALKIQRQQENKCFSRMKELAYICQEIKNPLNGIRFTNSLLEATDLTEDQKQFLETSAACEKQMSKIIMDVDLENIEDGSLELEKEDFFLGRIIDAIVSQVMSLLRERGLQLIRDIPEEIKTLAVNGDQVRIQQVLADFLLNMARHAPVPGGWVEIQVRPSLKQVSDGTNVVHTEFRIMCPGEGLPPELVQDMFHSSRWVSQEGLGLSMCRKVVKLMNGEVQYIRESERCYFLIILELPIPRRGSKSIIIG

>ItfHKL10

MAGSGTGSSSKRFIEHQSSSAQIAQSSGTSNSNNRYSVSKAVAQYTEDARLHAVFERSGGTGKSFDYSESVKVATPFVAEQQIAAYLSNIQRGGHIQPFGCMIGVEEGNFRVIAYSENAREVLGLMPQSVPSLDRPDILGIGVDVRTLFRPSSSVLLQRAFGAQEITLLNPIWVHSKNSGKPFYAILHKIDVGIVIDLEPARSEDPALSIAGAVQSQKLAVRGISRLQSLPGGNIKHLCDVVVECVRELTGYDRVMVYKFHEDEHGEVLAESKRPDLEPYIGLHYPATDIPQASRFLFKQNRVRMIVDCNATPVRVIQDESLKQPLCLVGSTLRAPHGCHAQYMANMGSIASLTLAVIVNGNEDEGVGGRNSMRLWGLVVGHHTSARSIAFPLRSACEFLMQAFGLQLNMELQLASQLAEKHVLRTQTLLCDMLLRDSATGIVTQSPSIRDLVKCDGAALYYKGKYYPLGVTPTEDQIKDIAVWLLTYHGDSTGLSTDNLADAGYSGAASLGDAVRGMAVAYITPKDFLFWFRSHTAKEIKWGGAKHHPQDKDDGQRMHPRSSFKAFLEVVKRRSLPWENAEMDAIHSLQLILRDSFKDAEASNSKAVVHAPPGELELQGMDELSSVAREMVRLIETATTPIFAVDAEGHINGWNAKVAELVGLPVEEAMGKSLVHDLVHMESQETTEKLLFNALRGYEDRNVEIKLKTFGTEQHTKAVFVVVNACSSKDCTNKIVGVCFVGQDVTEQKVVMDKFIHIQSDYKAIVHSPNPLIPPIFASDENACCSEWNIAMEKLTGWSKGEMIGKMLIGELFGGVCRLKGPDAMMKFMITLHHAIGGKDTDKFPFYFFDRNGKYVQTLLTANKRVNMDGQIIGAFCFLQIASPELLQAIKIQRQQENKWLTKSKVMAYICQEIKNPLNGIRFTSSLLEATNLTEHQKQFLETSAACQKQMSKILRDAGLENIEDGSLELEKEEFHFGSVIDAIVSQVMLLLRERGLQFMLDIPDEMKTLKVYGDQARIQQVLADFLLNVVHHAPTPKGWVKIHVRPSLRQSSDGTTIAHVEFRFICPGEGLPSALVQDVFNNSEWETREGLGLSMCRKIVTLMNGEVRYVREAERCYFLVILKLPVPTRGSKSS

>ItfHKL8

MDLQSQENKPPTSKKMENHAKAATFSSSATSNLNTGKAIAQYNADAKLMAEFEQSRESGKSFDYSRSVIGAPQNVTEEEMTAYLSRIQRGGLIQPFGCMLAIEEPSFKIVGFSENCFDLLGLKSGVEPPERMSLIGIDARTLFTLSSRASLAKAVASREISLLNPIWVHSKTNQKPFYAVLHRIDVGIVIDLEPANSADPALLLAGAVQSQKLAVRAISRLQALPGGDIGTLCDTVVEDVQKLTGYDRVMVYKFHDDSHGEVVSEIRRSDLEPYLGLHYPATDIPQAARFLFKQNRVRMICDCNAQPVKVFQSEELKQPLCLVNSTLRSPHGCHTKYMANMGSIASLVMAVVINSSESMKLWGLVVCHHTSARYVPFPLRYACEFLMQAFSLQLYMELQLASQLAEKKILRTQTLLCDMLLRDAPFGIVTQTPSIMDLVRCDGAALYYDGKCWLLGVTPTETQVKDIAEWLLHNHGDSTGLSTDSLSDAGYPGAPLLGDAVSGMATARITSKDFLFWFRSHTAKEVKWGGAKHHPEDKDDGGRMHPRSSFIAFLEVVKSKSLPWEDSEINAIHSLQLIMRDSLQGIGENYMKSVSSPQQTDSEGTRFYELSSMALELVRLVETATVPIFGVDSSGLINGWNAKIAELTGLQANVAIGKYLIDDVTHEDSHETFKGLMCRALQGEEDRNVEVKLLKFGEHPTKEVVYLVVNACTSRDYKNDIIGVCFVGQDITPQKAVMDKFVRLQGDYEAIIQSLNPLIPPIFASDENACCSEWNAAMERLTGLVKCEVIGKRLPGEIFGGLCRLKGQDALTKFMILLYQGISGHDTEKLSFGFFDRKGNFIDVFITANKRTDERGNIIGCFCFLQTMAVDPQTSARDIEDDRECLSTLKEFAYIQQQMKNPLNGIRFTHKLLEGTVTSDHQKQFLETSEACEKQILSIIENMDSGGIVDGNKVELKTEEFVIGNVIDAVVSQVMIPLKEKNLQLLHDIPDQIKSLPIYGDQIKLQLVLSDFLLSIVRHAPSPDGWVEIRVSPGLKLIQDGNEFIHIQFRMTHPGQGLPSALIEDMVRGGTRWTTQEGIVLHLSQKLVRMMNGHVHYVREQQKCYFLIDLDFKTQKPRSRESSMDTSRIT

>ItfHP1

MEVSQLQNSFLGYMAELFREGFLDAQFSQLQQLQDESNPTFVAEVVTLFFEDSERLLNDLNTTLNQPDVDFKKVDAHVHQLKGSSSSIGAQRVKNVCVAFRNFCEEHNIEGSLRCLQQVKQEYLLVKNKLETLFRLEQQIVAAGGAIPIFE

>ItfHP2

MDVVHQLQKQFVDLIASLYREGFLDDQFLQLQKLQDDSNPDFVFEVVSLFFEDSEKLINNLATALQQPVVDFNQVDAHVHQFKGSSSSIGAQRVKNACVSFRNFCEEKNLDGCVQCLQLVKNEYFVVKNKLETLLRLEQQILAAGGKIPILP

>ItfHP3

MEVVGQLQKQFVAYMASLYREGFLDDQFLQLQKLQDQSNPDFVVEVVSLFFEDSEKLINNMANAFQQQVVDFKQVDAHVHQLKGSSSSIGAQRVKNACVSFRNHCEERSLDGCVRCLQVLKNEYFLVKNKLETLIRLEHQILAAGGTIPLLS

>ItfHP4

MPSQAAKLRKSLFDQGYIDDQFIQLEELQDDANPNFVEEVVRLFYNDSTRQIHNIELALGSGACDFNRLDDMMHQFKGSCSSIGARKVKKECSEFQQYCDAGNIEGCRRAFQRLKQEYYTLETKLDTYFQMAKQGS

>ItfHP5

MERNHLPRQLATMRKSLFDQGYLDDQFVQLEELQDDVNPNFAEEVVTLFYRDSARLVQNIEHALERSPLDFAKLDGLMHQFKDSCSSIGARKVKYECTQFREHCRVANAEGCKRSFLQLKKEYSTLQKKLKAYFQFARQAGPVEVACRPN

>ItfHP6

MDVDLLQHQLIAHIQALQREGYVDEYLQICYGLKETSGLTFFLELIATFLTNSAATIHDMTQTMEYPILDYDEMQRLAIRLKGSSSCIGACRISASCSELRQAATKRSKINCKRAVEMISGEKSAWEIKLETIMQVT

>ItfRR1

MGRNMRTEKIAAVDGCSSTFVGGGRELHVLAVDDSYVDRKVIEKMLKISCCKVTVVDSGSRALQYLGLDGEESSVATDGLKVNLIMTDYSMPGMTGYELLKKIKGSSALRQIPVVIMSSEKILARIDSCLEEGAKEFLMKPVKLSDVKRVVDFILRGEAEEDGKEAESTTGSYSSSAPDNTTLSPESYLANIARISLDASNRT

>ItfRR2

MGRNMRTEKIAAVDGCSSTFVGGGRELHVLAVDDSYVDRKVIEKMLKISCCKVTVVDSGSRALQYLGLDGEESSVATDGLKVNLIMTDYSMPGMTGYELLKKIKGSSALRQIPVVIMSSEKILARIDSCLEEGAKEFLMKPVKLSDVKRVVDFILRGEAEEDGKEAESTTGSYSSSAPDNTTLSPESYLANIARISLDASNRTMGMAAAESQFHVLAVDDSLIDRKLIERLFRTSSCQVTTVDSGSKALEFLGLHEHDDENNNTNHHPSVLSNHPQPQEVEVNLVITDYCMPGMTGYDLLKKIKESSYLRNIPVVIMSSENVPSRISRCLEEGAEEFFLKPVRLSDVNKLRPHMMKTKCKKPEIDPRDSQEPSPEHPLIQQECAVEDVKLQPQNPQPQAQTEEQKPVMNSDEDSRKAIEESLSPGRTRTRQEPQGRAEEQPSNENKRKAMEERVSPDRTRPRYNNNGLTHCCL

>ItfRR3

MVIGKPEKVAAGDDCCSGGVQELHVLAVDDSHVDRKVIERLLKISACKVTAVESGSRALQYLGLDGEKGSAAIDGLKVNLIMTDYSMPGMTGYELLKKIKGSSALREIPVVIMSSENILARIDRCLEEGAEEFLMKPVKLSDVKRLKDFVLRGDGESKEGATTTRKRKPTDDSFTRPPLSLSLASSSPSIHPEPTPLSPRCSSVPLSKHPRLHQDTEPLVDP

>ItfRR4

MARNGVFSRRRTAAEMEDSDEVVLSEESHDVHVLAVDDSLVDRKVIEKLLKITACKVTTVDSGRRALQILGLDEEKTSVQFDGLKVDLIITDYCMPGMTGYELLKKIKGSSFREIPVVIMSSENVLARIDRCLEEGAEDFLLKPVKLSDVKRLKSYMFGDDRFHGEDGGTNKPPETPEISDDTSSSSAPSLSLSPSPTTSMDLSSCLSLSPSPTSIDLSATPSPPSTSSPSSPKTFSSSSSPSTNSSSPPSPVAPASPTRILKRRDGD

>ItfRR5

MARNGVFSRWRRAEGPAGFSLPSESHDVHVLAVDDSLVDRKVIEKLLKITSCKVTAVDSGSRALQFLGLDREESSVGFDGLKVDMIITDYCMPGMTGYELLKKIKGSSFREIPVVIMSSENVLARIDRCLEEGAEDFLLKPVKLSDLKRLKSHMFGEDDKNPREDSGINKRKLQEMSEDSSPPLPSPSPLLSPNPSTDLSSSSSSSSSPPSTSSSPSSPELLESPKTEE

>ItfRR6

MAAAVAAATHASESRFHVLAVDDNLVDRKLIERLLTTCSYQVTVVDSGNKALEILGLLEDSVTALSSDHHEVEVDLIITDYWMPGMTGYDLLRKVKECRRDIPVVIMSSENEASRINMCLEEGAQEFLVKPVRQSDVTNLIKPRPFVKGGDDNGVVSPVFCSGVDDNRHVTATETVISPADR

>ItfRR7

MSALFGSYFPRFIHTFSKIISSPNLLQNHFISSLPNPSTTRIYSSVSSSLLFTNTLSLFPLFSPLFLCSTKMATSSCSDLGKFHVLAVDDSIIDRKLIERLLRTCSYQVTVVDSGVKAMEFLGGRIEEVNLIITDYSMPGMTGYELLRKVKGCSSLKDIPVVIMSSEDVPARIDRCLEEGAEEFFLKPVQQADVNRLSSHLLRPKSPEPLPCRKRKAAPAEAQPTRPIRRRRLI

>ItfRR8

MGMAAVEPQFHVLAVDDSLIDRKLIERLFKTSSCQVTTVDSGSKALQFLGLNEDDQKSPIQPSVSPNNHQEVQVNLIITDYCMPGMTGYDLLKKIKESSSLRNIPVVIMSSENVPSRISRCLEEGAEDFFLKPVRLSDVNKLRPHMVKNKKAGEQEIQESSSSEESSAESGMTDVQSQAESNDNRCNKRKALDEGFAPKRTRTRCNSLTAFSDL

>ItfRR9

MGMAAADPQFHVLAVDDSLLDRKLIERLFRTSSCQVTAVDSGSKALEFLGLLEHDQDCPTQPSVLPNHNQEVEVNLIITDYCMPGMTGYDLLKKIKESSSLRNIPVVIMSSENVPSRINRCLEEGAEEFFLKPVRLSDVDKLKPHMMKTKGKKAGSDDTQEHKETSSEESSSVESGVTDVQSQLPQLPLEQPQSETQQHQPLPPDNNNNCNNKRKAMEEGLSPDRSRTRYNGLTSL

>ItfRR10

MGMAAADPQFHVLAVDDSLLDRKLIERLFRTSSCQVTAVDSGSKALEFLGLLEHDQDCPTQPSVLPNHNQEVEVNLIITDYCMPGMTGYDLLKKIKESSSLRNIPVVIMSSENVPSRINRCLEEGAEEFFLKPVRLSDVDKLKPHMMKTKGKKAGSDDTQEHKETSSEESSSVESGVTDVQSQLPQLPLEQPQSETQQHQPLPPDNNNNCNNKRKAMEEGLSPDRSRTRYNGLTSLMALLSSSSSSCSSSMAEAEDEMPHVLAVDDSSVDRKLIERLLATSSCKVTTAENGQRALEFLGLGEGHTSNRQSKVNLIITDYCMPGMTGYDLLKRVKGSSDLKEIPVVIVSSENVPTRIKKCLEGGAQEFMIKPLKQSDVKKLRCHMAKFKQPCSGRLCIGR

>ItfRR11

MSAVMHVLAVDDSTVDRTIVEQLFKAASCKVTTAENGLRALEFLGLLAGDDQNNSPNTNVPKLNLIITDYSMPEMNGYEFLKKVKGSAMFKDVPVVVMSSEDTPSQINQCMEAGASVFILKPLKQADVNQLKSQLMQA

>ItfRR12

MATSSRNGGDESPHVLAVDDNLVDRKLVEKLLKNSSCRVTTAENGLRALEYLGLGDEQHNTSNDNESSNMKDIPVVIMSSENIPTRINQCLEEGAQMFMLKPLKHADVKRLRGELMQCRG

>ItfRR13

MACPSSSMAMGENGEDEVIHVLAVDDDPVNLIILEKLLNSSSCKVTTAENGLRALEYLGILAGDDQQNSPNTNESAMLKDVPVVVMSSENVPSIINQCLEEGALMFMPKPLNQSDVKHLISQL

>ItfRR14

MNKLFSNPLFPAGVRVLLVDDDATCLRILEALLLACNYKVVKCRGAIDALRILQEGKEEIDIVLSELHMSRVNGFKLLDQIIGLQIDLPVVMMSSDDTVDAIKKIVIQGACGYLLKPVRKEEIKVLWQHVVRHKQGNLGKGIRPPQAAAFWDSGEMPRQQKSVENCGSSNNNRDEDTNATATTANVKKPRLVWTPQLHQQFVAVVSQIGLRNAVPKKILDLMNVPNLSRENVASHLQKYRLHLQRNGDQNSYKRFSIHQLHYEDMVLNKNNEQQVMPAGGDHNYVNVYDYGSLGRGSNVYNYGGLGRRVGGSNVYNYGSLGRRVGGSTTTMDNYGSLGRRVGGSTTTMDNYGSLGRRVEGSTTTNYFSAFPPYQPQSTGYTYNNNNPNQLGNTSSAPSQRQQDETQYNGEGDFGVSLYDFSGTDSNYT

>ItfRR15

MESVMAGGIFLPRSETFPAGLRVLVVDDDPTWLKILEKMLKKCSYEVTTCGLATEAISLLRERRNGFDIVISDVNMPDMDGFKLLELVGLEMDLPVIMMSVDGETSRVMKGVQHGACDYLLKPIRMKELRNIWQHVVRKRMQESRDIENHEGDQFDEAWMFNGIELQSGKKRKDFDYRFDERETSDSKSGDPSSVKKPRVVWTVDLHQKFVKAVNHIGFDKVGPKKILDLMGVPWLTRENVASHLQKYRLYLTRLQKENELKASSSGTKHPDLSPKESSSSACLQNLVDVKPSKSTNGKYAFHGEKFCVQEVESRNYEGEVKAAAPLSTAGVSRALVGENCDSQKSISCSKASWASEVSKTGFKHEFKPQIQTEDNINHLPSPKLPRNVHLDQAQPLLNLAPHQDINPGEIKSKPSNINTENPGVRTVSPLECAVDLLPAQPSQPQSCLTNFQAFEQIPSTTWSAKTPQILINGLESVEGNLFLGGGSWDKDFNAAALQGEFHSPCVVGPQSLELLDYSNTNLTGEIQPYFYDYEYAIDPVIDHGLFIL

>ItfRR16

MNLGGGQVGKGMSATCSNASWKSGDAVSDKFPAGLRVLVVDDDPTCLKILEKMLRTCLYEVTKCNRAELALSYLRENKNGFDIVISDVHMPDMDGFKLLEHVGLEMDLPVIMMSADDSKNVVMKGVTHGACDYLIKPVRIEALKNIWQHVVRKRKHEWKDKDPEQSGSADEGDRPQKPSDDADYSSSVNEGNWKNSKKRKDEEDEAEERDDTSTLKKPRVVWSVELHQQFVAAVNQLGIDKAVPKKILELMNVPGLTRENVASHLQKYRLYLRRLSGQGGLGNSFMGHPESPFGSMSSLNGLDLQALAASGQISAQSLATFQAAALGSSVTKSAISMPLVDQRNLFSFENPKSRFGDGPPQLGNSSKQIGLLHGIPTTMEPKQLASLHQSSPTFGGMSMQLNSQVHQNNPLLMQMAQPQPRAQMVNDPNGSQASRLPLSVPQPILSSAMAGGVLGGNSIVDNSCSAIHSSVSHAPSTVAFSVNQGTELQTNSYTTSNSGVSSLTSRGMLREQANPDVKGSRGFVPSYDIFNDLHQHKAQDWGLQNVGSTFDPPPHHSNLQGILDPPPSVMAQHGFSSNQKSGQNRNAPINKDVFLSGEQTGHGNNPMLGPQFNSLLGGNPVTIKTERLPDTSFQNTLFSDQYGQEDLMSALLKQQQDSLGPVENEFGFDGYQLDNLPVLNPTMLIHSRTNIALEPK

>ItfRR17

MSVHSSVASWKPVDGVSDQFPVGLRVLVVDDDPTCLRILEKMLRNCRYEVTTCNMAEVALSMLRENKNGFDIVLSDVHMPDMDGFKLLECIGLEMDLPVIMMSADDSKNVVMKGVTHGAYDYLIKPVRIEALKNIWQHVVRKRKQELKDKDVEQSGSVEEGDRQQKPSEDIDYSSSANEGNWKCLKKRKDEEDEGEERDETSALKKPRVVWSVELHQQFVAAVNQLGIDKAVPKKILELMNVPGLTRENVASHLQKYRLYLRRLSGVSQHQSGLNSSFMGPPDTTFGAMSSLNGLDFQTLAATGQISAQSLASLQAAALGRSATKPAISMPLVDQRNLFSFENPKFRFVEGQQPLNNNSKQIGLLHGIPTTMEPKQLASLNQSSQTFRGRGMQPPVHQNNSLLMQQMGPPQSQAHMLNEPNGTQVSRVTQPILSNGMPSELLARNGIVDNSRGAIYQPVSQAQPLVDFSVNQNTEMQGNSFISGNSGMSCLASKRMMIQEGVNSDVKRPGGGFAPPSYDIFNDLQQHKAQDDWGMGAVFEASRLPNAQGTLDASQSVMVQQGFSSSQNSAQNGRVSIGKAVFPSGQESGNPMVGPQLNSLLGGNSITIKAERLPDASYQNTLFPDQHGQDDLMSALLKQQESVGPVENEFSFDGFQLGNLPV

>ItfRR18

MAAVCKAEAAAVVPEQFPVGLRVLVVDDDLLCLRIIEQMLRKCKYNVTICSQATAALNLLRERRGCFDIVISDVHMPDMDGFKLLEHVGLEMDLPVIMMSADGRTNLVMRGIRHGACDYLIKPIRDEELKNIWQHVVRKKCNLSKENDHSGSFEDNDQPKQGGDDAEHASSVIEGADGVLKTVKKKRDFKDDDDDDDDDEIENDDPANAKKPRVVWSVELHQQFVSAVNQLGIDKAVPKRILELMNVPGLTRENVASHLQKFRLYLKRLSGVAQQQGGLPNSFCGPIEPNPKLGSLGRYEIQALAASCQIAPQTLAAIHAELLGRPTSGLVLPTIDHPALLQASLPGTKYILDDQAVAYGQPLMKCPPNISKQFTQHLSAEDIPSGVGAWPPKNVCVVPSINLSGLGAQNGNMLTTMMQHHQQQQKQQQMEQHQKLSTIPESCRPVNVRPSCLVVPSQSSANFQVTNSPASISQTSSFSKSNVMDSRILSPQSGNSSSGAGEVANWEQKLPCRSNMFCATGSLSPSLSSCSTNADNSASWQVQNSACIIGASRHAAGVVPNITGIPVPDNHKSNQLLDQGPIRNLGFSSRGSSIPSRFAIDESESPPISNIYHSRIYKESNTCKVKQEPDVNIADNAKVSVQTLQRIPPNDFMSVFQ

>ItfRR19

MAAKAPLVLLVCSVGCKFTTTNQAIQALQLLRENKNQFDLVISDVDMPDMDGFKLLELVGLEMDLPVIMLSAYGDTNLVMKGITHGACDYLLKPVRIEELKNIWQHVLRRKKFDCEEQKISNKPDGESGELGRGFRGMGETDRNGKPTRKRKDQSDDEDEELDENGGRNEDPSAQKKPRVVWSVELHQKFVAAVKHLGIDKAMPKRILELMNVEKLTRENVASHLQKYRIYLKRLNSVASQHANMVSVLGNADPSYLRMGSLNNIGNIPFITGCTQFSDAPLRSISSGSVLTRLNTPSGLGMCGFAPSSMIQLANAPNSSSSITSEINFRQSIQPGNQDMDILEGMPMPLGTDQVHNNLGVTHLYPFSNGMPERKIDVDGRRNLTIGVSDNSIILRSQGQCVQRKEFLDNQFPVIASPVSSASSPFLNTTRCNDNWPTASQSSLLEANSFGTSVYSHHAMPRDLGNNGSTLEVPMSSNLHNPLNSACPQVPDTRTEMQCLTTIIDNVSGVKMNFSPRQDWHDFEPDSAHVPSLVCSSAHTFLRPDGGQRQQQHHEFENAPVDMKQEYLEEQKMLAGNNAYGQMG

>ItfRR20

MTVDESRRRVEKESNSDNFPVGMRVLAVDDDPICLKLLECLLRKCQYHVTVTNQATTALEMLRENKDRFDLVISDVYMPDMDGFKLLELVGLEMDLPVIMLSANSDTSLVMKGVTHGACDYLVKPVRIEELRNIWQHVIRRKTFDSKHHSKSGDQDNEEEGRQGDQLSGTAEQNEKLNKRRKDEEDGSENEDPATQKKPRVVWSAELHRKFVAAVNHLGIEKAVPKRILDMMNVEGLSRENVASHLQKYRLYLKRISLVSTPQANMATPYMPMGSLGGFGDLQTLAGPGQLNRATLSPYVPGSLLGRLNSSAGVSLQNRNLLGLLQPSHAQSSGNSLDLLGKLNSNAPPTSQNPSLFRGIPSLELDQLQHGKFPKIEQVLNPMDNSKLLTSASTFTGSGSAFGNPINATILQGNSQQGQTGEGFGNPHSLNMASLRPEPLNTGVSSASNFLGHGGLNGNLGNSILASNVQPNCYPLMETFTHSQLDQNHVRGGNYSPAGPHLQSSPLGYNSTFSTSIPYENSRGQTQYQEGFIGDAIQSVNQAPTQFWGDHNSNNVFSNSSSQILGNGLMPPLSQIADQNNDIFNMKTDTPLIGQENGGSVVLFPHNENANFNQDSRMGSNEDYMLNSAKPQGAYSSLDDLMNGVIKGEQNGQFGFDDYLFGS

>ItfRR21

MTVEETMRNMGVDRENYHNFPLGMRVLAVDDDPICLKLLEGLLRKCQYHVTTTSQARMALNMLRENKDRFDLVISDVHMPDMDGFKLLELVGLEMDLPVIMLSANSDTKLVMKGITHGACDYLVKPVRIEELRNIWQHVIRRKKSESKGQNQDNGYRGNGEGGQGFPLTGSAEQNALLNKKRKDEEDETNENEDPSSQKKPRVVWSIELHRKFVAAVNQLGIEKAVPKRILELMNVEGLTRENVASHLQKYRLYLKRISSVATQQANMVAALRGKDSAFMRMASLDGLGDFQALGGPGRFNHATLSTYTPADMLGRLSSATGVSIRNLSASALVQSNHAQNLENSLGSDGNLNPNISLSSHNAATLFQGIPSPLAVQELNPLENSRALTAASAFADSGSVIGSSTNPMMLQGSPKQGLIGGGFGNQHSLNMASLSSELYNTGVNSSSNFLGHGRSSENWQTSIQVSEFQSGSYPLPEPFSHSQLPQNCEREHESSAATHLHSSPVGFSSTTSASTTFDDSREPQVPSQLWGDGKQNQNSNDIFRNLSSHVPPSLSQGMYQTSGNLNTKMNSFLMRRSNAGSLVLFHQNGNEMPTTEPRTRSNEDNLLESTKTHGAFVSQGFDTLDDLMNAVIKQEQDGGILVGEFGFDAYPFGSCM

>ItfRR22

MALKMLRENRERFDLVISDVHMPDMDGFKLLELVGLEMDLPVIMLSANSDTKLVMKGITHGACDYLVKPVRIEELRNIWQHVIRRKKFDSKSQNKSGDQDRSPHGGGEGGQGGPLSGSTDQNGKLNKKRKDEEDESDENGHENEDPATQKKPRVVWSIELHRKFVAAVNQLGIEKAVPKRILDLMNVDGLTRENVASHLQKYRLYLKRISSVATQQANMVAAFGGKDSAYMRMGSLDGLGDFRTLAGSGRFSHASLSSYTPGGMLGRLNSAAGVSIRNLTSPSLIQPSHGQNLGKPLGTLGKLTPNVPAVSQNACLFQGIPSSLELDQFQQSEGTPHIRGDLNPLDDSTLLGAANTFTDPGSGIGSSSNPMMLHGNSQQGLMAGGIGNQHSLNMASLNSEHFNIGVGGSSNFLDHGRSSDNWQNPIQVTNFQSSSLPLTETFNQGQMQQNCARENNSSIGPHLLGGCAGYSSLASTTTPFEDSRGEIQRRERLVGDAIPSINQVPSQQWGELKQNPNSNGVYSNLTAQVPASSIVPPLSQSMDQCNDTGNRRIDASGQSNLSSSVLLQHNKNEKLTSESRARYYEDYLFEPPKPQGAFPSQGYGSLDDLMSSVIKREQDGATLEGEFGFDAYSFGPCI

>ItfRR23

MASLKVLVVDDDSTCLAIVAALLVKMEFQVVALKNGNDAIEALRTQGGFDVVISDVHMPGMNGFELQQLIVKQFRIPVVLMSGDCEEGIVRQAMQNGAISFIRKPVSPNDLRGIWQYVIAQKRSKTTIEQVNTDDQDNIDLGTSSSGIRSYSGYDTTSKKKMVWTDQAHFIFLDAISSLGPENATPKNILKAMNVPGLTRENVGSHLQKYRQFLRRNIQDIENRNNGGGGNQPRFGSRKFRRLAKLEEMFAQGGLGAALSSPTSSAALGRSSAGGDARTRGGRGGSSERGQQGVILSEDDELMFCRGLRRGPIITSPNESSPASHEVNPGGSQESGPYPTLTALMQNRLGSSSNDNANVSGFGNVNVVSGNEDVQVPGEGNFDIYDAILNFIGETEDGGDENISMYGNITAGQHNSGYNQIGNLSNDFLNQGDGDDDDDAFVNSLYGPNPYGGAAGNGEGM

>ItfRR24

MGSKNVGKGLRVLVVEDDPATQMVHKMLLKKCGLEAQMAKNGEEAVMLHRFGARFNLLLMDKEMPVKDGVNATRELWEMGLESTYRSRAVRDEFIVAGLDECLMKLLGPEVILRLINQL

>ItfRR25

MGSKNVGKGLRVLVVVDDPATQMVHKMLLKKCGLEAQMAKNGVEAVMLHRFGARFNLLLMDKEMPIKDDVNATRELWEMGLESTYGSRAVRDEFIVAGLDECLMKLLDPEVISRLINQL

>ItfRR26

MDMGSKNVGKGLRALVVEDDPATQMVHKMLLKKYGVEAQVAKNGEEAVELHRSGARFDLLLMDKDMPVKDGVNATRELRELGVKNMIVGVTSHGPGAVRDEFMAAGLDECLMKPLGADMVLRLINQLVAKNGDKAMVLHHFDLLLMEKEMPVKDGVNATWELREMGLKSMIVGVTLHGPGEVRDEFMVAGLDECLMKPLGSDVVLRLINQLVA

>ItfRR27

MDMGSKNVGKGLRALVVEDNPATQMVHKMLLKKYGVEAQVAKNGEEAVELHHSGARFDLLLMDKDMPVKDGVNATRELRELGVKSMIVGVTSHGPGAVRDEFMAAGLDECLMKPLGADMVLRLINKLVA

>ItfRR28

MDMGSKNVWKGLRALVVVDNPATQMVHKMLLKKYVLEAQVAKNGEEAVVLHCFGACFDLLLMDKEMPVKDGVNATRELREIGVKSMIVGVTSHGPGSIRDEFIAVGLDECLMKQRGPEVILGLINSLTSWLAKNVLL

>ItfRR29

MDIGSKNVWKGLRALVVVDNPATQMVHKMLLKKYVLEAQVAKNGEEAIVLHCFGARFDVLLMDKEMPVKDGVNATWELCEMGLKSMIVGVTSHGSGAVRDEFIAAGLDECPDEVAGFRSDFAPD

>ItfPRR1

MKLTIEVYQDALQFGRKMKKRNTHKISKSSNEPRSEIEEWHYNSDCQVADEGSASPAIAANEGSTSPAFQLFSVFPLCAHRRMAQLIELSIGEIISHLELREDPEQGIVVAGLRCIKDDSVIVVLFLIEAANGLQAWRVLEDLTNHIDLVLTDLEMPCVSGIGLLCKIMSHKTRKNVPVALSATVMTTFMAPPPAATSLAFHPQDNNVIAIGMDDSSIQIYNVRVDEVKSKLKGHQKRVTGLAFSNVLNVLVSAGADAQLCVWSMDGWEKKASKFLPNPSSRAPNPVTQTHVQFHQDQIHVLVAHESQIAIYEALPIKMCYGGGFGANIWKNNPRKREDIYHTSHRVEFENAYCGVNPKGELDESAGYVLSRCIFVV

>ItfPRR2

MMEKNEIVKTGDGFIDRSKVRILLCDNDSKSSEEVFTLLCKCSYQVTSVRSPRQVIDALNAEGPDIDIILSEVDLPMSKGLKLLKYIMRDKELRRIPVIMMSSQDEVSVVVKCLKLGAADYLVKPLRTNELLNLWTHMWRRRRMLGLAEKNILNYEFDLVVSDPSDANTNSTTLFSDDTDEKSRKSINLETGPSTQQEDETNAITNAASPETLVIGSFECLPDVPGSSDRKTGKICSFPKKSELKIGESSAFFTYVKSSMPKSNDQVTVRENVTYHSRINEGGNVDIESKERANGDAIENHSQGDGYPSSNSIPDSLSMERSCTPPLSMEFPQQRMEEFSKVHMHPTNESHHDISGYHAHAHAAYPPYYIPRIMNQVMMPSSQMYQKNLPDLHNHANSAMLPTYSHVPHCPPHMPGMGSFPYYPMNMCLQPGQMPPQHPWPSYGSSSSADGKMGKIDHREAALMKFRQKRKARCFDKKIRYVNRKRLADRRPRVRGQFVRKPNGVLVDLNGHPASADDDEEDDEDEDDEDQTTTLDSSPEDDTSISLL

>ItfPRR3

MGKGKGKSIMVSGGDGGNATDLPDCSRVRVLLCDTNADSCRHVFQLLTQCSYQVALVTSRAQLFDTLRSEGPCMDIILAEIAILIANESSIMRYIKRDVRLKHVPVIMMVTIEEVSLIRKGLGFGAADYLVKPLSIHEIKDLGFHIKKN

>ItfPRR4

MKRKDLEMEIHAKEEMHVTGDDDKELTVNSASEFWEMFLHVTSIRVLVVENDDSTRRVISALLMNCNYEVIGASNGLEAWKILEDETNQIDLVLSEVVIPYLSGLDLLCKIRSHKPRSNIPVIMMSSHDSMSLVFKCLSNGAVDFLVKPVRKNELKNLWQHVWRSSHNSNGSVSGSGKETKISDDDGEDSRSSSDLKTGGGNE

>ItfPRR5

MTTISEGEKDLPDEDRKVEDGIVCEGQNASADVELKVESVSKDVNDEGRRALQAQGAIQVQQQQSQSGTICWERFLHVTSIKVLLVESDDSTRHIVTALLRNCNYEVIEAANVLQAWRVLEDLTNHIDLVLTELEMPCVSGIVLLCKIMSHKTRKNVPVIMMSSRDSMGLVFKCLSKGAVDFLVKPIRKNELKNLWQHVWRRCHSSSGSGSESGTQTQNSVKSKSIEKCGNNSGSSDGEDNGSDGLNIGDGSDDGSGAQSSWTKQAAEVDSSQAASPWDQVTECPDSTCAQVIRSNAENSGNRKVHVAATKDCQEEKQPDNTKCKYPAMTIPKKLETQCENPIGAPINSVGEKHTNMVEIDPSASNKRIEKEQIDRKEFEAQKMVAAVSEIENNTIHESRKAVIEPSLKRLREMKESRETSEDDRCVFRRSEQSAFTRYNTSSQSNPLRTPNGLTGNSLVIDSGLESANNVVSNNIDMGSTTNKLATKPLTVQDKSEATCTTNGLHPSSAYKPVKNDFRNCQSLIKTSDMQATTLLAPSSSHTDIPDQHLHHHNNHYPRNSHHFHNQEQQPASNHDKFSLKQLTANALNSDSSNVMAGPFEGTLGNHSLNKSASGSNHGSNGQNGSSTAVNVGGNNGKTETGLDGKGGSGSGDASGSGSGSRMDPNKLAQREAALSKFRQKKKSRCFKNKVRYQNRKRLAEQRPRIRGQFVRQTGQNNPSNTENE

>ItfPRR6

MRGVRVDGNGPPLKGLTEINHNGMRSEQNGVRDGVNGDGHGLSEEDESRINEDAEDRNDMRRDLMQVQAVLHTQQQQPQGPVVRWERFLPLRSLKVLLVENDDSTRHVVSALLRNCSYEVTAVANGVEAWKILEDLTNHIDLVLTEVAMPYMSGIGLLSKVMNHKTRKNVPLIMMSSNDSMGVVFKCLSKGAVDFLVKPIRKNELKNLWQHVWRKCHSSSGSGSESGIRTEKSTKSNKSIEGSENNSDSNDEGENGSIGLNTRDGSDNGSGTQSSWSKRAIEVESPQPMLPWNELPEPPDSTCAQVIHSRPEAQSANWVPTIATREYQDEEDDQENVPMGKDLQIGVPRSPDLQLNGPTSKALDGDASAKKGKLVNVDSSKDDEKLIGKLELNKTRKNELKDKDNGHVAAITIKDNDLMEITGNDVPTDPSKMTNTKEIATYNSKEMPSLELSLKQHREVGETGTTVQERNVLRHSDHLSAFSRYGTTSTANQAPTGNVGSCSPVNNSSEAAKTESLQNLRSNSSSMPNQRSNGSSNNNDMGSSTNNIFVKAEAFTDKPVNKSSAVNAHPCSAFQPVQHGQNSSLPGKADSAKSALAQARAMQQQFQVQHHHHHYHHHHHHVHSMQQQQQQQQQQQQQQQLLNEDSLPSRKTVADAPHGSGPYMLGTLTDGNTNYGSASGSNNASNGHNGSSGQNESNTAVIAEETNMATEDGIAGKCTVGGESGSGSRSGVDQCRQAQREAALNKFRQKRKERNFEKKVRYQSRKRLAEQRPRIRGQFVSQSSDKTKTKDTNC

>ItfPRR7

MGETAANGGDGGAGTESTVQETEQIEVIEPKQGKNDGAEPPQAAMRWERFLPKMVLRVLLVEADDSTRQIIAALLRKCSYRVAAVPDGLKAWEVLKERPSNVDLILTEVELPSISGYALLTLIMEHEICKNIPVIMMSSNDSVSMVYKCMLRGAADFLVKPVRKNELRNLWQHVWRRQAANRIDNLNGPVSPTRNEDCNEKGSDDENSCVKLEMEIGGENTEHVEESEQRFRGNSLPIHPEKQEQEDHNQVGDDDDDNVNDDPPYLKSSKQAIDLIGAFDNSPKCDYRSSCSKDSADKVESLPPLDLSLTRYPSGSMNRLNHSDASAFTRYINKGVQPRNSMAPETCNQHEDCGTDSDKHLSVHNLDGPTMKFHTLMQQARAEPGNNEIGLPIPVRGVGFKGLGNAHSSAGCPDSQFQTPLFRLLNHQAGTYQQSCGLVDQNTDNDTSQSEKKEENTDDHGHFSPFTDQSANSRTSVPLINSTAECSKEKAPLGQDGSYQQSQREAALTKFRLKRKDRCFEKKVRYESRKKLAEQRPRVKGQFVRQQPK

>ItfPRR8

MGEVVVSGDGGAAAMELETEEVEVVEAPASAAAASAVRWERFLPKMVLRVLLVEADDSTRQIIAALLRKCSYKVAAVPDGLKAWEVLKGRPRNVDLILTEVDLPSISGYALLTLIMEHEICKNIPVIMMSAHDSVSTVYRCMLRGAADFLVKPVRKNELRNLWQHVWRRQATSKSGQGPGDESVAQLKVEATAENNGFSNHSSGYKACIERNRECIEKGSDAQSSCTKPEMETGEENTKHIQEFGQPDWNKPRPADADMQKEEQHRDGSTKLRNPSDQAEGTGYNAATVASGEDRSSNENCCHLQVIGQASDEDPAIMNSCKRAIDLIGTFDNHGICTYISGSNISANNKVDSPPPLELSLTRYPSGSVNQFPDEKHKLNHSDASAFTRYVSKGVQPRDLISPKNKESETDSDKRLSVHNLDCNSDTHGPTASSQRLVPPTNFESGRAETELPSPGQRVLSAPIPVRGVRFEGLSNAYSFMTSPMQSPGSAGHQNSPRQANTFHRLNHQTINSQQRHSVIEQNVNTVSTQTEYKQGYQSEPDRGHFSSATDQSPNSSLCNGVVNCHYTGGGSNGRIPVTMIKSTAEYRNDEASVVQDANSQRSQREAALNKFRLKRKDRCYEKKVRYESRKKLAEQRPRVKGQFVRQLPSEPPPGDT

>ItfPRR9

MEELRKDTNTFFEAISLFLGERTYSSTSTRFLEGMELNEAAEAERTKDHESGGGGDGGGSAAVFRWERFLAKMAVRVLLVEADDSTRHIISALLRKCGYKVAAVCDGLKAWEVLKKKPHNVDLILAEVDLPSISGYALLTLIMEHQICKNIPVIMMSSQDSVSTAYGCMLRGAADFLVKPIRKNELTNLWQHVWRRQALSSGAMVENNDISNGECREKGSEDQSCCSKPDIDTEREMTEDIQDLLQPNWDRSLPIVDQASDEDIESCKQGIDLIGAFDDYLNCNHINPSSNASPNKQVDSAAPELDLSLTRTHPTSMLNQFVDNHRLNHSDGSAFTPYVNKGMQKQRGLTIPIGVRFEGASSPVISPSDSPASGNLDSPPGLDPNPVHPIPSTAECSRKEEVSLAQDGNSQRSSQREAALTKFRLKRKDRCFEKKVRYESRKKLAEQRPRVKGQFVRRQPIGTGDNEMISAVNQ

>ItfPRR10

MWRLPEYHIDNSYVNISMASYHRVTYGIHVLLADHDHKFVASTVDMLKRQFYKVTVVDSANAAISILNRKEEKFDAVIANIHSPDRQAYRLLREAVSMDLLVIFLCDEEDAEMAVRVIEHGAFALLQKPTCQETLKNLWQHVVRERSMLRAKQMIFMEKTNRELAVINNAVINNGVVGGGSDRGKGVMRVEENENYQMSYRGKGKRSREQSLSEATRMTTTMSQGMSRVKRKTCTEWTVDLHEKFMSAVHQLGDGRCYPKEILELMNVPGLTRMQVASHLQKCRNDNWRAPEERRAPPMSSASPASGSGSRNEQRRFGTMPKLTAAAAAAAAAAGGNSQQLGSTMSPEVQSSPSITGAVTGDPSSQPPADRQYLAIGAAAAFVSKLESSSPPSAAVQPAVVATAVGTFTATSGQASIVGSVNYGPGGILQALGCGGSGGSLFRNKDAFTDDPNNNTTAGDDSFVTPQPPRIHRRLQSADEFFSFNDVDYEYLIQGFSDNNARQAGVALQAPTHNNTSSSEFNDKAVTVVDSANAAISVLNRKEEKFDAVIANIHSPDRQAYRLLREAVSMDLLVIFLCDEEDAEVAVRVIEHGAFALLQKPTCQETLKNLWQHVVRERSMLRAKQMIFMEKTNRELAVINNGVVGGGGDRGKGVMRVEENQNYEMSYRGKGKRSREQSLSEATRMTTTMTQGMSRVKRKTCTEWTVDLHEKFMSAVHQLGDGRCYPKEILELMNVPGLTRMQVASHLQKCRNDNWRAPEERRAPPMSSASPASGSGSRNEQRRFGTMPKLTAAAAAAAAAAGGNSQQLGSIASPEVQSSPSITGAVTGDPSSQPPADRQYLAIGAAAAFVSKLESSSPPSAAVQPAVVASAVGTFTATSSQASIVGSVNYGPGGILQALGCGGSGGSLFRNKMLSLMTPTTTPPPVTTLSSLPSRPASTAGFSRPMNSSASTT

>ItfPRR11

MAAFPSFPHNFRLVDMGEFAGFRHSFPQVTDEANSSIGSLVFPPRRMHGVHVLLVERRADFVANGTSMVAEILKQFSYEVTVVESASAALLSLYHGKEKFDVLIANFYLPDKEVNVKLLEEAIKRKLLVVQISDEKDENGDEVARRAIEQGVFLYLEKPFPVDMLKYLWQHVYRERRLMNHSTHQALDISMVAETLMNGQNNIVFTDNQTATAFATDSDNVNLVPTRRRGAKFKWTEELHAKFMHAVNQLGAGNCYPKEISEMMNVPGLTREKIASHLQRCRDNKWRPVEEHGNRRRSRAMQSTSQPRRPRHKKFGLMPTVEELEANNNGIMPPQEQIVAAAPVANNEISSQNGGNNNNYEYRSLMTVCTNSVTYIDTGILQAVQSSACIIQDRRLLGEVQTHPIVADSVTNTDVGVFQTGQSSAGVLEIGQSSAGTSHDPYRPQLEEVQTHPVVTDSLNIDETAFVNSSPIPWQFDDGLMFDDLLNIPEGMLPRIFFGGQLNFPF

>ItfPRR12

MLCTANDLLGWKDFPKGLSVLLLDEDSNSAAKMRTKLEEMDYIVSTFQNKNEALLAIASKSVEFHVAIVEVNSSNSNEVFKFLETAKDLPTILVSNVYCLNTMMKCIALGAVEFLQKPLSDDKLRNIWQHVVHKAFNAGGGKDVESLKPVKESLVSMLQLRSTKNEANTANSDETEQSTSVQENNRDTLSSVCDKYPAPSTPTLKQCVRSLDDGECRDQTNLSMEHDSVEHDGESKSVETTYCNSVSETIPVINPPVIKQERESSPEQAGKNGNSACSESKDARANANSECGEPKKPSGVNSSTGTKANKKKVKVDWTPELHKKFVQAVEQLGLDQAIPSRILEVMKVEGLTRHNIASHLQKYRMHRRQILPKENEWKWPLSRDSTQRSCYPRKPVMAFPQYHSAPTVPAGHQFYSAWAHSGSYPGAHVWGSPYHYPGWQPTDDWNWQSNTGVYAQAWGCPVMPAPQGSYPTYPQNTSGHHRSGGAQDRYSMLDNTYDIQPAEELIDKVVKEAINNPWLPLPLGLKPPSTDSVLNELSKQGISTIPPRTNGSDLR

>ItfPRR13

MVCTANDLLDWKDFPKGLRVLLLDEDTNSAAEMRSKLEEMNYIVSTFQKENEALLAISNKSEAFHVAIIEVKTGNSDEAFKFLETAKDLPTIMTSNIHCLNTMMKCIALGAVEFLQKPLSDDKLRNIWQHVVHKAFNAGEKDVSESLKPVKESIVSMLQFESRNSGADAQNSNETIRENSQEFSSDSDKYPAPSTPQLKQGARSLDDCECLDQTNFLMEHDSVERDEESKSVETTCCNSVSSTNPAISPPVSLVEASIKGDCKSSLDHKSRTENSTSLQSTDAPLNVSNESAAPNKLSRVNSSSGTKVNKKKLKVDWTPELHKKFVQAVEQLGVDAAIPSRILEVMKVEGLTRHNVASHLQKYRMHRRQILPKDDAKRWPNPRDSTQRSCFPRDPILAFPPYHSPYSIPSDQYYPAWVQPGSYPSGVQMWGSPYHYPGWQSTDNWHWKPHPGAHANAWGCPVMPSPQGSYPTYPQNASGYYRADGVQNRYHMLEKSFDFQPAEEVIDKVVKEAINKPWLPLPLGLKPPSTECVLNELLKQGISTIPHKINGSHTR

>ItfPRR14

MHFVIDEHKLYATLVSNLDNSSIAPSTQKQLRRARDLPLLLAKAIHSSSSRFSEMEISKRVCLFVFNEDYICKNLVSEVLQHCSYEVLHIGRAMDALSEIGKRKNGISGVLTNMNRLKTKGAEIIQAIQEKLNLRVCLILPGNMEFNDTRGQDCNVSAYIVDLSDMKDMKELWQSAFEKEKARKAAISSPVVGVETGEPGNDHHNRKAKELREKQNEESGGETKKKPRLNWNPEMHQRFVEAVNKLGYDKAVPKKIVEFMNEPGLTREHVASHLQKYRMNLRKGQDSSRDFIYGHQKLTNDVTNPFFGSCLSALKFNSSHTYSSFPFERNNSVFSTPLLAQYSYLLNPNISTTLTQQPHMFPTNNLNHIFPTHQQASFPAAFCSSPYTENNSLLQGISGQGINTNDHPTSIVSLPPLLPGNSENLSSKQIESSSVIEINSYGGEEDISALLDAADNDTPNNNPEEGLWDDDDDFSDILSGFTK

>ItfPRR15

MGISKRVCLFVFNEDYICQNLVSEVLQHCSYEVLHIGRAMDALTEIGKRKHGISVVLTNMNRLKTKGAEIIQAIQEELNLRVCLILPGTMEFNDTRGLDCNVSAYIVNFSDTNDMKELWQSAFEKEKARKAAITTDNNEPGNDHHNRKAKELREELSEESGSETRKKPRLSWNPEMHQRFVEAVNKLGFDKAVPKKIVEFMNEPGLTREHVASHLQVCSVIL
