# Supplementary material for: Genome-wide identification and expression analysis of two-component system genes in sweet potato (Ipomoea batatas L.)
Source: Front Plant Sci. 2023 Jan 12;13:1091620. doi: 10.3389/fpls.2022.1091620 (PMC9878860; doi:10.3389/fpls.2022.1091620)
Supplement: Supplementary file 2 [file DataSheet_2.zip › Supplementary Dataset 3. Protein sequences of TCS members in Ipomoea trilobal.docx]

**Protein sequences**

>ItbHK1a

MAVGSNTSPVSSESLSPSITPKGSFLERILCRMFSSGMFCTSNQSPSSQRNFSRDVEEEEFQDASTLCLSSYYSVFVVRLAIMVMLAILIGLLTLLTWHFTRVYTTRSLNTLAFGLRHELLQRPILRMWNILNSTVEIATAQVKLSEYVIRRYSKPVNQAQQAELYEVMRDVTWALFASRKALNSITISYKNGFVQAFHRDHRSNNTFYIYSDLSNYSISGTYDVSMLTSRQGWNDQSIHNNTTAIWYRETLDPLTGVRVGRKSQIPPDELINIAGISQVPDGAATWHVAVSKFSDSPLLSSALPVWDASNESIVAVVGVTTALYSVGQFMKEIVEFHSGHIYLTSQEGWLLATSTSTPLLRNSTTRPELIMAVDSEDPVIKAGAQCLQKEYGNKFPPSNEVHIENAKLGDQMYYIDSFFLNLKRLPMVGVIIIPRKYIMGKVDERAFKTFVILISASICILFIGCVCIFILTNGVSKEMKLRAELIKQLDARRKAEASSNYKSQFLANMSHELRTPMAAVIGLLDILIYDDCLTNEQYATITQIRKCSTALLRLLNNILDISKVESGKLVLEETEFDLTRELEGLIDMFSVQCINHNVETVLDLSDEMPKLVKGDSGRVVQIFANLISNSLKFTTSGYIILRGWCESLNDLTNSRNFFFNQKDSWSVPKVKLKRAERRPFKKDSKTVLWFEVEDTGCGIDPNKWESVFENFEQADPSTTRLHGGTGLGLCIVRSLVNKMGGEIKVVKKNGPGTLMRLYLLLNAPTDGAEQHSHPTLAEQTTTVLLALNGRMGRLIMSKWLEKNGLHTCEAADWNELTQMLQGVFGSKNSVQDSGCERFSDNSSTLLIVVIDIGLLNLSTNIWKEQLNFLDKYSERAKFAWVLYHDTSNSIKSELRKRGHLMMVNRPLYKGKMIQILEAAFTKDKNLELQSAENTAIQVNMHECHHEIDASHSCLTSPDDSDKSETGNVRPVRTFLAEEKPNKHFRNVSSSSIYATLNNYFVDITQPNLGEDDASREDDRREKRNRSEEHSGSTRRVELTTVSSSKTANEQKSLSGLRILLAEDTPVLQRVATIMLEKMGATVVVVGDGQQAVDALKFCKNGPNGSSQEDDTSPTSPTEGFCSPPYDLILMDCQMPKMDGYEATKAIRRSEMETGTHIPIVALTAHAMSSDEAKCLEVGMDAYLTKPIDSKLMVSTILSLTTRKN

>ItbHK4

MGQKIHSQSHHHHTVAMRLGEQLSSKRKYTLICRNRLPQLLGCWILLIFFVSSWIFNNMDATHKEKRKEALVSMCDQRARMLQDQFSVSVNHVHALAILVSTFHYYKSPSAIDKETFAEYTARTAFERPLLSGVAYAERVLNSHRGSFEDQHGWTIRTMDKEPSPIRDEYAPVILAQETVSYLESLDMMSGEEDRENILRARATGKAVLTSPFRLLGSNHLGVVLTFPVYKSMLQANPSQQDRIEATAGYLGGAFDVESLVENLLGQLAGNQAIVVNVYDITNSSDPLVMYGQPGEEGDLSLTHVSKLDFGDPFRKHEMICRYLQKAPTAWAAVTTAFFIFVIGFLVGYMIYGAGIHIIKVKDDCHKMEALKVEAEAADIAKSQFLATVSHEIRTPMNGILGMLALLLDTDLSSTQRDYAQTAQACGKALITLINEVLDRAKIEAGKLELEIVPFDLRSILDDVLSLFSEKSRKKGVELAVFVSDKVPEIVFGDPGRFRQVITNLVGNSVKFTERGHVFVQVSLAEEAKAKSEACLNGGSERFIPSSGYHCETLSGYEVADNRNTWDSFKHVIPDEPLYYRAANKLMTDDASQNVTLMVSVEDTGIGIPLHAQDRVFTPFMQADSSTSRNYGGTGIGLSISKCLVELMGGQINFISRPDVGSTFSFTVNFQRHETNGSVDLKKGLSDDLPMSFKGLRAIVVDGKPVRASVTKYHLKRLGILVEVVNSIKKAVAVLGKNGSLISKGQLQPDMILVEKDVWISEDGGGLNLQIPNLKPNGHTYKVPKMILLAVDISGAEFEKAKAAGFADTIIMKPLRASMVGACLHQVLGMGKKTQGKDACNKSTLRGLLCGKRILVVDDNRVNRRVAAGALKKFGADVECAESGAAALALLQLPHNFDACFMDIQMPEMDGFEATRRIRKMENEANERVNGGLEGEGRHKWHVPILAMTADVIHATLDKCLKIGMDGYVSKPFEEENLYKAVAKFFESKPMPDV

>ItbHK2a

MIPPQHTKITSFGLACLSFLLPLCSTKRMSMNCKVHGMKGGFSSKFRLKKARESQHGPSRWRRQLLFLWLFFVAIGFIWLLISSSYGRLGRKVEAPPHLDGDTTNFLLQHFNVSRDEIHSLASNFLDTDQISLLKCSGSPRYESSVLKSENQVYEKKCKLGEKIEAYGQCPVSDENNFRNIDSVLQETSTPFLSHCASSSISSDHQFCEKETLQVRALGDQCKDIAFCFTKIFWWILLGIAVSWKLRWLRAESGRNEQQKLVSQQEFAQQPQLLEHLQQQQAHVASRVSRKLWEKLLVAFVLSGVIASIWFFWYLNEDIMFWRKETLASMCDERARMLQDQFNVSMNHVHALAILVSTFHHGKQPSAIDQRTFEEYTERTAFERPLTSGVAYALRVLHSERENFERQHGWAIKKMESEDQSLAQEYMPGNLDRAPDKDEYAPVIFSQQTVSHIVSIDMMSGKDDRENILRARASGKGVLTSPFKLLKSNNLGVVLTFAVYNTHLAPDATPDQRINATVGYIGASYDVPSLVEKLLHQLASKHTIVVNVYDTTNTHSPIKMYGADETETELLHVSNLDFGDPARKHEMHCRFKQKPPPPWIAIGASIGVLVITLLVGHIFHAAIARIAKFEHDYQKMMNLKHRAEAADIAKSQFLATVSHEIRTPMNGVLGMLQMLMDTNLDATQRDFAQTAHASGKDLISLINEVLDQAKIDSGHLELEAVSFDLRAVLDNVLSLSSGRSHEKGIELAVYVSDQVPEMVVGDPGRFRQIIANLVGNSIKFTKNKEGHVFVTVHLADEVRCPLDVKDEVLRQSLSLVQDQANRSFNTLSGFPVVDRWRSWQNFKKLSEEESDKIKLLVTVEDTGVGISLEAQGRIFTPFMQADSSTSRTYGGTGIGLSISKHLVDLMGGEIGFFSEPGTGSTFSFTAAFSRDQRGSVEAKWQQYDTGVLDFHGLRALVIDGKRIRAEVTRYHLQRLGLNVKITSTVDHACSYLSTCSKTSEPEHLVIMIFIDKDNWDTENSFALRNIVKDLRPYGSTTLNGATPKLFLLATEMSTTECNQLKSDGLVDNVLIKPIRLSVLASCLQEATGFTYKRQVTMPKPSTLGNLLKEKQILVVDDNIVNRRVAEGALKKYGAIVTCVDGGKAALALLKPPHNFDACFMDLQMPEMDGFEATRQIRKLENEYKETINSGEILADAPGKLAHWRLPILAMTADVIRASNEECMRCGMDDYVSKPFDEGQLYSALARFFESG

>ItbHK2b

MSWNCKTLGMKGSLSSNFRLRKLLSGGWRWRRKYLILWLIFVAIGLIGLLISLNNGLMRRKVDAPDLDEDSTNLLLEHFNVSKEGIQVLSAENVVYQKQYELAIEKLEANGQCPVPDENTLTNLDIVVQQIPLPISHCASLATSSDHQFCEKEPLQGRALGDQCKDAAFYFTKVCWWILLGIAISWKLCWLCGESGGNDWQKQVQQQELPQQPQLLQHLQQQQAQASSRIARKWWEKLLVISVSVGVMGSIWLFSHLNEEFTVRRKETIASMCDERARMLQDQFNVSMNHVHAWAFLVSTFHHGKQPSAIDQKTFEEYADRTAFERPLTSGVAYAIKLCHSERENFEKRQGWTIKKMESEDQSLAQEYISGNLDPAPIQDEYAPVIFSQQTISHIVSIDMMSGKEDRENILRARASGKGVLTSPFKLLKSNNVGVILTFAVFNTDLPPDATPEQRINATLGYFGAAYDFPSLVEKLLHQLASKHTIVVNVYDTTNASAPIRMYGMEEADLDETDRELVHVINLDFGDPARRHEMHCRFKQKRPPPWTAIAASIGVLVITLLLGHIFHAAINRIAKFERDYQKMMDLKHRAEAADIAKSQFLATVSHEIRTPTNGVLGMLQMLMDTNLDATQLEYAQTAHASGKDLISLINEVLDQAKIESGRLELEAVAFDLRAVLDKVLSLCSGRSHEKRIELAVYVSDQVPEVVIGDPGRFRQIITNLVGNSIKFTKEKGHVFVSVHLADEVKSPNDVKDEVLRQSLTLVQDRPNTSFNTLSGFPIVDRWRSWQNFKKLSEEKTENIKLLVTVEDAGVGIPLEAQGRIFMPFMQADSSTSRTYGGTGIGLSISKRLVELMGGEIGFFSEPGTGSTFSFTAAFARAEEGLLESKRQRNDPSVSELRGLRALVIDDKSIRAEVTRYHLQRLGLNVKIISKMDSSCSHLSTCLEASPLEHLALIFIDKDNWDDETSITLSKILKELRANSSNVVSGVIPKFVLLATNMSATNRNELRLAGLVDSILIKPLRLSALVSCIQETMGFMNKRHITRRKPSSLGSLLKDKRILVVDDNVVNRRVAEGAIRKYGAIVSCVDSGKAALALLKPPHKFDACFMDLQMPEMDGFEATRQIRCLESKYNENINSGEVLIEMHGKVSHWHTPILATTADVIQATNEKCLQCGMDDYISKPFDEWQLYSAVARFFESG

>ItbHK3

MSLLHVIGFGLKLGNLLLTLCSLVVTLISMNWLSNGGVMTTKTLLDDGEEILTKLWGKISENISKIQHSYSQYIGSKKVRKNWWGLLVIWLGFGAVLAFCAFWCLSTQAMEKRKETLASMCDERARMLQDQFNVSMNHVQAMSILISTFHHGKNPSVIDQRTFARYTERTAFERPLTSGVAYAVRVLHPEREQFEREQDWTIKRMDPQFHENEYNVDNLEPSPIQEEYAPVIFAQDTIAHVISVDMLSGKEDRENVLRARASGKGVLTAPFKLLKTNRLGVILTFAVYKKDLPSNATPNERIEATYGYLGGVFDIESLVEKLLQQLASKQTILVNVYDTTNLSDPISMYGTNVSIDDLEHVSSLNFGDPFRKHEMHCRFKQKPPWPWLAIITSFGIITIVLLLGHIFHATINRIAKVEDDYHEMMELKKRAEAADVAKSEFLATVSHEIRTPMNGVLGMLHMLMDTELDVTQQDYVRTAQASGKALVSLINEVLDQAKIESGKLELEAVSFDPRAILDDVLSLFSGKSQEKGVELAVYISDKIPKLLIGDPGRFRQIITNLMGNSIKFTEKGHIFVTVHLAEEVVVEHESSYALSGFSIEHESSSTLSGFLVADRRQSWKKFKAFQEGFSSFKLTSDQINLIVSVEDTGVGIPFEAQSRVFTPFMQVGPSIARIHGGTGIGLSISKCLVHLMKGEIGFVSLPKTGSTFTFTAVFANGSFSSNELKGQHINDESNSVFSEFKGMRALVVDPRPVRAQVSKYHIQRLGIYVKVVPDLNHGYTCLSTEKTNINIVLVEQEVWDMDSGMATEFVEKLRSYDISCSPKLFVLANCASATRANTSIFGVSTPFVIMKPLRASMLAASLQRALGVNNRGNYRNGGLSGVPLSELLHKRKILVVDDNPVNLRVANAALRKYGADVVCIDSGEQAISHLRPPHRFDACFMDIQMPKMDGFEATKRIRELERQANSQNEHGELLVNASNWHVPILAMTADVIHATNEQCLKCGMDGYVSKPFEPEQLYREVSRFFHVKSN

>ItbETR1a

MESCNCIDPQWPADELLMKYQYISDFFIALAYFSIPVELIYFVKKSAVFPYRWVLVQFGAFIILCGATHFINLWTFGMHTRTVAIVMTTAKLLTALVSCVTALMLVHIIPDLLSVKTRELFLKNKAAELDREMGLIRTQEETGRHVRMLTHEIRSTLDRHTILKTTLVELGRTLGLEECALWMPTRTGLELQLSYTLRHQNPVGFTVPIHLPVISQVFHTNRAVKISPNSPVARLRPAGKYIPGEVVAIRVPLLHLSNFQINDWPELSTKRYALMVLMLPSDSARQWHVHELELVEVVADQVAVALSHAAILEESMRARDLLVEQNIALDLARREAETAVRARNDFLAVMNHEMRTPMHAIIALSSLLQETKLTPEQRLMVETILKSSNLLATLINDVLDLSRLEDGSLQLEIGTFNLQALFWEVHNLIKPIASVKKLSVTLSLSSDLPEYAIGDEKRLMQVLLNVVGNAVKFSKEGSISVSAFVAKSEFLRDPQAPDFFPVITENHFYLRVQVKDTGVGINPLDIPKIFSKFAQNQSLATKNSGGSGLGLAICKRFVNLMEGHIWIESEGLGKGATAIFIVKLGIPGLSNELKPTLVPKLPANHIHTIFLGLKVLLMDDNSMSRMVTKGLLAHLGFDVTTANSGDECFRVVKQEHKVVIIDVSMAVVDGYKLSNQIHEKFSKCHERPFIVGLIGSTDRVMKEKCLRAGMDGVILKPISVEKMRNVLTELFEHGVVLDAQ

>ItbHK1b

MAYRANRTPSISSESSSTPTTPVGSLPERILHKMFGFGNLYRRNQSPTRRRIFRRDVEEEEEEFQYASTLCLSSYYSVFVVRLAIMVMLAILIGLLTLLTWHFTRVYTKRSLNTLAFGLRHELLQRPILRMWNILNSTVEIATAQVKMSEFVMRRYSKAINQEQQVELYEAMKDVTWALFASRKALNSLTINYRNGFVQAFHRDHRSNNTFYIYSDLSNYSISGTYDASMSSSRDGWNDQSIHGNTSAIWYREPLDPLSGVRIGKQSQIQPDELINIAGISQVPDGAASWHVAVSKYSDSPLLSAALPVWDPSNKSIVAVVGVTTALYSVGQLMKEIVEFHSGHIYLTSQEGWLLATSTNTPLLVNSTTRPELIMAIESEDPVIQAGAQCLQKEYGNKIPPGHEVHIENAKLGNQLYYIDSFFLNLRRLPMVGVIIIPRKYIMGKVDERAFKTLVILISASVCILIIGCVCIFILTNGVSKEMKLRAELISQLDARRKAEASSNYKSQFLANMSHELRTPMAAVIGLLDILICDDCLTNEQFATITQIRKCSTALLRLLNNILDLSKVESGKLVLEETEFDLSRELEGLVDMFSVQCINHNVETVLDLSDDMPKLVKGDSGRVVQIFANLLSNSLKFTSSGYIILRGWCENPNTLANSRKFSVNQKDSWSAPKVKLKPHGNHARRPSKKDNNKTVLWFEVDDTGCGIDTSKWESVFESFEQADPSTTRLHGGTGLGLCIVRTLVNKMGGEIKVVKKSGSGTLMQLCLLLNTPIDVTGQHGHLNFREQTMTVLLALNGRMGRLIMSQWLEKNGVHTCEASEWNELTQMLQRLSKTKTNSQGAGNANTSLFVIVIDIGLLDLSTNIWEEQLNFLDKYCGKAKFAWILYHDTANTIKSELRRRGHLLMVNRPLYKGKMIQILEAIVKENSLELQSAVNTTEENLHECHEIDANHSCIASPDDSDNSENGKDKAVNAFRAEERGNEHFAKASSTSQYGTLNNYFVDFTQTNLEDNTSPEDQPRQARNRSVECLGSPRPRESTVSSSNETNQQKSLAGLTILLAEDTPVLQRVATIMLEKLGAKVVVVGDGQQAVDALKLMLNSEDCRNEWSREEGSSTTTQTEGSCSMAFDLILMDCQMPKMDGYEATKAIRRSEVATGSHIPIVALTAHAMSSDQAKCLEVGMDAYLTKPIDSKLMVSTILSLTKSLQA

>ItbHKL4

MMEFWVFIVDCEFGFPGMMLKLLASGLFISSFLIVLAAADNGVRCNCDDIEGVWSIESILECQKVSDFLIAVAYFSIPIELLYFISCSNIPLKLVLFEFIAFIVLCGMTHLLSGWTYYGQHPFQLMLALTVFKVLTAMVSFATAITLITFIPLLLKVKVREIMLKKKAQDLGREVGMIKKQKEAGWHVRMLTQEIRKSLDRHTILYTTLIELSKTLDLCTCAIWMPNEGKTEMNLTHEVRGKDFSNLYNYSIPILDPDVQEIKKSVEVKLLDPKSALADASSGGTSEPGGVAAIRMPMLRVANFKGGTPELVPACYAILVLVIPAGQGRCWGNQEIEILKVVADQVAVAISHAAVLEESQHMREKLVEQNRSLQQAQKDALRANQARNGFQMVMSNGMRRPMHSISGLLSILHDEKLNREQKLLVDAMSKTSNVLSNLVNDVMDTSTKDNRKFPLDFRSFQLHSMIKEAACLIKCLCAFKGNDFAVEVDRSLPNRVMGDERRVFQVILHVVGNLLKISEGGCLKFRVVPEKASQGGNDFRWKTWRSNSSSENVYIRLEIGICSYKSRTEGATSNVSSQKYGSREVEDGLSFSLCRKLVKLMQGEIWMVPNSKGFDQSVAIILPFQLKPSIVLDISGESSNRTNPYFLFEGLEVLLADYDDLNRAVTCRLLEKLGCIVSTVSSGYDCLGALGHGVSSFQVVLLELNLPDLDGFELTMRIRKFQSHGFPLIIALTASSNEDVIGRCLQVGMNGIIRKPVLLQGIADELQRVLYSQTESYHLQNEMMEFWVFIVDCEFGFPGMMLKLLASGLFISSFLIVLAAADNGVRCNCDDIEGVWSIESILECQKVSDFLIAVAYFSIPIELLYFISCSNIPLKLVLFEFIAFIVLCGMTHLLSGWTYYGQHPFQLMLALTVFKVLTAMVSFATAITLITFIPLLLKVKVREIMLKKKAQDLGREVGMIKKQKEAGWHVRMLTQEIRKSLDRHTILYTTLIELSKTLDLCTCAIWMPNEGKTEMNLTHEVRGKDFSNLYNYSIPILDPDVQEIKKSVEVKLLDPKSALADASSGGTSEPGGVAAIRMPMLRVANFKGGTPELVPACYAILVLVIPAGQGRCWGNQEIEILKVVADQVAVAISHAAVLEESQHMREKLVEQNRSLQQAQKDALRANQARNGFQMVMSNGMRRPMHSISGLLSILHDEKLNREQKLLVDAMSKTSNVLSNLVNDVMDTSTKDNRKFPLDFRSFQLHSMIKEAACLIKCLCAFKGNDFAVEVDRSLPNRVMGDERRVFQVILHVVGNLLKISEGGCLKFRVVPEKASQGGNDFRWKTWRSNSSSENVYIRLEIGICSYKSRTEGATSNVSSQKYGSREVEDGLSFSLCRKLVKLMQGEIWMVPNSKGFDQSVAIILPFQLKPSIVLDISGESSNRTNPYFLFEGLEVLLADYDDLNRAVTCRLLEKLGCIVSTVSSGYDCLGALGHGVSSFQVVLLELNLPDLDGFELTMRIRKFQSHGFPLIIALTASSNEDVIGRCLQVGMNGIIRKPVLLQGIADELQRVLYSQTESYHLQNE

>ItbCKI1

MVNFVRSLKTMWPVYLALTLGGAKVEKRDVKLGMILLGINICITVTSVVVFTWWRSRVMMREMCVKAALIKQKEATEEAERKSMSKSVAVANASHEVRTALAGITGLIQMCRADADASAAHSELNDNLRHMESCTNDLYSLLNSILDASRIEAGKMQVEEDEFDLQELLEDVVDLYYPLGMKKGVDVILDPCDESVEKFRRVRGDRGKLKQVLSNLLYNAIKFTDEGYVALRVWARKPSPRPPSQSPPKPKRASSSSSPIAILKGFCGVPAKTGGGEEEEEVNDWVLERKDGGIEYIFKVVDTGKGIPKEKRNTVFENYAQVKDMGRGKKHQLGHGLGLGIAQSLVRLMGGEIGIEDKETGERGTCFKFNIVLDNIVILESSSSHNNNINNTYSSAHHVVVFMHCEERGKIIGRFLESRGIKVSLVQKGHQQLSRKLKKIKRGALNLPRSTTTPSYYSSSSSSKEELEDETMPLHTNTCMVLIMIDTSAAGEALFPEVIKAVSEFHRDLQPGCVRVLWIDTTALSRDNNFQLPSTDLIMSKPLQGSRLHSVLGLLPDFASSSQLGEIQVVIEKEKEEEDEDNGGGSSSTEKKALTGKRILVVEDNPTLRKICTTVVSSLGALTYACTNGEEALQLVSSALQDHHHQPPFDYILMDCEMPIMDGFEATKRIKEEGKAMGIWIPIIALTAHTGKEEMDKVTEAGMDYYLSKPINAASLLTAIHFLDKSTTHL

>ItbERS1

MESCDCVEILLPTDELLVKYQYISDFFIAFAYFSIPLELIYFVHKSAFFPYRWVLMQFGAFIVLCGATHLINLWTFSSHSKTVAIVMTIAKISTAIVSCVTALMLVHIIPDLLSVKTRELFLKTRAEELDREMGLIIKQEETGRHVRMLTHEIRSTLDRHTILRTTLVELGRTLDLAECALWMPTQRGMVLQLSHTLNNLIPVGSTVPINLGIINDIFNSSGAILIPHSCELAKMRSTNTGRHVPPEVAAVRVPLIHLSNFQINDWPELSAKSYAVMVLILPMNGIRKWREHELELVQVVADQVAVALSHAAILEESMRAHDQLMQQNIALDLARQEAEMAIHARNDFLAVMNHEMRTPMHSVIALCSLLLETDLNPEQRVMMETILKSSNLLATLINDVLDLSRLEDGSLELENVTFNLHGVFREVVNMIKPIAAVKKLSTTLSLALDVPIHAVGDAKRLTQIMLNVAGNAVKFTKEGQISIEASVAKPDYIRGSRQGEFYPPSTEGHFYLRMQVKDSGSGISPQDIPLIFTKFTEARSASNRSNSGAGLGLAICRRFVQLMGGHIWIESEGLGKGTTVTFIVKLGSCNYPNAPAIVAPRGRANQGSDDLFKYRQYHRADGSMYAPVPRYQRSL

>ItbHKL6

MSTSRPSQSSSNSARSKHSARIIAQTSIDAKLHAEFEESGDSFDYSSSVRVTSVDAGVQKPRSDKVTTAYLHQIQKAKYIQPFGCLLALDEKTFKVIAFSENAPEMLTMVSHAVPSVGDHPVLGIGTDIRTIFTSPSAAALQKALGFGEVSLLNPILVHCKTSGKPFYAIIHRVTGSLIVDFEPVKPYEVPMTAAGALQSYKLAAKAIARLQSLPSGSMERLCDTMVQEVFELTGYDRVMIYKFHDDDHGEVVSEITKPGLEPYLGLHYPATDIPQAARFLFMKNKVRMICDCRAKHVRVVQDEKLSIDLTLCGSTLRAPHSCHLQYMENMNSIASLVMAVVVNDGDDEGEASESGRIQKRKRLWGLVVCHNTTPRFVPFPLRYACEFLAQVFAIHVNKELELENQIVEKNILRTQTLLCDMLMRDAPLGIVSQSPNIMDLVKCDGAALLYKSKVHRLGITPTDFQLHDIVSWLSEYHMDSTGLSTDSLYDAGFQGALALGDAICGMASVRISDKDWLFWFRSHTAAEVRWGGAKHEPDEKDDGRKMHPRSSFKAFLEVVKTRSLPWKDYEMDAIHSLQLILRNAFGKEADTMDTKANANAIHTKLNDLRIDGMQELEAVTSEMVRLIETATVPILAVDVDGLVNGWNTKIAELTGLTVDEAIGKHFLTLVEDSSVHNVRKMLSLALQGKEEKNVQFEIKTHGQRSESGPISLIVNACASRDVQESVVGVCFIAQDITGQKTIMDKFTRIEGDYRAIIQNPNPLIPPIFGTDEFGWCSEWNSAMTNLSGWCRDEVMDKMLLGEVFGTQKACCRLKNQEAFVNLGVVLNNAITGQVSEKTRFGFFARNGKYVECLLSVSKRLDQEGAVTGLFCFLQLASQELQQALHFQKLSEQTAMKRLKVLAYIRRQVKNPLSGIMFSRKMLEGTELGKDQKSILHTSAQCQQQLSKVLDDTDLDCIIEGYLDLEMVEFKLDEVLQASISQVMTKSNGKSLRIINDIADNILCETLYGDSLRLQQILSEFLSVAVNFTPSGGQLALSSKLTKDNLGESIQLAHLEFRLTHTGGGVPEELLTQMFGSEADASEDGISLLISRKLVKLMNGDVQYLREAGRSTFIISVELAVASKPSS

>ItbHKL5

MKWIKECRTIGTMLRMSVLKLLVLSVHIAIAAADNGFARCNCEYEGFWSIENILECQKVSDFLIAVAYFSIPIELIYFISCSNVPFKLVLFEFIAFIVLCGMTHLLNGWTYYGQHSFQLMLALTVFKVLTAMVSFATAITLISLIPLLLKVKVRELMLKKKAWDLGREVGLIKKQREAGWHVRMLTQEIRKSLDRDTILETTLSELSKTLGLHNCAIWMPNQDRTVMNLTHEVRERNFSDVNDFLIPILDTDVQEIKASDEVKLLEPSSPLAAASSGRSSEPGCVAAIRMPMLRVANFKGGTPELVPACYAILVLVLPSGQGRSWGSQEIEIVKVVANQVTVAISHAAVLEESQHMRDKLAEQNRELQQAQQGALRANQARNAFQMVMSNGMRRPMHSIFGLLSILQEDENLNSEQHLLINATVKTSNVISNLINDVMDCSTKDNRKFPLETRCFELHSMIKEAVCVAKCICAYKGYEFSVEVDKSLPNHVMGDERRAFQVILHVVGNLLKNSNGGCLKFHVVPERSSQGGNDLGWRTWRSNSSRENVFVRFEIGVHGNNSQPEHTTSKVLNPNQKYCGKDFEGSLSFSVCKKLVQLMQGDIWVSPNPMGFDQQVMAVVLGFQLRPSVVIGISEYGDSSNRTHSDSLFPGLNVLLADYDDVNRAVTRRMLEKLGCIVSSVSSGYECLGCLGTTISPFQIVLLDLHLPDLDGFEVTMRIRKFKSRNWPLIVALTSNNDASIRGRCFQVGMNGVICKPLFLQGIADELQKVMLIASRTLS

>ItbHKL3

MSKILALGVLVWVSLVAFTVADNGFFRCNCDYDGFWSIETIMEWQKVGDFLIAVAYFSIPIELLYFVSCSNAPFKLILVEFIAFIVLCGMTHLLMGWTYYGQHSFHLMLALTIFKVLTALVSFATAITLVTLIPLLLKVKVREFMLKKKTWDLGREVGMIKKQKEAGWHVRMLTREIRKSLDRHTILYTTLIELSKTLDLHNCAIWMPNEEKTEMDLTHEVRGRSFLDGHNFPIPVLDPVVQEIKQSVEVKLLDPDTPLAVASSGGVCEPGSVAAIRMPMLRVANFKGGTPELVPQCYAILVLVIPAGQGRCWGNQEMGIVKVVADQVAVAISHAAVLEEVQNMRDKLEEQNRALHQAQQDALRASQARNSFQMVMSNGMRRPMHSILGLLSVLQDEQLNCEQKLLRDTLAKTSNVLSTLINDAMDTSTKQNRRFQLEMRSFQLHSMIKEAICLAKCLCTFKGYEFVVEVDKSLPNHVIGNEIRVFQVILHMVGNLLKSSSGGCIKFSVTREKDGQGGNDLGWRTKSSSEHVHVRFEIGIVGNCSKPEGVYKAAHCSEAYGRREVEEVLSFTVCKKLVQLMQGNISVVPNPKGFHQSMAVVLGFQLGPSTSGMSGCSESSSLTHPSSLLAGLKVLLADHDGINRGVTRRLLEKLGCNVSAVSTGYECLGALGPTACPFQVVLLDLHLPELDGFEVTMRIRKFRSRSWPLIIALTANDDEDASERCIQVGMNGIIRKPVILQGIADELTRVLLLKSRNIA

>ItbHKL1

MGAAMLRWLFLGLLVSSIFSVVSAIDYLCCDDEGLFSVSNILFMQKVGDVLIAVAYFSIPIELLYFISCSNIPFKWVLVQFIAFIVLCGLTHLLNVWTINTQPSFQMIMSLTVAKILTALVSCATAITLLTLIPLLLKFKVRELFLRQNVLELDQEVGMMKKQKEASMHVRMLTLEIRKSLDKHTILYTTLVELSKTLNLQNCAVWMPSGNRAEMNLTHELNPCSAREHHSLSINDPDVLEITKNEGVRLLKQDSVLAAASSGGSGQPGAVAAIRMPLLRGSNFKGGTPELIETCFAILVLVFPSVNDGDLSYDELEIVEVVADQVAVALSHATVLEESQSMQEKLKERNRVLQQAKEDAMKASQARNSFQKVMNNGMRRPMHSILGLLSILQDDNLKPEQKIVVDTLVKTSTVLSTLISDAMEISAKDDGKFPVEMRPFQLHSLIREASCLVKCFAIYKGFDFSTDVPSSLPNQVMGDEKRTFQVILHMVGHLFNVSDGNGSVIFRVASESGAEDGNNKVWNTRKPSSSDDNVTIKFEIEVTIGDSQSGTSVSVVPSGRKRHNSKDVKEGLSFTMCKKLVQLMQGNIWVSSNSRGRGQGMTLILRYQKQSSIRRRIFEYRNPSEQPLPSSMFDGLQVLLADDDDVNRMVTKKLLEKLRCQVSTVSTGFECLSALGPSATSFQVIILDLHMPEMDGFEVAMRVRKFRSRNWPLIIALTASSEDHMWERCLQVGMNGLIRKPVLLQRLAEELQRVLQRAGTEVM

>ItbHKL2

MLRWLFLALFIASVFVSVSAIDCHCDEEGVWGIESILECQKVSDFLIAVAYFSIPLELLYFISCANIPFKWVLVQFIVFIVLCGLTHLLNGWTFSAQPSFQLIVSLTVVKILTALVSCATAITLLTLFPLILKIKVREIFLRQNVLELDQEVDMMKRQKEASLHVRMLTREIRKSIDKHTILYTTLVELSKTLNLQNCAVWMPNEKGAEINLTHELNPGAAARKKCSLSINDRDVLEIKKIKGVRILRQDSVLAAASSGGTGEPGAVAAIRMPLLQVSNFKGGTPEIFSPRYAILVLVLPSTSDHSVWGNNEMEIVEVVADQVAVALSHATVLEESQSMREKLKERNHVLQRAKEDAMKASHARDSFQKVMNNGMRRPMHSILGLLSILQDDNINPEQRIIVDTMVKASTVLSTLMSDAMEITAKHNGKFLVEIRLFHLHSLIMEASSIVKCMSVYKGFGFLADIPNSLPNQVMGDEKRTFQVLLHMVGHLLNVSDGKGSVIFRVVQESGTEEGNNKVWNTRKPSPADDWVTIKFEIEVSVEGSRPDSSVSTIHFGAGRHNCKDVKKGLSFNICKKLVQMMQGNIWMSSDSQGRAQSMTLILRFQKQSSYRRRVFEFKNPREKQLSSSTLEGIQVLLADDDDVNRMVTKKLLGKLGCEVFAVSTGFQCLSALAPSGASFQVIILDLHMPEMDGFEVATRVRNSFRGRGSRPLIIALTASSEEHMWEKCNQVGMNGLIQKPVLLQRLADELQRVLHSAREGP

>ItbETR1b（仅有HATPase）

MVDRRWRVMSENKRCVHKKVFIMESCNCIDPQWLADELLMKYQYISDFFIALAYFSIPLELIYFVKKLVVFPYRWVLVQFSAVIILCGATHFCSATVLVCMPKAYNLIKPIASVKKLSVTLSLSSDLPEYAIGDEKRLMQVLLNVVGNAVKFSKEGSVSVSAFVAKSEFLRDPQAPDFFPVITVKDTGVGINPLDIPKIFSKFAQNQSLATKNSGGSGLGLAICKRFVNLREGHIWIESEATTPAIAATAEAVRGLPRRRHWFATPTTRRRLKTRHSPTSPPLQPTLTTGKICSSYLEFRLSPQTSAPMSTTPPQSPPDSSHRHRQVHRPFHRLKNAPLKLYLWLLTPRQHKMLLFPLTPTSKKIPNNFEQRT

>ItbHK5

MVSEMENAHTEEMDIEVLSSMWPEDINEAGKQFNIEQPGADLDMLEEVTINEEATTIVDFQRLMELTDYSDKGSSQLAYLVKNWEYKQANAVRLLREELDYLSKQQQESELKKLEILEQHRFEEERYGGDKRPVSILDEDLKYIYQDIPRRKKDVVVQHEKLEIEAEYDSIIYWKQRALHLQKLLAASIERENVLLEKLQESIEKLERQSSPVEELSQVLKRADNYLHFVLQTAPIVIGHQDKELRYRFIYNHFPSLREEDIIGKTDVEIFSGSGVKESQDFKKEVLERGLPAKREITFETELFGSKTFLIYVEPVFSKAGETIGVNYMGMEVTDQVRKREKMAKLREEIAVQKAKETELNRTIHITEETMRAKQMLATMSHEIRSPLSGVVSMTEILATTKLEKDQRQLVNVMLSSGDLVLQLINDILDLSKVESGVMKLEATKFRPREVVKHVLQTAAASLQKLLTLEGFVAEDVPTEVIGDVLRIRQILTNLISNAIKFTHEGKVGIKLYVVPEPSLGAKQGSHQKQSLDSLKSSSNNWKEDRCLSASHGKHDRTASFSYKDGEGTFENQMHKDGSNHSVSSGALDEDLDAHPDQEEKTVWICCDVYDTGIGIPENALPTLFKKYMQVGADTARKYGGTGLGLAICKQLVELMGGHLTVSSKEHHGSTFTFVLPHKVSPLCESSDENDEMSDMGSHDTSTDANEDDANSGFFQFQPRTLGSLFSSHGSGRAQKLSPNTFGFNTLHSCNGLPKNSYTFPANSVMLKDMGSVEDACSVIDVDILSDPESSFRQSSHSDNPSTLERDKHAHSGSNGQCHHHSSYSTDSTSTRKDEDVKTAVQEKRQPEGNSPCSSGNNQEVSKSAPKPRILLVEDNKINVMVTQSMMKQLGHQIDIVNNGIEAVRAVQRSSYDLILMDVCMPVMDGLQATRLIRSFEETGNWDAARTAGVEEVPSSSLSLKRSDSKSSNGRIPIIAMTANALSESADECFANGMDSFVSKPVTFQKLKECLQQYLPQRHRL

>ItbHKL7

MSSRSGTIRTNCSMSSSARSRHDARVVAQTSIDAKLHVEFEESEEQFDYSTSVNLSNSTSNIPSSTVSAYLQKMQRGSLIQPFGCLIAIDEHNFSVLGFSENAPEMLDLAPHAVPSIEQQEALTIGTNVRTLFRSTGAAALEKAASFEEVSLINPILVHCKNSGKPFYAILHRIDVGLVIDLEPVNPADVPVTAAGALKSYKLAAKAISKLQSLPSGDISLLCDVLVREVRDLTGYDRVMVYKFHEDEHGEVVAECRKPDLEPYLGLHYPATDIPQASRFLFMKNKVRMICDCLAPSVKVIQDKTLAQPLSLCGSTLRAPHGCHAQYMANMGSIASLAMSVTINEDDDEMDSDQQKGRKLWGLVVCHHSSPRFVPFPLRYACEFLVQVFSVQINKEVELAAQRLEKHILRTQTVLCDMLLRESPVGIVTKSPNIMDLVRCDGAALYYRNKFWLLGATPTEPQIRDIAQWLLDSHSSSTGLSTDSLMEAGYPNASVLGDSVCGMAAVKITSKDFLFWFRSHTAKAIKWGGEKHDPGDKDDGRKMHPRSSFKAFLEVVKRSLPWEDVEMDAIHSLQLILRGSLQDEVVDNSKMIVNVPAVDTSIQRVDELRIVTNEMVRLIETASIPILAVDTSGCINGWNIKVAELTGLVVQEAIGAPLVDLVVSEAVSTIKNVLSLALQGKEEKNVEIKLKKFGSQENNDPVILVANACSSRDVKGNIIGVCFVGQDVTGQKLITDKYNRIQGDYVGILRSPSALIPPIFLMDEHGRCLEWNDAMQKLTGLKRAEAIDQMILGEVFTVSSFGCKVKDSDTLTKLRILLNGVIAGQDAEDLLFGFFDKQNKYVEALISANKRTDVVGRITGVLCFLHVPSPELQYAIHVQKLSEQAAANSLKKLAYVRREVRNPLNGIKCIQNLMKSSDLSKDQMQLLKTSTMCQEQLAKIIDDTDIESIEESYMEMNCCEFSLGEAIKAVVNQAMIPSRERQVQIMCDLPVEASSLYLFGDNLRIQQVLSDFLTTAVLFTPHFEESSVLFRIIPRREQIGAKMHVVHLEFRITHPAPGIPEELIQEMFNYSQSMSREGLGLYISQKLIKIMNGTVQYLREAERSSFIILVEFPASLRSDHQ

>ItbHKL9

MASGSRSKNVQQNQAQSSGTSNVNYRDSVSKAVAQYTVDARLHAVFEQSGESGKSFDYSQSVKTITQSVPEKQITAYLSKIQRGGHIQPFGCMIAVDEPSFRVIGYSENAREMLGLTPQSVPSLERPEILAIGTDVRTLFTPSSSVLLERAFGAREITLLNPIWIHSKNSGKPFYAILHRIDVGIVIDLEPARTEDPALSIAGAVQSQKLAVRAISHLQSLPGGDIKLLCDTVVESVRELTGYDRVMVYKFHEDEHGEVVAESKRPDLEPYIGLHYPATDIPQASRFLFKQNRVRMIVDCNATPVQVIQDESLMQPLCLVGSTLRAPHGCHAQYMANMGSIASLTLAVVINGSDEEAVGGRNSMRLWGLVVGHHTSARCIPFPLRYACEFLMQAFGLQLNMELQLASQLSEKHVLRTQTLLCDMLLRDAPTGIITQSPSIMDLVKCDGAALYYQGKYYPLGVTPNEAQIKEIVDWLLTYHGDSTGLSTDSLGDAGYPGAASLGDAVCGMAVAYITSRDFLFWFRSHTAKEIKWGGAKHHPEDKDDGQRMHPRSSFKAFLEVVKSRSLLWENAEMDAIHSLQLILRDSFKDAEASNSKAVVRAPPGELELQGMDELSSVAREMVRLIETATAPIFAVDVEGRINGWNAKVAELVGLSVEEAMGKLLIQDLVHKESQETTEKLLFNALRGEEDKNVEIKLRTFGTEEDKKAIFLVVNACSSKDYTNNIVGVCFVGQDVTGQKIVMDKFIHIQGDYKAIVHSPNPLIPPIFASDENTSCSEWNTAMEKLTGWSRGETIGKLLVGEVFGSCCRLRGPDAMTKFMIILHNAIGGQDTDRFPFSFFDRNGKYVQALLTANKRANIDGQIIGAFCFLQIASPELQQALKIQRQQENKCFSRMKELAYICQEIKNPLNGIRFTNSLLEATDLTEDQKQFLETSAACEKQMSKIIMDVDLENIEDGSLELEKEDFFLGRIIDAIVSQVMSLLRERGLQLIRDIPEEIKTLAVNGDQVRIQQVLADFLLNMARHAPVPGGWVEIQVRPSLKQVSDGTNVVHTEFRIMCPGEGLPPELVQDMFHSSRWVSQEGLGLSMCRKVVKLMNGEVQYIRESERCYFLIILELPIPRRGSKSIIIG

>ItbHKL10

MAGSGTGSSSKRFIEHQSSSAQIAQSSGTSNSNNRYPVSKAVAQYTEDARLHAVFERSGGTGKSFDYSESVKVATPFVAEQQIAAYLSNIQRGGHIQPFGCMIGVEEGSYRVIAYSENAREVLGLMPQSVPSLDRPDILGIGVDVRTLFRPSSSVLLQRAFGAQEITLLNPIWVHSKNSGKPFYAILHKIDVGIVIDLEPARSEDPALSIAGAVQSQKLAVRGISRLQSLPGGNIKHLCDVVVECVRELTGYDRVMVYKFHEDEHGEVLAESKRPDLEPYIGLHYPATDIPQASRFLFKQNRVRMIVDCNATPVRVIQDESLKQPLCLVGSTLRAPHGCHAQYMANMGSIASLTLAVIVNGNEDEGVGGRNSMRLWGLVVGHHTSARSIAFPLRSACEFLMQAFGLQLNMELQLASQLAEKHVLRTQTLLCDMLLRDSATGIVTQSPSIRDLVKCDGAALYYKGKYYPLGVTPTEDQIKDIAEWLLTYHGNSTGLSTDSLADAGYSGAASLGDAVRGMAVAYITSKDFLFWFRSHTAKEIKWGGAKHHPQDKDDGQRMHPRYSFKAFLEVVKRRSLPWENAEMDAIHSLQLILRDSFKDAEASNSKAVVHAPPGELELQGMDELSSVAREMVRLIETATTPIFAVDAEGHINGWNAKVAELVGLPVEEAMGKSLVHDLVHIESQETTAKLLFNALRGYEDRNVEIKLKTFGTEQHTKAVFVVVNACSSKDCTNKIVGVCFVGQDVTEQKVVMDKFIHIQSDYKAIVHSPNPLIPPIFASDENACCSEWNIAMEKLTGWSKGEMIGKMLIGELFGGVCRLKGPDAMMKFMITLHHAIGGKDTDKFPFYFFDRNGKYVQTLLTANKRVNMDGQIIGAFCFLQIASPELLQAIKIQRQQENKWLTKSKVMAYICQEIKNPLNGIRFTSSLLEATNLTEHQKQFLETSAACEKQMSKILRDAGLENIEDGSLELEKEEFHFGSVIDAIVSQVMLLLRERGLQFMLDIPDEMKTLKVYGDQARIQQVLADFLLNVVHHAPTPKGWVKIQVRPSLRQSSDGITIAHVEFRFICPGEGLPSALIQDVFHNSEWETREGLGLSMCRKIVTLMNGEVRYVREAERCYFLVILKLPVPTSGSKSG

>ItbHKL8

MDLQSQENKPPTSSKKMENYAKAATFSSSATSNLNTGKAIAQYNADAKLMAEFEQSRESGKSFDYSRSVIGAPQNVTEEEMTAYLSRIQRGGLIQPFGCMLAIEEPSFKILGFSENCFDLLGLKSGVEPPERMSLIGIDARTLFTLSSRASLAKAVASREISLLNPIWVHSKTNQKPFYAVLHRIDVGIVIDLEPANSADPALLLAGAVQSQKLAVRAISRLQSLPGGDIGTLCDTVVEDVQKLTGYDRVMVYKFHDDSHGEVVSEIRRSDLEPYLGLHYPATDIPQAARFLFKQNRVRMICDCNAQPVKVFQSEELKQPLCLVNSTLRSPHGCHTKYMANMGSIASLVMAVVINSSESMKLWGLVVCHHTSARYVPFPLRYACEFLMQAFSLQLYMELQLASQLAEKKILRTQTLLCDMLLRDAPFGIVTQTPSIMDLVRCDGAALYYDGKCWLLGVTPTETQVKDIAEWLLHNHGDSTGLSTDSLSDAGYPGAPLLGDAVSGMATARITSKDFLFWFRSHTAKEVKWGGAKHHPEDKDDGGRMHPRSSFIAFLEVVKSKSLPWEDSEINAIHSLQLIMRDSLQGIGENYMKSVSSPQQTDSEGTRFYELSSMALELVRLVETATVPIFGVDSSGLINGWNAKIAELTGLQANVAIGKYLIDDVTHEDSHETFKGLMCRALQGEEDRNVEVKLLKFGEHPTKEVVYLVVNACTSRDYKNDIIGVCFVGQDITPQKAVMDKFVRLQGDYEAIIQSLNPLIPPIFASDENACCSEWNAAMERLTGLVKCEVIGKRLPGEIFGGLCRLKGQDALTKFMILLYQGISGHDTEKLSFGFFDRKGNFIDVFITANKRTDERGNIIGCFCFLQTMAVDPQTSARDIEDDRECLSTLKEFAYIQQQMKNPLNGIRFTHKLLEGTVTSDHQKQFLETSEACEKQILSIIENMDSGGIVDGNRVELKTEEFVIGNVIDAVVSQVMIPLKEKNLQLLHDIPDQIKSLPIYGDQIKLQLVLSDFLLSIVRHAPSPDGWVEIRVSPGLKLIQDGNEFIHIQFRMTHPGQGLPSALIEDMVRGGTRWTTQEGIVLHLSQKLVRMMNGHVHYVREQQKCYFLIDLDFKTQKPRSRESSMDTSRIT

>ItbHP1

MEVSQLQNSFLGYMAELFREGFLDAQFSQLQQLQDESNPTFVAEVVTLFFEDSERLLNDLNTTLNQPDVDFKKVDAHVHQLKGSSSSIGAQRVKNVCVAFRNFCEEHNIEGSLRCLQQVKQEYLLVKNKLETLFRVSQTQIPHFY

>ItbHP2

MDVVPQLQKQFVDLIASLYREGFLDDQFLQLQKLQDDSNPDFVFEVVSLFFEDSEKLINNLATALQQPVVDFNQVDAHVHQFKGSSSSIGAQRVKNACVSFRNFCEEKNLDGCVQCLQLVKNEYFVVKNKLETLLRLEQQILAAGGKIPVLP

>ItbHP3

MEVVGQLQKQFVAYMASLYREGFLDDQFLQLQKLQDQSNPDFVVEVVSLFFEDSEKLINNMANAFQQQVVDFKQVDAHVHQLKGSSSSIGAQRVKKACVSFRNHCEERSLDGCVRCLQVLKNEYFLVKNKLETLIRLEHQILAAGGTIPLLS

>ItbHP4

MPSQAAKLRKGLFDQGYIDDQFIQLEELQDDANPNFVEEVVRLFFNDSTRQIHNIELALGSGACDFTKLDDMMHQFKGSCSSIGARKVKKECSEFQQYCDAGNVEGCRRAFQRLKQEYYTLEAKLDTYFQMAKQDS

>ItbHP5

MERNHLPRQLATMRKSLFDQGYLDDQFVQLEELQDDVNPNFAEEVVTLFYRDSARLVQNIEHALERSPLDFAKLDGLMHQFKDSCSSIGARKVKYECTQFREHCRVANAEGCKRSFLQLKKEYSTLQKKLKAYFQFARQAGPVEVACRPN

>ItbHP6

MADEHAQQIANLRQSFDQQLLNEKFIRTEEIDVGLPGFQEDFYCTYFREAERLVGILKLELHGPPFDVESIEMYLHEFRSNTKSAGAVKVLTKIDRCIECCSVLDFAGCLACLEEANTEMKYLKRQIKNYFMVLIEHLLFVHKL

>ItbRR1

MARNGVFSRRRTAAEMEDSDEVVLSEESHDVHVLAVDDSLVDRKVIEKLLKITACKVTTVDSGRRALQILGLDEEKTSVQFDGLKVDLIITDYCMPGMTGYELLKKIKGSSFREIPVVIMSSENVLARIDRCLEEGAEDFLLKPVKLSDVKRLKSYMFGDDRFHGEDGGTNKPSESPEISDDTSSSSAPSLSLSPSPTTSMDLSSCLSLSPSPTSIDLSATPSPPSTSSPSSPKTFSSSSSPSTNSSSPPSPVAPASPTRILKRRDGD

>ItbRR2

MARNGVFSRWRRAEGPAGLSLPSESHDVHVLAVDDSLVDRKVIEKLLKITSCKVTAVDSGSRALQFLGLDREESSVGFDGLKVDMIITDYCMPGMTGYELLKKIKGSSFREIPVVIMSSENVLARIDRCLEEGAEDFLLKPVKLSDLKRLKSHMFGEDDKNPREDSGINKRKLQEMSEDSSPPLPSPSPLLSPNPSTDLSSSSSSSSSPPSTSSSPSSPELLESPKTEE

>ItbRR3

MVIGKPEKVAAGDDCCSGGVQELHVLAVDDSHVDRKVIERLLKISACKVTAVESGSRALQYLGLDGEKGSAAIDGLKVNLIMTDYSMPGMTGYELLKKIKGSSALREIPVVIMSSENILARIDRCLEEGAEEFLMKPVKLSDVKRLKDFVLRGDGESKEGATTTRKRKPTDDSFPRPPLSLSLASSSPSIHPETTTPLSPRCSSVPLTKHPRLHQDTEPLVDP

>ItbRR4

MGRNMRTEKIAAVDGCSSTFGGGGRELHVLAVDDSYVDRKVIEKMLKISCCKVTAVDSGSRALQYLGLDGEESSVATDGLKVNLIMTDYSMPGMTGYELLKKIKGSSALRQIPVVVMSSEKILARIDSCLEEGAKEFLMKPVKLSDVKRVVDFILRGEEDGKETESTTDNTPLSPESYLANIARISLDASNRT

>ItbRR5

MATSSRNGGDESPHVLAVDDNLVDRKLVEKLLKNSSCRVTTAENGLRALEYLGLGDEHNTSNDNGSKVNMIITDYCMPEMTGYELLKKIKESSNMKDIPVVIMSSENIPTRINQCLEEGAQMFMLKPLKHADVKRLRGELMQCRG

>ItbRR6

MGMAAADPQFHVLAVDDSLLDRKLIERLFRTSSCQVTAVDSGSKALEFLGLLEHGQDCQTQPSVLPNHNQEVEVNLIITDYCMPGMTGYDLLKKIKESSSLRNIPVVIMSSENVPSRINRCLEEGAEEFFLKPVRLSDVDKLKPHMMKTKGNKHQKAGTDDDTQEHKETSSEESSSVESGVTDVQSQLPQLPLEQPQSETQQHQPPPPDNTNNCNNKRKAMEEGLSPDRSRTRYNGLTSL

>ItbRR7

MGMAAVEPQFHVLAVDDSLIDRKLIERLLKTSSCQVTTVDSGSKALQFLGLNEDDQKNPIQSSVSPNNHQEVQVNLVITDYCMPGMTGYDLLKKIKESSSLRNIPVVIMSSENVPSRISRCLEEGAEDFFLKPVRLSDVNKLRPHMVKNKKAGEQEIQESSSSEESSAESGMTDVQSQAESNDNRCNKRKALDEGFALKRTRPRCNSLSDFSDL

>ItbRR8

MGMAAAESQFHVLAVDDSLIDRKLIERLFRTSSCQVTTVDSGSKALEFLGLHEHDDENNTNHHPSVLSNHPQPQEVEVNLVITDYCMPGMTGYDLLKKIKESSYLRNIPVVIMSSENVPSRISRCLEEGAEEFFLKPVRLSDVNKLRPHMMKTKCKKPENEPRDSQEPSPEHPLIQQECAVEDVKLQAQTEEQQQVMNSDEDSRKAIEESLSPGRTRTRQEPPQGRGDEPQPLVNNSNENKRKAMEERLSPDRTRPRYNNNGLTHCCL

>ItbRR9

MACPSSSMAMGENGEDEVIHVLAVDDDPVNLIILEKLLKASSCKVTAAENGMRALEYLGLLGDDDQHNTPNTNVPRIDLIITDYSMPGINGYQLLKKVKESAMLKDVPVVVMSSENVPSIINQCLEEGALKFMPKPLNRSDVKHLISQLP

>ItbRR10

MSAVMHVLAVDDSTVDRTIVEKLFKAASCKVTTAENGLRALEYLGLLAGDDQNNSPNTNVPKLNLIITDYSMPEMNGYEFLKKVKGSAMFKDVPVVVMSSENTPSQINQCMEAGAKKFILKPLKQADVNQLKSQLMQS

>ItbRR11

MECPSSMVTVGNGEEDAVMHVLAVDDSVVDRTIVEKLFKSSSCKVTTAENGVRALEYLGLLPGDDQHNSPNTNGSAMFKDVPVVVMSSENTPSQINQCIEAGAKMYILKPLKQADVNQLKSQLMQS

>ItbRR12

MAAAVAAATHASESRFHVLAVDDNLVDRKLIERLLTTCSYQVTAVDSGNKALEFLGLLEDSVTALNSDPHEVEVDLIITDYWMPGMTGYDLLRKVKECRRDIPVVIMSSENEASRINMCLEEGAQEFLVKPVRQSDVTNLIKPRAFVKGGDDNGVVSPVYCSGVDDNRHGTATATETVISPPAER

>ItbRR13

MATSSCSDLGKFHVLAVDDSIIDRKLIERLLRTCSYQGAEEFFLKPVRQADVNRLSSHLLRPKSPEPSPCRKRKAAPAEAQPTRPIRRRRLI

>ItbRR14

MPFALPAVVKCRGAIDALRILQEGKEEIDIVLSELHMSRVNGFKLLDQIIGLQIDLPVVMMSSDERVDAIKKIVIQGACGYLLKPVRREEIKLLWQHVVRHKQGNLGKGTRPPQAAEFWDSGEMPRQQKSVENCGSSNNNRDEDTNATATTVKVKKPRLVWTPQLHQQFVAVVNQIGLRNAVPKKILDLMNVPHLTRENVASHLQKYRLHLQRNGDQNSYKRLSIHHLHYEDMVLNKNNAQQAVMPAVFDHPDGRDYNYVNVYNYGGLGRGVGGSTTPMDAINYFSTFSPHQPQSTGYTYNNNNPNQLGNTSSASSKRQQDQTQVCT

>ItbRR15

MESVMAGGIFLPRSETFPAGLRVLVVDDDPTWLKILEKMLKKCSYEVTTCGLATEAISLLRERRNGFDIVISDVNMPDMDGFKLLELVGLEMDLPVIMMSVDGETSRVMKGVQHGACDYLLKPIRMKELRNIWQHVVRKRMQESRDIENHEGDQFDEAWMFNGIELQSGKKRKDFDYKFDERETSDSRSGDPSSVKKPRVVWTVDLHQKFVKAVNHIGFDKVGPKKILDLMGVPWLTRENVASHLQKYRLYLTRLQKENELKASSSGTKHPDLSPKESPSSACLQNLVDVKPSKSTNGKYAFHGEKFCVQEVESRNYKGEVKAAAPLSTTGVSRAQVGENCDSQKSISCSKASWASEVSKTGFKHEFKPQIQTEDNVNHLPSPKLPRNVPLDQAQPLLNLAPHKDINPGEIKSKPGNINTENPGVRTVSPLECAVDLLPAQPSQPQSCLTNFQAFEQIPSTTWSAKTPQILINGLESVEGNLFLGGGSWHKDFNAAALQGEFHSPCVVGPQSLELLDYSNTNLTGEIQPYFYDYEYAIDPVIDHGLFIL

>ItbRR16

MAAVCKAEAAAVVPEQFPVGLRVLVVDDDLLCLRIIEQMLRKCKYNGLFLFLLVTICSQATAALNLLRERRGCFDIVISDVHMPDMDGFKLLEHVGLEMDLPVIMMSADGRTNLVMRGIRHGACDYLIKPIRDEELKNIWQHVVRKKCNLSKENDHSGSFEDNDQPKQGGDDAEHASSVIEGADGVLKTVKKKRDFKDDDDDDDDDEIENDDPANAKKPRVVWSVELHQQFVSAVNQLGIDKAVPKRILELMNVPGLTRENVASHLQKFRLYLKRLSGVAQQQGGLPNSFCGPIEPNPKLGSLGRYEIQALAASCQIAPQTLAAIHAELLGRPTSGLVLPTIDHPALLQASLPATKYILDDQAVAYGQPLMKCPPNISKQFTQHLSAEDIPSGVVAWPPKNVCVVPSINLSGLGAQNGNMLTTMMQHHQQQQKQQQMEQHQKLSTIPESCRPVNVRPSCLVVPSQSSANFQVTNSPASISQTSSFSKSNVMDSRILSPQSGNSSSGAGEVANWKQKLPCRSNMLCATGSLSPSLSSCSTNADNSASWQVQNSACIIGASRHAAGVVPNITSIPVPDNHKSNQLLDQGPIRNLGFASRGSSIPSRFAIDESESPPISNIYHSRIYKETNTCKVKQEPDVNIADNAKVSVQTLQRIPPNDFMSVFQ

>ItbRR17

MNLGGGQVGKGMSATCSNASWKSGDAVSDKFPAGLRVLVVDDDPTCLKILEKMLRTCLYEVTKCNRAELALSYLRENKNGFDIVISDVHMPDMDGFKLLEHVGLEMDLPVIMMSADDSKNVVMKGVTHGACDYLIKPVRIEALKNIWQHVVRKRKHEWKDKDPEQSGSADEGDRPQKPSDDADYSSSVNEENWKNSKKRKDEEDEAEERDDTSTLKKPRVVWSVELHQQFVAAVNQLGIDKAVPKKILELMNVPGLTRENVASHLQKYRLYLRRLSGQGGLGNSFMGHPESPFGSMSSLNGLDLQALAASGQISAQSLATFQAAALGSSVTKSAISMPLVDQRNLFSFENPKSRFGGDGPPQLGNSSKQIGLLHGIPTTMEPKQLASLHQSSPTFGGMSMQLNSQVHQNNPLLMQMSQPQPRAQMVSDPNGSQASRLPLSVPQPILSSAMAGGVLGGNSIVDNSCSAIHSSVSHAPSTVAFSVNQGTELQTNSYTTSNSGVSSLTSRGMLREQANPDVKGSRGFVPSYDIFNDLHQHKAQDWGLQNVGSTFDPPPHHSNLQGILDPPPSVMAQHGFSSNQKSGQNRNAPINKDVFLSGEQTGHGNNPMLGPQFNSLLGGNPVTIKTERLPDTSFQNTLFSDQYGQEDLMSALLKQQQDSLGPVENEFGFDGYQLDNLPVLHPTMPIHSRTNISLEPK

>ItbRR18

MSVHSSAASWKPVDVVSDQFPVGLRVLVVDDDPTCLRILEKMLRNCHYEVTTCNMAEVALSLLRENKNGFDIVLSDVHMPDMDGFKLLECIGLEMDLPVIMMSADDSKNVVMKGVTHGAYDYLIKPVRIEALKNIWQHVVRKRKQEMKDKDIEQSGSVEEGDRQQKPSEDVDYSSSANEGNWKCLKKRKDEEDEGEERDETSALKKPRVVWSVELHQQFVAAVNQLGIDKAVPKKILELMNVPGLTRENVASHLQKYRLYLRRLSGVSQHQSGLNSSFMGPPDTTFGTMSSLNGLDFQTLAATGQISAQSLASLQAAALGRSATKPAISMPLVDQRNLFSFENPKFRFVEGQQPLNNNSKQIGLLHGIPTTMEPKQLANLNQSSQTFGSRGMQPRGMQPRAHQNNSLLMQMGPPQSQAHMLNEPNGTQVSRVTQPILSNGMPSELLARNGIVDNSRGAIYQPVSQAQPLVDFSVNQNTEMQGNSFISGNSGMSCLASKRIEVNSDVKRPIGGFAPPSYDIFNDLQQHKAQDDWGMGAVFEASRLPNAQGTLDASQSVMVQQGFSSSQNSGQNGGMSIGKAVFPSGQESGNPMVGPQLNSLLSGNSITIKAERLPDASYQNTLFSDQHGQDDLMSALLKQQESVGPVENEFSFDGFQLGNLPV

>ItbRR19

MTVEQRNDKQNDQFPLGMRVLAVDDNPTCLMVLENLLRKCQYHVTTTNQAIQALQLLRENKNQFDLVISDVDMPDMDGFKLLELVGLEMDLPVIMLSAYSDTNLVMKGITHGACDYLLKPVRIEELKNIWQHVLRRKKFDCKEQKISNKPDGESGELGRGFRGMGETDRNGKLTRKRKDQSDDEDEELDENGGRNEDPSAQKKPRVVWSVELHQKFVAAVNHLGIDKAMPKRILELMNVEKLTRENVASHLQKYRIYLKRLNSVASQHANMVSVLGSADPSYLRMGSLNNIGNIPFITGCTQFSDAPLRSISSGSVLTRLNTPSGIGMCGFAPSSMIQLANAPNSSSSITSEINFRQSIQPGNQDMDILEGMPMPLGTDQVNNNLGVTHLYPFSNGVPERKIDVDGRRNLTIGVSDNSIILRSQGQCVQRKDFLDNQFPVIASPVSSASSPFLNTTRCNDNWPTASQSSLLEANSFGTSVYSHHAMPRDLGNNGSTLEVPMSSNLHNPLNSACPQVPDTRTEMQCLTTIIDNVSGVKMNFSPRQDWHDFEPDSAHVPSLVCSSAHTFLPPDGGQRQQQHHEFENAAVDVKQEYLEEQKKLAGNNAYGQMG

>ItbRR20

MTVDESRRRVEKENNSDNFPVGMRVLAVDDDPICLKLLECLLRKCQYHVTVTNQASVALKMLRENKDRFDLVISDVYMPDMDGFKLLELVGLEMDLPVIMLSANSDTGLVMKGVTHGACDYLVKPVRIEELRNIWQHVIRRKTFDSKHHSKSGDQDNEEEGRQGDQLSGTAEQNEKLNKRRKDEEDGSENEDPATQKKPRVVWSAELHRKFVAAVNHLGIEKAVPKRILDMMNVEGLSRENVASHLQKYRLYLKRISLVSTPQANMAMPYMPMGSLGGFGDLQTLAGPGQLNRATLSPYVPGSLLGRLNSSAGVSLQNRNLLGLLQPSHAQSSGNSLDLLGKLNSNAPPTSQNPSLFLGTQMQHGKFTKIEQVLNPMDNSKLLTAATTFTGSGSAFGNPINATILQGNSQQGQTGEGFGNPHSLNMTSLRSEPLNTGVSSASNFLGHGGPNGNLGNSILASNVQPNCYPLMETFTHSQLDQNHVRGNYSPAGPPLQSSPLGYNSTFSTSIPYENSRGQTQYQEGFIGDAIQSVNQAPTQFWGDHNSNSNNVFSNSSSQILGNGLMPPLSQIADQNNDIFNMKTDTPLIGQENGGSVVLFPHNENANFNQDSRMGSNGDYMLNSTKPQGAYSSLDDLMNGVIKGEQNGQFGFDDYLFGS

>ItbRR21

MTVEETMRNMGVDRENYHNFPLGMRVLAVDDDPICLKLLEGLLRKCQYHVTTTSQARMALNMLRENKDRFDLVISDVHMPDMDGFKLLELVGLEMDLPVIMLSANSDTKLVMKGITHGACDYLVKPVRIEELRNIWQHVIRRKKSESKGQNQDNGYRGNGEGGQGFPLTGSAEQNALLNKKRKDEEDETNENEDPSSQKKPRVVWSIELHRKFVAAVNQLGIEKAVPKRILELMNVEGLTRENVASHLQKYRLYLKRISSVATQQANMVAALRGKDSAFMRMASLDGLGDFQALGGPGRFNHATLSTYTPADMLGRLSSATGVSIRNLSASALVQSNHAQNLENSLGSDGNLNPNISLSSHNAATLFQGIPSPLAVQELNPLENSRALTAATAFADSGSVIGSSMNPMMLQGSPKQGLIGGGFGNQHSLNMASLSSELYNTGVNSSSNFLGHGRSSENWQTSIQVSEFQSGSYPLTETFSHIQLPQNCEREHESSAATHLHSSPVGFSSTTSASTTFEDSREPQVPSQLWGDSKQNQNSNDIFRNLSSHVPPSLSQGMYQTSGNLNTKMNSFLMHRSNAGSLVLFHQNGNEMPTPDPRTRSIEDNLLESTKTHGAFVSQGFDALDDLMNAVIKQEQDGGILVGEFGFDAYPFGSCM

>ItbRR22

MTVEEIRGHMGSEKGNHDSFPVGMRVLAVDDDPICLKLLESLLRKCQYHVTITSQARMALKMLRENRDRFDLVISDVHMPDMDGFKLLELVGLEMDLPVINLRIDPIPLKFFSVLSANSDTKLVMKGITHGACDYLVKPVRIEELRNIWQHVIRRKKFDSKSQNKSGDQDRSPHGGGEGGQGGPLSGSTDQNGKLNKKRKDEEDESDENGHENEDPATQKKPRVVWSIELHRKFVAAVNQLGIEKAVPKRILDLMNVDGLTRENVASHLQKYRLYLKRISSVATQQANMVAAFGGKDSAYMRMGSLDGLGDFRTLAGSGRFSHASLSSYTPGGMLGRLNSAAGVSIRNLTSPSLIQPSHGQNLGKPIGTLGKLTPNVPAVSQNACLFQGIPSSLELDQFQQSEGTPHIRGDLNPLDDSTLLGAANTFTDPGSGIGSSSNPMMLHGNSQQGLIAGGIGNQHSLNMASLNSEHFNIGVGGSSNFLDHGRASDNWQNPIQVTNFQSSSLPLTETFNQGQMQQNCARENNSSIGPHLQGGCAGYSSLASTATPFEDSRGEIQRRERLVGDAIPSINQVPSQQWGELKQNPNSNGVYSNLTAQVPASSIVPPLSQSMDQCNDTGNRRIDASGQSNLGSSVLLQHNKNEKLTSESRARYYEDYLFEPPKPQGAFPSQGYGSLDDLMSSVIKREQDGATLQGEFGFDAYSFGPCI

>ItbRR23

MSGSSTMMRLSMESERSVKVIVVEDDYDSDPTCVHMLRKLNFQVEVVKHPKDAFDTLVRTRGEFDVVISDVNMPDINRFQQMIAQQFGLPVLLICDEKQEGTFGQKMQNFLRKPVSAYELKDLWQIITAHQKNCKLSIDKNAVPNKILRVMNEPGLTREHVSSHLQKYRKFKISGSKRMNKLARNDKVYHTQQSQQDVSSSYSSHHASSKGCHLGLSSFEQDEQMQNLTVSFFAKNENENLRVEGGKQQSLVSPVLENNIIYQETTVQMQIANNYAYDDNTARQDNSYGIGYHWIDFANDDFYNQLTINQCLPNQVRYY

>ItbRR24

MASGNDSLKVLVVDDDSTCLAIVAALLVKMEFQGMVSWLFSILSTPFKFRAWPVYLPCFFVVFSGLILGKLALVFGNVVALKNGNDAIEALRTQGRFDVVISDVHMPGMNGFELQELIFKQFRIPVVLMSGDYEEGIVRQAMQNGAISFIRKPVSPNDLRGIWQYIIAQKRSKATTEQVNTDDQDNAVIGGSSSGTHSYSGYDTTSKKKMVWTDQIHFIFLDAISSLGPENATPKNILKAMNVPGLTRENVGSHLQKYRQFLRRNIQDLENGNNGGGGNQPRFGSRKFRRLAKLEETFAQGGLGAALSSPTSSAALGRSSGDARIRGGRGGSPERGQQGVLLSADDESMFYRGLRRGPMITSPNESSPASYEVNPGGSKESSPYPTLTALMQNRLDSSSNDDNANVSGFGNVNVVSGNEDVQVPGEGNFDIYDAILNFIGETEDGGDENISMYGNITAGQDNSGYNQIGNLSMISSIK

>ItbRR25

MDMDSKNVGKGLRALVVEDDRAMQMVHKMLLEKYGLEAQVANNGEEAVELHRSGARFDLLLMDKDMPVKDGVNATRELREMGVKSMIVGVTSHEPGAVMDEFIAAGLDECLTKPLGQEVILGFINQLVA

>ItbRR26

MLLKKYGLEAQVAKNGEKEMVLNCFDLLLMEKEMPVKDGVNATWELREMGLKSMIVGVTSHGPGEVRDEFMVAGLDECLMKPLGSDVVLRLINQLVA

>ItbRR27

MVTASLRSSNSAAGGGGVFYIKQWRLQIAVVAAMSAALVDLLEFNLYTNGSCCYEVNLELGINAQDWGVINVFFFLGNEFLCLVRLPDMDMGSKNVGKGLRVLVVEDDTTTQMVHKMLLKKYGVEAQVAKNGEEAVELHRSGARFDLLLMDKEMPVKDGVNATRELRELGVKSMIVGVTSHGPGVVRDEFMAAGLDECLMKPLGADMVLRLINQLVA

>ItbRR28

MGSKNVGKGLRALVIVDDPATEMVHKMLLKKYGLDAQVAKNGEEAVVLHRFGARFDLLLMNKEMPVKDGFNATRELRQMGVKSMIVGITSHGPGVVRDEFIAAGLDEKWFCA

>ItbPRR1

MMEKNEIVKTGDGFIDRSKVRILLCDNDSKSSEEVFTLLCKCSYQVTSVRSPRQVIDALNAEGPDIDIILSEVDLPMSKGLKLLKYIMRDKELRRIPVIMMSSQDEVSVVVKCLKLGAADYLVKPLRTNELLNLWTHMWRRRRMLGLAEKNILNYDFDLVVSDPSDANTNSTTLFSDDTDEKSRKSINLETGPSTQQEDETNAITNAASPETLVIGSFECLPDVPGSSDRKTGKICSFPKKSELKIGESSAFFTYVKSSMPKSNDQVTVRENVTYHSRINEGGNVDIESKERANGDAIENHSQGDGYPSSNSIPDSLSMERSCTPPLSMEFPQQRMEEFSKVHMHPTNESHHDISGYHAHAHAAYPPYYIPRIMNQVMMPSSQMYQKNLPDLHNHANSAMLPTYSHVPHCPPHMPGMGSFPYYPMNMCLQPGQMPPQHPWPSYGSSSSADGKMGKIDHREAALMKFRQKRKARCFDKKIRYVNRKRLADRRPRVRGQFVRKPNGVLVDLNGHPASADDDEEDDEDEDDEDQTTTLDSSPEDDTSISLL

>ItbPRR2

MGKGKGKSIVVSGGDGGNATDLPDCSRVRVLLCDTNADSCRHVFQLLTQCSYQVALVTSRAQLFDTLRSEGPCMDIILAEIAILIANESSIMRYIKRDVRLKHVPVIMMVTIEEVSLIRKGLGFGAADYLVKPLSIHEIKDLGFHIKKN

>ItbPRR3

MEIHAKEEMHVTGDDDKELMVNSASEFWETFLHVTSIRVLVVENDDSTRRVISALLMNCNYEVIGASNGLEAWKILEDETNQIDLVLSEVVIPYLSGLDLLCKIRSHKPRSNIPVIMMSSHDLMSLVFKCLSNGAVDFLVKPVRKNELKNLWQHVWRSSHNSNGSASVGGKETKSSDDDGEDSRSSSDLKTGGGNE

>ItbPRR4

MTTISEGEKDLPDEDRKVEDGIVCEGQNASADVELKVESVSKDVNDEGRRALQAQGALQVQQQQSQSGTICWERFLHVTSIKVLLVESDDSTRHVVTALLRNCNYEVIEAANGLQAWRVLEDLTNHIDLVLTELEMPCVSGIVLLCKIMSHKTRKNVPVIMMSSRDSMGLVFKCLSKGAVDFLVKPIRKNELKNLWQHVWRRCHSSSGSGSESGTQTQNSVKSKSIEKCGNNSGSSDGEDNGSDGLNIGDGSDDGSGAQSSWTKQAAEVDSSQAVSPWDQVTECPDSTCAQVIRWSAENSGNRKVHVAATKDCQEEKQPDNTKCKYPATTIPKKLETQHENPIGAPINSVGEKHTNVVEIDPSANNNRIEKEQIDRKEFEAQKMVAAVSEIENNTMHESRKAVIEPSLKRPREMKESRETSQDDRYVFRRSEQSAFTRYNTSSQSNPLRTPNGLTGNSLVIDSGLESANNVVSNNIDMGSTTNKLATKPLTVQDKSEATCTTNGLLPSSAYKPVKNDFRNCQSLIKTSDMQATTLLAPSSSHTDIPDQHLHHHNNHYPHNSHHFHNQEQQPASNHDKFSLKQLTANALNSDSSNVMAGPFEGTLGNHSLNKSASGSNHGSNGQNGSSTAVNVGGNNGKTETGLDGKGGSGSGDASGSGSGSRMDPNKLAQREAALSKFRQKKKSRCFKNKVRYQNRKRLAEQRPRIRGQFVRQTGQNNPSNTENE

>ItbPRR5

MRGVRVDGNGPPLKGLTEINHNGMRSEQNGVRDGVNGDGHGLSEEDESRINEDAEDRNDMRRDLMQVQAVLHTQQQQPQGPVVRWERFLPLRSLKVLLVENDDSTRHVVSALLRNCSYEVTAVANGVEAWKILEDLTNHIDLVLTEVAMPYMSGIGLLSKVMNHKTRKNVPLIMMSSNDSMGVVFKCLSKGAVDFLVKPIRKNELKNLWQHVWRKCHSSSGSGSESGIRTEKSTKSKSIQGSENNSDSNDEDENGSIGLNIRDGSDNGSGTQSSWSKRAIEVESPQPMLPWNELPEPPDSTCAQVIHSRPEAQSANWVPTIATREYQDEEDEQENVPMGKDLQIGVPRSPDLQLNGPTSKALDGDASAKKGKLVNIDSSKDDEKLIGKLELNKTRKNELKDKDNGHVAAAITIKDNPLMEITGNDVPTDPSKMTNTKEIATYNSKEMPSLELSLKQHREVGETGTTVQERNVLRHSDHLSAFSRYGTTSTANQAPTGNVGSCSPVNNSSEAAKTESLQNLRSNSSSMPNQRSNGSSNNNDMGSSTNNIFVKAEAFTDKPVNKSSAVNAHPCSAFQPVQHGQNSSLPGKADSAKAALAQARAMQQQFQVQHHHHHYHHHHHHVHSMQQQQQQQQQQQQQLLNEDSLPSRKTVADAPHGSGPYMLGTLTDGNTNYGSASGSNNASNGHNGSSGQNESNAAVIAEETNMATEDGIAGKCTVGGESGSGSRSGVDQCRQAQREAALNKFRQKRKERNFEKKVRYQSRKRLAEQRPRIRGQFVSQSSDKTKTKDTNC

>ItbPRR6

MELNEAAEAERKKDDESGGSAAVFRWERFLAKMAVRVLLVEADDSTRHIISALLTKCGYKVAAVCDGLKAWEVLKKKPHNVDLILAEVDLPSISGYALLTLIMEHQICKNIPVIMMSSQDSVSTAYGCMLRGAADFLVKPIRKNELTNLWQHVWRRQALSSGAMVENNDISNGECREKGSEDQSCCSKPDIDTEREMTEHIQDLLQPNWDRSIPIVDQASDEDIESCKQGIDLIGAFDDYLNCNHINPSSNTSPNKQVDSAAPELDLSLTRTHPTSMLNQFVDNHRLNHSDGSAFTPYVNKGTQKHGGLTIPIGVRFEGASSPVISPSDSPGSGNLDSPPGLDPNPVHPIPSTADECRKEEVSLAQDGNSQRSSQREAALTKFRLKRKDRCFEKKVRYESRKKLAEQRPRVKGQFVRRQPIGTGDNEMISAVNQ

>ItbPRR7

MGEVVVSGDGGAAAMELETEEVEVVEAPASAAAASAVRWERFLPKMVLRVLLVEADDSTRQIIAALLRKCSYKVAAVPDGLKAWEVLKGRPRNVDLILTEVDLPSISGYALLTLIMEHEICKNIPVIMMSAHDSVSTVYRCMLRGAADFLVKPVRKNELRNLWQHVWRRQATSKSGQGPGDESVAQLKVEATAENNGFSNHSSGYKACIERNRECIEKGSDAQSSCTKPEMETGEENTKHIQEFGQPDWNKPRPADADMQKEEQNHDAGTKLRNPSDQVEGTGYNAATVASGEDRSSNENCCHPQVIGQTSDEDPAIMNSCKRAIDLIGTFDNHGICTYVSGSNISANNKVDSPPLLELSLTRYPSGSVNQFPDEKHKLNHSDASAFTRYVSKGVQPRDLISPKNKESETDSDKRLSVHNLDYNSDTHGPTASSHRLVPPTNFESGQAETRLPSPGQRVLSAPIPVRGVRFEGLSNAYSFMTSPMQSPGSAGHQNSPRQANTFHRLNHQTINSQQRHSVIEQNVNTVSTQTEYKQGYQSEPDRGHFSSATDQSANSSLCNGVVNCHYTGDGSNGRIPVTMIKSTAEYRNDEASVVQDANSQRSQREAALNKFRLKRKDRCYEKKVRYESRKKLAEQRPRVKGQFVRQLPSEPPPGDT

>ItbPRR8

MAETEANGGDGGAGTELEVQETEQIEVIEPKQGKNDGAEPPPPPPAAALRWERFLPKMVLRVLLVEADDSTRQIIAALLRKCSYRVAAVPDGLKAWEVLKERPSNVDLILTEVELPSISGYALLTLIMEHEICKNIPVIMMSSNDSVSMVYKCMLRGAADFLVKPVRKNELRNLWQHVWRRQAANRIDNLNGPVSPTRNEDCNEKGSDDENSCVKLEMEIGGENTEHVEESEQRFRGSSLPIHLQKQEQEDHNRVGDSADKVESLPPLDLSLTRYPSGSMNRLNHSDASAFTRYINKSVQPRNSMSPKTCNRHEDCGTDSDKHLSVHNLDGPTMKFHTLMQQARAEPGSNEIGLPIPVRGVGFKGLGNAHSSMMSSPMQSPGSAGCPDSQFQTPLFHLLNHQGVSFQQSCGLVDQNTDNDTSQSKKKEENHSEPADDQGHFSSFTDQSANSRTSVPLIKSTAECEKAPLGQDGSYQQSQREAALTKFRLKRKDRCFEKKVRYESRKKLAEQRPRVKGQFVRQQPK

>ItbPRR9

MASYHRVTYGIHVLLADHDHKFLASTVDMLKRQFYKVTVVDSANAAISVLNRKEEKFDAVIANIHSPDRQAYKLLRDAVSMDLLVIFLCDEEDAEVAVRLIEHGAFALLQKPTCQETLKNLWQHVVRERSMLRAKQMIFMEKTNRELAVINNGVVGGGGDNRGKGVMRVEENENYEMSYRGKGKRSREQSLSEATRMTTMTQGMSRVKRKTCTEWTVDLHEKFMSAVHQLGDGRCYPKEILELMNVPGLTRMQVASHLQKCRNDNWRAPEERRAPPMSSASPASGSGSRNEQRRFGTMPKLTAAAAAAAAAAGGNSQQLGSTMSPEVQSSPSITGAVTGDPSSQPPADRQYLAIGAAAFVSKLESSSPPSAAVQPAVVATAVGTFTATSGQASIVGSVNYGTGGILQALGCGSGGSLFRNKDAFTDDPNNNTTAGDDSFVTPQAPRIHRRLQSDEFFSFNDVDYEYLIQGFSDNNARQASVALQAPTHNNTSSSEFNDKAGFNPVQNQEAKAQTSEMDIAKISP

>ItbPRR10

MAAFPSFPHNFRLVDMGEFAGFRHSFPQVTDGTNSSIGSLVFPPRRMHGVHVLLVERRADFVANGISMVAEILKQFSYEVTVVESASAALLSLYHGKEKFDVLIANFYLPDKEVNVKLLEEAIKRKLLVVQISDEKDENGDEVARSAIEQGVFLYLEKPFPVDMLKYLWQHVYRERRLMNHSTHQALDISMVAETLMNGQNNIVFTDNQTATEFATDSNNVNLVPTRRRGAKFKWTEELHAKFMEAVNQLGAGNCYPKEISEMMNVPGLTREKIASHLQRCRDNKWRPVEEHGNRRRSRTMRSTSQPRRPRHKKFGLMPTVEELEANNNGIMPPQAQIIAAAPPANADISSQNGGNNNNNEFVSSMTVSTNSVTNIDAGILQAVQSSAGIIQDLRLLGEVQTHPVVADSVTNTDVGVFQTGQSSAGIIHDRPPLEEVQTHPIVADFVTNTDVGVLEIGQSSAGTSHDPYRPQLEEVQTHPVVTDSLNIDETAFVNSSPIPWQFDDGLMFDDLLNIPEGMLPRIFFGRQLNFPF

>ItbPRR11

MLCTANDLLGWKDFPKGLSVLLLDEDSNSAAKMRTKLEEMDYIVSTFQNENEALLAIASKSVEFHVAIVEVNTSNSNEVFKFLETAKDLPTILVSNVYCLNTMMKCIALGAVEFLQKPLSDDKLRNIWQHVVHKAFNAGGGKDVESLKPVKESLVSMLQLRSTKNEANTANSDETEQSTSVQENNRDTLSSVCDKYPAPSTPTLKQCVRSLDDGECRDQTNLSMEHDSVEHDGESKSVETTYCNSVSETIPVINPPVIKQERESSPEQAGKNGNSACSESKDARANANSSECGDPKKPSGVNSSTGTKANKKKVKVDWTPELHKKFVQAVEQLGLDQAIPSRILEVMKVEGLTRHNIASHLQKYRMHRRQILPKENEWKWPLSRDSTQRSCYPRKPVVAFPQYHSAPTVPAGHQFYSAWAHPGSYPGAHVWGSPYHYPGWQPTDDWNWQSNTGVYAQAWGCPVMPPHQGSYPTYPQNTSGHHRSGGAQDRYSMLENTYDIQPAEELIDKVVKEAINNPWLPLPLGLKPPSTDSVLNELSKQGISTIPPRTNGSDLR

>ItbPRR12

MVCTANDLLDWKDFPKGLRVLLLDEDTNSAAEMRSKLEEMNYIVSTFQKENEALLAISNKSEAFHVAIIEVNTRNSNEAFKFLETAKDLPTIMTSNIHCLNTMMKCIALGAVEFLQKPLSDDKLRNIWQHVVHKAFNAGEKDVSESLKPVKESIVSMLQLESRNSGADAQNSNETIRENSQEFSADSDKYPAPSTPQLKQGARSLDDCECLDQTNFLMERDSVDRDEESKSVETTCCNSGSSTNPAISPPVSLVEASIKGGCKSSPDHKSRTENSTSLQSTDAPLNVSNESAAPNKLSRVNSSSGTKVNKKKLKVDWTPELHKKFVQAVEQLGVDAAIPSRILEVMKVEGLTRHNVASHLQKYRMHRRQILPKDDAKRWPNPRDSTQRSCFPRDPILAFPPYHSPYSIPSDQYYPAWVQPGSYPSGVQMWGSPYHYPGWQSTDNWHWKPHPGAHANAWGCPVMPSPQGSYPTYPQNASGYYRADGVQNRYHMLEKSFDFQPAEEVIDKVVKEAINKPWLPLPLGLKPPSTECVLNELLKQGISTIPHKINGSHTR

>ItbPRR13

MGISKRVCLFVFNEDYICQNLVSEVLQHCSYEVLHIGRAMDALTEIGKRKHGISVVLTNMNRLKTKGAEIIQAIQEELNLRVCLILPGNMEFDDTRGLDCNVSAFIVNFSDTKDMKELWQSAFEKEKARKAAISSQVVGVETTTSENNEPSLDGEPGNDHYNRKAKELSEEQSEESGSETRKKPRLSWNPEMHQRFVEAVNKLGFDKAVPKKIVEFMNEPGLTREHVASHLQKYRMNLRKGQDSSSNFIYGHQKLTNDVTNPFYCSYPSALNLNSSFPFERNNNSVFSTLLGQSSLLNPNISTTLTQQPHMFPTNNLLGALNHIFPPQLSFDAKRVMNQNGEVGHSSGSSSVSMNQQQPTSFLGLRVVNNVLQFGESCGETHRESSSLFTADWTYSSGNNNADSVPFLETSTDQSVNHSETVSPAGTFIPQHQQASFPAAFGSSPYTEINTNDDPASIVSLLPLLGNSENLSSKQIESSSSLSVPPEVIEINSYGGEEDISALLDAADNDTPNNNPEEGLWDDDDFSDILSGFTK
