# Supplementary material for: Genome-wide identification and expression analysis of two-component system genes in sweet potato (Ipomoea batatas L.)
Source: Front Plant Sci. 2023 Jan 12;13:1091620. doi: 10.3389/fpls.2022.1091620 (PMC9878860; doi:10.3389/fpls.2022.1091620)
Supplement: Supplementary file 2 [file DataSheet_2.zip › Supplementary Dataset 4. Protein sequences of TCS members in Ipomoea nil.docx]

**Protein sequences**

>InHK1a

MAFGSNTSPVSSESLSPSITPKGSVLERIFCRMFSSGMFCTSNQSPSSRRNFNRDVEEEEFQDASTLCLSSYYSVFVVRLAIMVMLAILIGLLTLLTWHFTRVYTTRSLNTLAFGLRHELLQRPILRMWNILNSTVEIATAQVKLSEYVIKRYSKPVNQAQQAELYEVMRDVTWALFASRKALNSITISYKNGFVQAFHRDHRSNNTFYIYSDLSNYSISGTYDVSMLTSRQGWNDQSIHNNTTAIWYRETLDPLTGVRVGRKSQIPPDELINIAGISQVPDGAATWHVAVSKFSDSPLLSSALPVWDPSNESIVAVVGVTTALYSVGQFMKEIVEFHSGHIYLTSQEGWLLATSTSTPLLRNSTTRPELIMAVDSEDPVIKAGAQCLQKEYGNKFPPSNEVHIENAKLGDQMYYIDSFFLNLKRLPLVGVIIIPRKYIMGKVDERAFKTFVILISASICILFIGCVCIFILTNGVSKEMKLRAELIRQLDARRKAEASSNYKSQFLANMSHELRTPMAAVIGLLDILIYDDCLTNEQYATITQIRKCSTALLRLLNNILDISKVESGKLVLEETEFDLTRELEGLIDMFSVQCINHNVETVLDLSDEMPKLVKGDLGRVVQIFANLISNSLKFTTSGYIVLRGWCESLNDLTNSRNFFFNQKDSWSAPKVKLKRPERHERRPFKKDSKTVLWFEVDDTGCGIDPNKWESVFENFEQADPSTTRLHGGTGLGLCIVRSLVNKMGGEIKVVKKNGPGTLLRLYLLLNTPTDGAEQHSHPTLAEQTTTVLLALNGRMGRLIMSKWLEKNGLHTCEAADWNELTQMLQGVFGSKNSVQDSGCEHFSDNSSTLLIVVIDIGLLNLSTNIWKEQLNFLDKYSERAKFAWVLYHDTSNSIKSELRKRGHLMMVNRPLYKGKMIQILEAAFTKDKNLKLQSAENTAIQVNMHECHHEIDASHSCLTSPDDSDKSETGNVRPVRTFLAGEKTNKHFRNVSSSSLYATLNNYFVDITQVNPGEDDASREDDRRGNRNRSEEHSGSTRRVELSTVSSSKTANEQKSLSGLRILLAEDTPVLQRVATIMLEKMGATVVVVGDGQQAVDALKFCRNGPNGSSQEDDTSPTSPTEGFCSPPYDLILMDCQMPKMDGYEATKAIRRSEMETGTHIPIVALTAHAMSSDEAKCLEVGMDAYLTKPIDSKLMVSTILSLTKRKN

>InHK1b

MAYRANRTPSISSESSSTPTTPVGSLPERILHKMFGFGNLYRRNKSPTRRRIFRRDVEEEEEEEFQYASTLCLSSYYSVFVVRLAIMVMLAILIGLLTLLTWHFTRVYTKRSLNTLAFGLRHELLQRPILRMWNILNSTVEIATAQVKLSEFVMRRYSKAINQEQQVEQLYEAMKDVTWALFASRKALNSLTINYRNGFVQAFHRDHRSNNTFYIYSDLSNYSISGTYDVSMLSSREGWNDQSIHGNTSAIWYREPLDPLSGVRIGKQSQIQPDELINIAGISQVPDGAASWHVAVSKYSDSPLLSAALPVWDPSNKSIVAVVGVTTALYSVGQLMKEIVEFHSGHIYLTSQEGWLLATSTNTPLLVNSTTRPKLIMAIESEDPVIQAGAQCLQEEYGNKIPPGQEVHIENAKLGNQLYYIDSFFLNLRRLPMVGVIIIPRKYIMGKVDERAFKTLVILISASVCILIIGCVCIFILTNGVSKEMKLRAELISQLDARRKAEASSNYKSQFLANMSHELRTPMAAVIGLLDILICDDCLTNEQFATITQIRKCSTALLRLLNNILDLSKVESGKLVLEETEFDLSRELEGLVDMFSVQCINHNVETVLDLSDDMPKLVKGDSGRVVQIFANLLSNSLKFTSSGYIILRGWCESPNTLANSRKFSVNQKDSWSAPKVKLKPHGNHARRPSKKDNNKTVLWFEVDDTGCGIDPSKWESVFESFEQADPSTTRLHGGTGLGLCIVRTLVNKMGGEIRVVKKNGSGTLMQLCLLLNTPIDVTGQHGHLNFREQTMTVLLALNGRMGRLIMSQWLEKNGVDTCEASEWNELTQMLQRLSKTKTNSQGAGNANTSLFVIVIDIGLLDLSTNIWEEQLNFLDKYCGKAKFAWILYHDTANTIKSELRRRGHLLMVNRPLYKGKMIQILEAIVKENSLELQSAVNTTEENLHECHEIDANHSCIASPDDSDNSENGKDKAVNTFRVEERGNEHFAKASSTSQYGTLNNYFVDFTQTNLEDNASPEDQPMQAKNSSLECLGSPHPRESTVSSRNETTQQKSLAGLTILLAEDTPVLQRVATIMLEKLGAKVVVVGDGQQAVDALKFMLNSKDCRNEWSREEGSSTTTQTEGSCSLAFDLILMDCQMPKMDGYEATKAIRRSEVATGSHIPIVALTAHAMSSDQAKCLEVGMDAYLTKPIDSKLMISTILSLTKSLQA

>InHK2a

MSMNCKSHGMKGGFSSKFRLKKPRESQHGPSPRRRQFLFLWLFFVAIGFICLLISSSYGSLGRKKVEPPPHLDGDTTNFLLQHFNVSREEIHALASYFLDTDQISLLKCSGSPRYGSSLKNTITCVLKVLTSENQVYEKKCKLGEKLEAYGQCPVSDENIFRNIDSVLQQTSSSISSDHQFCEKETLKVRALGDQCKDIAFCFTKIFWWILLGIAVSWKLRWLRAESGRNEQQKLVSQQESSQQPQLLQHLQQQQAHAASRVSRKLWEKLLVTFVLSGVIGSICLFWYMNEDIMFWRKETLASMCDERARMLQDQFNVSMNHVHALAILVSTFHHGKQPSAIDQKTFEEYTERTAFERPLTSGVAYALRVLHSERENFERQHGWAIKKMESEDQTLAQEYMPGNLDRAPDKDEYAPVIFSQQTVSHIVSIDMMSGKEDRENILRARASGKGVLTSPFKLLKSNNLGVVLTFAVYNTHLAPDATPDQRINATVGYIGASYDVPSLVEKLLHQLASKHTIVVNVYDTTNTYSPIKMYGTDETETELLHVSNLDFGDPARKHEMHCRFKQKPPPPWIAIGASIGVLVITLLVGHIFHAAIARIAKFEHDYQKMMNLKHRAEAADIAKSQFLATVSHEIRTPMNGVLGMLQMLMDTNLDATQRDFAQTAHASGKDLISLINEVLDQAKIESGHLELEAVSFDLRAVLDNVLSLSSGRSHEKGIELAAYVSDQVPETVVGDPGRFRQIIVNLVGNSIKFTKNKEGHVFVTVHLADEVRCPVDVKDEVLTQSLSLVRDPTNRSFNTLSGFPVVDRWRSWQNFKKISGEERDKIKLLVSVEDTGVGISLEAQGRIFTPFMQADSSTSRTYGGTGIGLSISKHLVDLMGGEIGFFSEPGTGSTFSFTAAFSRDQRGSVEAKWQQYDPGVLDFHGLRALVIDGKRIRAEVTRYHLQRLGLNVEITSTVDHACSYLSTCSKTSEPEHLVMIFIDKDNWDTENSLKGLRPYGSTTLNGATPKLFLLATEMSSTECNQLKSDGLVDNVLIKPIRLSVLASCLQEATGFTYKRQVTMPKPSTLGNLLKDKQILVVDDNVVNRRVAEGALKKYGAIVTCVDGGKAALALLKPPHNFDACFMDLQMPEMDGFEATRQIRRLENEYRETINCGEILVDVPGKLAHWRLPILAMTADVIRASNEECMRCGMDDYVSKPFDEGQLYSALARFFGSG

>InHK2b

MNFSALNELGFSFLRPFLKICRWALKKMSWNCKNLGMKGSLSSNFRLRKLLSGGWRWRRKYLILCLIFVAIGLIVLLISLNNGLMRRKVEAPDLDEDSTNLLLEHFNVSKEHIQVLSAKNVVYQKQYELAIEKLEANGQCPVPDENTLTNLDIVVQQIPLPISHCASLATSSDHQFCEKEPVQVRALGDQCKDAAFYFTKVCWWILLGIAISWKVWWLCGESGGNDRQKQVQQQELPQQPQLLQHLQQQQAQASSRIARKWWEKLLVIFVSIGVMGSIWLFSHLNEEFTVRRKETIASMCDERARMLQDQFNVSLNHVHALAFLVSTFHHGKQPSAIDQKTFEEYADRTAFERPLTSGVAYAIKLCHSERENFEKRQGWTIKKMESEDQSLAQEYISGNLDPAPIQDEYAPVIFSQQTISHIVSIDMMSGKEDRENILRARASGKGVLTSPFKLLKSNNVGVILTFAVYNTDLPPDATPEHRINATLGYFGGAYDFPSLVEKLLHQLASKHTIVVNVYDTTNASAPIRMYGMEEADLDETDRELVHVSNLDFGDPARRHEMHCRFKQKRPPPWTAIAASIGVLVITLLLGHIFHAAINRIAKFERDYQKMMDLKHRAEAADIAKSQFLATVSHEIRTPMNGVLGMLQMLMDTNLDATQLEYAQTAHASGKDLISLINEVLDQAKIESGRLELEAVAFDLRAVLDKVLSLCSGRSHEKRIELAVYVSDQVPEVVIGDPGRFRQIITNLVGNSIKFTKEKGHVFVSVHLADEVKSPNDVKDEVLRQSLTLVQDRPNTCFNTLSGFPIVDRWRSWQNFKKLGEEKTENIKLLVTVEDAGVGIPLEAQGRIFMPFMQADSSTSRTYGGTGIGLSISKRLVELMGGEIGFFSEPGTGSTFSFTAAFARAEEGLLESKRQRYDPAVSELRGLRALVIDDKSIRAEVTRYHLQRLGLNVKIISKMDCSCSHLSTRLEASPLEHLALIFIDKDNWDDETSITLSKILKELRANSSNVVSGVIPKFVLLATNMSATNRNELRSAGLVDSVLIKPLRLSALVSCIQEATGFMNKRHITRRKPSSLGSLLKDKRILVVDDNVVNRRVAEGAIRKYGAIVSCVDSGKAALALLKPPHKFDACFMDLQMPEMDGFEATRQIRCLERKYNENMNSGEVLIEMNGKVCHWHTPILATTADVIQATNEKCLQCGMDDYISKPFDEWQLYSAVARFFESG

>InHK3

MSLLHVIGFGLKLGNLLLTLCSLVVTLISMNWLSNGGVMTTKTLLDDGEEILTKLWGKISENISKIHHSYSQYIGSKKVRKNWWGLLVIWVGFGTILAFCAFWYLSTQAMEKRKETLASMCDERARMLQDQFNVSMNHVQAMSILISTFHHGKNPSVIDQATFARYTERTAFERPLTSGVAYAVRVLHPEREQFEREQDWTIKRMDPQFHENEYNVDNLEPSPIQEEYAPAIFAQDTVAHVISVDMLSGKEDRENVLRARASGKGVLTAPFKLLKTNRLGVILTFAVYKKDLPSNATPNERIEATYGYLGGIFDIESLVEKLLQQLASKQTILVNVYDTTNLSDPISMYGTNVSIDDLEHVSSLNFGDPFRKHEMHCRFKQKPPWPWLAIITSFGIITIVLLVGHIFHATINRIAKVEDDYHEMMELKKRAEAADVAKSEFLATVSHEIRTPMNGVLGMLHMLMDTELDVTQQDYVRTAQASGKALVSLINEVLDQAKIESGKLELEAVSFDPRAILDDVLSLFSGKSQEKGVELAVYISDKIPKLLIGDPGRFRQIITNLMGNSIKFTEKGHIFVTVHLVEEVVVEHESSYALSGFSIEHESSSTLSGFLVADRRQSWKKFKAFNQEGFSSFKLTSDQINLIVSVEDTGVGIPFEAQSRIFTPFMQVGPSIARIHGGTGIGLSISKCLVHLMKGEIGFVSLPKTGSTFTFTAVFANGCFSSNELKGQHINDESNSLFSEFKGMRALVVDPRPVRAQVSKYHIQRLGIYVKIVPDLDHGYARISTEKTNINIVLVEQEVWDMDSGMATQFVEKLRNSDISCSPKLFVLANCASATRANASIFGVSTPFVIMKPLRASMLAASLQRALGVNNRGNYRNGGLSGVPLSELLHKRKILVVDDNPVNLRVANAALRKYGADVVCIDSGEQAISHLRPPHRFDACFMDIQMPKMDGFEATKRIRELECQVNSQNEHGELLVNASNWHVPILAMTADVIHATNEQCLKCGMDGYVSKPFEPEQLYREVSRFFHVKSN

>InHK4

MGQKMHHHTVAMRLGEQLSSKRKYTLIHRNRLPQLLAFWILLMFFISSVIFNSLDAMNKEKRKEALVSMCDQRARMLQDQFSVSVNHVHALAILVSTFHYYKTPSAIDKETFAEYTARTAFERPLLSGVAYAERVLNSQRGSFEDQHGWTIRTMDKEPSPIRDEYAPVILAQETVSYLKSIDMMSGEEDRENILRARATGKAVLTSPFRLLDSNHLGVVLTFPVYKSMLPANPSQQDRIEATAGYLGGAFDVESLVENLLGQLAGNQAIVVNVYDITNASDPLVMYGQPGQEGDLSLTHVSKLDFGDPFRKHEMICRYLQKAPTAWAAVTFAFFIFVIGFLVGYMIYGAGIHIIKVEDDFHKMQELKVEAEAADIAKSQFLATVSHEIRTPMNGILGMLALLLDTDLSSTQRDYAQTAQACGKALITLINEVLDRAKIEAGKLELETVLFDIRSILDDVLSLFSEKSRKKGVELAVFVSDKVPEIVFGDPGRFRQVITNLVGNSVKFTERGHVFVQVSLAEEAKAKSEACLNGGSERFIPSSGYHCETLSGYEVADNRNTWDSFKHVIPDEPLYYRAANKLMTDDASQNVTLMVSVEDTGIGIPLHAQDRVFTPFMQADSSTSRHYGGTGIGLSISKCLVELMGGQINFISRPDVGSTFSFTVNFQRYETNGSVDLKKGLSDDLPMSFKGLRAIVVDGKPVRASVTKYHLKRLGILVEVVNSIKKAVAVFGKNGSLISKSQLQPDMILVEKDVWMSEDGGGMNLQIPNLKPNGHTYKVPKMILLAVDISSAEFEKAKAAGFADTIIMKPLRASMVGACLHQVLGMGKKTQGKDACNKSTLRGLLCGKRILVVDDNRVNRRVAAGALKKFGADVECAESGAAALALLQIPHNFDACFMDIQMPEMDGFEATRRIRKMEKEANDRVNGGGLEGEGRRMWHVPILAMTADVIHATLDKCLEFGMDGYVSKPFEEENLYKAVAKFFESKPMPDV

>InHK5

MVSEMENAHTEEMDIEVLSSMWPEDINEAGKQFNIEQPGADLDMLEEVTINEETTTIVDFQRLMELTDYSDKGSSQLAYLVKNWEYKQANAVRLLREELDYLSKQQQESELKKLEILEQHRFEEERYGGDKRPVSILDEDLKYIYQDIPRRKKDVVVQAEKLEIEAEYDSIIYWKQRAVHLQKLLAASIERENILLEKLQESIEKLERQSSPVEELSQVLKRADNYLHFVLQTAPIVIGHQDKELRYRFIYNHFPSLREEDIIGKTDVEIFSGSGVKESQDFKKEVLERGLPAKREITFETELFGSKTFLIYVEPVFSKAGETIGVNYMGMDVTDQVRKREKMAKLREEIAVQKAKETELNRTIHITEETMRAKQMLATMSHEIRSPLSGVVSMTEILATTKLEKDQRQLVNVMLSSGDLVLQLINDILDLSKVESGVMKLEATKFRPREVVKHVLQTAAASLQKLLTLEGFVAEDVPTEVIGDVLRIRQILTNLISNAIKFTHEGKVGIKLYVVPEPSLGAKQGSHQKQSLDSLKSSSNNWKEDRCLSASHGKHDRTASFSYKDGEGTYENQMHKDGSNHSVSSGALDDDVDAHPDQEEKTVWICCDVYDTGIGIPENALPTLFKKYMQVGADTARKYGGTGLGLAICKQLVELMGGHLTVSSKEHHGSTFTFVLPYKVSPLCESSDENDEMSDMGSHDTSTDANEDDANSGFFQFQPRTLGSLFSHGSGRAQKLSPNNFGFNTLHSCNGLPKNSYSFPANSVMLKDMGSVEDACSVIDVDILSDPESSFRHSSHSDNPSTLERDKHADSGSNGQCRHHHSSYSTDSTSTRKDEDIKTVVQEKRQPEGNSPCSSGNNQEVSKSAPKPRILLVEDSKINVMVTQSMMKQLGHQIDVVNNGVEAVRAVQRSSYELILMDVCMPVMDGLQATRLIRSFEETGNWDAARTAGVEEVPSSSLSLKTPDSKSSNGRIPIIAMTANALSESADECFANGMDSFVSKPVTFQKLKECLQQYLPQRHRL

>InETR1

MESCNCIDPQWPADELLMKYQYISDFFIALAYFSIPVELIYFVKKSAVFPYRWVLVQFGAFIILCGATHFINLWTFGMHTRTVAIVMTTAKLLTALVSCVTALMLVHIIPDLLSVKTRELFLKNKAAELDREMGLIRTQEETGRHVRMLTHEIRSTLDRHTILKTTLVELGRTLGLEECALWMPTRTGLELQLSYTLRHQNPVGFTVPIHLPVISQVFHTNRAVKISPNSPVARLRPAGKYLPGEVVAIRVPLLHLSNFQINDWPELSTKRYALMVLMLPSDSARQWHVHELELVEVVADQVAVALSHAAILEESMRARDLLVEQNIALDLARREAETAVRARNDFLAVMNHEMRTPMHAIIALSSLLQETKLTPEQRLMVETILKSSNLLATLINDVLDLSRLEDGSLQLEIVTFNLHALFWEVHNLIKPIASVKKLSVTLSLSPDLPEYAIGDEKRLMQVLLNVVGNAVKFSKEGSVSISAFVAKSEFLRDPQAPDFFPVITENQFYLRVQVKDTGVGINPLDIPKIFSKFAQNQSLSTKNSGGSGLGLAICKRFVNLMEGHIWIESEGLGKGATAIFIVKLGIPGLSNELNPTLVPKLPANHIHTIFLGLKVLLMDDNSMSRTVTKGLLAHLGFDVTTANSGDECLRAVNHEHKVVIIDVSMTGVDGYKLANQIHEKFSKRHERPFIVGLIGTTDRVMKEKCLRAGMDGVILKPVSVEKMRNVLTELFEHGVVLEAQ

>InERS1

MESCDCVEILLPTDELLVKYQYISDFFIAFAYFSIPLELIYFVHKSAFFPYRWVLMQFGAFIVLCGATHLINLWTFTTHSKTVAIVMTIAKISTAIVSCVTALMLVHIIPDLLSVKTRELFLKTRAEELDREMGLIIKQEETGRHVRMLTHEIRSTLDRHTILRTTLVELGRTLDLAECALWMPTQRGMVLQLSHTLNNLIPVGSTVPINLGIINDIFNSSGAILIPHSCELAKMRSTNTGRHVPPEVAAVRVPLIHLSNFQINDWPELSAKSYAVMVLILPMNGIRKWREHELELVQVVADQVAVALSHAAILEESMRAHDQLMQQNIALDLARQEAEMAIHARNDFLAVMNHEMRTPMHSVIALCSLLLETDLNPEQRVMMETILKSSNLLATLINDVLDLSRLEDGSLELENVTFNLHGVFREVVNMIKPIAAVKKLSTSLSLALDVPIHAVGDAKRLTQIMLNVVGNAVKFTKEGQISIEASVAKPDYIRGSRQGEFYPPSTEGHFYLRMQVKDSGCGISPQDIPLIFTKFTEARSASNRSNSGAGLGLAICRRFVQLMGGHIWIESEGLGKGTTVTFIVKLGSCNYPNAPAIVAPRGRANQGSDDLFKYRQYHRGDGSMYAPVPRYQRSL

>InCKI1a

MGRGTNNVIGDKISLIIIIIIIIIIIIIIMSFKLIIITSARREMCVKAALIKQKEATEEAERKSMSKSVAVANASHEVRTALAGITGLIQMCRTDAIAHSKLNDNLKHMESCTNDLYSLLNSILDASKIEAGKMQVEEEEFDLQELLEDVVDLYYPLGMKKGVDVILDPCDESVEKFRRVRGDRGKLKQVLCNLLYNAIKFTDEGYVTLRVWARKPSPLPPPPSKAEVASSISSMTILKGCFFCDAVAITIGGDEEEGVNNNLVFQRKDGVMEYIFKVVDTGKGIPKEKRKTVFENYAQVKETSEHQLGHGLGLGIAQSLVWLMGGGIGIEDKEIGERGTCFRFNIFLDNIIINNSCCVHALRGKG

>InCKI1b

LLLLFIIIIIIIIIIIIIMILCLMRDMCVKAELIKQKEATEEAERKSMSKSVAVANASHEVRTALAGIAGLIQMCRANADASAAHSALNDNLKHMESCTKDLYSLLNSILDASKIEAGKMQIEEDEFDLQELLEDVVDLYYPLGIKKGVDVILDQCDESVEKFRRVRGDRGKLKQVLCNLLFNAIKFTDEGYVALRVWARKPSPLPPPPTPSLAKARRASSSSSPIITILKGCLAPFSNLFSTGGGEEKEEEEVNDNSEVLQRKDGEMEYIFKVVDTGKGIPKEKRNTVFENYAQVKETGGYQLGHGLGLGIALSLVRLMGGEIGIEDKENGERGTCFKFNIFLDNIIDNNSSSVGYASLSTSWSHVVIFMHCEERGKIIGRFLESRGVKVTVIHKGHKQLSRKLKKIKREAFLPRSSSSSSSRSREELLEDETMPLHTNTTSMVLIIIDTSAALFSELIQAVSKFHKDLQPGCSRVLWIDTALGAHKLDNDFHLPSTDLLVSKPLQGSRLHNILGLLPDFASSSQQGEIQVVIDKEKEEKDEDESAGGSEKKALALAGKRFLVVDDNSMLRKICSTVVSHLGALAFTANNGEEALQLVSKALQDHPHQPPFDYILMDCEMPIMDGFEATKRIKEEGKAMGIWIPIIALTAHTGKEEMEKVTEAGMDYYMPKPINPATLLAAIQVVDKSTHLL

>InHKL3

MSKIFALRVLVWVSLVAFTVADNGFFRCNCDYDGFWSVETIMECQKVGDFLIAVAYFSIPIELLYFVSCSNAPFKLILVEFIAFIVLCGMTHLLMGWTYYGQHSFHLMLALTIFKVLTALVSFATAITLVSLIPLLLKVKVREFMLKKKAWDLGREVGMIKKQKEAGWHVRMLTREIRKSLDRHTILYTTLIELSKTLDLHNCAIWMPNEEKTEMELTHEVRGRSFLDGRNFPIPVLDPVVQEIKQSVEVKLLDPDTPLAVASSGGVGVCEPGSVAAIRMPMLRVANFKGGTPELVPQCYAILVLVIPAGQGRCWGNQEMGIVKVVADQVAVAISHAAVLEEVQNMRDKLEEQNRALHQAQQGALRASQARNSFQMVMSNGMRRPMHSILGLLSVLQDEQLNPEQKLLRDALSKTSNVLSTLINDAMDTSTKHNGRFQLEMRSFQLHSMIKEAICLAKCLCAFKGYEFAVEVDKSLPNHVIGNEIRVFQVILHMVGNLLKNSGGGCIKFSITREGGNDLGWRKSSSEYVHIKFEIGIVGNSSQPEGVYKGSQCSETFGRREVEEVLSFTVCKKLVQLMQGNISVVPNPKGFHQSMAVVLAFHLGPSTSGMSGCTESSSYTHPSSLLPGLEVLLADHDGINRGVTRRLLEKLGCNVSAVSTGYECLGALGTQVCPFQVVLLDLHLPDLDGFEVAMRIRKFRSGSWPLIIALTANDDEDASERCIQVGMNGIIRKPVILQGIADELTRVLLLRSRSIA

>InHKL1

MGAMLRWLFLGLLVSSIFSVVSAIDYLCCDDEGLFSVSNILFMQKVGDVLIAVAYFSIPIELLYFISCSNIPFKWVLVQFIAFIVLCGLTHLLNVWTINTHPSFQIIMSLTVAKILTALVSCATAITLLTLIPLLLKFKVRELFLRQNVLELDQEVGMMKKQKEASMHVRMLTLEIRKSLDKHTILYTTLVELSKTLNLQNCAVWMPGGNRTQMNLTHELNPCSAREHHSLSVNDPDVLEITKNEGVRLLKRDSVLAAASSGGSGEPGAVAAIRMPLLRGSNFKGGTPELIDTCFAILVLVFPSVNDGDSSYDELEIVEVVADQVAVALSHATVLEESQSMQEKLKERNRLLQQAKEDAMKASQARNSFQKVMNNGMRRPMHSILGLLSILQDDNLKPEQKIVIDTLVKTSTVLSTLISDAMEISAKDDGKFPVEMRPFQLHSLIREASCLVKCFAIYKGFDFSTDVPTSLPNQVMGDEKRTFQVILHMVGHLFNVSDGNGSVIFRVASESGTEDGNNKVWNTRKPSSSDDNVTIKFEIEVTIGDSQSGTSVSVVPSGRKRHNSKDVKEGLSFTMCKKLVQLMQGNIWVSSNSRGRGQGMTLILRYQKQSSIRRRIFEYRNPSEPPLPSSMFEGLQVLLADDDGVNRMVTKKLLEKLRCQVSTVSTGFECLSALGPSATSYQVIILDLHMPEMDGFEVAMRVRKFRSRNWPLIIAVTASSEDHMWERCLQVGMNGLIRKPVLLQRLAEELQRVLQRAGNEVM

>InHKL5

MLRMSVLKLLVLSVHIAVAAADNGFARCNCEYEGFWSIENILECQKVSDFLIAVAYFSIPIELIYFISCSNVPFKLVLFEFIAFIVLCGMTHLLNGWTYYGQHSFQLMLALTVFKVLTAMVSFATAITLMSLIPLLLKVKVRELMLKKKAWDLGREVGIIKKQREAGWHVRMLTQEIRKSLDRDTILDTTLSELSKTLDLHNCAIWMPNQGRTVMNLTHEVRERNFPDVDDFSIPILDTDVQEIKCSDEVKLLEPSSPLAAASSGRSSEPGCVAAIRMPMLRVANFKGGTPELVPACYAILVLVLPSGQGRSWGNQEIEIVKVVANQVTVAISHAAVLEESQHMRDKLAEQNRELQQAQQGALRANQARNAFQTVMSNGMRRPMHSIFGLLSILQDENLNSEQHLLINAMVKTSNVISNLITDVMDCSTKDNRKFPLETRSFELHSMIKEAVCVAKCICAYKGYEFSVEVDKSLPNHVMGDERRAFQVILHVVGNLLKNSNGGCLKFHVVPERSSQGGNDLGWRSWRSNSSRENVFVRFEIGIHGNNSQPEHTTSKAVNFNQKYSGKDFEGSLSFSVCKKLVQLMQGDIWVSPNPMGFDQQVMAVVLGFQLRPSVVIGISEYGDSSNRTHSDSLFRGLNVLLADYDDVNRAVTRRMLEKLGCIVSSVSSGYECLGCLGTTISSFQIVLLDLHLPDLDGFEVTMRIRKFKSRNWPLIVALTSNNDAGIRGRCFQVGMNGVICKPLFLQGIADELQKVMLMASRTIS

>InHKL9

MASGSKSKHVQQMQNQAQSSGTSNVNYRDSVSKAVAQYTVDARLHAVFELSGESGKSFDYSQSVKTITQSVPEKQITAYLSKIQRGGHIQPFGCMIAVDEPSFRVIGYSENAREMLGLTPQSVPSLERPEILTIGTDLRTLFTPSSSVLLERAFGAREITLLNPIWIHSKNSGKPFYAILHRIDVGIVIDLEPARTEDPALSIAGAVQSQKLAVRAISHLQSLPGGDIKLLCDTVVESVRELTGYDRVMVYKFHEDEHGEVVAESKRPDLEPYIGLHYPATDIPQASRFLFKQNRVRMIVDCNATPVQVVQDESLMQPLCLVGSTLRAPHGCHAQYMANMGSIASLTLAVVINGSDEEAVGGRSSMRLWGLVVGHHTSARCIPFPLRYACEFLMQAFGLQLNMELQLASQFSEKHVLRTQTLLCDMLLRDAPSGIVTQSPSIMDLVKCDGAALYYQGKYYPLGVTPTEAQIKDIVDWLLTYHGDSTGLSTDSLADAGYPGAASLGDAVCGMAVAYITSRDFLFWFRSHTAKEIKWGGAKHHPEDKDDSQRMHPRSSFKAFLEVVKSRSLLWENAEMDAIHSLQLILRDSFKDAEASNSKAVVRAPPGELELQGMDELSSVAREMVRLIETATAPIFAVDAEGRINGWNAKIAELVGLSVEEAMGKLLIQDLVHKESQETTEKLLFNALRGEEDKNVEIKLRTFGTEEDKKAIFVVVNACSSKDYTNNIVGVCFVGQDVTGQKIVMDKFIHIQGDYKAIVHSPNPLIPPIFASDENTSCCEWNTAMEKLTGWSRGETIGKLLVGEVFGSCCRLRGPDAMTKFMIILHNAIGGQDTDRFPFSFFDRNGKYVQALLTANKRANMDGQIIGAFCFLQIASPELQQALKIQRQQENKCFSRMKELAYICQEIKNPLNGIRFTNSLLEATDLTEDQKQFLETSAACEKQMSKIIMDVDLENIEDGSLELEKEDFFLGKVIDAIVSQVMSLLRERGLQLIRDIPEEIKTLAVNGDQVRIQQVLANFLLNMARHAPVPGGWVEIQVRPSLKQVSDGTNVVHTEFRIMCPGEGLPPELVQDMFHSSRWVSQEGLGLSMCRKVLKLMNGEVQYIRESERCYFLIILELPIPRRGSKSIIIG

>InHKL2

MPEMFIVLCGLTHLLNGWTFSAQPSFQLIMSLTVVKIMTALVSCATAITLLTLFPLILKIKVREIFLRQNVLELDQEVDMMKRQKEASLHVRMLTREIRKSIDKHRILYTTLVELSKTLNLQNCAVWMPNEKGAEMNLTHELNPGAAREKCSLSINDRDVVEIKKIKGVRILRQDSVLAAASSGGTGEPGAVAAIWMPLLQVSNFKGGTPEVFSPRYAILVLVLPSTTDHSVWGNNEMEIVEVVADQVAVALSHATVLEESQSMREKLKERNHVLQRAKEDAVKASHARDSFQKVMNNGMRRPMHSILGLLSIFQDDNINPEQRIIVDTMVKASTVLSTLMSDAMEITAKHNGKFLVETRLFHLHSLITEASSIVKCMSVYKGFGFWADIPNSLPNQVMGDEKRTFQVLLHMVGHLLNVSDGKGSVIFRVVQESGTEEGNNKVWNTRKPSPADDWVTIKFEIEVSVEGSRPDSSVSTIHFGGGSHNCKDVKKGLSFNICKKLVQMMQGNIWMSSDSQGRAQSMTLILRFQKQSSYRRRVFECKNPREKQLSSPTFEGIQVLLADDDNVNRMVTKKLLGKLGCKVFAVSTGFQCLSALAPSGASFHVIILDLHMAEMDGFEVATRVRNNFRGRGSRPLIIALTASSEEHVWEKCNQVGMNGFIQKPVLLQRLSDELQRVLHSAREGPRR

>InHKL4

MTEFWVFTVNCEFGFPGMMLKLLASGLFISWFLVVLAAADNGVGCNCDDIEGVWSIESILECQKVSDFLIAVAYFSIPIELLYFISCSNIPLKLVVFEFIAFIVLCGMTHLLSGWTYYGQHPFHLMLALTVFKALTAMVSFATAITLITLIPLLLKVKVREIMLKKKARDLGREVGMIKKQKEAGWDVRMLTQEIRKSLDRHTILYTTLIELSKTLGLCNCAIWMPNEGKTGMNLTHEVRGKDFSSLYNYSIPILDPDVQEIKKSVEVKLLDPESPLAGASSGGNCEPGGVAAIRVPMLRVANFKGGTPELVPACYAILVLVIPAGQGRCWGNQEIEIVKVVADQVAVAISHAAVLEESQHMREKLVEQNRALQQAQKDALRANQARTGFQMVVSNGMRRPLHSISGLLSILQDEKLNTDQKLLVDAMAKTSNVLSNLINDVMDTSTKDNGKFPLEFRSFQLHSMVKEAACLIKCLCAFKGNDFAVEVDRSLPNRVMGDERRVFQVILHVVGNLLKISGGGCLKFLVVPEKASHGGNDFRWKTWRSNSSSENVYIRLEIGICSYKSRTEGATKSNGSREVEDGLSFSLCRKLVKLMQGEIWMVPNSKGFDRSVAILLPFQLKPSIVLDISEYGELASNHTNPYFLFEGLEVLLADYNDLNRAVTCRLLEKLGCIVSTVSSGYDCLGALGPGVSLFQVVLLELNLPDLDGFELTMRIRKLRSRGSFPLIIALTASSDEDVIGRCLQVGMNGVIRKPVLLQGIAGELQRVLLLTNRIISPRE

>InHKL10

MAGSGTGSSSKRIEHQSSSTQVPQSSGTSNSNNRYSVSKAVAQYTEDARLHAVFERACGSGKSFDYSESVKGATHFVPEQQIAAYLSNIQRGGHIQPFGCMIAVEEPSFRVIAYSENAREVLGLMPQSVPSLDRPDILAIGVDVRTLFRPSSSVLLQRAFGAQEITLLNPIWVHSKNSGKPFYAILHKIDVGIVIDLEPARSEDPALSIAGAVQSQKLAVRAISRLQSLPGGNIKHLCDVVVECVRELTGYDRVMVYKFHEDEHGEVLSESKRPDLEPYIGLHYPASDIPQASRFLFKQNRVRMIVDCHASPVRVIQDESLKQPLCLVGSTLRAPHGCHAQYMANMGSIASLTLAVIVNGNEDEGVGGRNSMRLWGLVVGHHTSARSIAFPLRYACEFLMQAFGLQLNMELQLASQLAEKHVLRTQTLLCDMLLRDSATGIVTQSPSIRDLVKCDGAALYYKGKYYPLGVTPTEDQIKDIAEWLLTYHGDSTGLSTDSLADAGYSGAASLGDAVRGMAVAYITSKDFLFWFRSHTAKEIKWGGAKHRPQDKDDGQRMHPRSSFKAFLEVVKRRSLPWENAEMDAIHSLQLILRDSFKDAEASNSKAVVHAPPGELELQGMDELSSVAREMVRLIETATTPIFAVDAEGNINGWNAKVAELVGLPVEEAMGKSLVHDLVHMESQETTEKLLFNALRGYEDRNVEIKLKTFGTMQHTKAIFVVVNACSSKDCTNKIIGVCFVGQDVTEQKVVMDKFIHIQSDYKAIVHSPNPLIPPIFASDENACCCEWNTAMEKLTGWSKGEMMGKMLIGELFGGVCRLKGPDAMMKFMITLHHAIGGKDTDKFPFYFLDRNGKYVQTLLTANKRVNMDGQIIGAFCFLQIASPELLQAIKVQRQQENKWLTKSKVMAYICQEIKNPLNGIRFTSSLLEATNLTEHQKQFLETSAACQKQMSKILRDAGLENIEDGSLELEKEEFHFGSVIDAVVSQVMLLLRERGLQFMLDIPEEMKTLKVYGDQARIQQVLADFLLKVVHHAPAPKGWVKIHVRPSLRQSSDGITIAHVEFRFVCPGEGLPSALVQDVFHNSEWETREGLGLSMCRKILTLMNGEVRYVREAERCYFQIILKLSVPTRGSKSS

>InHKL6

MSTSRPSQSSSNSARSKHSARIIAQTSIDAKLHAEFEESGDSFDYSSSVRVTSVDVGVQKPRSDKVTTAYLHQIQKAKFIQPFGCLLALDEKTFKVIAFSENASEMLTMVSHAVPSVGDHPALGIGTDIRSIFTSPSAAALQKALGFGEVSLLNPILVHCKTSGKPFYAIIHRVTGSLIIDFEPVKPYEVPMTAAGALQSYKLAAKAIARLQSLPSGSMERLCDTMVQEVFELTGYDRVMIYKFHDDDHGEVVSEITKPGLEPYLGLHYPATDIPQASRFLFMKNKVRMICDCRAKHVRVVQDEKLSIDLTLCGSTLRAPHTCHLQYMENMNSIASLVMAVVVNDGDEEGETSESGRIQKRKRLWGLIVCHNTTPRFVPFPLRYACEFLSQVFAIHVNKELELENQIVEKNILRTQTLLCDMLMRDAPLGIVSQSPNIMDLIKCDGAALLYKNKVHRLGITPTDFQLQDIVSWLSEYHMDSTGLSTDSLYDAGFQGALALGDAICGMASVRISDKDWLFWFRSHTAAEVRWGGAKQDPDKKDDSRKMHPRSSFKAFLEVVKTRSIPWKDYEMDAIHSLQLILRNSFGKEADTMVTNANANAIHTKLNDLRIDGMQELEAVTSEMVRLIETATVPILAVDVDGLVNGWNTKIAELTGLTVDEAIGKHFLTLVEDSSVHIVRNMLNLALQGKEEKNVQFEIKTHGQRSESGPISLIVNACASRDVQESVVGVCFIAQDITGQKTIMDKFTRIEGDYRAIIQNPNPLIPPIFGTDEFGWCSEWNSAMTNLSGWRRDEVIDKMLLGEVFGTQKAYCRLKNQEAFVKLGVVLNNAITGQMSEKTRFGFFARNGKYVECLLSVSKRLDREGAVTGLFCFLQLASQELQQALHFQKLSEQTAVKRLKVLAYIRRQVKNPLSGIMFSRKMLEGTELGKDQKSILRASAQCQQQLSKVLDDTDLDCIIEGYLDLEMVEFKLDEVLHASISQVMTKSNGKSLRIINDIADNILCETLYGDSLRLQQILSEFLSVAVNFTPSGGQLALSSKLTKDNLGESIQLAHLEFRLTHTGGGVPEELLTQMFGSEADASEEGISLLISRKLVKLMNGDVQYLREAGRSSFIISVELAVASKPPS

>InHKL7

MSSRSGTIRTNCSMSSSARSRHDARVVAQTSIDAKLHVEFEESEEQFDYSTSVNLSNSTSNIPSSTMSAYLQKIQRGSLIQPFGCLIAVDEQNFSVLGFSENAPEMLDLAPHAVPSIEQQEALTIGTNVRTLFRSTGAAALEKAASFEEVSLINPILVHCKNSGKPFYAILHRIDVGLVIDLEPVNPADVPVTAAGALKSYKLAAKAISKLQSLPSGDISLLCDVLVREVRDLTGYDRVMVYKFHEDEHGEVVAECRKPDLEPYLGLHYPATDIPQASRFLFMKNKVRMICDCLAPSVKVIQDKTLAQPLSLCGSALRAPHGCHAQYMANMGSIASLAMSVTINEDDDEMDSDQQKGRKLWGLVVCHHSSPRFVPFPLRYACEFLVQVFSVQINKEVELAAQRLEKHILRTQTVLCDMLLRESPVGIVTKSPNIMDLVRCDGAALYYRNKFWLLGATPTEPQIRDIAQWLLDSHSSSTGLSTDSLMEAGYPNASIVGDSVCGMAAVKITSKDFLFWFRSHTAKAIKWGGAKHDPGDKDDWRKMHPRSSFEAFLEVVKRSLPWEVVEMDAIHSLQLILRGSLQGEVVDNSKMIVNVPAVDTSIQRVDELRIVTNEMVRLIETASIPILAVDTSGCINGWNTKVAELTGLAVQQAIGVPLVDLVVSEAVSTIKNVLSLALQGKEEKNVEIKLKKFGSQENNDPVILVANACSSRDVKGNIIGVCFVGQDVTGQKLIMDKYNRIQGDYVGILRSPSAMIPPIFLMDEHGRCLEWNDAMQKLSGLKREEAIDQMILGEVFTVSSFGCKVKDSDTLTKLRILLNGVIAGQDAEELLFGFFDKQNKYVEALISANKRTDSVGRITGVLCFLHVPSPELQYAIHVQKLSEQAAANSLKKLAYVRREVRNPLNGIKCIQNLMKSSDLSKDQMQLLKTSTMCQEQLAKIIDDTDIESIEESYTEMNCCEFSLGEAIKAVVNQAMIPSREIQVQIMCDLPVEASSLYLFGDNLRIQQVLSDFLTTAVLFTPLFEESSVLFRIIARREQIGAKMHVVHLEFRITHPAPGIPEELIQEMFNYKQSMSREGLGLYISQKLIKIMNGTVQYLREAERSSFIILLEFPASLRSDHQ

>InHKL8

MDLQSKEKKKPPTSKKMENYGKAVTFSSSATSNLNTGKAIAQYNADAKLMAEFEQSRESGKSFDYSRSVIHAPQNVTEEEMTAYLSRIQRGGLIQPFGCMLAIEEPSFKIVGFSENCFDLLGLKSGVEPPERMSLIGIDARTLFTLSSRASLAKAVASREISLLNPIWVHSKINQKPFYAVLHRIDVGIVIDLEPANSADPALLLAGAVQSQKLAVRAISRLQSLPGGDIGTLCDTVVEDVQKLTGYDRVMVYKFHDDSHGEVVSEIRRSDLEPYLGLHYPATDIPQAARFLFKQNRVRMICDCNAQPVKVLQCEELKQPLCLVNSTLRSPHGCHTKYMANMGSIASLVMAVVINSSESMKLWGLVVCHHTSPRYVPFPLRYACEFLMQAFSLQLYMELQLASQLAEKKILQTQTLLCDMLLRDAPFGIVTQTPSIMDLVRCDGAALYYNGKCWLLGVTPTETQVKDIAEWLLHNHGDSTGLSTDCLSDAGYPGAPLLGDAVSGMATARITSKDFLFWFRSHTAKEVKWGGAKHHPEDKDDGGRMHPRSSFIAFLEVVKSRSLPWEDSEINAIHSLQLIMRDSLQGIGENYMKSVSSPQQNDSDGVRFYELSSMALELVRLVETATVPIFGVDSSGLINGWNAKIAELTGLQANVAIGKYLIDDVTHEDSHETFKALMCRALQGEEDRNVEVKLLKFGNHPTKEVVYLVVNACTSRDYKNDIIGVCFVGQDITPEKAVMDKFVRLQGDYEAIIQSLNPLIPPIFASDENACCSEWNAAMERLTGLVKCEVIGKRLPGEIFGGLCRLKGQDALTKFMILLYQGISGHDTEKLSFGFFDRKGNFIDVFITANKRTDERGNIIGCFCFLQTMAVDHPQISARDIEDDRECLSTLKEFAYIQQQMKNPLNGIRFTHKLLEGTVTSDHQKQFLETSEACEKQILSIIENMDSGGIVDGNRVELKTEEFVIGNVIDAVVSQVMIPLKEKNLQLLHDIPDQIKSLPIYGDQIKLQLVLSDFLLSIVRHAPSPDGWVEIRVSPGLKLIQDGNEFIHIQFRMTHPGQGLPSALIEDMVRGGTRWTTQEGVVLHLSQKLVRMMNGHVHYVREQQKCYFLIDLDFKTQKPRSRESSMDTKAE

>InHP1

MEVSQLQSSFLGYMTELFREGFLDAQFSQLQQLQDESNPTFVAEVVTLFFEDSERLLNDLNTTLTQPDVDFKKVDAHVHQLKGSSSSIGAQRVKNVCVAFRNFCEEHNIEGSLRCLQQVKQEYLLVKNKLETLFRLEQQIVAAGGAIPIFE

>InHP3

MEVVGQLQKQFVAFMTSLYREGFLDDQFLQLQKLQDQSNPDFVVEVVSLFFEDSEKLINNMANALQQQVVDFKQVDAHVHQLKGSSSSIGAQRVKNACVSFKNHCEERSLDGCVRCLQVLKNEYFLVKNKLETLIRLEHQIVAAGGTIPLLS

>InHP2a

MDVVPQLQKQFVDLIASLYHEGFLDDQFLQLQKLKDDSNPDFVAEVVSLFFEDSEKLISNLAKALQQPVVDYTQVDAHVHQFKGSSSSIGAQRVKNVCVSFRNFCNEKNLDGCVQCLQLVKNEFFVVKNKLETLFRLEQQILAAGGKIPILP

>InHP2b

MDVVPQLQKQFVDLIASLYHEGFLDDQFLQLQKLQDDRNPDFVAEVVSLFIEDSEMLISNLAKALQQPVVDYNQVEAHVHLFKGRSSSIGAQRVKNVCVSFRNFCNEKNLDGCVQCLQLVKNEFFVVKNKFETLLKLEQQILAAGGKIPILP

>InHP4a

MPSQAANLRKSLFDQGYIDDQFIQLEELQDDANPNFVEEVVRLFYNDSTRQIHNIELALGSGACDFTKLDDMMHQFKGSCSSIGAKKVNKECSEFQQYCDAGNIEGCRRAFQRLKQEYYTLKTKLETYFQMAKQGS

>InHP5

MERKHLPRQLATMRKSLFDQGYLDDQFVQLEELQDDVNPNFAEEVVTLFYRDSARLVQNIEHAMERSPLDFAKLDGLMHQFKDSCSSIGARKVKYECTQFREHCRVANAEGCKRSFLQLKKEYSTLQKKLKAYFQFARQAGPVEVACRPN

>InHP6

MLGLGAERLRVDMNRLLALLFHQGVLDEQFLQLQQLQDEASPNFVSEVVNIYFHESEKLLRNLRALLMESEIWDYKKMGMHLNQLMGSSSSIGAKRVRNVCVAFRAAAEQNNRFGCLRALEALDHEYCYLKNKLVELFQLEQQRVLAAGVRYPVLHLPHT

>InHP4b

MPSQAANLRKSLFDQGYIDDQFIRLEELQDDSNPNFLEEVVRLFYNHSTMKIHNIELALESGALDFTKLDDMMIQFKGSCSSIGARKVNKECLEFKQYCDAGNIEGCRSAFQRLKQELYILKTKLEIYFQMAKQGS

>InHP7

MDVDLLQQQLIAHIQALQREGFVDDYLQMCYGLKETSGLTFFLELIATFHTDSAAAIDDMTVTMECPILDYDKMQGLAISLKGSSACIGACRISASCSELLQAATKRSKTDCKRAVEMISREKSAWEVKLETIMQLERKIVDRQMQ

>InHP8

MDLALLQQHLIANIEALQREGYVDHYMKMCYGLKETSGITFFLELIVTFLTDAATVIDDMTATVEHPILDYDKMQELAIKLKGSSACIGACRISAGCTQLLQEIRRRSRRDCKLVVEMMIIERSTLEIKLETIMQLEREIVDRQLQ

>InRR11

MSLWPAKPAEISLSAFWDFCFFFCFFFCFWAEEMARNGVFSRRRTAEEMEDSDEVVLSSESHDVHVLAVDDSIVDRKVIEKLLKITSCKVTTVDSGRRALQILGLDEEKTSVQFDGLKVDLIITDYCMPGMTGYELLKKIKGSSFKEIPVVIMSSENVLTRIDRCLEEGAEDFLLKPVKLSDVKRLKSYMFGDDRFHGEEDGGTNKPESPPEISDDTSSSSSSPPSLSLSPSPTTSMDLSSCLSLSPSPTTSSPPSTSSPSSPKAFSSSPSPPSTNSSPPPSPVAPDSPTRILKRHDGD

>InRR12

MARNGVFSRWRRAEGPAGLSLPSESHDVHVLAVDDSLVDRKVIEKLLKITSCKVTAVDSGSRALQFLGLDKEESSVGFDGLKVDMIITDYCMPGMTGYELLKKIKGSSFREIPVVIMSSENVLARIDSRCLEEGAEDFLLKPVKLSDVKRLKSHMFGEDKDPREDSGINKRKLQEMSEDSSPPLPSPSPLLSPNPSTDLSSSSSSTSSSPSSPELLESPKTEE

>InRR10

MVMGKTEKVAAGDDCCSGGVHELHVLAVDDSHVDRKVIERLLKISACKVTAVESGSRALQYLGLDGEKGSATIDGLKVNLIMTDYSMPGMTGYELLKKIKGSSALREIPVVIMSSEKVLARIDRCLEEGAEEFLMKPVKLSDVKRLKDFVLRGDGESKEAGATTRKRKSTDESFTRPPLSLSLASSSPSIHPETTTPLSPRCSSVALPLPLSKHPRLHQDTEPLVDP

>InRR9

MGRNMRPEKIAAVDGCSSTFGGGGRELHVLAVDDSYVDRKVIEKMLKISCCKVTAVDSGSRALQYLGLDGEESSAATDGLKVNLIMTDYSMPGMTGYELLKKIKGSSALRQIPVVIMSSEKILARIDSCLEEGAKEFLMKPVKLSDVKRVVDFIVRGEEEDGKETEESTTASYSSAPDNDGLYLANIATISLDASKRT

>InRR1

MNMGMAAVEPQFHVLAVDDSLIDRKLIERLFKTSSCQVTTVDSGSKALQFLGLNEDDQKNPIQPSVSPNNHQEVQVNLVITDYCMPGMTGYDLLKKIKESSSLRNIPVVIMSSENVPSRISRCLEEGAEDFFLKPVRLSDVNKLRPHMVKNKKAGEQENQESSFSSSEESSAESGMTDVQEQAESNDNSCNKRKKGLDEGFTPKRTRPRCNGLTAFSDL

>InRR3

MGMAAAESQFHVLAVDDSIIDRKLIERLFKTSSCQVTTVDSGSKALEFLGLHEHDENNTNHPSVLSNHPQEVEVNLVITDYCMPGMTGYDLLKKIKESSYLRNIPVVIMSSENVPSRISRCLEEGAEEFFLKPVRLSDVNKLRPHMMKTKSKKPENNAPDSQEPSPEQECAVEDVKLQPQNPKPQAQTEELQPVMNSDEDTRKGMEESLSPGRTRTRQEPQGRGEEQQPVANSNENKRKAMEERVSPDRTRPRYNGLTHCCL

>InRR2

MGMAAADPQFHVLAVDDSLLDRKLIERLFKTSSCQVTAVDSGSKALEFLGLLEHGQDCPTQPSVLPNHNQEVEVNLIITDYCMPGMTGYDLLKKIKESSSLRNIPVVIMSSENVPSRINRCLEEGAEEFFLKPVRLSDVDKLKPHMMKTKGKNHQKAGTDDTQEHKETSSEESSSVESGVTDVVQSQLPQLPLEQPQSETEQHQPPPDNNNNNCNKRKTMEEGLSPDRSRTRYNGLTNL

>InRR5

MATSSRNGGDESPHVLAVDDNLVDRKLVEMLLKNSSCRVTTAENGLRALEYLGLGDEQHNTSNENENVSRQGSKVNMIITDYCMPEMTGYELLKKIKESSNMKDIPVVIMSSENIPTRINQCLEEGAQMFMLKPLKHADVKRLRGQLMQC

>InRR14

MYKFRNEKRKRQPSFVYVREERWQRASYRKSHSKSYRKSQRFSFDFVLAIYELQITIIFCFFPQQACGFSCVGEMARNGVFSRWRRAEGPAGLSLPLESHGVHVLAVDDSLVDRKVIEKLLKTTSCKVTAVDSGSRALQFLGLDKEESSVGFDGLKVDMIITDYCMPGMTGYELLKVLVSVATYTRRHLLKLDQIEWQNFSCLDGGRRGRER

>InRR16

MAAAVAVAAAATHASSESRFHVLAVDDNLVDRKLIERLLTTCSFQVTAVDSGNKALEILGLLEDSMTAMNSDHHEVEVDLIITDYCMPGMTGYDLLRKVKECRRDIPVVIMSSENEASRINMCLEEGAQEFLVKPVRQSDVNNLIKPRLFVKGNDNNNDVVSPVYCSGVDDNRHGTATATATETVISPADR

>InRR15

MARNRVFSRWRRAEGPAGLSLPSESHGVHVLAVDDSLVDRKVIEKFLKTTSCKVTAVDSGSRALQFLGLDKEESSVGFDGLKVDMIITDYCMRGMTGYKLLKALVSVATYTRRHLLKPDQIEWQNFSCLDGGRRGRER

>InRR17

MATLDDGSEFFHVLAVDDSVVDRKLIERLLKTSSENVKVTVVDSGSKALGLLSEVEVNLIITDYSMPGMTGYDLLREIKGCAAFKDIPVVIMSSEDVPSRITRCLAEGAEEFFLKPVRQSDVNRLKPHLLNGCKGKAAFSPESTAAVCGIV

>InRR8

MACPSSSMAMGENGEDEVIHVLAVDDDPINLIIIEKLLKSSSCKVTTAENGVRALEYLGLLAGDVQHNSPNTNVPKVNLIITDYSMPGMNGYELLKKVKDSAMFKDVPVVVMSSENIPSRINQCMEVGAKMFILKPLKQADVNELKSQLMHKRLSM

>InRR7

MVTVGNGEEDAVMHVLVVDDSVVDRMVVEKLFIKSFSCKVTTAENGVRALEYLGLLAGDVQHNSPNTNVPKVDLIITDYSMPGMNGYELLKKVKDSAMFKDVPVVVMSSENIPSQINQFMEAGAKMFILKPFKLADVNQLKSQLMQS

>InRR13

MARNGVFSRWRRAEGLAGLSLPSESHDVHVLAVDDSLVDKKVIEKLLKITSCKVTAVDSGSRALQFLGLDKEESSVGFDVSEFHKFVDFYFFSLS

>InRR4

MTSNRQSKVNLIITDYCMPGMTGYDLLKRVKGSSDLKEIPVVILSSENVPTRIKKCLEGGAQEFMIKPLKQSDVKKLRCHMAKFKQPCSGRLCIGR

>InRR6

MLNALLIEFSLRFSLTLWSFPFLQGSKVNMIITDYCMPEMTGYELLKKIKESSNMKDIPVVIMSSENIPTRINQCLEEGAQMFMLKPLKHADVKRLRGQLMQC

>InPRR6

MRGVRVDGNGPPLKGLTEINHSGMRSEQNGVRDGVNGDGHGLSEEDESRINEDAEDRNDMRRDLMQVQAVLHTQQQQPQGPVVRWERFLPLRSLKVLLVENDDSTRHVVSALLRNCSYEVTAVANGFEAWKILEDLTNHIDLVLTEVAMPYMSGIGLLSKVMNHKTRKNVPLIMMSSNDSMGVVFKCLSKGAVDFLVKPIRKNELKNLWQHVWRKCHSSSGSGSVSGIRTEKSTKCKSIEGSGNNSDSNDEDENGSIDLNIRDGSDNGSGTQSSWSKRAIEVESPQPMLPWDELPEPPDSTCAQVIHSRPEAQSANWVPTIATREYQDEEDEQENVPMGKDLQIGVPRTPDLQLNGPTSKALDGDASAKKGKLVNVDSSKDDEKLIGKLELNMTRKNELKDKDNGHVAAIAIKDNPLLEITANDVPTDPSKITNTKEIATYNTKEMPSLELSLKQHREVGETGTTVLERNVLRHSDHLSAFSRYGTTSTANQAQTGNVGSCSPVNNSGSEAAKTESLQNLRSNSSSMPNQRSNGSSNNNDMGSSTNNIFVKAEAFADKPINKSSAVNAHPCSAFQPVQQGQNSSLPGKGDSAKAALAQARAMQQQQYQVQHHHHHYHHHHHHVHNMQQQQQQQQQQQQQQQQLLNEDSLPSRKTVADGAGPYMLGTLTDGNTNYGSASGSNNASNGQNESNTAVIAEETNMATEDGIAGKCTVGGESGSGSRSGVDQCRQAQREAALNKFRQKRKERNFDKKVRYQSRKRLAEQRPRIRGQFVSQSSDKTKTKDTNC

>InPRR5

MEIHAKEEMHVTGDDDKEFMVDGASEDAKDVGNKRALPAEFCVREQQKRGETTIFWEMFLHVTSIRVLVVEIDDSTRRVISALLMNCNYEVIGASNGLEAWKILEDETNQIDLVLSEVVIPYLSGLDLLCKIRSHKPCSNIPVIMMSSHDSIHLVFKCLSNGAVDFLVKPVRKNELKNLWQHVWRIGSCHNSNGSVSGSGKETKSFDGDVGEDSRSSSDLKTGGGHE

>InPRR7

MVLRVLLVEADDSTRQIIAALLRKCSYRVAAVPDGLKAWEVLKERPSNVDLILTEVELPSISGYALLTLIMEHEICKNIPVIMMSSNDSVSMVYKCMLRGAADFLVKPVRKNELRNLWQHVWRRQAANRIDNLNGPVSPTRNEDCSEKGSDDQNSCVKLEMEVGGENTENVEESEERFRGVPIHPDVQKQEQEDHNQDGDGDDDEDDPAYLKSSKRAINLIGAFDNSPKCDYRSSCSKDSAEKVESLPPLDLSLTRYPPSGSPNRLNHSDASAFTRYINKVVQPRNSMAPKTSNQQDDCGTDSDKHTLGGPTMNFHALMRQARAEPGNNEIGLPIPVRGVGFKGLGNAHTSMQSPGSAGCQDSRFQTPLFHLLNHEAVSYQNTDNDTSQSEKKEENQSEPADDHGHFSSFTDQSANSSTCNGKTSVPLVKSTVECSKEKAPLGQDGSYQQSQREAALTKFRLKRKDRCFEKKVRYESRKKLAEQRPRVKGQFVRQQPK

>InPRR9

MGEVVVSGEGGAAAMELEVLETEEVEVAEVGQRKAEERDGLVRWERFLPKMVLRVLLVESDDSTRQIIAALLRKCNYRVAAVPDGLKAWEVLKGRPRNVDLILTEVDLPSISGYALLTLIMEHEICKNIPVIMMSAYDSVSTVYRCMLRGAADFLVKPVRKNELRNLWQHVWRRQATSKSGQGPGDESVAQLKVEATAENNDFSNHSSGYKACIERNRECIEKGSDAQSSCTKPEMETGEENTKHIQEFGQPDWNKPRPVDADMQKEEQHRNPSDQVEGTSNDNCCHLQVIGQASGEDPATMNSSKRAIDLIGTFDNHGICTYVSGSNISPNNKVDSPPPLELSLTRYPSGSVNQFPDEKHKLNHSDASAFTRYVSKGVQPQPQPRDLISPNTRNEHKESETDSDKRLSVHNSDPTASSHRLVPPTNFESGQAETRLPSPGQRVLSAPIPVRGVRFEGLSNAYTFMTSPMQSPGSAGHQNSPRQANTFHRLNHQTINSQQCHRVIEQNVNTVSTQTEYRQGYQSEPDRGHFSSATDQSANSSLCNGVVNCHYNGGGSNGRIPVTMIKSTAEYRNDEASAVQDANSQRSQREAALNKFRLKRKDRCYEKKVRYESRKKLAEQRPRVKGQFVRQLPSEPPPGDT

>InPRR1

MMEKNEIVKTGDGFIDRSKVRILLCDNDSKSSEEVFTLLCKCSYQVTKVRSPRQVIDALNAEGPDIDIILSEVDLPMSKGLKLLKYIMRDKELRRIPVIMMSSQDEVSVVVKCLKLGAADYLVKPLRTNELLNLWTHMWRRRRMLGLAEKNILNYDFDLVVSDPSDANTNSTTLFSDDTDEKSRKSINLETGPSTQQEDETNAITNAASPETLVIGSFEFLPDVPGSSERKTGKFCSFPKKSELKIGESSAFFTYVKSNMPKSNEQGTVRKNVTHDSRINEGGNVDIETKEHGFGDAIGNHSQGDGYPSSNSIPDSLSMERSCTPPPLSLEFPQQRMDEFSKVTNESHHDISGYHAHAAAYPPPYYIPRIMNQVMMPSSQMYHQKNLPDLHNHANPAMLPTYSHLPHCPPPHMPGMGSFPYFPMNMCLQPGQMPTQHPWASYGSSSSADGMMGKIDHREAALMKFRQKRKARCFDKKIRYVNRKRLADRRPRVRGQFVRKPNGVVVDLNGHPASGDDDEDEDDDDQTTTLVSSPEDDTSISLL

>InRR23

MTVDESREMVEKENYSDNFPVGMRVLAVDDDPISLKLLECLLRKCQYHVTVTNQARMALEMLRENKDRFDLVISDVHMPDMDGFKLLELVGLEMDLPVIMLSANSDTSLVMKGVTHGACDYLVKPVRIEELRNVWQHVIRKKKVDPKLHSKSGEEGRPGDQLSGTVEQNEKLNKRRKDEEDGSVDRCENEDPGTQKKPRVVWSVELHRKFVDVVNQLGIEKAVPKRILDLMNVEGLTRENVASHLQKYRLYLRRISLVSTPQDNMATPYMRMGSLDGFGDLPTLAGPEQLNRATLPSYAPGSMLGRLNSSAGVSLQNVNPLGLLQPSHAQGSRHSLDPLGKLNSNAPPTSQNPSLFLGIPSLELDQLRHGKFTKIEPDLNPLDNSKLLAAASTFTGSGSGFGNPINAAILQGNSGFGNPHSLNMTSLRPEPLNTGVISSSSNFLGHGGLNGNLQNSILASNVYPLMDQNHVRGNYSFVGPHLQSSPLVYPLMENSRGQTQYQEGFIGDAIQTVNQAPTQFWGDHEQNHNSNNVFSNPSSQILGTGLMPSLTQIADQNNDVFNMKTNTSLIGQENGGSVVLFQPNVNENFSQDSRMGSNGDYMLNSAKAQGAYASLDDLMNGVINEEQNGQFGFNDYSFGP

>InPRR3

MTTISEGEKELPDENRRVEDGIVCEGQNASADVELKVESVIKDVNDEGRRALQAQGALQVQQQQSQSGTIRWERFLHVTSIKVLLVESDDSTRHVVTALLRNCNYEVIEAANGLQAWRVLEDLTNHIDLVLTELEMPCVSGIGLLCKIMSHKTCKTIPVIMMSSRDSMGLVFKCLSKGAVDFLVKPIRKNELKNMWQHVWRRCHSSSGSGSESGTQTPNSVKSKSIEKCDNNSGSSDGEDNGSHGLNIGDSSDDGSGVQSSWTKQAAEVDSSKAVSPWDQVTECPDSTCAQVIRSDAENSGNRKVHVAATKDCQEEKQTDNTKCKYPATSIPKRLETHVNPIGAPINSVGEKHKNVVEIDPSASNNRIEKEQIDRKEFEAQKMVAAVSEIENNTIHESRKAVTEPSLKRLREMKESRETSQDDRYVFRRSEQSAFTRYNTSSKSNPLRTPNGLTGNSLVIDSGLESANNVVSNNIDMGSTTNKLATKPLTVQDKSEATCTTNGLHPSSAYKPVKNDFRNCQSLIKTSDMQATTLLAPSSSHTDIPDQHLHHHNNHYPHNSHHFHNQEQQPPSNHDKFSLKQLTANALNSDSSNVMAGPFEGKLGNHSLNKSASGSNHGSNGQNGSSTAVNVGGNNGKTETGLDGKGGSGNGSGSGSRMDPNKLAQREAALSKFRQKKKSRCFKNKVRYQNRKRLAEQRPRIRGQFVRQTGQNNPSNNENE

>InRR27

MDSSAANAMKILVVDDDSTSLAVVAALLRKLNFQVVTVKDPREALIILGGVEAAGGFDVVIADVHMPEMNGFELQHQITTHFQLPVILMSVDDRDTMVGRGVDFGATLFISKPVSVNDVRKYLCQIAAAAPKKDDQNTNIIPPLPPTDDPAANTNNNNNNNYEITREGEVTFKTNKLTGKKKLVWTARLHYKFLDAITLIGLNNAVPKKILEVMDVPGITREHVASHLQKYRMFLKRVTELTVPSQRNNVDWGFGSGVNLGPRIAAPRRYGGMLSLINNLQTSLGGGGRWNNNITAEEQPPIPMPPAGTFSSSSSSSFLFSGPPRLAAASPLCNQPSWRPGYGTGGRQSSLLLSPHHHQQQQRFNFGLQSSPSLIPARTNMVNPIYQRSFTLLPPLVIRGNINDAIAAASPPVPALQFQGGGGSSMLQPPQTYNNINNNNAAAETTRYRNIEIMVRGAAAAATRPNQLSGRVVEEVPPNPLDFGFLFDINDDDVFKFLLSDSTENQSDDASQNSRPNTCTEGGGNRPPPLFVPVDHDPTVQPQTDLTPPPSHPLHDDAIGTNTATGGDENSNLSNSSAVTTTQYYDDHEDLPSLDQLLLEPRNDEELFLQSVYASMPSVGAAQQHHTSNN

>InPRR10

MGEVGSEGGAGVELNDESGGSAAVFRWERFVAKMAVRVLLVEADDSTRHIISALLRKCGYKVAAVSDGLKAWEVLNKKPHNVDLILAEVDLPSISGYALLTLIMEHEVCKNIPVIMMCSQDSVSTAYGCMLRGATDFLVKPIRKNELTNLWQHVWRRQASSSGAMAENNDISNGECREKGSEDQSSCSKPDIDTEGEITEQIQDLLQPNWDRSLPIVDQASDEDIKSCEQGIDLIGAFDDYLNCNHINPSSNTSPNKVDSAAPELDLSLTRTHPSSMLNQFVDKHRLNHSDGSAFTPYVNKGTQKHQGLTIPIAERGVRFEGVSSPVISPSDSPPGSGNVDSPPLEPNPVHPSTAECRKEEVSPGQDGNSHRSSQREAALTKFRLKRKDRCYEKKVRYESRKKLAEQRPRVKGQFVRRQATGTGAPPGDNVMISTVNQ

>InPRR8

MKVAAVPDGLKAWEVLKERPSNVDLILTEVELPSISGYALLTLIMEHEICKNIPVIMMSSNDSVSMVYKCMLRGAADFLVKPVRKNELRNLWQHVWRRQAANRIDNLNGPVSPTRNEDCSEKGSDDQNSCVKLEMEVGGENTENVEESEERFRGVPIHPDVQKQEQEDHNQDGDGDDDEDDPAYLKSSKRAINLIGAFDNSPKCDYRSSCSKDSAEKVESLPPLDLSLTRYPPSGSPNRLNHSDASAFTRYINKVVQPRNSMAPKTSNQQDDCGTDSDKHTLGGPTMNFHALMRQARAEPGNNEIGLPIPVRGVGFKGLGNAHTSMQSPGSAGCQDSRFQTPLFHLLNHEAVSYQNTDNDTSQSEKKEENQSEPADDHGHFSSFTDQSANSSTCNGKTSVPLVKSTVECSKEKAPLGQDGSYQQSQREAALTKFRLKRKDRCFEKKVRYESRKKLAEQRPRVKGQFVRQQPK

>InRR19

MSVHSSTASWKSVDAVSDQFPAGLRVLVVDDDPTCLRILEKMLRTCHYEVTTCNMAEVALSLLRENKNGFDIVLSDVHMPDMDGFKLLEYIGLEMDLPVIMMSADDSKNVVMKGVTHGAYDYLIKPVRIEALKNIWQHVVRKRKHEWKDKDIEQSGSVEDGDRQQKPSEDVDYSSSANEGNWKSSKKRKDEEDEGEERDETSALKKPRVVWSVELHQQFVAAVNQLGIDKAVPKKILELMNVPGLTRENVASHLQKYRLYLRRLSGVSQHQSGLNSSFMGPPDPPFGTMSSLNGLDFQTLAATGQISAQSLASLQAAALGRSATKPAISMPLVDQRNLFSFENPKFRYVEGQQQLNNNNKRIGLLHGIPTTMEPKQLTNLQNQSSQAFGSRGMQPHQGHQNNSLLIQAQMLNEPNGSQVSRVTQPILSNGMSSELLARNGIVDNSRGAIYQPVSQADFSVNHNTEMQNNSFVSGNSGMSSCLASKRMMIPEEVNSDVKRPRGGGGFAPPSYDIFNDLQQHKAQDDWGVGAVFEASRLPNAQGSLDASPSVMVQQGFSSNGRVSIGKAVFPSGQESGNPMVGPQLNSLLGGNSITIKAERLPDTSYQNTLFPVDQHGQDDLMSALLKQQESVGAVENEFSFDGYQLGNLPV

>InRR24

MTVEEIRGHMGSEKGNHDNFPVGMRVLAVDDDPICLKLLEGLLRKCQYHVTISSQARMALKMLRDNRDRFDLVISDVHMPDMDGFKLLELVGLEMDLPVIMLSANSDPKLVMKGITHGACDYLVKPVRIEELRNIWQHVIRRKKLEPKSQNKSGDQDRSPHGGGEGGQGAPLSGSTDQNGKLNKKRKDEEDESDENGHENEDPSTQKKPRVVWSIELHRKFVAAVNQLGIEKAVPKRILDLMNVDGLTRENVASHLQKYRLYLKRISSVATQQANMVAAFGGKDSAYMRMGSLDGLGDFRTLAGSGRFSHASLSSYTPGGMLGRLNSAAGVSIRNLTSPSLIQPSHGQNLGKPLGTLGKLTPNVSQNACLFQGIPSSLELDQFQQSDGIRDLNPLDDSTLLGAANTFTDPGSGIGSSSNPMMLHGNSQLAAGGIGNQHSLNMASLNSEHFNIGVGGSSNFLDHGRASDNWQNPIQVTNFQSSSLPLTETFNQGQMQQNCARENNSSIAPHLQGGCAGYSSLSSTATPFEDSRGEIQRRERLVVGDAIPSINHQWGELKQNPNSNGVYSNLTAQVPASSIVPPLSQSMDLCNDTGNRRMDASGQSNLGGSSVLLQHNKNEKLSTESRARYYEDYLFEPPKPQGAFPSQGYGSLDDLMSSVIKREQDGATLEGEFGFDAYSFGPCI

>InRR18

MNLGGGQVGKGMSATCSNASWKSGDAVSDKFPAGLRVLVVDDDPTCLRILEKMLRTCLYEVTKSNRAELALSLLRENKNGFDIVISDVHMPDMDGFKLLEHVGLEMDLPVIMMSADDSKNVVMKGVTHGACDYLIKPVRIEALKNIWQHVVRKRKHEWKEKDPEQSGSAEEGGDRPQKPSDDADYSSSVNEGNWKSSKKRKDEEDEGEERDDSSTLKKPRVVWSVELHQQFVAAVNQLGIDKAVPKKILELMNVPGLTRENVASHLQKYRLYLRRLSGQGGLGNSFMGHPESPFGSMSSLNGLDLQALAASGQISAQSLATFQAAALGSSVTKSAISMPLVDQRNLFSFENPKSRFGDGPPQMGNSSKQIGLLHGIPTTMEPKQLASLHQSSPTFGGMSMQLNSQVHQNNPLLMQMAQTQPRAQMVNDPNGGQASRLPLSVPQPLLTGGVLGGNSIVDNSVSHAPSTVAFSVNQGTELQTNSYTTSNSGVSSLTSRGMLREQANPDVKGTRGFVPGYDIFNDLHQHKAQDWGLQNVGTTFDAPPHHFLDAPPSVMAQHGFSSNQKRNAPINKDVFLSGEETGHGGNNPMLGPQFNSLLGGGNPVTIKTERLPDTTFQNTLFSDQYGQDDLMSALLKQQQDNSLGPVENEFGFDGYQLDNLPVLHPTMLIHSRTNIPLDPK

>InPRR4

MTQSLWFYSVRLSSYMKLVIEAANGLQAWRVLEDLTNHIDLVLTELEMPCVSGIGLLCKIMSHKTCKTIPVIMMSSRDSMGLVFKCLSKGAVDFLVKPIRKNELKNMWQHVWRRCHSSSGSGSESGTQTPNSVKSKSIEKCDNNSGSSDGEDNGSHGLNIGDSSDDGSGVQSSWTKQAAEVDSSKAVSPWDQVTECPDSTCAQVIRSDAENSGNRKVHVAATKDCQEEKQTDNTKCKYPATSIPKRLETHVNPIGAPINSVGEKHKNVVEIDPSASNNRIEKEQIDRKEFEAQKMVAAVSEIENNTIHESRKAVTEPSLKRLREMKESRETSQDDRYVFRRSEQSAFTRYNTSSKSNPLRTPNGLTGNSLVIDSGLESANNVVSNNIDMGSTTNKLATKPLTVQDKSEATCTTNGLHPSSAYKPVKNDFRNCQSLIKTSDMQATTLLAPSSSHTDIPDQHLHHHNNHYPHNSHHFHNQEQQPPSNHDKFSLKQLTANALNSDSSNVMAGPFEGKLGNHSLNKSASGSNHGSNGQNGSSTAVNVGGNNGKTETGLDGKGGSGNGSGSGSRMDPNKLAQREAALSKFRQKKKSRCFKNKVRYQNRKRLAEQRPRIRGQFVRQTGQNNPSNNENE

>InRR20

MAAAVVPEQFPVGLRVLVVDDDLLCLRILEQMLRKCKYNVTICSQATAALNLLRERRGCYDIVISDVHMPDMDGFKLLEHVGLEMDLPVIMMSADGRTNLVMKGIRHGACDYLIKPIRDEELKNIWQHVVRKKCNLSKENDHSGSFEDNDQPKQGGDDAEYASSVIEGADGVLKTVTKKRDFKDDDDDDDDDDEIENDDPATAKKPRVVWSVELHQQFVSAVNQLGIDKAVPKRILELMNVPGLTRENVASHLQKFRLYLKRLSGVAQQQGGLPNSFCGPIEPNPNLGSLGRYEIQALAASCQIAPQTLAAIHAEFLGRPTSGLVLPTIDHPALLQASLPGTKYILDDQAVAYGQPLMKCPPNISKQFTQHLSTEDIPLGVGAWPPKNVCVVPSVNLSGLGAQNGNMLTTMMQHHQQQQKQQQMEQHQKLSTIPESCRPVNVRPSCLVVPSLSSANFQVTNSPASISQTSSFSKSNVMDSRILSPQSGSLSPSVSSCSTIADNSASWQVQNSACIIGASRHVAGVVPNITGIPVPDNHKSNQLLDQGPIRNLGFASRGSSIPSRFAIDESESPPISNIYHSRIYKESNTCKVKQEPDVNIADNAKVSVQMLQRIPPNDFTSVFQ

>InRR26

MNKLFSNPLFPAGVSVLLVDDNATCLRILEALLLACHYKVVKCGGAIDALRILREGKEEIDIVLSELHMSRVNGFKLLDRIIGLQIDLPVVMISSEERVDAIKKIVIQGACGYLLKPVRKEEIKLLWQHVVRHKQQGNWGKGIRPPPLPPAAAAAGEMPPQQKSVENCESSNNNRDEDTNATATTADVKKLRFVWTPQLHQQFVDVVNQIGLRNAVPKKILDLMNVPHLTRANVASHLQKYRIQLQRDGDQKSHKKLSIHHFHYEDMVLNNEQQAVTPTVFDHLGGGDYNYVNVYNYGGLCRRVRGSPTIDNILNTTTTTVDAVILQSQPYTGNNYPNQLGSASSMLRQQDHTQYNGEEGDFGVSLYDFSGTDSN

>InRR25

MTVEETRRNMGVDRENYHNFPVGMRVLAVDDDPICLKLLEGLLRKCQYHVTTTSQARMALKMLRENKDRFDLVISDVHMPDMDGFKLLELVGLEMDLPVIMLSANSDTKLVMKGVTHGACDYLVKPVRIEELRNIWQHVIRKSKGQNKFDDQDNGYRGGQGFPLTGSGEQNALLNKKRKDEEDETNENEDPSTQKKPRVVWSIELHRKFVAAVNQLGIEKAVPKRILELMNVEGLSRENVASHLQKYRLYLKRISSVATQQANMVAALRGKDSAFMRMASLDGLGDFQSLGGPGRFNHTTLSTYTPADMLGRLSSATGISIRNLSAHNHAQNLENSLGSDGNLNPNISLSSHNAASLFQGIPSPLAVQELNPLENSRALTAFSDSGSVIGSSTNPIMLQGSPKQELIGGGFGNQHSLNMASLSSELYNTGVNSSSNFLGHGRCSENWQASIQVSEFQSGSYPLTETFSHSQLPQNCEREHDLHSSPVACTTFEDSREAQVPRQQNQNSNDIFRNLSSHVPGNGMTPSLSQGSLVLFHQNGNEMATPNPRTRSNEDNLLESTKTHGAFVSQGFDALDDLMNAVIKQEEDNRGTLDGEFGFGAYPFGSCI

>InRR22

MYVTGPKMTVEQRNDKQNDEFPVGMRVLAVDDNPTCLMVLENLLRKCQYHVTATNQAIRALELLRENKNEFDLVISDVDMPDMDGFKLLELVGLEMDLPVIMLSAYGDTNLVMKGITHGACDYLLKPVRIEELKNIWQHVLRRKKFEQKISNKPDGELGRGFRGMGKTDRNGKPTRKRKDQSDDEDEELDENSGRSEDPSAQKKPRVVWSVELHQKFVAAVNYLGIDKAVPKRILELMNVEKLTRENVASHLQKYRIFLKRLDSVASQHANMVSVLGSADPSYLRMGSLNNIGNIPFNPLRSISSGSVLTRLNSPSGLGMCGFAPSSMIQLANAPNSSSSITSEINFQQSIQPGNRDMDILEGMPLPLGTDNLGVTHLYPFSNGTRMPERKIDGDGRRNLNIGVSDNSIILRSRGQCVQRKDFLDNHFPVIASPMNTERCNDNWPTATQSSLLEANSFGTGVYSHHAMPGDLGNNAMSSNLHNPLNSVCPQVPDTRTEMQCLTTIIDNVSGVKMNFSPRQDWDDFEPDSAHVPSLVCSSSANTFLPPDGGQRQQQQQHDEFENAAVDMKQEYLEEQ

>InPRR11

MWRLPEYQINNPYVNIPMASHHRVTNGIHVLLSDHDHKFLASTVEMLKRHFYKVTVVDCASDALSVLSQKEQKFDAVIANINSPDMQGFKLLRQAVSMDLIVILLCDEEEAEMAVRLIEHGAFLLLQKPMCQETLKNLWQHVVRERNLQRGKEMMLMEKNNRELAVINNGGGGADRGKGVRVEENENYEMSYRGKGKRTRQQQSLNESTQMMMNPGLARVKRKTCTEWTVELHEKFMNAVHQLGDGRCYPKEILELMNVPGLTRMQVASHLQKCRNDNWRAPEERRAPPTSSASLASESASRNEQRRFGTMPRLTAAAAAAAAAAAAAAGNSQTERQQLESTTSPEVQSSQSNGSGRGDSSQTQIHRQYLTIGDVSKLESSSPPSIVRPVAASTSAAAAAIAAAVATFDATSGQASIVGSLNYGSGGGLQGLGGGSGGGGGSLFGNKDNFSDNPSNTTTGDDSYATPPPPRLRTLQSEEFFTFDVDYEYLINGFPDNNARQGGVGLQAPNHNNTSFNIGQRQEANKAQTSEMMNVAKMSP

>InPRR12

MNDTQQGRLTSSGIPIHSSSSIPFHSPSSPSSDMGRICLLVFTDDFVCRNLVSEVLQHCSYEVLHIGRATDALGEIGKRRNGISFVLTNLNRLKTNGAEIIQTIEKELNLGVCLILPPSMEFDTKGQECNVSSYILNFSDIREMKALWQSAFEKEKVRKPSAIRSPVMGVEIRIENNNEPSVDGEPGNYNDHHNRRAKEVREKPNEESGGEKKKKPRLSWNPEMHQRFEQAINKLGIDKAVPKKIVELMNEPGLTREHVASHLQLDLDETFIGKSLEGSE

>InPRR14

MVCTANDLLDWKDFPKGLRVLLLDEDTNSAAEMRSKLEEMNYIVSTFQKENEALLAISNKSEAFHVAIVEVNTGNSDEAFKFLETAKDLPTIMTSNIHCLNTMMKCIALGAVEFLQKPLSDDKLRNIWQHVVHKAFNAGEKDVSESLKPVKESIVSMLQLESRNSGADAQNSSETIRENSQEFSSDSDKYPAPSTPQLKQGARSLDDCECLDQTNFLMERDSVERDEESKSVETTCCNSGSSTNPAISPPVLLVEASSIKGECKSSPDHKSRTDAPVNVSNESAAPNKPSRVNSSSGTKANKKKLKVDWTPELHKKFVKAVEQLGVDAAIPSRILEVMKVEGLTRHNVASHLQKYRMHRRQILPKDDAKRWPNPRDSAQRSCFPRDPILAFPPYHSPYSIPTDQYYPAWVQPGSYSSGVQMWGSPYHYPGWQSTDNWHWKPHPGAHANAWGCPVMPSPQGSYPTYPQHCRMHLDITGLMECRTDTTCWKSQLIFSLQRR

>InPRR15

MLCTANDLLGWKDFPKGLSVLLLDEDSNSAAKMKTKLEEMDYIVSTFQNENEALLAIASKSVEFHVAIVEVNISNSNEVFKFLEIVKDLPTILVSNVYCLNTMMKCIALGAVEFLQKPLSDDKLRNIWQHVVHKAFNAGGKDVESLKPVKESLVSMLQLRSTKNEEANTVNSDETEQSTSVQENNRDTLSVCDKYPAPSTPQLKQCGRSLDDGECHDQTNLSMEQDSVEHDGESKSVETTYCNSVSETIPAINPPVIKQERESSPEQAGKNGNSTCSESKDVRANANSECRDPKKPSGVNSSTGTKANKKKVKVDWTPELHKKFVQAVEQLGLDQAIPSRILEMMKVEGLTRHNIASHLQKYRMHRRQILPKEDEWRWPLSRDSTQRSCYPRKPVMAIPQYHSAPPVVPAGQFYSAWAHPGSYPGAHVWGSPYHYPGWQPTDDWNWKSNTGVYAQAWGCPVMPPPQGSYPTYPQILQNTSGHHRSGGVQDRYNMLEKTYDFQPAEELIDKVVKEAINNPWLPLPLGLKPPSTDSVLNELSKQGISTIPPQTNGSDLR

>InRR28

MTEKICSHYQKMCIGLLEMDMDSKNVGKGLTALVVEDSPILQLVHKTLLKKYGVEVQVVNNGEEAVVLHRSGARFDLLLMDKEMPIKDGVNATKELREMGVKSMIVGATSHGPGPVRDEFLAAGLDECLVKPLRAEVVLGVINKLVA

>InRR29

MDSKNVGKGLKALVVEDSPVLQLVHKALLKKYGVEVQVVNNGEEAVVLHRSGARFDLLLMDKEMPIKDGVNATKELRELGVKSMIVGATSHGPGPVRDEFLAAGLDECLVKPLGAEAILRVINKLVA

>InPRR13

MGEIGGFCHSFPQVVTDDFQPPRLMHDVHVLLVEHRVNFVVNAKELGTSMIAEILKQFSYEVTVVESASCALSRLYNGTEKFDVLMVNFYLPEISDEEDENGDEVARSAIEQGVFLYLEKPFPVDMLKYLWQHVYRERRLMNHSSQALDISMVAETLINGENNIVFTDNQTATDSNNINHLPRRRGPKFKWTEELHAKFLDAVNQLGAGNCFPKEIWEMMKVPGLTREKVASHLQRCRDNKWRPLEEHRNRRRSRTMQFSSQPRRPRGQKFGFMPSVEETNNNGNIPPQEKNIAAANDGTSSQNGDHNNEFDSSLTMSTNSVTNTDVGILQTVQISEGTIHDRSLCCEFVANPLAGHRRGDV

>InRR21

MESVMAGGIFLPRSSETFPAGLRVLVVDDDPTWLKILEKMLKKCSYEVTTCGLATEAIGLLRERRNGFDIVISDVNMPDMDGFKLLELVGLEMDLPVIMMSVDGETSRVMKGVQHGACDYLLKPIRMKELRNIWQHVVRKRMQESRDIDNHESDQFDEAWMLNGIELQSGKKRKDFDYKFDERETSDSKSGDPSSAKKPRVVWTVELHQKFVKAVNQIGFDKVGPKKILDLMGVPWLTRENVASHLQKYRLYLTRLQKENELKASSSGTKHPDLSPKESSSSACLQNLVDVKPSKSTNGKYSFPGENFFVQEVESRNYEGEVKAAASLSTAGVSDSQKSISCSKASCVPVDSVDLEHAGEIPIQYCWASEVSKTGFKHEFKPPQIQTEDNVNHLPSPKLPQHVQLDQAQPLLNLAPHKDINPGEIKSKPTKTPGVRTVSPLECAVDLLPAQPQSCLTNFQAFEQIPSTTWSMKIPESIEGNLFLGGGSWDKDFNAAALQGEFHSPCVVGPQSLELLDYSNTNLTGEIQPYLYDYEYAIDPVIDHGLFIL

>InPRR2

MGKGKGKSIMVSGGDGGHGNNTTHLPADSSTVRILLCDTNADSCRHVFHLLTQCSYQVALVTSRAQLFDTLRSEGSCMDIILVEIAILIANESSIMRYIKRDVRLKHVPVIMMVTNEEVSLIRKGLGFGAADYLVKPLNIHEIKDLGFHIKKN
