# Supplementary material for: Genome-wide identification and expression analysis of two-component system genes in sweet potato (Ipomoea batatas L.)
Source: Front Plant Sci. 2023 Jan 12;13:1091620. doi: 10.3389/fpls.2022.1091620 (PMC9878860; doi:10.3389/fpls.2022.1091620)
Supplement: Supplementary file 2 [file DataSheet_2.zip › Supplementary Dataset 1. DNA, CDS, protein sequences and promoter regions of TCS members in Ipomoea batatas.docx]

**Genomic DNA sequences**

>IbHK1a

ATGGCTGTTGGATCCAATACAAGTCCTGTCAGCTCTGAATCCTTATCGCCTTCTATCACACCAAAGGGATCATTCCTTGAGAGAATTTTGTGCAGGATGTTTAGCTCTGGGATGTTTTGCACAAGCAACCAATCTCCTAGTAGCCGGAGAAATTTCAGTAGGGATGTAGAAGAGGAAGAATTTCAGGATGCAAGTACCCTTTGTTTATCTTCGTACTATAGCGTTTTTGTGGTCCGCCTTGCTATCATGGTATGTGAAAGATGATGTTTCTCAAGTCTGCACTCATGTTTAAATGTGATTTCATAGTACATTCTTGTGTCTGCACATCTTATCTTATCCATCTTCCTCTGAAAACTTGCAGGTACATGTACTCCATTTTCCATCTTTGGAAATTAGAAAATGTCATTCTCAATTGCCAATCTCATAGTGTGAACTTATGTATTATTCTCTTTTTTTGGTAAATTGGTTTTGCAGGTCATGCTAGCAATTTTGATTGGATTGCTAACCTTACTAACATGGCATTTTACCAGAGTTTACACAACCAGGTCATTGAACACATTGGCATTTGGGCTTCGACATGAGCTACTTCAAAGGCCTATTTTGCGAATGTGGAACATCCTCAATTCTACTGTTGAAATAGCAACTGCTCAGGTTAAATTGTCAGAATATGTAATCAAACGATATAGCAAGCCTGTAAATCAAGCACAGCAAGCTGAGGTTGTTGCACCTTCTTTTCCTTGCCTAATTTTTTTTTTAATACTTCAGATTAGTTAGGTAAGATATTTCAAGCTTAATGTCATTGATTTTCTTTCAAATCTCCACTCTGAACAGCTATATGAAGTCATGAGGGATGTAACATGGGCATTGTTTGCCAGCCGGAAGGCTCTGAATTCGATAACAATCAGTTACAAAAATGGTTTTGTCCAGGCTTTCCACAGAGACCACAGAAGTAACAATACATTCTACATATACTCTGATCTTTCCAATTATTCAATAAGTGGAACATACGATGTCAGTATGTTGACATCTCGTCAAGGGTGGAACGACCAATCTATACACAACAACACGACAGCAATTTGGTACAGAGAAACTCTGGATCCTCTGACAGGTGTCAGGGTTGGAAGAAAAAGTCAAATTCCACCAGATGAGTTAATCAATATTGCAGGAATTTCGCAAGTACCTGATGGTGCAGCGACATGGCATGTAGCGGTGAGCAAGTTCAGTGATTCGCCGCTGCTTTCTTCTGCACTTCCGGTTTGGGATGCATCCAATGAAAGCATAGTTGCTGTTGTGGGAGTTACTACAGCTCTTTATAGTGTTGGTCAATTCATGAAAGAAATTGTGGAATTCCATAGTGGACATATTTATTTAACATCTCAAGAGGGTTGGTTACTTGCTACTTCCACAAGTACTCCTCTCTTGAGGAACTCAACCACACGGCCCGAGCTGATAATGGCTGTTGATTCTGAAGACCCCGTAATAAAAGCTGGTGCTCAGTGCTTGCAGAAAGAATATGGGAACAAGTTTCCCCCTAGTAATGAAGTTCATATAGAGAATGCCAAGCTTGGAGATCAGATGTACTATATTGACTCTTTTTTCCTGAACTTAAAGAGACTTCCTATGGTAAATCCAGACTCCAAAGCATTTATAATTTGCAGTTTAGACAGCTAAATAAATTTTAATAAGCAATTCTTTGCAGGTGGGAGTTATAATTATTCCAAGAAAGTATATAATGGGAAAGGTTGATGAGAGAGCTTTCAAAACATTTGTGATATTGATATCTGCATCTATATGCATCCTATTCATTGGGTGTGTCTGCATATTCATATTAACAAATGGAGTATCAAAGGAAATGAAACTGAGAGCAGAATTGATAAGACAGTTAGATGCAAGAAGGAAGGCAGAGGCATCAAGCAACTACAAAAGCCAGTTTTTAGCAAACATGAGGTAAGCATCAAAGTCAAGGTCGTTTAACAATCTAATAGGATAAATTATATGTGGATAGTTATGGTCCTGGATTGTGTTTCCTGACTTGACACCCATAACATGAGAACTTCCATTAAAAGATTGAAACATAATGCTACTTCTGTAAGTCTGAGGCAGTTGTTCTTAGTTGGAGATTTGTTTTCGTATTTCAAATTTATGATTCCATTTGCTGGTAACTTATGGTGGTCATAAATCAAAATAAGGAGATCTATTATTGAAGAAATCCATTTAAGCAATTTCATAGTTATAATCTGAACTTCCTTTTGCTTCATCTGTATGCATTATGTATCTTTGTGTGTCGTAGAGATATAACAATGTTCTATGATTCGTTCTTTATACTAGTCTACTTCAGTGACTCTTAACACAGAAGTATTGCTTTCTTTGATATCAGTCTTCCCTTTTCAGCTAAAACTTGTATCTTCAATGATCTTAAAGTTGCATGTTTCTGGAGTTACTTCATGTTTTTATTTTTTCAGTCATGAATTACGAACACCTATGGCTGCAGTGATTGGCTTGCTGGACATTCTTATATATGACGATTGCCTAACAAATGAGCAATATGCAACAATTACTCAAATTCGCAAATGTTCCACTGCTTTACTTCGGCTTCTGAACAATATTCTGGACATCAGTAAGGTGAGATTTGGACAAATGTTCGATTGTCGGGAGGAGAGACATCATATGTATGTTATTGGTTGGTTCTGATGCAGGTCGAATCTGGAAAGCTAGTGCTGGAAGAGACGGAATTTGACTTGACTCGTGAACTTGAAGGTCTTATTGACATGTTCTCTGTCCAATGCATTAACCACAATGTGGAGACTGTTCTAGATCTCTCTGGTACAATCACTTTCTCACTATATCTATAGCAAAGCCTGGCAATTGAAATATGCAACTGCTGTTCTGCTTTCTTAGTTCTCAATGGACAATATTTACTCTATTTTTCTTCTCAGATGAGATGCCAAAACTAGTCAAAGGAGACTCGGGAAGGGTTGTTCAAATATTTGCAAACCTAATCAGCAATTCTCTGAAGTTCACTACTTGTAAGCGTGCTTCACCATTGATAAATTTGAATTTCTATCTAGTTTTGCTGATTGGATTTAGAAATTACATTACCTAAATGTGTCTATTGTTTCAAGATGGAAACAAGAATCATAGATCTTAACAGTTTTCGCTAAGCAATGCTCAATGCTTCTTCATTTGAAATTTACATTTAGATCATTCTCAATTCTCAAATGCCTTTCAGCTGGATATATTGTTCTGCGGGGATGGTGTGAGAGCCTGAATGATCTCACAAACAGCAGGAACTTTTTCTTCAATCAGAAGGATTCTTGGTCTGCACCTAAAGTGAAGTTGAAGCGAGCAGAAAGACGACCCTTCAAGAAAGATAGCAAAACAGTTCTTTGGTTTGAAGTCGAGGACACTGGTTGTGGTATAAATTATAAACTGTAAACAAAATATACAAAGGAATTCTTGAATCTTGACATAGGAGATATACAGCTTTCACTACTATATACTAACTTTCCATCTTATATTCTTATCTAGGAATTGATCCAAACAAATGGGAATCTGTGTTTGAAAACTTTGAGCAAGCTGATCCCTCAACAACTAGATTGTAAGTAGTTTATACATCTCTATACTCCCATATGAAAACGGTTTATTGAATGGATGAAAGTGCTCAGTGCCAACTTAGTATTCGACTGCCCTTTGTTAGATTGTTAATTTATGTTAGTAGTTCGAGCAAAACATTTCATGCATCTGATTCTATTATATCATCTCTATGCAGGCATGGTGGCACTGGTCTTGGCCTATGCATAGTACGTTCCCTGGTGAGTCGTGAGCTCCAATTATTCCATTGCAAATATGAAAACATATTTGGTAACTTTCTCCAACAGGAAAACGAGGATGAAAATGGGAGTTAACAGCAAACCAAACCTACTGCATCTACCTCGTTGAATCTCTTTGTAGTTAACACTTTCTAATGGAGAGAAAGGTTTTGACAGGTGAACAAAATGGGCGGTGAGATCAAAGTCGTGAAGAAAAATGGACCGGGAACTCTGATGCGGCTTTACCTGTTACTCAACGCTCCCACTGATGGTGCAGAGCAGCATAGCCCTCCAACTTTAGCAGAGCAGACGACGACTGTGAGTAAAAACATTTCTGCATCCTACCTGTGTTTATGCAGATAAATTCTCGCAAATTGGCATGTTGGAGAAACACTTTTCTTTTGATAAGTGCAGGTGTTGCTTGCACTTAATGGCAGAATGGGTAGGCTAATCATGTCCAAATGGTTAGAGAAAAATGGGCTACATACCTGTGAAGCAGCAGACTGGAATGAGCTAACACAGATGCTTCAGGGGGTTTTCGGATCCAAAAGTAGTCTGCAAGATTCTGGATGTGAACATTTTAGCGATAATAGTTCGACACTTTTGATCGTAGTTATTGACATTGGCCTTCTTAACTTGAGCACGAATATTTGGAAGGAGCAGCTAAATTTTCTGGATAAGTACAGTGAGAGAGCAAAGTTTGCTTGGGTTCTTTACCATGACACTTCCAATTCCATCAAATCCGAGCTGCGAAAAAGAGGGCATCTGATGATGGTAAACAGACCACTCTATAAAGGAAAAATGATTCAGATTTTGGAAGCTGCATTCACAAAAGATAAAAATCTCGAGTTGCAATCTGCAGAAAATACAGCTATACAAGTAAACATGCATGAATGCCATCACGAAATCGATGCCAGCCACTCATGTCTCACTAGCCCTGATGATTCTGACAAGTCAGAAACTGGGAATGTTAGACCTGTGAGGACATTCCTTGCTGAAGAGAAGCCCAACAAGCATTTCCGGAATGTATCTTCTTCCTCGATCTATGCCACACTTAACAACTACTTTGTCGACATCACTCAACCAAATCTGGGAGAAGACGATGCTTCAAGGGAGGATGATAGAAGAGAAAAAAGGAACAGATCAGAAGAACACTCAGGAAGCACTCGTCGTGTAGAATTAAGCACTGTCAGTGTCAGCTCTAGTAAGACAGCGAACGAACAAAAATCCCTGTCAGGGCTGCGAATCCTACTTGCTGAAGATACACCGGTACTTCAGAGAGTCGCAACCATAATGCTGGAAAAAATGGGAGCTACGGTTGTGGTTGTTGGAGATGGACAACAGGCCGTGGATGCTCTCAAATTCTGCAGAAACGGTCCAAACGAATCCTCCCAGGAAGACGACACCTCACCAACCTCACCAACCGAAGGATTCTGCTCTCCACCTTATGACTTGATCCTGATGGATTGCCAAGTAAGTAAAATAACGATTACTTTTCGAGGCTGCTATCATATCAAATCGAACAACGAAGTGATTAACTCATTAACAAACTAAAACCTTTTCTTATAGATGCCAAAGATGGATGGCTATGAAGCAACAAAAGCCATTAGAAGATCGGAAATGGAAACTGGAACGCACATTCCTATCGTGGCATTGACAGCTCATGCAATGTCGTCGGATGAAGCAAAGTGCCTGGAGGTAGGAATGGACGCTTATTTAACAAAGCCCATTGACAGCAAGCTAATGGTCTCCACTATCCTTTCATTAACAAAGAGAAAAAACTGA

>IbHK1b

ATGGCGTACAGAGCCAATAGAACTCCCTCTATCAGCTCTGAATCATCATCAACCCCCAACACACCCGTGGGATCACTCCCGGAAAGAATTTTGCATAAAATGTTTGGTTTTGGAAACCTTTACAGACGAAACCAGTCTCCCACTAGAAGAAGAATTTTCCGCCGAGATGTTGAAGAAGAAGAAGAGGAGTTTCAATATGCAAGTACCCTTTGCTTGTCTTCATATTATAGCGTGTTTGTGGTTCGTCTTGCCATCATGGTATGTTTATACTCAGGTTTCTCATGTCCGTACTAACTGTCTGATGAAATGCCTGTGTGAAAGCATATATTCTCTCTTTATTATTGACAAATCTTTATCTTTACTCTCAGTGTCACAATGGATGGAATTTAGCTTTGGGGGTGTTTAGTTTTGTCTGATTAAACTTTTGGAATTGAAATTTTTTTTTTTTTCTTATGCAGGTCATGCTAGCAATTTTGATTGGATTGTTAACCTTACTAACATGGCATTTTACCAGAGTTTACACGAAAAGATCACTTAACACGCTAGCATTCGGTCTTCGCCATGAACTGCTGCAAAGGCCTATACTAAGAATGTGGAACATCCTTAATTCTACTGTCGAAATAGCAACCGCTCAGGTTAAGATGTCAGAGTTTGTAATGAGGCGTTATAGCAAGGCTATAAATCAAGAACAGCAAGTTGAGGTTGCTGCCACTTCTTTAACTTTGCTTGTTTGATACTTTGAGTTACTTATGATGCATGTATTTCAAGCTTTTGAGTTTTGGCCCACTGTTTTAAAATGCTTTTTAATCTTAGCAGTTGTATGAAGCTATGAAGGATGTGACGTGGGCGTTGTTTGCCAGCAGGAAAGCTCTCAATTCGTTAACCATCAATTACAGGAATGGCTTTGTCCAGGCTTTCCATAGAGATCACAGGAGTAACAACACGTTCTACATATACTCTGATCTTTCCAATTATTCGATAAGTGGAACATATGATGCTAGTATGTTGTCGTCTCGTGATGGATGGAACGATCAATCTATACATGGCAACACATCTGCTATCTGGTACAGGGAGCCCCTGGATCCTCTTTCTGGTGTAAGGATTGGGAAACAAAGCCAAATCCAACCAGATGAGTTGATCAATATTGCGGGCATTTCTCAAGTGCCTGATGGTGCAGCCTCGTGGCACGTGGCTGTCAGCAAGTACTCCGATTCGCCACTGCTTTCTGCAGCACTTCCAGTTTGGGATCCATCTAATAAAAGTATAGTTGCTGTTGTGGGAGTTACTACAGCTCTTTATAGTGTTGGCCAATTGATGAAAGAAATCGTCGAGTTCCATAGTGGACATATATATTTAACCTCACAAGAGGGCTGGTTACTTGCTACTTCCACGAATACTCCTCTCTTGGTGAACTCCACAACAAGGCCGGAGTTGATCATGGCTATTGAGTCTGAGGACCCTGTGATACAAGCTGGAGCCCAATGCTTGCAGAAAGAGTACGGGAACAAGATTCCTCCCGGTCATGAAGTCCATATAGAAAACGCAAAGCTTGGCAATCAGCTCTACTATATTGATTCATTTTCCTGAACTTAAGGAGACTTCCTATGGTAATAATCCTCACTTTTTTGCACTTATAATTTCTAATAATCAGTGTGAAAAAATTACAACAATCGGTTTGCAGGTGGGAGTTATAATCATTCCAAGGAAATATATAATGGGGAAGGTTGATGAGAGGGCATTCAAAACATTGGTGATATTGATATCTGCATCTGTGTGCATCCTGATCATTGGATGTGTCTGCATATTCATATTGACAAATGGCGTTTCAAAGGAAATGAAACTTAGGGCAGAATTGATAAGTCAATTAGACGCAAGAAGGAAAGCAGAGGCATCAAGCAACTACAAAAGTCAGTTTTTAGCAAACATGAGGTAATCTTCATGATAAAAAGTTATTTAGTAAGCAAATTCATAAAGCAAAAGCATAGGTGGTTTACTGGTTGTTAGTGGAGTTTGACATGATTTCTACTTATTCCACCTGCCCAGCTTGTGTTTTCTGATTTGCCACGCATATAACAAAATTTATTTATTTTGAAATAAAAAATAAAAAAGACGAGAAAAAGTGTGCTGCTTCTCTGAAATTTAGGTAAATTTCATTGTTGGTGATTTGTTTTATATATTCCTGAAGCATGATAAGCATCTATTATTAACTATTACAGTACTAATCAGAATAAATAATTTCGATTTTAGTTTTTCTTGATCTGTTTGCATATGGACCTTTTTGTATGTCAGAAAGATTATAGTCTACTGATAAGTGGCTTTTAACATGGTCTTCTAGCTTTTCCTGAGTCATGAATTCGTTTTTCTTTTCTGAATTCAGTAACTTTATGGATTTTCTTTGATTTTTGAAGTTGTGTACTTCTGCAGTTACCTGATGTTTTGCTTTCTTTTGACAGTCATGAATTACGAACACCAATGGCTGCAGTGATTGGCTTGCTGGACATTCTTATATGTGACGATTGTCTCACAAATGAGCAATTTGCAACAATCACTCAGATACGCAAATGTTCAACTGCTTTACTTAGGCTCCTAAACAACATTTTGGATCTCAGTAAGGTGAGTGTCAGAAGCATATTGCCATGCTTTGCTATACAAAATTGATATATATTGTCCACTGGTTCCTGGTGCAGGTAGAATCTGGAAAGTTAGTTCTGGAAGAGACAGAATTTGACTTGAGCCGAGAACTGGAAGGTCTTGTTGACATGTTCTCTGTCCAATGCATTAACCACAATGTGGAAACTGTTTTAGATCTCTCTGGTATGGCCAGTTCTGCAGAATCTATTCTATAGTAACCACAATTCCACAAAAGAAATGTGCTTAACAGTTGAGTCAAGAAATTTTTACTTAGTTATCGATTTCTGAGTGTGCATTATTTGCTTTAACTCCTCTCAGATGACATGCCAAAACTAGTTAAAGGCGACTCGGGAAGAGTTGTTCAAATATTTGCGAATCTATTAAGCAATTCTCTGAAGTTCACAAGTTGTAAGTCTGCTTTTGTAACTGATAACCATTAGTTTTCTGGTGGTCTGCTTACTCCAATGTTACATTTAGGTTTGACAGTCTCAAATGCCTTGCAGCTGGCTATATCATTCTCCGGGGATGGTGTGAGAACCCAAACACTCTCGCAAACAGCAGGAAGTTTTCTGTCAACCAGAAGGACTCTTGGTCTGCGCCTAAAGTGAAGTTGAAGCCACACGGAAACCATGCCAGAAGACCTTCCAAGAAAGATAACAACAAAACTGTCCTTTGGTTTGAAGTTGATGACACCGGTTGTGGTATGCATCCTAAACTGTATAGAAACTGCAAGCGGGACTTCTTAGGACATGTGCATATTTTCAGTACTTCAAATTTCCATTCTATTATCCAGGAATCGATACAAGCAAATGGGAATCTGTGTTTGAAAGCTTTGAGCAAGCTGATCCCTCCACTACAAGATTGTAAGCAGTTTATGCATGTCCTATCTTCATGTGGAAAAATGCATATTGAAATTAATGTCTTTGTGCTGATCTGAATGGATCTGATCTCAAATTATTCTGTTATGCAGGCATGGCGGCACTGGTCTTGGTCTATGCATAGTTCGTACCCTGGTAAGTTGTAACTGCTAATCATTTTGGTAGATATGTTGTTCCTAGATCCAAAAACACCCTACAAAAGAATAGATCACATGAATTAGTATCGAATGGGAAGATATGAATACACCATAATGCATATTTGAACCATATTTAAGCAGCTTACCTTAAGAAAACCAAAATTACTGGCACATAAACAGATGTCTGCGTGGCTGGATCTCTGTGTACTATCTTAGTCTTATGATTAAGAGCTTTTTACAGGTGAACAAAATGGGCGGTGAGATCAAGGTTGTGAAGAAAAATGGATCGGGCACTCTGATGCAACTTTGCCTCCTACTCAACACTCCTATAGATGTCACAGGACAGCACGGACATCTGAATTTTAGAGAGCAGACAATGACTGTACGTAAAATGATTTTGCATTTTCGACCAACCACACAACTAATGCTGTCTATACTGAACTTAGTAGATTGGGTTTAGCCTATATGGATTCTATATCTCCATTTGACTCTCTTTAATACTCTAAATCACAACTCTTATCGTTTGACACCTGAATGCAGGTTCTGCTTGCACTAAATGGCAGAATGGGAAGGCTAATTATGTCCCAGTGGTTAGAGAAAAACGGAGTTCATACTTGTGAAGCGTCGGAGTGGAATGAACTGACACAAATGCTTCAGAGGCTTTCTAAAACCAAAACCAACTCTCAAGGTGCAGGCAATGCAAACACCTCGCTTTTTGTCATAGTTATTGACATTGGCCTGCTTGACTTGAGCACAAACATATGGGAAGAACAGCTAAATTTTCTTGATAAATACTGTGGGAAAGCAAAGTTTGCGTGGATTCTTTACCATGACACTGCGAATACCATCAAATCTGAGCTCCGGAGAAGAGGGCATCTGTTGATGGTAAATAGACCGCTTTACAAGGGGAAAATGATTCAGATTCTGGAAGCTATAGTAAAAGAGAACAGCCTTGAGCTGCAATCTGCAGTAAACACAACAGAAGAAAATTTGCACGAATGCCATGAAATTGATGCCAACCACTCTTGTATTGCCAGCCCAGATGATTCTGACAATTCAGAAAATGGGAAGGATAAAGCTGTGAACGCATTCCGTGCTGAAGAAAGGGGGAATGAACATTTCGCCAAAGCCTCTTCCACGTCACAATATGGAACTCTCAACAACTACTTTGTTGACTTCACTCAAACAAATTTGGAAGACAATACATCACCTGAGGATCAACCAAGGCAGGCAAGGAATAGGTCGGTAGAATGCTTGGGTAGCCCTCATCCCAGAGAAAGTACTGTCAGCTATAGTAATGAAACAAACCAGCAAAAATCTCTGGCGGGGCTGACAATACTACTTGCTGAAGATACACCCGTACTTCAGAGAGTTGCAACGATAATGCTGGAAAAACTGGGAGCAAAGGTTGTAGTTGTAGGTGACGGGCAGCAGGCTGTGGATGCTCTCAAGTCAAGGGAAGAAGGCAGCTCAACAACTACCCAAACCGAAGGATCTTGTTCCATGGCTTTCGACTTGATCTTGATGGATTGCCAAGTAAGTGAAACTATTCCAGAATCTGTACATCTACTTAACTGTTATTTATAGTGAACCTATGTAACGAACTAATGATATTCTACGTATAGATGCCAAAGATGGATGGGTATGAAGCAACAAAAGCGATCAGAAGATCTGAAGTAGCAACTGGTTCACACATACCAATTGTGGCATTGACAGCTCATGCAATGTCTTCAGATCAAGCAAAATGCCTGGAGGTGGGAATGGATGCTTATTTAACAAAGCCCATCGACAGCAAGCTGATGGTCTCCACAATCCTCTCATTAACGAAGAGCCTACAAGCTTGA

>IbHK5

ATGGTATCTGAGATGGAGAATGCTCATACTGAAGAAATGGACATTGAAGTCCTGTCTTCAATGTGGCCCGAAGATATTAATGAAGCGGGAAAACAATTTAATATTGAACAGCCGGGAGCAGACCTAGATATGCTGGAGGAGGTTACAATAAATGAGGAGGCGACGACTATAGTTGATTTCCAACGTCTCATGGAGCTTACGGACTATAGTGACAAAGGCTCTTCTCAGTTGGCATACTTGGTAAAGAATTGGGAGTATAAGCAGGCAAATGCTGTACGGTTGCTGAGAGAAGAGCTTGACTATCTTAGCAAGCAGCAGCAAGAATCCGAGCTCAAAAAATTGGAGATACTGGAGCAACATCGATTCGAGGAAGAAAGATACGGGGGTGATAAGCGTCCTGTTTCCATATTGGATGAGGATTTAAAATACATCTATCAAGATATCCCGAGGAGGAAAAAAGATGTGGTAGTTCAAGCTGAAAAGCTAGAAATAGAGGCCGAGTATGATAGCATCATATACTGGAAGCAGCGGGCCATGCATTTACAAAAACTGTTGGCAGCAAGCATTGAGCGAGAGAATATACTACTTGAGAAACTGCAAGAAAGTATAGAGAAACTTGAGCGGCAGTCCTCCCCTGTTGAAGAACTATCACAGGTTTTAAAGCGAGCAGATAACTATTTGCATTTTGTTCTTCAGACTGCACCTATTGTCATTGGTCACCAGGTATGTTCCTCTTGAAATCATGCTTATCAAAAACATAAGCAATGATGTAATATCCTTGTATAGATCTTCGAGGTATAAACCATATTTATAAACCTTATAAAAGATATAAAAGAAAAAAGTTTAGAAAATATAGAAATACTCCCAAAATTGGGGGTGGGGCTTATTGCAACTTGATTCAGCTCCCGATAGGTGAATACCGATTTATGTGACTAAAGATGTAAAAATATGGCATCAATGACTTGAATGCTGAAGAACTTGAAATTTTCTCCCTATATGGCTATTGTTTTCTGCAAGTCAGTTCAATTTCAGCAAAAAACAGTGCTTTGTTTGAGATACATTACGGATTTTGGATTCAATGTGCCAAAATGAAAGCCATTATTAATATCCAAATTCTAATGACTATTTAATAGCAAAAGCACAACTTTGGCATAAATGATTAGCTTTTCTATTTCAAACATATTTTGAATTAGTTTTCGTAGTTTACTATAAGAATGCAAATGCACATGCAATCTTACGTTGTATTTTGTTTCACTTTTAAGGTTGACCTAATTATAAATTCACTACCAAATTTCCGAAGAACAACTGTTACATTAAAAAAAAAGTTATATGAATAAAGAACTGTAGTACATATCAGCAATCTGATAATTTTTCTTTGAAAATATTTACAAGGTGTGATTGAAAGATGAACCTAATCATTTGTGATAACTCAAGGGTTATCTAGTCAAACTTATTTCTGTATTTAAATATTAGTTCTGATCATCGTTTTTATTTTTCTAGGATAAGGAGCTGCGCTATCGGTTCATCTATAATCATTTCCCAAGTTTGCGTGAGGAGGTAATCCAACGCTATATTAAATCCTTGAAATATTTGGCTTCCTAAGGTGCCCTTCCCTAAGTATATGGAGACCAATGAGGTCCTTTGCTTCAGGACTTCACTGCCTCTGAAAATTTCAAGTGAATTTTACGATTCAAAGTATGGAAGTGGAAAATGTTTATTGCATATTAATTGGCTCTATAGTGTAATGATGGTCATCATATTTACAGAATGATGTTCTTATTTTAATAGCAAGTTTCATTTTTCCGAAATTTTTGTTGGTGTTTAAACGCACTGTTTGCTGAACCTGGAATATGCATTCTTCACCTATTTTTTCAGGATATAATAGGCAAGACAGATGTGGAGATTTTTTCCGGGTCTGGTGTAAAGGAGTCCCAAGATTTCAAAAAGGAAGTTTTGGAACGTGGGTTGCCTGCAAAACGGGAGATTACCTTTGAGACAGAACTATTTGGATCGAAAACATTTTTAATATATGTCGAACCAGTATTCAGCAAGGCCGGGGAGACTATTGGTGTAAATTATATGGGGATGGAAGTAACTGATCAGGCGAGTTTGAAATGCAAAATATTCTCCCTATGAAGCGTAAGGTTATTTGTAACTGATGATAAGTAAAGCTAATATGATTATGGTACAGGTTCGAAAACGAGAAAAGATGGCAAAGCTTCGCGAGGAGATAGCTGTACAAAAGGCCAAGGAAACCGAACTTAACAGAACAATCCACATAACAGGTTCACCCATCTCTCATTCATGCTTTTTATGGGATTTGTGTGCAATAAAAAGCGGAAAGGAATTATCTCTAAAGTTTAGTTATTTGCTTTTTGCAGAGGAAACAATGCGGGCAAAACAAATGCTTGCAACCATGTCCCATGAGATAAGATCTCCTTTGTCTGGTGTTGTAAGCATGACCGAAATTCTTGCCACCACTAAACTAGAAAAGGATCAACGCCAACTTGTAAATGTCATGTTGTCTTCGGGTGATTTGGTTCTCCAACTGATAAATGACATCCTTGATCTTTCCAAAGTTGAGTCAGGTTGGTGATGATCCATTTTCCAAATTGTTTCTTTTTTCATGAAAGCATTCCAAGATATATGATATTTCTTCCAATTTCATTGCCTTTTTTCTTTGTACACTTTTGAAGAGTAATCTTCCAGCAATTATTTCGCTTCGCACATTGTGCCCCAATGAGAAAAATTTCTTGGAAAATACATGAGAATGGGCAGTAAATGGTAGTTTATTCTCAAAAGACCAGAATAACTCCATACTTGCCTCTCTCTCTCTCTCTCTCTCTCTCCCCTCCCTGTGGTTTATGTTAGAAACTAAAGTATACTGAGAATTGAAAAAACAAGGTTGCTTTTGATATAACATTTTTCTTGAATAACACGAAACTGAAATTTGTTTTTTTTTTTTTTTATTTATATTTGTCCTTTAGGGGTAATGAAATTGGAAGCTACGAAGTTCAGGCCAAGAGAGGTAGTAAAACATGTGCTGCAAACCGCAGCTGCATCACTGCAGAAACTACTGACCTTAGAAGGTTTTGTCGCAGAGGATGTCCCAACAGAGGTGAGAACTTGAATATAACACACCAAGTTTCCCAAAGATATTTCACAAGTTAGCATGATGAATTTCATCCACTATTGTTTGATTCTAAACCACAGAGAAAAAAAAAAGAATAATGTAATTGTTCCTCGATGGAATAGAGGAGGGAATTCCCACTAGCAAATATCTGCTTTGGTAGCCACTCGAAGAGAGTTCACTTCTTTAGCCTTTTATCTGGTTAGGTAACTTTATGAATCAATCACCAAATAGCGAGATAAATAGTTCAAAAACTTATCTATTCTAAAATATCGAATAGGCATGGATCTGCAGAGAAGAATCTGAATTAAGGTAGAAGACAAAAGGAAGCACCACTTGCTTATTTGGTCTATGAGGTTGAAATCTGACTCAAAGAACTGTATCTATTTCAGGTCATTGGAGATGTTCTTAGAATTCGCCAAATTCTCACCAATTTGATCAGGTACAGAGTTTGGTTAATTTAAAATTTTCTTTAGTCCTAGTATTGCATTTTCAGCTAATTGAAATCTCTGATTTTTAGTTTCATGTTGAGTGCAATGGCTTAAATTGAGATGTAGATCTACCAATTATTCTAAGTGCTAAATCTGTGCTTTTATCTTCTGCAGCAATGCAATAAAATTCACTCATGAAGGAAAAGTCGGTATAAAACTGTATGTGGTTCCCGAGCCATCTTTGGGCGCAAAACAGGGATCTCATCAGAAGCAATCTTTAGATTCCCTGAAAAGTTCGTCAAACAATTGGAAAGAGGATAGGTGTTTGTCAGCGTCTCATGGGAAGCATGACCGAACGGCCTCTTTTAGTTATAAGGATGGAGAAGGAACTTTCGAAAATCAGATGCATAAAGATGGGTCCAACCATTCAGTAAGCAGTGGGGCTTTGGATGACGATTTAGACGCCCATCCTGATCAAGAGGAAAAAACTGTGTGGATATGCTGCGACGTTTATGATACCGGGATTGGCATACCTGGTATCCTCATATTCTTGACCTCACTGGATTCTAAAAAAATTTATTCGTTTTAAGCTTCATTCGATTCTTATGGTTTTGCTAGATTTTTAGAATTCGCTTTACTTAGCATTCTGTCTTGTTCAACAGAAAACGCTTTGCCCACTTTGTTTAAAAAGTACATGCAAGTCGGTGCAGATACAGCTCGAAAATATGGCGGGACTGGACTAGGCTTAGCAATCTGCAAGCAGCTGGTAATCTCGATGAATCACATTTTTTTGTCCCTTTTTGCATCTGTACTTTGAGCAAAATTTTAGATTATCATTTTAAGTTGATTCTGCAAAATCCGTTTCATTGCAATCTCTACATTTTACATTCTTTGTTCTATACAAAGCAAAAGAATAAATAAATCAAAAGTGAAGAGCATAAATTGAACCAATACTTTACATGTCAATATGTAAATCTAAAACCAATGTGTACAGTTATTGTTCTTATGGACTAGAGAAAGAAGGGGTTTTGTTTGTTATCATGTTTATTCTAAGAAATGACGGGCTATTTAAAGTATAAATAATTCAAGAATGAATAAATTAAGAAGGGCATGTTCTAGATAAGCTATTGGCTTGGAGTCATAAAGTGCTATTATAGGATGTACGAGAGAGATAGAGATAGGTTCTTGAAAATATTGAATTCAGTTGTGAACTTTTCAATTCTGTTTTTGGCACAGGTTGAGCTCATGGGTGGCCATCTCACGGTGTCCAGCAAAGAACACCACGGTTCTACTTTCACGTTTGTTTTACCGCACAAGGTTTCACCGTTGTGTGAAAGCTCTGATGAGAACGATGAAATGTCTGATATGGGTAGTCATGACACCTCGACTGATGCAAATGAAGACGATGCAAATTCTGGCTTCTTCCAGTTCCAACCGCGTACTTTGGGTTCTTTATTTTCTTCCCACGGTTCTGGAAGAGCCCAAAAGCTGTCACCGAATACTTTTGGGTTTAATACTTTGCAGAGTTGCAATGGATTGCCAAAGAACTCCTACACTTTCCCCGCTAATAGCGTTATGCTGAAAGATATGGGATCGGTAGAAGATGCCTGTTCGGTCATAGATGTTGACATATTGTCTGACCCCGAAAGTTCTTTCAGGCAAAGCTCACATTCTGATAACCCGAGCACATTGGAAAGAGACAAACACGCTCATTCTGGTAGCAATGGTCAATGCCATCACCACTCTTCCTATTCAACCGATTCCACGAGTACAAGGAAAGATGAGGACGTGAAAACAGCTGTTCAAGAAAAAAGACAGCCTGAGGGAAATTCTCCATGCTCCTCTGACAACAACCAGGAGGTTTCAAATCAGCCCCCAAACCTAGAATCCTCCTAGTTGAAGATAACAAAATTAATGTGATGGTAACGCAATCAATGATGAAGCAGTTGGGGCATCAGATTGACATTGTGAATAATGGAATAGAAGCTGTGCGGGCAGTTCAACGCAGCTGTTATGACCTTATTCTAATGGTAATTTTCGGAATATTGTGTTTCTTTTATACTTCTTTGAGTAGAATGCAGCTTATTTTGCATTTCTGACATGATGCGGTTTGCTGTCTATATCAATTAAATGAGGGGTGGGGCAACTCTAGTCAAGTTGGTCAAACTGTTAACTTGGTAACCACAAGGTTACAAGTTAAATTCCCAGCGGGAGTGACCTATTGGCCTTCTTGGTTTGAGCCGGTCAGTTGGACAACCTAGGCTAGTTTAGCTCTTTGTGGTCCTTTGTTGGCTAGGATCACAAGGCGGGTAGTGGCTGTTGCTTTCTCTCGTCACTAAAGTATTCATGTTCAGAGTTGATGAATCATATGACGTTTTTCCTGCATGAAGTTATCCTTCATTTTTAGAATATCCTTCCGATACACAAACATATTTCATGATTTACAGGATGTATGCATGCCTGTAATGGACGGACTTCAAGCCACAAGACTTATCCGATCATTTGAAGAAACGGGTAATTGGGATGCCGCCAGGACTGCTGGAGTCGAGGAGGTGCCTTCTTCAAGCTTGTCACTAAAACGTTCAGATTCCAAATCATCAAATGGAAGAATTCCAATCATTGCAGTAAGCACTCAAACTTATGACTAACTTCTAATTAATGCTTCCGCGTTTTATTTATTTTAGCGTGTCCCAAACCGAGCACTTTGTTAAGAGGAAACAGCATGTCTACACAAGTGCTTTAGATTTCCAAATGTGCAACATTCAATTCTTTTTTGCATTTCCTTGATAAAATTTAGAACCCCGAGTTATATGTACTCCATGCGTTCATCTTTGTTCTCGTTTGTATGATTCATGACACCATACACGATCTCACTATCGAGCCATTTACAGATGACGGCCAATGCATTGTCAGAAAGCGCAGACGAGTGCTTTGCAAACGGCATGGACTCCTTCGTATCAAAGCCGGTCACGTTTCAGAAGTTGAAAGAATGCCTCCAGCAGTATTTGCCGCAGCGCCATCGCCTGTAA

>IbHK2a

ATGTCTATGAACTGTAAAGTCCATGGAATGAAGGGAGGCTTCTCTTCCAAATTCAGGCTCAAGAAGGCAAGAGAATCCCAGCATGGACCAAGTCGATGGAGGAGGCAATTATTGTTTCTTTGGCTCTTTTTTGTTGCCATTGGATTCATTTGGTTGTTGATTAGTTCCTCTTATGGGCGTTTGGGGAGGAAGGTAGAGGCCCCCCCACACTTGGATGGAGATACCACCAACTTTTTGCTTCAACATTTCAATGTTAGCAGGGAAGAAATTCATGCTCTGGCTTCCAATTTTCTTGATACAGATCAGGTCAGCTGTTCATTGTTTCAAATTTCCACACTCAATTGTCATATCTCTAGATGCTTTTCTGTTTAACTTGTCCAGACAGTTTTTCCCTTTTTGGGTGTTGGGAATGGGTAGGTGGGTATGCTGTTCAAAGATCTTGTTCATCATACTAAATAAACAAGATTAGAACTTCACATGGTTGACACTTGACAGTAGGAACAGCCCCTTCTAATTGTCAACTCTGTTTAGTGTTTACCACTTGCACTGCTGGGAGTCCTAACTAATTGAAACATCACTTTGAGGTTGAATTTAGTTAAACTGGAAAATTAGAATACTGATTCCAAAGAAAAAGTGAACAAGGTTTAAGGCTTCTTTCATAATATTTAGCTTTCTGCATACTGTATTTGTTGTGGGTTGTGTAATTGGTGAAAGTGATGAATCCCTAATTTCAACGCAGTACTGTTATCAGTTAAAAAGTTTGCTTTCGTTGTGTATGATTCTTATGGCATCCTTTTACTTATCAAGAGAGACATTTTGAATTATTATTATGATGAGTATAATTAGGAGTGTTTATGATAAAATCATGGGGTGGCATTTGAATGTTCTATAAAGAGGGAATGGATGTGGGTTTGGATTTTACTTAGAACAAGTGAAGTGTAATAATACACTTTTCTGAAATAATATATCAACTTTGGGGTAACCACTGTACTGTCTTCTAGAGCCTGGTTATTTAGGACAGTTCATTGTTCCAATTAAAGTGGCACTAAATAAAATGCCGGATGTCCTAACTAAGATTGATAGAAGGATAGGCGGCATACAACTGGGGCCATATATTGGACATTGTCTCAGCCCTTGAAAGCATTTTGGAAATCTAGTAGCAAATAGGAATAAGATAATTTCTGAATTCTTAGATTCAAATTTTGTTGAGTTTGAGAATTTTCATTTTCTACTTGATTTTCCTTCTCTTAGTATTGATGATCAAATATTAGTCTAATACATTTCTTGTTACTAATTTCTATTCTGCAGATCTCATTGTTAAAATGTAGCGGAAGTCCCAGATATGAATCAAGCGTTCTCAAATCAGAAAACCAAGTTTATGAGAAAAAATGCAAATTGGGAGAGAAAATAGAGGCTTATGGCCAGTGTCCTGTTTCAGATGAGAACACTTTTAGGAATATTGACTCTGTACTACAACAGACATCTACACCATTTCTTTCACATTGTGCATCATCTTCAATTTCATCAGACCATCAGTTTTGTGAAAAGGTACAGAGTTCTTTTAGATGGATAACGATGGTCTTGACCATCATGGCATCATGTGCCATGAAAATAAAAATAATCACATCTTTTAGTTCTTTTTGGTTTGCTTCGCTTGCTAGGACAATTGAACTTTGTACTTTCCCTCTTTCCTCCTTATTGTGATATATGTTCCAGGAACTAATTCTTCTGCAATTTTATTTTTAAAGCACTTTTACCCGTTGCACTAGTTTGCTAGATGTTGTGGCATGAATAGTTGCTTCCTCTCGCATGGGTGCACAGTGCCTCAATGTGTCATACATTTGAATTGATTCTGTAGATGTTCCCTTTTAATCTACCTGGATTTACAGAGAATATGATAATAATAAAGGAGAAAAATTCTATAATTTTGTGTGTGTGTGTGTGTGTGTATATATATGTAATAAAATGACCTGATAAGATAAGATTTGGCAAATTTATATTTCCTATATCATTTACTTGCATCAGGAAACATTGCAAGTGAGAGCACTAGGGGATCAGTGTAAGGATATAGCCTTCTGTTTCACCAAGATATTCTGGTGGATCCTTCTTGGCATTGCTGTCAGCTGGAAACTGCGGTGGTTACGTGCAGAATCTGGTAGAAATGAACAGCAGAAATTAGTTTCGCAGCAAGAATTTGGTCAGCAACCTCAGCTACTAGAGCACTTGCAACAACAGCAAGCTCATGTTGCTTCTAGAGTTTCTCGAAAGTTGTGGGAAAAGCTTCTTGTTGCATTTGTATTATCTGGTGTGATAGCATCCATTTGGTTCTTCTGGTACTTGAATGAAGACATCATGTTCTGGAGGAAAGAAACACTGGCAAGCATGTGTGATGAACGGGCACGAATGCTGCAGGATCAATTTAACGTCAGCATGAACCATGTTCATGCCTTGGCTATTCTTGTCTCCACCTTTCACCATGGAAAGCAACCTTCAGCTATAGATCAGGTGATGTTGGTACTTCATTAGCTTCCACTTTTCAGCTCATTTATGTTCTTGTTGCATTCAACTTTGGGGTAACCTGAGATTATCTCTGTATTTCAATTCAAATCCCTGACAATTTCTTCTTCTTTGGTGGCTATGCAGAGAACTTTTGAAGAATACACTGAGAGAACAGCTTTTGAGAGGCCACTCACAAGCGGTGTTGCCTATGCTCTAAGGGTTCTCCACTCAGAAAGAGAAAATTTTGAGAGGCAACATGGATGGGCAATTAAGAAAATGGAATCTGAGGATCAATCTTTGGCTCAAGAATATATGCCTGGGAATTTGGATCGTGCTCCTGATAAAGATGAATATGCACCAGTCATATTTTCTCAACAAACTGTCTCCCATATTGTTTCAATTGATATGATGTCTGGAAAGGTGTGTTTTTCTTGCACTGGCTGACTCAGCATCCTTTGATATTTCCATAAATAAACTCTCATAGAATCCTAATTTTTTCCCCCCGATAGTGTTTTGAAGAGAATATCCTCTTTTAGGACCATTTGTTTGCAAAATGACAATAGAAAAGATAAGGTACTGTGTGATTGTTTGAGGCAGTATATTTATCAATCATTTTGAGAATGGATACCAGAAATTAGTTCTAAGGATAGAAAACTTCATTTGTTTAGTTCCATTTGAGAGGTGAAGATACATCCTTGTCCAAGAAGGGATGTATCTTCTCTCTCTTTCTGATCCCTCCCTCTTTCCCTCAAAGATCATGATTGGGCCATATCTTGTTTCAAAATTTTGCACTTCATGACTACTGACTAGTACAATATGTACATATATATATATATATATATATATATATATACATATACACATATATATACATATATATATATATTAGAAAGTGTTGTCAAACCGTTTAAAGTTATAACGTTGTGTCTGTGATAAGCTAGTTGAAAGAAATTTCTTACTATTGGTTGATTTCACAAGGTATATACTCTTAGTCATCAGTGAACTATGGAGGGTGTTATATTTTGAGGTTTATTATCCTCCTATATTAGCTTTTTGTTACTGTTTTACAGGATGACCGTGAAAACATATTGCGGGCAAGGGCATCTGGGAAGGGAGTCCTGACATCACCTTTTAAGTTATTGAAGTCCAATAACTTGGGTGTAGTACTTACATTTGCTGTATATAACACTCATCTTGCTCCCGATGCTACACCAGATCAACGTATTAATGCTACTGTCGGGTAGATCATCTGCTTTTCTTGTATAGATCTTAGATTAAGTAATTTTATTGTCAAATTGCTTTGTTCTTTATGACTTAATTACTTTCTTTTGATGCTGTCATCTTTGATAAAGGTATATTGGTGCATCATACGATGTCCCCTCATTAGTTGAGAAGCTTCTTCACCAACTTGCGAGCAAACACACTATTGTTGTAAATGTTTATGATACAACAAACACACATTCTCCAATTAAAATGTACGGTGCAGATGAGACCGAGACAGAATTATTGCATGTTAGCAGCCTTGACTTTGGAGACCCTGCTCGGAAGCATGAGATGCATTGCAGGTTAGTATCATGTAGAATTGAATATATGAGAAGTGTAGTAACCGTACATTTGTTTAATTGATTGCCTGTCTTGCTCACAGGTTCAAGCAAAAACTTCCCCCGCCCTGGATAGCAATAGGAGCTTCTATCGGTGTTCTTGTAATCACCTTGCTTGTTGGTCATATTTTTCACGCTGCAATAGCTCGGATTGCCAAATTTGAGCATGACTATCAGAAGATGATGAATCTCAAGCATCGTGCTGAGGCTGCAGATATTGCAAAATCCCAGGTACCTTTCCCCTCTGTAGATTGGTAATCCCTAAATGTGGTATTCTGCACTTTGTGATGTCTCTCTTCATTTCTTTGAGATGCCCCTCCTATTCAATTCCATGGCCACTTTATCATTTCCCTCTATTTCCTTCGAGGCTTTTCCATATTTCTAGAGTTCTTTTTTAAAATTGTATATAATATTTTAATGAGCATTTCGTCTTCTGCAGTTTCTTGCTACAGTTTCTCACGAAATCAGGACCCCAATGAATGGTGTTTTAGGTACATAATCTCTTGCCCAAACTCCTATTAGGGATGGGGAGGAGTTTAATCCAGGATTCCTATTATCCATGCTTTGGATGATAACTGACATAATATGTTTCATTATTGTTAGGCATGCTTCAGATGCTCATGGATACAAATCTCGATGCTACACAACGGGACTTTGCGCAGACTGCTCATGCTAGTGGGAAGGATTTGATATCTTTGATCAACGAGGTATTGGATCAGGCTAAGATTGACTCAGGACGTCTTGAGTTGGAAGCTGTACCTTTTGATCTGCGAGCTGTACTCGATAATGTCTTATCACTTTCATCTGGAAGATCTCATGAAAAAGGGATTGAGGTACGAAATGGAACTCCTCGTCTCTTATAACCTTACATATGTTTATATATTATTGCAGCTGTCTTTCTGCAATGATTCTTACCATGGTTTGAATGTTCTCCGGGGGAGAATAGTTGGCTGTTTATGTCTCTGATCAGGTCCCAAAAATGGTTGTTGGAGATCCCGGAAGGTTTAGGCAAATAATCGCGAATCTTGTTGGAAACTCAATCAAGGTTTGCCAGTTATTTCATACTTTAGCCCAATAGGAAACTATATGCTTGAGAAATGTGTGCTCATTTGTATTAATGATTAAATAATCTACTTTCGGGGCATATTGCTCGAATGGATCTTATTGACGATCTTAATTATTTAACAACTCTTGTTCATATTGCAGTTCACAAAAAACAAAGACGGGCATGTGTTTGTCACGATGCATTTAGCAGATGAAGTGAGGTGCCCGCTTGATGTGAAGGATGAAGTCTTGAGACAGAGCTTATCCCTCGTTGAAGACCAGACGAACAGATCTTTCAACACATTGAGCGGGTTTCCGGTAGTTGACAGATGGAGAAGTTGGCAAAATTTTAAGAAGCTTAGTGAGGAAGAAAGTGACAAGATCAAGTTGTTAGTGACAGTTGAAGATACGGGTGTTGGAATTTCTCTTGAAGCACAAGGCCGCATTTTCACGCCTTTTATGCAGGCCGATAGTTCAACGTCTCGAACATATGGAGGGACGGGAATAGGATTGAGCATCAGCAAGCATTTGGTGGACCTTATGGGCGGGGAGATTGGATTCTTCAGTGAACCAGGCACCGGCAGTACCTTTTCTTTCACCGCAGCCTTTTCGAGAGACCAAAGAGGTTCGGTAGAAGCAAAGTGGCAACAATATGATACAGGTGTTTTAGACTTTCACGGGCTAAGGGCATTGGTGATAGATGGCAAAAGAATTCGAGCCGAAGTCACCAGATACCACCTTCAAAGGCTGGGATTAAACGTGAAGATAACTTCCACGGTAGATCATGCGTGTTCATATCTATCTACTTGTTCAAAGACAAGGTAATTTTCTTTTTATAAGCTTGTTTTCAAGAAAGTGTTCATTTTTTAGGGTTCCTTATATGACAATGTCTTATACTTGCCTTTGCAGTGAACCCGAGCATTTGGTCATCATGATTTTCATCGATAAAGACAACTGGGATACGGAGAATTCTTTTGCACTCCGTAACATTGTAAAGGATCTTAGGCCATATGGCTCAACGGCTCTCAATGGAGCCACGCCAAAATTGTTTCTGTTGGCAACAGAAATGAGCTCGACAGAAAGCAATCAGCTTAAATCGGATGGGCTGGTTGATAACGTATTAATAAAACCTATTCGGTTGAGTGTGTTGGCGTCATGCTTGCAAGAAGCTACTGGCTTTACGTATAAGAGGCAAGTGACAATGCCAAAACCGTCGACTCTTGGAAATCTGCTGAAAGAAAAACAAATTTTGGTCGTGGACGATAATATTGTAAATAGAAGAGTGGCAGAAGGTGCCTTGAAGAAGTATGGCGCTATTGTGACCTGTGTTGATGGCGGGAAGGCTGCTTTGGCACTTCTTAAGCCACCTCACAACTTTGACGCTTGCTTTATGGACCTCCAAATGCCCGAAATGGATGGGTATTATATTCTACTTCATCTTATATCTTCGTTCTTCCTTACGCGTTAAAATTAGTCCTAATGGATGATTTCATTTGTTATATTCTAACCTAGAACCGACTTTTGCTACTCATTTTACCGTGTCATACTGCCATTGTGCAGGTTTGAGGCTACTCGACAAATCCGCAAGCTAGAGAGCGAATATAAAGAAACAATAAATTCTGGTGAGATATTGGTTGACGCTCCTGGCAAACTGGCTCATTGGCGTTTGCCAATATTAGCAATGACGGCAGATGTTATTAGGGCATCGAATGAAGAGTGCATGAGATGCGGGATGGATGATTATGTATCAAAACCATTCGACGAAGGGCAGCTATATTCAGCATTGGCGCGCGTTTCTTTGAATCGGGATGATTATGTATCAAAAGTTCAAAACCATTCGAGGAGCTAGGCTGTTCAAATATCTGACTTTTGACTTTGCTGTGGCTATCAACTGCTTCGTAGCATCGGTCAGACTACACTTGGCCTTTTGTCCCGTGCGGTAACTCTCCCCTTTGCCTTAGATCTCCTTTCTTGAAAATTTCAACGAAGCATAAGCCCTTATAGTATTTTTATGAACTGAAAATGAAATATTAGTATGTTGTTGCAGGGGATTGTTAATGTCGGTGATGTGCATGTGTACTTACTTGAATGAACTCGTTTCCAGGAACGAATGCTAACCCCGCGTTTTTGCAAGGATGTTTCTTGATTTCTACTCGGGTAAGTAAAGTAGCTTTTCTACTTTCTCCGTTCAATTCATAACTTCACAAGGGTACCTATAGAAGATAGGTTCTGCAGATGTACTATATACCCTCGTATTTGTTGTCAATTCTTTTGTTGCTAACTTGCTCGCATCCATGAAGCAGCCATGCGTAGTGATCCCGTCTTGTTTGGTTCAGAAACTGCACCGCAATGGCACCCGGACCAGACAAACCATTCGGGTCGAGTTCCATTTGCAAGGGTTTGTGTACTTGGAGCAACATGGCAATGAACATTGGTTTATGGCTCTGTGA

>IbHK2b

ATGGGTAAAGCTTTGATGGGATCAGTTGGTGAGAATAGACAACACCAGGAGCAGCAATTTTTTGTGCTTGCTGTGGCCCATGCTCTTGTTGAAGTCACTAATTGGTGCTTTGTTTGCTCAAGAATCAATGTGATTGTGTACTTGGCATTTGTTTATTCGCCTTTGTGGTCCAGCCATCCCTTGTGGGTTTATTTACATGAGAGCCTACAAAACTAGAATCAAGATTTCACCTTTTTTTTTATTGAAGTTTTGAAATTGGTCTACTGAAGAATTGAGTGAATTTGAGACAAACTTTTGGAGAATATCTTATTAATTTCTTGATGAACTTCTCTGCTTTGAACAAGTTAGGTTTCAGTTTTTTAAGGCCTTTTCTGAAAATATGTAGGTGGGCTTTGAAGAAAATGTCTTGGAACTGCAAAAACCTTGGCATGAAGGGAAGCCTCTCTTCCAATTTCAGGCTGAGGAAGTTACTAAGTGGTGGGTGGAGATGGAGGAGAAAATATTTGATTTTGTGGCTCATATTTGTTGCCATTGGATTAATTGGGTTGCTGATTAGTTTGAATAATGGGCATATGAGGAGGAAGGTAGAAGCTCCAGACTTGGATGAAGATAGTACTAACCTTTTGCTTGAACATTTCAATGTGAGCAAGGAACGCATTCAGGTGAGCTGTTTCATTCCATATTCTAGGCCCAATATATTTCCACATTTCAATATGCTTTTCCCTTTTAATTTATTTGACAAGGAACCTTTTGTTTAAAATTTTGAGGGAAACATGGTAAGATAATACTTCTCTGAAACCTCTTTCATAAACTTTAGTAGATATTCAGAGTAGGAATATGTTCCTTCGTATTATCTTCCCGGATTTACTCTCCATGTAATGAAATTATGGACTAATAGATCCATGACTTTTAATTTTAGGCATTCAGTTCAAATAAATAAACATAAAAAGATGCACAGCTTTTCACATGATAAGGTCTTGCAGAGTGCTGTTTATCTTGACAGTTTGAAAAACTGAAGTAATGAAGTGTGAAATTCAATGTGGATCTGTGATAAGCACAGAGTTTTTTTTTTTTTTTTTTTAATTTTATTTTTATAATTAGAGTTTTATGTTTCATACTTAAAATAGAAAACTGCCTTAATTAAAATCAATAAAGTTAGATTGTATGAGATGCTGTAATATGCGATCCTACAAAACCATTTTAGTAGGTTGCTGTTCTACCATAGTTTTTGTATTCTTTAAATGATTTTGTTTTCTAAGCTAGTGAAACAGTTGTAGGATTCTCAATATATGGAGATGCTAAGGCAAATGAACAAATACATGTTTTAGATTTTGTTGTCAGGGAGAGCAAAGGAATCATATAGTAATACACTGAAGATTTCCTTACGAAATTTGAAATTAGATAGTCAGTGATACATTGTGACATGGAGATGCAGGGCCACCAATAAAATAATTGCTAAATTCCAAGATTAAGTATTGGAGTTATAAAGCTTCTATCGATTTTGTGATTATGCTTTTATCAATTTTGGAACGCGTCAAATCTCAGCATAATGTACTTATTCAGTTATTCTTGTCAATTCAACTGCACATATTTCATTATTAAAGTGTAGCAGAAGTTCCAGACATGAAATAACGCTGTCATTTGTGTTGTTAAGGTGCTAAGTGCAGAGAATGTTGTGTACCAGAAGCAATATGAACTGGCAATCGAGAAATTAGAAGCAAATGGCCAGTGTCCTGTTCCAGATGAAAACACTCTTACGAACCTTGACATTGTAGTGCAACAGATACCTTTACCAATTTCGCATTGTGCATCGTTGGCAACTTCATCAGATCACCAGTTCTGTGAAAAGGTAGAGTTCTTTTGGATGGATAAGGCCCGTTCTGGCATCATCCTCCATGAAAGAATCATCTGTGTCATTTCGGTTGGTTATTTCTGTGTCATCAGGAACCGCTTCAAGGGAGGGCACTTGGAGATCAGTGCAAGGATGCAGCCTTCTATTTCACAAAAGTATGCTGGTGGATCCTTCTTGGCATTGCTATCAGCTGGAAACTATGCTGGTTATGTGGAGAATCTGGGGGGGAAATGACCGGCAGAAACAAGTTCAGCAGCAAGAATTGCCTCAACAACCTCAGCTACTTCAACACTTGCAGCAGCAACAAGCTCAGGCATCCTCAAGAATTGCTCGGAAGTGGTGGGAAAAACTTCTTGTTATTTCTGTCTCAGTTGGAGTAATGGGATCCATCTGGTTATTCTCACACCTGAATGAAGAATTCACAGTGAGGAGAAAAGAAACAATAGCAAGCATGTGTGATGAAAGAGCCCGAATGCTACAGGATCAATTTAATGTCAGCATGAACCACGTTCATGCATGGGCTTTTCTAGTCTCAACGTTTCACCATGGAAAGCAACCTTCAGCTATAGATCAGGTTATGTTGGGTTTTTTCATTTCTGTGTTTATCTCAAATTTTCAATCAACTGGTATAGTCTGTTTCTGTCAAAAGTTCAAATGCAATCTGACCCTCCCACCCCATCTAATCCCCTCCGCCCCCTCGCTTCCCCCCAAAACCTTAAAACGAGAGAGAGAGAGAGAGAGAGAGAGTTTCTGTGGCAACTCTGATAGTATTTCATTACAAGTTGCAGAAAACTTTTGAAGAATATGCTGACAGAACAGCTTTTGAGAGGCCACTTACCAGTGGGGTTGCCTATGCTATAAAACTTTGCCACTCAGAAAGGGAAAATTTTGAGAAGCAGCAAGGATGGACCATAAAGAAAATGGAATCCGAGGATCAAAGTTTGGCCCAAGAGTATATATCTGGGAACTTGGATCCTGCTCCAATTCAAGATGAATATGCACCTGTTATATTTTCTCAGCAAACAATCTCCCATATCGTGTCAATTGATATGATGTCTGGAAAGGTGTGCTTTATCTTCTCCCATCCAAGCAAAATGATGATAAAAAAATAGTACTGGCTGTAGGTTTGAAGTAGCTTTTCGACCGATTAGTGTTTATCGGATATTAGATCCATTTAAGAGGTACGTGTTTATAGAGCTCGGAAAAAAGCTCAAGTATTATGATGGAAATTGCATAAGTATGAAGCTATTTGCACTGTAGTGGATGAGCCATACTTTGAAATGTATAGCATTCAAAAGAACTGAATCTTGGACAGGAGGGGTTAGTTTGGTCAGTGAGCATTCCTAACTTACAACCAGTAGGTTGTGAGTTCTAGTTACCATGTGGGGAAATGAAACACCTTATTTACGTTGGCTCTCAACAAAAATAAAAATAAACTTGAATACACCTTGATCATGTGGTGGTGCTGAATGGCACAAGTGATCACTGACCAGCATTTTTTTTAGAGTTTTTGTTCCTGATGTGTACAACAAATATGCAACTAGGAAGTAGTGTTATGCAATTTAATAATAAATATGAGGCAGAATGGAGTGTTAGCTTACAGTTACTGCTGTTTCTGCTTTACAGGAAGACCGTGAAAACATATTGAGGGCAAGAGCTTCTGGAAAAGGAGTCTTGACATCACCTTTAAGTTATTGAAGTCCAATAATGTCGGGGTTATACTTACTTTTGCAGTGTATAACACTGATCTTCCTCCTGATACAACACCAGAGGAACGTATTAATGCTACTCTTGGGTAGATCATCTGCTTGGATTTCTTGTATAGTGCTTAGATGAAGTAATTTAGCTTGCATAATGTTTTCACCTTCATTGACTAAATTTCTCCTCTTTTTTTTTTTTTTTTGCTGTTGTCATCTTTGATAAAAAGGTATTTTGGTGCTGTGTATGATTTCCCCTCATTAGTTGAAAAGCTTCTCCACCAGCTAGCAAGCAAGCACACTATTGTTGTAAATGTTTATGATACAACAAATGCATCTGCTCCGATTAGAATGTATGGAATGGAAGAAGCTGATTTGGATGAGACTGATAGAGAATTAGTTCATGTTATCAATCTTGATTTTGGAGATCCAGCTAGGAGGCACGAGATGCATTGCAGGTAAGATTGGTCTAGCGTTTGGATGTATAAAACAGCTGTATCCATGAATCCGTTAAAATTTAATTCCTGCCTTGCTCACAGGTTTAAGCAGAAACGCCCTCCGCCCTGGACAGCAATAGCTGCATCCATAGGAGTCCTTGTAATCACTTTGCTTCTTGGTCATATTTTCCATGCAGCCATAAACCGGATTGCAAAATTTGAGCGTGATTATCAGAAGATGATGGATCTCAAACATCGTGCTGAGGCTGCAGATATTGCAAAATCTCAGGTACAGATTGGTAGTGCGATATTCTGCATCCACTCTGACATCTCTTCTAATTTAATTGAGAGGCTTTCAACTTCCATGACTTCTTTTACCTTCTCCTTTACCCATATACTGTTTATTCCTAGTATTTGTATCGTCCTTTTTAACCTCCTAAATGGTTATCTGCAGTTTCTTGCAACAGTTTCTCATGAAATCAGGACCCCAATGAATGGTGTTTTAGGTATTATATGTTGCAGCTCTTACTGCATGATTACATGAGGGATAGGACTTTTAACCAGTTTCAAACCATTGATAGTAAACTGATATAACATGCTCCCCTTCTATCAGGCATGCTTCAGATGCTCATGGATACAAATCTCGATGCTACACAACTGGAATACGCACAGACTGCCCATGCTAGTGGGAAAGATCTGATATCACTAATCAACGAGGTGTTGGATCAGGCTAAGATTGAATCAGGCAGGCTAGAATTGGAGGCTGTAGCTTTTGACCTACGAGCTGTACTTGATAAAGTTTTATCACTCTGCTCCGGAAGATCTCATGAAAAACGGATTGAGGTACTAAACGAACTTGCTTCTGATACATGCTCTCTCTCTCTCTCATTCACTCATTATTCCAGATAGTCGAAATGCCCATTCTTACCATGGTTTTTATAATTTTTTTGGTGGAGAGAAGTTGGCCGTTTATGTCTCTGATCAGGTCCCAGAAGTTGTTATTGGAGATCCAGGAAGGTTCAGGCAAATAATTACCAATCTTGTTGGAAACTCAATCAAGGTTGGTCACTTTCATCAAACTACATGTACCTTATTATGCAAACTCTGTGTACTTCAAAAGTAGGAATGCAAATTAATGGGAATAAAAGATGAAACTTATGGTACTATGTAGATCTATTGGCAAAAGTATACCTTTGAAGGGCTACTCTATTTTATATTTGTGGGCGCTAGTGCTTATTTACTCTTGTACCGTATTGCAGTTCACAAAGGAAAAGGGGCATGTGTTTGTTTCAGTGCATTTAGCAGATGAAGTGAAGAGCCCAAATGATGTGAAGGATGAAGTCCTGAGACAAAGCTTAACCCTTGTTCAAGACCGGCCAAACACGTCTTTCAATACATTGAGTGGGTTCCCCATAGTTGACAGATGGCGAAGTTGGCAGAACTTTAAGAAGCTCAGTGAGGAAAAAACCGAAAATATCAAGTTGTTAGTGACTGTTGAAGACGCTGGTGTTGGAATTCCTCTCGAAGCACAGGGCCGTATCTTCATGCCATTTATGCAAGCGGATAGTTCAACATCTCGTACGTATGGTGGGACAGGAATAGGACTAAGCATTAGTAAACGCTTGGTGGAGCTTATGGGTGGGGAAATTGGATTCTTCAGTGAACCTGGCACTGGCAGTACCTTTTCTTTCACAGCAGCCTTTGCCCGAGCAGAAGAAGGTTTGCTAGAAAGCAAGAGGCAACGGAATGATCCATCTGTTTCAGAACTTCGGGGGGTTAAGAGCATTGGTCATAGATGATAAAAGCATTAGAGCAGAGGTCACTAGATACCATCTTCAAAGATTGGGATTAAACGTGAAGATAATTTCCAAAATGGATTCTTCGTGCTCACATCTGTCTACTTGTTTAGAAGCAAGGTAACATTTTTTTTTTAATAGTCATATGTTTAGGGATTATACTTAAAAAATAAGTAGTTTGGGAATAAAAATAAATCTTACATTCAAAATGAAAACATCTTAATGAAGGGAAAATTGCATGCACAAATCTTATGAAGGATTCTTTATTATTAAACTTAATTTTTGCACAATATCTGAGAAGCTTTCGAAACTGAGTATTAATTCTTAGAAGTTAGGATTAAAAGAATCAGCATCACTGGATAGGTTTTAACACAGTGCTGTTCTTGATATTACTAATGACTTCCTTTTTCTATTTCTGCAGTCCATTGGAGCATTTAGCTCTGATTTTCATTGATAAAGATAATTGGGATGACGAGACTTCTATTACACTCTCTAAGATTCTGAAAGAGCTGAGAGCCAACAGCTCCAACGTTGTTTCTGGAGTCATTCCAAAATTTGTTCTGTTGGCAACAAACATGAGTGCCACCAATCGCAATGAGCTGAGATCGGCTGGGCTTGTAGATAGCATATTAATAAAGCCTCTTCGCTTGAGTGCATTAGTTTCATGCATCCAGGAGACCACAGGCTTTATGAATAAGCGGGCATATAACGCGTAGGAAACCATCATCTCTTGGAAGTCTGCTGAAAGACAAAAGGATTCTGGTGGTGGACGATAATGTTGTCAATAGAAGAGTAGCAGAAGGGGCCATAAGGAAGTATGGTGCAATTGTGAGCTGTGTAGATAGTGGGAAGGCTGCATTGGCACTACTTAAGCCACCTCACAAGTTTGATGCTTGCTTCATGGACCTCCAAATGCCAGAAATGGATGGGTATTCTACTATATATAAACACTCTCTTGAACCTTTATTTTCAAAAATGGGTGGCACTGTGAATACAGGGATAGGGGTAAAGTCAGGATCTGAGATTCACTCCCATTAATGAATGACAAAACCCCGAAAATTCATTACTCCTAGATGGAGTTTTGTTATAGTTTCTTTCTTCTTGAACATTTAGATTTTTGATCCTGATGTTATTCCTTTTGTAGGTTTGAGGCTACTCGACAGATCCGCTGTCTAGAAAGCAAATATAACGAGAATATCAATTCAGGAGAGGTGTTGATTGAAATGCATGGAAAAGTGTCTCATTGGCACACACCAATATTAGCAACGACAGCAGACGTTATTCAAGCAACAAACGAGAAGTGCTTGCAATGCGGGATGGATGATTACATTTCAAAGCCGTTTGATGAATGGCAACTTTATTCAGCAGTGGCGGTTCTTTGAGTCTGGTTGATTTGGTCATTTGGATTGACATTTGGCTGTTCCCACTTCTCAGTAGGTGGCGTAACTAACCTGGGGCCTCATTTCAGATGTCTGAATACTGACTTCTTGGTTGCTGCTTGATCAAAGGACCTAGGCTGTTCAGATATGACTCTATTGCTCGGTCATGGACATGGACAAAGTACGCCTATCTTGTCTGTCCTGTGCGGTAACTACTTCCAAAAATCTGAAAACTTTTAAATTTAGACTCTCAGTACTCCAGGTCCAAAACCTAAAAGTTGGAAACTAGAAGGCCCATTTACCCTATAAAAATAGGCAATCCAGCTTAGTCCTTGAGCAGATTAATCAATGTGAATTGTTGTCTTTTATTTTGTCCTGTAATGTAATGAATTGATTATTTTTGGCAGGTTTGAAAAGACACAGACAACATTTTCATTGTAGTTTCCAGCATCTTCATTATCATCCCTTGTTCCTTGTTAATGTCTGCTTATTTTCTGTCTATGGAGTACAGTAAAGAAAGTCAATCCAAGAAGCAGAAGTGCAGGTCCGGTGAAAATCACCTGAGAGATGATATGGCTTCTCTACCCCGAGAAGTTTCCCTCGACATAATCTCCAGGCTTCCCATTACATCTCTCGTGCGATTCAGGTCAGTATGCAAATCCTGGCACAACTTGTCTCATGATCATCAGCTTGTTCATTTGCACCTGTCTCGAGCATCAAACGACAATCCATGCCTCATATTTCACTGTAAATATCCCATCAGAAACTGGCTTTACTTTGTTTTGTTGTCTGGTCGTGATGATGATGATGAGCGAGTAGTGAGAAGAATCGATCCCCCTTTTGCAGCAGACTTTAATGTGGTAGGATCATGTGCCGGCCTGTTATGTCTAGCTGATTCTTTGTTCCACTCTTCTCTCTTCATATATAATCCTTTTACTGGGAACCACAAAGAACTCCCAAAATCCATTGTATTTCAGCACCAGGAACAAAAGGTGGTTTCTGGATTCGGATTTCACCCGATTTCTAAGCAGTACAAGGTGATCAAGATTGTCTATTATGCCACCGACCCGAGCTACTGCAGGCCTTCTGGTAGGGTCAGGACTCGCTGCTTCAACCAATCAGATGTTCAAGTGCTCAGCCTTGACAGCAGCAATTGGAGAAGCATTGGAGAAGCCCCTTACTGGCTGGAATTCGGGTCAACCGGGGTGCTGGTGAACGGAAGGCTGCACTGGTTAAGAAGAAATTCCGGGTATTATCTCGATGGGAGCATTGCATCCTTCGATCTAGCTGAGGAGCGGTTTCAGGACATCCCGAAACCTTATTTTGGCGAGATCTGCAGCCTTATGGTTCTTCAAGGTTGTCTTTCTGGCGTGACATTCGATAATAGGTGTTTGAAGATTTGGGTCATGAAAGAAGAGTCCTGGGTGAAACAGTTCACAATTGAGACTTCACTAATCCCCAGTTTTAACTATCCAAAGCTACCTTATAAGCTGTGGAAAGATGTTTTATGGTGCATCCCTGCAGTGAAAGTTCTGTGCCTTATGAAAAATGGTGAGTTGCTGATACAGTGCAAAGGTGTTGGCCTGGTTGCATATAATCCTGAGAGTGGTGTGTTTAGGCATCTAAACTTTCCTGGGCTGCCTAATATCTTTCTCACAATTGTTCATCTTGCTAGCCTTAATTGGATTGATATTGCTATTTGA

>IbHK3

ATGAATTGGTTGAGTAATGGTGGAGTCATGACCACCAAGACTTTGCTTGATGATGGAGAGGAGGTACTGACCAAGTTGTGGGGGAAGATCTCTGAGAACATCTCCAAGATCCAGCATAGTTACTCTCAGTATATTGGGTCCAAGAAAGTAAGGAAAAACTGGTGGGGTCTTTTGGTGATATGGCTAGGTTTTGGGGCAGTTCTAGCTTTTTGTGCTTTCTGGTGTTTGAGTTCTCAAGCTATGGAGAAGAGGAAAGAGACACTTGCAAGCATGTGTGATGAGAGAGCTAGGATGCTACAGGATCAGTTTAATGTCAGCATGAACCATGTCCAAGCCATGTCCATTTTGATCTCGACGTTCCACCATGGCAAAAATCCTTCTGTTATTGATCAGGTTATATACCTCATCCATTGTGTTTCTGAATTTTCAAACTCATTGAAATTCAGATATTATAGGAACAAATGATTCTTACTTTGCATTGTTCAATTGCTTATGCTTCATAGGAAAATGCTAATTGCTTAAACAATGAGAAACTCATTTTTTCCTGAAAAAAGATGGGTTTGGGCACTTGGACTTTAGTTCATAGTGATGAAAATTTTATCATGAAATTATATTTGATGCAGAGTAGAGGGAAAAATGCATTTCACATTAACTGTGGTGATATTGCTGCACTATCATCTTTATTTTGGTCCAAAAAAAAAAATATAGAGCACTTCAGAATTTTGTTAGTTTCATCTAGTATCTAGGTTTTTTTTCAGTGACATTTAGTGCTTTAGAACCAACTTTGTATTCTCTTCCAAGTACCTTGACCTTCATCAAATATTCTAATTTATTAAGCTTTGCAGAGGACTTTTTCGAGATATACAGAAAGGACTGCTTTTGAGAGGCCTCTGACAAGTGGTGTTGCATATGCTGTAAGAGTTCTCCACCCTGAACGAGAACAATTTGAAAGGGAGCAGGATTGGACAATTAAAAGAATGGACCCCCAATTTCATGAAAATGAGTATAATGTAGATAACCTGGAGGCATCCCCAATTCAGGAGGAATATGCACCTGTTATCTTTGCTCAGGATACAATTGCTCATGTAATTTCCGTTGATATGCTCTCTGGAAAGGTAAGACTTTTTGGATTGCTTCCGAGATATAAGTATCTGAACTTGTCATGATCACTCATGCAGGATCACATTTGTGTTGATTTTATCCATTATGCTTAAATATACTGTTTTATGATATTATCGGCAGCTTTCTTAACTCATGTGATTATACTATGCATTCAATTTCATGAGTGTTGCTCCACCAGGAGGATCGTGAAAATGTACTGCGTGCAAGAGCCTCAGGAAAGGGTGTTCTCACTGCACCCTTCAAGCTACTCAAAACAAACCGACTTGGAGTAATACTGACATTTGCTGTCTATAAAAAGGATCTTCCCTCAAATGCAACTCCTAATGAGAGAATTGAAGCAACTTATGGGTATGCCATGTTACTTCACATGATTGTATTGCATGCTTAGAAAGTATGGAACACCAAGAATCCAATTTAATTGCAGGCCATATAGAGAAGGAAAATTATTCTTGATGAGTATTTTGAAATTCAATATTCAAAGACAATCTCATGGGATGTTTGACCAAAAAAAAAATGGTAAAATAATAATTGAATTCTGGGGAAGCCAACAACTCTGGTTTTGAGTTATCAAGTTGAGCTTATCTGAAACAAATGATTCTATACATGGTCAAATTTCCCCAATGGTAATAGTTGATTCCTTTCCTTTACAAGTGGATCAAATTTAAGCTGTGACTTCATATTTAAAATTTGATCTTCAGATGGGATGAAACCAGTAGGTTTGCTGAAAAAACTGGTTTGGTGTTTTCAATATAAACAGGTACCTTGGAGGGTCTTTGATATTGAATCACTTGTAGAGAAGCTCCTTCAACAGCTTGCAAGTAAACAAACCATCCTTGTTAATGTTTATGATACAACTAATCTCTCCGATCCTATAAGTATGTATGGTACAAATGTATCAATTGATGACCTGGAGCATGTTAGTTCCCTAAACTTTGGGGATCCATTCAGAAAGCATGAAATGCATTGCAGGTGATAACTCTTGACTACTCTTGAATAATTTGTGCTCATCTTTACCTTTTGGCCTTAAATTCACTACTTTATTAATATTGTTGTTCCCCTTCATTGTCATGGATTTAACTAACCATTTTAGCAGACTGAATTGAGTCAGGCTATACTACTTTTGGATATCCACTTTGTACAAATAAAACTATAATTATGCCTTTCTATAGCTTGCTAGATAATGTCTCTGGTCTACTCAGAAATGTCATACCAGGTGACTAATTCTTATTTTGGATGCAGATTCAAACAGAAGCCACCATGGCCATGGCTTGCTATTATTACCTCCTTTGGCATCATTACAATCGTGTTGCTCTTAGGGCATATATTTCATGCAACAATAAACCGAATAGCAAAGGTTGAGGACGACTATCATGACATGATGGAGCTCAAAAAGCGTGCTGAGGCAGCTGATGTTGCAAAATCAGAGGTATATGTGTTGTTTCTGTTATAATTTAATAGTCCTATTCTAGCATGTATATCTGGTCTTTTTGATAATGTGCTAATATATTTATTTCATTATATTGTAGTTTCTTGCTACTGTTTCCCATGAAATCAGGACCCCAATGAATGGTGTTTTAGGTGAGTCTTAGAATTTATACTAATGAACAAAATTGCTGAACATGATAGAAGTGACAATAAAATTATGTGCCACTATGTTCAGGGATGCTTCATATGCTTATGGACACTGAGCTTGATGTAACCCAACAAGATTATGTTAGAACTGCACAGGCTAGTGGTAAAGCTCTAGTTTCACTCATAAATGAGGTTTTGGACCAAGCCAAAATTGAATCTGGAAAACTCGAGCTTGAGGCAGTGTCTTTTGATCCGAGGGCAATTTTGGACGATGTCCTGTCACTTTTTTCTGGGAAATCACAGGAGAAAGGAGTTGAGGTATTTTATATTTGATCATTTGTTCATGCAAGCCTTCATCCCTTCTCCTATCAAATCTGCTAATATTTCTATTTTTATTAGTTATTTATTCATTTGTGTTCATTTATTTATTTATTCTTTTTCCAAAAAAACTATATGAGAGAGCAGTTGGCAGTTTATATCTCGGATAAGATACCAAAGTTGCTAATTGGTGATCCAGGGAGATTTCGGCAGATCATCACAAACTTGATGGGAAACTCAATCAAAGTAAGTCATTTAACTTAAAATTCGAAAAGAAAAGGAATATTTTACATCTGAAATTTTAGTGATATGAGAAAAGAAAGCCAATACCTGTATTTCTAATGGATAAAGAAAGAGATTCATGCTTGTTAATCTATGTCCTGAAGGATAGAAAATTTTGTGCCTTAACTGGGTACATTGCATCAAAGATCAGTCTAGCCTGCATTCAGTTTTCTTACCACTTATGATTTGTTTATTGGCAGTTTACTGAGAAGGGGCATATATTTGTTACTGTCCACCTTGCCGAGGAGGTGGTTGTTGAGCATGAATCAAGTTATGCTTTGAGTGGGTTCTCGATTGAGCACGAATCAAGTAGCACTTTGAGTGGATTCCTGGTAGCTGATAGACGACAGAGCTGGAAAAAATTCAAAGCTTTTCAAGAAGGATTCTCTTCTTTCAAGTTGACATCTGACCAGATCAATTTAATTGTCTCGGTTGAAGACACAGGCGTTGGGATTCCGTTTGAAGCTCAATCTCGTGTATTCACCCCCTTTATGCAAGTTGGCCCCTCTATTGCACGGATTCATGGGGGTACTGGTATTGGATTGAGTATAAGCAAATGTTTGGTGCACCTCATGAAAGGTGAAATTGGTTTCGTAAGCTTGCCAAAGACTGGGTCCACATTTACTTTTACAGCTGTTTTTGCTAATGGTTCTTTTAGTTCGAATGAGCTGAAGGGTCAGCACATTAATGACGAGTCCAACTCTGTTTTTTCTGAATTCGAAGGGATGAGAGCCTTAGTTGTGGATCCCAGACCTGTACGAGCCCAGGTCTCAAAGTATCACATTCAACGCCTTGGTATTTACGTTAAGGTGATTCCAGATTTGAATCATGGGTACACTTGTTTAAGCACTGAGAAAACAAATATAAATATTGTACTTGTGGAACAAGAAGTGTGGGATATGGATTCAGGGATGGCCACAGAGTTTGTAGAGAAATTAAGAAGCTACGATATCAGTTGTTCTCCTAAACTATTTGTTTTAGCTAATTGTGCAAGTGCTACACGAGCAAACACCTCAATTTGTGGTGTTTCTACTCCATTTGTCATTATGAAGCCATTACGGGCTAGTATGCTTGCTGCTTCTCTTCAGCGTGCTTTGGGGGGTTAACAACCGAGGAAATTACCGGAATGGAGGGCTTTCTGGTGTTCCTCTTTCTGAGCTCCTTCACAAAAGAAAGATACTTGTTGTGGATGACAACCCTGTGAACCTAAGAGTAGCTAATGCTGCCCTGCGTAAGTATGGTGCTGATGTGGTCTGCATAGATAGTGGGGAACAGGCAATCTCACATCTGCGGCCTCCTCATCGCTTTGATGCCTGTTTTATGGATATTCAGATGCCAAAAATGGACGGGTAAGTCTCACTTGATCACTCTTATGCTTAATGTATGTATATATTGTTCCTGCACTATTATTTGCTGCCTGCTTCCATTTACGTATTGACATTGCTGTATTTCTTTATTTTAACTTAAATTTTATGCTCATACGGTTAATTTTTTATATATCCATAATCTGTTATACATTTTCATAAAAGAAGTGTTAAGTTGACAATCATTATTTGATCATACAGGTTTGAAGCTACAAAAAGAATCCGTGAACTTGAACGTCAAGCTAATAGTCAAAACGAACATGGTGAACTTCTGGTAAATGCCTCAAACTGGCATGTGCCCATTCTGGCGATGACTGCTGATGTAATTCATGCTACAAATGAACAATGCCTGAAGTGTGGAATGGATGGATACGTCTCGAAACCATTTGAACCAGAACAACTTTACCGCGAAGTTTCAAGGTTTTTTCATGTCAAATCAAATTAGAAACTGTGGAAAGCGCTTCTGGTTTCGTGCTTGTAGTAGTGGCTTTGAGCTCAGACAGATACCTGTTCTTCGAGACACTACAGAGCAGTTAAAACAATGACACAAGTTTCCCAGACAGTGAGTATATTTTGGCTTAAATTTGCTTGACATTCTGATTTTTAGTTTCGATGATTACTTGGACTGATACATGTATCTCCAGATTCCAGAAAATGCATGTCTTTAATCAACACGGAATGTATATACAAGTCGAGAGTTGTTTGCGTGGATGCCATTCAAAGCTCAACCACGAGGGAAGAATCTGA

>IbHK4

ATGGGTCAGAAGATTCATAGCCAAAGTCACCACCACCACACTGTGGCTATGAGGTTGGGTGAGCAATTGAGCAGTAAGAGGAAGTACACATTGATCTGCAGGAATAGGCTCCCACAGTTGTTGGGTTGTTGGATTCTTCTCATATTCTTTGTGAGTAGTTGGATATTCAATAATATGGATGCTACCCACAAGGAGAAGAGGAAGGAGGCTTTGGTGAGCATGTGTGATCAGAGGGCTAGGATGTTGCAAGATCAATTCAGTGTCAGTGTTAACCATGTCCATGCCCTTGCCATCCTTGTCTCCACTTTCCATTACTACAAGTCCCCTTCTGCCATTGATAAGGTTAGGTTTTGTTGAGTAATTTTGAGTAATTTGTGATCTGTTGAGCATATATAATATATAAACATAAATGTGCAATCCAACTATCAGCTTAGGTTTTTAGTTGAGATGGAGCAGATGCTTCAATTTGGTATTAGAGCCAAACTCATGTCAAAGGTGGCTCCATGTGTTGCTTGGAGCCCATAAAAAAGTTGTGTTCTTGTTGATGTTATGCTTTGGCTGATTTTGCGGTTTTATAATGCAGGAAACTTTCGCTGAATACACTGCCAGAACAGCCTTCGAGAGGCCGTTGTTGAGCGGGGTAGCCTATGCGGAGAGAGTTCTTAATTCGCACCGGGGGAGCTTCGAGGATCAGCACGGATGGACTATTCGGACGATGGATAAAGAGCCTTCACCGATCAGGGACGAGTATGCCCCGGTCATACTCGCACAAGAAACTGTTTCTTACCTCGAGTCGCTTGACATGATGTCAGGGGAGGTAATGGAGTAGTGTCTGGTTATATGGATTTGGTTTCGTCGTTGTTCTCCAAGCTTATCTGATTATACGGTAGCTTATTGATGCAGGAGGACCGGGAAAATATCTTGAGGGCTAGGGCTACTGGGAAGGCTGTTCTTACAAGTCCCTTTAGGCTTCTAGGCTCTAATCACCTTGGTGTCGTTTTGACATTCCGGTTTACAAATCCATGCTGCAAGCTAACCCGTCACAGCAGGATAGGATCGAAGCAACTGCAGGGTAAATCTATATTTACGTGTCACTATAATAAATAAGGTGTTGTTAGCGAGTGGACTATAATAAATAAGGTGTTGTTAGCGAGTGGTTGACATATTTACATGTTAAATGAACTGGAAATTTTTTAGAGTTAGGTTGGAGGGCATCATGTGCAATTGAAATGAAATGAAATTTTTTAAAGTTAGATTAGAGGACATCATGTGCAATTGACCTGTTTTATGAGAGTTGGAAAACGATAAATTATGTAGCCCTTGCGGTTTGTGGAAAACTTATTTTTTAAGATAATATAGTTTTCTTGTCATTCTAATATAGTCTCCTGAGAGCGGTTTAGTCCCGTTTTAAATTTGTAGCGTATTCTAAATGGCGAACCGCACCTTCCCGTGTTGAACTTTATATGTGAATCCTAAAATTCAGAAAATTCTGCAATGGCTATTACTTTTTTAGAGAAAAACTCTGGATTTTATGACGGAATAACTTACAAGCTTTTAATTTTTTTGAATGACGAGGGAAACCTGTAGCCACTACCCGAGGGTTTGCATTGGTTAAACCCTCCTTATGACCCTAGCCAGAAACAACCTGATCAATTTGGCTAGGGTTGCCCCGATTAGGCTTTATTTTTTGATTAACTATCTCTCTGTAAAGGAGGTTTATTTTTTGTTTAATGTATTGCTGTAAGCAGATACCTTGGTGGGGCCTTTGATGTCGAGTCTCTAGTTGAGAACCTGCTCGGCCAACTTGCTGGGAACCAAGCAATCGTCGTGAATGTTTACGACATTACCAACGCTTCTGATCCTTTAGTCATGTATGGACAACCGGGCGAAGAGGGCGACCTGTCCCTGACACACGTGAGCAAGCTTGATTTTGGCGATCCATTTCGTAAGCACGAGATGATATGCAGGTCAGTTTCCTCAGTTGCTGTCTTCATTTTTGGGAAGCCGTTTTACCTTAAAAACGAAAAGCCGGAACTTCTCGAGGTTTCTTAAAGATGTACATTGTGAATATATCTATGCAGGTATCTTCAGAAGGCTCCGACAGCGTGGGCTGCAGTAACCACTGCATTCTTTATCTTCGTGATCGGCTTTTTGGTTGGATATATGATATACGGTGCTGGAATTCACATTATTAAAGTCGAGGATGATTTCCACAAAATGGAGGCATTGAAGGTTAAAGCCGAAGCTGCTGACATTGCAAAATCTCAGGTATTTGAACAAACCTTTTCGGAATGTGATATGCGATTCCTATTCATTAATCTGTTGCCCTTTGTCCAAACGTCTGAATCTCGTTTATGCTAATAACAGTTTTTAGCTACCGTTTCACACGAAATAAGAACTCCCATGAATGGAATCTTAGGTAAGGTGAACCTTTTCCTTGTTCGATTTTTAGGGTTTCGTTTAAAAGAATCAATTTCGGTATCTTAAATCTCTTTAATGTTGCTTTGTTATTGCAGGAATGCTTGCTCTGCTTCTTGACTCGGATTTGAGTTCGACTCAAAGGGATTATGCTCAAACGGCACAAGCCTGTGGAAAAGCGCTGATAACATTGATAAACGAAGTGCTGGATCGAGCAAAAATTGAAGCGGGGAAATTAGAACTCGAGATCGTCCCGTTTGATCTTCGCTCAATACTCGATGATGTTCTCTCTTTATTCTCCGAGAAGTCTAGGAAGAAAGGTGTCGAGGTAGGGACCATGAAGCTTTCCGTACAAATTCATATATTGCTCATGATTGTATACTGCATTGATAAGCTTTTCGGTAATTCATAGTGCAGTTAGTTCATGATTTTTCTGTTTTGTTTTAAATTTTCGGTTTCTCCAGTCATAGCATTTCTCGTGTACTTACTTGTGTTTTTCGCCTGAATTGCGGTGGGTGGTGTTCACTCCTTCCCGACACTACACTAATTTATGGAAGTGAAGTTGGCTGTCTTTGTTTCTGATAAGGTTCCTGAAATTGTTTTCGGGGATCCCGGAAGATTCAGACAAGTGATAACAAATTTGGTTGGCAACTCTGTCAAAGTAAGTGATGTCTGAAAACCTTAAACATTCTATTCTATCATCGTAATCTTCTCGTTCTTGGAGACAATATGAACATTTAAAGACTACGATCACGTTAATCTATTGATGACATACAATTTTCCTATCGTCCTTGTAGAAACTTTAACAAGAATGTAAATGAATCATTAGACTTTTAATAGAAATGTTGTTAAGTATGAATAGCCTTTTATAAGATTTTTGCGTTATTTACATATTGATTTTTTTTTTTGTCTTAACAGTTCACTGAACGAGGGCATGTATTTGTTCAAGTTAGTCTAGCCGAAGAAGCAAAGGCAAAATCCGAAGCATGCTTGAATGGAGGATCTGAAAGATTTATACCGTCGAGTGGGTATCATTGCGAAACCCTTAGTGGTTATGAAGTTGCCGATAACCGGAATACTTGGGACAATTTTAAGCATGTAATTCCCGATGAACCGTTGTACTATAGAGCTGCGAACAAACTGATGACTGATGATGGGTCTCAGAATGTTACTCTGATGGTATCTGTTGAAGATACTGGGATTGGGATCCCGTTACATGCACAAGACAGAGTTTTCACGCCCTTTATGCAAGCCGACAGTTCCACTTCTAGAAACTATGGAGGAACCGGGATTGGATTGAGCATTAGCAAGTGTCTAGTTGAGCTGATGGGCGGTCAGATAAACTTTATCAGTCGTCCCGATGTAGGAAGCACATTTTCTTTCACGGTTAACTTCCAAAGATACGAGACGAATGGCAGCGTTGATTTGAAAAAGGGTCTTTCCGACGATTTGCCTATGTCGTTTAAAGGACTAAGAGCTATTGTCGTGGATGGGAAGCCAGTTAGGGCTTCCGTAACAAAGTATCATTTGAAGAGACTCGGGATCCTGGTCGAGGTGGTGAATAGCATTAAGAAAGCTGCAGCGGTATTCGGGAAAAACGGTTCCCTGATTTCCAAGTATGTTTTATTTTTATCGATGAAATTGGTGAAATTCATTTTCGTGAAGAACAGATAGCATTAAGTTATGTACATTGTGTTTGTTTTTGCAGAGGTCAACTTCAGCCGGATATGATTCTTGTCGAGAAAGATGTGTGGATATCTGAAGATGGCGGGGGGCTGAACTTACAGATTCCGAACTTGAAACCGAATGGGCACACATACAAGGTGCCTAAGATGATTCTTCTTGCGGTGGATATTTCCAGTGCGGAGTTTGAGAAGGCTAAAGCTGCGGGATTTGCGGATACCATCATAATGAAGCCTTTGAGGGCAAGCATGGTGGGGGCGTGCCTTCATCAGGTGTTGGGAATGGGAAAGAAGACTCAAGGAAAAGACGCGTGCAACAAATCAACTCTTCGTGGCCTACTTTGCGGCAAAAGAATCTTGGTGGTCGATGATAACCGGGTAAACCGGAGAGTTGCAGCTGGCGCACTCAAGAAGTTCGGGGCTGATGTCGAGTGTGCGGAGAGCGGGGCAGCTGCTCTTGCATTGCTTCAGCTACCACACAATTTTGACGCTTGCTTCATGGACATTCAGATGCCCGAAATGGACGGGTATGTGTAAAATCTCTTAATTTTCTTTACTCTTGATGTAAGCCCGACTTGCTCAATTTCGCGTCGTATAGGCGAGAACTCTTTTGATCTTTAGGGTAAGAGTAAGTATTGTTTAGTTAAAATGTAATACTTTTCTATGCATTTATAAAAAGTGTTATATGTTTCCTGGTGAGACGGTCTCACACAACTGCATTTGTAGATCATGAAACCGTGACTATCGATATCTAAGCGTGAGCGGATTATGGTTTTGGAACCAAATGCTGTGTATAAATTTGGTTAACGAGGCTGTGAATTTTGTTTGCTGCAGATTTGAAGCCACTCGTCGCATTCGTAAAATGGAAAACGAAGCTAATGAGCGGGTGAACGGAGGACTAGAGGGAGAAGGAAGGCACAAGTGGCACGTCCCGATATTGGCCATGACTGCCGATGTCATTCATGCCACACTAGACAAATGCCTCAAAATCGGGATGGATGGATACGTCTCAAAGCCATTCGAGGAAGAGAATCTCTACAAGGCCGTGGCCAAGTTCTTCGAATCGAAGCCCATGCCCGATGTCTAAATTCCCCGCTAAGCAATCCCCACAGCACGGGTCTCCTGAAAAGTGCATCTGAGCAGCAAAGTTTTCCGCGTTCTACTGGTGCGGTGTTGGAAGCCGTGGATGTCCTGACGAAGCGTCGTTCAGATGTGTTCCTGGGGATGATAACAGGGTGGTCGTTGTCGGTTATTTGTAACTAACCGGTTTTCAGTTTTTAACTAATTAGCTATAAAATGCGTGTACCTATAGTTGCCCCAGGAATGTTATTATGTTCCTTCTCCAGTTCTCCCTCCCAGGTGTATGTAGATTTTAGAAGAGGAGGAAGCTAAAGAGAGTACAATCTGACAATGGTAGCCAAAGATGTACAATATTGACATTCTTGTATGGTCTTCAGAGGTTTCTGTTGAGGTTATCCATTCCTTTCATTTGTGCATCAGTCCTGTCCTCTTTTTGGTCTAGGTAATGTTTTTCTGCAAAAAAAAAAAAAAAGAAAAAGAAAGTGTATTATTTCATTTGCAAAATTTCATTTTTGTTTTGGTAGTACTAGTACATCTTAGAGAAATGAAGACTTCACAGGTTATTGTAGGTGTTTGACTTATTTAATTCATTTTTTTTAATTTTATTTTTGGGTGACAAGAACCCGTAATCACTCCCTGAGGACGTGGATTAGAGTTTGGGTAAACAGAGTTTTGTGTAATAGCTCTCAAATTACTAACCACAATAAAAAGATTTGTGTGACGAGTTAGCTCAAAAGGGTGTCGGTTATTTACTTTATTTTTGATTGTTTTGTTGGTGGCCTGTGTCGAACATAATACATATAAATAGTGTATAGTCTGGTTATGTAACATTGTTAAAATAATCAGAGTGTTGAAGATATCGAATGCAAGATGAAAAAAAAAATATAAAATATAAAATATTTTAAAAATGTCTAAAAATAGTTTTTCATAGAAGGTGCGAAATTCCCGCTTAGTCGAATAAACTCACTCCCTCTCCCCTCTGCTATTTATTTTATTTTAATTTTTCTAAAAACATAAAAAAATACCATTTTTGATTTGATTCGAGATTCAAGGATTGTAGCACCTGACATTTCATTCTTGACAAATTCAAAATGTCGGTCAGTGAAAAATCTTGGAATTTTAGTTCGGATTATGATGCCAATTCTTCTATGATTTTCTATGCATTTGTTAGATGAATTATTATTATTAATTTATTATTATCAATTTCATCAGAATTGAGAATACCCTGGAATGAAAAATGGAATTATAATTATTATTATTGTTATTATGATTTTTGATTGGTTTGTTGTTTTGTGTGATTGAGAAAGTGAAGTGTAGTGTAGGTCCCTCCTGCTGCTGCTGATGTTGATAACAAGCCTGGAGATGATTGTCCCCCAACTTGGACAATCAATGTGAATGAAGTGAGTCAGATTCATTCTTAAACCAACATTCTTTTTTTTTCTTTAAGAATGTTTGGTTTTACATTCTTGTTTTGGTTCTCTGATAGCTCAGATTTGTTTTAAAAAAGGCATTGAATTCTTGTTTCTGTGTCACAGGTAAGAACGCTGAAAGTAAGCAATATCTCAGTGTCTGCTTCTGAGGATGATATCAAGGAATTCTTCTCTTATCCTGGGGATCTGCATTTCATTGAAGTGCAGAGGTGAGTAAGTTCTGACAATATCAATCCACCTTTGTAAATGCACTTACACATTCGGGTTGTTGAGCATATAATATATAAACATAAATGTGTAATCCAATTATCAGCTTAGACTTTCAGTTGAGATAGAACACATGCTTCAATTTGGGTGGGTGCGAAAAGATTTGTGTTCTTTTCTTGGAGAGTTGTGAATTGTGATTATATATATGTGTGTGTGTTGCAGGGAAAGTGACACCACTCAATTTGCATATGTCACATACAAGGATCCAAAGGGAGCAGAAACCGCAATGCTTCTTTCGGTATGTCACATTTTGCGGTTTAGTTTCTACATATTGAACTGGTATCTGCATCTGTGCAATGAGACTAATCTAGTCAAGTCGAACTTTAATCGAGAGGCGACTCTGCAAAGGGCATTGCTTAGTTTAAATTTCAAAAACATATCTCTAACATAGTTTGTTTAGTGGACTGACATTCTCATTACAATTTTATCTCTTTTATCACTTTTATGTATGATTTTCTTCAGGGAGCTGTCATATCTAATCTTTCCATTTCTATAGCACCAGCTGAGAACTATGATCTGCCTCCTAATGCTCCTCCTCTAACCCTAGTAAGTTTTTCTTTTTTTTCTCAATAATTCATGAAAGCAAAAGCTGCAATAGCTAGAATGCATGATGATTATAACAGGATTAACAGCAGAAAACAACTAACCTGAAAAGCTGCCAAATCTATGCACCATTTCTCCATATGCATCTGGTACACAACAGCCAATCTTATTTGCAGCAGAGGAATAAATCTACGGCTGTCATATATCTCCATAAGCTCAGGTGTCTTACAAAACAGTTATTGTCCTTCCAATACATCTCACCTATTCACAAAACAATGACAACTGGAATTTGGATATTATTAGCAATCCAATTCCAAACACCATAAATAAGTTCTCCAGGACTTGAAAACTTATTCTCAAACAGAAAACCGTTTCTATGTCTCCATAGCATCCACGATATAAAAGAAAATTCAGTTTTCCTTTCTTCACTCATACCATCAAGTTGATTTGAAATCCAGAAATCAGTGCTTACATCTCGAAAGACATTCTTGTCAAGTCCAATACAAGAAAGGGCGCAAACTTGACAAGCATACAGACAGTCACTTAGGTTGTGTACAGTGGTTTCATTCCCATAATGGGAAGGAGTATATGCTTAAAATTAAACAGTCTTGGAATCCAATTGTTCTTCCAAATTTCAACACACTCCCCATCACCTATAGCTAAGATAGCTTCTTTCTGTTACTAATATTGTTTTCTGTTAATGGCATTATTTATCTTTCACATATTTCGTTATTAAGGGAACACAGCCAGAATCCGATTCTGATGCTGTCAAGAAGGCGGAGGATGTTGTAAGCTCCATGCTTGCCAAAGGCTTCATCCTGGGAAAGGATGCCGTAAACAAGGCAAAATCTTTGGATGAAAGTTACCATGTGACATCAAATGCCTCAGCTACGGTTGCTTCTGTTGACCTTAAAATGGGAATAAGTGAGAAGCTAAGCATAGGGACAGCTGTAGTTAACGAAAGGGTTAAGGAGATGGACCAGAAGTACCAAGTTACAGAAAAGGCAAAATCTGCACTTGCGGCAGCTGAGCAAAAGGGTCTGCTATCATGAGCAATCCTTATGTCTCAACTGGAGCATCCTGGGTTTCAAACGCATATAATGCCGTAGCGAAAGCAACAGAGGACGTGAGCACAATGACAAAGGAAAAGGTTGAAAAAACCGAGGAGGAGAGACGGGCTGCAATTGGTAATGATTCTGAAGCTGCCCTTCTTGATCAGTCTTTGGCTGGAGATCCTGCCCCTAATTACTCTACTGATAGCAAAAAACTCGTATAA

>IbERS1

ATGGAGTCTTGTGACTGTGTTGAGATTCTGCTTCCAACTGATGAGCTGTTGGTGAAATACCAGTACATATCAGATTTCTTTATAGCGTTTGCCTACTTCTCAATTCCATTGGAGCTTATTTATTTTGTCCACAAATCAGCCTTCTTCCCCTATAGATGGGTGCTGATGCAATTCGGGGCGTTTATTGTACTTTGTGGAGCAACCCATTTGATAAATCTCTGGACATTCTCTTCGCACTCGAAAACAGTTGCTATAGTAATGACGATTGCAAAAATCTCAACGGCCATTGTGTCGTGTGTCACTGCTTTGATGCTTGTTCACATCATTCCTGATCTGCTAAGCGTGAAAACAAGAGAATTGTTCTTGAAAACGCGCGGGCAGAAGAGCTTGACAGGGAAATGGGGCTTATTATAAAGCAAGAAGAAACTGGGAGACATGTTAGAATGCTGACCCATGAAATTAGAAGCACGCTTGACAGGCATACGATATTAAGAACTACTCTTGTTGAGCTAGGTAGGACCTTGGACTTGGCGGAATGTGCTCTGTGGATGCCAACACAAAGAGGGATGGTTTTACAGCTTTCCCATACTCTTAACAATCTTATACCCGTTGGATCTACTGTGCCTATAAACCTTGGCATCATCAATGATATTTTCAACAGCTCAGGAGCCATACTGATTCCACATTCTTGCGAACTAGCAAAGATGAGGTCTACTAATACTGGGAGACACGTTCCACCTGAAGTTGCTGCTGTTAGGTGTTCCACTTATACATCTCTCAATTTCCAAATAAATGACTGGCCCGAACTCTCTGCAAAAAGTTATGCCGTCATGGTTCTTATCCTCCCAATGAACGGAATTAGAAAATGGCGTGAGCATGAGTTAGAACTTGTTCAAGTTGTTGCTGACCAGGTTTTTATCCATCTCTGATCTTGATTTTGGCATCTTCGTGGATATTCCATTCTTATGTTAACTCTCTAATGCGTATAGAGGGTAACTTGCCTGTAAAGCTTATCTTTAATGTATACTTGCAGCCTGTATCTAATTGTGTTTTATGTATGGTTGTAGTTTTCCCTTTCAAATTTACGTTTGATTCCTTCGATTGATCCTGTAGTCATGTTGGTTCAAGCAATGAATCTATCAAATAGTCATATTATATTAAATGAAATCTATCTACTGGTCCAGGTTGCGGTGGCTCTTTCTCATGCTGCAATTTTGGAGGAGTCAATGCGGGCGCATGATCAACTCATGCAGCAGAATATTGCTTTAGACTTAGCCCGACAAGAAGCAGAGATGGCTATCCATGCTCGCAATGATTTCCTAGCTGTGATGAATCATGAAATGAGAACACCGATGCACTCAGTCATAGCTTTGTGCTCACTGCTTTTAGAAACTGACTTAAATCCCGAACAGAGGGTTATGATGGAAACTATACTTAAGAGTAGCAATCTTCTAGCAACACTGATTAACGATGTCCTAGATCTCTCAAGACTCGAAGATGGCAGTCTCGAATTAGAGAACGTAACATTCAATCTTCATGGAGTGTTTCGGGAGGTAAAATCTCAACACTACACTTCACATACTGTATTACATCTGAGCTACAGAATCTAACATGAATGTTGTTTTACCTTATTGCAGGTCGTTAACATGATTAAGCCTATTGCAGCAGTGAAGAAATTATCTACGACTTTATCTTTAGCTCTTGATGTTCCCATCCATGCTGTTGGAGATGCCAAACGTCTTACGCAAATCATGTTGAATGTTGCTGGGAATGCTGTTAAGTTCACAAAGGAAGGTCAAATTTCTATCGAGGCTTCTGTTGCGAAACCAGACTACATTAGGGGCAGTCGGCAGGGAGAGTTTTATCCACCGTCTACCGAGGGCCACTTTTATCTGCGTATGCAGGTAGAGTATTCCTTTTCTTTATCACCTGAAAAAAAAAAAAGTGATTGGCCTCAACATGGCTATTAGACTGTCATGCATCAGCAAACAATGTTTATGGAAATTGTCTGTATCTAAATGTCAAGCAGAATCCTAGATTTTCCGTTTTTGGTCAAAGGTTTCTTCACGGCCCTCCTATATTCCATGCCTTCTCTTCCCAACATCATCTCGTGTTTCCCCAGTCTGGAAATCCCTGTTTTAGTGTAGTATAATAACAACGTATAGAATATAGAATATAACCTCGTCTCGCTATTCTCTTCATGCAGGTCAAAGATTCGGGGTCTGGTATTAGCCCCCAAGATATTCCACTCATCTTCACCAAGTTTACAGAGGCTCGAAGTGCATCAAATCGAAGCAACAGCGGGGCTGGTCTTGGACTTGCCATTTGCAGAAGGTATTAGCCCATGGTTTTAAAGCCACAAGCTTTAATTTTGCAAATCTCAACTCATAAATAACTTCCATTACTGCCATATAACAGCATGTTGCCCTTGTTCATTCTTTTATTGCCATATGCATTGTATTTAAGTCTTTTGATGCGAAACAATGAATTGAACCAGGTTTGTACAGCTGATGGGTGGTCATATTTGGATAGAAAGCGAGGGCCTAGGTAAGGGCACCACGGTTACTTTCATTGTTAAACTTGGCTCGTGCAACTATCCAAATGCCCCGGCCATTGTGGCCCCTCGAGTGAGAGCAAATCAAGGCAGCGACGATCTCTTCAAATATAGACAGTACCATAGAGCAGATGGTTCAATGTACGCCCCCGTTCCACGCTATCAAAGGAGTCTTTAG

>IbETR1

ATGGAGTCTTGTAATTGCATTGACCCGCAATGGCCTGCCGACGAGTTACTGATGAAGTATCAGTATATATCAGATTTCTTCATCGCACTTGCTTATTTTTCCATCCCAGTTGAGTTGATATACTTCGTTAAGAAATCTGCAGTTTTCCCTTATAGATGGGTGCTTGTGCAGTTTGGTGCGTTTATTATTCTTTGTGGAGCTACACACTTTATCAACTTATGGACATTTGGCATGCATACAAGAACAGTAGCTATAGTCATGACCACCGCAAAGCTATTGACTGCATTAGTTTCATGTGTGACTGCGCTCATGCTAGTGCACATTATACCTGATTTATTAAGTGTCAAAACTAGGGAACTGTTCTTAAAAAAACAAGGCTGCAGAACTTGATCGAGAAATGGGCCTTATCCGCACACAAGAAGAGACAGGTAGACATGTTAGAATGCTTACTCATGAAATTAGAAGCACTCTTGATAGACATACTATTTTAAAGACTACACTTGTGGAGTTGGGAAGGACGCTAGGTTTGGAAGAGTGTGCACTGTGGATGCCTACCCGTACTGGACTAGAGCTTCAGCTTTCATACACTCTTCGGCACCAAAATCCTGTTGGATTTACTGTACCGATTCACCTTCCTGTAATTAGTCAAGTATTCCATACAAATCGTGCAGTAAAAATATCGCCAAATTCTCCAGTAGCAAGGTTACGACCTGCTGGAAAGTACATCCCAGGCGAGGTGGTTGCCATCCGTGTCCCTCTTCTGCATCTCTCAAATTTCCAAATTAATGACTGGCCTGAACTTTCGACAAAACGCTATGCTTTGATGGTCTTGATGCTTCCTTCAGACAGCGAAAGACAATGGCATGTCCATGAGTTGGAGCTTGTTGAAGTTGTGGCTGATCAGGTTTGAAACTTCTGCACATCTCTTGATTAGCTACAAATGAGCTTGAATTGCAATTTATTAGAAAATTCTTCAATACAAAGATTCAATAGTTCCCCTCCTCAGCTAGAAAATTCCTCAAGCATAAAAAGCTTTTCTGAACTCATTTTGTGTCGGGAAAATTGCACTTTTCGTCCCAAAGTTATAGGGTAATTGTAGAATTCGTCCCTAATTGTTGGTTATGCTCACTTTCGTCCCTAAGCTATAATTGACGTTGCAAATTTTGTCCCTTTATTAACAAAACATTAGCGATGAAATTTGCAACATTAGTATAGCTTAGGGACGAAAAGTGAGCATGACCTGTTATTAAAAGGACGAAATTTGCAACGTCAATTATAGATTAGGGACGAAAAGTGAACACGACCAACAAATAGAGACAAATTCTACAATTGTCCTATAACTTGGGGACGAAAAGTGCAATTTTTCCTTTTGTGTCCTTTTTTGGTTCTGTTATAGGTGTTGACCTAACTTGTTGTCATTGTTGCCCCAGGTTGCTGTTGCTCTCTCCCATGCTGCAATTTTAGAAGAGTCAATGAGGGCAAGGGATCTTCTTGTGGAGCAGAACATTGCCCTTGATCTGGCAAGAAGAGAAGCTGAAACAGCTGTTCGTGCTCGTAATGATTTCTTGGCTGTTATGAATCACGAGATGAGAACACCCATGCATGCAATAATAGCGCTTTCTTCCTTATTACAAGAAACCAAGTTGACGCCTGAACAGCGTCTGATGGTGGAAACAATCCTCAAAAGCAGCAATCTTTTGGCAACACTCATCAATGATGTCTTGGATCTTTCAAGACTAGAGGATGGTAGCCTGCAACTCGAGATAGGCACTTTTAATCTTCAGGCTCTCTTTTGGGAGGTAGGATTATTTGTTTTGTTCGTCTATGGTTGCCTTGTTTTCTCCATTTTTCCTTTACAGCTTTGGATGATTAAGAATGTCTCATTATTACTGTAGGTCCATAATTTGATCAAGCCAATTGCCTCTGTGAAAAAGCTGTCTGTTACTCTAAGTTTGTCTTCAGATTTGCCCGAGTATGCCATTGGTGATGAAAAACGGCTAATGCAAGTTCTGTTGAATGTTGTTGGCAATGCTGTAAAATTCTCTAAAGAAGGCAGTATATCGGTTTCTGCTTTTGTTGCTAAATCGGAATTTTTAAGAGATCCTCAAGCTCCTGACTTCTTCCCAGTGATAACTGAAAATCACTTTTATTTACGTGTACAGGTCTGTAAATTAGGCATTTTTTCTTTTGTGCCTATGTGCCAATGTGCCATTACCAATCAAATGATTGATTTCATATATCTTGATTTTCTCACTATCTGCTTTTCGTTTGTGCAGGTTAAAGATACAGGAGTGGGAATTAACCCTCTGGACATTCCCAAAATCTTTAGCAAATTTGCTCAAAACCAATCATTGGCTACTAAAAAACTCTGGTGGTTCTGGGCTTGGCCTTGCTATTTGTAAGAGGTATGTTCTCCCAGTTTAGTTCCACTTGCCCGTGATCTGTGTAACAAAAGGAACACGTTTTCCTTTCTATGGAATATGAAATGCAAATACACAAGGGTGCTCGACTTGAAATTATGATTGTTTTCTTTATCTGATATAATCTTTCGATTGTGCCATGACAGGTTCGTAAATCTTATGGAAGGACATATTTGGATTGAAAGTGAAGGTCTTGGCAAAGGAGCTACTGCTATTTTTATCGTCAAACTTGGAATTCCTGGACTCTCAAATGAATTGAAGCCCACTCTTGTTCCCAAACTTCCAGCTAATCATATCCACACAATTTTTTTAGGACTCAAAGTTTTGCTTATGGATGATAATAGGTGAGTTACTGTATTATGACCTTTTCCCGTTGATTTCATCTTTTCATTCATTCATACTGCAACATCCATGTCGTTACACGTTAGACTTGTCTAGGCATCAAAACTATCGGTTTCTGCTTCTTTCTTTCTTGATACTGAATTGCTGAGAAGGGATTGGAAAATGAAACTTACACCTTATGAAATACAAAATAACTAGAATCTGTTCATCAACAAAAACCAAAAAGTTAAAAGTAATGTGAAAATGAATTACTTTTCCAAAAATACTGTATAGTATCACATGAACTGTATGGATACATACATGAAATACATGTGAATCTCAGTGTATTTGAAGGATATCTTACTAGACAATTCTATTTGATTTACCCTGTAGCTTAATTGTCGAATAACTTTGAATTTGATGGATATATTGATTATTGTGGCAGTATGAGCAGGATGGTGACCAAGGGACTGCTGGCGCACCTCGGGTTTGATGTAACAACTGCGAATTCTGGAGACGAGTGCTTGAGAGTGGTTAACCAGGAGCACAAGGTAGTAATTATAGATGTGAGCATGGCAGTCGTAGATGGATACAAGCTCTCTAATCAGATACATGAAAAGTTCTCAAAATGTCACGAGAGGCCATTCATCGTGGGGCTTATAGGAACCACGGACCGAAGCGATGAAAGAAAAATGCTTGCGGGCTGGTATGGATGGAGTAATACTAAAACCCATTTCTGTGGAGAAAATGAGGAATGTTTTGACCGAGCTTTTTGAGCATGGAGTTGTCCTCGACGCTCAATAG

>IbHKL2

ATGTCGAAGATATTAGCATTGAGGGTGTTGGTTTGGGTTTCCCTTGTTGCATTCACAGTTGCTGATAATGGGTTCTTCAGATGCAACTGTGATTATGATGGATTTTGGAGTATCGAGACGATTATGGAGTGGCAAAAAGTGGGCGACTTCTTGATTGCAGTGGCCTATTTTTCCATCCCGATTGAGCTTCTTTATTTCGTTAGCTGCTCCAATGCGCCGTTCAAGTTGATACTTGTTGAGTTTATTGCCTTCATTGTTCTGTGTGGAATGACGCATTTGCTCATGGGCTGGACTTATTATGGTCAACACTCGTTTCATTTGATGCTTGCCCTTACCATTTTCAAAGTTCTCACGGCTTTGGTGTCTTTTGCTACTGCTATTACCCTCGTAACGCTTATTCCCTTGCTGCTCAAGGTGAAGGTACGAGAGTTTATGCTGAAAAAGAAGACTTGGGATCTTGGTCGAGAAGTTGGGATGATAAAGAAACAGAAAGAAGCGGGATGGCATGTCCGGATGCTCACCCGAGAGATACGCAAGTCACTTGATCGACACACAATTTTGTATACGACTCTTATAGAGCTATCGAAGACGCTGGATTTACATAATTGTGCCATATGGATGCCAAATGAGGAGAAAACGGAAATGGACCTCACTCATGAGGTGAGAGGGAGGAGCTTCTTAGATGGGCATAACTTCCCTATTCCGGTCCTCGATCCAGTCGTGCAGGAAATTAAGCAGAGCGTTGAAGTGAAACTACTCAACCCCGATACACCACTTGCTGTTGCAAGTAGCGGAGGGGTTTGTGAGCCAGGAAGTGTGGCTGCGATTCGGATGCCAATGCTGAGGGTCGCAAACTTTAAGGGTGGGACCCCTGAGCTTGTCCCGCAATGTTATGCTATCCTCGTTTTGGTGATTCCTGCTGGACAGGGTAGATGCTGGGGCAACCAGGAAATGGGAATAGTAAAGGTTGTAGCTGATCAAGTTGCTGTGGCAATTTCCCATGCTGCAGTGCTTGAGGAGGTCCAAAATATGCGGGATAAATTAGAAGAGCAAAACCGAGCTCTGCATCAAGCACAGCAAGATGCTTTGAGGGCGAGTCAGGCTAGGAATTCATTCCAGATGGTCATGAGCAATGGCATGAGAAGGCCTATGCACTCAATTTTGGGCCTTCTCTCTGTTTTGCAGGACGAGCAATTAAATTGTGAGCAGAAACTTCTTAGAGATACCTTGGCGAAGACCAGCAATGTCCTCTCGACTTTAATTAACGATGCAATGGATACATCAACAAAGCAAAACAGAAGATTCCAACTTGAGATGCGATCCTTTCAATTACATTCTATGATAAAAGAAGCCATCTGCCTTGCCAAGTGCCTTTGTACTTTTAAGGGTTACGAATTTGTGGTTGAAGTGGACAAATCTCTGCCTAATCATGTTATTGGCAATGAAATTAGGGTTTTTCAGGTAATCCTGCATATGGTTGGAAATCTACTGAAGAGCAATGGTGGAGGGTGCATCAAATTTTCTGTAACCCGTGAAAAGGATGGTCAGGGAGGAAACGATTTAGGATGGAGAACAAAGTCATCTAGTGAGCATGTTCATGTCAGGTTTGAAATTGGGATTGCCGGTAATTGTTCTAAGCCCGAGGGTGTTTACAAAGCCTCACATTGTAGTGAGGCATATGGCCGGAGGGAGGTTGAAGAAGTTTTAAGCTTCACCGTGTGCAAAAAGCTAGTTCAGGTATATTGGTCCTTATAGTAGCATCATATGAATGTGAAATAACCTGTTTCCCTTTTGTTCGAGTTTCATTTATCTCCATCTCTCTCGCATTGTTGAATTATGTTTCAGATTGTAAACTAACTTTTTGCTGGAAAATACAATATGCAGTTAATGCAAGGAAACATCTCGGTAGTGCCAAACCCTAAGGGCTTTCATCAAAGCATGGCAGTTGTTCTCGGCTTTCAACTCGGGCCATCCACTTCAGGCATGTCTGGGTGCAGCGAATCTTCCAGTCTTACACATCCGAGCTCCCTTTTGGCCGGGCTCAAAGTTCTGTTAGCCGACCATGATGGAATCAACAGGGGAGTAACCCGTAGGCTGCTCGAGAAACTGGGGTGCAATGTTTCTGCAGTTTCAACCGGATACGAATGTCTCGGGGCTCTAGGACCCGCAGCATGCCCATTCCAAGTTGTCCTTTTGGACCTTCACCTGCCTGAACTCGACGGTTTTGAAGTCACCATGAGAATTCGCAAGTTCAGAAGCCGAAGCTGGCCACTGATCATCGCTCTAACTGCAAATGACGATGAAGATGCGAGCGAAAGATGCATTCAGGTCGGGATGAATGGTATTATTCGTAA

>IbHKL5

ATGATGTTGAAGCTGCTAGCATCAGGACTGTTTATTTCGTCGTTCCTAATTGTGCTAGCAGCTGCCGATAATGGCGTTAGATGTAACTGTGATGATATCGAGGGAGTTTGGAGCATCGAGAGCATTTTGGAGTGCCAAAAAGTTAGCGACTTTTTGATTGCCGTGGCCTATTTTTCCATCCCGATTGAGCTTCTTTACTTCATTAGCTGCTCGAACATCCCGCTTAAGTTGGTGCTCTTTGAGTTTATTGCGTTCATCGTTCTGTGTGGGATGACTCATCTGCTTAGTGGTTGGACTTACTACGGTCAACATCCGTTTCAGCTTATGCTTGCCCTCACAGTTTTCAAAGTTCTAACGGCCATGGTCTCGTTTGCTACTGCTATAACGCTTATAACTTTCATCCCCTTGCTGCTGAAGGTGAAGGTGAGGGAAATAATGCTGAAGAAGAAGGCTCAGGATCTTGGGCGAGAAGTTGGAATGATAAAGAAGCAGAAAGAAGCAGGGTGGCATGTCCGGATGCTTACTCAAGAGATTCGAAAGTCACTTGATCGCCACACAATTCTGTATACGACACTGATTGAGCTATCCAAGACGTTGGATTTGTGTAATTGTGCGATTTGGATGCCTAATGTGGGAAAAACAGAAATGAATCTGACTCATGAGGTGAGGGGGAAGGACTTTTCGAATTTATACAACTACTCTATTCCAATCCTTGATCCGGATGTACAAGAAATCAAGAAGAGCGTTGGAGTGAAACTACTTGACCCTAATTCTGCACTTGCTGCTGCTAGTAGTGGAGGGACAAGCGAGCCCGGAGGTGTGGCTGCAATAAGGATGCCGATGCTGAGGGTTGCTAACTTTAAAGGTGGAACTCCCGAGCTTGTGCCTGCATGTTATGCAATTCTGGTGTTGGTGATTCCCGCTGGGCAGGGGAGATGTTGGGGCAACCAGGAGATTGCAATATTAAAGGTTGTAGCTGATCAGGTTGCGGTGGCGATTTCCCATGCTGCAGTGCTCGAAGAGTCTCAGCATATGAGGGAGAAATTAGTGGAGCAAAATCGATCACTACAACAAGCACAGAAGGATGCACTGAGGGCCAATCAGGCAAGAAATGGATTTCAAATGGTCATGAGCAATGGGATGAGAAGACCTATGCACTCGATTTCAGGCTTGCTCTCGATCTTGCAAGACGAGAAATTAAACAGAGAGCAAAAACTCCTTGTAGATGCAATGGCAAAAACCAGCAACGTCCTCTCAAACTTAGTAAATGACGTGATGGATACTTCGACAAAGGACAACGGAAAATTCCCACTAGACTTCAGGTCCTTTCAGCTACACTCTATGATAAAAGAAGCTGCCTGCCTTATCAAGTGCCTTTGTGCTTTTAAGGGTAATGATTTTGCTGTTGAAGTTGACCGATCCCTTCCCAATCGTGTTATGGGTGACGAAAGAAGAGTTTTTCAGGTTATTCTTCATGTGGTCGGGAATCTCTTGAAGATCAGCGGAGGAGGGTGCCTTAAATTTCGCGTAGTACCTGAAAAAGCAAGCCAGGGAGGAAACGATTTCCGATGGAAGACATGGAGATCAAACTCGTCTAGTGAAAATGTTTATATCAGGCTTGAAATTGGGATATGTAGTTATAAGTCTAGGACGGAAGGTGCAACATCGAACGTGTCCAGTCAGAAATATGGCAGTAGGGAGATCGAGGATGGCTTAAGCTTCAGCCTGTGCAGAAAGCTAGTTAAGGTATGTTACTATGTTTGCCATTTTTAATTGCACTATACATTTAATCGGGACTAGTGATAACTAGTCATCATGTATTTAGATTCACTTTCACATATAAGTTTACTTTCTACTTGGAACTTGTACAAACATTATGCAGTTAATGCAAGGAGAGATATGGATGGTCCCAAATTCGAAGGGATTTGATCAGAATGTAGCAATTATTCTTCCGTTTCAACTCAAGCCATCAATCGTTTTAGACATATTCGGGGAGTCCTCTAACTATACAAATCCGTATTTTCTGTTTGAGGGACTCGAAGTCTTGTTAGCCGACTACGATGATCTGAATAGAGCAGTAACTTGCAGGCTGCTCGAGAAACTTGGATGCATCGTTTCCACAGTTTCGTCTGGATACGATTGTCTCGGTGCTCTGGGCAATGGGGTATCCTCGTTTCAGGTAGTCCTTTTGGAGCTTAATTTACCCGATTTGGATGGCTTTGAATTAACCGTGAGAATCCGGAAGTTTCAAAGCCGTGGCTTCCCACTTATCATTGCTCTAACAGCAAGCAGCGATGAAGATGTGATCGGGAGATGCTTGCAAGTTGGAATGAATGGTATTATTCGTAAACCCGTTCTTTTGCAAGGAATTGCTGACGAGCTTCAACGAGTCCTATTACTCACAAACAGAATCATATCACCTCGAGAATGAATGATTCACTGGAGTGGTCCATTGCACGACCCGGAATTATGAAGGTCAAAATGGTATTTGGAACACAAAACAGTTCCACCACCAAGAATCCAACACAGACATGCTGCATAGCACCGCACAGGATCAAATCGGTATAGAATATGCACTTCCAATTTTTTTTTTTCTTTTTTTTTTGCTCTTGTGTCATAATCTTCAATCTTGATATTCAATTAGCAGTTCATTCTATGCATCTTCATACTTATCACCCAACTCCTTCTGTCTAGCGATCACTTTAAGTATGTACAGTCGATGAGTCCATGAATACATAACAAGGAACATCATAGCACACTACCAAGGCACTACCGAGAAACTGTAAATTGCTCACTTGTTGTTAGGAGCCAGTTTTGTTTAACTTCTACCCAGCCTGAATATGCATTGTGTTATATACATGACTCTCTATTATTGTGATCTTCAGATTATATAATGTATATATACTTGCACTACTCTGTAACTGTAGTTTTCTTGTGTTATCACATTATTATTGTTGGGCGGAGGCCTGCGAAGGGCCAAAGTCAGCATCCTGCCTCGCCTTCGCTATTGGCTATAGCTTTTGGCTAACAAGTGGTATCAGAGTCAGGTTATGGGTTCGAGTGACAAACGCTATTATGAGGCTGGGACCACTATGCCAAACGCTATAACCAGTAGCAAAGGTGCACCTTTATTTCCTTATTATGAGGCTAGTGGTGGACTTGGCCCTTCACCCTTCACCCCTAGACTGCCCTAA

>IbHKL4

ATGTCGGTGTTGAAGCTGTTGGTTTTGTCTGTCCACATTGCGATGGCAGCAGCCGATAATGGCTTTGCTCGGTGTAATTGTGAGTACGAGGGGTTTTGGAGCATTGAGAACATTTTGGAGTGCCAAAAAGTTAGCGACTTTTTGATTGCGGTGGCGTATTTTTCCATCCCAATTGAGCTCATTTACTTCATCAGCTGCTCGAATGTTCCGTTCAAATTGGTGCTCTTTGAGTTTATCGCGTTCATTGTGCTCTGCGGGATGACCCATCTGCTCAACGGTTGGACTTATTACGGCCAACACTCGTTTCAGCTTATGCTTGCCCTTACGGTTTTCAAAGTTCTCACTGCCATGGTCTCGTTTGCTACTGCCATAACCCTTATCAGCCTCATCCCGTTGCTGCTCAAGGTGAAGGTGAGAGAGCTTATGCTCAAGAAGAAGGCGTGGGATCTTGGTCGGGAAGTTGGGCTAATAAAGAAGCAGAGAGAAGCGGGATGGCATGTCCGGATGCTCACCCAAGAAATCCGCAAGTCACTTGATCGAGACACAATACTCGAGACGACTCTGAGCGAGCTGTCGAAGACGTTGGGTTTACATAACTGCGCTATTTGGATGCCCAATCAGGACAGAACAGTAATGAACCTGACTCACGAGGTGAGGGAGAGGAACTTTTCGGACATGAATGACTTCTTGATTCCAATACTCGACACCGATGTGCAAGAAATTAAGGCCAGCGATGAAGTGAAACTACTCGAGCCTTCGTCGCCTCTCGCTGCTGCGAGTAGTGGAAGGAGTAGCGAGCCAGGATGCGTGGCTGCGATAAGGATGCCGATGCTGAGGGTTGCTAATTTTAAAGGCGGAACGCCCGAGCTTGTCCCGGCTTGTTATGCTATTCTGGTTTTAGTCCTCCCGTCTGGACAGGGCCGATCTTGGGGCAGCCAGGAGATTGAAATAGTAAAGGTCGTTGCCAATCAGGTTACCGTGGCTATCTCCCACGCTGCCGTGCTCGAGGAGTCTCAGCATATGAGGGACAAATTAGCGGAGCAAAATCGAGAACTGCAGCAAGCGCAGCAGGGCGCACTGAGGGCGAATCAAGCAAGAAACGCGTTTCAGATGGTCATGAGCAACGGGATGAGAAGGCCTATGCACTCGATCTTTGGCCTTCTCTCGATCTTGCAGGAGGACGAGAACTTAAACAGCGAGCAGCATCTTCTTATAAACGCAACGGTGAAAACCAGCAATGTTATCTCGAACCTAATAACCGACGTGATGGATTGCTCAACAAAAGACAACCGAAAGTTCCCACTCGAGACTCGGTGCTTCGAGCTACATTCTATGATAAAAGAGGCGGTCTGTGTTGCCAAGTGCATCTGTGCTTACAAGGGTTACGAATTCTCCGTTGAAGTCGACAAATCCCTGCCTAATCACGTTATGGGAGACGAAAGACGAGCTTTCCAGGTTATTCTCCATGTAGTCGGGAATCTCTTGAAGAACAGTAACGGAGGGTGCCTTAAATTTCACGTAGTGCCCGAAAGGTCTAGTCAGGGAGGAAACGATTTAGGTTGGAGAACTTGGCGATCAAACTCATCCCGTGAGAATGTCTTTGTCAGATTTGAAATCGGGATACATGGAAACAATTCTCAGCCCGATCACACAACATCCAAAGTCTTGAATTCTAATCAGAAATATTGTGGAAAGGATTTCGAGGGAAGCTTGAGCTTCAGTGTCTGCAAAAAGCTAGTTCAGGTATGTATCTTTCCCGTTATAACAATATTCTCGTACTGCTGTACTAATTGTTGCAAGATCCTTTCTTCTTGTGTAAACTAAGATTACCCGATCAATATGCCGATTCATATGTGTTGAGCATATATAATATATAAACATAAATGTGCAATCCAATTATCAGCTTAAGCTTTTAGTTGAGATGGAACACATATTTGAATTTGGTATCAGAGCCAACCCCATGCCAAAGGTCATGGGTTCGTTTTTAATTAACTGCCATAGTTTTAACTTTATTTATGCGTGTGCAGTTAATGCAAGGAGACATCTGGGTATCCCCAAATCCGATGGGGTTTGATCAACAAGTCATGGCTGTTGTTCTAGGATTTCAACTCAGACCCTCGGTCGTCATAGGCATATCGGAATACGGTGATTCTTCCAACCGGACACATTCGGATTCTCTTTTCCGGGCTCAATATTCTGTTAGCTGACTACGATGATGTGAATAGAGCAGTAACCCGCCGTATGCTAGAGAAACTCGGATGCATCGTTTCTTCGGTTTCATCTGGATACGAATGCTTAGGCTGTCTCGGCACTACAATATCCCCGTTCCAAATCGTGCTTTTGGATCTCCACCTACCCGATCTGGATGGGTTTGAAGTCACCATGAGAATCCGAAAGTTCAAAAGCCGTAATTGGCCACTGATCGTTGCCCTAACGTCAAACAACGATGCCAGCATCCGTGGAAGATGCTTTCAGGTTGGGATGAATGGCGTCATCTGTAAACCGCTATTCTTGCAGGGAATTGCAGACGAGCTTCAGAAAGTTATGTTAATTGCGAGCCGAACCCTATCTTGA

>IbHKL3

ATGTCGAAGATATTAGCATTGAGGGTGTTGGTTTGGGTTTCCCTTGTTGCATTCACAGTTGCTGATAATGGGTTCTTCAGATGCAACTGTGATTATGATGGATTTTGGAGTATCGAGACGATTATGGAGTGGCAAAAAGTGGGCGACTTCTTGATTGCAGTGGCCTATTTTTCCATCCCGATTGAGCTTCTTTATTTCGTTAGCTGCTCCAATGCGCCGTTCAAGTTGATACTTGTTGAGTTTATTGCCTTCATTGTTCTGTGTGGAATGACGCATTTGCTCATGGGCTGGACTTATTATGGTCAACACTCGTTTCATTTGATGCTTGCCCTTACCATTTTCAAAGTTCTCACGGCTTTGGTGTCTTTTGCTACTGCTATTACCCTCGTAACGCTTATTCCCTTGCTGCTCAAGGTGAAGGTACGAGAGTTTATGCTGAAAAAGAAGACTTGGGATCTTGGTCGAGAAGTTGGGATGATAAAGAAACAGAAAGAAGCGGGATGGCATGTCCGGATGCTCACCCGAGAGATACGCAAGTCACTTGATCGACACACAATTTTGTATACGACTCTTATAGAGCTATCGAAGACGCTGGATTTACATAATTGTGCCATATGGATGCCAAATGAGGAGAAAACGGAAATGGACCTCACTCATGAGGTGAGAGGGAGGAGCTTCTTAGATGGGCATAACTTCCCTATTCCGGTCCTCGATCCAGTCGTGCAGGAAATTAAGCAGAGCGTTGAAGTGAAACTACTCAACCCCGATACACCACTTGCTGTTGCAAGTAGCGGAGGGGTTTGTGAGCCAGGAAGTGTGGCTGCGATTCGGATGCCAATGCTGAGGGTCGCAAACTTTAAGGGTGGGACCCCTGAGCTTGTCCCGCAATGTTATGCTATCCTCGTTTTGGTGATTCCTGCTGGACAGGGTAGATGCTGGGGCAACCAGGAAATGGGAATAGTAAAGGTTGTAGCTGATCAAGTTGCTGTGGCAATTTCCCATGCTGCAGTGCTTGAGGAGGTCCAAAATATGCGGGATAAATTAGAAGAGCAAAACCGAGCTCTGCATCAAGCACAGCAAGATGCTTTGAGGGCGAGTCAGGCTAGGAATTCATTCCAGATGGTCATGAGCAATGGCATGAGAAGGCCTATGCACTCAATTTTGGGCCTTCTCTCTGTTTTGCAGGACGAGCAATTAAATTGTGAGCAGAAACTTCTTAGAGATACCTTGGCGAAGACCAGCAATGTCCTCTCGACTTTAATTAACGATGCAATGGATACATCAACAAAGCAAAACAGAAGATTCCAACTTGAGATGCGATCCTTTCAATTACATTCTATGATAAAAGAAGCCATCTGCCTTGCCAAGTGCCTTTGTACTTTTAAGGGTTACGAATTTGTGGTTGAAGTGGACAAATCTCTGCCTAATCATGTTATTGGCAATGAAATTAGGGTTTTTCAGGTAATCCTGCATATGGTTGGAAATCTACTGAAGAGCAATGGTGGAGGGTGCATCAAATTTTCTGTAACCCGTGAAAAGGATGGTCAGGGAGGAAACGATTTAGGATGGAGAACAAAGTCATCTAGTGAGCATGTTCATGTCAGGTTTGAAATTGGGATTGCCGGTAATTGTTCTAAGCCCGAGGGTGTTTACAAAGCCTCACATTGTAGTGAGGCATATGGCCGGAGGGAGGTTGAAGAAGTTTTAAGCTTCACCGTGTGCAAAAAGCTAGTTCAGGTATATTGGTCCTTATAGTAGCATCATATGAATGTGAAATAACCTGTTTCCCTTTTGTTCGAGTTTCATTTATCTCCATCTCTCTCGCATTGTTGAATTATGTTTCAGATTGTAAACTAACTTTTTGCTGGAAAATACAATATGCAGTTAATGCAAGGAAACATCTCGGTAGTGCCAAACCCTAAGGGCTTTCATCAAAGCATGGCAGTTGTTCTCGGCTTTCAACTCGGGCCATCCACTTCAGGCATGTCTGGGTGCAGCGAATCTTCCAGTCTTACACATCCGAGCTCCCTTTTGGCCGGGCTCAAAGTTCTGTTAGCCGACCATGATGGAATCAACAGGGGAGTAACCCGTAGGCTGCTCGAGAAACTGGGGTGCAATGTTTCTGCAGTTTCAACCGGATACGAATGTCTCGGGGCTCTAGGACCCGCAGCATGCCCATTCCAAGTTGTCCTTTTGGACCTTCACCTGCCTGAACTCGACGGTTTTGAAGTCACCATGAGAATTCGCAAGTTCAGAAGCCGAAGCTGGCCACTGATCATCGCTCTAACTGCAAATGACGATGAAGATGCGAGCGAAAGATGCATTCAGGTCGGGATGAATGGTATTATTCGTAAACCGGTTATTTTGCAAGGAATTGCTGATGAACTTACCAGAGTCCTGTTGCTAAAAAGCAGAAACATTGCATGA

>IbHKL1

ATGGGTGCTGCAATGTTGAGGTGGTTGTTTCTTGGGTTGTTAGTTTCTTCGATTTTCTCCGCGGTGTCAGCTATTGATTATCTCTGTTGTGATGACGAGGGATTGTTTAGTGTAAGTAACATTCTATTTATGCAGAAAGTGGGCGACGTCTTGATTGCAGTTGCTTATTTCTCGATTCCTATTGAATTGCTGTACTTCATTAGCTGCTCGAACATACCTTTCAAATGGGTGCTCGTTCAATTCATTGCGTTCATAGTGCTTTGTGGATTGACCCATTTGCTCAATGTGTGGACTATCAACACTCAGCCTTCCTTTCAGATGATAATGTCATTGACAGTCGCAAAAATCCTGACTGCACTCGTGTCCTGTGCAACTGCAATCACCCTTCTCACGCTTATCCCGCTTCTTCTCAAATTTAAGGTGAGGGAACTGTTTTTGAGACAGAATGTGTTGGAGCTGGATCAAGAGGTCGGGATGATGAAGAAACAGAAAGAAGCGAGTATGCATGTCCGAATGCTGACACTAGAAATTAGAAAGTCGCTTGATAAGCATACTATACTCTATACCACTCTGGTCGAGCTTTCTAAAACGTTGAATCTTCAGAACTGTGCAGTGTGGATGCCAAGTGGGAACAGAGCAGAGATGAACTTGACGCACGAGTTGAACCCCTGTTCGGCTAGAGAGCATCATTCCCTTTCAATTAATGACCCGGATGTGCTGGAGATAACAAAGAATGAAGGGGTGAGGTTACTGAAGCAAGATTCAGTTCTTGCAGCTGCGAGCAGTGGTGGGTCTGGTCAACCCGGTGCTGTTGCAGCTATTCGGATGCCATTGCTTCGTGGTTCAAACTTCAAAGGTGGAACGCCAGAGCTTATTGAAACGTGTTTTGCCATATTAGTGCTGGTTTTTCCCAGCGTTAATGATGGTGATTGGAGCTATGATGAGTTGGAGATAGTGGAAGTGGTTGCTGATCAGGTGGCCCGTGGCTCTGTCCCATGCAACAGTTCTTGAAGAGTCTCAGTCGATGCAGGAGAAGCTGAAAGAAAGAAACCGTGTATTGCAACAAGCTAAGGAGGATGCTATGAAGGCTAGCCAGGCAAGGAATTCGTTCCAGAAGGTAATGAACAATGGAATGAGGCGACCAATGCACTCGATTTTGGGCTTGCTTTCCATACTCCAAGATGACAATTTGAAACCCGAGCAGAAAATTGTCGTCGACACATTGGTGAAAACCAGCACGGTACTTTCAACCTTAATTAGTGATGCAATGGAGATATCTGCCAAAGATGACGGGAAATTCCCTGTCGAAATGAGGCCCTTTCAACTTCATTCATTGATCAGGGAGGCCTCTTGTCTCGTGAAATGCTTTGCCATTTATAAGGGCTTTGATTTTTCCACAGATGTTCTGAGTTCTTTGCCTAATCAGGTGATGGGTGACGAGAAGAGAACATTTCAGGTTATACTTCATATGGTTGGGCATTTATTCAATGTCAGTGATGGAAACGGCTCCGTCATATTCAGAGTTGCTTCGGAAAGTGGAACTGAGGATGGGAATAATAAAGTTTGGAATACGAGAAAACCGAGCTCGAGTGATGACAATGTAACAATAAAATTTGAAATTGAAGTCACTATTGGAGATTCTCAATCAGGTACCTCGGTTTCAGTCGTTCCTTCTGGCAGGAAAAGGCATAACAGCAAAGATGTGAAGGAGGGCTTGAGCTTCACCATGTGCAAAAAGCTTGTGCAGGTAATTAGTTTTTATTTGCTCTCCTTAGTTTGTCGTAATTTCGGGTATTTTTAATTTTGGGGGGGAAGGGGCGGGATTGGCTTTCGGTTGGTGCTTGGGAAGAGGCTCGATTCATGTAAAAGGTTTTGGTTTTGAGTTTGTAAGTCTTACAACCCAAGTCACGGGGTCGGATTAACTCAGGAAAACAGAATAAATTGAAGAAGAAAGCTTAGGGATAGAAAAGTGAAGGTTCAGGGAAGAGAGCAGAATAGAAGAGAGAGAGAAAGATTTTAGATATGCTATTCAATAATTGCCATCCATCCATCAGTTAGCTTAACAGCCTATTTATACATCTCATTACGTAAGAATTTCATCAAACTAAGAAATTTCCAGAAACACAACTGTGTATTTTGCTGAAACGTGTAGTTGCTCAGTGGCAAAAGTGCATTTTTTTTAACACTTCATAACAGCTTTAACGGCAACTTCAAACGTCACTGTTTGAGTCAACAACTATTGGAAATTTAACCAATTGACATCACACTAAAACGCACATTATTTATTTAAAAACAACCTGAGCAACACTGTTTCTCTCCCTAGTTTATTGTTCTTCATTCTTTATTCTTCTTGCTACTAGGGTCATTACTTGAGGTAAGAAGAGAGTCGGGATCTTAAGGCAGATAAATGTGTAAGGACTTGAGTTCTATCTATATCTCTAGGTTTCTAAATTTTCAGATACTTCTTACAGTTATAAAAACTAATCTGAATCGAGCAATGTAAATCTTACAATCTTTGAGGTGATTCTCTGTCTCTCCATGAAATTCATATTGCAATGCTTATTAATGCTCATTCCTTTCAACTGTCTTGAGTAGACTTTGAAAACGGTATCAATAAAAAAAATTATTTACTACTCTGTTGAATGAAAATCTATACCAAAATATGAATGTGAGCTCTGAAAAGAGACATAATGTCACTTATTATGCCACTTATTTGCCCTTTAAGCGTGATTTGAAATAACTGCTTTTATGTGTGATTATGGATGATTTGCAAATAATCAAAGTAAATAAAAGGCTATGTTTGGCATACCTAGCTTATTAGCTAGCTTAAAGCTGAAAGCTTAAAAGCTAATAGCTACTAGCTTATTTTGAAACGCTATTTAAGATAGTGTTTCAAAATAAGCTACTCCGTAATATTTTAAGTTCATGTTTTACCAAACAGAGCGTATAGCTTATTTGTAGCTTAAAATAAGCTCTACCAAACATAGCTAAAGTAGAGTGATAGTTTTTATAGTGGTTCAGGATTTTCCCTACTCCTCATCCCCTTTTTGTTTCTCTTTACTGTCATTTTCGTATATTTCTTTGTGTCTTGTAGGAAACAACCATTTTTTCCTTAATATTTTGATCCTCTAGTCTTGATTGTGACTTCATACTGCATCCCAAAGTGTTAAGTTACTGAAATACAACAATGTTCCTTCTCTTTTTAAAAACTTTTGGAGTTCCCATTGAAATTTGTTTGAACTACTTGGACATGCCTTTCCACGCCACGTTTTCAGAATGTGTATTTCGCATGCATTGTCATTACATGTTCAAGCAATTCAGACGAATTTCAGCTTGAAACCAGGGAATTCTTTAGAAAGAGAAGACATAATGTCGCATTATAGGGATGCAATGCATTCTGGAGATGCATATGATGTCACATTTAGGAATTTGATATAATAAACATGACATCATGTTGCATTTACATAACGTGTGGTTCACATTTTAATATAGCTTTTCTTAAGAATGCCGATTTAGTAAATGATTTCTGCTGGCGGAAGTTTTTTGTTTACATTTTACCTGCAAAATTTGAATAGTTGGTCTTAATTTTACGTTAAGCTAGCTTAACGTGTGATTATTGTTTGACCTATGGTGCTTGTTGCAGTTGATGCAAGGAAATATATGGGTCTCCTCAAATTCTCGGGGCCGTGGACAAGGCATGACGCTGATTCTAAGATATCAGAAGCAATCCTCGATTAGAAGACGAATCTTTGAATACAGAAATCCTTCGGAGCAACCGCTTCCAAGCACAATGTTTGAAGGCCTACAAGTTCTTCTGGCTGACGATGACGGCGTAAATAGAATGGTGACTAAGAAGCTGCTCGAGAAGTTACGCTGCCAAGTATCCACGGTTTCGACGGGTTTCGAATGTCTAAGCGCCCTAGGCCCCTCGGCAACGTCCTTCCAAGTCATCATCCTAGATCTCCACATGCCGGAAATGGACGGGTTCGAAGTGGCGATGAGAGTGCGCAAGTTTCGCAGCCGCAACTGGCCGTTGATCATAGCCCTGACCGCGAGCTCGGAAGACCACATGTGGGAACGATGCCTCCAGGTGGGGATGAACGGTCTGATACGAAAACCCGTCCTCCTACAGAGACTCGCGGAAGAACTTCAGAGAGTCCTCCAGCGGGCTGGCACCGAAGTCATGTGA

>IbHKL6

ATGTCAACTTCCAGACCTAGTCAGTCTTCTAGTAATTCAGCGAGATCAAAACATAGTGCTAGGATCATAGCACAAACCTCTATAGATGCAAAGCTCCACGCGGAGTTTGAGGAATCGGGGGATTCCTTTGATTACTCTAGCTCAGTCCGAGTCACGAGTGTGGATGCTGGAGTGCAGAAACCTAGGTCTGACAAAGTAACCACTGCTTATCTCCATCAGATCCAAAAAGCCAAATACATCCAGCCATTTGGCTGTTTGTTAGCGCTGGATGAGAAAACTTTTAAAGTCATAGCATTCAGTGAAAATGCTCCCGAGATGCTTACCATGGTCAGCCATGCTGTTCCAAGTGTTGGAGATCATCCAGTTCTTGGTATTGGGACTGATATTAGAACCATTTTTTACCAGTCCCAGTGCAGCAGCATTACAGAAGGCCTTGGGATTTGGAGAGGTTTCTCTGTTAAATCCTATCCTTGTTCACTGCAAAACCTCTGGGAAGCCATTTTATGCCATTATCCATAGGGTAACAGGTAGCTTGATTGTTGACTTTGAGCCAGTCAAACCATATGAAGTACCCATGACTGCTGCTGGTGCATTGCAATCGTATAAACTTGCAGCAAAAGCTATTGCTCGATTGCAGTCCTTGCCAAGTGGCAGCATGGAGAGGCTCTGTGATACTATGGTCCAAGAGGTGTTCGAACTCACAGGTTATGATAGGGTGATGATTTATAAATTTCATGATGATGACCACGGGGAGGTTGTTTCTGAGATCACTAAGCCTGGCCTTGAACCTTACTTGGGCTTGCATTATCCAGCAACCGATATTCCTCAAGCTGCACGCTTTTTGTTCATGAAGAATAAAGTACGGATGATTTGTGATTGCCGAGCAAAACATGTCAGGGTGGTCCAAGATGAGAAACTTTCGATTGATTTAACATTGTGTGGTTCCACACTTAGGGCTCCTCACAGCTGTCATTTACAGTACATGGAGAATATGAATTCAATAGCGTCACTGGTAATGGCAGTCGTAGTTAATGATGGAGATGACGAAGGGGAGGCCTCAGAATCTGGACGGATACAGAAGAGGAAAAGGCTTTGGGGCCTAGTTGTATGCCATAACACGACTCCCAAGGTTTGTTCCTTTTCCTCTTCGGTATGCATGTGAGTTCCTTGCACAGGTGTTCGCCATTCATGTCAACAAGGAACTAGAATTAGAAAACCAGATTGTTGAGAAAAACATTCTACGCACTCAAACTCTCTTGTGTGATATGTTAATGCGAGATGCGCCACTGGGTATTGTGTCACAGAGCCCCAATATAATGGATCTTATCAAATGTGATGGCGCTGCTTTGCTGTATAAGAGTAAGGTACACAGATTGGGAATTACCCCAACCGACTTTCAGTTGCATGATATAGTCTCTTGGCTCTCCGAGTACCATATGGATTCCACAGGTCTGAGCACAGATAGCTTGTACGATGCTGGCTTCCAAGGGGCTCTTGCCCTTGGTGATGCAATATGCGGGATGGCTTCTGTCAGAATATCTGACAAGGATTGGCTTTTCTGGTTCAGATCGCACACTGCTGCTGAAGTTCGTTGGGGTGGTGCGAAGCACGAACCTGATGAGAAGGATGATGGTAGGAAAATGCATCCAAGGTCATCGTTTAAAGCGTTTCTTGAAGTTGTCAAGACAAGAAGTTTACCTTGGAAGGACTACGAGATGGATGCGATTCATTCGTTGCAGCTAATACTGAGAAATGCTTTCGGCAAGGAGGCTGATACTATGGATACAAAAGCAAATGCAAATGCTATCCATTCAAAGCTTAATGACCTAAGAATTGACGGTATGCAAGAACTAGAAGCCGTAACAAGTGAAATGGTCCGCCTGATTGAAACGGCCACAGTGCCAATCTTGGCAGTTGATGTGGATGGGCTGGTGAATGGGTGGAACACAAAAATTGCTGAGTTGACTGGTCTAACTGTCGATGAAGCAATTGGGAAGCACTTTCTTACACTCGTGGAAGACTCCTCAGTTCATAATGTGAGAAAGATGTTGAGTTTGGCGTTGCAGGGTATGTATTTAAGTATTATCCCTCTGATGCATTTAATGTATTTTAATTGATCAAGGAGTTATTATATATAGAATATAAATAAATAATGTAAACTCCATCTATTATCTCCAAATCACTACTTAATCGGCAACATTTGTGTCTTCCCCAGTATATTCTCTTAACTTTTCCAATGCCATCTTTTCCAGGGAAAGAAGAAAAAAATGTACAGTTTGAGATAAAAACACATGGGCAAAGATCAGAATCTGGTCCAATCAGCTTAATTGTGAATGCTTGTGCAAGCAGGGATGTTCAAGAAAGTGTGGTCGGTGTCTGTTTTATTGCACAGGATATAACTGGACAGAAGACTATTATGGACAAGTTCACACGAATTGAAGGGGATTACAGGGCTATTATACAAAATCCTAACCCTTTGATTCCCCCAATATTTGGAACCGATGAATTTGGGTGGTGTTCTGAGTGGAATTCAGCTATGACAAATTTATCTGGATGGTGCCGTGATGAAGTTATGGACAAGATGCTTCTGGGGGAGGTTTTCGGGACACAGAAAGCCTGCTGTCGTCTCAAGAATCAAGAGGCTTTTGTAAATCTGGGCGTTGTACTGAACAACGCTATAACTGGTCAAGTGTCTGAAAAGACCAGGTTTGGTTTCTTTGCACGAAATGGGAAATATGTGGAGTGCCTACTTTCTGTGAGCAAAAGATTGGACCAAGAGGGAGCCGTCACGGGGCTATTTTGTTTCCTGCAGTTAGCAAGCCAGGAACTGCAACAGGCCCTTCATTTTCAAAAATTGTCCGAGCAAACTGCAATGAAGAGGTTGAAAGTGTTGGCGTACATAAGAAGGCAGGTCAAAAAACCCTCTTTCGGGAATCATGTTCTCTCGAAAGATGCTAGAGGGGACGGAGTTGGGTAAAGATCAGAAAAGCATATTGCATACTAGTGCCCAGTGTCAGCAGCAACTCAGTAAAGTTCTTGATGACACAGATCTTGACTGCATCATCGAGGGGTACGATGAAATAAGCGATTTACAATTATTTTTTTTTTAGTTTAACTATACCATGAAAAAATTTCACCTTTTACTCTTTTCAAATTTCTTCTATTCAGTCTCTCTTAATCAATTTATTTTTTGGATGATGAGGGAAACCCCACAACCCTTTTCGAGTGTAAAGCCTTGTGACCCTATCTGACAAAGAACCACAAGGAAGTAAACCAGCCTAAGTTACCAATAGCTGACTGGCTCAAACAAAGGGGCAAGGATTCGAACTCGTGACCTTTGTGGTTACAAGCGGGGATCTCTTGCCATCTTGGATGGGGTTATCCCCTCTTAATCAATTTATTTCATTCTACTATTGGTAGTTAATGCTTTAGTTTATCTGTTATTCAGTTTATCTGTTATTCTTAAATGCAGGTATCTGGATCTGGAAATGGTTGAATTTAAGCTAGATGAAGTGTTACAAGCTTCAATTAGTCAAGTAATGACAAAGAGCAATGGAAAGAGTTTAAGGATAATTAATGACATAGCTGACAATATTCTTTGTGAAACTTTATATGGAGATAGTCTGAGGCTTCAACAAATCCTCTCTGAATTTTTGTCAGTTGCTGTGAATTTTACCCCGGGCGGAGGCCAGCTTGCTCTTTCATCCAAGTTGACTAAAGATAATTTGGGAGAATCTATTCAGCTTGCCCATTTGGAATTCAGGTACCACTGCGACTATTTGGTAATATTTACATAATTATATTATTGAAATTATAGACTAATGATCCATGCACTTTATAAATGTTTGGCAAAGAATAAAATGAATTATATAGCTTGTGCAGCTCTTTTCAGCTTATTTAAGCTATATAATAATCCATCTGTGCTTTAGAGTTGATAATCTTGCTAAACAAGACTTAATTGAACGATATGCTCCGTCTTAACTAAAAGTTTATCTGATAGTAAGACTATTCATTTATATTTTATATATTATATGCTCAGTGGATCTCTACTTAGTGACAACTCAAGTAAAGGGGCACATGAAGTTCCCTTTTACCAGTTCTGCTCATTGTTGTTAAGGCAGACTTTCAAGGCGGGGTTAATTTTATTATGATTAAAAATTTGAAAGTTTAGTAATAATAAGATCCTAGCCTTTGATTTTTCAAGATGGAAGGTGTGGATGTGTCCACACTTTTTACAGGGTTACAGGGAAGATATTCTCACCTCAACCCTCCTATATATATATATATATATATATATATATATCAATTCATAATTTAGCATTTTAGCATATAAACATATTCTCAGATCTCAACAGAAGTACTAAAGCATTTCCAGCATAAGCCTTTAGATTTAGCCTTCCTTTTAAGATCCATTGATGGATCATAATTGGGGTTATTTTCATTCTCAGCCATAACCTGTCATAAAACAAAGTAAACAACCACTAAGGCGAGGTTTTGGGGGGGGTGGGGGTGGAGTTGTCTTAGGCTCTCAGCTATGTATTAGTAGGGTTTTCTTTTTAAAGCGAGGCGAGCTTGTTCGGCGACCGATCTCCATTGACTGGACGCCTTGATGATGCGTGATGCCTTAGCAACAATGGGTCTGCTCACAAGTGCATGTTGGATTAGTTACCAAATATGACCGACCCATGGGCATTTGGAAGGTGGGGAAGTAGCTGTTCATACTATACTCATAACCATTTGCGATATTATGGTTGCAGGCTAACGCATACAGGCGGTGGGGTGCCAGAAGAGCTGCTGACCCAAATGTTTGGTAGCGAAGCAGACGCATCAGAGGACGGGATCAGCCTGCTTATAAGCAGAAAGCTGGTGAAGCTCATGAACGGGGACGTTCAGTACCTCAGGGAGGCGGGTCGATCCACCTTCATCATATCTGTCGAACTTGCAGTTGCTTCTAAACCTTCTTCGTGA

>IbHKL9

ATGGCGTCAGGGAGCAGGTCCAAAAACGTGCAGCAAAATCAAGCTCAATCTTCGGGTACAAGTAATGTTAATTATCGCGATTCAGTGAGCAAAGCTGTTGCGCAGTACACTGTGGATGCTAGGTTGCACGCTGTTTTCGAGCAGTCGGGAGAGTCTGGCAAGTCTTTCGATTACTCCCAATCTGTGAAGACTATTACTCAGAACGTTCCCGAGAAGCAAATCACCGCCTACTTGTCCAAAATTCAGAGAGGGGGTCACATTCAACCCTTTGGGTGTATGATTGCGGTGGACGAGCCCAGTTTTCGGGTAATTGGCTATAGCGAGAATGCCCGCGAAATGCTTGGTTTAACGCCCCAGTCAGTTCCCAGCCTCGAGAGGCCTGAAATCCTCGCGATTGGGACGGATGTGAGGACCCTCTTTACGCCCTCCAGCTCCGTTTTGCTTGAACGCGCCTTTGGAGCGCGAGAGATCACCTTGCTCAACCCAATTTGGATTCATTCTAAGAATTCCGGCAAGCCCTTTTACGCCATTTTGCATAGGATTGATGTTGGAATTGTGATTGACTTGGAGCCTGCTAGGACTGAGGACCCTGCATTGTCTATTGCTGGCGCTGTGCAGTCTCAGAAGCTCGCTGTGAGGGCAATTTCCCACTTGCAGTCACTTCCCGGCGGGGATATTAAGCTTTTGTGTGATACTGTGGTTGAGAGTGTGAGGGAGCTAACCGGGTATGATCGAGTCATGGTGTATAAGTTTCATGAGGATGAGCATGGGGAGGTTGTGGCCGAGAGCAAAAGGCCTGATTTAGAGCCCTATATTGGATTACACTATCCTGCCACTGATATTCCTCAAGCTTCCAGGTTTTTGTTTAAACAGAATAGGGTTAGGATGATAGTTGATTGCAATGCCACTCCGGTGCAGGTTATTCAGGATGAATCACTGATGCAACCACTGTGTTTAGTTGGATCAACCCTTCGTGCTCCTCATGGCTGCCATGCACAGTACATGGCCAATATGGGCTCCATTGCTTCGTTAACACTCGCAGTTGTTATTAACGGTAGTGATGAGGAAGCTGTAGGAGGGAGAAACTCAATGAGGCTATGGGGGTTGGTTGTTGGGCATCACACTTCTGCCAGGTGCATCCCTTTCCCCCTCCGTTATGCTTGTGAATTTCTTATGCAGGCGTTTGGTCTGCAGTTGAACATGGAACTGCAATTGGCATCACAATTGTCTGAGAAGCATGTCCTAAGGACGCAAACGCTTTTGTGTGACATGCTTTTGCGAGATGCCCCCATTGGCATTATTACCCAGAGCCCCAGCATTATGGATCTTGTCAAATGTGATGGAGCTGCACTATACTACCAAGGGAAATATTATCCTTTAGGTGTGACACCTAATGAAGCCCAGATAAAGGAAATAGTTGACTGGCTATTGACTTACCATGGTGACTCAACTGGGTTGAGCACAGATAGTTTAGGTGATGCAGGGTATCCTGGTGCAGCTTCACTCGGTGATGCAGTTTGTGGGATGGCAGTTGCTTATATAACTTCAAGAGATTTCTTGTTCTGGTTCCGGTCTCACACTGCAAAAGAGATTAAGTGGGGTGGTGCTAAGCATCATCCAGAGGACAAAGATGATGGTCAGAGGATGCATCCGCGCTCTTCGTTCAAGGCATTTTTGGAAGTTGTTAAGAGTCGCAGTTTGCTTTGGGAGAATGCTGAAATGGATGCAATTCACTCTTTGCAGCTTATCCTACGTGATTCATTTAAGGATGCTGAGGCAAGCAATTCTAAGGCTGTTGTGCGTGCTCCGCCTGGAGGATTGGAGTTGCAAGGAATGGATGAACTGAGTTCTGTTGCTAGAGAAATGGTTAGATTGATAGAGACTGCTACAGCTCCCATATTTGCTGTAGATGTTGAAGGGCGCATAAATGGGTGGAATGCAAAAGTTGCTGAGTTGGTAGGCTTGTCGGTTGAAGAAGCTATGGGAAAGTTGTTGATTCAAGATTTAGTTCACAAGGAATCACAAGAAACTACCGAGAAGCTTCTGTTTAATGCTTTAAGAGGTAATTGCTTTCTGTTCTATTTTGTTCTGTGCTTGTTTTCATTTTGCTATTATATAAAAAGTAATCCTTCTACAGTTGCATAAGAGGTTAGCCTTGACATGCTAGTTGGTAAGTAGGTTTTATGATGGCCAGAGTTAGTCTTTACTTTGAACTTCTGTTAAGCCATTCACAATTAAAGCATGTAGTGATCCTTATCCTTTGGATTTTGATGTGACAAACTGAGTTATAAGATACATAAATTACATGAGTATTAATTAAGTTCAATGTATGTTTTCCAGAAAGTCCCAATGGCTATCTTCTTGCATGATAATATAAGAAAGTTGACTGTTCATAGACCTTAATCCTATTGCTAAATTATTGCAGGTGAAGAAGATAAGAACGTAGAGATAAAGTTGAGAACATTTGGCACTGAGGAAGATAAGAAGGCTATTTTTTTAGTAGTCAATGCATGTTCTAGCAAGGACTATACAAATAATATTGTTGGTGTCTGCTTTGTTGGTCAAGATGTTACAGGACAGAAAATTGTAATGGACAAATTTATTCACATACAAGGTGATTACAAGGCTATTGTACACAGTCCCAATCCTCTGATCCCTCCCATATTTGCTTCAGATGAGAACACATCCTGCTCTGAGTGGAATACTGCCATGGAAAAGCTTACTGGGTGGAGCAGGGGTGAAACGATTGGCAAGTTGCTAGTTGGTGAGGTTTTTGGAAGCTGCTGCAGGCTCAGGGGTCCAGATGCCATGACTAAATTTATGATCATATTGCATAATGCAATTGGAGGCCAAGACACAGACAGGTTTCCATTTTCATTTTTCGACCGAAATGGAAAGTACGTGCAAGCTCTCTTGACGGCAAATAAGAGAGCAAATATGGATGGACAGATTATTGGAGCCTTCTGCTTCTTGCAGATTGCCAGTCCTGAATTGCAGCAAGCTCTCAAAATCCAGAGGCAGCAGGAAAATAAATGCTTTTCGAGAATGAAAGAGTTGGCTTACATCTGTCAAGAAATTAAAAATCCACTGAATGGCATACGCTTTACAAATTCATTATTGGAGGCAACAGATTTGACAGAAGACCAGAAGCAGTTCCTGGAGACTAGTGCTGCTTGCGAGAAACAAATGTCAAAGATTATAATGGATGTTGATCTGGAAAACATTGAAGATGGGTAAGCTTCTGTTATTGATTATGCTTGCTAGTCTGTTAGTTGAAGAAAAAATCGTTTATAACTTCTAACTCATTTTGGAGTATATGCCTTGCTTTTATTCATCTTTTTGTTGGCTCTTCTAAAAAATTACACATTATAATGATTGCATCACTTTTGGTGCATCTCAATAAAATATTTGGTGATCCTTTAAAAAAAATAAAGAAGAGAAAGTCATTTGTTCTTATGTGTACAAGATGACTAAGATTTTGTGTTCTGACAGTTCACTTGAGCTGGAGAAAGAAGATTTTTTTCTTGGGAGGATAATAGATGCTATTGTTAGCCAAGTAATGTCGTTGCTGAGAGAAAGAGGTCTCCAACTTATCCGGGATATTCCAGAAGAAATTAAGACACTGGCTGTGAATGGCGATCAAGTGAGAATTCAGCAGGTGCTGGCAGATTTTTTGCTAAACATGGCACGCCATGCACCAGTTCCAGGAGGATGGGTAGAAATCCAAGTTCGCCCTAGTTTGAAGCAAGTTTCTGATGGCACAAATGTTGTGCATACTGAATTCAGGTATTTGATACACATTACTTTACCTTTGATAACAAATTTTGTTGTTATATTGGTGACTTGAACTCAACTGTTGTCTTTGAAGTTCCTTCAGCATATGGTAGATTTAGTAAGTTAGTGAAGAAAAATGCATGAATGAATTAATTTTAAAAGGTAAAGAATTTACAAAGGTTGTATGTGAATGTGATTGATTGGTCATATTTTCAGTTGCTGTTGGTTAGGACTCCATAGTTACAGTGTGTTTTTTGTTTTACAACCTCCTAAACTTGCTGTTTTACACAATCATGGCACTTTTGTAATGAAGTTGAGTGATGATGTTGTTTGAATTCCCTTGGAAAAGGATAATGTGCCCGGGCGAAGGTCTTCCTCCTGAATTGGTGCAAGACATGTTCCACAGCAGTCGATGGGTGAGTCAAGAAGGACTAGGGCTGAGCATGTGCAGGAAAGTAGTAAAGCTTATGAACGGGGAAGTCCAATATATCAGAGAATCAGAAAGATGTTACTTCCTGATCATCCTTGAGCTACCAATCCCCCGAAGAGGTTCAAAGAGTATTATTATTGGCTAG

>IbHKL10

ATGGCGGGCTCAGGGACAGGGAGTAGTAGTAAGAGGTTTATTGAGCACCAGAGTTCTTCAGCTCAAATTGCTCAATCTTCAGGTACGAGTAACTCTAATAATCGCTATCCTGTAAGCAAGGCGGTAGCGCAGTACACTGAGGATGCTAGGCTCCACGCGGTGTTTGAGCGGTCCGGCGGGTCGGGGAAGTCGTTTGATTACTCTGAATCAGTGAAGGTCGCGACTCATTTTGTAGCGGAGCAGCAAATCGCCGCGTACCTGTCCAACATTCAGAGGGGAGGTCACATACAGCCTTTTGGGTGTATGATTGGCGTGGAGGAGGGGAGTTTTCGCGTGATTGCGTATAGCGAGAATGCACGCGAGGTGCTTGGTTTAATGCCTCAGTCAGTTCCGAGTCTGGATCGGCCGGATATCCTGGGGATTGGGGTGGATGTGAGGACGCTTTTCAGGCCTTCGAGCTCGGTGTTGCTCCAACGGGCGTTTGGAGCGCAGGAGATCACGTTGCTGAACCCTATTTGGGTTCACTCCAAGAATTCCGGTAAGCCTTTTTACGCGATTTTACATAAGATTGATGTTGGCATTGTGATTGACTTGGAGCCTGCTAGGAGTGAGGACCCTGCCCTGTCCATAGCCGGGGCTGTGCAGTCACAGAAGCTCGCCGTGAGGGGCATTTCGCGCTTGCAGTCGCTTCCCGGTGGGAATATTAAGCATCTTTGTGATGTTGTGGTTGAGTGTGTGAGGGAGTTAACCGGGTATGATCGAGTTATGGTGTATAAGTTTCATGAGGATGAGCATGGGGAGGTTTTGGCTGAGAGCAAAAGACCAGATTTAGAGCCTTATATTGGGCTGCACTATCCAGCTACTGATATTCCTCAAGCCTCCAGGTTTTTGTTTAAACAAAACAGGGTTAGGATGATTGTTGATTGCAATGCCACCCCGGTACGCGTTATTCAGGATGAATCACTAAAGCAGCCGTTGTGTTTAGTTGGTTCGACTCTACGGGCTCCTCATGGTTGCCATGCCCAGTACATGGCCAATATGGGCTCCATTGCCTCGTTAACTCTCGCAGTTATTGTAAATGGGAACGAAGATGAAGGTGTTGGAGGGAGGAATTCGATGAGGCTATGGGGGTTGGTTGTAGGCCATCACACTTCGGCTAGGAGTATTGCGTTCCCCCTTCGTTCTGCCTGTGAGTTTCTTATGCAGGCCTTTGGGCTCCAGTTGAATATGGAATTGCAATTGGCATCACAATTGGCAGAAAAACATGTGTTAAGGACACAAACACTGTTGTGTGACATGCTTCTAAGGGACTCCGCCACTGGGATTGTTACCCAGAGCCCTAGTATAAGGGATCTTGTGAAATGTGATGGGGCTGCATTGTACTATAAGGGTAAATACTATCCTTTAGGCGTGACACCTACTGAAGACCAGATAAAGGATATAGCTGTGTGGTTATTGACTTACCATGGAGACTCGACAGGTTTGAGCACTGATAATTTGGCTGATGCAGGGTACTCTGGTGCAGCTTCACTTGGTGATGCAGTTCGTGGGATGGCTGTTGCCTATATAACACCAAAAGATTTCTTGTTCTGGTTTCGGTCCCACACTGCAAAAGAGATTAAGTGGGGTGGTGCTAAGCATCATCCACAGGATAAAGATGATGGACAAAGGATGCACCCTCGATCTTCATTCAAGGCATTTTTAGAAGTAGTTAAGAGACGTAGTTTGCCATGGGAGAACGCAGAAATGGATGCAATTCACTCTTTGCAGCTTATTCTACGCGATTCTTTTAAGGATGCTGAGGTAAGCAATTCTAAGGCTGTTGTGCATGCTCCGCCAGGAGAGTTGGAATTGCAAGGAATGGATGAGCTGAGCTCTGTTGCCAGAGAAATGGTTAGATTGATAGAAACTGCAACCACTCCGATATTTGCTGTAGATGCTGAAGGACACATAAATGGATGGAATGCGAAAGTTGCTGAGTTGGTGGGGTTGCCAGTTGAAGAAGCAATGGGAAAGTCATTAGTTCATGATCTTGTTCATATGGAATCACAAGAAACCACTGAGAAGCTTTTGTTTAATGCTTTAAGAGGTTATCTCTTTCTGTCTATGTTTAATTTATTTTGCTATATCGTACCATATAATCCTTATGCAGTTTCCATCTGAAATGCAAAACCACATTTCAGCCTTTGGTTCTTTAATTTGAGTTTTTGCTGAACTTTAAAGTGATGTTGAAGCATGTTTGTCTTATCATTGTGGCTAAATTTACGGAGTGTTCATAGAATTGGCTAATCGTATGGCTAAATATTGCAGGTTCTGAAGACAGGAATGTAGAGATCAAGTTGAAAACATTTGGCACTAAGCAACATACAAAAGCCGTTTTTGTGGTGGTCAATGCTTGCTCTAGCAAAGACTGTACAAATAAAATTGTTGGTGTATGTTTTGTTGGTCAAGATGTGACGGAACAGAAAGTTGTAATGGACAAATTTATTCACATACAAAGTGATTACAAGGCAATTGTACATAGCCCCAATCCTCTGATCCCTCCCATATTTGCTTCAGATGAGAACGCTTGTTGCTCTGAGTGGAACATCGCCATGGAAAAGCTTACTGGGTGGAGCAAAGGGGAAATGATGGGGAAGATGTTAATTGGCGAGCTTTTTGGAGGAGTCTGTCGACTCAAGGGTCCAGATGCTATGATGAAATTCATGATCACATTGCATCATGCGATTGGAGGCAAAGATACAGACAAGTTTCCCTTCTACTTTTTTGACCGAAATGGAAAATATGTGCAAACCCTCTTGACCGCAAATAAGAGAGTGAATATGGATGGTCGGGTTATTGGAGCCTTCTGTTTCTTGCAGATAGCAAGTCCTGAATTGCTGCAAGCCATCAAAATCCAGAGGCAACAAGAAAACAAGTGGCTCACTAAGTCAAAAGTGATGGCATATATCTGCCAGGAAATTAAGAATCCACTGAATGGTATACGCTTTACAAGTTCTTTATTAGAGGCAACAAATTTGACAGAACATCAAAAGCAGTTTCTGGAGACTAGCGCCGCTTGTCAGAAGCAGATGTCGAAGATTTTAAGGGATGCTGGTCTGGAAAACATTGAAGATGGGTAAATTTCTCTTTATTGATTATGCTTGCTAGTAAGTGCGTTTAAATAAAAATGCTTTTCATTTATGACTTAACTTCTAATATATCGAGAACTTGTGGGTACATCTTTTTTTTTTTTTGGGTGACGAGGGAAACCCGCAGCCACTACCCGAGTGTGCAGGTAAACCCCACCTTGTGACCCTAGTGAAAGGATCATAAAAAAGCCATACCGACCTCAAACCAAGGAATTTGAACTCGTGACCTTGTGGAAACAAGTTTCTATCTTTAGCCAGCTTGACTAGGGTTACCCCCACTTGTGGGTACATCTTAGATGGTGGAGATGGTGTGTTTGGTGTGGGGGCTACTGTATCTGTAAAATATCTGGAGTAAAGAGGCACTTTATTTCTACAAGATTGACTAATGTTTAGTGTTTTGACAGTTCACTGGAGCTAGAGAAAGAAGAATTTCATTTTGGGAGTGTTATAGATGCTATTGTCAGCCAAGTAATGCTATTGCTGAGAGAAAGAGGTCTGCAATTTATGCTGGACATTCCAGACGAAATGAAGACGCTGAAAGTGTATGGTGATCAAGCAAGAATTCAACAGGTGCTGGCAGATTTTTTGCTGAACGTGGTACATCATGCGCCAACTCCAAAAGGATGGGTAAAAATCCATGTTCGGCCTAGTTTGAGGCAAAGTTCTGATGGAATAACCATAGCGCACGTTGAATTCAGGTGAGGTTGACTGTAACATTGTCTCCTTCCTTTTCGTTGAGTTTAACTGTCATATTGTTGGAGTGAACTACATCTGTCATATAGTTGGAGTGAACTACATCTACCGCTATCACTTGTCTCCTTCCTTTAGAGAATAGATACTTAAGTCATGACAATAGGAGAGCAAAAAAATGATACAAGTGTGGTCGTGGAATGTAATTCTTCCAACAAATCTTGATATATCTATGTATCTAGGAAACTTTGATAAGGAATCTAGATTCCTTGGATGGCTAGACATAGAACTTATCGAGTATAGCTTACATGTGTACACCAATTAGTTAGATTAAGAAGGCAAAAAGAAGTAGAAGTCTAGTGGATGAGAATGTTACTAGACTCACTTCGAGCGGCAGGTGAGGGATATTGGGGGATTTATTTCCTCGTGGGCTAGAGGGGGTTTATGCTCATATACTCTAGTATTTGGTAAAGCCGTAAAGTAACTAGTTAACAAAGTTATGGATCACATTTCTTGTTTTCCGCTTGTATTGTAAGTTACAATTACGTTTCTTTTACGAGCTATGAAGTCGGTTTCAGTCAAAAAATTGTCACCTGTTTTAATTTTTCTTGAAAAAGGTTTATCTGCCCCGGTGAAGGTCTTCCTTCAGCGCTGGTCCAGGACGTGTTCAACAACAGTGAATGGGAAACTCGGGAAGGATTAGGGCTGAGTATGTGCAGGAAAATAGTGACACTCATGAATGGAGAAGTGCGATATGTAAGAGAAGCAGAAAGGTGTTATTTCCTAGTTATCCTCAAGCTGCCCGTGCCCACAAGAGGCTCAAAAAGCGGTTGA

>IbHKL8

ATGGATTTGCAAAGCCAGGAAAACAAACCACCCACAAGCAAGAAAATGGAGAATCATGCCAAAGCTGCGACCTTTTCTTCGTCTGCTACTAGCAACTTGAACACCGGCAAGGCCATAGCTCAGTACAATGCTGATGCGAAGCTAATGGCTGAGTTTGAGCAGTCTAGGGAGTCTGGTAAGTCCTTTGACTACTCCAGGTCTGTTATTGGTGCTCCACAGAATGTGACTGAAGAAGAAATGACTGCTTATTTATCAAGAATCCAGAGGGGTGGGCTTATCCAACCCTTTGGTTGTATGCTTGCAATTGAAGAACCCAGTTTCAAGATTGTAGGGTTTAGTGAGAATTGCTTTGATTTGTTGGGTTTGAAGAGTGGTGTTGAGCCTCCGGAGAGGATGAGTTTGATTGGGATTGATGCCAGGACTCTTTTCACCCTTTCTTCAAGGGCTTCTTTAGCCAAGGCTGTGGCATCTAGGGAAATTTCTCTTTTGAACCCAATTTGGGTGCATTCTAAGACTAATCAAAAGCCTTTTTATGCTGTACTCCATAGAATTGATGTAGGGATTGTGATTGATTTGGAGCCTGCTAACTCTGCTGATCCTGCACTGTTGCTTGCTGGGGCAGTGCAATCACAGAAACTCGCGGTCCGGGCTATTTCTAGGCTTCAGGCACTCCCTGGGGGAGATATAGGGACATTGTGTGACACAGTAGTGGAGGATGTACAGAAGCTGACCGGGTACGATAGGGTAATGGTTTATAAGTTCCATGATGACAGTCATGGCGAGGTTGTGTCCGAAATTAGGAGGTCAGACTTAGAGCCTTATTTAGGATTGCACTATCCTGCAACAGATATCCCACAAGCGGCGCGTTTCTTGTTTAAACAGAACAGGGTTAGGATGATCTGTGATTGCAATGCACAACCCGTCAAGGTTTTTCAAAGCGAAGAACTAAAACAGCCTCTTTGCTTGGTGAATTCGACTCTTAGATCACCTCATGGCTGCCATACCAAGTATATGGCTAACATGGGGTCTATAGCCTCGTTGGTGATGGCTGTTGTTATTAATAGTAGTGAGTCCATGAAGCTTTGGGGATTGGTAGTGTGCCACCATACTTCTGCTCGCTATGTTCCTTTCCCTCTTCGCTATGCATGTGAGTTCCTTATGCAGGCGTTTAGTCTTCAGCTTTATATGGAGCTTCAATTGGCCTCACAATTGGCTGAAAAGAAAATTCTCCGCACTCAGACCTTACTATGTGACATGCTTCTCCGAGATGCTCCGTTTGGAATTGTGACGCAAACTCCTAGTATAATGGATCTCGTGAGGTGTGATGGGGCTGCACTCTATTATGACGGGAAATGTTGGTTGCTTGGTGTAACACCAACTGAGACACAAGTTAAAGATATTGCAGAGTGGTTGCTACATAATCACGGGGATTCTACAGGTTTGAGTACGGATAGTCTTTCGGATGCTGGCTATCCCGGAGCACCCTTATTAGGTGATGCAGTTTCTGGCATGGCTACTGCAAGAATCACATCCAAGGATTTTCTATTCTGGTTTAGGTCTCACACTGCAAAGGAAGTTAAATGGGGAGGGGCTAAGCATCATCCCGAGGATAAAGATGATGGTGGAAGAATGCACCCCCGATCTTCATTCATTGCCTTTCTAGAAGTGGTGAAAAGCAAAAGTCTGCCTTGGGAGGATTCAGAAATTAATGCTATTCATTCTTTACAGCTCATAATGAGAGATTCTCTTCAAGGGATTGGAGAGAACTATATGAAAAGCGTGTCATCACCCCAACAGACTGATTCCGAAGGGACAAGGTTCTATGAACTTAGTTCAATGGCGTTGGAACTGGTCAGGTTGGTCGAGACAGCAACAGTTCCTATTTTTGGTGTTGATTCATCTGGCTTAATCAATGGATGGAATGCAAAGATTGCAGAACTAACAGGATTGCAAGCTAATGTTGCGATTGGAAAGTATCTCATTGATTACGTTACTCATGAAGATTCACATGAAACTTTCAAAGGTCTTATGTGTCGAGCTTTACAAGGTAAATCTTTTCTATAACTCTTGGTTCAATAAAAATTGAAAATTTGGGAAATATAGCACATATTCATTTCATGCCTTCTAAACTTGAGGAGTTGCCTTGGATTTGATATTCATTACTACTGGTGGCAAACACCGGGTTAACACAAGTGACAAAGTGGACCGGACAGGCTTTGATACTATGTTCTAATAGTCTATTAGATTATTGGGCCAAGATCGGTCTTTCAACTGGGTTATAATGCAATTGGGCAAACTACACTTAAAGACTGAATTCAATCAATTAGTTAGTCTAACCCATAACCTTATAAACCCATCTGTTTCTCTCCTATTTTTCAATGTGGAACTCTTAATTGGTTCAAACTTCCAAAGTTTATGCTGCATACATATCATTGCCTTACATATTGACTCGGTTAACGTAATCATTACTTGTTAGTTGTTACTCTGTCCCTAATGTGATGTGCTGTGCTAAGCTCTGTATTGCTTTGTTTACTTCAGCAAAACTGAACCCATTATCTTTTTATTTTGCGATTATGATCCCTATTATCAATTTCTTTACAAGATTCAATTTTTGGATACTTGCTTATCAGGCTCTTCTTATTGTGACCATATTATGCACACAATAATTCTTGATTCACGCTGGTTCAGGTGAGGAGGACAGAAATGTAGAAGTAAAACTGCTAAAGTTTGGGGAGCATCCAACAAAGGAAGTTGTATACCTTGTCGTTAATGCCTGCACAAGTAGGGACTACAAAAACGATATTATTGGGGTGTGCTTTGTGGGTCAAGACATCACTCCTCAGAAAGCTGTGATGGATAAATTTGTTCGGTTGCAGGGAGATTATGAGGCTATTATACAAAGCCTTAATCCACTAATCCCGCCAATATTTGCTTCTGATGAGAATGCCTGCTGCTCTGAATGGAACGCGGCTATGGAGCGGTTAACTGGTTTGGTGAAATGTGAGGTTATAGGGAAGAGACTTCCGGGTGAAATTTTCGGTGGCTTGTGTCGGCTTAAGGGTCAAGACGCTCTTACCAAATTTATGATTCTCTTGTACCAAGGGATAAGTGGTCATGATACCGAGAAGCTCTCATTTGGATTTTTTGATAGGAAGGGGAACTTTATAGACGTGTTTATAACCGCAAATAAGAGAACTGATGAGCGTGGGAATATAATTGGATGTTTCTGCTTCTTGCAAACGATGGCTGTTGACCCGCAGACATCCGCAAGAGATATAGAAGATGATAGAGAATGTCTCTCGACTCTTAAAGAGTTTGCTTACATCCAACAGCAGATGAAAAATCCCTTGAATGGAATTCGTTTCACCCACAAGCTCCTTGAAGGTACCGTTACTTCAGATCATCAGAAACAGTTTCTCGAGACAAGTGAAGCCTGTGAGAAACAGATACTCTCTATAATCGAGAATATGGATAGTGGAGGCATCGTGGATGGGTAACTCCTTTTCTTTGAATCTCCAGTTATTACTAGCTATTTGCTTGCATATTTTGTTAAAGGAATAATGTTCAAATAGGCTACTGAACTACACACAAAAATACAATTGAGCATCCAACTCAAAAAGACTGCTATTGGGTTCCTGAACTGTCCAAAATTAATCAATTAAACCAAAATTGACTTGTTCTTCCGGTTACTGTTGACGTGGTCCTTTTATTTATTTATTTATTTAATTTATTTTAATAATTTTAAATAATTAAAAAAAAAAATCCTGCCCCCTTCCCCCGGTCTTCGTCTCCGTCTCTGCCGGAGACCAAGACCTTTTCTTCGTTTCTTGCCAAAGACGTAGAGTTTTCTTCATCTCTAGCCGGAGACGAAGACGTTTTCTTCGTCTCCGGCCTTGAGATGAAGACGTTTCTCGGAGACAATGACAGGGAAGGGGGGGGGGATGCCAATATGCCATAACATTTGAATGTTTCTAATATAAAAAATGTCACAATTTTAGATGGTGCAAAATCTCTTGTTATCTATAGGAAAGTAATTGATTAAACATCCACCAAGCTCGAGTTATTAAGAATAGATCATTCAATATACCTTACATAATTTTGCCTCTTGGCTGAATTTATTTATTTATTTATTTTTTCATATTGGTGATTAGTCTATCTAGAATCTCGATTGCTTTCCAAATGTATAAAGTTAATATTTACTTCTAAAAGCAACTAAAAATCTGCAATGAGCCATTTTAAGTAAATGCGGGGAAGACTTTTGAACAATATAGTAAGTGCGATGAAAATTAGCTTAGTAAATGGCTTTCTGATTATGTTACTTTCTCGTGGTTACAGCAACAAAGTGGAGCTAAAGACGGAAGAGTTTGTTATAGGAAATGTCATAGATGCAGTGGTTAGTCAAGTTATGATCCCGCTGAAGGAGAAGAATTTACAGCTACTTCATGATATTCCGGACCAGATCAAATCTCTCCCTATATACGGGGATCAAATTAAGCTTCAGCTTGTCCTATCGGATTTCTTGCTCAGCATAGTGCGCCACGCACCTTCTCCAGATGGTTGGGTGGAAATCAGGGTATCCCCGGGGTTGAAGCTGATTCAAGACGGGAATGAGTTTATCCATATCCAGTTCAGGTATCATGGTCCTATACATTCATTTTTACCTCTAAAATGATTCATATTTCTTTTGTTGAGCATATATATATATATATATATATATATAAACATAAATGTGCAACCCAACTATCAGTTTAGGCTTTTAGTTGAAATGGAACACATACTTCAATTTGGTATCAGAGCCAATCCTATGCCCTCATCAAACAGAGTGTATGGTTCACAGCTCGCACACGTGAGGGGGCGTGTTGAGCATATAATATATATGAGATGGAGCACATGCTTCAGTTAGTTTGAAGTTGCTTACATTATGATGATCCTTAATGATTCCATGTTCTCAGTACTTCTCCATCTGATTATCTCATTAATTGTCTCCCGTAGAATGACTCATCCCGGGCAAGGCCTCCCTTTTGCTCTTATCGAAGACATGGTAAGAGGAGGAACTCGATGGACAACACAAGAAGGAATTGTTTTACATTTGTCACAGAAACTCGTTAGAATGATGAATGGTCATGTCCATTACGTCAGAGAACAACAAAAGTGCTACTTCCTAATCGACCTTGATTTCAAAACACAAAAACCAAGGTCGCGGGAGTCAAGTATGGATACGAGCAGAATAACCTGA

>IbHKL7

ATGTCGTCTAGATCGGGTACAATCAGGACAAATTGCTCCATGAGCAGCTCTGCTCGGTCGAGGCATGATGCTCGCGTTGTTGCTCAGACATCTATTGATGCTAAGCTCCATGTGGAGTTTGAGGAGTCTGAGGAACAGTTTGATTATTCTACCTCTGTTAATTTGTCCAATTCAACTAGCAATATCCCCCTCTTCAACTGTGTCTGCTTATCTCCAGAAGATGCAAAGAGGAAGTCTTATACAACCATTTGGCTGCTTGATAGCCATTGATGAGCATAACTTTTCTGTCCTTGGATTTAGTGAAAATGCACCAGAAATGCTGGATTTGGCACCACATGCAGTTCCAAGCATTGAACAGCAGGAAGCTCTGACTTTTGGCACCAATGTTAGGACACTGTTTCGGTCAACTGGTGCTGCTGCACTTGAAAAAGCAGCAAGTTTTGAGGAAGTTAGTTTGATTAATCCTATTCTGGTTCACTGCAAAAATTCGGGTAAGCCTTTTTATGCAATTTTACACCGAATTGATGTTGGATTAGTTATAGATTTGGAGCCTGTCAATCCAGCAGATGTTCCAGTGACAGCTGCTGGAGCATTGAAATCATATAAACTAGCAGCTAAAGCCATTTCGAAGTTGCAATCACTGCCAAGTGGAGATATATCATTGTTGTGTGATGTGTTAGTTAGAGAAGTAAGGGATTTGACAGGTTACGACCGAGTTATGGTTTATAAATTCCATGAGGATGAGCATGGGGAAGTTGTTGCAGAATGCCGCAAGCCTGATCTTGAACCTTATCTTGGCTTGCATTACCCTGCTACTGATATACCACAAGCTTCAAGATTTCTTTTCATGAAAAACAAGGTCAGAATGATATGTGATTGCTTAGCTCCATCGGTAAAAGTAATCCAAGACAAGACATTGGCTCAACCATTAAGCCTTTGTGGATCCACATTAAGAGCTCCCCATGGATGTCATGCACAATACATGGCCAATATGGGGTCCATTGCATCTTTGGCAATGTCTGTGACAATCAATGAGGATGATGATGAGATGGATAGTGATCAACAAAAAGGAAGAAAACTCTGGGGATTGGTGGTTTGCCATCATTCAAGCCCAAGGTTTGTTCCATTCCCCCTTGAGGTATGCATGTGAATTCTTGGTCCAAGTTTTCAGCGTTCAGATCAATAAGGAAGTGGAGTTGGCAGCTCAACGTCTAGAAAAGCATATATTGCGAACCCAGACTGTACTCTGTGACATGCTTCTCAGAGAATCTCCTGTGGGTATTGTCACCAAGTCTCCTAATATTATGGACCTTGTCAGATGTGATGGAGCTGCACTTTACTATAGGAACAAATTTTGGTTGCTTGGTGCAACGCCAACAGAGCCCCAAATTAGAGATATAGCACAATGGCTTCTTGATTCTCATAGTAGTAGTACAGGGTTAAGCACTGATAGCCTCATGGAAGCTGGCTACCCAAATGCTTCCGTTCTTGGTGATTCAGTCTGTGGAATGGCTGCTGTCAAAATAACTGCAAAAGATTTTCTCTTCTGGTTTCGATCTCACACAGCAAAAGCGATTAAGTGGGGTGGTGCAAAACATGATCCTGGAGACAAGGATGATGGGAGAAAGATGCATCCAAGATCATCTTTCAAGGCTTTCCTGGAGGTGGTTAAGAGGAGTCTGCCTTGGGAAGATGTGGAGATGGATGCAATTCATTCCTTGCAGCTGATACTGAGAGGCTCTTTGCAAGACGAAGTTGTTGATAATTCTAAAATGATTGTGAATGTACCTGCTGTGGACACCAGTATACAGAGGGTTGATGAACTTCGAATTGTGACAACTGAAATGGTTCGCCTTATTGAGACAGCATCTATACCCATTTTGGCTGTTGACACTTCTGGCTGTATCAATGGGTGGAACATTAAAGTGGCTGAGCTAACCGGATTGGTTGTACAAGAAGCTATAGGTGCGCCCTTAGTTGATTTGGTTGTCAGTGAAGCTGTCAGCACCATTAAAAATGTGCTCTCCCTCGCTTTGCAAGGTTATATTCTATCTTCTTGCTCCTTAATTGCATGTTTAAAAATTCTGTACAGAAGATATGTAAGCTGCAGCTGTAAACCTTTGTACCTTTGTCAGGATAAAATATTAGTTACACATACTCCAAATTAAAATAAGGGAGAGAGAAACTAAATTAAAAGGCCAGTTAGAGGCATTACGGTCACTTTTAGGCTTTCCATTTTTCTTAACCAAACCCATGTTATTTTAACATTGTAAATTTGTAACTTTCAGTTTCTTTGTCAATTTTGATGGACACATCACACGTCGCAAGGGATCTTGGGATTCTTGGGGGTGGGACATGATAGGAAATACTTTTTTTCTTTCAAACATCATGCTTTTACTGTCTCAGATATCTCAAGTTCTTGAATTTTGAAATAATCTGTTTATATGCTTCACAGTACTGACATTATCACTCTCATCAGCAGCTTGATATATAGTTTCTTTGAAAAAGTTGTATGGGTTATATTTTACATGAAATGTATCTATACTACAAACTGATCTACGATGTTGAATGTCAGGCAAAGAGGAGAAAAATGTTGAAATCAAACTTAAAAAGTTTGGTTCTCCAGAAAACAATGATCCTGTTATTCTGGTAGCTAATGCCTGTTCAAGTCGGGGACGTAAAAGGAAATATTATTGGAGTTTGCTTTGTAGGACAAGATGTTACAGGACAAAAGTTGATTATGGACAAATATAACCGTATTCAAGGTGATTATGTTGGAATTTTGCGCAGCCCTTCCGCATTGATTCCTCCAATCTTTTTGATGGATGAGCATGGTAGATGCTTGGAATGGAATGATGCAATGCAAAAGTTGACTGGTCTGAAGAGGGCAGAGGCCATTGATCAAATGATTCTCGGTGAGGTCTTTACGGTTAGTAGCTTTGGTTGCAAGGTCAAAGATAGTGACACATTAACCAAACTCAGGATATTACTGAATGGAGTAATTGCAGGACAGGATGCAGAGGATTTGTTATTTGGTTTTTTTTGATAAGCAGAATAAATATGTTGAAGCATTAATATCAGCAAATAAAAGAACTGATGTAGTGGGGCGGATCACTGGGGTCCTCTGCTTTTTACACGTTCCTAGTCCAGAACTTCAGTACGCAATACATGTACAAAAACTATCTGAACAAGCAGCTGCAAATAGCCTTAAAAAGTTGGCTTATGTTCGTCGAGAAGTTAGAAACCCGTTAAATGGTATAAAGTGCATTCAGAATCTGATGAAATCTTCTGACCTAAGTAAGGATCAGATGCAGCTTCTGAAGACCAGTACAATGTGCCAAGAACAGCTGGCTAAGATTATTGATGATACCGATATTGAAAGTATTGAAGAAAGGTATGATAATCTAACTTGCACCATTTCATTTGTGGTTGGAAGATCTGTTAATATATGTGTTACAGGCTCATGACCATTGTTCTATTCATCTCTGTGATTTCCAGGGGCATTTACTTGATAACATACATGCAAATAAGATGATGTCTTGCACTGCTTTGGCTCTACTATGTGCCTCTCTTAAAAGAAACGCTATGCCAATAGGCCGCGGGTGTTTTTCTATGTGTTTATTTGCCTTTTCATTTCTTTATCTCTCTATTCTTCTTCTTCTCCTCCTTCTTTTGGGTGGGGTGGGGAGGGGGTGTATTTACTGGATTTGCCTTCAATATATGGCATTTAGGTTCTAATTCTTTGTGAAGCAACCTGTATATGCTTAACTCCTTGGTGTCTGAATAACTGGATGGCCGATATCTGTTTAGATGCTACATCTTTGGCAGTTTTGTATTGGTGCTTATCCTTGTGAAAACCTTTTCAGCTATATGGAAATGAACTGTTGCGAGTTCAGTCTTGGTGAGGCTATTAAAGCAGTCGTAAATCAAGCTATGATTCCAAGTCGGGAGCGCCAGGTGCAGATCATGTGCGATTTACCTGTTGAAGCATCATCCTTGTACTTGTTTGGAGACAATTTGAGGATTCAACAAGTGCTCTCAGACTTCTTGACAACTGCTGTACTCTTTACACCTCATTTTGAAGAATCATCCGTTCTATTCAGAATTATTCCTAGAAGGGAGCAGATTGGAGCCAAGATGCATGTAGTGCATCTTGAATTTCGGTAATACTATTTATATACTCAGCTTAGCATATGATCACCTCCATATAACTTGTTTAGTCATGAAAAGAGATTTCTAACTTAGTGTGATCTTTTGAGATCTTCGGTCATTATATCCAATCTGATCCCAGTTAAGTTGTTCAGGTTTAAGAATTGCTAACATTTAAGTAAAACAGGGAAGATTATAGGCAAGCATAATAATGATCTTTTGAGATCTTCGGTCATTATATCCAATCTGTTTTGAGTCAGCAGTGTATTAGATGCATGAATAATGCCCACCAACATTTTTCCCCTCCGAAATTTGATTAAATACTAAATATATCTCATTCTTATCTTTTTCATCAAGGAATTGGTAGCCGTGGCATTTTGTAAACATATTCATGCAGTTCTCCGCTATTTTACTTCTATTGAATGGTCTGTTTATCTTCCAGGATCACACATCCAGCCCCGGGGATTCCAGAAGAGCTAATCCAGGAAATGTTCAACTATAGCCAAAGCATGTCGAGGGAAGGTCTTGGGCTGTATATAAGCCAGAAACTTATTAAAATTATGAATGGCACTGTACAGTATCTTCGAGAGGCAGAGAGGTCATCATTCATAATCTTAGTAGAATTTCCAGCATCTCTACGAAGCGACCATCAATAACTTCAATCATCAGTGTGCTCACCGCCTGCTTGGGGAGAAACTGCACCTTAATTATGGCAGACGAGTACTCAAACATCATGGCTTGCCCGATCTGCCTCGTCCTCTTCAGCAACAACCATAATAAAGAATGA

>IbCKI1

ATGGTCAACTTTGTTCGCTCACTCAAGACTATGTGGCCTGTCTACTTGGCCCTCACTCTCGTAGGTTTCCCCACAAACTTTTTTTTTTTTTTTAATATAATTTTCGAATGTATTGAGTGAATAAATTATTGCAGTGTTTAGCCGGACTGGGGATTGCAGGTTGGCTGATTTCGGTGTTGGTTGCGATTCAACACCATACTACCCAGGTATATATATATATATTCTGTTCATAATTCTCAACCTATTGAAGCACAAACATCATTAAAAACATCTCTAATTAAGCATAAACATCTTTTTTTTTTTTTTACAATGTAGTATTGTTCATAACTACTGAAACATTCTTAGGAATGAATATAATGTTGTTTGGTTGGTTGCAGGGGGGAGCGAAAGTTGAGAAGAGGGATGTGAAACTGGGGATGATTCTCCTGGGCATAAACATTTGTATTACAGTGACGAGTGTGGTGGTGTTGACATGGTGGAGGAGTAGAGTGATGATGAGGGAGATGTGTGTAAAGGCGGCGCTGATAAAGCAGAAGGAAGCCACAGAAGAAGCGGAGAGGAAGAGCATGAGCAAGAGCGTGGCGGTGGCCAATGCGAGCCATGAGGTGCGGACTGCTCTTGCAGGGATTACTGGTTTGATTCAGATGTGCCGCGCTGATGCTGATGCTTCTGCTGCTCATTCTGAACTCAACGATAATCTGAGGCACATGGAATCATGCACCAATGATCTCTATAGTAAGTAATTAATTAATTAATTATAAGATCGAATAAATGTATGAATGAATATTTGTTTGTCTGAATTTTGATTCTGCTGGATTGCAGGTTTATTGAATTCGATTCTGGATGCTAGCAGAATAGAAGCGGGTAAAATGCAGGTTGAAGAAGATGAATTTGATTTGCAGGAACTGCTAGAAGATGTGGTGGATTTGTACTATCCTGTAGGTATGAAGAAGGGAGTGGATGTAATATTGGATCCGTGTGATGAGTCCGTGGAAAAGTTTAGGCGTGTGAGGGGGGATAGAGGCAAACTCAAACAAGTTTTGTCTAATTTGTTGTTCAACGCCATTAAGTTCACAGATGAAGGCTATGTTGCTCTTCGTGTTTGGGCACGAAAACCTTCTCCTTGTCCTCCTTCTCAATCTCCTCCTAAACCCAAACGACCTTCTTCTTCTTCTTCTCCTATCGCCATTTTAAAGGGCTGTGTAGCAACCTTTTGCAGTGTTCCTGCAAAAACAGGAGGTGGGGAAGAAGAAGTAAATGATTCGGTGTTGGAGAGAAAAGATGGTGGGATAGAGTACATATTTAAGGTGGTGGATACAGGGAAAGGGATTCCCAAGGAGAAAAGGAATAGTGTGTTTGAAAACTATTCTCAGGTGAAAGATATGGGTAGGGGAAAGAAACATCAATTAGGGCATGGATTAGGGCTTGGGATAGCTCAGTCTCTAGTGCGGTTAATGGGCGGGGAGATTGGGATTGAGGATAAGGAAACCGGGGAAAGGGGGACTTGTTTTAAGTTCAACATAGTCTTGGACAACATTGTTATTCTAGAATCATCATCATCGTCTCATAATAACAACATTAATACTTATTCATCGGGTCATCACGTTGTTGTGTTCATGCATTGCGAGGAGAGGGGTAAAATCATAGGGAGATTCTTGGAAAACCGTGGCATTAAGGTTAGTTTGGTTCAGAAAGGACACCAGCAACTGAGCAGGAAATTGAAGAAGATCAAACGTGGGGCATTAAATCTCCCCCGTTCCACAACCACACCCTTACCCTCTTACTATTCTTCTTCTTCGTCCTCAAAGGAGGAGTTGGAAGACGAAACCATGCCTCTCCATACCAACACTTGTACGGTGTTGATAATAATAGACACAAGTGCAGCAGGAGAAGCATTATTCCCAGAAGTGATTAAAGCAGTTAGTGAATTCCATAGGGACCTTCAACCTGGTTGTGTTAGGGTTCTTTGGATAGACACTACTGCTTTAGGTAGAGGTGTGGATAACAACTTTCAGCTCCCTTCAACTGATCTCATCGTGTCCAAGCCCTTGCAAGGCTCCCGTCTGCATAGCGTGTTAGGGCTTCTACCCGACTTTGCATCAAGTAATTATGCGTCTTTAGCTAGCTTGCTATATTATTTCATTTCAATCAAACATAAATAATACTTTTGCATGCATGCAGGTTCCCAACTGGGAGAAATACAGGTGGTGATTGAGAAAGATAAGGAAGAAGAAGACGAGGATAATGGTGGTGGGAGCAGCAGTAGTGAAAAGAAGGCATTGACGGGGAAAAGAATCTTGGTGGTTGAAGACAATCCAACGCTGCGCAAAATATGCACCACAGTGGTTTCAAGTCTGGGTGCTCTAACTTACGCCTGTACTAATGGTGAAGAAGCTTTACAGCTCGTGTCTTCGGGTCTTCAGGACCACCATCATCAACCTCCCTTTGATTACATTCTAATGGACTGTGAGGTAAACAACATATATATATATATATTTTCACTCAAAATATAACAAACAATGATATGTAATTATATATATATGTATGTTTTGAATGACATGAATGCAGATGCCGATAATGGATGGGTTTGAAGCCACAAAGCGCATAAAAGAAGAAGGGAAAGCTATGGGGATATGGATTCCCATTATTGCTCTAACAGCACATACAGGAAAGGAGGATATGGACAAGGTGACTGAAGCTGGAATGGATTACTACTTGTCCAAGCCCATCAATGCTGCTACTCTTCTAACAGCTATTCACTTCTTGGACAAATCCACCACCCATCTCTAA

>IbHP2

ATGGACGTTGTGCCTCAGCTGCAGAAACAATTTGTGGACCTCATAGCTTCTCTATATCGAGAGGTCTTTGTAACAGTCCAAAAGATTTGGACTTTATTTGGCTTTTATGATTCATGTGTCTTGTGTTTTCCATTTCTTGGAGGATTCTGAGACTGGGTTTTTGTTTTGCTTGTGAAAAAAAATAGGGCTTTTTGGATGATCAGTTTCTGCAGCTTCAGAAACTGCAAGATGACAGCAACCCAGACTTTGTGTTTGAGGTTGTTTCACTTTTCTTTGAGGATTCTGAGAAGCTTATTAACAATCTGGCCACAGCTCTGTAAGATTTTCATTCATTTCTTTATTTTTATGGTTTGCTAGTCAAAAATGAAATCTTTTTAATGTTTGGGTATTTCATTTGTTTCCTTTTTCTCTATTGTATAGTCAGCAGCCAGTTGTAGATTTTAACCAGGTTGATGCCCATGTTCACCAGTTCAAGGGAAGCAGTTCCAGGTATCCTGATTGTTTTTGGCTTTTCATTATTGATTCATAGGCTATTTGTACCATGTAGCTGATGATTAGTTAGTTTTGTTTTATGGGATTTATGGATGCTAATAATGATTATATAATAGTGTAGGAAAGGATTATGAATCAACTACTTGTTTTGCTTTGTGTGAAATGGATTTCCATCTGATTGACTCTTAATGGATGCTGTGTTTAGTAATAAAGTCAAGAGTTTTCTTTTGTGTGGCTGTTCCTGAATGGTTTTGTTGTTCTGATAGGTTTTTGTGGCTTGAATTTTAAAGAATTAACCTAGTAAAGTGCTAATTCACTTTATGTTAGTGGTTTGTCTTAATCATTAGCTCATGCTCTCTGCTTAAACCACTTTAATGTTAGTGCTTTGAACTGTACTCAATAAATGGCTGGATTTGAATTTCTTCAAAGGGAGTGAGATTGTGAAAAGCTTTGTAAATTGGTTTATTTGGATTGAAGTGACTGTTTTTTTGGGTCTATTTGTACAAAATCGTCAACTGTCCTGGGCATTTTTTACCAAACAAATTTGTTTCAAAGCTTGTTGTATACAACTATACACCCAATTGTGTAATGCCAACAACTTTGAAATGGATAAGTGTAATAGAGGCTTCTGCTTCAATTTATGACAAGTCCAAATCATGACCATTCTTACTTAGAGTGTCGTGTTGAATGCTCTCTTTGAGAAGTCCTAAAAGCCAGCTTGATGTTGTATGAACAATGGGATGATCTCAATGAGGTGGCCATATCAACCTTTCAAATGTGTTTAGAAAAATTTAGTTCCATTTAACCTATAAAGCTCCAATTAACTTTAAGTTAGTGGTTTATTTAACAATTAATTCATGTTCTCCACTTAAACCACGGACTTTGGTGTTTAGTTTTATCCCTTTGAATTTTACCCATAAAATTTTCAGTTTGAGACTTTGCAAAGGGAGTGAGACTTAGAAACATTGGAAATTGGTGTATTTCGGTTTGGGGATTGAATGACTTTGTTGCCTATTTCGTTGTCCTCCGTATTTGTTTACAGATATAGCTTTGCCATCTTTGTTGCCTAACATGTTAACTCAAAATTCTATTTTTTGGTCTGCAGCATAGGTGCACAAAGAGTAAAGAATGCATGTGTTTCTTTCAGAAATTTTTGTGAGGAGAAGAACCTCGATGGGTAAGTTTTACTTAAGCTCTTATTGCAACTAATCCTTCATTTATACATGTAATCTTTAGGGATATTAAAAAAAACACAATGAACTGATGCTGAGATTTGGTTGCTTTGTGAATCTTATTATTGCATTTCATTCTCTATTTGCACACTTCAAAAGGTGGAAAAGGGTATGGCTAAAATGGCCTATCTGAAGAAAGAATATGCTAAAAAGAGATGAGGGAAGAGAAGGAACACAAACAATTGATTGGTCAGAAAGGATTTCATTGATTGCTTAGTCTATGTCTAGTAAAAGGAGATCTTGCACGTTGTTCTTGTTAACTAAACATGCTTAATGGCAGCATTCATACCTTTCGATAGTTCTGTATGTGTAAATTATTTGCGATAAAGATGTTGGTATTCAGAAGCTTTGATACTGAAATTACTAATATTTTTGGTGTAGGTGTGTGCAATGCCTGCAACTCGTGAAAAATGAATACTTTGTTGTGAAGAACAAACTCGAAACTTTACTCAGAGTAAGTACTATATTCCATATATCAAAACATTATTTTTCGATATTGTACGACTTCTGCATGAATGAACATGGTGCTGTCTAGTACTCGGGTTTATGAACCCCTGAATATAATCTCAGGCGGGTCTAGTACTCGCCTTTTCAACTGTTACATACTTACATTTGTTATTTTATGGACAAGAACACAACAAAACCTAAAACTTTTGACAGTTGATTCACTACTTACCTTGATTTCTGGGTGTAAACCAGTGTAAAATATCGAAATTCTGTTATATTAACTGTAATTTTCTCTGTTTAAATGCAGCTCGAGCAGCAAATCTTGGCCGCTGGTGGGAAAATTCCTGTTCTGCCATAA

>IbHP1

ATGGAGGTGAGCCAATTGCAGAACAGTTTTCTTGGGTACATGGCAGAATTATCTCGTGAGGTTAGTAAAATTATATATTAAATAAGAAAAAAAAAACCCACTTGACATCAGATTGTGTAGAAGACATGTGGGGTTTGGGTAATGTTTGTTGGTTTGTTTGTATTTGGTTGATTGTTGTTTCTGTGATGGTGTTTTTGATGTATGGTTGGGAATGTTATGGTTGAAGGGATTCTTGGATGCTCAGTTTAGTCAGCTCCAGCAACTTCAGGATGAAAGCAACCCTACTTTTGTAGCTGAAGTTGTGACTCTCTTCTTTGAAGATTCTGAAAGACTACTCAATGATCTCAACACAACTCTGTAAGTCCCCTTCCCCACCCCACCCCACCCACACATCATATATAGATACATATATACATGTAAGTTCTATGAGGCTTGGGATATATAATATAACATGCTCTTTTTTTTGTATGTTTATAGTAATCAGCCAGATGTGGACTTTAAGAAGGTTGATGCTCATGTTCACCAGCTCAAAGGTAGCAGCTCAAGGTAAAGTTCAGCTGGAACTCTTTTTTTTTTTGCTGTTTTATGTAAATTATGAAGAAAAAATACTGGGGAAAAGTTAAATCAGCTCAAATAATACCCAAGGGACTTGAAGACAAAACCAAGTTAAAGCAGCACAGTAAACACTTTTTAAGATAAATCACCTTTCAATAAGCTCAAAACAAGTTATTATTACGCAAGAGAGGGATTTACCCGTGCATACCTTTGAGTAGTGACTGTTGGTTTTCTTCGTCACTAGAAACAAAAAAGAAGTTATTATTGCCAAAGAACTATAGTCCATCGATATCAATCTATAATTCTAAATAAGATATCACACTTTTGATCTCTATTCCCTTTAAATGTACTCCAGAAAAGTTGGGATTAAGGAAAGAGTGGTTTCTTGAATGTGATTGAACAGCATAGGTGCTCAGAGAGTGAAGAATGTCTGCGTTGCTTTTCGCAACTTCTGTGAAGAACACAACATTGAAGGGTTAGTGCTTTGCTGATCATATGTTTACTTCATTTTTTTTTCTTGGATTTTCTTGATAATATTTGGGCTTTATGAGTTTCTTATGCCCAGAGAAGAATTGCAGCAGTACTGAGAACTCTTTTCTTTCAAATATAGGAGCTTGAGATGCTTGCAACAAGTAAAACAAGAGTACTTGCTAGTCAAGAACAAGCTTGAAACTTTATTCAGGGTGAGCCAAACTCAAATTCCTCATTTTTATTAAATCTCTTTGGATCCATATTGATGCCGATCACACCCGATACCATTTGTATGCTGTACATGATTGTACTGGGTATTATTGGTATCGTACCTAGTATAATCATGTTGGTATGCATGATGGTACTAGGTGCAATCAGTATCGTAGATTTTTTTCTTCTTTTAAGATAATTTTTATTTCAAATTTGCTGTTGATCAAGTCCAGAGTCCTTGATAATTTATTTAATTATTTATATGTATTTATTGGGGCAACAACAGTTGGAGCAGCAGATTGTGGCAGCAGGTGGAGCAATTCCAATATTCGAATAAAAACATAAGCAAACAATAATACTAGGAATTAAGCTAAGGCTGTGGTGTGGACAACTTTGTGAGTTGCTGTGTTTAGTAACAAAATTACTAATTAAATTAAGAGGTTCTCTGAGCTACTCAGATCTGTGCTTCTTGTACAATTTGGTGGCCATGTCATGCAGTGCTATCATCCTTTTTATGCAGTGATTTTTGTCATTCTGAACCCCAATGCTGCTGCTGCTGCTTATTGATGAAAATGAAATTGGTTTCTGATAACTTGTGAGCTATTATGGGCTATAAGATGAACCTTATTTCAATTCATTTTTACTGTGATCTTTTTTAAAGTTTGAACTCCAAACTAGAGTCCCTCACTGCTTCTACCCTGGAGACAGGGGGAGGTAAAGCCCCTTACTGCTTATACCTTGGAGACGGGTGGGGGAGATAAGTCACGGGATATGTCTCAAGTGACCTACAAGCTCCTAAATGCTTGTACACTAGTGCCCACAACAAAGTGA

>IbHP3

ATGGAAGTTGTAGGACAGCTGCAGAAACAGTTTGTAGCGTACATGGCTTCCCTGTATCGTGAGGTCAGTGCCTTGCTCGACTCTGCAACAGTTTCAAGATTCTATTTTTCATAATTTTTCAGCATTTTAGTGATGTGGGTTTTTTGTGTTAAAGGGTTTCTGATTCTGGGTTGGTGTTGTTTCCCTGTTTTGAACCAAAAAAATTAGGGCTTTTTGGATGATCAGTTTCTGCAGCTTCAGAAACTGCAAGATCAGAGCAACCCAGATTTTGTGGTTGAAGTGGTTTCTCTTTTCTTTGAGGATTCTGAGAAGCTTATCAATAATATGGCCAATGCCTTGTGAGAATCACTTCTGCCCTTTAATTTCTCTTGAATATTTCTTTTTCTGCCTTGTTAAAATGGGTATTTTGAGTTTAATAAGTCTGTTTTTTTTACAGTCAGCAACAAGTTGTAGATTTTAAGCAGGTTGATGCCCATGTCCATCAGCTCAAAGGTAGCAGTTCCAGGTACATATCCTTGGACTATTTGTCTTTGGTTGTTAATCATTTGCTGTTTATGGTACTTTTTGTCTGAGATTTCTTCAGCTGGTTAGTAGTCATATGAAGTTGGGATTAATTAATTGATGCTAATATCAATCTCCAACTATCCCTTGCTGGTGGCTTGAGTAGATCTAAATGGAATATTTAGTAATGAAGTTAATAATACTTTATGTAAACTGCTGTTTTGAACTATTTCATTGGTTTTCCTCAGCATAGGTGCACAAAGAGTAAAGAACGCTTGTGTTTCTTTCAGAAACCATTGCGAGGAGAGGAGTCTTGATGGGTGA

>IbHP5

ATGGAAAGAAACCACTTGCCTAGGCAGCTTGCCACCATGAGGAAGTCTCTTTTTGATCAGGTAATTACTTCTTCTTATTCCTTACTTTACTTTAATTAACTTAGGGTGGGTTCGAGTCTCAGTGGAGACGACTGTTGACTTTGTGCTTCAGTTGGTTGAGAAAGTAGCTATGAACAGATACTACATTGTAACAAAGTCAGTAGTACTAAAAAAACACTCTTATTATGTTAAGTTGTATAGTAAAAATTTCAAGATCAGGCTTTATATATCCAATAATATACACATGTGGGCACTCAATATATATTATTGGTGCAGGGGTATCTAGATGATCAATTTGTTCAGTTGGAAGAGCTGCAAGATGATGTTAACCCTAACTTTGCAGAGGAAGTTGTCACTTTATTCTATCGAGATTCTGCTCGCTTAGTGCAAAACATTGAACATGCATTGTAAGTATTATTAATTAAATTACCCCTTTAAGCTAAAGATATGATAAACCGCGTATATCTTAAAATATATTTTTTGGTACATCGGAAGGATTGAGAATCAAATATGACATTACATTCTAACCTAAAGTTATAAGTTGGTGCACGTCACTCGACCACGTACACGTCTTAGCTATCAATACTAAGATATTAAGATACAAGAGTTTGCCGCCTTTATTGAAACTTTTGCCTTTAATCAAGGATTTGATCCCCATTTAACTTTAACGTCATCATATCATTATTTGTATAGCTTGTTAGATCGACCTAGCTCTTTTAATTTCACTTTCTCATCATAAATGTATAATATTAAAATACTTCACACAAAATTACTTATTTCTAATGAAACTCAGCTTATTTAACAACCACCTTCAATTTTTTTTCATTAAGATAAATTATCATCGACTTATTAACGTCACTTGAATTGAATAGAAATTTTCGATTTTCATAGGGAGAGAAGTCCTCTTGACTTTGCTAAGCTGGATGGGTTGATGCACCAGTTCAAGGACAGTTGCTCAAGGTTTGTGCAAATTAAAACAATGTATACATGCATGGCTACAATATAATGGTGAAATCTAAGTAATAATAATCTTATAATTTCTTCTTTGTTTTTGTGTGCAGCATTGGAGCCAGGAAGGTGAAATATGAATGCACGCAGTTCAGGGAGCATTGCAGGGTGGCAAATGCAGAGGGGTACATAATAATTTTCATCATACTTAGTATCTTAATTACTTTCTTGCATGTTAATTATCATTTCATAAGGTAAATATTTTAGACATGACTTTATTTGATTAACTTATAGTGGGGTCGGTTTGTTGGTTGGTTTGCAGATGCAAGAGAAGTTTCCTGCAGCTAAAGAAAGAATATTCCACACTCCAAAAGAAGCTCAAAGCTTATTTTCAGGTGCATAAATTCTCTCTACTACTAAATAATTGAAAATTTGTTTATTTTTTCATATGATTATGTTTAATGTAGTTTTTGTGATTGATGCAGTTTGCAAGACAAGCTGGGCCTGTTGAGGTGGCATGTCGCCCTAACTGA

>IbHP4

ATGGGATACATTGACGATCAATTCATTCAGCTTGAAGAATTGCAAGATGATGCTAACCCTAATTTTGTTGAGGAAGTTGTCAGATTGTTCTACAACGATTCAACTAGGCAGATTCACAACATAGAATTGGCACTGTAAGTATTTTTTAAAATTAGTATTATTATTATTATTATTATTATTTAAATGTGTGTATATAATACACAAATAATATATTTTCGTTTTTGTTGTATAGGGGAAGTGGAGCTTGTGATTTTACTAAGCTTGATGATATGATGCATCAGTTCAAGGGAAGCTGCTCAAGGTGATAAATTAATCATTTTTCATTAATTTTCCAAAAAAAAATCATTTTTCATTAATTCTGAGAATATTTATGCCGCAGAATATACTAGTACTTTTTGTATCAAGTATAACCATGCATGTTATTTAAATTACATCGTACGGGATACTTGGTAGCACTTGTTACAACAATACTCATTATTTAGTAGCGTTAAATATACCATGAAAATAAGTGGTCGAGCAGGGTGAAACATGATTCTCGTATCAAGATGTCACGAGTTTGATTTCTACTGTAATTATCGACACTGAAAAATTACTTTAGAAATTTAATAAGTTTGGACATACTCAATTTGCAGTATTGGTGCCAGAAAGGTGAAGAAGGAATGCTCAGAATTTCAGCAATACTGTGATGCTGGAAATGTTGAAGGGTATGTACCTTCAGCCCTGTTTTTTTAGGACAATTATATTATAACATCTTAAATAATACTGTCATTTTACGTGTGATTTTACCTAATTAACTTGTTATAAGATTTGTCAAATAACATATAACAAACGAATATACAAAAAATCATAAGAGAAATATATATATACAATGTAATATGATAATGTTATATTGCAGCTGCAGGAGGGCATTCCAACGATTGAAGCAAGAATATTACACTCTTGAGGCAAAACTTGACACTTACTTTCAGGTCTCTCTCATTACATATACATGCAATGCATATATATACACGCTCGCGCACACACACATATGTGTGTAATATTATATATAAATAATATTACGCGATCTAACTTTGGGTATTCGAACTTGAACTTTTCAGATGGCAAAACAAGATTCTTGA

>IbHP6

ATGTTGGGTTTGGGTGCGGAGCGGTTGCGAGTCGACATGAATCGCTTGCTCGCCCTACTCTTTCACCAGGTTTCAATCTCCATCTCATATGCTTTAATATAAATTTGTCTTCTCCTAGCTTTATAATTCTTCATGTTAGTTGTTTTTTTAAGGTAAGAATTAAGGTGGTAATTGTGTGGATATATATGTAGGGAGTGTTGGACGAGCAATTCTTGCAACTACAACAGCTCCAAGATGAAGCTTCTCCCAACTTTGTCTCCGAGGTTGTCAACATTTACTTCCATGAATCCGAGAAGCTCCTCAGGAATCTCAGAGTTTTGCTGTAACCACTACTCTCTCTCTCTCTCTCTTTCTCTCTCTCTAGGAAGCTGTGTGTGTATGGGTAAAAGGAATTTTTGGTGGTGATGGATGGGCATATTCTTTTGTTAAGTAGCTATGCATGGGGAAAAGATAGGGAACAAAAAGTATATATGTTTAGCGACACCCCTGCCCTTTTCATTAATTATTGTTTGTTTCTTGCCCATGCATACATAATAATCTTTCCTGTCGTCTACGTTATACTATACGTACGTACAACGTTAATTATAATGTTAAAAATTAAAAATAATGGGTCAAAGTTGGTCATACGGTTAATTTGATATAACTATAATGGTACAATTCGACTCTCAGCAGGAACAGTAATCTATTGATTTTTTTGTTTGAGACAGCTAGTCAATTATGGGCTGAATTACTTAGTATATACTATCCAGTAGTGACGGGTAACTGAAAATAATGAATAATGGTATTACATATATATATATATATATATATATATATATATAAATGCAGGATGGAGAGTGAAATATGGGACTACAAGAAAATGGGAATGCATTTGAACCAGTTGATGGGAAGCAGCTCCAGCATTGGAGCCAAACGAGTGAGAAATGTATGCGTGGCCTTTCGTGCTGCCGCCGAACAAAACAACCGTTTTGGGTTCGTGTCCCTTCGCTCCATTAATTTTGCATTAATTAATCTCGTACACATGTCGAAGTTGAACTGTACTGTCCTATATATTATAATTCAGGTGTTTGAGAGCCTTAGAGGCACTTGACCATGAATACTGCTATCTCAAGAACAAACTCGTAGAACTATTCCAGCTAGAGCAACAGCGGGTTTTAGCAGCTGGAGTGAGATACCCACTACTTCATCTTCCTCAGACTAATTAGAAGCTAAGCTATATATCAACCATGGCGGCCAGTCAAAATGGGAGACATAATTTATTTTATTTGATGTAATTAATTCGTTGTCGTACGTAGTAATTAGCATGGTTTTAGGTGGTCGGTCGAATGAGAATGTCTATCTGTCTGTGTTGGTTCAATTTCATTGCGCCGAAATGAAAAAAAATAAGTGTGGGTTTAGCAGCAAAATCAGATCTTTTATTTATGACTTACCAATGACCAATCCAATCGTATCAGACATCTCATGCATTGGATTCAATAGCCGTACTTGCTACGCCTATCTGATCTACTCTATTTAATGAATTTGACAATTATTCTGAGAACACATCTCCCAGATTCACTACCCATCATTTGTACTTTTTGTTTGCTTGCTTTTGCCAAATCTTCCAAAGGATATGATCCTTCAAATATCTATTTATTCCTAATTTATTTTTATAGGTCTGATCTAATATATTTAATTGTTCATTTATATTGACTTAATACCCTCTATTGGCTTAGTGTCTTTTAAAGTATTAAAATATCATTTTTAAAATAGTTAATGAATATTAATGCACTAATATATTTATTAGTCCTGGACAAGCTAAAAGTCAAGAAGGGAGTTGTATAGATTTTTAAACTTTTTTTTAAAAATAGTTCTGAGTTGTATAGATTTTTATTTATTTTTTTTAAAATAGTTCTAAGTGTGCTAAAAATTTGAAGTCAAAGTAAAGTAAATTTCCTTACCGCAAAAGTCTTTTTAGCTTGCCAACTAAAATTATATTGCAGCTAAGTTTGATGATAAAGTGTGATGTGTCTATGGATTATCGGGTTAGTTACACTGAATAAGCTGTATAAATGGAATGTAAAGTACAAAAGAGATTTAAGGGTCGTTTACTTCATTTCTCTGTTTTTTCTTTTTCAGTTTTTCATTTTTTTCATTTTCTAATTTTTCATCTCCATTTCTCCAATTTTTTTTGGCTGAAAAGTTATCGGAATGTTGCCTGAAACCTAATGACATGTGATATCAGGTCATTAACCGGTTTTTGAGCTTCTTTGAATCAAAATAGATTGTTTATGGGTGTAATTACACTTTTAAAAAGTTTAGGGGGCTAATTGACTTTTCAGCTAAAGTTTGAGGGTCTATTTGACCATTTTCTTTTAATACTTTAAAAGCTCAAGAAAATATTAATTTTAAATGAATTTAAAAATACCAAGATGGAATATTTGTGGCAAAAATCTCGACAGAAAAATAATTCCGTTTTTTTTTTTTTTTTCGATGGAAATTCTTCTCCAAAATGGTGTCGGAATAAGCGACGGAATTATTTTCCATCGCCAAATTGCGACGTAAAAAAATTCCATCGCTATATCGCGATGGATATTTCCGCGTGAGCGGATTCCATCGCGAAAATCCATTTCAGCGACGAATTTTTCACACGAATCGTGATCTTTCTTGTAGTGAAAGCTTGATGATGATGGCTTTATTGTCTCTTTGTGATAGACATTGGAAGATCACTAAAGGTTCACTGATTATTACACGAGGTAATAAGTCTTCTAACTTATACTATTTACATACTTCTATTTCTAGTGGTTCAGTGAATGTGGCAGGAAAAGAATGTGCATCAGAATTGTGGCATAAACGTCTTTGTCACATGAGTGTGAAGAGGATTGATGATTACCTTGCCAAAAAGAGTAAGCTTTATGGGGTAAAGGACGCTAGATTGGATAGATGTGTTCACTGCTTGGCTGGAAGAGTTTCGTTCATGAGCCATCCTCCTTCAAAGATGTTAGAGCCACTTGATGAGTTGATACATTTTGATGTGTGTGGACCAATGAAGGTTAGGTCACTTGGAGGAACATCTTACTTTGCATTGTGACCTTTATTGATGATTGTTCTAGGAAGCTTTGGGTCACACTTTGAAGCACAAAAATGATGTGCTTGGTGTGTTAAAGAATTTCATGCTCTTGCTGAGAGACAGAAAGTAAAGAAATTAAAGTGCATCCGTACTGATAATAGAAGTGAATATCGTGGCCCATTTGATGAGTATTGTAGAATGCATGGTATTAGAAATAAAAAAAACTTGTCCTAAAACTCCTCAGTTAAATGGCTTAGTAGAGAAGATGAATAGAACAATTATTGATAAGTGTCAAAGACATTCATATTTAAGGGTCTTTATGGGGTCATTTTATGCTCAATTCCAATCCTTTGTGGTTGATTCATGGTTATAATTGCTTGAATGGGCTAATGTGTTGCAAAGCTCTATCATATATTGCACTTTAGTTGTCTTGAAGGGTTTTGAACTTAAGGGAAGGCATATTGACTTAGGAGCAACCAAGGAAGAATCACATGAAGGGAGCCTGTCAATGAAGGGTCCACAGTCGTGGATGGAGAATAGAAGGTTCCACATCCAGGCCCACGATTGTGGATGGGAACGTGGCTAGGATAAGTGGCACTGTCCACGAGCGCTCCATGCTCATGGACAGGTTCGTGGACGGGAATCGGCGGAAGCAATTGGAGAATGTGCTTGGAGGGAAGACTATTTAA

>IbHP10

ATGGACGTCGATCTCCTCCAACATCAGCTCATTGCACACATTCAAGCTTTGCAGCGTGAAGTAATGATATTCATCGTCTCATATAAGTGTTTTTTGTTCGTTTTTTTTTTTGTTTAATTTTATGATGTCTTCCTTTTCGATCTGGTTTATGAATTTGCTGGAGCTTTCTGTTTTTTTCCTTGTTGTTCTGGATTTGAGCTGGATTTCAGGTTTTTGAGGCGAAGAATACTACTTGCGCACACGTACTATACGTACTGATTCTCATTTATTACGTTGCAGGGCTATGTTGATGAGTATCTGCAGATATGCTATGGGTTGAAGGAGACTTCTGGCTTAACATTTTTCCTAGAATTGATTGCTACCTTTCTCACAAACTCTGCTGCTACTATACATGATATGACTCAAACTATGTAACTTCAATTCGATAATCTGCCCTAAAACTTATTTTTTCCTCCAAAAACACTAATGTTTTTTGAGTTAATAATATAGTCGCCAGAGTGGTTTCTTCAATTTAGTCCTAAGTGATTTTTTGTACGTCATTGACTTTAATGGTTTTACCCACTTTAGTCCTCTATTAAGAATTCTGTTTGTTGAGTGTTAATAATAAGGTTAATATGGTAATTTCATTTCTATTTTATTTTTTTATCAAATTAATTATTAATTATATGTCTTAATTTCTCGATAGCTTCCAGTGAGTTTTCTGTGTGTTTATTGAGGAAGGAAGGGAGAATTGTATCGGCCGAAATTGAGAAGAGTGAGGGGAAGGGGGGAATTGTATGGGCGACAGAAGAAGGAGAAGAGGGAGAGAAATTAGGACATATAATTAATAATAAATTTGAAAACAAAAATAAAATAAAAATAAAATTCCCATGTTAACCTTGTTATTAATACTCAACAAACAGAATTCTTAATAGAGGACTAAAGTAGGTAAAACCATTAAAATCGATGACTAAATAGAGTACAAAAAGTCACTTAGGACTAAATTGAGTAAAACCACCATAGTCGATGACTATATTGGGTATTAACTCAATGTTTTTTTGATTGCATATAGGGAATACCCGATTCTTGACTATGATAAGATGCAAAGGCTTGCAATCAGGCTCAAGGGAAGTTCATCATGGTAACTACTTCAACACTATTTCTTTTCTTACTAAACAAATTAAAACCCAGATTATGTTATATATTACTAAGTTCTAATCGATCATATCATGGAACTTAATTGTATATACAGCATTGGGGCATGTCGGATAAGTGCTAGTTGCTCTGAGTTACGCCAGGCAGCTACAAAAAGATCTAAGATAAAGTATGGGATCATTATTATTATTATTATTTTAATTACAATAAAATTTTATAATTGAACTGTGTGTCATTATTTTCCGTTACGTTTTATATCATCAGCTGCAAACGGGTAGTGGAGATGATCAACGGAGAAAAATCCGCATGGGAAATCAAGTTGGAAACTATTATGCAGGTGACATGAATGAAGTTTTATTATATTATACAGTATTGTCTATTTGTCTACACATGTATGTATTAATATTTTGTTATCTTACATTCTTGGATGCGTTACGTTCTTGCTTTCAGCTGGAGCGTCAGATCGTTGACAAACAGATGCAGTGA

>IbHP11

ATGGACGTCGATCTCCTCCAACAGCAGCTCATCGCACACATTCAAGCTTTGCAGCGTGAAGTAATGATATTCATCGTCTCATATAAGTGTTTTTGTTTTTGTTTTTGTTTAATTTTCTGATGTCTTCCTTTTCGATCTGGTTTATGAATTTGCTGGAGCTTTCTGTTTTTTTTTTTCATTGTTGTTCTGGATTTGAGATGGATTTCAGGTTTTTGAGGCGAAGAATACTACTTGCGCACACGTACTATACGTACTGATTCTCATTTATTACGTTGCAGGGCTACGTTGATGAGTATCTGCAGATATGCTATGGGCTGAAGGAGACTTCTGGCTTAACATTTTTCCTAGAATTGATTGCTACCTTTCTCACAAACTCTGCTGCTACTATACATGATATGACTCAAACTATGTAACTTCAATTCGATCTGCCCTAAAACTTATTTTTTCCTCCAAAATACTAATGTTTTTTGAGTTAATACCCAATATAATCATCGACTATAGTGGTTTTACTTAATTTAGTCCTAAGTGATTTTTTGTACTCTATTTAGTCCTGGACTTTAATGATTTTACCCACTTTAGTCCTCTGTTAAGAATTCGGTTTGTTGGGAGTTAATAAGAAGGTTAACATGGTAATTTCATTTCTATCTTATTTTTATCAAATTAATTATTAATTATATGTCATAATTTCTCTCCATCTTTTGCATAGTTGATACAGTTCCCTCATTCCCTTCCTCCGGCATAAAAACGTATTACAGACACATTGGCAGATAGCTTCCGGTGAGTTTGCTGTGTGTTTATTGGGTTTGAGGAAGGAAGAGAGAATTGTATCGGCATAAACTGCGAAGAGTGAGGGGGAGGGGGAATTGTATGGGTGACAGAAGATGGAGAAGAGTGAGAGAAATTAGGACATATAATTAATAATTAATTTGATAAAAAAAATAAAATAGAAATAAAATTACAATGTTAACCTTGTTATTAACACCCAACAATCAAAATTCTTAACAGAGGACTAAAGTGGGTAAAACCATTAAAGTCTAGGACTAAATAGAGTACAAAAAGTCACTTAAGACTAAATTGAGTAAAACCACCATAGTCGATGACCATATTAGGTATTAACTCAATGTTTTTTTAATTGCATGTAGGGAATACCCGATTCTTGACTATGATAAGATGCAAAGGCTTGCAATCAGGCTCAAGGGAAGCTCAGCATGGTAACTACTTCAACACTATTTCTTTTCTCACTGAACAAATTAAAACCCGGATTATGTTATATATTACTAAGTTCTAATCATATCATGGAACTTAATTGTATATACAGCATTGGGGCATGTCGGATAAGTGCTAGTTGCTCTGAGTTACGCCAGGCAGCTATAAAAAGATCTAAGATAAAGTATGGGATCATTATTATTATTATTATTTTAATTACAATAAAATTTTATAATTGAACTGTGTGTCATTATTTTCCGTTACGTTTTATATCATCAGCTGCAAACGGGCAGTGGAGATGATCAGCGGAGAAAAATCCGCCTGGGAAATCAAGTTGGAAACTATTATGCAGGTGACATGAATGAAGTTTTATTATATTATACAGTATTGTCTATTTGTCTACACATGTATGTATTAATATTTTGTTATCTTCCATTCTTGGATGCATGCATTACGTTCTTGCTATCAGCTGGAGCGTCAGATCGTTGACAGACAGATGCAGTGA

>IbHP7

ATGGATGATTCAAGCACCTTTCCTCAGGCCGCCTCGGGTTTGTTTTTTACACTTTATGCATACTCAACGCTCAAACTCTCTCCATTCTCTGTTTAACTTTTTTTTTTGTTAATTGCTTCATTCTCTGATCTCTAGGGTCCGCTAGTGCGAATGGAAAGACCAGAGTGGATGTTCTCAAACAACAAGTGATTGCTCACATTGAACAGCTGCAGCGAGAAGTATCGATCGATTCATTTTATGATGATCAGATATCAGTTTTTCTTCGTTTTTGTTGCTTCTTCAATTTTGGTTTATATTATGAATTTGCGGTTCGATTCTGATTTGTGTTGCGAAATGGAGTGCAGGGCTTTGTTGATGAGTATTTCAGGATGAGCTACGGGCTGAAGGACGATACTTCTGAGAAAACATTCTTCATAGATTTGGTTTCTACGTTTCTGATGGAGATTGCCGCCACCATAGATGATATGATTGATAGTCTGTAAGTTTTTTGCTATATGAATCCAGTTTAATTTGTTCCCCTACCTAAAATTAATTAATTTCAATATATAGAAGCAAGTTTTATATATATATATATATATATATATATATATATATATACATAACATTTTGATGGAGTGCATCGCATGTTTTAGGGAGTATCCAATCATTGACTATGACAAACTGTATCAGCTTTCCATGAAGTTGAAAGGGAGTTCATCATTGTAAGTGTAATCCATTTCTCAATAATATTTGTTCCTATACAACAATATAATCCATTTATTTTTGTACGACTCACTTGCTTAATTGTATATGTAGCATTGGGGCTTGCCAGCTTTCAACTGGTTGCATCAACTTGATTCATGCCATTGTCAACAAATATATGAATGAGTAAGAAAGCTTTTCTACCTCAAAATTAATATATATATATATACCACACAAAACTAGTACGTATATCTTTCTCATTATTCGTGAATGTTTATTATCTTATCGTTTATTGTTTTCCCTCTTCATTCTATCAGGTGCAGGCGGATAGTGGAAAAGCTCAATAAGGACAAAGTTGTCTTGGAAATGAAGTTGGTGGAGGTTATGGAGCTGGAGCATGAGATTGTTGCAGACGGTGAATGA

>IbHP8

ATGGATGCTTCAAGCACCTTTCCTCAGGCCACCTCCGGTTTGTTTTTTACACTTTTTGCATACACAACGCTCAAACTATCTCCATTCTCTGTTTAACTTTTTTTTTTGCCGACATCGCTTCATTCTCTCGATCTCTGTGGTCCGCTAATGGAAAGACTGATGGATGTTTCTCAAACAACAAGTGATTGCTCACATTGAACAGCTGCAGCGAGAAGTATCGATCGATTCATTTTATGATGATCAGATATCAGTTTTTCTTCGTTTTTGTTGCTCTTCAATTTTGGTTTATATTATGAATTTGCGGTTCGATTCTCGATTTGTGTTTAAATGGAGCGTGGCTTTGTTGATGAGTATTTCAGGATGAGCTACTGGCTGAAGGACGATACTTCTAAGAAAACATTCTTCATAGATTTGGTTTCTACGTTTGTGATGGAGATGACGCCACCATAGATGATATGATTGATACTGAAGTTTTTCTCGCTATATGAATCCAGCTTTAATTTGTTCACCTACCTAAAATTAATTAATTTCAATATATAGAAGCAAGTTTTATATATATATATATATATATATATATATATATATATATATATATATATATATATATATATATATATATATACATAACATTTTGATGGAGTATGCATGTTTTAGGGAGTATCCAATCATCGACTATGACAAAGACGCATTTCCATGAAGTTGAAAGGGAGTTCATCATTGTAAGTGTAATCCTATTTCTCAATAATATTTGTTCCTATACAACAATATAATCCATTTATTTTGTACCACTCACTTGCTTAATCAGATATGCAAAGATTGGGTTCAGCCTTTCAACCTGGTTGCATCAACTTGATTCATGCCATTGTCAACAAATATATGAATGAGTAAGAAAGCTTTTCTACCTCAAAATTAATTATATATATATATATATATATATATATATATACCACAAAACTAGACGATATCTTTGTCATTATTCGTGAATGTTTATTATCTTATCGCTTTTGTTTTCCCTCTTCATTCTATCAAAAGCGATAGTGGAAAAGCTCAATAAGGACAAAGTTGCCTTGAAATGAAGTTGGTGGAGGTTATGAAGCCGGAGCATGAGATTGTTGCAGACAGTGAATGA

>IbHP9

ATGGACCTCGCTCTCCTTCAGCAGCACCTCATTGCACACATTGAAGCTTTGCAACGTGAAGTAATTATATTCAATCATCCTTGCATATCAGTTTATCTGAACCTCCTTTTTTTTTTTTTTGGTTTATATATGAATTTGTGGAGCTTAATTTCAGTTTTTTCCTTTTTGTTCTGGAATTGATGGAGTTGGATTTCAGGTTTTAATTTTGAGGGGAAGAAATTGATCTGTGTTCGACAATCATTCCATTTAAGCTGATATGTCTGTGTGTATGTATGATCATGTATATGCATATGTATATGTATATGTGTGTGTATCTACACACACAGGTCCTATATATATGTGCTCATATATCATATTACAACGCTAAGATGAGTACAAATTTTACATTTTTTTGAAAATAAAAATGTTTTTTTTTTAAAATTCAAATTAATATTTTTTTTTTTGTAATTTTTCTTCAAATAAAAATAAAAACTGTTGGATATGAAATCTGTATAGTTACTTGTTTATGAAGCATTTGGTATGTGCCACTATGTTGAATAAGGTTTTTGAGGAAACAATGCATATGTGGAAGAGGGAGGGTGAGGGTGAGGAGAGAGAAGTAAAGGGAGCTTGGTAATTGTCGGCTATGTGGGCCCCACAAAAAATGCAACAATACTTAAAAGAAAGCCTCACGTGAGGCACCAATCTCGCGTCCAGCCCAAATCTTTATTTATTTATTTATTTTTTAATTTTTTCCCTCTTTCATTTTTTCTTTTCTAGTCACACCCTACAAAAATTCCTAGAGGAGATTCTCTGACAGACATATATGCATCTATATATCATATATAGGGTTTACAAATAGCTAGGAAGATTTCATTATGTAGAAAACATTGTAAAATTAGGTTAACAATGGTTGCGCACATACTGATGATTCTGGTTTGTTGCAGGGTTATGTTGATCAGTATCTGCTGATGTGCTATGGGCTGAAAGAGACTTCTGGCATAACATTCTTCCTAGAATTGATTGTTAATTTTCTCAGAGAAGCTGCTGCTGACATATATAATATGACTGCCACTGTGTAAGTTCTGACCTACCAGTTTTTTTTTTTCTCTGAAAAATATTAGGCTTGTGTTAAAAAAGATAAATATGTATATATGTTTTTTACTGCATGTAGTGGATATCCGATTCTGGACTATCATAAGATGCATGAGCTTGCCATCAAGCTCAAGGGAAGTTCTTCATGGTAAGTACTTGAACACAAAACTTTGATTATATATATATATATACATATATATATATATATATATATCTATACATATGTATGTACATATATGTATGTATATATACATACATATATATATATATATATATATATATATATATATATATATATATATATATATATATATATATATATATATATATATATATATATATACATATAAACTTGTGTGAGACCGTCTCACCAATTCTTATCTGTGAGACAAGTCCGGTCAAGATGAAATACAATATGTATACTAGCAAATGTAGTACTAAATCATAAATAAAATGTTTACTACTTATATGAAAAAATAGAATATTTTTATATTTTGAGGTAAAAGTATTACATTTTCATTTATAAGTGCAACATTACTTATAAGGAAAATATAATATTTTTGATGAAAATAGTAATACTTTTACATCACAGAGACAAAAGTATTACGTTTGTGTTTTCTCAAGTAACAAACATTATTTTATTTTCCTGATTTAGTATTACATTTGCATATTGACCCGATCCACAGATCTAGTGAAAGTGAGATCATCTCTTCCTGTGAATCTGTGAGACTGCACTCAATCTACTATACGGATGCACACAAACTACTCCATAGTAGATTGTGTCCATTCATGTTGACAAAATGCACACAATCTAGTTTGTGTGCATTCGTATAATATATATATATATATATATATATATATATATATATATATGATGATATTTGATACTAAGGTATATATAGTTATATACTACTCATCAGTTCTAATCATGGAGCTTAATTGTATATGCAGCATTGGGGCTTGTAGGATAAGCGTTGCTTGCACTCAGTTACTCCAGGAAATTAGTAAAAGATCTGAGATAGAGTATGGCTGGATTATTAATTTTATTTTTCAAACAAATTCACTGCCCAACAATATAAAATCTTAAAATTGAAGTGTGTCTTTGTTTTCTTTCTCATATATATCAGCTGCAAACTGGCAGTGGAGATGATCATCACAGAAAAATCCACATTGGAAATCAAGCTGGATACTATTATTAAGGTGACATGATGAAGTTTATATGCAAGATTTATTATATTATACAGCATTGTCTTTTGAATACTACTAACTCTATTACAATGCAGTATCTGTTCATAACTACTTTCTATACAACATTGTCTAAGCATTCTTAGATGCGTTTATGTTCTTGTTTTCAGCTGGAGCGTGAGATCGTTGACAGACAGTTACAGTGA

>g47182

ATGGCGAGGAACGGGGTGTTTTCGCGGCGGCGGACGGCGGCGGAGATGGAGGATTCCGACGAGGTGGTGCTGTCGTCGGAATCTCACGATGTTCATGTTCTCGCCGTCGACGACAGCCTCGTCGATCGGAAAGTCATTGAAAAGTTGCTGAAAATTACGGCCTGCAAAGGTTTGGAATGTTTAATGTTAAACTGAGATTTTTGTGTTTTCTGTTTGATTTTTTGAGTGTAATTTGAAACTGAGAGTTTGGTTTTACAGTGACGACGGTGGATAGTGGGAGGAGAGCTCTGCAAATTCTGGGACTGGACGAGGAGAAAACCTCTGTCCAATTCGATGTAAGAATTTTTTTTAAGGTTGTTTTTTTTTTTGAATTGTTTATGTTTGGATTTTAAGGTTTAATTCAATGTTGAAATTCTAGGGTTTGAAGGTGGATCTGATAATCACAGATTATTGTATGCCTGGGATGACTGGCTATGAATTGCTCAAAAAGATTAAGGTCTTTTTTCTGCTCTATATTTCGATTTATTTTATTTTATTTTACTTTTGCTTGTAAGATTTTTTTTTTTATTTAAAAAAAACAGAGAATCAATTATCAGAAAATGAAGTTAATTGAAGATGTTCTCATTTTTTTTTTTTTTTGGGTGGTTGAAAATTTGGTTTTGTGAACAGGGTTCATCGTTTAGGGAAATACCAGTGGTGATCATGTCGTCCGAGAATGTTTTGGCACGAATAGACAGGTATTTAATTTTGCAAATTTCTTCGATTTCTTGGTGTTGTTCCTGAATTGGATCTAATCTGTTTGTGTGCCGATGTTTTGCAATATTTTTTTGAATTAGCATGTTGAATTTGTATCTGCATAAATGATAGTAGTTCATTTCGAGAATAAGAATTAGAGGTAGTTCGAGAATCTCGTATTAAAACTATTCCCGTGGTGTTTTATGTGTTAGTATAGTTCAGTTTCTCGTGTTAAAACCATCCCAGGATGTTTATGTCTTAGAGTTAAAGAATCTTGATTGTGTTTTTGACAAAAGAATCTTGATTGTGTTAAAACCTTTATCGGGGTGTTTATGTGTTAAGAGTTTCCAATAATTTCGTGTTCAAACCTTTCTCGGGATGTTTGTGTTAGAGTTCAAGAATCTATTAAAACCATCCCCGGGGCGTTTATATGTTAGAGTTCAATAATCTCGTGGTTAAAACCATTCCCAAAGTGTTTATGTTTTTGAATTCAACAATCTCGGGTTAAAACCTTTCCCGGGGTGTTTATGTGTTAAAATATTACCAATTTGAGCTCGGGAATAAAAGCAAGTTAACTAAAAAATTTGCTTCAAGTTTGGTTTTGGACTTAAATTCTGACGGGGCATAGTGATGCTAAATCTCGAAATGAATTAGGAAAGGGACAGGCATCTCTGTTCGTATCATTCTTCATGTGCTTTGAAGATTTGCTTTGTTGTTAGATTGATTAAAAAAATGAAGTTTGACTTGTTGGAGATTTATGAAAGCGGGATACGTTTCAACTAATCTTCTTAGAATGGACTATAACCAATGAACCAGCATCCACTTGCACATTACTTTAATGAACATAAGATACTATAATAATGGAGATCATGCAATAGAAAATCATATCCCAAGCATCCCACTGTCTGTTATCAACGAAGAATCATCATTCTGTCTTACAACACTTCAACTTTAGCCCCGTAAAACATCCCGAGTTTTGACAAAGCTCGAGTTTCATATCACTTCATTAGGAAGGATGTCCACGTGTTCGGTCTTAACTGCTTACCTAAAATGTTCTTGTTCTGGTTACTTTTTGCCTTTACTAGTAGGGGTGGGTGAAAAAAGTCGGTTAAGTCGATCGACTACGTAACTGAGGATTGATAATCCAATCGACTGAAGTTTTTGATTATTGGTCGGTTATCGATTATGAAGTTGAAAGTTTTTGATTTATTGGTTTTTTATTGGTTAGTTATAGAGCTGTCAATACGGGCCAATCCGTCACGGGTTGGCGAAATACAGGATGGGTTGGGATTTCTCCGACCCAACCCGTCCAAAACACGGGTTAAACGGGCCCAACCATGGCCCGTTTTGACAGCTCTAGTCGGTTATGGACTTACGGCTATCATGATGGCTAGAGTTCGGTGAGCACTTAAACTTAGGTTGTACAACAGCGGATTATTAGTTATGAAGTTAACAAAGACATTGATTATTTTAGTTTTTCAGTTGGTTACGGTTATCTTAAAATTCTTTCGGTTATTAACTGAAATTGACCTTTTGCTACTAAGTTATTTAAGGAAAGAAAGAGCCTTGTAAACATGGAAATGTTCCTCATAAAACCTTGTTTGTTTGGTCTTAATGCTATAACAGATGTTTGGAAGAAGGCGCCGAAGATTTTCTCTTAAAGCCGGTGAAATTGTCCGACGTTAAACGCTTGAAGAGCTACATGTTTGGCGACGACCGGTTTCATGGCGAAGACGGCGGGACAAACAAGCCGGAAACGCCAGAAATATCCGACGACACATCGTCATCATCGGCGCCATCTCTCGCTCTCGCCATCACCAACGACGTCAATGGATCTCTCTTCATGTCTCTCGCTCTCGCCATCTCCAACATCAACTGA

>g12608

ATGATGTCGTCGTCGTCGTTCAGCGTATCCCGCAAGAACTCCGGCCGCCGGGCGGCAGACACATGGGATCCAGAAACTCTTAGCAGCCAGCCGGCGAGCGGAGGAGACAACGGTGGGTTTACCTCAAAGTTGTCGAGCAAGATGAGCCAACAGTTTGAGGCGATTAAGGTGGGGTTTCGACGGATTAAGGAGAGCGAAAAGTTGGATAAGATTATGGTCTTAAGCTCCAAAGGCGTGGAGAAAGTGAAGGATGGGGCGTGTTCTGGTTTTCAGTGGATAAAGGACAAGTGCCGCAACACTGCTCACAATTCACGAGTCGTCATTGTATGATACATATATACAAGATGCAAAATAGCAAATAGATCAGATCTGTTCAGCTGTTCCACCTTTAGAGGTGTCTGCAATTCATCGTGTCTCCGGACAATAAAAATTTTAGATTACACCGAAAAAATAGATCAGATGTAATATATATAGGATTGATGAGTTTTGCATTTAAGGTGCATGTTTTAATTTGTTATGTATGGATATTGAATTAATTAATTAATTAATTTTGGTATTTGTGTTTATTTGTGTTTCTCTTACAACAGAATGTTTTAAAAGAATACTTAATTATTAAGCAACAAGGAAAAGGAGGTGAATTTTTTTTTTTTGGGAATGTTTCATCTTTATTTAAACAAAGTTGTGAATCGGTTTTCTACCGGTGTTCTTGAACCAACTTAGGGCCTCGCATGCTCCTCGCTTAACATCTAACGAGCCACATGCATTCTTGCAAGGAAAGTACCATCATCATCCCGAAGGACCACCCAAGACCCATAACATTATGGTCCTGATGTATCGCGACAACAGAATGTTTTATACTCCGTAGTTATTAGCAACAAGTTTAATTAAGAATACATAACCACATCATATATGCAACAAAAATTCAGACTAAACGGTACGAGTTATTGGGCCTATGGACACTACTGGGCTCAAGAGGAAGAAAATTGATAATGGGCCTACTCACATTGCTGAAGTCTGCTGGATGGAGGAAGTATTTAGCTCATCTACTGGGCCAAAAACTTGCAATGGTAAGCCATTTAACCAAATCTCCGTGATAAGTCAGCGTCGAATTGGCTTAATATGGCGGCGTAACATCATCTCACTGAATGGTCAGAAGTGCATGACAAAGATGATTTCCAACCTTCACACCATTCCAACTTCATTGGCAAAAACAAAATGCAGGCTGGCTCAAGATTAATGTGGATGTTGTCGTAGACACTATCGAGGGACGAATGCGTTTCAATGACTTGTAAGAGATGATCAAAGGTTTCTATATTAATTCAGGAATTATTTCTATATCTGACTGTAAGGAGGAGACTGTCTTCCCTAACCATATATTTACAAAGGTTTGATTTTGGCTAAGTTGTCTAGTATCATTTGTATACTTAACATTTATAAGGAAAATTAATCTTATGGTTGATGCACCATATATTTACAAAGTATATTGATATTTTTCTTTTATCAATTTCATGATAACCAACAATTTATACGTAGTATAATATTTTGAATGAATATATTTACTATTTTTGTATTAATATAGTAATATTTTTACTGATAACATAAAAGTCAACAATTTATTTAACATTTTGAAGAAAAAAAGTTGCATTTACAATTTTTATAAGTGACTTGATAAATTAGTGTATATAATTCTTTAGAATTAAAATCTTAGTTTTTAAAATTAAAATTACATTAATTTAAGATTTTGACTACGAATTTATCGAGCAGGAATTCCCATCTCACCTAATTATCCACGAGCGTGGATGAGGACTATTTTAGTTTCCGTCGAGTATACTCCCGCCCATACCAGTTGTAATCCCTAATGAATAAAGAGATTATATTTAAGCTCTAAAATCAGTCACATTCACAGTTCTTCTCCTCAAATAAGGTAAAATGAGGAGGAAAATTGTGAATTTTGATCGATCTTAGGACTTAAATCCATTTATTCAGCTCAAATTGAGATATTAAGTTGAAAAGAACAAAAATACCCTTAACAATTTTAAATTTTTACACGTTTTAAACGGATGGGTGACCGGCGTGATGAATTTTTACACTAAAATAATACGGAGTAATTATGGATAAAAGTTAGTACTAAAACAATAGTCGTCGGATCAAAATTGATTGAAATTAAACATGTAGGCCAAAAATATCGTTTTACTAATAATCGTGTGGTAAAATTGGTATTTCTTCCGATTAAGAACATTAAACAAGTATAGAAGTGCTATGGGGAGTAGCTTTCCAAAAATGCCCTTGAATGTACACATTCAAAAATCAGAAGAGAAAGAGACGGCCTAGCCTTGTATATGTGCTGTGCGCGAGGAAAGATGGCAACGTGCATCGTATAGGAAATCGCACAGCAAATCATATAGGAAATCACAGAGATTCAGTTTCGATTTTGTTCTTGCCATATATCATATGAGCTTCAAATCACCTACACCACCATAATCTTTTGCTTTTTTTCCTGAAAAAGAAAAAGCAAGCTTGCGAATTCAGCTGTGTGGGAGAGATGGCGAGGAACGCGGTGTTTTCACGGTGGCGGAGGGCTGAGGGACCCGCCGGATTGTCGCTTCCCTCAGAATCTCACGACGTTCATGTCCTCGCCGTCGACGATAGCCTCGTCGACAGGAAAGTAATCGAGAAATTGCTCAAAATTACATCCTGCAAAGGTGGAATTTCGCCCTGAAAATTCGCAACTGCTTTGTTAGTTTCCATGTTCACTGAGATTGTAAGAAAAACAAATATTTGTTTGTTGCAGTGACAGCAGTGGATAGTGGGAGTAGAGCTCTGCAATTCTTAGGATTGGACAGGGAAGAGAGTTCTGTTGGATTTGATGTAAGTGAATTCCGTAAATTTGTTGATTTTTTTTTTTCTCTTTCATAATGTAGAATATTAATGGGTTTGGTTTCAGCAAAATTTGATTGATGATTGTTTTTGTGGATGTGAATTTAGGGTTTGAAGGTGGATATGATAATCACAGATTATTGCATGCCTGGAATGACTGGCTATGAATTGCTCAAAAAGATTAAGGTCCATTTTCTGTACTATATTTTTATTCGTTTCTATTTTGGCTTGTAAGAAATGATGATAAAAAACTGAACTTCAACTGGAAAAGTATTTGTGTTCATTTATTTTATTTTTTACCCAGTTTGTTTTTATGAACAGGGATCATCTTTTAGGGAAATTCCTGTTGTCATAATGTCATCTGAGAATGTTTTGGCAAGAATTGACAGGTATTTTTTAATGTACTAGTGTTGATTTCTTCAATGTTGCTTTGTTGTTGTTTCCATTTTGAGATTGCCATGAATTTACAAATGAATATTGGACTTTACTTCATTCAAGAACATGAAAGCCTCTTGTTAATCATTCGAGATGGTTTTGACTGTTTAACATTACTGTTTAAACCCTCCACAGGGTGTGAATGTTCTTGTTTGTTTCTTAAAGGAAAATAGAACGAAGAAGAGTAAATGCACGAAAACTGTAGATCCTATCATTCCCCTTCTTTCTTATTGAAATTTTGTTAGGAAAAAGGAGGTAAAGAGATGCAGAGAACTCCGATTGGTATTGCCATTTGTTATCAATAAATTGTTTGCTTGATTGACTTTAGCGAAACAGTTTGTTAATAGGTCGTCTTAGATGACTTAAATCAATAAACTGATTGCCACTTGTTCACTAGTTTAATGCATTTAACATACTATACTAGTGCGTTGGAGATCATGCAATAGAAAATCATATTCAGAGATTTCCAATGTTGTTTATCCATAAAGAATAGCCGTTTTGTATAAAAATTTAGTTCTATTGTGGTAAACTTTGAAGGAAATGCACATGATTTTGGGATCCCTCAATTCACCTACTTGAACTGTAAATATGTACTTTACTAAACAGATAAAAAAACAGAGTACTTTTCTGCCTAAATAGTTTTCTTTCCAGAGTTGCTTTTGTTCTGATGGTTTACTATAAACACATTGCACTTAGACGAACCGTTTTCGATGTAAAATCTTCTGTTGCCTTTATGTGATAATAGCAGATGTCTGGAAGAGGGGGCGGAAGATTTTTTGTTGAAGCCAGTGAAACTGTCGGATTTAAAACGATTGAAGAGTCACATGTTTGGCGAGGACGACAAGAATCCAAGAGAAGACAGCGCGATAAACAAGCGAAAGTTACAAGAAATGTCCGAGGATTCATCGCCTCCCTTGCCTTCACCATCACCATTGCTGTCTCCAAATCCGTCAACCGATCTTTCTTCTTCATCATCATCACCATCATCATCATCCTCCCCACCCTCCACTTCATCATCACCTTCCTCACCTGAACTACTTGAATCTTCCAAGACAGAGGAATGA

>g12082

ATGGACACTACTGGGCTCAAGAGGAAGAAAATTGATAATGGGCCTACTCACATTGCTGAAGTCTGCTGGATGGAGGAAGTATTTAGCTCATCTACTGGGCCAAAAACTTGCAATGGTAAGCCATTTAACCAAATCTCCGTGATAAGTCAGCGTCGAATTGGCTTAATATGGCGGCGTAACATCATCTCACTGAATGGTCAGAAGTGCATGACAAAGATGATTTCCAACCTTCACACCATTCCAACTTCATTGGCAAAAACAAAATGCAGGCTGGCTCAAGATTAATGTGGATGTTGTCGTAGACACTATCGAGGGACGAATGCGTTTCAATGACTTGTAAGAGATGATCAAAGGTTTCTATATTAATTCAGGAATTATTTCTATATCTGACTGTAAGGAGGAGACTGTCTTCCCTAACCATATATTTACAAAGGTTTGATTTTGGCTAAGTTGTCTAGTATCATTTGTATACTTAACATTTATAAGGAAAATTAATCTTATGGTTGATGCACCATATATTTACAAAGTATATTGATATTTTTCTTTTATCAATTTCATGATAACCAACAATTTATACGTAGTATAATATTTTGAATGAATATATTTACTATTTTTGTATTAATATAGTAATATTTTTACTGATAACATAAAAGTCAACAATTTATTTAACATTTTGAAGAAAAAAAGTTGCATTTACAATTTTTATAAGTGACTTGATAAATTAGTGTATATAATTCTTTAGAATTAAAATCTTAGTTTTTAAAATTAAAATTACATTAATTTAAGATTTTGACTACGAATTTATCGAGCAGGAATTCCCATCTCACCTAATTATCCACGAGCGTGGATGAGGACTATTTTAGTTTCCGTCGAGTATACTCCCGCCCATACCAGTTGTAATCCCTAATGAATAAAGAGATTATATTTAAGCTCTAAAATCAGTCACATTCACAGTTCTTCTCCTCAAATAAGGTAAAATGAGGAGGAAAATTGTGAATTTTGATCGATCTTAGGACTTAAATCCATTTATTCAGCTCAAATTGAGATATTAAGTTGAAAAGAACAAAAATACCCTTAACAATTTTAAATTTTTACACGTTTTAAACGGATGGGTGACCGGCGTGATGAATTTTTACACTAAAATAATACGGAGTAATTATGGATAAAAGTTAGTACTAAAACAATAGTCGTCGGATCAAAATTGATTGAAATTAAACATGTAGGCCAAAAATATCGTTTTACTAATAATCGTGTGGTAAAATTGGTATTTCTTCCGATTAAGAACATTAAACAAGTATAGAAGTGCTATGGGGAGTAGCTTTCCAAAAATGCCCTTGAATGTACACATTCAAAAATCAGAAGAGAAAGAGACGGCCTAGCCTTGTATATGTGCTGTGCGCGAGGAAAGATGGCAACGTGCATCGTATAGGAAATCGCACAGCAAATCATATAGGAAATCACAGAGATTCAGTTTCGATTTTGTTCTTGCCATATATCATATGAGCTTCAAATCACCTACACCACCATAATCTTTTGCTTTTTTTCCTGAAAAAGAAAAAGCAAGCTTGCGAATTCAGCTGTGTGGGAGAGATGGCGAGGAACGCGGTGTTTTCACGGTGGCGGAGGGCTGAGGGACCCGCCGGATTGTCGCTTCCCTCAGAATCTCACGACGTTCATGTCCTCGCCGTCGACGATAGCCTCGTCGACAGGAAAGTAATCGAGAAATTGCTCAAAATTACATCCTGCAAAGGTGGAATTTCGCCCTGAAAATTCGCAACTGCTTTGTTAGTTTCCATGTTCACTGAGATTGTAAGAAAAACAAATATTTGTTTGTTGCAGTGACAGCAGTGGATAGTGGGAGTAGAGCTCTGCAATTCTTAGGATTGGACAGGGAAGAGAGTTCTGTTGGATTTGATGTAAGTGAATTCCGTAAATTTGTTGATTTTTTTTTTTCTCTTTCATAATGTAGAATATTAATGGGTTTGGTTTCAGCAAAATTTGATTGATGATTGTTTTTGTGGATGTGAATTTAGGGTTTGAAGGTGGATATGATAATCACAGATTATTGCATGCCTGGAATGACTGGCTATGAATTGCTCAAAAAGATTAAGGTCCATTTTCTGTACTATATTTTTATTCGTTTCTATTTTGGCTTGTAAGAAATGATGATAAAAAACTGAACTTCAACTGGAAAAGTATTTGTGTTCATTTATTTTATTTTTTACCCAGTTTGTTTTTATGAACAGGGATCATCTTTTAGGGAAATTCCTGTTGTCATAATGTCATCTGAGAATGTTTTGGCAAGAATTGACAGGTATTTTTTAATGTACTAGTGTTGATTTCTTCAATGTTGCTTTGTTGTTGTTTCCATTTTGAGATTGCCATGAATTTACAAATGAATATTGGACTTTACTTCATTCAAGAACATGAAAGCCTCTTGTTAATCATTCGAGATGGTTTTGACTGTTTAACATTACTGTTTAAACCCTCCACAGGGTGTGAATGTTCTTGTTTGTTTCTTAAAGGAAAATAGAACGAAGAAGAGTAAATGCACGAAAACTGTAGATCCTATCATTCCCCTTCTTTCTTATTGAAATTTTGTTAGGAAAAAGGAGGTAAAGAGATGCAGAGAACTCCGATTGGTATTGCCATTTGTTATCAATAAATTGTTTGCTTGATTGACTTTAGCGAAACAGTTTGTTAATAGGTCGTCTTAGATGACTTAAATCAATAAACTGATTGCCACTTGTTCACTAGTTTAATGCATTTAACATACTATACTAGTGCGTTGGAGATCATGCAATAGAAAATCATATTCAGAGATTTCCAATGTTGTTTATCCATAAAGAATAGCCGTTTTGTATAAAAATTTAGTTCTATTGTGGTAAACTTTGAAGGAAATGCACATGATTTTGGGATCCCTCAATTCACCTACTTGAACTGTAAATATGTACTTTACTAAACAGATAAAAAAACAGAGTACTTTTCTGCCTAAATAGTTTTCTTTCCAGAGTTGCTTTTGTTCTGATGGTTTACTATAAACACATTGCACTTAGACGAACCGTTTTCGATGTAAAATCTTCTGTTGCCTTTATGTGATAATAGCAGATGTCTGGAAGAGGGGGCGGAAGATTTTTTGTTGAAGCCAGTGAAACTGTCGGATTTAAAACGATTGAAGAGTCACATGTTTGGCGAGGACGACAAGAATCCAAGAGAAGACAGCGCGATAAACAAGCGAAAGTTACAAGAAATGTCCGAGGATTCATCGCCTCCCTTGCCTTCACCATCACCATTGCTGTCTCCAAATCCGTCAACCGATCTTTCTTCTTCATCATCATCACCATCATCATCATCCTCCCCACCCTCCACTTCATCATCACCTTCCTCACCTGAACTACTTGAATCTTCCAAGACAGAGGAATGA

>g33858

ATGGTCATTGGGAAGCCGGAGAAAGTCGCCGCCGGTGATGATTGCTGTGCCGGCGGTGTACAGGAGTTGCATGTTCTTGCCGTCGATGATAGCCACGTGGACAGAAAGGTCATTGAGAGGTTGCTCAAAATCTCTGCCTGTAAAGGTTGGTTCTTTGAACCTTTTTTTTTAAATTTAATTTTTTTTATTGATTTCTGAAAGTGTTGAAATGTGGGGATTTGCAGTTACAGCAGTAGAGAGTGGGAGCAGAGCTCTACAATATCTGGGCTTGGATGGAGAGAAAGGCTCTGCTGCAATTGATGTAAGAATGAAAAGTTTTTTTTTTTTTTTAAATAATTTGGAATCAGAAAATGGGATTAAAGATTGAAGCTTTAAGATCTTTTGGGTGTTTGGTCTAAAGCTTAAAAGGGGTTTACTTAAACTAAGGAAAAGTTCATTAAAGAATGATGGTTTGGTGTTTTCTTAGGTTGGGTGTTTGTGAAACTTTAACTTAATCATATGTGGTTGTTTGATTGGATTCATGAATGGGGTAAATATTAAGATTAAGGGACAGGTTATGTGGGGTAAAGCAGTAAAGGAAAAGGCATTTAAGGATTAAAGTTAAGTGAATTTGTATTTGGAGTTAAGTTTGGGTATCCTTGATTTCAGGGTTTGAAGGTAAATCTGATAATGACAGACTATTCAATGCCTGGGATGACTGGTTATGAACTACTCAAAAAGATTAAGGTTTGTAATTCATTCCCTTTCTTCCTATTGTTTCATGACCTTAAACTTTTTATGGTCTTGAATGATGAGATTGATCCTTTCTCTCTGAGCAGGGTTCTTCAGCACTGAGGGAAATTCCTGTTGTGATAATGTCATCTGAAAACATTTTAGCTCGCATTGATAGGTATATATTCCTTCCATCGGATTAAAACTGTATACATTTTTTATAGTTTGACTGAGTCAAATCAAGAAGACAAGAAGGACAATAGGCCGTTTTCATTCAATTGTCCAACCAACTTAGTTGGGATTGGTCTTCATACATTCTTGATTGTTGGGCAGAGGTCCGTGAAGGGCCAAATCCAACACCACTAGGTAGCTAGAGGATTGTTAGGTGGAGGTTAAATGTATACACCCCACCAGGTGTATACATTCTTTTGTTTTTTTTTTTTTTTTAAGCGATGGACTTAAACCAAGAAGGCCAATAAGCGGTTTTTATTGGAAGCTTAACTTGTAATCTTGATGTTACCCAGTCAATTGTTTGACCAACTTGGTTGGGGTTGGCCTTCGTACATTCTTGATTGTTGGGCGGAGGCCCGTGAAGGATCAAGTCCGGCACCACCAGGTGGTTAGGGGATTATTAGGTGGAGGTTAAAGGACCAAGTCTAGCATGTTGCCTCTCAAAAATATGATGGTATATAAGGAAATAAAATTGTGCTTTTGCTATGGGCTCTAGTTTTTAGTGTAGTTGTAAGGATTTAAATCCAAACAAGTGGTAAGGGGTTCGAGTTCTAGCGATAGTAAAAATTGTGCAGTTAAGGCACCTTGGGAGGGGGAATTGTTGTTGGTTTTCATAAGATTGCCATTAGAAGATGCAAAATTTATGAAAGTTTGGATCTGTTTCATCATCTAATTGCATATCTTTCACAACAAACAGATGTCTGGAGGAAGGGGCCGAGGAGTTTCTGATGAAGCCTGTTAAGCTGTCCGATGTAAAGCGTCTCAAAGATTTCGTCCTGAGAGGAGACGGGGAGAGCAAAGAGGGAGCAACAACCCGGAAGAGGAAACCGACAGACGACTCCTTCATAATGCCGCCATTATCTCTTTCCTTGGCGTCGTCTTCGCCCTCCATCCATCCCGAGACCACTACGCCTCTGTCCCCGAGGTGTTCATCAGTTCCTCTCTCAAAACATCCTAGACTGCACCAAGACACTGAGCCACTGGTGGATCCCTGAACCATCTGGTGTGCTCAGATCAGATCATCTCAGTTTTTGAATTTTGATTTTCGGTACCCTTTGCTGCCCTTCTTTGTCTGCTTAATTCAATCACACTTGTACATGCTTACCTGTAGAAACACCATTCCACCACACCCTTTGGGGAGAAGGAAAAAATTATATATATCTTAACCTGATTTTGTTCACATTTTGTTTTGTGGATTCTAACACCACAGCAGCTCTGTAAATCTGTAATTTATGGTGTTTGGTTGGATGATTTGATGTTTTGATTCCCACAACAATGTTGTGTCCATTCATATTTTTGTTGATTTGGACATGACATACAGTACTAACATATGACTCATGGCATGGCATAAGTATTGCAGAAAAAAGTGATAAGAATGTTGCAAGTTATCCCATCACAAAAGTCCTTTTTTCTTATGGAATAAGGGCCAAATTAGTAGCGAAAATCTGTCGGTCAACCAAAAGCGTGCAATTAAGTCACTGAACAGCGCAAATACATCCGGATTCACTTGCTAGCCGGTTTCCATCGATCCTTTCCGGTTGTCTGCTGACTAGGATGATTAGCTGGCATTTATTTCTTTTATTTTCCTTTGTTGGCTCAGAGTCCACCCCACCGTGTATTTTCCTTTCCGGATCCTCAAGCTCTTCTCCACCGACTCAAGTTTGGTGTACTGAAACGAATCAATCCGTTCGATTAGTCCGAGGTAGTCGAGGATCTTATCCACCCATTCCCACCGGCTGTTCAGCGAGTGGTTCAAATCTGTTTCATCGTCTCCGACCACGTTGCCGGAGAAATCCGATTCTTCAAAAAATTCACAATTTTTATGATCCTCTCATTCCTCTTCACGCACAAAACCCACCCCTCAATCCGTTTCTTCACATACTTAACCATCCCGCGAGGATCTATCCGATGATATTCTTCATCTGGCCGCCGTTTTAACCGTTCATACTCCTCCTGCGCATCCTTCATTATCACCACGGTCCACTCAGAGAACACGAGCTTCACCATAGCTATGGAGTCCAAGAGAACGGCGCTGCCCAGGAGGATATAAGTGACAGAAATGTCCCGGACATGAATGCTGCGCTTGTTGCGGTAGAAAAAGATGATGGTGACGACTATAAGTGAGATGTGGAGCACCAAATCGAACGAACGGATTTGGATCTATGTAATGAATACATTTTGCAGGCGGACAAATCCACCATTGTTGACGAAGTTGTGAACTATATCAAAACCCTACAACAGACTTTGCAGAAGCTGCAGACTTGAAAGCTGGAAATCCTCCATGGATTTAACTCCAATAATCCATCGCCGTCCATTTTCGGCTCACAGAAACTCAACGCCGAGCTCACTACGAGGGAGGCGTTCCTGGCCGATCATCATCAAGGATCTTCCGGCGCTCTGGCTTCCTTCATCGAATCATCATCCGCCGGCGTTCCAGACCTGGACTTCTCCGAATGTTATCCTGAATGTTTACGGAGACGATGCTCAAATCAGTCTTTGCTGTCCTAAGAAGCCATGCCTCCTTCAGGCCATTTGCTTCGTTCTGGAGAAGCATAAAATCGAGGTGGTTTATGCTCAGGTTTCATCGAATCATCACCGCACTAGTATTTCCGGCGCTCACTTTTGTGATTTTGCTTCGTCGTCGTCGTTGTTTTGA

>g58574

ATGGGCATGGCAGCAGTAGAGCCACAGTTCCATGTTCTAGCTGTTGATGACAGCCTCATAGATAGAAAGCTCATTGAGAGACTTTTCAAGACCTCTTCTTGTCAAGGTATTTCATTCATTAAACACAATATAATGATGGGACAAAGGGATTTTTATGAACTTGTTGAAGATGGATTTTCTTGATTACAGAGTGTACTGATTCTGGGTTTTTCCTTTGTGTTCATTGTAGTAACTACAGTGGATTCTGGTAGCAAGGCCTTACAATTTCTGGGATTGAATGAAGATGACCAGAAAAATCCAATTCAACCTTCTGTTTCCCCCAACAATCATCAGGTCACTTTTTTTTTTGTTCTGTATTGATTTCAGGGATTGAAGATTCTTTCTGCTTTGATTGCTATCTGCAATATGTCTGAAAATTTTGTTTGTTGTTTCTGGTATTTTCAGGAAGTGCAGGTGAATCTGATCATTACAGACTATTGCATGCCTGGGATGACAGGCTATGATTTGCTCAAGAAAATTAAGGTATGCTTTAGCTTTCAATTTTACCCATATCCACATATACTGCATAAATATGATGCTGAAATAGCATTTGCAATTGTCTTTGTTCATTATTTTCTTTAGTGATCTTCCCCAATTGTTAGGTGCTCTAGAAATCATGTTTTGTTTGGTACAAGGTTTGCTAGGAATGGTTTTGGCAAATGAAACATGTCTGTTCTTTATCTCTCCCACAGGAATCTTCATCTCTGCGAAACATACCTGTGGTCATTATGTCATCTGAGAATGTTCCTTCAAGAATCAGCAGGTAAGAGCTTCTTGAGAAGAATCTGAAATTCTCCTTTTTCTTGGAGATGATGATGATTGGTAGCATTAAAGGGTGCTTATAAAACCTAAACAGATGCTTAGAAGAAGGGGCAGAAGATTTTTTTCTCAAGCCAGTGAGGCTATCAGATGTGAATAAGCTCAGACCCCATATGGTGAAAAACAGGAAGGCAGGAGAACAAGAAATTCAAGAATCATCATCATCAGAGGAGTCATCTGCAGAATCTGGTATGACAGATGTTCAATCACAGGCGAATCGAACGATAATCGTTGTAATAAGAGGAAGGCCTTGGATGAAGGTTTTACACAAGAGAACAAGAACAAGATGCAATAGCCTCACTGCTTTCTCTGATCTATGA

>g53645

ATGAGAACGGAGAAAATCGCCGCCGTTGATGGCTGTTCTTCCACCTTTGTCGGCGGTGGCCGGGAGTTGCATGTCCTTGCCGTCGACGATAGCTACGTGGATAGGAAGGTCATTGAGAAGATGCTCAAGATTTCTTGCTGTAAAGGTTCGAACTTTTTCCTTTTATTTATTTATTTATTATTTATTATTTTTATTTTTATTTTTATTTTTGAAATTCTGAGCTAGAATTTTTCATTGGTTTTTGGAATGTGGGTATTGCAGTGACAGTAGTAGATAGTGGTAGCAGGGCTCTGCAGTATTTGGGCTTGGAGGGAGAGGAGAGCTCTGTTGCAACTGATGTAAGAATTTTTAAAGTATTTAATTATTTTCTCTAAAAATGTAGATGAAAATTTTCTGTGATATATTGCTATTATTTTTTTTTTGGTTATTTATTTCAATTCTTTGGTGAGAAAAAGAAATTAATTATTAAAGGCTTGGTGTTTTGTATGATTGGGTGTTCCTTTGAATTTTGTTGGTTAATTGGTTTGAGGGGTTTTGTTGGCTTCAGGGTCTGAAGGTGAATCTGATAATGACAGACTATTCAATGCCTGGGATGACAGGATATGAGCTCCTTAAAAAGATCAAGGTTTGTCAAACAACCTTTTTCCAATACTAGAATGAAGTGGCCATAGTGGGAACCTGTGAATTGAATTCCAGATTTTAAATGCAGATCTTGAAACTGTCATAATTGTAAATAATAATACATTAGTTCTTCTTGATCAGGGTTCGTCAGCATTGAGGCAAATCCCCGTTGTGGTAATGTCATCCGAAAAGATTTTAGCCCGCATTGATAGGTTTGCTTCCTTTTTGACCATCCAGCTCTTGATTATGTCTTGTGTATTTGATATATATAGCTAGGTTTCACCCGCATTCTTGATTCTATGTCATGCGTAGAAGATTCTTTGCTTCTCAGGGCAGAGGAGATAGAATCCCAGTTGTAAATACATTGTTAATGTTTATCTGGCTAACTTCAATGAATAAGCACATGAGTTATTTTGTGCAAGAATATCAACAAGTTGTAGGATTTGGCCTGTAATGTGAACATTTGAAGTTTTACGGTTGAAACTAAAATCTGCAGGGTAGATGGCCTGCATAATTTTTATTTAGAAGTGTATATAGACTAACTTAATTTTTATCTAAGGGCCTGAAAATTTGTGAGGATCGTGTTTTAGTGATGTTGGAACAGTAGTTATAACACAGAGGCTTTTAACACAGTTTGAGAAAAAGCCTAACCCCCCTTTGTCAAATTTTTGGTAAAAACATATTCTTTAGGGCCATTTGTATCTGAATTTCTTTGAGATCTTGAAATCTTTGAATGCCTAACTACTAAACCTAATAAATCCTAAATAAACCGAACTTTACCAAGTTGGTTTGCCCCGAGTTTCATGTTATGAAATCAAGTAGTTTTTGCCCTATTATTTTGAACTTTTACAACTTTGCAATTCTCGTATCATGATTATGCTCTGTCTACTTGATTTCACTCGACGGTGTATGCTTAATGGCCATCTGATTTTTGTTCATATGTTTCTTAACCGATAGTTGTCTGGAGGAAGGGGCTAAAGAGTTTCTCATGAAGCCCGTTAAGCTGTCTGACGTGAAGCGTGTGGTAGATTTCATACTGAGAGGCGAGGAGGATGGCAACGAGACAGAATCAACGACGGGTTCCTTTTCATCATCAGCTCCTGATAACACGCCATTATCTCCAGAGTCGTATCTCGCTAACATTGCCAGAATCTCTCTCGACGCATCCAACAGAACATAG

>g24894

ATGGGCATGGCAGCTGCAGATCCACAGTTTCATGTTTTGGCTGTTGATGATAGCCTCCTGGATAGGAAGCTCATTGAGAGGCTCTTCAGAACCTCCTCTTGTCAAGGTCTGATCTTTTCTTGGTTTGATACATTGATGTGACTGTGGGATTTGATTCCTGGGGTTTGATTGTGGGGTTTTTGCATTGATACTGATTCTGGTTTTTTTTTTTTGGGTGTTTTGCATTGCAGTTACTGCAGTTGATTCTGGTAGTAAGGCCTTAGAATTTCTTGGATTACTAGAACATGGCCAGGATTGCCAAACCCAACCTTCTGTTTTACCCAACCACAATCAGGTAAAAATTTCTCTCATTTTCCCTTTTGTCTTGGTTTTAGGAATTCCAGAATTTCTGTAAAATTTCCTCAATGTTTTCTGGGATTTGCAGGAAGTGGAGGTCAATCTTATCATCACAGACTACTGCATGCCTGGGATGACAGGCTATGATTTGCTCAAGAAAATCAAGGTAGGTATATTGGTTGCAATATGCTTTGTGTTTTGTAATTCCTGGAGGGCCAATCTTATCCACTGATTTTATATTTTGCTTTGTTGATTGAGAAATAGAGAGATAGAGATTCTATAAATTTCTTTCCTTTCATGGAAAAAAAAAGCCCATGACAAAAGATTTCCCATCCATACCCAAGCTGTACTGTAGTCTGTAGCCTCCTCCAAAGTGGGTCCTTTTCTCAGCTTTCAATATTTGAGATCAGTGGGAATCTGGCAAAAAATCTTGCCCACCTATTTTTGCATAATTAGATGTTGAAAGGGCATGTGCTATTGTCACCTCTACCACTCACCTCCCTATTGTCTTTATTCTTTACTTTTTTTTATTTTGTGCATTGTAATGGCTACATTGAATTTTTTTTTTTTTTTCTGGCCTGGGAATGATAGGGTTGTGGTGAATAAATATGGGTGTTTGCTTTTTCCCTGACAGGAATCTTCATCTCTGAGAAACATACCAGTTGTCATCATGTCATCTGAGAATGTTCCTTCTAGAATCAATAGGTATGAATATGAACAATGCTAGATCAGACCAACTTGAACCAAGTTCGGTTGATTACGACTGATAACCATAAAGTTACAAGTTTGACTTTCATGGTCTAAAGGCCGACTTTCTTGGTTTGAGTTGGTCAGCTATGGGCAACCAAGACTGGTCAGATTGTTGATTCGGTAACCATAAGGTTACAAAATCGGCTTTTAGTAGCCTAAAGGCCCGTCTTTTTGGTTTGAACTGATTAGCTATGGGAAACTCCAACCAAGTTGGCCAGATTGTTGATTTGGTAACAATAAGGTTACAAGTTCTTCGACTTTCAGTGGCCTAAAGGCCTACATTCTTTGTTTGAGCTGGTAAGCTATGGGCAACCTAGGCTGGTTTATGGTCTGAATTGTGTTTTGTTGAGGATGAAATGATGAATTAATGGGGATCTTGAAAAATTTACAGATGCTTAGAGGAAGGGGCAGAAGAGTTTTTCCTGAAGCCAGTGAGGTTGTCAGATGTGGATAAGCTTAAACCTCATATGATGAAAACCAAGGGCAATAAACAGCAGAAAGCAGGGAGTGATGACACCCAAGAACACAAAGAAACATCATCTGAAGAGTCATCATCAGTTGAGTCTGGTGTAACAGATGTTCAATCACAACTGCCACAACTACCATTAGAACAGCCACAATCAGAGACACAACTGCACCAACCGCCACCCGATAATAACAATAATTGTAACAACAAGAGGAAGGCCATGGAAGAAGGCCTTTCACCAGATAGATCAAGAACTAGATACAATGGTCTCACCAGCCTCTAA

>g24753

ATGGAAGGCCTTCCTCTGGAGGGCTATCAAGGATGTTCTCCCTACTACTACAAATCTCATTTTAAAGAGGGTAGAGGTTCTTCCAACATGTCCAATGTGTGGTCAATGTCATGAGAACACTATGCACTCCCTTGTCTTGTGTGATTTCTCAAAATTAGTTTGGCATGAGACTTCACTTCCAATTTCATCTGTGGGGATACTTTTGTAAATTGGTTTTCTAGTTTATTATCTCTGCTCACGGATGATCAGCTTATATATGCAGCAGCAACACTATATTATATTTGGTCGGCTCGCAACAAGGCGGTGTGGGAGCACTTGCTACCGCGGCCAACCTGGAGAGCGGCTTCGGCGGCGGTGCGAGCCTGGCAGCACGTCCATCATCCGCAGACAGCAAACGCAGCACCTCCACAGCTTGGTTACCACCCGACACCCATTGCAGAACCGTCCAGCCTCATCTGCCATTTCGACGCTGGTTTTCAACCGGCAACGAAAAGGGCAACGGTTGGTGCAATCGTCCGATCTAGCACTGGCGGCTTTGTGGCAGCGTTCAATGGTCTTTTTCGGAGCTGTATGTCGCCTCTAATGGCGGAATCCCTTGCATGTAAGGAGGTACTTTCCTGGATGAAGGATAAAGGCATTGATCGTGTCGTTCTTCACACTGATAATTCGATCCTTCAACGATGGCTTACGGCGGAGAATAATGAGTTCTTTTCTTATATTGCTTTTTCTATTGATGCCTCTAGGGCTATTATGTCGTCATTTTCGCATTGTTCCATTAGGTGGGTGCCAAGAACGGCTAATTTAGGTGCCCATGCTCTTGCCTCTTTGGCGTTTTCTCAGTCTGATTTTCTGTATTGGGATTCTATCCCTCCTGACATTATTTCGTCTTTGATTTAATATACCACTATTGGTTGTTTTCAAAAAAAACAACGGAAGTAAGAAAGAATTGGGAAAAACAAAAGAAAAACAATAATAATAATAATAATAATAATAAACAACGAAAGTAAGAAAGAATTGGGAAAACAAAAGAAAAACAAGAATGGGAATAAGAGATGCTAGGCGCACACCACACCCAGGAATCGATCCTTGGACCTCGCGCACTGTTCACCCAAAACACTGTTTATCCGTGTATCCATTCCTCCATTTGGAGAGTAGATGTTTTCCTTTCTCCAAAACTGTATAAGAATTGGAAGAATGGAGATGAGATTGAGATCGGAAAACAAAATTTAGTAAACAAGTTTTCCATTTTCCATTTTTCCACTTCTCTCATTCTCTATTTTTTTGAAAAATCTTCCATTTTTCTAAGTTAGTAAACGACCCCTGGGTCTCTTCCCCTTTCTCCCCCACCCGAAGCAAGACATGGTAAGCGCACATGATTTGGCACCACCATAAATCGACCGCACTGCTCACATCACTTGTATCTCTTGGCAGGATTTTGGTGGACCAATCCAACCCTACTACTATATTTTTATTCTTTTTTCAACTAAATAATCAATATAATGCATACAACTTATATACAAATATACTAAATACTTCGTACGTGTATCTAATTACATGACATGATACCATACATGTTATATTTATGTGAATTAATATTGTTTATAGTTATAGGAGGTTTGTGAATTTATCACTTTAATTTAGGGGTATTTTTGTCTTTTTGATTGTTTTTTTTTTCTTTTTCACTAAACCCGACTTTCTCCAAATTTCTCTAAACTCTTACCGTAATTGTTTCTTATTGAAAATTGGAATAATTCAAAGGCGTTTCCATCTTTTCAAAAGAAATCACTAAAAGTGACAAACCAACAATATTCCATAATAAAATAATGTTATAAGCGCTTATGAGGCTTAGTGTATTGGTACGTCGTCTGAATTGATGCCGATCACCATTGAAGGCGGTAGTGGGAGGCCATGGAGGCTTAGGATGATGGTGGGTGTGGATTGGATCGCTTGTGCAAGCATGAATGCTAAGATCTTCTTGACAGACGCTGATTTACCTCTCATGACTTTTAGACATGGTAAGCCTCTACATCACTCAAATTATATTTTAACATTGAGTTATGTATAATAAACACACGTAAACAATTATATAAATAGTTATTAAAAATTTATATTTACTTTGTGATGTATTGATGTCATGGTCCAAAAAATTATAAAGTTAGTCCATTAAATTTCAGTCCTTTTTAAACAAATATTTGAATTACCACACCATGCAACAATAGAGAAGCTCCAACTAACAGTACTACTACATAACATAAAAAACAGCTTATATATATATATGCTTTAACCTTGTCTCAAATTCTTCCCCAAGCCTTCCCCCTGGTTGTCAAGACAAAAACCCCATGAGTAAACTTGCTAGTTTTCTTCATAGCATATATACATGAAAGCCCAACCTTGAAAGCAATATTCTATACTATACTTCTTAGCAGCCCCATGTTCTTTCTGGATATGCAATCTTTTAGTTAACTTTGTATAAACATATTCTTTTATTGTCTCTTTCCCATTGGTTTGCCTTGTAATTCAAAACCAGGATTGGAAAACCGGGGTGGAGATATCTTGGCAAGAAATCTGGTCAGATCTTGCTTTGTGGGTTTGTGGATTTTGGTGGGTCTTGGTTTTCAATACAAAGTTTCTTCTCCATTTACCCTCTTCTTCTCATCTCATTATAAAGTAGGGGGGAAAAAGAAATAGAAAAAAAGGGGAAATTAAGAACTCATATATATATATATATACCCCAACAAAGAAGGAAGGCACCTTTTTTTCCATCTTTCCCTTTTTTGGTTTCCTGTTCTTGGTTATTCTAGCTTTTAAAGCTCTGTTTGGGTTCTGGGGATTGAAGAAGAATTTGGAGAAATGGGCATGGCAGCTGCAGATCCACAGTTTCATGTTTTGGCTGTTGATGATAGCCTCCTGGATAGGAAGCTCATTGAGAGGCTCTTCAGAACCTCCTCTTGTCAAGGTCTGATCTTTTCTTGGTTAGATACATTGATGTGACTGTGGGATTTGATTCCTGGGCTTTGATTGTGGGGTTTTTGCATTGATACTGATTCTGTTTTTTTTTTTTTTTTGGGTGTTTTACATTGCAGTTACTGCAGTTGATTCTGGTAGTAAGGCCTTAGAATTTCTTGGATTACTAGAACATGGCCAGGATTGCCCAACCCAACCTTATGTTTTACCCAACCACAATCAGGTAAAAATTTCTCTCTTTTTCCCTTTTGTCTTGGTTTTAGGAATTCCAGAATTTCTGTAAAATTTCCTCAATGTTTTCTGGGATTTGCAGGAAGTGGAGGTCAATCTTATCATCACAGACTACTGCATGCCTGGGATGACAGGCTATGATTTGCTCAAGAAAATCAAGGTAGGTATATTGGTTGCAATATGCTTTGTGTTTTGTAATTCCTGGAGGGCCAATCTTATCCACTGATTTTATATTTTGCTTTGTTGATTGAGAAATAGAGAGATAGAGATTCTATAAATTTCTTTCCTTTCATGGAAAAAAAAAAGCCCATGACAAAAGATTTCCCATCCATACCCAAGCTGTACTGTAGTCTGTAGCCTCCTCCAAAGTGGGTCCTTTTCTCAGCTTTCAATTTTTGAGATCAGTGGGAATCTGGCAAAAAATCTTGCCCACCTATTTTTGCATAATTAGATGTTGAAAGGGCATGTGCTACTGTCACCTCTACCACTCACCTCCCTATTGTCTTTATTCTTTACCTTTTTTATTTTGTGCATTGTAATGGCTACATTGAATTTTTTTTTTCTGGCCTGGGAATGATATGGTTGTGGTGAATAAATATGGGTGTTTGCTTTTTCCCTGACAGGAATCTTCATCTCTGAGAAACATACCAGTTGTCATCATGTCATCTGAGAATGTTCCTTCAAGAATCAATAGGTATGAATATGAACAATGTTAGATCAGACCAACTTGAACCAAGTTGGTCGGATTGTTGACTTGATAACCATAAAGTTACAAGTTCTGACTTTCATGGTCTAAAGGCCGACCTTCTTGGTTTGAGCTGGTCAGCTATGCGCAACTAAGGCTGGTCATTGTTGATTCGGTAACCATAATAAGGTTACAAGATCGGCTTTCAGTAGTCTAAAGGCCCGTCTTCTTGGTTTGGGCTGATTAGCTATGGGAAACTCCAACCAAGTTGGCCAGATTGTTGATTTGGTAACAATAAGGTTACAAGTTATTCGACTTTCAGTGGCCTAAAGGCCTACCTTCTTTGGTTGGGCTGATCAGCTATGGGCAACTTAGGCTGGTTTACATGGTCTGAATTGTGTTTTGTTGAGGATGAATTAATGGGGATCTTGACAAATTTACAGATGCTTAGAGGAAGGGGCAGAAGAGTTTTTCCTGAAGCCAGTGAGGTTGTCAGATGTGGATAAGCTTAAACCTCATATGATGAAAACCAAGGGCAAGAAAGCAGGGAGTGATGACACCCAAGAACACAAAGAAACATCATCTGAAGAGTCATCATCAGTTGAGTCTGGTGTAACAGATGTTCAATCACAACTGCCACAACTACCATTAGAACAGCCACAATCAGAGACACAACAGCACCAACCGCCACCAGATAATAACAATAATTGTAACAACAAGAGGAAGGCCATGGAAGAAGGCCTTTCACCAGATAGATCAAGAACTAGATACAATGGTCTCACCAGCCTCTAA

>g55375

ATGGGCATGGCAGCAGTAGAGCCACAGTTCCATGTTCTAGCTGTTGATGACAGCCTCATAGATAGAAAGCTCATTGAGAGACTTTTCAAGACCTCTTCTTGTCAAGGTATTTCATTCATTAAACACAATATAATGATGGGACAAAGGGATTTTTATGAACTTGTTGAAGATGGATTTTCTTGATTACAGAGTGTACTGATTCTGGGTTTTTCCTTTGTGTTCATTGTAGTAACTACAGTGGATTCTGGTAGCAAGGCCTTACAATTTCTGGGATTGAATGAAGATGACCAGAAAAATCCAATTCAACCTTCTGTTTCCCCCAACAATCATCAGGTCACTTTTTTTTTTGTTCTGTATTGATTTCAGGGATTGAAGATTCTTTCTGCTTTGATTGCTATCTGCAATATGTCTGAAAATTTTGTTTGTTGTTTCTGGTATTTTCAGGAAGTGCAGGTGAATCTGATCATTACAGACTATTGCATGCCTGGGATGACAGGCTATGATTTGCTCAAGAAAATTAAGGTATGCTTTAGCTTTCAATTTTACCCATATCCACATATACTGCATAAATATGATGCTGAAATAGCATTTGCAATTGTCTTTGTTCATTATTTTCTTTAGTGATCTTCCCCAATTGTTAGGTGCTCTAGAAATCATGTTTTGTTTGGTACAAGGTTTGCTAGGAATGGTTTTGGCAAATGAAACATGTCTGTTCTTTATCTCTCCCACAGGAATCTTCATCTCTGCGAAACATACCTGTGGTCATTATGTCATCTGAGAATGTTCCTTCAAGAATCAGCAGGTAAGAGCTTCTTGAGAAGAATCTGAAATTCTCCTTTTTCTTGGAGATGATGATGATTGGTAGCATTAAAGGGTGCTTATAAAACCTAAACAGATGCTTAGAAGAAGGGGCAGAAGATTTTTTTCTCAAGCCAGTGAGGCTATCAGATGTGAATAAGCTCAGACCCCATATGGTGAAAAACAGGAAGGCAGGAGAACAAGAAATTCAAGAATCATCATCATCAGAGGAGTCATCTGCAGAATCTGGTATGACAGATGTTCAATCACAGGCGAATCGAACGATAATCGTTGTAATAAGAGGAAGGCCTTGGATGAAGGTTTTACACAAGAGAACAAGAACAAGATGCAATAGCCTCACTGCTTTCTCTGATCTATGA

>g42973

ATGGGCATGGCAGCTGCAGAGTCACAGTTTCATGTTTTGGCTGTTGATGATAGCCTCATAGATAGGAAGCTCATAGAGGCTCTTCAGAACCTCCTCTTGTCAAGGTAGGTGCTTTCTTGGTTAAACTAACACATTGATTTTGATTAGATCAAGGATTTGATTCCTGATGGTGATGGGGTTTTTTTTGTTTGTTTGGTTCTGATTTAAGAGATTTCTTTGTAATTTTGCATTTTTTTGTTCTTCTTTGCAGTTACTACAGTGGATTCTGGTAGCAAGGCTTTAGAATTTCTGGGTTTACATGAACATGATGATGAGAACAACACAAACCATCATCCATCTGTTCTATCCAACCATCCCCAACCCCAGGTAATTTTACATATCCCTTTGATTTTCAGGGATATGATTTCTGGGTTTTCTTTCCCCTTTAATTAATTGATTGTTCTCTGGATTTTTTCTGAAATGTTTCTTTGGGATTTGGCAGGAAGTAGAAGTGAACCTTGTAATTACAGACTACTGCATGCCTGGGATGACAGGCTATGATCTACTCAAGAAAATTAAGGTATGTTTTGTCTTGTTCCTCTTCTTGCCAATATTCCTTTTTCTTACTTTCCTGCCACCTCTGCATCACTTCCCTCTCCAGGAAAGAAATATAATTTACTCCATCACAAAAACTTCCAAACCATACTAAGTTCTAAACTTACCCAGTCCTTTCATTGGCATTCAATTTTGAGATCAATTACAATCTGTACCCAAAATCTTGCCCACCTGCCCCAAGTACTTGGCTTTGTTGTCTTTATTTATTATGACCCCTTTCTTTAGTAAGCTTCCCTAAATTTTAGGCATTGCAGCCTAGACTTTTACCCAGAAATAGTTTTGGTTTTATTAATTCATACTTTGTTGTCCCTCTGACAGGAATCTTCATATCTGAGAAACATACCTGTAGTCATTATGTCATCTGAGAATGTTCCTTCAAGAATCAGTAGGTAAGAACAATGAGCAATGAAAAATGAACAATTTCTGGTCTGGGTTTATGTGATTCTTGAAGAATTTTTAGCAGGATATAGGATTAATGGGAATTTGTAAAATTTGCAGATGTTTAGAAGAAGGGGCAGAGGAATTCTTCCTCAAGCCTGTGAGGTTATCAGATGTAAATAAGCTTAGACCTCATATGATGAAAACCAAATGCAAGAAGCCGAAAACGATCCCTGGACAGCCAAGAACCATCACCAGAACACCCTCTAATCCAACAAGAATGTGCTGTGGAAGATGTGAAATTGCAGCCCCAAAACCCGCAACCACAGGCACAGACAGAAGAGCAAAAACCAGTGATGAATAGCGATGAAGACTCGAGGAAGGCCATCGAAGAAAGCCTTTCACCGGGAAGAACAAGAACGAGACAAGAACCACAAGGGCGGGCCGAAGAGCAACCGAGTAATGAAAACAAGAGGAAGGCCATGGAAGAAAGGGTTTCACCAGATAGAACAAGACCAAGATACAACAATAATGGTCTCACTCACTGCTGTCTCTAA

>g42966

ATGCTTAATCCAGATTGGAAAATTCTGGGAGATCTTTGGTTGAGATCTGGTCAGACCTTGCCCTTTCTGTGTGTTGTTGTTGTGGATTTTATTTTGGTGGGTCTTCTTACTTCTTTCCACACAAACTTCCTTCATATACACACACCCACAAAACACAACCCCACAAAGGAGCCACCTTTTGGCCCCCCATATTTTCTGTTTTCAAGATTGAGGAATTGGAGCAATGGGCATGGCAGCTGCAGAGTCACAGTTTCATGTTTTGGCTGTTGATGATAGCCTCATAGATAGGAAGCTCATAGAGAGGCTCTTCAGAACCTCCTCTTGTCAAGGTAGGTGCTTTCTTGGTTAAACTAACACATTGATTTTGATTAGATCAAGGATTTGATTCCTGATGGTGATGGGGTTTTTTTGTTTGTTTGGTTCTGATTTCAGAGATTTCTTTGTAATTTTGCATTTTTTTTGTTCTTCTTTGCAGTTACTACAGTGGATTCTGGTAGCAAGGCTTTAGAATTTCTGGGTTTACATGAACATGATGATGAGAACAACACAAACCATCATCCATCTGTTCTATCCAACCATCCCCAACCCCAGGTAATTTTACACATCCCTTTGATTTTCAGGGATATGATTTCTGGGGTTTTCTTTTTTTTCCCCTTTAATTAATTGATTGTTCTCTGGATTTTCTGAAAATTTTCTTTGTTATTTCCTGGGATTTGGCAGGAAGTAGAAGTGAACCTTGTAATTACAGACTACTGCATGCCTGGGATGACAGGCTATGATCTACTCAAGAAAATTAAGGTATGTTTTGTCTTGTTCCTCTTCTTGCCAATATTCCTTTTTCTTACTTTCCTGCCACCTCTGCATCACTTCCCTCTCCAGGAAAGAAATATAATTTACTCCATCACAAAAACTTCCAAACCAAACTAAGTTCTAAAGTTACCCAGTCCTTTCATTGGCATTCAATTTTGAGATCAATTACAATCTGTACCCAAAATCTTGCCCACCTGCCCCAAGTACTTGGCTTTGTTGTCTTTATTTATTAGGACCCCTTTCTTTAGTAAGCTTCCCTAAATTTTAGGCATTGCAGCCTAGACTTTTACCCAGAAATAGTTTTGGTGTTAATAATTCATATACTTTGTTGTCCTCTGACAGGAATCTTCATATCTGAGAAACATACCTGTAGTCATTATGTCATCTGAGAATGTTCCTTCAAGAATCAGTAGGTAAGAACAATGAGCAATGAAAAATGAACAATTTCTGGTCTGGGTTTATGTGATTCTTGAAGAATTTTTAGCAGGATATAGGATTAATGGCAAGTTTGTAAAATTTGCAGATGTTTAGAAGAAGGAGCAGAGGAATTCTTCCTCAAGCCTGTGAGGTTATCAGATGTAAATAAGCTTAGACCTCATATGATGAAAACCAAATGCAAGAAGCCCGAAATCGATCCCCGGGACAGCCAAGAACCATCCCCAGAACACCCTCTAATCCAACAAGAATGTGCTGTAGAAGATGTGAAATTGCAGCCCCAAAACCCGCCCCCACCGCAGATAGAAGAGCAACAACCAGTGATGAATAGCGATGAAGACTCGAGGAAGGCCATCGAAGAAAGCCTTTCACCGGGAAGAACAAGAACGAGACAAGAACCACAAGGGCGGGGGGACGAGCCACAGCCACTGGTGAATAATAGTAATGAAAACAAGAGGAAGGCCATGGAAGAAAGGGTTTCACCAGATAGAACAAGACCAAGATACAACAATAATGGTCTCACTCACTGCTGTCTCTAA

>g30557

ATGGCGACTTCTTCCTGCTCAGATCTTGGCAAGTTTCATGTCTTGGCTGTTGATGATAGCATCATTGATCGAAAGCTCATCGAGAGGCTCCTCAGAACTTGTTCTTATCAAGGTATTGTGCTTTACTGTATTTGGTTTGTTAGATCTAAATATAATCTAGTGATACTGAATAATAGTCTAAGAGAAACCTAATTTCGTAATAGCGGAAGCAAAAACAGTATTGATCTTCAGTAGATCGTCTATATAATCTCATCAATTCAGATTTATATTAAGCACGAAGTCTGATTGAATAACAAATTCTAGGGTTTCTGATGTTTTTTTTGGGAATTTGCAGTGACGGTGGTGGATTCTGGGGTGAAGGCGATGGAGTTTCTGGGCGGGAGGATTGAGGAAGTGAATTTGATCATAACGGATTACAGTATGCCGGGGATGACGGGGTACGAGCTTCTGAGGAAGGTGAAGGGGTGTTCTTCATTGAAAGATATTCCGGTGGTGATCATGTCGTCGGAGGATGTTCCGGCGAGGATCGACCGGTGTTTGGAGGAAGGGGCGGAGGAGTTTTTCCTGAAGCCGGTGCGGCAGGCGGATGTGAACCGCCTGAGTTCCCATTTGCTGAGACCTAAATCTCCAGAACCTTCGCCCTGCCGGAAAAGGAAGGCGGCGCCGGCGGAGGCGCAACCAACCAGGCCGATAAGAAGAAGAAGATTGATCTGA

>g20447

ATGGCAACTTCTTCAAGGAATGGGGGAGATGAATCACCTCATGTACTAGCTGTTGATGACAACCTCGTCGATCGCAAACTCGTTGAAAAGCTGCTCAAGAATTCATCCTGCAGAGGTAAGAACCGAGTGGTAGATTGTGAGTAAAAACACAGGATCAAGCCCTGACAGTTGTAGTGTGGAGTTACCATTGTGACTGGCTCCTTGTGAGCACCAGACACTGGTCCTCCCACCTAATGGCCTAAGTCACTGTGATTTACTCTTCCACTATCTTGTTGGGGTGTGGGGGGCCTGAGAGCGAGGGAATTTTGTTGCCCTTTTGAGGGCAAGAGTTGTGTGTGTTGTTTACAGTTAATCATAGAGTAGAATTTGTATTGACAGTTTGATTTGCATTGCATCAGTGACTACTGCAGAGAATGGGTTGAGGGCTTTGGAGTATTTGGGGTTGGGAGATGAACAACACAACACTTCAAATGACAATGTCAGCAGACAGGCAAATCTGTATATATTTTGAGAAGAAAACACACTGTTTTCATTGCGATTTTCGTTAACTTTGTAGAGTTCCGATTTCTGCAGGGATCAAAGGTGAATATGATCATCACAGATTACTGCATGCCAGAAATGACAGGCTATGAGCTGCTCAAGAAAATCAAGGTAGTTTAACTATCAACTGTTGATTTAATATTGTTGTTTTTCTGATCATAAACCTTAACATCTAAACATCCAATGATGTTGTTGAAACCAGGAATCTTCCAACATGAAGGACATCCCTGTTGTGATAATGTCATCTGAGAACATTCCAACTCGGATCAACCAGTGAGTACTGTAAACTTCAAAGTTGAGTTATAATTTGGTCATTTTGATGCCATGAAATGTTTCTAACTGGGAAAGTATGCAGATGCTTGGAGGAAGGAGCCCAGATGTTCATGCTAAAGCCACTCAAACACGCGGATGTGAAGCGATTAAGAGGCGAATTGATGCAGTGCAGAGGCTAA

>g5095

ATGGCGTTGCTTTCATCATCACCATCTTCTTGTTCGTCATCAATGGCGGAGGCGGAAGACGAGATTCCGCATGTGTTAGCTGTGGATGACAGCTCAGTAGACCGCAAGCTCATCGAAAGGCTACTCGCTACCTCTTCCTGCAAAGGTTATGTCTCTCTCTCTCCCTTTCTCTCTACAGCTTCAAGCTATGGAGTCTTGTGATTTCTTGAATTGGAATGTTGTTATGGTGTTTGTTATCGTAGTGACCACAGCGGAGAATGGGCAGAGGGCTCTGGAATTTTTGGGGCTCGGAGAGGGGCATACCAGTAACAGGGTAAGATCACTGGCCAAATTAATTAGGTTATTGTCTTAATAATCACAAACTTACAATTGGGGCTCCTAAATCCTAACTAGACTCTTAATCATAAATTAAGTCTAGCTATTTGGGGTATTGGGTGTAAATTAGAATTGTGAATTTGATTATACCTGTCTGCATTTTGCAGCAGTCAAAGGTGAATTTGATCATAACAGACTACTGCATGCCGGGGATGACAGGCTATGACCTGCTTAAGAGAGTCAAGGTAACCAACCAATAACTCCATTTGCAGAAACCTTAAACTCCATAGCTACTAATAACTAACTGCGACGATGCTTGGTTGTTGAACCGACAGGGATCATCTGACCTGAAAGAGATACCGGTAGTGATAGTGTCATCCGAGAATGTTCCAACAAGAATCAAGAAGTAAACACTCCATCCTCTGCTTTAGCATTATCCCAGGATTCATTGTAAACACATGTTGACTGTTCTTAATTAAACTGTAACAGGTGCCTGGAAGGAGGGGCTCAAGAATTCATGATAAAGCCGTTGAAACAATCAGATGTTAAGAAATTAAGATGCCATATGGCTAAGTTTAAGCAGCCCTGCAGTGGACGGTTATGCATTGGAAGATAA

>g6428

ATGGCGTTGCTTTCATCATCTTCTTGTTCGTCATCAATTGCAGGCCGAATACGAGATGCCGCATGTGTTAGCTGTGGATGAAAGCTCAGTGGACCGCAAGCTGATCGAATGGCTACTCGCTAGCTCTTCCGTAAAGTGACCACAGCGAAGAATGGGCAGAGGGCACTGGAATTTTGGGGCTGGGAGAGGGCATCATGCCATGACAAAACTTCCATAAAGATAAAGAATGAATATTCCAGTGAAAGGGAGAAAGAACATGTAGAAACTTCCATAAATTTTGATCCTCTGTACCAACTTTGTTCTACTGAGATTTTCAAAGTTTACAATTTTTCCTTAATAAAAAAATCAAGGAATCGAGTCCGAAGCTATGGAGTGATCGTCAATAGCGTTTACAAGCTTGAACCAGTATGCCCTCCTCTCCTCTTTAACACAGAAATTGAAGAGAAAGCCCTACAGGAAAAAGGAACAAAATTATCACCCTTATCTAGCGAAGAACGACACGAGATCTTGA

>g35948

ATGCCGGAAGGATGCGAGAGGATGGACCAGCTGACCTTCGACGACTTGGTCCCCAAATACTTCAAAGCCACCGCCATGCTTGTGGATGACAGCTCAGTAAACTGCAAGCTCATCGAAAGGCTATTCGCTAGCTCTTCCTGCAAAGTGACCACAACGGAGAATGGGCAGAGGGCACCGAATTTTTGGGAGTGGACGGTTAAGCAACCCTGCAGTGGAAATTAAGATGTCATATGGCTAAGTTTAAGCAGCCTTGCAGTGGATGGTGAATCTGTATTGTATCAGGTTATACATTGGAAGATAAAGAATGAATATTCCAGTGAGAGGGAGAAAGAACATGTAGAAAAGAGTTCTAGAAGGTAGAGCTATGGAGTATGGACTGGAGAACATCGCCGGAAGTCTGCCGCCCGCTGCCGTCTTGGCGGAGAACTTCGACGATTCACGCCGATCAACGGTGAATCGAACTGGCCGCTTCGGCAGAAGCGCAGACGCCGCAGGCGCCGCTGACCAACATTGTCGCCGCTACTGGTGA

>g41064

ATGCTTGTGGATGATAGCTCAATAGACCGCAAGCTCATCGAAAGGCTACTCGTTAGCTTGGTTTCATCTCCGACTGAGGCTAAGGAGCGGAGGCTTCGAAGGAAACGACCGGCGGTTGAGTTTCGCCGCGACCAATTTCTTCGCTGTCATCCTCACCGCAAAAATCGGAGACAACAAAGCTTTAACCGGCATCCGTTTGGCGACAAAGCACGACTAACGCAATGATAATGGCCCTGCAGGAGAAGATCTGAGAGAGAACTTTGATATTCCATTTGGATGGCACGTTGGAAGCAGTAACCGGCGACCTCCCCTGCTCTTAGCGAGCGGTGTTGCAGCGGTAGCTGGTTGCGGAGATGATGTTTCCGTGGAAGCCGCCAGCGAGTGTTCTCTTTCCAGTGAGCAGCTATGGCGTCGGCGAGGTTTGGGGATCGGCGGAGGAATCTCTCCTCTTACTCCCTGCTTTTCTCCGCAAAGTATCAAAGGCGTGACCGGCACGTGGTCCATCTCCGACCATCTCTCTTCTCCCGCGTAA

>g8841

ATGGCAACCTTGGATGATGGTTCGGAGTTTTTCCACGTTCTTGCCGTGGACGACAGCGTGGTGGACCGGAAACTCATTGAGAGGCTGCTGAAGACATCCTCGGAGAACGTAAAAGTGACGGTGGTGGATTCCGGGAGCAAGGCGTTAGGGCTGTTGAGCGAGGTGGAAGTGAATCTGATCATCACGGATTACAGCATGCCGGGGATGACGGGGTACGATCTGCTGAGGAAGATCAAAGGCTGCGCCGCGTTCAAGGATATTCCGGTCGTGATCATGTCGTCCGAGGATGTCCCGTCCAGGATCACCAGGTGTTTGGCGGAAGGCGCGGAAGAGTTCTTCCTTAAACCGGTTCGACAGTCCGACGTCAACCGCCTCAAACCGCATCTGTTAAACGGCTGCAAAGGAAAGGCGGCGTTTTCCCCAGATTCCGCGGCGGCGGTTTGCGGGATCGTTTGA

>g30555

ATGGCGGCAGCGGTGGTGGCGGCTACTCATGCATCAGAATCAAGATTTCACGTTCTGGCCGTCGATGATAACCTCGTGGATAGAAAGTTGATCGAAAGGCTTCTCACAACTTGTTCTTACCAAGGTACCAGTTTTCTTGGCTGTTGTTGAATTTTCTAAGAATTATGAATATTAATCCACTGCTATGCTATAACTCAGTTAGTTTTTGAGTAATAAATTATGAATTACGATATAAAATTGCGTCTCAACAATTGCATTGTGGAAATTTCACTTTTGACTGTTATGGTGACGAGATACATCTCTTGTAAATCTTATATTGCTAATGATCATAGCAAAAGAACCACAAAGAAATAAATTAATTTAGATTATTGTCTATAGTTGATCAGCTCAAATAGCTAATCAAAGTGGTCAATATGCTACTTCTGGAGAGTCAAATTTGTAATTTTATAATTTGAGTTGTTTTGAATTACGCGTATTTATCTCTCCACAATTCTGTTTGCCCCGGAATTAGGGTGATTGATGGACCAGGAATTTTCTCTTTTGTAAGCAAGATTTTAATTTAATTTGCTGTATTTGTTTGGACAGTGACTGTAGTGGATTCTGGGAACAAGGCGCTGGAGATTCTTGGGCTGTTGGAAGATTCCGTGACGGCTCTGAACTCCGACCACCACGTACGGCCGACCGGAGTAATAATTAACCACCGTTAAACATAAACTATGTTTGTTAACTAACGTTGTCCATGGACTAATTGCAGGAAGTGGAAGTGGATTTGATCATCACAGACTACTGGATGCCGGGGATGACGGGGTACGATCTGCTGAGAAAGGTGAAGGAATGCCGGAGAGACATTCCGGTGGTGATAATGTCGTCGGAGAACGAAGCGTCGAGGATCAACATGTGCTTGGAAGAAGGCGCGCAGGAGTTTCTGGTGAAGCCCGTCCGCCAATCTGACGTCACCAATCTGATCAAACCGCGCCCGTTTGTGAAAGGCGGCGACGATAACGGCGTTGTTTCGCCGGTGTATTGTTCAGGCGTCGATGATAATCGACATGTAACGGCGACGGAAACTGTTATCTCCCCGGCGGACCGATAA

>g3932

ATGTCTGCAGTCATGCATGTGCTGGCGGTTGATGACAGTACGGTTGACCGTACCATTGTTGAGCAATTGTTCAAGGCCGCTTCTTGCAAAGGTATTTCATTCAATTCATTTTTATGGGTAACCAAAGAAATCTGTCGTCAGTATCTTAGTCAGTGCACGGATAAATTAAGTATTCTAAGAATCAAATTCATGACTTTTAATACACTCATTTACACTTTTAATGCCATTCAATTCATTAAAATGCTTAGTTTCTAATATCGTAAAGAAAATGTTTTTTTTTTCCTCCTAAATTCTATGGTTACTTTGGGAAAATTTCGAGGGGTGAGCAACATACTAAATATAGAATGCTTATATTCTTGATTGAGAAACAAATTAAATCACACTTGACATGATGCTTTCTAGTGCCATGGATTAAAATGCCGAGTTCTATGTAAAATTGTACCTTGTGCTTTAACAGAAGTACATGTAGAGAGAGTACAATTTGTAAAGCGTAAAGTACAACTTTACGAAAAATACGATATTACTTATTGTTTTTTTATCATGAAATTTGGGTTCAGTAACAACCGCAGAGAATGGATTGAGAGCCTTGGAGTTCCTGGGGTTATTAGCAGGAGATGACCAAAACAACTCTCCAAACACCAACGTTAGTACTAAATTTCATTCCATATATGCACAAACCTTAATTACTTTGAATATTTATATTTTTGAATAATATTATCTTTCTGCAGGTCCCAAAGCTGAATTTGATAATAACAGATTACTCCATGCCTGAAATGAATGGCTATGAGTTTCTTAAGAAAGTCAAGGTCAAAATGTTTAATTTTAATTAATTTGAATATCACTGAAATTATATTTGCTAGATAAGTCGTATCGTGTAAGACTGTTTTCCATGAGACGTATAATACTTTATAGCTATATATAGTCACATTTATAAGGCTATCCAAATATGACACTAATGTGAAAAACTAGTTTGGTATTTCATGATATAATCTTAATTGTTGGTGAGTATGTCTTATATATATGGAATAAGAGTGGAGCGGATTGGGTCAACCTATAGTATATGAATATATTTTTATACATGTATATGTTTAGGATTGTATTTAAATATGACGTTTGTAACCCTAGATATTTTATCGTTACTAATCTCTAACATTAATTAATATGTTGGATGTTGGATTTTACAGGGCTCAGCCATGTTTAAGGATGTTCCAGTTGTGGTAATGTCATCCGAAGACACTCCAAGTCAGATCAACCAGTAAGTACAATTTATTACCAGTTCCTTAATTGGGGTTAATCAGACAACCTTAAATTCTGCTAACAGTAATTAATTATTGTTTAATTATTTGATAAATCAATAAAAATTGATTGACAGATGCATGGAGGCAGGAGCTAGTGTGTTCATCCTGAAGCCTCTCAAGCAAGCTGATGTGAACCAACTGAAATCTCAGTTAATGCAAGCCTGA

>g3934

ATGTCTGCAGTCATGCATGTGCTGGCGGTTGATGACAGTCTGGTTGACCGTACCATTGTTGAGAAATTGTTCAAGTCCGCTTCTTGCAAAGGTATTTCATTCAATTCATTTTTATGGCTAACAAATAAAATCTGTCGTCACTATCTTAGTGTGTGCACCTGATAAATTAAGTATTCTTAAAAATCAAACTCATGACCTTTTGGTATGAGAATTATGTTCTACTCACTCATTTACACTTTTAATGCCATTCAATTCATTAAAATGCTTAGTTTTCAATATCGTAAAGAAAATGTTTTTTTTTCCCTCCTAAATTCTATCGTTACTTTCAAAAATTTCGAGGGGTGAGCTGCTTCATACTAAATATAGAATGCTTATATTCTTAATTGAGAGACAAATTAAATCACACTTGACATGATGTTTTCTACTGCCATGGGTTAAAATGCCGTGTTCTATGTAAAATTGTACCTTGTACTTTAACAGAAGTACATATAGAAAGAGTACAATTTGTAAAGCGTAAAGTACAACATTACGAAAAACATGATATTACTTATTGGTTTTTTATCATGAAATTTGGGTTCAGTAACAACTGCAGAGAATGGATTGAGAGCCTTGGAGTACCTGGGGTTATTAGCAGGAGATGACCAAAACAACTCTCCTAACACCAACGTTAGTACTAAATTTCATTCCATATATATATGCACAAACCTTAATTACTTCGAAAATTTATATTTTGAATAACAATATTATCTTTCTGCAGGTCCCAAAGGTGAATTTGATAATAACAGATTACTCCATGCCTGAAATGAATGGCTATGAGTTTCTTAAGAAAGTCAAGGTCAGAATCTTAACTTTAATTAATTTGAATATCACTGAAATTATATATGCTAGAGTAAGTATTATTATGTACATATAATACTTTATAGTTATATATAGTCATATTTATAAGGTGGTTGCAATTGTTCCTTTCATCCTTGACTCCAAAAACTATCCAAATATGACACTAACGTAAAAGATTGTAGTTTGATATTTCATGATATATATATATATATATATATAAATATATAAAAGGATATTAAGAGGGGAGTGGATTGGATCAACCCATAGTACGTATATGGATATTTTTGTACACGTATATCTTTAGGATTGTATTTAAATATGGCGTTTGTAACCCTACATGTTTTATCATAGAATAGCGTAGACACATTACTGAACCACGTAAATCTTGTCTTTTTTTTTACTTTCTGTCATATATTATTAATATTGTTTGTTGATCTTGTTAATCTCAACATTAATTACAGGGCTCAGCCATGTTTAAGGATGTTCCAGTTGTGGTAATGTCATCCGAAAACACTCCAAGTCAGATCAACCAGTAAGTACAATTTATTACCAGTTCCTTAATTGGGGTTAATCAGACAACATTAAATTCTGCTAACAGTAATTAATTATTGTTTAATTATTTGATAAATCAATAAAAATTGATTGACAGATGCATGGAGGCAGGAGCTAAGATGTACATCCTGAAACCTCTCAAGCAAGCTGATGTGGACCAACTGAAATCTCAGTTAATGCAATCCTGA

>g3935

ATGGCTTGCCCTTCATCAATGGCAATGGGGGAAATTGGGGAAGATGAAGTCATCCATGTGTTGGCTGCTGATGACGATCCAGTTAACCTTATCATCCTTGAGAAATTGCTCAACTCCTCTTCTTGCAAAGGTTTGTGCATTATGACTAATATAAGCTAGCTAGCTTAGTTGTTTTAATTTGGTCATTGAATTATATTTTCGATTTTTGGAAATATATATAGTCATTTGTTGTACTGTGTCATTCTAATATGTTGTGTTTTCGGTTCTTGATATTGTAACTTTAATGATTTGTGGTAATGTCTCTATCTCTACCTCTATACCATACCTCTATACCCCACTTCCACCCCATATATAGCCTTATATGTATATATATGTATATATAGTATATGTTTTAGATATAAATATATTAAAACAGATATTATAAACAATGGGTTAACTATCTATTTTGTTTTAATACTTCATCCCGCTAGAAGAAATTTCTGGGATCCGTCCTGATTTCATAAATTAGGCTTTGTATTGATATCACAACTAATTTTCAACAATCCATGAAATTTGGGTTCAGTAACAACTGCAGAAAATGGATTGAGAGCCTTGGAGTACCTGGGGTTATTAGCAGGAGATGACCAACAAAACTCTCCAAACACCAACGTTAGTACTAAATTTCATTCCATATATTCACAAACCTTAATTACTTCGAATATTTATATTTTGAATATTATTATCTTTCTGCAGGTCCCAAAGGTGAATTTGATAATAACAGATTACTCCATGCCTGAAATGAATGGCTATGAGCTTCTTAAGAAAGTCAAGGTCAGAATCTTTAATTTTAATTAATTTGAATATCACTGAAATTATATATGCTAGAGTAAGTATTATTATGTAAAACTGTTTTTGAGATATAATACTTTATAGTTATATATAGTCATATTTATAAGGTGGTTGCAATTGTTCCTTTCATCCTTGACTCCAAAAACTATCCAAATATGACAATAACGTAAAAGACTGTAGTTTGATATTTCATGAGATAATCTTAATTGTTGGTGATGAGTATGTCTCTTTTTTTTTTTTTAAATATTATATTGTAATATGTTGAGGTTGGATATATATTGGGCGCAGGAATCAGCAATGCTTAAGGATGTCCCAGTTGTGGTAATGTCATCAGAGAATGTTCCAAGTATTATCAACCAGTAAGAACAATAATATTAATATTGTTGGATTAGTTGGTCAACTGTTAAACTACCTTAAATTCAGTTAAAAGTAATTAATAATTAATTTGATGAATGAACAGATGCTTGGAAGAAGGAGCTCTTATGTTCATGCCAAAGCCTCTCAATCAATCTGATGTGAAGCACTTAATATCTCAGTTGCGTTAATGCATGCACTTCTTCATTTTTTTTAATCGATTTGTTTTTTGTTTTTCTTTTATGATTATTGTGTGTATGCTTATTGGATATTCAAATATATTACAATGTATGATCTATTTATATATATATATAACAAGTGTAATGGTTAATCACTCAATCGTTCAAAAATCAACTAATTAACTTTCAATCACTTTGGATAGTTGAAACTCTTTTGATGAACTCCAAGCAACACGTAAACTATAGTTTTTTTTTAATCTAAATTGAAACATATGTTTTATCTCAACTAAAAATCTAAGCTGGTAGTTGAATTGCATATTTATATTTATATATTATATATGCTCAACATGGGTTGTCAACCGTCACCAAGATATTATATATATTGGCCTTAAATACAATTTTTTCTATTCTATTCTATTAATGACAAAAATAAATATTATAGTTTAATAACAAAGTGAACGAATATAATTCGTCAATAATATAATGCATGGTTCTTCGTGATCATTCCATATTCAATAATACTATTTTTTCTAAAGAAAGCTACATGGTATATTAACAGATGAAAAATCAAATTAAACGAGTAAAAAAAAAAAAAATTTATATAACTTAGACTTCTCGATCATACTATATCTAGACCCCATGAATATACTTCAAAATCTATAAAGAAAATAAGTCCTAAATTACTTAAGTGATTCTAAATTTTCATCACTTATATAAAGTCTATAAACATTATTCTTTCATATATATCCACTTCAAAGTTCCAAGAGAGTATGAGATGCACACTCCCGTGACACGTGTAAGGCACAGATTGGATAGCCACATTAGAGAACCAAAATAAGCGCCTGAGAGTCAAGGAAGCTCGGAGGCACAACAATGGCGTCCCTTTTGGAGACTCCATTTTCTTCACCTCACTCACTCTTCAACTCTCACTCCTCACTCTTCACACCCACAATCCCACAACCCCAATTCACTGCAATGCTTCCCTGTCAAGCCCTTTCACCTCCCTATTCTCCGCTCTTCCTCCACCACCGGCGGAGCAGTCGACCCTTCGCCGCCGCCGCCGCTGTCGAACCAGGACGAGATTTCGCTTGATGGGTCAAGTAAAGATCGGCGCAAGGTGGTCAAGTTTGCCTGGGAGAAGCTGGTTCGATGGTCTCGCTCATGGCGGTCCAAGGCCAAGACTGACGTTCTTGAACGCACTAACAAGGTCTATACACTTTCTGTGTTAATGTTAATGCTAGCAAGTAGTAATTTGGGAATTTTAAGGTAGGATTGGTTTTAAAGCTCTCATTTTTATGTTTTACTTCTGTTTACTTGAATGGGTTTCTGCTGCTCTTTATATGAAATTAATTAGAATTTCAGTTAAATAATCCATTGGTATCTCCATATCTGTATGACTGTATATGAAGTTCTGAGAATGGTAATGCTAGAACTCAGGTAGGGCATCTCCCTTTAGCTGTAGTATGCAAAGGTGTAAAATGCCTATGTTTTTTTTTGAGCTAAGAGGGAAACCACAGGTGTGCACGGGGTAAACCCGCCTTGTGACCCTACAAGGCACAATGTTGCCCATAGCTGACCACACAAGGAGATAAAACAAGGCTAGGTTGTCCATAGCTGAGCTCAAACCAAGAAAGTTATTAGGCCGCTCCTGCTAGGAGTTGAACTTGTAACGTTATGGTTATCAAGTCAAAAGTCTGGTTAAACTTCGGCTAGGATTGCCCCAATTATCTGAGGGTTTGCATTGGGTAAGTCCCAACTTATGACTCTAGCCAACAAAGAGCCACAATGAGGTATACAACTTAGCTCAAACTAAAAAGGCCAATAGGCTGCTTTGCTTGCTGTCGAACTTGTAACTTTGTGGTTACCAAGTCAACAACTTGGGCAATTTGGCTGGGGTTGCCCCAATAAAATGTTTAATTAGTGCTATTGTTGCTTCACTTTGGGTAAATGTTTAATTTTGTATTGCTTAGAACTTTAGGTGGTGGTTCTTGGAGGTGGATCTTTTGGAACAGCTATGGCTGCCCATGTTGCAAATAGAAAGGCTGAATTGGAAGTTAATATGCTCGTACGAGATCATCGAGTTTGTGAATCCATTAATGAGAATCACTGCAATAGGTGAGTTTGTGGTTTGTGTGTCATCCTTTTTCCACATTCCACTTTCATATTCCATAGCTTCATCTTTATGGTCGTTTTTGTTCGATTTTCTTTTGCTCCAGTAAGTATTTCCCAGAGCACAAGCTACCAGAAAACATAATTGCAACAACTGATGCCAAGGCTGCTCTGCTGGGTGCAGATTTTTGTTTCCATGCTGTACCTGTTCAGGTAATTCAGATCATATTTTCTTTGGTACTTCATTTCCCTAGATTTTATGAAATTATGTTGGATAACCTCATAACCATAGAGTTCAAAGACATGGGTATGTGCAAAAAGAGAAATTTAAACTAGGTTTTTTCCGGCTTTTACTGGCTTGGTTATGATAGTTTAAATTGATGTTAACATTTCGGTTTCTTCTTCTTTGGCTTGTTTATGCTGTCTAGTTTGACCTCATGACCAAAGAATGCCACTTATTTGCAGATTATATCTTTTTTTTTTTTCAGTTCAGCTCGGTATTCCTTGAGGATATTGCAATGCATGTTGATCCGAGCTTGCCATTTATATCTCTCAGCAAGGGTTTAGAGCTCAATACATTAAGAACAATGTCTCAGATAATCCCCCGAGCTCTAAGAAGTCCTCGGCAGCCCTATGTCGTTCTATCTGGACCTTCTTTCGCACTCGAGTTGATGAACACGTTACCAACAGGTTATTTATTTAATCTCCATAGCTTATACTTCATGCACTGGATTAAGTTTGTTAGTAATCAAAATCATTCCCGATAACTATGCAGCAATGGTGGTAGCGTCTAAAGACAAAAAAGTTGCAAATGCAGTTCAGCAACTTCTGGCCTCTAGAAACCTGAGAATCAATACATCAAGGTTGTTCTTCTAATCCTTATCCTATACTGATCTTTTTTTTTGACCTAGCAGCAATTAGGAGATCTTTTGCACCATAATTTCTTAGTGACAGCTAAAATAAATGTCACCTTGCAAGAGAACTAGAGATTCGAGGTTCCAGCTGTTAAAATATGTATGCACTTGAGTGCTTTCTTGTAACACCGAAGCTTCAGTGATGTTACAGGGATTGAGATTGCAGGTGCACTGAAGAATGTACTAGCAATAGCAGCTGGTATTGTGGAAGGATTGAATCTCGGGAACAACTCCATGGCTGCTCTTGTAGCACAAGGGTGCTCTGAAATTCGATGGTTGGCAACAAAGGTTGGTTGAATCTTTCTATGATTCTTTACTGTTTAAGGAAAGTTTTAGTTCATGGGTTAACATTATCTTGGTGACATCAAAACCATGAACAATGATTATGAGAAAGTGATCATCTTCTCTGTTGTTTTAGTTTACTTACCAAGTCATGCTGTTTTTGTTCCACTAAAGATGGGTGCAAAGTCAACTACATTAACGGGGCTTTCAGGGACCGGGGACATCATGCTCACGTGTTTTGTGAATCTTTCAAGAAACAGAACTGTTGGGGTTCGTCTTGGATCAGGAGAAAAGCTTGAAGACATACTCGGTTCTATGAACCAGGTTAGTGAAAATTCTCTCGAGGTTTCTTGTGTGAAACAGTCCCGGGGTTTCTTTCTTTCACCCGAAATGCTTTGTGTTTTTTAAGGTGGCTGAAGGTATAACAACAGCAGGAGCTGTGATTGCGCTAGCGCAAAAATACAAGGTCAAAATGCCGGTTTTGACAGCAGTTGCCCGGATTATTGACAATGAACTCACTCCAACTAAAGCTGTTTTTGAATTGATGAATCTCCCTCAGGTTGAAGAAGTTTAG

>g34926

ATGGACATGGGTTCGAAGAATGTTGGGAAGGGGCTGAGGGCGCTTGTGGTCGAAGACGACCCCGCTACGCAGATGGTCCACAAGATGCTGCTGAAGAAATACGGAGTGGAAGCTCAGGTGGCCAAGAATGGCGAGGAGGCGGTGGAGCTCCACCGCTCCGGCGCCCGCTTCGACCTCCTGCTCATGGACAAGGACATGCCTGTCAAGGATGGCGTCAATGTGAGCCCATTATTCTTCCCTTTCCCTCTTTACACTTATATACATATATATATCTATTCAGAAAATGTTAAGATGACAATTTGCACGGCCCATGCACGATTCTGTATATGTTTTTATGTGTATTCGAGAAACCTTAAATGCACAATTTTGTATCTACCTTATATGTATATTTAAGAAACAGTAAGGTGACTATTTAATTTACAATGAATACTATTTTTATATATTCGAGAAACTTAAGGTGACAATTTATACCCGGTACTATTCCATATTTGATATAATCCGTGCAAATTGGCACGATATTGATCGAACCCTAAAAAGGAAAGCATGTTAATTTGTTGTGTTTTGGCATGGTTGAATTAGGCAACTCGGGAGCTGCGGGAGCTGGGCGTGAAGAGCATGATAGTTGGTGTAACTTCGCACGGGCCAGGTGCGGTGAGGGATGAGTTTATGGCGGCGGGGCTAGACGAGTGTCTGATGAAGCCGCTAGGTGCAGATATGGTTTTGCGCCTGATTAACCAGCTGGTAGCCTAAAAATGAGTTCCAAATAGGGAAAATATGTATGCTCCGCTTGGTTGTGTAATTGTACTCTAAGTCTCCAATAATAATAATAAATGAAATTATGAGGTTGAAAAATATATTATATTATTGTCTAAGTCTGCACCTTGATGTGAAACATCGCAGCGGCCACCAAGCCTATGTGCTGGGTGTCTCAACCACCTCTTTCCCTCATCATCCCAAAAGCATATAACAATTAATGTATTAATTCTAATTATCAAAAGATGTATTTATTTGGATTATTAAAAATTCATTAAGCTCAAATGGTGATGCATTTGTATATATGACAATTAATCAACTAACACCGTACCATACATGGTCTGATACAATTTGTCTAAATTGATATATTAACAAAATTATTGAATAGTTAAAATGCTAAAATCATTTTAAATTTTAATATTTACAATGGATAAAAAAACTTAGGTTGTTTATCCGTTCAAACAAAAAAGGTCAATATATCGCTCAAGTTGTGTTGGGTAAATCCCGCATTATGACCATAGCCGGTCATAGGCTTAAACTAAAAAGGTTAATAGTTTGGGTAAATCTCACCTTGTGACAATAGCCCATAAATGACAAGGTAAACGAGACTAAGTTGTTTATACTGACTCAAATCAATAAGAAGACTCAAATTGAGGGAGGAACTTGTAACCTAAAAGTTACCGGTCAAACCAACCGTATGATTGATTAGCTGGGCCCCCTCTCTTCCCTTACTTGGTACACAAGATGCTGCTGAAGAAATACGGATTGGATGCTCAGGTGGCCAAGAACGGCGATAAGGCGATGGTGCTCCACCACTTCGACCTCCTGCTCATGGAGAAGGAAATGCCTGTCAAGGATGGCGTCAATGTGAGCCCATTCTTCTTCCCTTTCCCTCTTTACTCTTATATATTCATTCCAAAAATGTTAAGATGACAATTTGCACAGTTGGTGCACGATTCTGAATCTGTCTTTTATGTGTATTAAAAAAACCTTAAATGCACAACTTTGTATCTACCTTATATGTATATTCAAGAAACGTAAGGCGACTATTTAATTTACAATGAATACTATTTTTTATATATGTATTCTAAAAACTTAAGGTGACAATTTGTACCTTGTATCGGTTTTGTATTTGATATAATTCGTGCAAATTGGCACGATATTGATCGAACCCTGAAATGGAAAGATGTCACTACAAAAAAACGCTAAAATAACGACGGATTTTATGACGGATTCATTCCGCTAAATTAACCACTTTAATAGCTTTAATTAGATTTACTTATTTTCAAAAAATTAAAATTTAGCTTACCTGACAGCTTAACGGATTAGCAACGTAATTTATACGAAAACGCTTGTCGTTAAACCGTGAAGCTAATTTTAGCGCTAAATTGCCGCCGATAAAATTAAGAGGATTAGCTACGGTTTAGCGATGGATTTTTGGAGGGGCTGATTAAATAATTTTATTTGCTATGGATTAGCGACGGATATTTTTCCGTCGCTAAGGTTTAAAGCATCTATTTATTTTTTACAACGAAGCGACGAAATAGTGACTGAAAATGGTATCCGAGTTACTCAGCTTACCGGATGAGTATCCGCTGCTGAGTAATACGCCCCATTTTAAAGAATATAGCTTACTGATTCAAACTGCCGCTACATTTTATAGTCGGAGTTATTTTTTTTTATTTAGCTTAATTGATATGTAATCCGTCGCTAAGTTAAACCGCTTAATGTCTTAAGTATGTCATGTAGCGATTTGAGGTTCCGTCATATATATAGCAACTTCAAACCGCCGCTAAGTTAATGACGCTTTATTTAAGATTTTTTTTTTGAAAACCTAGCAACGGATTGTCTTATCCATCGCTAAACTTTGGAACAAAAGAGGCTTTGCAAACGCCTAAGAAAAACGAATTGCCATAACCCTGCAGCCGTTGGTTCGGTAATCTGGTGATTACCTGTAACTGAAGTGAGTAATGTTATATTACCTTGTTTGTTTCAAGTTATGAGATTACGGTAATGTTATATTACCTTAAAGTCGATGTGCAGAATGTTTCAAGGAATGTGATTGCTTTTTTTGGTAATCTAAGATACCTTGATTATTCCAACTTTGCCCATGTTAAGTAATCCTTATATATAATAATAATAATAATAATAATAACACAATAACATATATAAAATAAATATATGTTTATATAATCAAATATACATGTGTGTATATTTATATATTTATTTTTTAATATTAAGCATCAATACATTCATACATATAAATAAATAAAAATACACACAATAAAGGCACACAAATAAATAAATAAATAAATAAATAAATATATATATATATATATATATATATATATATATATATATATATATATATATATATAGGACATTACAGAATCGTTCCATATAACCTGTAATATAACCCTTAACCAAACAGTAATATTATATTCCACAATCTAAACCAAACACATCAGGTAATCTTACATTCCCAAAATCTCACATTATCTTCCTGAGGTAATCCTATTATCCAAGATTCCGTGAACCAAACGCTCCCTAAGGATTAGCCGCCCACTGCTTTCTCATAACCTCGCAAACCTGCGTCCTGCTCCTCCAACCGCCGCCCGCGAACTGCCGACCGACCACCGTAAACAATCCGCTGCTCACCGCCAGCCGCCGACCGCCGACTGCCGGAAACAAGGTCTGCTCCTCCGAAAACAACCGACCGCCCAATTCCCACTCTCCTCTCCACGCTCCGTCGCTGCACACCGCCCACCTACGACCACCGCTGCCAGTGCGCTGCCACAGTGA

>g34929

ATGGACATGGGTTCGATGAATGTTGGGAAGGGGCTGAGGGCGCTTGTGGTCGAAGACGACACCACTACGCAGATGGTCCACAAGATGCTGCTGAAGAAATACGGAGTGGAAGCTCAGGTGGCGAAGAATGGCGAGGAGGCGGTGGAGCTCCACCGCTCTGGCTCCTGCTTCGACCTCCTGCTCATGGATAAGTCAATGCCTGTCAAGGATGGCGTCAATGTGAGCCCATTATTCTTCCCTTTCCCTCTTTACACTTGTATACATATATATCTATTCAGAAAATGTTAACATGACAATTTGCACGGCCCATGCACGATTCTGTATATATGTCTTTTTATGTGTATTCGAGAAACTTTAAATGCACAATTTTGTATCTACCTTATATGTATATTTAAGAAACGGTAATGTGACTATTTAATTTACAATGAATACTATTTTTATATATTCGAGAAACTTAAGGTGACAATTTATACCTGATACTATTCCATATTTGATATAATCCGTGCAAATTGGCACGATATTGATCGAACCCTAAAAAGGAAAGCATGTTAATTTGTTGTGTTTTGGCATGGTTGAATTAGGGCAACTCGGGAGCTGCGGGAGCTGGGCGTGAAGAGCATGATAGTTGGTGTAACTTCGCACGGGCCAGGTGCGGTGAGGGATGAGTTTATGGCGGCGGGGCTGGACGAGTGCCTGATGAAGCCGCTAGGTGCAGATATGGTTTTGCGCCTGATTAACAAGTTGGTTGCCTAAAAATGAGTTCCAAATAGGGAAAATATGTATGCTCAGCTTGCTTGTGTAACTGTACTCCAAGTCTCCAATAATAATAATAAATGAAATTATGAGGTTGAAAAATATATTATATTATTGTCTAAGTCTGCACCTTGATGTGAAACATCGCAGCAGCCACCAAGCTTATGTGCTGGGTGTCTCAACCACCTCTTTCCCTCATCATCCCAAAAGCATATAACAATTAATGTATTAATTCTAATTATCAAAAGATGTATTTATTTGGATTATTAAAAATTCATTAAGCTCAAATGGTGATGCATTTGTATATATGACAATTAATCAACTAACACCGTACCATACATGGTCTGATACAATTTGTCTAAATTGATATATTAACAAAATTATTGAATAGTTAAAATGCTAAAATCATTTTAAATTTTAATATTTACAATGGATAAAACACCTAGGTTGTTTATCCGTTCAAACAAAAAAGGTCAATATATCGCTCAAGTTGTGTTGGGTAAATCTCGCATTATGACCATAGCCGGTCGGTCATAGGCTTAAACTAAAAAGGTTAATAGGTTGGGTAAATCTCACCTTGTGACTATAGCCCATAAATGACAAGGTAAAGGAGACTAAGTTGTCTATACTGACTCAAATCAATAAGAAGACTCAAATTCGAGAGGAACTTGTAACCTAAAAGTTACCGGACAAACCAACCGTCTGATTGATTAGCTGGGCCCCTCTTCCCTTATTTGGTACACAAGATGCTCTGAAGAAATACGGAATGGAAGCTCAGGTGGCCAAGAACGGCGAGAAGGCGATGGTGCTCCACCACTTCGACCTCCTGGTCATGGAGAAGGAAATGCCTATCAAGGATGGCGTCAATGTGAGCCCATTCTTCTTCCCTTTCCCTCTTTACTCTTATATATTTATTCCAAAAATGTTAAGATGACAATTTGCACGCTAGGTGCACGATTCTGTTTCTGTCTTTTATGTGTATTAAAAAAACCTTAAATGCACAACTTTGTATCTACCTTATATGTATATTCAAGAAACGGTAAGGTGACTATTTAATTTACAATGAATACTATTTTTTATATATGTATTCTAAAAACTTAAGGTGACAATTTGTACCTGATACTGTTTTGTATTTGATATAATCCGTGCAAATTGGCACGCTATTCATCGAACCTTGAAAAGGAAAGCATGTTATGTTGTTGTGTTTTGGCATGCTTGAATTAGGCAACTTGGGAGTTGAGGGAAATGGGCTTAAAAAGCATGATAGTTGGCGTAACTTCGCACGGGCCCGGTGAGGTGAGGGATGAGTTTATGGTGGCGAGGCTGGATGAGTGCCTAATGAAGCCGTTGGGTTCAGATGTTGTTTTGCGCCTGATTAACCAACTTGGTTGCCTAAAAATGAGTTATTATGAAGAGTATTTTCCTATTTTACTATGCCTTAGTGCTTAGGTGCAAATTGGGAAAATCCTAGTGTATGTTCAGCTTGTTTGTGTAACTCCAAGTCTCCAATAATAATAACAAATGAAATTATGAGGTTGAAAAATATATTATATTATTGTCTAAGTCTGCACCTCGATGGGAAACATGGCAGTGGCCACCAAGCGTATGTGTTGCGCGTCTCAACCACCTCTTTCCCTAATCACCAAAAAAATATATAACAATTAATGTATTAATTCTAATTATCAAAAGATGTATTTATTTGGATTATTAAAAATTCTTTAACCTCAAATGGTGATGCATTTGTATATATGACAATTAATCAACTAACACCCTACATGGTCTGATACAATTTGTCTAAATTGAAATATTAAAAAAATTATTGAATAGTTAAAATGCTAAAATCATTTTAAATTTTAATATGTACAAGGGATAAAACAACTTAGGTTGTTTATCCGTTCAAACCAAAAAGTCAATAGATCGCTCAAGTTGTGTTGATTAAATCCCGCATTATTACTATAGCCGGTCATAGGCTTAAACTAAAACGATCAATAGGTTGCTCCCATTTTGTTGGGTAAATCACACCTTGTGACTATAGTGGATAAAGAACAAGGTAATTAAACGAGACTAAGTTGTCTATATATACTGGCTCAAATCAATAAGACGACTCAAATTGAGAGGGAAACTTGTAACCTAAAAGTTACTGGTCAAACCAATAGTCTGACTGATTGGCTGGTTTCCCCACTCTCTTTCCTTATTTGGTACACAAGATGCTGCTGAAGAAATACGAATTGGAAGCTCAGGTGGCCAAGAATGGCAAGGAGGCTGTGGTGCTCCACCGCTTCGACCTCCTACTCATGGACAAGGAAATGCCTGTCAAGGATAG

>g33149

ATGGGTTCAAAGAATGTTGGGAAGGGGTTGAGGGTGCTTGTCGTCGTAGACGACCCCGCTACCCAGATGGTCCATAAGATGCTACTAAAGAAGTGCGGACTGGAAGCTCAAATGGCCAAGAATGGCGAGGAGGCAGTGATGCTTCACCGCTTCGGCGCTCGCTTCAACCTCCTGCTCATGGACAAGGAAATGCCTGTCAAGGATGGCGTCAATGTGAGCCCATTCTTCCCTTTCCATCTTACTCTTATATATTTATTCGAAAAAGGTTAAAGTGACAATTTGCACGGTCGATGCACGATTCTATATCTGTCTTTTATGTGTATTTGAGAAACCTTAAACGCACAATTTTGTATCTGCTTTAAATTTATATTCGAGAAATGATAATGTGACTATTTAATTTACAATGAATACTATTTTTGTATATGTATTCCAGAAACGTTAAGGTGACAATTTACACCGGGTACTACTCAGTATCTGATCTAAATTGGCACCATATTGATGGAACCCTAAAACGGAAAGCATGTTATGTTGTTGTGTTTTGGCATGGTTGAAATAGGCAACTCGGGAGCTATGGGAGATGGGGTTGAGAAAGACGCACGGTCACGCAGCCGGTGATGAGTTTATTGTATGGACCGACGAGTGCTTAATGAAGCCGGGTCCGTAG

>g33158

ATGGGTTCAAAGAATGTTTGGAAGGGGCTGAGGGCGCTTGTAGTTGTAGACAACCCCGCTACCCAGATGGTACACAAGATGCTGCTGAAGAAGTACGTACTGGAAGCTCAGGTGGCAAAGAACGGCGAGGAGGCAGTAGTGCTCCACTGCTTTGGCGCCCGCTTCGACCTCCTGCTCATGGACAAGGAAATGCATGTGAAGGATAGCGTCAATGTGAGCCCATTCTTCCCTTTCCCTCTTACTCTTATATATCTATTCCAAAGAGGTTAAGGTGACAATTTGCACGGTCGATGCACGATTCTATATCTGTCTTTTATGCACGGTCGATGCACGATTCTATAGTTGGCGTAACTTCGCACGGGCTAGGTGCAGTCAGGGATGGGTTTATTGCGGCGGGACTGGACGAGTGCCTGATGAAGCTGCCGGGTCCAGAAGTGGTTTTGGGCCTGATTAACCAGCTGGTGGTTGCCTAA

>g33222

ATGGGTTCAAAGAATGTTTGGAAGGGGCTGAGGGCGCTTGTAGTTGTAGACAACCCCGCTACCCAGATGGTACACAAGATGCTGCTGAAGAAGTACGTACTGGAAGCTCAGGTGGCAAAGAACGGCGAGGAGGCAGTAGTGCTCCACTGCTTTGGCGCCCGCTTCGACCTCCTGCTCATGGACAAGGAAATGCATGTGAAGGATAGCGTCAATGTGAGCCCATTCTTCCCTTTCCCTCTTACTCTTATATATCTATTCCAAAGAGGTTAAGGTGACAATTTGCACGGTCGATGCACGATTCTATATCTGTCTTTTATGCACGGTCGATGCACGATTCTATAGTTGGCGTAACTTCGCACGGGCTAGGTGCAGTCAGGGATGGGTTTATTGCGGCGGGACTGGACGAGTGCCTGATGAAGCTGCCGGGTCCAGAAGTGGTTTTGGGCCTGATTAACCAGCTGGTGGTTGCCTAA

>g33229

ATGGGTTCAAAGAATGTTGGGAAGGGGTTGAGGGTGCTTGTCCGACCCCTGAGATGGTCCATAAGATGGAAGAAGTGCGGACTGGAAGCTCAAATGGCCAAGAACGGCGAGGAGGCAGTGGTGCTTCACCGCTTCGGCGCTCGCTTCAACCTCCTGCTCATGGACAAGGAAATGCCTGTCAAGGATGGCGTCAATGTGAGCCCATTCTTCCCTTTCCATCTTACTCTTATATATTTATTCGAAAAAGGTTAAGGTGACAATTTGCACCGATGCACGATTCTATATCTGTCTTTTATGTGTATTTGAGAAACCTTAAACGCACAATTTTGTATCTGCTTTAAATTTATATTCTAGAAATGATAATGTGACTATTTAATTTACAATGAATACTATTTTTGTATATGTATTCCAGAAACGTTAAGGTGACAATTTGCACCGGGTACTACTCTGTATTTGATCAAAGTTGGCACCATATTGATGGAACCCTAAAACGGAAAGCATGTTATGTTGTTGTGTTTTGGCATGGTTGAAATAGGCAACTCGGGAGCTATGGGAGATGGGGTTGGAGAGCACGGGTCACGTGCAGTCAGGGATGAGTTTATTGTGGCGGGACTGGACGAGTGCTTAATGAAGCTGCTGGGTCCAGAAGTGATTTTGCGCCTGATTAACCAACTCTAAAAATGTGTTATTATGAAGAGTATTTTCCTATTTTTTTATCTCCAAAATGGGAAAATACTAGTGGTCAACAGGTTGTGTAACTTTAAAAAAAATAATAACAATAATAAATGAAATGATGGGGTACGGTTGAAAAATATATTATATTATTATCTAATTTGATTTGAAGATTAGGTTTCTCTTAGTCTGTATCTGGATAGAAAACACAACAACAGCCACCAAAACCTATGTGTTGGGCGTCCCGACTACCTCTTTCCCTTATCATCAAAAAAGTATATAAAAATTAATAATGTATTAATTCTACTTATCAAAAAGATGTATTTATTTGGATTATTAAAAATACATTAACCTCAAATGGTGATGCATTTGTATATATGACAATTAATCAACTAACACCCTACATGGTTTGATACAATTTTTCTAAATTGAAATATTAACAAAATTATTGAATAGTTAAAATGCTAAAATCATTTAAAATTTTAATATGTACAAAATGATCAAAATTTTCTTCCTACCAAAGGGAGGCTGATTTTAATTCCTTCACAATCTCGGTATGAACGGTGGGGTTTATTTCTCTCGGACTGCATTTCATTTCTTTTTGTCGTCATCAGAGAGTGGTTCTGTTTCTCTCTTTCCTTTTTTTTTTTTGAGTGATGGAGAAACGTGTAGTCTTGATCTAAAAATACATACGGGGTAAATCTTGCCTTGTGAATCTAATTACAGGGGATAAAACAACCTAGGTTGTTACTGACTCAAACCAAAAAGATCAGTAGACCACTCCAGTTGTGTTGAGAAAATCCGGCATTACGACCATAGTCAGTCATAGGTTCAAACTAAAAAGGTCAATAGGTTGCGCCCATTTTGTTAGGTAAATTTAACTTTGTGACCATAACTGGTAAAGGGCAAGATAAACGAGGTTAAGTTGTACATACTGGCTCAAACCAAAAAGACGGTTCCGATTGAGAGTGGAACTTATGACATAACCTAAATGTTTAACAAACCATACCAATATGGTTGGTTGATTGGTTGCTAGGGGTGTGCAAACTTCGGTGAATATTCGGTTAACCGACCGAACCGACAAAGTTTGGTTAACCATTTTTAACCAAACTTTTTCGGTTCGATTAATGGTTAAATGTTTTTTAAAATTTTTGTTAATGGTTAATTCGGTTCGAAACTACGGTTAACCGAATTAACCGAAATTTTAAATATACAAATAAATAAATAAATAATCTAATATTTAAAATTCCTATAAATTTGTAATGTTGTTATGGTATTGTTTTGGTATATTTCTTATGGATTTATTATTACATGTATTATTTTAATTGAATCTACTATTATAGATATTATTTTTATGATTTAAATCTTTAGCTTAAAAAGTTTGGTGTATCGGTTAACGGCAAGGAGGCGGTGGAGCTCCATCGCTCCGGTGCCCGCTTCGACCTCCTGCTCATGGACAAGGAAATGCTTGACAAGGATGAAGTCAATGCGAGCCCATTCTTCCCTTCCCCTCTTTACTCTTATATATCTATTTCAAAAATGTTAAGGTGACAATTTGCACAGCCGATGCACGAGTCTATATAATTCTTATATATGTATTCGAGAAACTTTCTGTTAGAAATAATTATTTCCTAACATTGATTTATATAATAAAGGTTGTAATTTCATAATGGACTCACGTTGATGATTAGTGATATATAATGGTGGAATTACTGAGTGAAATTCCCTCCCTCTCATTTGTTAAAATTTAAGTTTATAAGGGTTAAAAGATGTATAATGGAAAAAAGGATATGGGTTCTCCTAAAAGGAGGTTTCACATTCCTAAAGGGATTTCTATTCTTGAAAGGAGGTTTCCCAATCCTCTTGAAAGAGGGTTTCTCATCACATCTATAAATACCAAGGAATACTCTAGGGATTATATGGCTTTCTAAGCATAAGTTTTTGTCTCGGAGTTTTCTAGTCTCCATATCATCAGGAGGTGATTAAGGCATACAGATCAGAGAGGTGAACCATTCTTGATTATTCAGGTTAGAATTACATGGAATCAAGTAAGTCTTTATCCTTATTCTTGTTTTAATTTAAGATTCATGATCATGAACATTGTTTAGTTGATTGATTTGATTTACATGCGATTCATTGTTAATTGTGCTATCGCATTCAAGTTATAAGCGCATGAAGGTTTCAATTAACATTTGGTATTAGAGCTTGCTTGTAAATTGATTAATCACTAAAATTAGGGTTCATAATATTTGGGTATTTTTAATTATAAGGTTTTAATTGATTGTGGGTTTTAAAGTCGTCACGGTTCGTATTTGTTGGTAATGAATTGATTATATGGATTTCATTCATCAAATCGGCAATTCACAATCAAAGAATTTGATGATTATTTTGTTGAGCCTATTTGGATTGTTTAAGGAATTATGGACTTACTTTAAATTCAAGGCTCATTGAATTTGGGCTTTAAAATTTTTTTAGGGATTATATATAAACTTGGGCTCTTTTGGTTTGTTTAAAACATTTGTACTAGCTTTAAATTTTGGGCTCATATATGTATATAAAGGATCAATTATGGCTGGAGATTTGATCCACTCTTAATTTATATGTTATAATATAATGACATATAAAATAAGGTTTATTCGCATCAATGATTTATTAGCATGAGGCGAGTGCGTTAGTCGCCAAAGCGATCCACCCATGGTAAATAGCTAAATAATCATTTATGTTTATGTTTACAACATAAATTAAGGGTTATTCACATAAATAAATTATTAGCATAAGGCGAGTGTGTTAGTCACCAAAGTGGCTCACTCAAGGTAATAACTAAATAATTATTTATGTTTACAAATGTTTATCGTACGCATGGTATTTTGCATCATTGAATGACACTAAGGATTGATAGACATTTGTGATCATTAACACATAATTCTAGCTGTCACCCAAAGGTGGATTAGATATGTGATTAATGACAATTTAGGAGATTATGATCCTTGTTTTGTTAATATGTAGTGCATGTCCAAAGATGACCTATATATTAAATTTAAACTTGATGAATAATCTCATTCAAGTATGTGTGAATGATGCATGTCGGTAAATTGACATTGTATTTAAATTTGACCCAAAGGCAGATTTGAATGCAAACTTAAGGTTTATAAATCATAGTCTGAATAAAGGTTTGTTAACTCAAAGCAATTAATTAATATGTGAGATAAAAGGTTGCAGTGAACACTATTGTATCATGCTATAATGGTGACATTTATGATGTCAGAGATAATGGCTTTTGCCTTGATATTGAGAATTGCCTGTATATTCCAGAATGTTCTCGAAATCTTGCTTCAGCATCTAGGTTAGACATATTGGGTTTTAACCTTAAGATTAGAAACAAATGTTTTCTTTGTATCATCAAAAGACCTTTTTGTGGTCCTGGTGTTTTGTTGGATACTCTATATTGTTTCAATCTTAATGTAGACTTTGTTAATTCCTTGTTTAATGTTATGAGTGAGGTTTGTGTTATCTTTAACTTACTTGTGGCATAAAAGGCTTGGTCATAAATCTAAATAAAGGACATTGAGACTTATTAAGGATGAAATTTTGTCTCATATGGATTTCCTGACTAGAATGATGCATGTATCGATTACATAAAGGGATAGCAAGTCAGGCTTGGGATTACTTGATACATACAAATATCCTTGGCTCTTTTGATAGGCAAAATCATACTTTTATGAAAATGGTTAGGAGTATGGTTAATAAACTGTATATTGCTTGTTTCATTGTGAATGTATGCATTAAGGACAACAGCATACTTACTCAATAGGGGTCCGAGTAAGGTTGTTTCTAAAACACCTTATGAACTGTGGATGGGAAGGAAACCTAGTTTGAGGGATCTTCACATTTGCGGCTATCAAGCTAAAGTGAGGATATATAATTCACATGAAAATAATTTGGATTCCATAACCATTGGTGGTAATTTCATTGGATATCCAGAAAGGTCTAAAGGATATAGGTTTTATTGTCCTAATCATAGTACGAGGATTGTTGAGTCTGGTAATGCTCACTTCATTGAAAATAACACAGTCTGTGGGAGTGTGGGAGCTCGTAATGTTGAGATTAAGGAGTCATTGATGGATCTATGTTCATCAAGTGATCCTTTGTAG

>g13465

ATGAATCTGGGTGGTGGTCAAGTGGGAAAGGGGATGTCGGCTACGTGCTCAAACGCTTCTTGGAAGTCCGGTGATGCGGTCTCCGACAAGTTTCCGGCGGGTCTCCGGGTGCTGGTTGTGGATGATGACCCCACTTGCCTCAAGATCTTGGAGAAGATGCTTAGGACTTGCCTCTATGAAGGTATCAAATCTCTCTGTACTGTCATATACACAACTATTTGGCCATCAAGTTTTGTTTTTTCTGCAGTTTGGACTGAATTTGTGGTGATTTTTTTGGTTGTTTTTCAATTTATGCAATTGATTTTGTCTGGGTAATTGAAAAGTTCAAGTCTTTTTGCTTTCTTTTTCCAATTTTTGATGGGAATGTTTGAGGGGATTTGGAAATCCCAGGTTTGGATTTGAGTAACTGAGTAAGCATAGAAAAGCTTGGGGATTCTGCTGCAATTTGTATAGCAATTATTCCCAAGATTCAAAATAGTTAGATTTGGGGATAATTCTGTTATTCTTTGAAGTTGAAATTGGAAGATGTCTACAACTAAATTAACACATTCTCATGCCCTTTGAAGTTAGTCCAACTAACATTCAAGATTTTAAATCTTGTGATTGTATATGACTATATGTATATATGTACACACACTCATATATATATATATTTGGGGTGGGGGGGTTTGGTAACTTGTGTGAAATCTAACTAAGAAACACCATTGGTAAGGGTAGATTGCTTGGGTTGGGAATGTTTAATTTGGGGGTAGGAACTTGTGATATCATAAGATTTGTTTTATTTATTTATTTTTTCCTGTTTTCACACAAATTTTCTTGTTTTTTTCCAGTAACCAAGTGCAATAGGGCAGAGCTTGCTCTATCGTATCTTCGGGAAAATAAGAATGGCTTCGATATCGTTATAAGTGATGTCCATATGCCAGACATGGACGGTTTCAAACTTCTCGAGCATGTCGGTCTCGAGATGGACCTGCCTGTAATTAGTAAGTGTGCTGTTTTATTTGGTTTCAGATTACAGCCTAATTTTTACTGTTGTAACGGATGTCAATTTAACGTTTGGTTCTATAATTCTGCAGTGATGTCAGCTGATGATAGCAAGAATGTTGTGATGAAGGGTGTCACGCACGGCGCGTGTGATTATCTGATCAAACCGGTCCGTATCGAGGCACTGAAAAACATATGGCAACATGTGGTTCGCAAAAGGAAGCACGAGTGGAAGGATAAGGATCCCGAGCAATCAGGGAGTGCAGATGAAGGCGATCGGCCTCAAAAACCATCAGACGATGCTGATTACTCGTCTTCAGTAAATGAAGGGAATTGGAAAAACTCCAAGAAAAGAAAGGACGAGGAAGATGAAGCGGAAGAAAGGGATGACACGTCCACGTTGAAGAAGCCACGCGTGGTTTGGTCGGTGGAGCTCCACCAGCAATTTGTCGCAGCAGTAAATCAATTAGGAATCGACAGTATGTACCATTCCCCGTCTTACTTGGCTGGTTGCTTAACGTTCTATGTTAACTTGTTTTCTGTGTAGAACTATGATGCTTCACTTTTCAATCTGCTCTCTACTTTCGTTAGTCATGGCTTTCTGAAGTCTTTTACCTACATAATTTTTACATAAAATGGTAAAATGATGCATCGACCATGAATTTAGCACAGAGGAGAAAACTGCTCACAACTTGCTTTTAACTGTATTGAAGAGTAATCGGTCCTTGATAAGTTGCTGTATTAGAGCTATCCAAGTACTTTTATGATAAGTTTAGTTCCCTATAATTTGGTCTGGGCTAGAAACATGGAATTTTCGTTTTGCAGATGTTATTTTTAGTCCATTAGGGAATGATATATATCTGTCAAGCACAGATATATGGTCGGTCCTCATTTTTCAATACTGTGTTTACACTTCAATGAACATGAATCTTTTGAGTGTCTACTATTTCACGATTTGAATTAGCAGATTGAGCCTAATGATACATGAATCTGTTTTCACGGCATTTTTCTGTTAATGCAGAGGCTGTCCCAAAAAAATTCTGGAATTGATGAATGTTCCTGGGCTTACGAGAGAGAATGTTGCTAGCCACCTTCAGGTAGGTGGAATTCTTTCTTCCTTGTATAATAGACCCGTATAATAAACCGATTTCTATATATGTATATATATGTATAATGTTTTGCAATGAGTTGCTGAGAGAAAGCCTTTCCTTCTTTCCTTCAAAGAGAACTAACTTTACGACCATGGGTTTGCAGAAATATCGTCTTTATCTCAGGAGGTTGAGTGGTCAGGGGGGACTGGGTAATTCTTTTATGGGGCATCCAGAATCGCCCTCCGGGTCTATGTCTTCCTCAATGGGCTTGATCTCCAAGCGGCTAGCCGCCTCGGGTCAAATATCTGCACAGAGTCTTGCTACATTCCAGGCGGCGGCACTGGGCAGTTCTGTCACTAAATCAGCAATATCAATGCCCCTTGTAGACCAAAGAAACCTATTTAGCTTTGAAAACCCAAAGTCGAGATTTGGTGATGGACCGCCTCAACTTGGCAACAGTAGTAAGCAAATCGGTTTGCTTCATGGAATCCCGACAACGATGGAGCCAAAGCAGCTTGCAAGCTTGCACCAATCTTCCCCGACTTTCGGTGGTATGAGTATGCAATTGAACTCCCAGGTGCACCAAAATAACCCTTTGCTAATGCAGATGGCACAACCCCAGCCCCGGGGCTCAAATGGTAAGCGATCCAAATGGCAGTCAAGCTTCACGGCTGCCGTTATCGGTGCCACAGCCCATTTTGTCGAGTGCAATGGCGGGTGGAGTCCTCGGAGGGAATAGTATCGTTGACAATTCGTGCAGTGCCATTCACAGTTCTGTTTCCCATGCCCCATCAACAGTGGCTTTCTCTGTAAACCAGGGCACCGAGTTGCAAACTAATAGCTATACTACTAGCAATTCTGGAGTGTCGTCTCTAACATCTATAGGAATGCTCCGAGAACAGGCTAATCCCGATGTCAAAGGATCGAGAGGATTTGTTCCCAGTTATGATATATTTAACGATCTCCATCAGCATAAAGCTCAGGATTGGGGTTTACAAAACGTCGGATCCACTTTTGATCCCCCTCCTCACCATTCAAATTTACAAGGAATCCTCGACCCCCCACCCTCAGTTATGGCTCAACACGGGTTTTCCTCTAACCAAAAGAGTGGACAGAACAGAAACGCACCCATCAACAAGGACGTTTTCTTAAGCTGGGAACAAACCGGGCATGGAAACAACCCGATGCTCGGTCCACAATTCAACTCACTACTCGGAGGAAATCCAGTCACAATCAAGACCGAGAGACTCCCTGATACGAGCTTTCAGAACACGCTTTTCTCCGACCAATATGGCCAGGAAGACCTAATGAGTGCCCTTCTCAAACAGGTTAGTCATTTCCTGTACTCCTTTTGCACTGTGAATGTGCTAAAAGCATCTCACCGTTCTAACATTTCTTATATGCAGCAGCAAGATAGCCTTGGGCCAGTCGAGAACGAGTTTGGCTTCGATGGATACCAATTGGATAATCTTCCAGTGTAGGATGTTTCTACTGTCTCTAGCTTGCATTATATGGATATATGGTTGCAGTTACTGGTGTACATAGTTGCACTTACACCCGAAAAAAAAAACTTATTTGATGTTGGATACTGGCCTCACCTTGCTGTCCCGTCTTCTTTGTTAGTTTACATCCCACCGCTGATCCATTCTAGAACAAACATCGCACTCGAGCCAAAATAATGGATGGATGAAGGAGAAGGAGAAGTGTGTACTATACTGCTGAACTATCTGCAAAAGGAATACTCGCAAAAGTTTTGACTGCTCTAGGTAGGTGTGATTGTAGGGAGATCGGTTCATATATAAGTGCTCGTGATGAAGTATCAGATTCGGCGCAAGTCTTCTTCGGTGGGTAG

>g6082

ATGTCAGTTCATAGCTCAGTTGCTTCTTGGAAGCCCGTTGATGGGGTCTCCGACCAGTTTCCGGTGGGTCTCCGGGTGCTTGTTGTGGATGATGACCCCACTTGTCTCAGGATCTTAGAGAAGATGCTAAGAAATTGCCGCTATGAAGGTATCCAATCTTCATTTCACGCGTACAGTACAGTTGTATATATGAAGATTATGATTATCACTATCATTATTATTCTGTCTGAAGCTAAATGTTTGGTTTGATTTTATCTTTGATGATTGAAACTTTCGAATCTGTATATGTTTGTGTGAGAAATTGGTTGCAGAGATTAGAAATCTGGGGTAATTTAGAAGAATTAGGAATGGGATCAAATCATGGAGATCGATTTGACTGTTATATATATCATTTAACAACTAATTCAGGGTTCAATTGTTTGGGAGTCTCTTTTCTTTGTGTATTGAAGCTGAAATGGGAAGTTGATTTCACTTTAGATCTTGTATATACAAAACCCCTTGGTGTTTTTGAAGTTCCCATGCTTATTCAATTTCAATTCTGTGAGAAGTTTAACATATTGGGTGTTGGGCAGAGGCCGGTAAAGGGCCAAGTCTAGTACCCTGCCCTCTTGAGAATGTGATGGCGGTTTATAAGGAAATAAAGTTGTGTCTGCGCTAAAGCTATAGCTTTTGGTGTAATGGTAAGCGCTTGATCCTGATATTGGGGTTTTGAAACTCCATTGGTTAGAGAAGATTTTGTTTGGAGTGGTGTGCCTTTGGAGATGAGAAATTGTGATCTTGTTGGTTTTTGGATCTTGTAATTGAAGTTTTTCCTTTTTTATGTTCTTTTTCTTTCAGTAACTACATGCAATATGGCTGAGGTTGCATTATCTATGCTCCGGGAAAATAAAAATGGCTTTGATATTGTTCTAAGCGATGTACACATGCCAGACATGGATGGTTTCAAGCTTCTTGAGTGCATTGGACTTGAGATGGATTTGCCTGTTATTAGTAAGTAAACTGTCTGTGTTATTAGTTTCAGATTCCACCAACGCTAAGACTAAGAATTAGTTAGTTTGGGCTTACACGGGCTTAGCTCACTCAGTAGGCAATATTTGAGAGAAGAGACTAATGTTTGAAACTCGGCAGCTACAGTATGGGGATTATGCCCAATTTGGGCAAATCCTTTGTGCGCACCGGCATTTGACCCTGACTGTTGAGTCAGTGAGTTACCGTGATTAACATGTCGTTCTGTAATGTGGCAGTGATGTCGGCAGATGATAGTAAGAATGTCGTGATGAAGGGTGTTACGCACGGTGCGTATGATTATCTGATAAAACCAGTCCGGATTGAAGCGCTGAAGAACATATGGCAACATGTTGTTCGCAAAAGGAAGCAGGAGTTGAAGGACAAGGATGTTGAACAATCGGGAAGTGTGGAAGAAGGAGACCGACAGCAAAAACCATCTGAAGATGTCGATTACTCATCTTCGGTAAATGAAGGAAATTGGAAATGCTTGAAGAAAAGGAAGGACGAGGAAGATGAAGGCGAAGAGAGGGATGAGACATCTGCACTGAAGAAGCCACGTGTGGTTTGGTCGGTGGAGCTCCATCAACAATTTGTAGCAGCTGTTAATCAATTGGGAATCGACAGTATGTATCATGCTTTGGTTTATATGTTTAGCCTATACTAGCTTCTATCTTGTTTCACACGTGTTTTCCCCGTCCTTTTGTTACAATTTACTCTCTTTTTTCCCATATCCCTCTCTAGTTAACACAGTTTATATGTTTAGCTTTCTATCCACTATATATGCCATAATATTTCTTGGATTTCCCTGAATCAATTCCCTCGTCCTCCTATTCATGCAGAGGCTGTTCCAAAGAAAATTCTGGAATTGATGAATGTACCTGGGCTTACTAGAGAAAATGTTGCAAGCCACCTGCAGGTCAGGAAGTTTTCTTTTTATACTTTTAAGTTTTAGCAGTGCAACAGTCTGCATAATGTTGCGACCTGACTCTGATATCACTTGTCAGATCAAGTGCTTATCATCACGCCAAAAGCTATAGCTAGTGCCTAGTAGCGAAGGCGTAACTTTATTTCCTTATAGACTACCGTCACATTTTGCAGAGGTAGGGTTTTGGACTTGGCCCTTCACTGGCCTCTGCCCAACAATCTGCCTAGCACCAATTGATGGACTTGGCCATTTACTGGTCTCCGCCTAACACATTTTTGCAACACATAGTTGCTTGAAAGCTTTCCATTATTATTATTATTATTTTTTTTTTGAAAAGCACTCATGTTTTGAACATTGGTAGCAGAAATATCGCCTTTACCTAAGGAGGCTGAGTGGTGTGTCACAGCATCAAAGTGGACTGAACAGCTCTTTCATGGGGCCACCCGACACGACCTTTGGGGCGATGTCTTCCCTTAATGGACTCGATTTTCAAACCCTAGCGGCCACGGGCCAAATTTCAGCTCAGAGTCTGGCTTCACTCCAGGCAGCAGCACTTGGCAGGTCAGCTACAAAGCCAGCAATATCGATGCCCCTAGTAGATCAAAGAAACCTGTTTAGCTTTGAAAACCCAAAGTTCAGATTTGTTGAAGGCCAGCAGCCACTGAACAATAATAGTAAACAAATTGGTTTGCTTCATGGAATCCCTACGACTATGGAGCCAAAGCAGCTCGCAAGCCTGAACCAATCTTCACAGACTTTCAGGGGTTGGGGTATGCAACCTCCGGTGCACCAAAACAACTCCTTACTAATGCAGCAGATGGGGCCACCCCAGTCACAGGCTCACATGTTAAATGAACCCAATGGCACTCAAGTTTCGAGGGTGACACAGCCCATATTATCTAATGGGATGCCTAGTGAGCTATTGGCACGAAATGGTATTGTTGACAATTCGCGTGGTGCCATATACCAACCCGTGTCCCAGGCCCAACCACTGGTAGATTTCTCGGTGAATCAGAATACAGAGATGCAGGGCAATAATTTCATAAGTGGCAACTCGGGAATGCCATGTCTAACATCTAAAGGATGATGATCCAGGAAGGAGTTAACTCCGATGTCAAAAGACCAGGAGGGGCTTTGCTCCTCCCAGTTATGATATATTCAATGACCTCCAACAGCATAAAGCACAAGATGACTGGGGCATGGGAGCAGTTTTCGAGGCCTCTCGTCTCCCAAATGCACAAGGAACCCTAGATGCGTCACAATCAGTGATGGTTCAGCAAGGGTTTTCTTCAAGCCAGAACAGTGCACAAAATGGGCGCGTGTCTATTGGCAAAGCTGTGTTTCCAAGTGGGCAAGAAAGCGGGAACCCAATGGTTGGTCCACAATTGAACTCACTGCTCGGTGGCAATTCTATAACAATCAAAGCTGAAAGACTCCCTGATGCAAGCTATCAGAACACACTTTTCCCCGACCAGCATGGCCAGGACGACCTCATGAGTGCTCTTCTCAAACAGGTAATTTTCATTTCAATATTCTAGCTTGCATAACTATTGCATTTAGCTGACGGGCTGCTTGCTTGTACAGCAAGAAAGCGTAGGACCAGTTGAAAACGAGTTCAGCTTTGATGGATTTCAATTGGGCAATCTTCCAGTCTAA

>g50246

ATGCAGGTTGAATTCATCAATGACATAGCAGCTTGCTGCTCGGTTGCAGCTCTGACAGAGGTACGGAGTTCCGAGTGGTCCTGGGCGTCGTACGGAGTGTAATTGTGTGGTTTGATTTCTCGCGCGGTAGGGTTGTGATTTGTGATGGCTGCCGTATGTAAGGCGGAGGCTGCTGCGGTTGTGCCGGAGCAGTTCCCGGTTGGGCTCAGGGTGCTAGTAGTTGACGACGACCTTCTTTGCCTCAGAATTATAGAGCAAATGCTTCGCAAGTGCAAGTACAATGGTTTGTTTTTTGTTTTTGTATTATTTACTTATTTTTATTGCTTCCCATTTGATCAACGTATCTTTTCCGTGAAATAGCGTCATTTAGTATATTTTGCTTGGTGATTTGAGATGTTTTTGAACTATTAGTTTGTAATTCCGCTAATCCGCTTTGAGATTTATTTATTTATTTATTGCCACACTTCATTGAGAGTGGTGTCTCCGTTGGCGGGATAAGAAATATCATCTTTAAAATTTTTTTTGGCTGTTTTCCATGGTGATAAGCATAAGTTTAGTCCTTTTCTTATATTTTTTGAGAATTGTCCTTGATTTGTAAACACAATTCTGAAGTTTTTCAAGTACTTGAGTTGATATATTTTCGAGCAAATTCCACTTCTAGTACTGGACTATTGTGGCATTGCCACATTTAGCCCTACTCTTTAAAATTTGCCTCTTGACATCCTCACTTTTACGGTTAGTCTTCTGGTTAAATTTTTGTTATTTTACAATTTAGTAAAGACTGCTAACATGTAATCCGTTCAAGTAAAGGGGTTTACGAAATAGGAAGATAAATTACATGTTGGGACAAGAGACTTGAATGTGCCAATGCTCTATTAGTGACTTGAATGCTTTTATGTCCACCTATAAAGCATCAGGTTGATCATTTAGTTTGATTATATCATTTGTGGATCTGTTATACAAGTCTTTGCAATTGCTATAAATCCTCTATCCTCTTTGCTCTAGCAAATTAAATTGTTAAAATCCATCCTTACCACTTTTGGTTTCTTTTTAACTGATTGCAGTGACCATTTGTTCCCAAGCAACTGCAGCATTAAACTTATTACGGGGAAAGGAGAGGCTGTTTTGACATTGTGATAAGTGATGTCCATATGCCTGACATGGATGGATTCAAACTTCTTGAGCATGTTGGGCTGGAAATGGACCTTCCTGTTATAAGTGAGTGATAAACTGATAATTCTCTTCCTGAAATTCATGAAATATTTCTTTGAACTGATACATACCTGTTTAATGTTTTGCAGTGATGTCAGCAGATGGAAGAACTAACCTTGTCATGAGGGGAATTCGACATGGGGCTTGTGATTATCTGATTAAGCCTATACGTGATGAGGAGTTGAAGAATATCTGGCAGCATGTTGTTAGGAAGAAGTGTAATCTAAGTAAAGAAAATGACCATTCTGGCAGCTTTGAAGATAATGATCAGCCTAAACAGGGAGGTGATGATGCTGAGCATGCTTCTTCTGTTATTGAAGGAGCTGATGGAGTCTTGAAAACGGTGAAAAAGAAAAGAGATTTTAAAGATGATGACGACGATGATGATGATGATGAAATAGAAAATGATGACCCAGCTAATGCAAAGAAACCACGTGTGGTTTGGTCAGTGGAACTTCATCAACAGTTTGTTAGTGCTGTTAACCAACTTGGGATTGACAGTATGAACTTCTGCAACTTTGTCTTCTATGAATTTTATTTACAAAATATATCCTTACCACAGGATTTCCAATTTTAATATTTCTAACTTTTATTTGTTGTATTTCTTTACAGAGGCTGTACCTAAGAGAATCCTAGAATTAATGAATGTTCCTGGTTTAACAAGAGAAAATGTTGCTAGCCACTTGCAGGTGCAATGTCTTATAGAAATTGTATAAATGCGTTACTTTTCATGTTAATATGGTTCACTTTTTACCTCGCCCATATACATAAAGGTACAATGGGAGAATCACAAGCACTTCAAATATATCTTCCACGAAAAGAGTACACCAACATTAACTCACGCTCTATCATTATAAAAAAAACAGAGATAGCCCTTATGCTTTGAGTGATATGCTATTTGACCAATTGACCCCAATAAGGGATGGATTTATGGAAGGGGTAGGACAGTGGAGGGGGAACATGTGTGTGTATAACACAGAAGCTGTGTTTGTTATTCTGTTGTTAGTATATGGGCGAAATGCATACATCTTAATCACTAATTTTTCCCTGAATGATTATTAATTAATTAATTAATTAATTATTATTATTATTATTATTATTATTATTATTATTATTATTTGGGGATCTATAATTCTATATATATGGTCCCTGGATTTACTACATCACTTTCTAAGAGACTAGATGACTAATGCCAGGGGGTTCTAAATAACTGAGGAGTTGTCCTACTGTTATAAGATGCAACATGGTTCTATACAGTTCTTTTACCTTTCAAAAAAGCATAACCATCTTACCATCATCATTTATTTGTTTCTTTTTCCTTCTTTGTGTGGTCAGTAGAAGGATGGTTTAATATAGTAAGGTTTAGAGGCTAAAGTTAAAGGAAATATGGCTACACTGGTGTTTTTCTGCAGTATATCACCTTTTAATGTGTTTGGAAGTACTCCATATGTTGTTAATTTGCACCTACTAGAACCATGGTATTTGTAAAAGATTCTGATGATTATTAAATTGTGTCTAGTATTTAGAGAAAATCCGTATGTTTGTGGAGAAGGTACATAGTGATATGGTGATACCCTGGATATATCTCTAGAGTGTTGTGTTTCCTCTATCTCAACAAATTCAACTAGTGCTTAAATATGCAATTTAGTTATCTTTTTACAACTAAATAATGACGTCCTAGGATTTGGACAAACACCAGTTTTTTTGTAAGCAAAGTTCATAGTTGTCTTATAGCCACAGTGCACACTCATTTCTATTTGGTAACTCTTTGATTCCCAACATGCAGAAATTTAGGCTTTATTTGAAACGGCTGAGTGGAGTTGCTCAACAACAGGGTGGCCTTCCTAATTCCTTTTGTGGACCAATTGAACCAAATCCAAAGCTGGGTTCTTTGGGGAGATATGAAATTCAAGCACTAGCAGCCTCCTGCCAAATTGCTCCACAAACTTTGGCAGCTATTCATGCTGAGCTTTTAGGGCGACCTACAAGTGGTCTAGTTTTGCCAACAATTGACCATCCAGCTCTGCTACAAGCATCTCTGCCAGGCACAAAATACATTCTTGATGATCAAGCTGTGGCTTATGGTCAGCCTCTGATGAAGTGCCCACCAAACATTTCCAAACAATTTACTCAACATTTGTCAGCAGAGGATATTCCTTCAGGTGTCGGAGCTTGGCCCCAAAAATGTTTGTGTGGTTCCCAGCATCAATCTTAGTGGTCTAGGTGCTCAAAATGGTAATATGTTAACCACAATGATGCAACACCATCAACAACAGCAAAAACAGCAACAAATGGAGCAACATCAGAAGTTATCTACTATTCCAGAGTCCTGCCGTCCGGTTAATGTGCGGCCCTCATGCCTAGTGGTTCCATCTCAGTCTTCAGCCAATTTTCAAGTCACAAATAGTCCTGCTTCAATCAGCCAGACCAGCAGTTTTAGCAAAAGTAATGTAATGGATTCTAGAATTCTTTCTCCACAATCAGGTAATTCTTCTTCAGGTGCTGGGGAAGTAGCAAATTGGGAACAGAAACTCCCATGTAGGTCTAATATGTTTTGCGCTACAGGTTCTCTCTCTCCATCTCTCTCATCATGCTCTACGAATGCTGACAACAGTGCTAGCTGGCAAGTTCAGAACTCAGCATGTATCATTGGTGCTTCTAGACATGCGGCAGGTGTTGTGCCTAACATTACTGGCATCCCTGTTCCAGACAATCACAAATCAAATCAATTGCTTGACCAGGGACCGATTAGGAATCTTGGGTTTGCCAGTAGAGGATCATCCATTCCTAGTCGCTTTGCTATTGATGAATCTGAATCACCACCAATAAGTAACATATATCATTCAAGAATCTATAAGGAAAGCAATACATGCAAAGTGAAGCAGGAGCCAGATGTGAATATTGCTGACAATGCTAAAGTTAGTGTCCAAACGCTGCAAAGAATTCCTCCAAATGACTTCATGAGTGTTTTTCAGTGA

>g26505

ATGACTGTTGATGAAAGTAGAAGAAGGGTTGAAAAGGAAAATAATTCTGACAATTTCCCAGTGGGTATGCGTGTTCTTGCTGTTGATGATGACCCAATTTGCTTGAGGTTATTGGAATGTCTGCTTCGGAAATGCCAGTACCATGGTATATATGTTTATTGATCTCTTGTTAAATCTTCCAGGTTTTGTTTGTTTGGCTTCTCAAGTGAATTTTATGAATTAGTCTGTAACTTTCTTTATCGCCCATACCAGGTTATCGTTAAGAATTTTGTTTTTATATATCTTGATTGATGAATTCTCTTTTCATTGTTGAGTTGTAGTGTCCAAATTCAAGGGAAATTAAGTATTAACTGATTGAGATTTTTTACTTTCATATATCATAGTGTTCCAGAACTTCATTTATTTGCATGTTGCATGCATGTTTCTGGAGGAAGAAGAAAAGAGATTAGCAGCTTCTCTTGTCTTTATTAGTTTTGGCCTTTTGGCTTTGTTTTTTTGTCTGTTTTCTTCTTTTTTGGGGTGCTGGGGGCTTATAGGAACTGATTGTGGCATTTCAGTTTGTACTTTTGTGACTAATTGTGTTTTTCCTTATTTATACTGCTTGCAGGATCAATCCAAATCCCAAAAATAGTGTTTTTTCTTTATAATTGGTCCCCCATTAGTCCAACTAAACCAAAAATTATGTTTGATTCCTTTCATTGGAGTTGTGTTGTCAAATTAGTACATTGTTTTCTGCCAATGTTTATGTTCTGCTGTTATAAGTCTTGTCTGCTGGTTGGTGTTTGAACATAGGCCATACTTGCCAAACACACAAATGTTTTTGAAGATTTTATCCTAGATTTTGTTCCTCATTCCTCGATTGAATGCCTGTTGCAAGTTGCAACTGTTCATTGTGATATTGAAAATATTCGATTAAGTTAGTTTTATCTTGAATATGATTTGGGATTCTTTTGCAGTAACTGTAACGAATCAAGCAACAACGGCACTGGAGATGTTGAGAGAAAACAAGGACAGATTTGATCTGGTGATCAGTGATGTCTATATGCCTGATATGGATGGTTTTAAACTGCTAGAGCTTGTTGGTCTTGAGATGGACCTTCCTGTAATCAGTAAGCATTCGAACTTATATTTTATTCGCATCAAATATTTTATTCCGAGGAGGCGTCTTTCTTTGATATGTGAATAGGAATAATAGTAACGGTAGGATCATTTTCTGAACGTTATCAGTTTTGCAACGTTTCTAACATCTTTGAAATTCACTAGTGTTGTCGGCAAACAGTGATACCAGCCTTGTAATGAAGGGAGTTACTCATGGTGCTTGCGACTACTTGGTGAAACCTGTGCGGATCGAGGAGCTGCGCAACATTTGGCAACATGTAATCAGGAGAAAGACGTTCGATTCCAAACACCATAGCAAGTCCGGTGATCAGGACAATGAAGAAGAAGGTAGACAGGGGGACCAATTATCAGGTACTGCAGAGCAGAGCGAAAAACTGAATAAGAGAAGAAAGGATGAGGAAGACGGGAGTGAGAATGAAGACCCGGCAACGCAGAAAAAGCCCCGTGTTGTTTGGTCTGCAGAACTTCACAGGAAGTTTGTTGCTGCTGTTAACCATTTGGGCATCGAAAGTGAGCTTTTCAGCACCAATGCCTATCGTTCCACTAGTATCTCAGTTTTGGAACTTCTCCTTTTCGTGTTATTCATCATCTCCGTTCTTTAAATGCAGAAGCTGTTCCTAAAAGGATTCTTGATATGATGAACGTTGAAGGGCTTAGTCGAGAAAATGTGGCAAGCCATCTCCAGGTACCAATTTGACTTTCATTTCGTTTTAAAATATTATTATTGTTGTTATTATTTGCTATACATAAAGTTTTGTTTCTTGAAGCAAAAATCATTAAACAGCATGATTAATCATTCAAAAATTTGGAGTGATGGATTTAAATATTCCCTAACGAGGCACTATTATTTGAACATTTTTGTAATAAATTTTAGTAACTGTGTTGCAGAAGTATAGGCTTTACTTGAAAAGGATCAGTTTGGTTTCAACCCCACAGGCCAACATGGCAACGCCATATATGCCGATGGGTTCACTAGGTGGGTTTGGAGATTTGCAAACATTGGCTGGACCTGGACAACTTAACCGTGCTACATTATCACCATATGTCCCAGGCAGCCTGCTCGGTAGGCTAAATAGCTCTGCTGGTGTAAGCCTTCAAAACCGAAACTTATTGGGTTTGCTCCAACCAAGCCATGCCCAATCTTCTGGTAACTCCCTTGATCTGCTCGGGAAGTTGAACTCAAATGCTCCGCCCACAAGCCAGAACCCGAGTTTGTTTCGAGGAATTCCATCGTTAGAGCTTGATCAGTTGCAACACGGTAAATTCCCCAAAATCGAACAGGTCCTCAATCCGATGGACAACTCGAAGCTTCTCACAGCTGCTACTACATTCACTGGTTCGGGATCTGCTTTTGGTAACCCAATCAATGCTACGATCCTTCAAGGGAACTCCCAGCAAGGACAAACCGGGGAAGGGTTTGGAAATCCACATTCACTCAATATGGCTTCTTTAAGGCCTGAGCCTTTGAACACTGGTGTTAGCAGTGCTTCGAACTTTCTTGGCCATGGAGGACTGAATGGGAATTTGGGGAATTCTATTTTGGCATCAAATGTTCAGCCGAATTGTTATCCGTTGATGGAAACCTTTACTCATAGCCAGTTGGATCAGAATCATGTAAGAGGGAATTATTCGCCCGCTGGCCCTCATTTGCAGAGCAGCCCTCTTGGTTATAATTCTACTTTCTCAACCTCTATACCTTATGAAAACTCGAGGGGACAAACACAATACCAAGAAGGCTTCATCGGTGATGCTATTCAGAGTGTGAATCAGGCACCTACCCAATTTTGGGGAGATCATAACTCAAACTTAAATAATGTTTTCAGCAACTCGAGCTCTCAGATTCTTGGTAATGGATTAATGCCTCCCTTAAGCCAGATTGCCGACCAAAATAATGATATCTTCAATATGAAAACGGATACACCTTTGATTGGTCAAGAAAATGGAGGCTCTGTGGTTCTCTTTCCGCACAATGAGAACGCAAATTTCAATCAAGACTCAAGGATGGGATCCAATGAAGACTACATGTTGAACTCCGCAAAACCACAGGGTGCTTATTCTTCTCTGGATGATTTAATGAATGGAGTAATCAAGGGGGTATGAAATCCTTCCAACCATGTCTCTTTAATTCTCAATATCTTCAGAATGCCCGCATTTCATTCTAATAAACCTCCCGTGATGCAGGAACAAAATGGGCAATTCGGATTTGATGATTACTTATTTGGGTCATGA

>g50506

ATGGAGAGTGTCATGGCCGGCGGGATCTTTCTACCCAGGAGCGAAACTTTTCCGGCCGGTCTCCGGGTTCTCGTCGTCGACGATGATCCAACCTGGTTGAAGATTCTTGAGAAGATGCTCAAGAAGTGCTCATATGAAGGTTTTCCATCACTTGCAAAAATACCCTTTTGCTTTGTTTTTAACTTAGTGTTTCTGACAAAAAAAGATTTCATTTTTAGCATGTAAACTTCATAATTTTGTGTTTTATGGCTGAAATCCTAGGTTGTCAACTCCATATCCTTCTTTTATCAGTTAATCTAAGCATATCTGTTGTTTGGTTCTGTGTAATAATCTTAGGTTGGGCTTCTGCACCCATTTTTTTTTGGAATTCTTGGACTTTATACTCATATCTGATCATGTTTGACTCATATCTAAGGAGACAACAATGGCCATGTTTAGTGTTCTTAAACACAGGAACTTAAGAATCTTAGGCTTTTGAGGTTTTACTGATGGTGTTGCATATGTATAGTGCTTAAGAGCAAATGATGTGAATTTAGAGGGTTTTTTATGCTATAAAATTTGGTTTCTTGAAAAAAAAAAGAGAAGACATGTCTATGAGGAATAGGACATAAAGTTTTCAAAATCATTCTTGTCAGCAATTGATAACAGCAGAAATGTTATGTCTTCAATCCTTGCAGTGATTTCCATATTTTACAACCCCATAGTTGAAAATTTTGAGGGATTTTTATTTTTCAGTTAAGATTTGCATTTAGAATTTACAAACCGAATTGCTCCTGTGTAATATTGCAGCTGCAGGAGTCTATAGGTGGACTCAATCTTTCTTGAGATTATTCTTTCCTTTTTTTGAGTGACGAGGGAAACCTACAGCCACTAACCAAGGGTGTGCATTGGGTAAACCTCGCCTTGTGACCTTTGCTGCAAAAGGACCACTAAAAGGTAAACCAGCCTTGGTTGCCCATAGTTGGCCGGCTTAAACTAATAAGGCAAAAGACACTGGTCCTCCACCTGGGGGTGCCTGGACAATTTTATAAATATAAAAATAAAGCAACCATTTTCAAAGTATTTGTTAATGTAGATGACTAATATGCAAAAGCATTCAACATGTGTTTGCTTTCTTTCAGCCTTGGCTGATGAGGATAACCCCGTTGTGTAATTTGAGCTGTGCATTTGATTTCCATTTCCACTTGGCATTCTACTGAACACAAAGCTCGAACTCCATGTTTTCCAATTTGGTTGTGCTTTTGAAATGAAAAATCTCGGTTGTTTCAGTAACTACATGTGGTCTTGCAACGGAGGCTATTAGCCTGCTCCGAGAAAGAAGAAATGGATTTGACATTGTAATAAGCGATGTTAACATGCCTGATATGGACGGTTTCAAGCTTCTGGAACTCGTTGGACTTGAGATGGACCTTCCAGTTATAAGTCAGTTTACTATCCTTAACGCATTCTGCTTCTCTCTCCCTAGATTTGATTTAAATATTCGATATTTATAGTCCTCTTCTAATTGTTTCAGTGATGTCTGTTGATGGAGAAACAAGCAGGGTTATGAAGGGTGTACAGCACGGTGCCTGCGATTATCTTTTAAAGCCTATTAGGATGAAGGAGCTAAGAAACATATGGCAGCATGTAGTTAGGAAGAGGATGCAAGAATCACGAGACATAGAAAATCACGAAGGAGACCAATTCGATGAAGCTTGGATGTTTAATGGAATTGAACTACAATCGGGAAAGAAAAGAAAAGATTTTGATTACAAATTTGACGAGAGAGAAACAAGTGATTCTAGAAGTGGCGACCCTTCCTCTGTGAAGAAGCCTAGGGTAGTTTGGACGGTAGATCTCCATCAGAAATTTGTCAAAGCTGTAAATCACATTGGATTCGATAGTAAGTTTGTTGCACCATCACTTCCTCTTAACTTGATTCACTATGATATAATACTCAATTCAAATCCTACCCACATTTTGGTCGAGGTAGAGAACATAAATATCTTTTTCTTCTGGCAGAGGTTGGTCCGAAGAAGATTTTGGACTTAATGGGTGTACCTTGGTTGACTCGAGAAAATGTTGCTAGCCACTTGCAGGTAAATATCCTTCAAACTCTCACTGATAAGGCGGGAGTGGATTTTGCCAAAATAGGCTGTTCAAAAGGAAGTTATAATTGCTCACATACACTTCTAATATGGAAACAGTTTCAAAACACGCCTTCCTAAAATCCTATACAATGTTTAGAGACTGGAAAACAAAATATAAAGTTTGAACAAAGATTGCCAATGGGCTTGCTTTTAGTTTTTATGATGTGTTTTGAAGAGATAATGAATGTATAAATCTATTGTTTTTCTTTGTGGGAAGAGAAAGCGGTATTGCACATCTCGATTTGTGAGAGTGGTCTTGTTTTGTTTTGCAGAAGTATCGCCTATACTTGACGAGGTTGCAGAAAGAGAATGAGCTTAAAGCCTCGTCCAGTGGGACAAAGCACCCGGATCTCTCTCCAAAAGAATCCTCTTCAAGTGCGTGCCTTCAGAATTTAGTTGACGTGAAACCAAGTAAGTCAACAAATGGAAAGTACGCTTTTCATGGGGAAAAATTTTGTGTGCAAGAGGTTGAGTCCAGAAATTACGAGGGCGAGGTTAAGGCTGCTGCTCCGTTGTCAACAGCGGGCGTGAGCAGGGCTCTAGTAGGCGAAAATTGTGATTCTCAGAAGAGTATCAGTTGTTCAAAAGCGAGCTGGGCTAGTGAAGTTTCCAAAACCGGTTTCAAACATGAATTCAAGCCGCAGATTCAAACAGAGGACAACATTAACCATCTACCTTCACCGAAGCTTCCTCGAAATGTCCATCTCGATCAAGCACAACCTCTCCTCAATCTTGCGCCTCACAAAGACATAAACCCCGGGGAAATAAAGAGTAAGCCTGGCAACATCAATACCGAAAATCCCGGGGTAAGAACAGTATCTCCGTTAGAATGTGCTGTAGACTTGTTGCCTGCTCAGCCTTCTCAGCCTCAGAGTTGCTTGACAAATTTTCAAGCTTTCGAGCAAATTCCCAGTACTACATGGAGCGCGAAGACTCCCCAGATTCTAATAAACGGTTTAGAATCCGTAGAGGGAAACCTTTTCCTTGGAGGGGGATCATGGGACAAGGATTTTAATGCTGCTGCTCTTCAAGGCGAATTTCATTCCCCATGTGTTGTTGGCCCTCAAAGTTTAGAGCTGTTGGACTACAGCAACACAAACCTCACTGGTGAAATACAACCTTACTTTTATGACTATGAGTATGCCATTGATCCTGTGATAGATCATGGTCTGTTTATATTATGA

>g22427

ATGACTGTAGAGCAGAGGAATGATAAGCAAAATGATCAGTTTCCACTGGGGATGAGAGTGTTGGCTGTGGATGACAACCCAACTTGCCTCATGGTGTTGGAAAATCTGCTTCGGAAATGCCAGTACCATGGTCTGTGTTCTTTCTTCTGTCCCATTTGTTCTGCCAGCAATACACATAGATAGATGCTTGTGTCTATATTGATATTGTGGGTGTGTGTGAGAGTGAATATCTGATTTGTTTTGTTTTTGTTTTTGTTTTCCTTTTTTCAGATTTCAGTTGTTTTGTTTGGGTTTTCCTTTCTCTATAATGAGTTGAAGTATATTTATGCATGTAGAAGAGAAACTATACCTCCAATCAGTGAGTGCCTGTTTTGTTAAAACTATTTAATCACATTGTTGATTTTATGATTCAGATTATTGATTCAATAGTTCGCTTTAAGTGTTTGAGAAAAAAAATATAGTTATGGATTGCTCAATGGTAGCCAATAATCCATATGCTATTTGGGTTTTAGCCATTGATTATTCACAATCTTCAATAATAAGCTTTACCTCTTTACTAAATGGGATGGGACCTATATTTTATTAGTTGTGGTATAAAGTTTGGATTTTCTTCTCATCTTGTGACTAGATATATTCACACATCATATGTGGTGACTGTTTGGTCAATGGAATGGGAGCAAAGGCACCACTTGTACTGATAGTCTGCTTCGTAGGGTGTAAATGTAAAATGCTGTACTTTGAAATGTTTTCATGTAATAAGATGTTGAAGTTTTGGTCACAGTTGCAGTTCTGCTCGAATCAGCTTCTGTAATAGACACGGTCTTGGTCATTAATTCTCTGTTCCTATTTGCAGTCACGACAACAAACCAGGCCATCCAGGCATTGCAATTATTACAGAGAACAAAATCAGTTTGATTTGGTCATTAGTGATGTTGATATGCCAGATATGGATGGTTTCAAGCTGCTAGAGCTTGTAGGGCTGGAGATGGATTTACCTGTCATAAGTGAGTGATAGAACCCTAACTCACGGTGACATTATTATGGAACATTTTCGTAATTTATAGCGTTCATATTGACATATACATTTTCATTAGATATGAACACTAACTTATGGTTATTCTTATTTTGTAGAACAAGGATGTTTTTTGAAACAGCTTTTATTTGAAAGAAGAGTTGAAATCAAAATATCTTATTTCCAATTTAATTGTCTATATTATTCAAACTTTAGAGCATGAACACAATATGTTTCACTTTAGTATTTGATAATCCTTGATTAATTGTTTTCAATTTCTGTGATTCTAGTGCTATCAGCATATGGTGATACGAACCTTGTGATGAAGGGTATCACCCACAGACTTGTGATTATTTGCTGAAGCCTGTCCGCATTGAAGAGCTAAAGAACATATGGCAACATGTACTAAGGAGAAAGAAGTTTGACTGCGAGGAGCAGAAAATTTCTAACAAACCTGATGGTGAGTCTGGTGAACTAGGCAGAGGGTTCAGAGGGATGGGAGAAACTGATCGGAATGGTAAGCCTACCAGGAAAAGGAAGGACCAGAGTGATGACGAAGATGAGGAGCTTGATGAAAACGGTGGGCGAAATGAGGACCCATCAGCTCAGAAAAACCTCGTGTTGTTTGGTCAGTAGAATTGCACCAAAATTTGTTGCTGCAGTTAATCACCTGGGAATTGATAGTAAGTTTTCATCTTCATCAAAAAATTTGGCAGGTCTTTCAGAGGGATCCTCTGTAATTTCACTTTATTAGTTAGCTTATCATTAACATGTTGCTCTTCACGACTTGATATTCTTTTATCCTTCTTTGACAACATCTGTTACTCAATTTGATGAAGAACATATGATAATTTTGAATGCATTGTTTCTGTACCATTATTTATTGAAATTTTCGTTCATCTTGGTATTGTAGTATAACTGACCCAGTATTTTTATCAAGTTCCGAGACTTGGATTTGTCATCTTTACTTTCTATCATTCCAACCATGCTCTATTTTCTTATAATATTCATAATTTTTTTTTTTAAATTGAAAAGCTTTAGGAGCATAGAGAATGCAGTTTCATCTACATATTCTCATTATTATGTTCTTATTTGCATTATAGAGGCTATGCCTAAAAGAATTTTAGAGTTGATGAATGTGGAGAAGCTGACCCGAGAAAATGTGGCAAGTCATCTCCAGGCAAATTCTGTGCCCTTGGAACTTTTTTGATGGACTGCTTTCACTTTTACTAGGCTTACTGTTGCTTTTATATGATTGCAGAAATACAGAATTTACCTCAAAAGACTTAATTCTGTTGCATCGCAGCATGCCAACATGGTTTCAGTTTTGGGAAGTGCAGATCCTAGCTACTTAAGAATGGGTTCTCTGAATAATATTGGAAATATCCCTTTTATAACTGGCTGTACTCAATTTTCAGATGCCCCGCTCAGATCCATCTCATCTGGTTCAGTGCTTACTAGACTGAACACTCCTTCTGGACTTGGGATGTGTGGGTTTGCCCCTTCTAGCATGATTCAGCTGGCCAATGCACCTAATTCTAGCAGTTCAATCACTAGCGAAATTAACTTTCGGCAGTCCATACAGCCTGGAAACCAAGATATGGATATTCTTGAAGGGATGCCAATGCCATTAGGAACTGATCAAGTGAACAATAATCTGGGAGTTACTCATCTTTATCCTTTTTCTAATGGTGTGCCAGAAAGAAAAATAGATGTTGATGGCAGAAGGAACTTGACTATTGGTGTTTCAGATAACTCTATAATTTTGAGGTCCCAAGGTCAATGTGTTCAAAGGAAAGATTTTCTTGACAATCAGTTCCCTGTTATAGCATCTCCAATGAGTTCAGCAAGTTCTCCCTTACTGAATACTACGAGATGTAATGACAATTGGCCAACAGCTAGTCAATCATCTCTTCTTGAAGCAAATTCTTTTGGTACTAGTGTCTACTCACACCACGCTATGCCCAGGGATTTGGGAAACAACGGATCTACTTTGGAGGTACCTATGTCTTCTAATTTACATAACCCTTTAAACTCAGCGTGTCCTCAAGTACCTGACACTAGAACAGAGATGCAATGCCTGACAACGATAATTGACAATGTTTCTGGAGTGAAGATGAATTTTAGCCCTCGACAAGATTGGCACGACTTTGAACCGGATTCAGCTCATGTTCCAAGCCTTGTATGCAGCTCCGCTCACACATTTCTTCCTCCAGATGGTGGCCAGAGGTTGGACTTCGAGAAACTCCATATATATAATAATGATGATCTTAGTTCTATGGTGTAAATGAATAGTGTTGGTTCTTTCTCCAGGCAGCAGCAGCATCATGAATTTGAAAACGCGGCTGTAGATATGAAACAAGAATATTTAGAGGAGCAAAAGATGCTGGCAGGGAATAATGCTTATGGCCAGATGGGATAG

>g16498

ATGACTGTGGAAGAAATAAGAGGACACATGGGGGGTGAAAAGGGAAATCATGACAGTTTCCCAGTGGGCATGAGAGTTCTTGCTGTTGATGATGACCCAATTTGCTTGAAGTTATTGGAAAGCCTGCTTAGGAAATGCCAGTATCATGGTATGATTTCTTCTATTTTTATGATTTTTTTTTTTATTGAATGATTGTAAGTTTTTGAGGCTTTTGGGGCATTGACTTAAGTTGTGTGAACTAATTTTGACTCTCAAGTAGATTTCAGAAGCCATTGATTTCCAGGGCTATTTGCTGATTCTTTAGACTTTTCTTCATAAATTTCACTTTCAGGATCTAGGTCTGCTTTCCTAATCCATGGGAAATTTAGATCTCACTTTTGATTCTTTATTTCATCATTTCCAGTGCATGTTGCATGGTTTGGGAAGAAACCAAAAAATAAAAAGATGGTGATTGTTGGTGGTGATGGTGGGGCCTGGTGGGATGGAAATTGACTGTAGCATTTCAGTTGTTTTTTTTTTGAAAACTTCAGTTGTTGTTAGTAATTCTCCTGAATTCAATTGGTCCTTGTGGTCCCTGTTGCATATGAGAAGATGAAAAAGAAAATTGGTTCTTGTAAATGCTTTCTCTTAGTCCAAATCCCAAGAATTCTTGTTTGTTTCTTTTAAGGTAGATCACTGCTATTGTTTGTACATATCTTATGTTGTTTGGAATTGTTCTATGATGTTCTGCTGGAACTTGAAACCTCTGCTATGCCCCCATGAGTTTTACCTGAATCTTTAACTGTCAGGGCACCTGCTTTTTAAAGGGTTTTAGATATTCCATAGCTATCTTTGATTTTGAGGTTTTGTCCCAGATTTCATCAAATCCAATGCCTGCAAAGTTATTTATAGCCTCAGGATTTCCCTAAAATGAATCTTTGTTTATTCGAAAATCTTTGAGAAATTTGCTGTTATCTTGAATACAATCTGTGTTTGCTTTTTCAGTAACTATAACAAGTCAGGCAAGAATGGCACTGAAGATGTTGAGAGAAAACAGAGAGAGATTTGACCTGGTGATCAGCGATGTTCATATGCCCGATATGGATGGTTTTAAGCTGCTAGAGCTCGTTGGTCTTGAGATGGATCTTCCTGTCATCAGTAAGCGTCTGAATTTCTTTGATTAAAAGCCTATTTTCGCCCGACATTTTGTTACCTCGTTTGAATTCAGTATCCGGATAGATCTAATTCTGTTAATGTTAATCTGGCATGTTTTGTGGGTGTAGATTTGCGAATTGACCCCATTCCTTTGAAATTTTTTTCAGTGTTGTCGGCGAATAGTGATACCAAACTTGTTATGAAGGGAATAACTCACGGTGCTTGTGACTATCTCGTGAAGCCTGTGCGGATTGAGGAGCTGAGGAACATATGGCAGCACGTAATCCGAAGAAAGAAGTTCGACTCCAAGAGCCAAAACAAGTCGGGTGATCAAGACAGATCTCCTAATGGGGGTGGAGAAGGCGGGCAGGGGGGTCCACTGTCGGGTTCCACAGACCAGAACGGGAAGCTCAACAAGAAAAGGAAGGACGAGGAAGACGAGAGCGACGAGAATGGTCACGAGAATGAAGACCCTGCAACGCAGAAGAAGCCTAGGGTTGTTTGGTCTATAGAACTCCACAGGAAGTTCGTTGCAGCCGTTAACCAGTTAGGCATTGAAAGTGAGTTCCCAAACGTCATTCCTTTATTTCGGATCTCATCTTATTCACATTATTTCATCCTCTCGTCCTCTAAATGCAGAAGCTGTACCTAAAAGGATTCTTGACCTGATGAACGTTGATGGGCTTACCCGAGAAAACGTGGCAAGCCATCTCCAGGTAATAATCTCCGCTCTTTAGTACTTTTTAAGTATTATTGTTGTTTTCTTTAAGTATTTGATGCATCGACAACTTTCATGATAGCTAATACCCGCAACTAATTAAAAGTGTGATCAATAATAATGGAGATTTCTTATTAAGATATACTATGTTTTTGGGCATTTTATAATAACGATACTGTGCTGCAGAAATATCGGCTCTATTTGAAAAGGATCAGTTCAGTTGCAACCCAACAGGCTAACATGGTGGCGGCCTTTGGGGGTAAGGATTCTGCTTATATGCGAATGGGTTCGCTAGATGGGCTTGGAGATTTTCGGACACTGGCTGGATCGGGAAGATTTAGCCATGCTTCTTTATCATCATATACGCCAGGTGGCATGCTCGGTAGACTTAATAGTGCTGCTGGTGTGAGCATCCGAAATCTTACCTCACCATCGTTGATCCAACCAAGTCACGGGCAAAATTTGGGCAAGCCCCTTGGTACTCTCGGGAAATTGACTCCGAATGTTCCAGCTGTGAGCCAGAACGCTTGTCTGTTTCAAGGAATTCCATCGTCGTTAGAGCTTGATCAGTTTCAACAAAGCGAAGGCACCCACACATACGGGGGGACCTCAATCCCTTGGATGATTCAACGCTCCTTGGAGCTGCTAATACATTCACGGACCCTGGATCGGGGATTGGTAGTTCGAGCAATCCTATGATGCTTCACGGGAATTCTCAACAAGGGCTAATGGCGGGAGGGATTGGAAACCAGCATTCCCTCAACATGGCTTCTTTAAATTCCGAACATTTTAATATTGGTGTTGGCGGTTCTTCCAATTTTCTTGACCATGGACGATCTAGCGACAACTGGCAGAACCCCATTCAGGTGACCAACTTTCAGTCGAGTTCTCTTCCATTGACTGAAACTTTCAATCAAGGCCAGATGCAACAAAATTGTGCGAGAGAAAATAATTCTTCGATCGGCCCTCATTTACAGGGCGGCTGTGCAGGTTATTCTTCCCTCGCCTCTACTGCCACGCCTTTCGAAGATTCAAGGGGAGAAATTCAACGACGGGAAAGGTTGGTAGGCGATGCTATCCCGAGTATTAACCAAGTACCTAGCCAACAATGGGGAGAACTTAAACAAAACCCTAACTCAAACGGCGTTTACAGCAACTTAACCGCTCAGGTTCCCGCCAGCAGCATCGTGCCTCCTTTAAGCCAGAGTATGGACCAATGCAATGACACCGGCAACAGAAGAATGGACGCTTCCAGTCAATCAAATCTCGGTTCTTCGGTTCTCTTGCAACAAAACAAGAACGAAAAGTTGACCTCAGAATCAAGGGCGAGATACTATGAAGACTACCTTTTTGAGCCCCCGAAGCCACAAGGCGCTTTTCCTTCTCAAGGTTACGGCTCTCTGGACGATCTGATGAGCTCAGTGATCAAGCGGGTATGACGTCTGTCTCGCCATATTCCTTTAGTTGATAAGTGTTCTTTCTATAGCTTTGAATACCATAGTGTGAACAACTCGTGTCGTTTTCTGCTTGTTGTTTGACTTGGATGGATTGCATCTATGTGTTTCGGTGAAGTATATAAATATAAAGTATGAAATTACATCTCTCGTGTCACATATGAAGTAACTGCAACAAAACGAAGAATCGCAACATCTAATGTACCAGATAACGAGAACTGATACAGACATTCAATTCTGATCTTACTTGTTGCGGTGCAGGAACAAGATGGAGCGACACTAGAAGGGGAATTCGGGTTTGATGCTTACTCGTTCGGGCCGTGTATATGA

>g47091

ATGACTGTGGAGGAAACTATGAGGAATATGGGGGTTGATAGGGAAAATTACCATAATTTCCCACTGGGTATGAGAGTTCTTGCTGTTGATGATGATCCTATTTGCTTGAAATTATTGGAAGGCCTGCTTAGAAAATGCCAGTATCATGGTATGTTTCTTCTCTTTTTTTTTTTTTACTAAAGTTTCCTATTTTCAGCTCTTCTGTTCCATTAAGATTTTGTTTTCCTATTCCTTATGTTTCCAACATTCATATAGGGAGGGGGAGGGCTAACTAATTATGTATTTGTGTGGTTGTTCATTTAAACTGCTGCAGGCTGAATCCAAACCCCAAATTTCCCTGTTTCTTGCTTGATCTCTCTCAAGTGCTTATTATGGTTCTTTATGATACCCAGGAAGTTTAAAAGATTTGATCTTTTGAGTGTTCTTAGCATACAAAAGTTTGGTTTTCTCAATTCTTTAAGCTGCTGATCTTTTGAAGTTGTCCTTAGGAATCATATGGTGTTTGGGCTTGATGTTTGTTGGCTTGTTGCCATGTTTTCACTTTGGGTTTCTTTGCAGTAACTACAACCAGTCAGGCAAGAATGGCACTGAATATGTTGAGAGAAAACAAAGACCGATTCGACCTGGTAATCAGTGATGTTCATATGCCTGATATGGATGGTTTTAAACTGCTTGAACTCGTGGGGCTCGAGATGGACCTTCCAGTCATCAGTAAGCATTTCTTTGGGCCAAATGAGATCACTTGGATCTAATTCTGTATTTTCTAACATATTCAGGTTTATGGTTTAATATCTTTGAATTTGTTCTCAGTGCTGTCAGCTAATAGTGATACCAAGCTTGTAATGAAGGGAATAACTCATGGTGCTTGTGACTATTTGGTGAAACCTGTGCGAATTGAGGAGCTGAGGAACATCTGGCAACACGTAATCAGGAGAAAAAAGTCGGAATCCAAGGGCCAAAATCAAGACAATGGTTATCGTGGAAATGGAGAAGGTGGGCAGGGGTTCCCACTGACAGGTTCCGCGGAACAGAATGCATTAGTCAACAAGAAAAGGAAGGATGAAGAGGACGAGACTAATGAGAACGAAGACCCATCCTCGCAGAAAAAGCCTCGTGTGGTTTGGTCTATAGAACTTCACAGGAAGTTCGTTGCAGCTGTTAACCAGTTGGGCATTGAAAGTGAGTTTCTCGACTTCTCAGATTCAAACATTCTCGTATTTCACAAATTTTCCCTTTATCATATCCTTCTTGTTCTTCAAATGCAGAAGCTGTACCTAAAAGGATTCTTGAACTGATGAATGTTGAAGGGCTTACTCGTGAAAATGTGGCAAGCCATCTACAGGTACAGTTAACTAATAAAGAATGCTTGTAAATGTATTCTTATCTGACTCCAAACAGGCATCAGAATTCAGATTGATAACTTATCATTAGCAAGCAAGAAATTGTTTTTAATAGAACCAAGATTTTCTCGTGAAGATAAATATATTAAAGTTATTATTGTTTGAGCATTTTTGTAATAAAACAAAACATTCTTGGTATTGAGTTGCAGAAATATAGGCTTTACTTGAAAAGGATCAGTTCAGTTGCAACCCAGCAGGCGAACATGGTGGCTGCTTTAAGGGGTAAAGACTCGGCTTTCATGAGAATGGCTTCACTAGATGGGCTTGGAGATTTTCAAGCATTGGGTGGACCTGGAAGATTTAATCATGCTACATTGTCTACATATACACCTGCTGACATGCTTGGTAGACTAAGTAGTGCCACTGGTGTAAGTATTCGCAACCTTAGTGCATCCGCCTTGGTCCAGTCAAATCACGCCCAAAATTTGGACAACTCCCTTGGTTCTGATGGGAATTTGAACCCAAACATTTCGCTTTCAAGCCACAATGCTGCTAGTTTGTTTCAAGGAATTCCATCGCCTTTAGCTGTACAGGAGCTGAACCCTTTGGAGAATTCTCGGGTGCTTACTGCAGCAAGTGCGTTTGCAGATTCTGGATCTGTTATTGGTAGCTCAACGAATCCTATGATGCTTCAAGGAAGCCCCAAGCAAGGGCTAATCGGGGGAGGATTTGGAAACCAACATTCTCTCAACATGGCTTCTTTAAGTTCTGAACTTTATAATACTGGTGTCAATAGTTCCTCCAATTTTCTTGGCCATGGCAAATCTAGTGAGAATTGGCAGACTTCTATTCAAGTTTCAGAATTCCAGTCTGGCTCTTATCCATTACCCGAACCATTCAGTCATAGCCAGTTGCCTCAAAATTGCGAGAGAGAACACGAGTCTTCAGCTGCCACACATTTACATAGTAGCCCTGTTGGCTTCTCTTCCACCACCTCAGCTTCTACAACTTTTGAAGATTCAAGAGAACCCCAAGTACCTAGCCAACTGTGGGGCGATGGTAAACAGAATCAGAACACAAACGACATTTTCAGAAACTTGAGCTCTCATGTTCCACCTTCTTTAAGCCAGGGCATGTACCAAATGAGTGGCAACTTAAATACAAAGATGAATTCATTTTTAATGCGTCGGTCAAATGCAGGATCTTTGGTTCTCTTTCACCAAAATGGGAATGAAATGCCAACCCCAGACCCCAGGACAAGATCCAACGAAGATAACCTTTTGGAGTCCACAAAGACACACGGGGCATTTGTATCTCAAGGCTTTGACGCCCTGGATGATCTAATGAATGCAGTGATTAAGCAGGTATGCCATATATGAACATTTTCTCATACTTAATGGTCTTAGCACATATATATATATATGCATTTTCAAGAGGGATAAGGTTACTGTCATCCTCTCTAATATGTTAACTGCTTGGATTAGTATAAGAAAACAAAAAGAAGAATACAAAGATTCAATTTTGATGGTAGTTGGGTTGATGCAGGAACAAGATGGAGGCATATTGGTTGGGGAATTTGGGTTTGATGCTTACCCATTTGGTTCTTGTATGTGA

>g22235

ATGACTGTAGAGCAGAGGAATGATAAGCAAAATGATCAGTTTCCACTGGGGATGAGAGTGTTGGCTGTGGATGACAACCCAACTTGCCTCATGGTGTTGGAAAATCTGCTTCGGAAATGCCAGTACCATGGTCTGTGTTCTTTCTTCTGTCCCATTTGTTCTGCCAGCAATACACATAGATAGATGCTTGTGTCTATATTGATATTGTGGGTGTGTGTGAGAGTGAATATCTGATTTGTTTTGTTTTTGTTTTTGTTTTCCTTTTTTCAGATTTCAGTTGTTTTGTTTGGGTTTTCCTTTCTCTATAATGAGTTGAAGTATATTTATGCATGTAGAAGAGAAACTATACCTCCAATCAGTGAGTGCCTGTTTTGTTAAAACTATTTAATCACATTGTTGATTTTATGATTCAGATTATTGATTCAATAGTTCGCTTTAAGTGTTTGAGAAAAAAAATATAGTTATGGATTGCTCAATGGTAGCCAATAATCCATATGCTATTTGGGTTTTAGCCATTGATTATTCACAATCTTCAATAATAAGCTTTACCTCTTTACTAAATGGGATGGGACCTATATTTTATTAGTTGTGGTATAAAGTTTGGATTTTCTTCTCATCTTGTGACTAGATATATTCACACATCATATGTGGTGACTGTTTGGTCAATGGAATGGGAGCAAAGGCACCACTTGTACTGATAGTCTGCTTCGTAGGGTGTAAATGTAAAATGCTGTACTTTGAAATGTTTTCATGTAATAAGATGTTGAAGTTTTGGTCACAGTTGCAGTTCTGCTCGAATCAGCTTCTGTAATAGACACGGTCTTGGTCATTAATTCTCTGTTCCTATTTGCAGTCACGACAACAAACCAGGCCATCCAGGCATTGCAATTATTACAGAGAACAAAATCAGTTTGATTTGGTCATTAGTGATGTTGATATGCCAGATATGGATGGTTTCAAGCTGCTAGAGCTTGTAGGGCTGGAGATGGATTTACCTGTCATAAGTGAGTGATAGAACCCTAACTCACGGTGACATTATTATGGAACATTTTCGTAATTTATAGCGTTCATATTGACATATACATTTTCATTAGATATGAACACTAACTTATGGTTATTCTTATTTTGTAGAACAAGGATGTTTTTTGAAACAGCTTTTATTTGAAAGAAGAGTTGAAATCAAAATATCTTATTTCCAATTTAATTGTCTATATTATTCAAACTTTAGAGCATGAACACAATATGTTTCACTTTAGTATTTGATAATCCTTGATTAATTGTTTTCAATTTCTGTGATTCTAGTGCTATCAGCATATGGTGATACGAACCTTGTGATGAAGGGTATCACCCACAGACTTGTGATTATTTGCTGAAGCCTGTCCGCATTGAAGAGCTAAAGAACATATGGCAACATGTACTAAGGAGAAAGAAGTTTGACTGCGAGGAGCAGAAAATTTCTAACAAACCTGATGGTGAGTCTGGTGAACTAGGCAGAGGGTTCAGAGGGATGGGAGAAACTGATCGGAATGGTAAGCCTACCAGGAAAAGGAAGGACCAGAGTGATGACGAAGATGAGGAGCTTGATGAAAACGGTGGGCGAAATGAGGACCCATCAGCTCAGAAAAACCTCGTGTTGTTTGGTCAGTAGAATTGCACCAAAATTTGTTGCTGCAGTTAATCACCTGGGAATTGATAGTAAGTTTTCATCTTCATCAAAAAATTTGGCAGGTCTTTCAGAGGGATCCTCTGTAATTTCACTTTATTAGTTAGCTTATCATTAACATGTTGCTCTTCACGACTTGATATTCTTTTATCCTTCTTTGACAACATCTGTTACTCAATTTGATGAAGAACATATGATAATTTTGAATGCATTGTTTCTGTACCATTATTTATTGAAATTTTCGTTCATCTTGGTATTGTAGTATAACTGACCCAGTATTTTTATCAAGTTCCGAGACTTGGATTTGTCATCTTTACTTTCTATCATTCCAACCATGCTCTATTTTCTTATAATATTCATAATTTTTTTTTTTAAATTGAAAAGCTTTAGGAGCATAGAGAATGCAGTTTCATCTACATATTCTCATTATTATGTTCTTATTTGCATTATAGAGGCTATGCCTAAAAGAATTTTAGAGTTGATGAATGTGGAGAAGCTGACCCGAGAAAATGTGGCAAGTCATCTCCAGGCAAATTCTGTGCCCTTGGAACTTTTTTGATGGACTGCTTTCACTTTTACTAGGCTTACTGTTGCTTTTATATGATTGCAGAAATACAGAATTTACCTCAAAAGACTTAATTCTGTTGCATCGCAGCATGCCAACATGGTTTCAGTTTTGGGAAGTGCAGATCCTAGCTACTTAAGAATGGGTTCTCTGAATAATATTGGAAATATCCCTTTTATAACTGGCTGTACTCAATTTTCAGATGCCCCGCTCAGATCCATCTCATCTGGTTCAGTGCTTACTAGACTGAACACTCCTTCTGGACTTGGGATGTGTGGGTTTGCCCCTTCTAGCATGATTCAGCTGGCCAATGCACCTAATTCTAGCAGTTCAATCACTAGCGAAATTAACTTTCGGCAGTCCATACAGCCTGGAAACCAAGATATGGATATTCTTGAAGGGATGCCAATGCCATTAGGAACTGATCAAGTGAACAATAATCTGGGAGTTACTCATCTTTATCCTTTTTCTAATGGTGTGCCAGAAAGAAAAATAGATGTTGATGGCAGAAGGAACTTGACTATTGGTGTTTCAGATAACTCTATAATTTTGAGGTCCCAAGGTCAATGTGTTCAAAGGAAAGATTTTCTTGACAATCAGTTCCCTGTTATAGCATCTCCAATGAGTTCAGCAAGTTCTCCCTTACTGAATACTACGAGATGTAATGACAATTGGCCAACAGCTAGTCAATCATCTCTTCTTGAAGCAAATTCTTTTGGTACTAGTGTCTACTCACACCACGCTATGCCCAGGGATTTGGGAAACAACGGATCTACTTTGGAGGTACCTATGTCTTCTAATTTACATAACCCTTTAAACTCAGCGTGTCCTCAAGTACCTGACACTAGAACAGAGATGCAATGCCTGACAACGATAATTGACAATGTTTCTGGAGTGAAGATGAATTTTAGCCCTCGACAAGATTGGCACGACTTTGAACCGGATTCAGCTCATGTTCCAAGCCTTGTATGCAGCTCCGCTCACACATTTCTTCCTCCAGATGGTGGCCAGAGGTTGGACTTCGAGAAACTCCATATATATAATAATGATGATCTTAGTTCTATGGTGTAAATGAATAGTGTTGGTTCTTTCTCCAGGCAGCAGCAGCATCATGAATTTGAAAACGCGGCTGTAGATATGAAACAAGAATATTTAGAGGAGCAAAAGATGCTGGCAGGGAATAATGCTTATGGCCAGATGGGATAG

>g49840

ATGAATGATAATGATGTGTGGGTTGAGGGGAATGATCTAACTTGATTACTTTCATCGATTTTTGCATCAAATAATTCTACCTTCATATATTCTTTTTTGAATGTCATAGTGTTCTTTCCCAAGGGTGAATGATACTAATGTGGTGTTAATCCCAAAGAAGGCTACCCCAGTGAAAGTGTCTGATTTACGCCCTATAGCCCTCTGTAATGTAGTCTATAAAATTATGGCTAAAATGATTGCTAATAGGATGAAACCTCTGTTGGGAGATATAATTTCAGAGTCACAGAGTGCTTTTATTCCGAATAGGCTCATTACTGACAATATTCTAATTGCTGCGGAGGTGGGACACAATTTGAATAGGAAGCAGTGTTTTGGTCGAAGTCGATATGGCAAAGGCATATGACCGGATGGAGTGGTCTTATTTGCGAAAGATGTTGTTAGCTCTGGGTTTTTCTGTGACTTGGGTAGACTTGGTTATGTTATGTGTGACCACAGTCTCCTATAATTTTCTTGTGAATGGTATAGATATTGGTCAGGTTATCCCCACGCGTGGCATTCGCCAAGGAGACCCCCTATCTCCCTATCTGTTTATTATTTGTGCAGAAGGACTCTCCCTACTACTCCAACAGGCTGAAACAAGGGGTGACTTTCATGGTCAGGGTTGCAAGAGTGCACCTCCCCTCTCATCTTTTTGCGATGATAGCCTCCTGTTCTTTAAAGCGAATCTTCAAGAGGCTGAGGCTATCAAGCAATGTCTTTATTATGAGCACATGTCGCCGGGTCAACTATCATAAATCCGTGTATGTTTTAGTAAAACACGACTTGAGCAGAGTTCATGTGGCTACGGTTTTGGGTGTAGTTCTCGCACCAAATTTTGGTAAATACCTAGGGCTACCATCGTTCGTAGGAAGAAATAAGAAAGTTGTGTTCTCGTACATTGAAGATAAGATCAAGCAAAGGATTGGGTCATGGAATAAGAAACTGCTATCACAGGCAGGAAAAGAAGTTCTACTCAAGAGTGTGGCCCAATCTATGCCTACATTCGCTATGAGTGTATTTTTACTTCCTGACTCAGTTTGTGTTGCTATTGAGAGGGCAATGAACCGTTATTGGTGGGGTTCGGGAATGAGAGAGCATACACCGGAAAGCTTGGGATAGTCGTACCCAAGAAATTTGGTGGTTTGGGTTTCAAGGACCGAGGGCTTTAATCTTATGCTAAAGTTGCTCGAGGTTCTTGGTAACCCCCAATCATGGTGGCTCGTGATTTATAAGGCCGATATTTTCCAAAGTCATCTTTTATTGATGCCACCGTAATTGCCGGTTATTGTTGAGGAACATTATGGCCTCCCATGAATTAATTTGTATGTGTAAGGAGGAGAGTGGGGAATGGAAATTCAACTCTAATCCGGGGTCATCCGTGGTTACCTGACGAACCTGATCCAATGATACACACTACCATGCCTCATAATTTAGTTGGCTCAGTGGTTTCAGGCTTGATTGACCCGAGCACAGGAACATGGGACCATTCCATCATTCAGGATATTTTTCAACATGATGACGTAGCCCGTATATTGAAGGTGCCAGTGGCTCCCCTCTATGAGGATTCCTAGTTCTGGCAAGGAGACCCAAAGGGCATCTATTCGGTTAAAGAAGGCTATAAGCGAGTTGTTGGAGACCTACCGCCTACTACGGGTTTTTATGATAAATGGCTACATCTTTGGAAAATAAAATGCCCTGCCAAGTGGAAAGTTTTTATATGGAGAGCTCTTTCCAATATTCTTCCAACCACTACGAATTTGATCATAAAAAGAGTGGAGATTGACCCACATGTCCACTGTGTGGTGTTTTAAATGAAAATGTTATGCATTCACTTCTTTTATGTGATTTTTCAGCATTAGTATGGAATGAATCATTGCATGTTGCTAGTGTGGGGAAGATTTTGGTGTGTGGTTTGCTAATGTGTTTTCCATATTAACGGAGGAAGATGTTGTGACTGTTGTAGCAACCTTGTACCATATCTGGAAGGTGCGAGCGCTTTGGGAAGCTTCCCGAGATCGTGGGCGCATAACGCAGCGGCCGTCCGCCCCATCCGCTTCGCTCATCTTTAGGCGCCACTACCTACGGTCAGTTTACAAGGCTTTGGAGCAAGTTATCGCTGTTATGTAGACGCGGCTTACCTTCACCAAGACGGAGCAGCTAAAACGGACCTTCATGGCGGTACCTTCAACGGTGGTTTTGTTTTTCACGCTCATGGCCGAAGTCGCTAGCATGCAAAGAAGCTCTATCATGGCTAAAGGACCTGGTCTTACTGTGCACATCTACACGCATTGTTCCACGCTCAAGAATTTGTTATCTACATCTTCTGGTAATCTTTTGTCATATGTTGGTTTTTCTATTGATGCCTCTAGGGCACTTATGTCGTCTTTTGATCATTGTTCAGTTAGTTTCATTCCTAGAACTGCAAATAGAGGAGCTCACGCTGGCTAACGCTTGGTCTCTCAGCTTCTTCTTTGTTTTGGGATAATAATCCCCCTGACTCTATTTTTGAGCTTATTTAATAAAGTCACCTATTGGTTTTCAATATATATATATATATATATATATATATATATATATATATATATATATATATATATATATATATATATATATATATATATATATATCATAAGATAAGTTTAATGACATGAGGTCTTTAAAAAAATCAAATGGAGTATTTGATTTTCAATAGGAGAAAAATTAGCCAAATTGTTAGCATGCATTATCTTTAAGGATATTAATGATTAATAATCGTTAACATCCCTCAGGAACATATATTCTATTCATTATCTATTGATCTAATTTCTCATATAAAATAACCCTAAACTCATCATTTGCCACATTTAGTTCATAATAATATGAACAAAGTGTTTTCTAATCCGCAATTTCCAGCTGGTGTTAGTGTTCTTCTTGTTGACGATGATGCTACCTGTCTACGAATACTAGAAGCTTTGCTACTCGCATGCGAATACAAAGGTGCTTTTCTTCTTTCTTTAATTTCTACTCCATTTCCTAGCTAAAAGATAGGACATGCCGTTTGCCTTGCCTGCAGTGGTGAAATGTAGAGGGGCTATAGATGCTTTGAGAATCCTTCAAGAGGGTAAAGAGGAGATCGACATTGTTTTAAGCGAGCTACACATGTCTCGCGTCAATGGATTCAAGCTTCTCAATCAAATCATTGGACTGCAAATTGACCTGCCTGTTGTGAGTAAGCACTTTCAATTTCATCTCATTGCTCAATCTATAAGAACTTTGAAAATTCAATCGGTAAGCACTCACACTGCTCACCTTAAAAGGATTGTTCGTCAATTAAAGCGGTACAGAACTGTGTTCAAAACGTCATGAGACAGTTTGGTCAATTTGGTCCATATTCACCTATTCGGTGTGCATATTAGAACATTGAGAGGACCGGAAAGGTCCCACCTTGGTGTACCAGTTATCGCGCCCATAATAAATGCTAGGTAGCCAGAACGACTGAAAGCATCTAAGTAGTAAATCCATCCCAACTTATTTTCTTATCTATAGACCACCATTACATTGTTTGAGACAAGCCAGGAACAGTCTCAGGCCCTTAGTACTGAGAATTAATTGGCTTAATTGATGGTATCAGTGATGTCGAGTGACGAGAGAGTAGATGCAATAAAGCAGATAGTGATCCAGGGGGCATGTGGCTACTTACTAAAGCCGGTGAGGAAAGAAGAAATCAAGGTCTTATGGCAGCATGTTGTTCGTCACAAACAGGGGAATTTGGGGAAGGGCATAAGGCCGCCGCAGGCGGCGGCGTTTTGGGATTCCGGTGAGATGCCGCAGCAGAAAAGTGTAGAAAACTGTGGAAGCTCAAACAATAATAGGGATGAAGATACTAATGCTACTGCTACTACGGCCAATGTTAAGAAACCGCGATTGGTGTGGACACCACAGCTTCACCAGCAATTTGTGGCTGTCGTTAACCAAATTGGTCTTAGAAGTATGAATCTATTGGCCTTTAGTTTTCTTGCATTCATATTTTCTATTTCTGATCTGTAATTAATTAGTTTGCTTCACCTATTGGGTTAGATGCTGTTCCAAAGAAAATATTGGATTTGATGAACGTTCCCCATCTAAGCAGAGAAAATGTTGCCAGTCATCTCCAGGTACATCTGTATATCCTACCTTACTTGATAGAATTGAATTGAATAAGCTAAGACTATCTCCAGCTTTGAAAGTGCAATTATTGTAAGACACAGCAGAATTGCAACACTTGCATTGGTGTGAAATAGAGAATTGTATGAAAGAAAAATGAATAGTAATTAATTGTAAATTTAATGAAATAAGATTTTGAAAGTGAGATAATTTACTAAAACTAATAATGTAGAAATATCGGCTTCATCTACAAAGAAATGGGGATCAGAATTCATACAAGAGGTTGAGCATTCATCAACTCCATTATGAAGACATGGTCTTGAACAAGAACAATGAACAACAAGTGATGCCAGCGACCGGTGACCATAACTATGTTAATGTTTACAACTATGGCAGCCTAGGCCGAGGGAGTAATGTTTACAACTATGGCGGCCTAGGCCGAAGAGTAGGAGGGAGTACAACTACCAACTACTTTTCGGCTTTTCCGCCCTATCAACCCCAATCCACCGGGTATACATACAATAATAATAATCCCAACCAGTTGGGAAACACTTCCTCTGCTCCCAGCCAGCGCCAACAAGATGAGACTCAGGTTTGTACTTATTACTTTTAAACCATTTCAACCTTTTGCACTTAGTTACTTTTAAACCATTTCGACCTTACTAAATAAAATTTTACTTTTAAACCACTCCACCCTTACTAAATAAATTGTCGCCTAACTTATTACTTTTGTGCCATTGACCTATTGAAAATTATATTAGACTACTTTTGTCTAATTGAGCTATTAAAAATTATATTAGATTACTTTTGTCTAATTGAGCTATTGAAAATTATATTATATAAAAAAATATATGTGCAATTGTAATTGCTTCATTTATAGAAGACATAAAAATTTAGGCAAAACAAAATAGTAACACTCATTTCAAAATATTGAAGAATTTTATATCTGCTCATTATTTTTCTATTTTGTGTGTTGCAGTACAATGGTGAAGGAGATTTTGGAGTCTCTTTGTACGATTTTTCTGGCACGGATAGTAATTACACATAACTTGTATGTATGATATACAGATACTTACACCTATAAGAACTTAATATTTAATTTAGTGATATTATACATTCTTATAAATGGAATTGAGAATTCTAAAGAGCTGTCATATTGTTAACAATTTTTATAAAGTTGAACAGAAATTCTTTGTACAACAAAATTCTTTGTACAATTTTGTTGAAAACATTGTCTCTAACATTGTCCCTTATGTATTAATCTCAAAAGAAATATATTACTTAAATTATTAATATATATTATTAATTTTATCATTATTATGAAAATTAATATTCATTTATTATTATTTGTATATACTATTTACTATTTATTATGAATTTTTTTATAGAAATTATTATTGGTTATAGAAATAATAGTTTGATAATTTTTAACATGTATTTTTTTTACATTATTACTCTAATATATATATATAGAGAGAGGGGGAGGTAAGTAAAAATTGATATGAACCATCCATTTAGGTAGATCATGATATAATGTAATTAGGAAAAGTAAAAGATTTTAAAATGTTTTTACAAATGTACAACATTATAAACTATTATGAAAACTCTTAGAGCATCACCATCACTATTATGAAAACTCTTAGAGCATCACCATCAGAGAAGCTTGAGCAAATAATGTCAGAAAAAGTAAGAGAGAAATTGAAATGGGTTCATCCGCAAATGAGTTGTTTTCTGACAAAAGGGATTAAGGCCAGTCTTGCCTGTTTGACAAATAAGCGCGCCCGACACCTTCATATTTTTATTATTATTTTTTAAAATTTGATTTGTTTTTTTTCTCTCATTTTCTCTTTCCTAATCACACTTACAAAAACTCCTCCTCAATAACCGCGAAAAAATTCCATTGATAAGGATGCTCTTAAAAACATTTTGAGAATTTGTTATTTGGATTTAAATTTGAGTTGTATAAGTATTATATAAAATCTAAAAAAATATTATTTTCATTCCAAAATTTTTGCTAATATTTCATGATGACGTGGCTTATAATTAATAAGAATATTGTTGTTTTAATAGTTAGTACGAAATCTCTATTATGTTGCAGCCTCTAAGAGGTTATAAGTATGTAATTAAAGTAAGATACACATATTGGTGAGGGCTCTTTGTGCGTAATAAGCAGAGGTCTAGCATCTGTGTTCAGTTGAGTTAGGAAGAATGGAAAGAAGCAAATAATTGAAATGTGTAATTCGGTAAGAAAAGATTAAAGAATATAAAACAAAGATGTATCAGATTAACAATGTTACCAACTCATCATTAGAGAAGAGTAAGGATCCACCAAATTTACTCCAATCCGTTAAATCTGTTTCGATCCAATTTGAATTCACTTAGGGTATGTTTGGAAAGAAGGAAAATGACTTCTAGAAAATATTTTCTAGAAAATGAGTCATTTTCTAGAAATATTTTTCTTTCCGTGTTTAGTTGCATTTCAGAAAACTGTTTTGATGTGTTTGGTTCATTTTCTAGAAAATGAAAAAAAATTGTAATCTAAAAATTAAAATTATATAAAAATTACAAACATTCTATCAATTATTATCATCAAATAAATTAGTGTCATTATAAAATAAATAAATAAAAAATAAAAAACTCTCTCTAGTTTAACTAGAGAGATCGAGAAGAGTCACTGGTTTCCATCTGGTAACCAGAGAGGTTAGACTCCTCTCTGGTTTTCGGTCGGAAACCAGAGAAGAGTCTCTAGTTTCCAATTGGAAACCAGAGAGTCTCTAGTTTCCCTCTCTGTGTTCCGGTTGAAAATTAGAGAGTCGAGATTCTCTGTCTTCCGATCGAAAATCAAAGATGGTGATTTTCAATCGGAAAAGATGATTTTTATGATTTCCAATGGAGGCTGTCGACTTGAAGATGAAGAAGGCCCTTGGTTTGTTGCGATTGTGTAGCAAGAAGAGTGAAGATGGAGAAGGAGGGAAGAGAAAAACCCTATGCACCAAGTCTGGAAAATGACTTCCCCAAATTTTGGGAAGTCATTTTCTAGAAAATGAGCCTCATTTTCCTTTGACCCAAAGCTATTTTTCTTTGACTTTTGTTTTCTATGTCTTGCCAAACACTAGAAGCCCGAAAAATGATTTTCATAAATCATTTTTCGGGCTTCCAAACACACCCTTAGCTTAAAGTGGATGGAATATGGATGCTTCTAAAAAATCTATTATATTTAATATGCTCCATTAGGTTTGATTTTGCACCATAACGTGTTTAGAAATGCACCAAGGTGTGGTGCATTTTTGTGGTGCATTTGTGCATTCCACTCTGGTGCATTTCTGAACACGTGGTGGTGTATGACAACACGTCCTCATGGGAAGGCATGCTCACATTTGATTCAGACGTCTGTGTGGGTAATCATTGAATATGTAAGCCTTGGAACTTTTCTAATAAAGATTAGGAAATATTTTGTGTTGTAATAAGAAATTGGTTCCTAATTTGTTATTCCAAAAAAAAATATTTGAAATCAAGGATATTTAGAGATTTTTGTGAATGAAATGCTTGAAATTGATTATTTTGACTTTTTTAATATTTGAAATCTAGTATTATAAATTATTTTATTTTTTAACTTAAAATTCGTCATTCAATATGCATCCAATCCGTTATTAGCGGATCGAATCCGTACTCTTTTTAGTGGTAAGTTAGCTTTGGTGACTTGAAAATGGATAGCATATATAATAAACAACAGAATAACTAAAGGACATAATACATAACTTATTTTCTCATATCAATAGGCCACTGTCACATTGTTTGAGACAAGCTAGAGCTTGTCTCGGACCCTTAGGGACTTTATCCGCAAAGTCCGCTTCGGCCCAACAATATTTAGTGCTGAAAATTAATGAGCTTAATTAGTTAATTGATGGTATCAGTGATGTCGAGTGACGGGAGAGTAGATGCAGTAAAGAAATTATGAACCACTAGTTCAAATTAAAAGATCCAGGGGCGTGTGGCTACTTACTAAAGCCGGTGAGGAAAGAAGAAATCAAGCTCTTATGGCAGCATGTTGTTCGTCACAAACAGGGGAATTGGGGGAAGGGCGACATAATAAGGCGGCCGCAGGCGGCGGCGTTTTGGGATTCCGGCGAGATGCCGCGGCAGCAGAAAAGTGTAGAAAACTGTGGAAGCTCAAACAATAATAGGGATGAAGATACTAATGCTACTGCTACTACGGCCAATGTTAAGAAACCGCGATTGGTGTGGACACCACAGCTTCACCAGCAATTTGTGGCTGTTGTTAACCAAATTGGCCTTAGAAGTATGAATCTATTGGCCTTTAGTTTTCTTGCATTCATATTTTTTTTATTCCTTCCGATCTGTACGTAACTAATTAGTTCGCTTCACCTATTGGGTTAGATGCTGTTCCAAAGAAAATATTGGATTTGATGAACGTTCCCATCTAA

>g20548

ATGGAGAGCCTGAAAGTCCTTGTTGTTGACGATGATTCCACCTGCTTAGCCATTGTTGCAGCCTTGCTCGTGAAAATGGAGTTTCAAGGTATGGTTTCATGGCTTTTTCAATTCTCTCCGATCCGTTCAAGTTCCGTGCATGGTCCGTGTACTTACCGTGCTTCTCCGGTCATTTTCTCCGATTTGATTTTGGGTAAACTTGCTTTGATCTTCGGAAATGGTGAATTAGGGTTTTTTCTCCCACCCTCCCTTTCTTGTTTTTCGAATGTAAAAGTCGTCACAATTTTTTGGGGGAGGAATTCCGGTCGGGTTCCGTGCACCTGCCGTGCGATTCGTGGTTCCATTCCTCAATCGGCTGCAAATTTGTTTTCCTCAACTATTTTCAGTCCAATTAGCGTAATCTTAGGTTTTTAGTTTTTATAGCATAATCCTAGGTTTTTCATATCATGTTGGCCCGGTCGGTTTGACACGTGTGCCCTAAAAATTTTAGGCTTTATTTTTATTTGGGGATTTATTTGTACTGATTAAATTAATTTATTTGGTTTAGTTGTGGCTTTGAAGAACGGCAACGATGCTATTGAGGCCTTACGATCTCAGGGAGGCTTTGATGTGGTCATCTCCGACGTGCATATGCCGGGCATGAATGGATTTGAACTCCAACAACTGATTTTTAAACAATTTCGGATTCCTGTAGTTTGTGAGTTTAATTTTTTGGTCTCATTTTCGTTGTTAATTTGGTAAAAAATCATAAATTAATTCGGTATATGCATGCATGACATGTTTGTGCGAAAGTTGATCAATTAAGAAATGACTAATGCAGTGATGTCCGGTGACTGCGAAGAGGGAATTGTGAGACAAGCAATGCAGAACGGAGCTATTTCGTTCATCAGAAAACCGGTTTCACCTAATGATCTGAGAGGGATATGGCAATATATCATTGCCCAGAAGAGGAGCAAAGTCACAACGGAGCAGGTGAATACCGGTGACCAGGATAACGTTGATATTGGTACGAGTTCTAGCGGGACACGCTTCATATTCCGGTTATGATACCACATCGAAGAAGAAGATGGTGTGGACAGATCAAATCCATTTCATATTCTTGGATGCCATCTCAAGTTTGGGACCGGAAAGTAAGATTATTCTCTCTTTTGTTTCCTTATTTTTTTTTCTATTAATTTCAATAAAGAATTGTGGAGTAGTAATTTCCCATTTTATTAATTTTCCAAAATTTTTCTTATTACATGCAGATGCTACGCCAAAGAATATATTGAAGGCCATGAATGTGCCTGGATTAACAAGAGAAAATGTTGGCAGTCATTTGCAGGTCCACTCCTAATCACTAATTATATTTTTAATTAGGATTGGAACATAATTTTTTTTTAAAATAAAATTCCTAAAAGAGTGATTAAAAACTTGAATGTTGTGTAAAGATTTTGAGTAATGAAAGATAAGCTAACTACATAAACTTCAACACTCATATTAAATTATTTATTTATTAACGACTATATATTAATTATCAAATCTAATCTGTTCAAATTAAATATCCTCATATCTTTAATAATTATCAGATCTTGTTCATATTAATTATTTATTGGCTATTATAAATTCATCATAAGATTTCGTTTATAATACTTGTGCATCTATTTTTTTTTATTGTAAATTAATCATAAAATGTCCTTTACAGAAGTATCGACAATTTCTTCGTCGGAATATTCAAGATATTGAGAACAGAAACAATGGTGGTGGTGGCAATCAACCCAGATTTGGATCAAGAAAATTTCGCAGACTTGCCAAGCTTGAAGAAATGTTTGCCCAAAGTGGACTAGGTGCCGCATTATCGTCGCCGACATCGTCGGCGGCGTTGGGCCGGAGCAGTGCCGGCGGAGATGCAAGAACTCGCGGCGGCCGTGGTGGTTCATCGGAAAGAGGTCAGCAAGGTGTGCTTTTGTCGGAGGACGATGAATCAATGTTTTATCGGGGTTTGAGAAGGGGTCCGATGATTACTAGCCCTAATGAGTCTTCGCCGGCGAGTCATGAGGTGAATCCCGGCGGTTCACAAGAGTCCGGCCCGTATCCTACTCTAACGGCGTTAATGCAGAACCGTTTGGATTCCAGCAGCAACGACAACGTTAACGTTTCGGGTTTCGGAAACGTTAACGTTGTTTCAGGGAACGAAGACGTGCAAGTTCCGGGTGAAGGGAACTTTGATATTTATGACGCTATCTTGAACTTCATCGGAGAAACTGAGGACGGCGGCGACGAAAACATATCCATGTATGGAAACATCACCGCCGGACAAGATAATTCAGGCTACAACCAAATCGGAAATCTCTCAAACGATTTCCTCAATCAAGTAAACATTAATCAGACATACACTCCCAATCAGGTATTATCTATTGACCATATAATATACAAAATTAAATATGCAATTCAACTATCAGTTTTTTAGTTAAGATGAACACATGTTTCAATTTTATCATATTCCATCTCAATCAATAATTTTATTATTATTATTATTATTATTATTAATTTATTATGTATTTAATGTGCAGCTTATGTGCAACCAATATGGACAAAATTATAACTCTGGGCAGTGGTACGACTATAATCCATGGTATCAGCCGGAGACATCTCAACCAAATTACAACCACCAATACTCCTTCCAACCGCAGGCATGCCTGATCTTTAAACATTCATTTTTAGACCAAATTTAGGTTAAACATCGTACTCAAATTTCATAATAACAAGACTATATTATCTTGGTTTTGGTTTTGTTGTAGGGTGACGGCGATGATGATGACGACGCCTTTGTGAATTCTTTGTATGGACCAAACCCTTATGGAGGAGCTGCTGGAAATGGTGAAGGCATGTGA

>g61530

ATGTGGAGATTACCTGAATATCATATTGACAACTCTTATGTTAACATTTCAATGGCTTCATATCATAGGGTAACATATGGCATACATGTCCTCCTGGCTGATCATGACCACAAGTTTCTTGCATCAACTGTTGATATGCTCAAGCGACAATTTTACAAAGGTATAGATGCTTTATTGATGAATTTCAATATTTTTTTTTTGGAAACTATGAAAAAAAATATTTTATAGAGAGATTATTATATTCTCTTGAAGTAAAGTTATCATCCCGCGTTCGTGATTTTCTTCCATTAACTTCATATCTAATTTTGACGACGAAAATCTTCCCTTGATATATATGTCGTCATATGCCTGTTTATTTTGAATACTAGTTTCTCTCAGAGACTTGTTTCATTATTAATTTTGTTATGAATTAATCTATAGTTAAATTCGCCAATTTTTATGTGTTTTTTTGTTGTGCATGAGAATCAATTTTATCAACATTTTTCGTTTGGCTCTACCGAAATTTTTCTATAATTGAATCTTTGATTTTTGAGCCGCTAATAATATCATTTTATTTTTTCAAAGAGTCAAAAAATTTTAATTAAATTTTGCTAAAAGTAAATACGGAAATAATAAAATCTTCCCTTGATATATATGTCGTCATATATCTGTTTATTTTGAACACTGATTTCTCTCAGAGACATGTTATTCCATCATTAATTTAGTTACGAATTAATATAAAGTGAAACTCGTCCAATTTTATGTGTTTTTTTGTTGTGCATGATAATCAATTTTATCAACATTTTTCGTTTGGCTCTACCGAAATTTTTCTATAATTGAATCTTTGATTTTTGAGCCGCTAATAATATCATTTTATTTTTTCAAAGAGTCAAAAAAATTTAATTAAATTTTGCTAAAAGTAAATACGGAAATAATAAAATCTTTATATATGTCGTCATATATCTGTTTATTTTGAACACTGATTTCTCTCAAGAGACATGTTATTCCATCATTAATTTAGTTACGAATTAATCTAAAGTGAAATTAGTCAACTTTTATGTGTTTTCTTGTTGTGCATGATAATCAATTTTATCAACATTTTTCGTTTGGTTCTACCAAAAAATTTTCTATAATTGAATCTTTGATTTCTGAGCCACTAATAATATAATTTTATTTTTTCAAAGAGCCAAAAAATTTTAATTAAATTTTGTTAAAAATAAATACGGACATAATAAAATCTTCCCTTGATATATATGACGTCATATATCTATTTATTTTGAACACTGATTTTTCTCAGGGACATGTTATTCCATCATTAATTTAGTTACGAATTAATCTGTTTTCAAAAAAAAAAAAAGTTACGAATTAATCTAAAGTGAAATTCGTCAATTTTTATGTGTTTTCTTGTTGTGCATGATAATCAATTTTATCAACGTACATTTTTTGGTTGGCTCTACCAAATTTTTTCTATAATTGAATCTTTGATTTTTGTGCTGCTAATAATATAATTTTATTTTTCCAAAGAGTCAAAAATTTTTAATTAAAATTTGTTAAAAGTAAATACGGAAATAATAAAATTTCCCCTCATATCATTCGTGTGGTGGTGTTAGGTTTTTTATTTTTTATTTTTAAATTTTTATTTATATATAATCTTTATATATGCGATGTTGGCTACATTTAATTCATATAATTACTTAATTTCCTCTTATAAAAAAAAATTAATTAGATTATTATTTCCTGTTAAGATGTTTTCCTAATTAGTGTGGGGCCCTATCTGGGACAACCACTTGCCATAATTTTTATTAATATATTCTAAATCTCAATTTGGAAAATATTAAAATGAATTAATGACCGTTTTTTCTTTCGATTATACCGTAATTATTAAATTAGATCTCAATTATCAATTTTAACCAATTAAATTCTTAATTATATATTGATTTCATAACCGATTTAGTCCTACATAATTACCAAATTTGACGAAGGACCAAATCGGTTACGAAATTAATAGTTGAGGATTTAATTGGTCAAAATTAATAATCGAGAACCAATTTGATAATTACAAAATAGTCGAGGGACCAAAATGATCATTAATTAATTCTATTAGATTCGGTCCCCATTTCAATTTTGTATTGATAGTTTTAATTTTTTATTTGTGCTCTTTCACTATTACATTTTTACACTCTGATTTTCGAGTTTAAAATAGATTGAATATGATTTTTAATTATTAAAAATGTATTATTACTTTTATCTTCAAAAAAAATGTATTATTACTTTTAATCCTAATATAGAAGTATAGGACTAAAGGTGTCACATTTTATTAGTTGAAGGCTTGACCATATCTGGTTAAGATTAATTTTAATTTTAATGATAACAAGTAAGAAAAAAAATGACTAGAACTCTAATTAACTATTTTAGATTTGGAGGACTAAAATGTAATTTTGGTCCCTCAAATATTATATTTCCTTTCCTTTAGGGTTTTAAGTAAAATCAATCAAATTTGACTCTTCAGTATTGAAAACAGTTGTTATACCGTTGACCATGGTCCACACAACGTTGTGTGGACTATGATCATCGAATAAAAATATAGTAAAATTAAAATAGTACTTTAGTGCAACTAGTGTGTGAAAATTAAACTATAAAACAGAGCACATTATTCAAGTTTCTAAACCTACCCTTAACTAAAATTTTCTAGTTCCAATTCCTCTGGGGACGGGTTTGAACCAAACAGGTGAAATATCTAATCTCTACCGTAACCAATTTGAACTAAAGATCACATATGAATTTCAAGGAAAAACAATGTTGTGAGTAAAGTTTTCACAAATTTGCAATTGATTTCATAATTTTTGTGTTTGTAGATTAAGTAGATAATATAGTTTGAAATACATATATATTGGTGCGTGCGTGCAGTTACCGTGGTGGATTCTGCTAATGCTGCAATTTCAGTCCTTAACCGGAAAGAAGAGAAATTCGATGCGGTGATTGCGAACATTCATTCACCTGATAGGCAAGCGTATAGGCTTCTCCGCGAGGCTGTCAGCATGGATTTGCTAGTGATCTGTAAGTAACTAATTTCTTCGAATTTAATTTTTTTAATTATTGTTATTTCTTTGAATTTGAATTGAATAATATAATGTATATGTATATGTTCTTGGCCTGGATCATATCCCTGGAATTGTTGTAATGCAGTTTTGTGTGATGAGGAAGATGCTGAGATGGCGGTTAGGGTTATAGAGCACGGAGCATTCGCCCTCCTGCAAAAGCCAACGTGTCAGGAGACGTTGAAAAACTTGTGGCAGCATGTTGTGAGGGAAAGGAGTATGCTGAGAGCAAAGCAGATGATATTTATGGAGAAAACTAACAGGGAATTAGCTGTGATCAACAATGCTGTGATCAACAATGGCGTCGTCGGGGGCGGGGGCGATAATAGGGGTAAGGGAGTGATGAGGGTGGAAGAAAATGAGAACTACCAGATGAGCTACAGGGGAAAGGGGAAGAGGAGTCGGGAGCAGAGCTTGAGCGAGGCGACTCGGATGACGACGACGATGAGTCAGGGCATGTCTAGGGTTAAACGTAAGACGTGCACGGAATGGACTGTGGATCTCCATGAGAAGTTTATGAGTGCTGTTCATCAGCTTGGAGATGGAAGTATGTTTAATTAATTAATGTTTCAATCTCTATCTCTCTGTTTAATTTATTTATACATGATCGAATTGAATTGGTGGATATCATATCGCTTGAATCTTCATGAGTGCTGTTCATCAGCTTGGAGATTGAAGTATGTTTAATTAATTAATGTTTCAATCTCGATCTCTGTTTAATTTATTTATACATGATCGAATTGAATTGCTGGATATATATCATGAGTGCTGTTCATTAATTAGCTTGGAGATGGAAGTAAATATATATAGATGTTTCAATCTCTGATATGATCTGTTTAATTGCTTGCTGGATATCGGTTGAATGTTTAATTTGTGTTTGATTGAATTAGGGTGTTATCCGAAGGAGATTCTGGAGCTGATGAATGTGCCTGGGCTGACAAGGATGCAAGTGGCAAGCCATTTGCAAAAATGCCGCAATGATAACTGGAGAGCTCCTGAGGAGAGAAGAGCTCCGCCGATGTCCAGCGCGTCGCCGGCCAGTGGATCCGGGTCCAGAAATGAGCAGCGGAGGTTCGGGACGATGCCTAAGTTAACCGTAGCTGCTGCGGCTGCGGCGGCGGCGGCAGGAGGGAACTCCCAACAGCTGGGAAGCATTGCGTCGCCGGAAGTTCAGTCCTCGCCGTCCATCACCGGTGCGGTGACAGGAGACCCCAGCTCCCAGCCCCCCGCGGACCGCCAGTATCTGGCCATCGGAGCCGCCGCCGCCTTTGTCTCCAAGCTGGAATCCTCTTCTCCCCCGAGCGCCGCCGTGCAGCCCGCGGTCGTCGCCACCGCCGTCGGCACCTTCACCGCAACTTCCGGCCAGGCCTCCATTGTTGGGAGCGTTAATTACGGCACCGGAGGAATTCTGCAAGCACTCGGCTGCGGCGTTGGTGGTAGTGGTGGGAATTTGTTCCGCAACAAAGATGCTTTCACTGATGACCCCAACAACAACACCACCGCCGGTGACGACTCTTTCGTCACTCCCCAGCCGCCCCGCATCCACCGCAGGCTTCAGTCGGCCGATGAATTCTTCAGCTTCAACGACGTGGACTATGAATATCTGATTCAAGGTTTCTCCGACAACAATGCTCGCCAGGCAGGCGTTGCGCTGCAGGCCCCTACCCACAACAACACATCCTCCTCTGAGTTCAACGACAAAGCAGGTTTCAACCCCGTGCAGGTCAAGAGCGAAACACTTATAATTCTTTACAATCTGTTTATATATATTTCAATTCAAATTTTAAAGTAAGATATAGTGATTAATAGATGCTATATTTGTGGCAGAACCAGGAAGCAAAGGCACAAACTTCGGAGATGGACATTGCGAAGATCAGTCCTTAG

>g38332

ATGCTTTGCACTGCAAATGATTTATTGGGTTGGAAGGATTTTCCCAAGGGGCTTAGTGTTCTCCTCCTTGATGAGGACAGCAATTCTGCTGCTAAGATGAGAACGAAACTCGAGGAAATGGACTATATAGGTAATAGCATAATTTCCGATTTTCTTAATCTAGAGATACTTAACCTTTGCCAAGACCTTGTGGTCTAGTGGCACCAAGTTGTGCTCCTATGGGAGGTTGTGAGTCCGAGCCTCAGTAGAGCGGTATTGACTCTTTGTGCTTCAGTAGGTTGAGAAAGTAGTTATGAGCAGAATGCGTTGTAGAGTAGTAAAAAAAAAAAAAAAGAGATAATTTTCTCCAGCTTTCTGGAAATGAGGCCAAAACGATTAAATGGCTTTGTTAACTTTTGTAGTTTTCATAATGTTGCTACTATTAGCCTTAATCGGGATAGTAAAGATAGTCCGAGTTCTAGGATGCCATTTTCTATTTGGGTAGAATGACATCTTTCCCTGTCACTGCCTGTCTGTCTGTCCTATAATTCTGCAAGTTGGTACTGTCTTGTGTTTTGAATTGTTGTGGGGTTTTTCAGTTTTCCTAACTGAAAATTTGAATCAGTTGAACCAGTGTATGTTCTGTTACTCGAGATTTATTCAAGAGAAGTAACATGTTTATGAAGTAATAAGTGAACTATAACTTTGCCATCGTTACGATTTCCTTAAAAATTGTAGTACACTGCAAAAATGAGCATTCGATGCAGCCTTTTCTTTGTATACGACTGACATCAATATCCCCGATAATCCTTCTTGCAGTTTCAACGTTCCAAAACGAGAACGAAGCACTGTTAGCAATCGCAAGCAAATCGGTGGAATTTCACGTTGCTATTGTGGAGGTACTAATTTTTTACTCACTCCAAAGTTTTGTATAATTGCAAAATCAAAACCTGTGTGATTTCGATATTCACTTTCAAAAAGGTTTTGACGAATAGTGGAACTAAAATTATTATGCTTGAGACCTGTATCTTTTGCCACTCTCGATTTGGAAGTGACTTCTAAAAATGTAATGAGTACTGCTTTTACTTTTTGGTTATCTTTAGCCTCTGAAATTCTGCCATGTCGAACCCCACTTATCAGTTCAACTTTAGGGTTTTAACTTGTGGATAATAAAAGTTTGAAAAGCGGGACTTTCGTGTGTTGTGTTGCTGTCTTTTTACCCGTAACTGTTGAATTTTTGCAGGTTAATACTAGCAACAGCAATGAGGTATTCAAATTTCTGGAAACCGCTAAAGATTTGCCCACGATACGTAAGTCATTGACATGATTGTATATAATGCTACTGAAAATTCTGTTGTGCGTCTAATTTTCGTAAAAATTTACCACTATTTGCCACATTTGTCACGTTACAAATGCTGCACGAGAATCTGCTAAGTTTCTTTTGTCGTTTTTTAAAAAGTTATTGTACTAACAAAATTAATTCTTCGGGGGGCTATCGTGATGTCTTAATAGTTCTAACATCACGTTTCTTGTTCTTATTGCAGTGGTGTCAAATGTCTACTGCCTTAACACCATGATGAAGTGCATAGCGGTGAGAGATTCTTGGCTCAAATTCATTGTTTGATCATTTTCGTTTTTTACAATCAAGAGGAGAAACATTGAACAATAGTCATTGCAAAAAGTTTAACGATATTCGAGATCATTTGAAGATGGATATCGTTTATAGTTTTTGAAAGAAATCAATTCAGGACCGAGACTTTTGATCGAGTATCTCTTAATTATTTTGCAGCTCGGTGCTGTTGAGTTTCTTCAGAAACCGCTATCAGATGACAAACTACGAAATATTTGGCAGCACGTTGTTCACAAGGTTTGTTCGATTCGTTACGAATGTCACTCGAATGCCTACTGTTTTCTCGATTTTTCGCCTCGTTTTATGGAAAGCGTGCCCTGAGCTTATTGTCTAAACAGGCATTCAATGCAGGAGGAGGAAAGGATGTCGAATCGCTTAAACCTGTCAAAGAATCTTTAGTTTCAATGTTGCAGCTACGATCAACGAAGAACGAAGCGAATACAGCGAACTCCGACGAAACTGAAGCAATCAACCTCGGTGCAGGAAAACAACCGGGACACGTTATCATCAGTATGTGATAAATACCCGGCTCCTTCGACTCCAACTCTGAAACAATGTGTGAGGTCACTTGACGATGGCGAATGTCGGGATCAAACTAACCTCTCAATGGCTTTCTTGTATTTTCACCTTGCTGGCAGTTACAACTGCCTTCCTTCCCATAGCCGTTCATATATAAATACCATCTGTATTTTCCATCTTTCAGTTGTAACAAACAGTATGATCAATAATGCACCTTTCTCCAGTTTCAGTTTACATTCTTGCTTCTCTTGTTTACTGCAGTCGTAGCTAACTCATCATGGTATCAGAGCGACGCTACCGGAGCTACGCGGCGGCAGTCGTGACAGAAGCAAACGGCGATGAAGATCGAGCGGAGCATATCGGAACCTCATCGCATCAGGCATCGATGAATCAACGGAACGACGAGTTGTTTGACGATCCTCTGTACCTGCATATCACAGAAAATCCTAATCTGAGCTTGGTATCGCCTCCTCTCACTGAAATCAATTATTCCTCATGGAGCAGATCCATGAAAATTGCTCTTGAAGTCAAGAACAAATTCGGTTTTGTCAACGGAAGCGTTCCAAGCCCTGAGGAAACTGATCCGAGATACGCATCATGGAGGAGATGCAATCGGATAGTCTGTTCGTGGATCCTGAAATCCATCAGCCCTAGCATCGCCGATGGTGTCATGTACTTCGACAAGGCATCGGAAATCTGGAATGCTTTGCATAAAAGGTACTCTCAATCTGATCCACACAAAATAGCTGAAGTTCAAAATGAAATTTATAGGATTATGCAAGGGAATATGACTGTCAACGAATACTTTACCAAGAGCAATGCTCTGTGGCAGCAGCTGAATGTCTTAAGGCCCCTACTCTTATGTGAATGCACACCTGTATGTTCTTGCACATTGTTGAAAAGAATGCAGAAGGAAAGGGAGGAGGATCAGATAATCCGCTTCCTAGATGGTCTAAATGAGGAGTATGAGGTTGTTAAGTCAGGAGTCCTTGTTATGGATCCAATTCCTGACATAGAAAGAGTTCTGAATATGACACTGAAGGTTGAAAGAAAAATCAGAGGATCAATCATTCAGAAACACAGTGAGATCATCCAGGCAAATGCTATTCAGAATGTTCAATCACAGCCAGAAGAAGAACAATCTTTTGTGGCAGCCTTTAACAATAGAAAGAATTTCACAAGCAATGGAGGGAAAAGTGTGCCAAAGTGCACATATTGTGGCATGACAGGCCACACTGTTGAGAAATGCTATAAGAAGCATGGGTACCCATCAGGTTGGGTTGCAGGCTACAAATCCAAGTTTAAGCAGAATCAGAACATGCAACAGTCAAGCAATGCCTCTGTGAGTCAAGTTAGTGACACAGGGTTGTCTAATGATCAGTTTCAGAAGTTAATATCCATATTGCAGAACCAGAATAAGGGGAATCAGACTGTAAACAGTGCTGTCATGACAGTTGCCAGTTCTGGAATCAAGGCTGACTTCACAGACTCCATGGAGGAACATGATGAAGGTAATGTTCCACCTAAAATCTGTGTTACTGCTGCTTTGAGTACAAATGTTTCATCTAGAGTCTATATTAATGCTGCCTTGAACACACACAATCTTTGGATTCTAGATTCAGGAGCCACAGACCATATAGCATGCTCCCTAGAATTTTTTGATACTTATCACAAGGTCCAAGGCTTATCAGTTAAATTGCCTAATGAGGAATCAGTCATTGTTACATGCATGGGACAGATCAAACTTGATGAGAATATATTGCTTCAAAATGTTTTGTACATTCCAAATTTTACCTTCAACATTGTTTCTGTTAGTAAACTGACCAAGGAATCAGGTTGTGAATTGCTGTTGGGGGCTGACTATTGTAATATTCAGGGACCCCTTGGGAAGGTGGTTGGTTTTGCTAAAGAAAGGAATGGGCTGTATTTGATCAATGATCCCCCTGAGAGAGTTAATGGACCTGCTGTTCGGATAAAGGGCTATATTTTGTATGATTTTTCTGACAAATCTGTGTTTGTTTCCAGAGATGTAAGGTTTCTTGAACAAGTTTTCCCTTTCAAGAACCAGAGTGCAGGGTTCTCAGAGTGCAATGAAGATGAGATCATCAATCCTTCCCTTCCTCTGGTGCCTACATCAATAGAAACAGACTTTGATCTAAGGAGACCAGCCAGCCCTACAATCCTGCAAGACAGTGCAGACAATAGTTTTTGGGTAGTGATGATCATAGCCACAATGATCACACTGCAAACAGTGAGGAGATAAGTAACAGTGAAGAAATGGACAATCAGCCTCTCAGTGACAACATCAGTCCACCACAGATTCAGACTGAGCAGCCTAGAAGGTCTGCTAGAATAAGAAATGCTCCTCCTAAACTCCAAGATTATTATTGTCAGTCTCTTGTGGGAAAGGAGGACACAACTAGAACTTCTCCTCATCCCATAGGCAAGTTCATTTCCTATAGCAACTTGTCTCCCTACTCATAAGGCTCTTGCTGCATCCATCACTTCTACACAAGAACCACAGTCCTATAAAGAAGCAGTACAGCATGAACCTTGGAGAGAGGCTATGCAGTCTGAAATTAAAGCCTTGGTCCAGAACAGTACCTGGACTCTAACAGATCTACCCTCAGGCAAGACTCCAATTGGTTGCAGATGGGTTTATAAAGTGAAATACAAGGCAGATGGATCAGTAGAAAGGTATAAGGCTAGACTGGTGGCCAAAGGCTATACACAACAAATGGGAGTGGACTACATTGAGACCTTTTCTCCTGTTGCTAGGATGACAACTATCAAAACTTTTTTGGCTGTGGCAGTAGCTAAGAATTGGGACATCCAGCAGCTTGACATCAACAATGCCTTCTTGCATGGAGATTTGGAGGAAGAGGTATACATGGTTCTACCTCCAGGGTTCAAGAGTGATAAGCCTAATCAAGTATGCAAGCTGCTAAGATCCCTCTATGGCCTGAAACAGGCCAGCAGACAGTGGAATGCTAAGCTGACAAAGGCTTTACAAGGCTATGGCTTTCACCAATCCACAGCTGATCCATCCTTGTTTACCAGAACTTCTCACAACTCCTTCCTAGCTCTATTGGTGTATGTAGATGACATACTAGTGGCTGGAACAGATCAGACTCAAATCAAGGAGTTGAAACAAAGATTGGATGATTCTTTCAAGATAAAAGATCTTGGGGAGTTGAACTATTTTTTGGGGATTGAAGCATTTAGGTGTCCCACTGGTATTAATCTATGCCAAAGAAAATACACTTTAGAGATTCTGCAAGAGAATGGCTTATTGGATGCCAAGCCTGCTAAAACACCATGTGTCACAGGACAAAGACTCACAAACACTGAAGGAACTCTTCTAGATAATCCTGAAGTATTCAGGAGATTGATTGGAAAGCTTTTATATCTCACAAATACTAGGCCTGACATCTCTTATTCTGTACAACAACTTAGTCAATTTGTGGATAAACCTAGAGACACTCACCTGGTTGCTGCACACAGAATATTGAGATACCTCAAAGCTTCACCTGGAAAAGGGCTGTTTTATCCCTCTGATTCCTCCATCAAGTTACAGGGATTCTCTGATTCAGATTGGGCCACCTGCTCAGAAACAAGGAAATCTATTACAGGCTATTGTATATATCTTGGAAACTCTCTCATATCTTGGAAAACCAAGAAGCAAGCCACTGTTTCTAGGTCCTCATCAGAGGCAGAATACAGAGCTTTGGCATCTACTACATGTGAAATTCAATGGTTAATATACCTACTCACAGATTTGAAAGCAGACATAAAAGGTCCCATTGCATTGTTTTGTGACAACAACTCAGCCATTGCTATAGGAGAGAATTATGTGTTCCATGAAAGAACCAAACACATTGAGATCGATTGCCATGTGGTGAGACAGAAAGTTCATGAAGGTGTGATTAAATTGCTAAGCATTCCCTCTCAAAAGCAAATTGCAGATGGCTTTACCAAAGCCTTACCTCAACCTTTGTTTGACGTTTTCCACTCTAAGCTGGGCCTTCAAGATCTTCATGCTCCAGCTTACGGGGGGGTGACGAAGTGATCAACAGCCCCAAGGGCTTTCTTGTATTTTCACCTTGCTGGCAGTTACAACTGCCTTCCTTCCCATAGCCGTTCATATATAAATACCATCTGTATTTTCCATCTTTCAGTTGTAACAAACAGTATGATCAATAATGCACCTTTCTCCAGTTTCAGTTTACATTCTTGCTTCTCTTGTTTACTGCAGTCGTAGCTAACTCTGAAACAATGTGTGAGGTCACTTGACGATGGCGAATGTCGGGATCAAACTAACCTCTCAATGGAACACGATAGTGTGGAGCACGATGGAGAATCTAAATCTGTCGAAACTACTTATTGCAATTCTGTTTCTGAAACTATTCCCGTGATTAATCCACCTGTTATCAAGCAGGAACGAGAATCAAGCCCTGAACAGGCGGGGAAGAATGGTAATTCTGCTTGTTCAGAGAGTAAGGATGCTCGTGCCAACGCCAATAGTAGTGAGTGCGGTGATCCTAAAAAACCGTCTGGTGTCAATAGTTCAACCGGGACAAAAGCTAATAAAAAGAAAGTCAAGGTTAGATGCTTATCAATTTGTACATCAAGTGAGTGACAGTAATACTGACTCTGTTACAATGTAGTATCTGTTCATAACTACTTTCTCAACCTACAAAAACAAAGAGATGCCTCCACTGAGGCTATTCGAGTTTTCATATAATCGTTCTGTCACGCAAATTGTCATTCGTGGGATTTTAAAACGGAGAAAACGTTGATGAAGTTCGTTTGGTGATGGTAATTTCAATCTGGTGTTTGGTTGGTCAGGTAGACTGGACACCGGAACTACACAAGAAGTTTGTACAAGCGGTGGAACAACTTGGTCTCGATCAGGCCATTCCTTCACGAATTCTCGAAGTGATGAAAGTTGAAGGTTTGACGAGGCACAATATAGCCAGCCATCTTCAGGTATTTCGATGTCTATACTTGTATTACATGCTCAAACTCTTTGTTCTCGGAGTAAAACTCGGGAGTTTGTTTTTCTCCTCGTAATTGCAATAGCCAAAATGTAAACACACGAGAACCCACATTATAAAACGTGAAGAATGCAAATGAAAAAAAAAAAATGGAAAACACGGGGAATGCTTTTCTGAAGTGTATGTACTATACTTTCTTTTCGAAACAGAAGTACAGAATGCACCGGAGACAAATATTACCAAAAGAAAACGAATGGAAATGGCCTTTATCACGAGATTCAACGCAAAGGAGTTGTTATCCACGTAAACCTGTAGTGGCCTTCCCACAGTACCATTCTGCTCCCACCGTCCCAGCTGGTCAGTTCTATTCTGCGTGGGCGCATCCAGGCAGCTATCCCGGTGCCCATGTGTGGGGCTCGCCTTACCACTATCCCGGATGGCAGCCTACAGACGATTGGAACTGGCAGTCCAACACAGGGGTAAGGTTAATCTCCTCGAGAGTTGCATCGATCAGTTCATATTAACCTCGAATTATTGTCGTGATAGTTAAATATGGCTCGAGACACATATTCACAACATTAACTGAAAAACAATTATGTATTTTAAGGTTTATGCTCAAGCATGGGGCTGCCCCGTTATGCCAGCACCTCAGGGATCATATCCAACATATCCTCAGGTACACTTTACACAATTTTACTAAATACCTGCAATTAGCAGTGCCCTCGTTTTGTCACCATAAACCGATTGATTTGGATGATTTTTTTATTATTATAACACATTCGTTAGATATTGCAGAACACCTCGGGGCATCACAGGAGTGGCGGAGCACAAGATAGATACAGCATGCTAGACAACACATACGATATTCAGCCAGTAAGTTTTTGGCATTCGATATTTCATCTGTCATTTAAATTAGACGACTCAAATTGCAGAATCCCGATTACATTATGCTTCATACCGAATTCCCCGCTCTTATTATATATTGACTGTTATAATGTAGTATCTGTTTCTACTGAAGCACAAAGAATCAATGCCCTCATTTGAGAAGTCATAGCTGTGCCGACCACAAGGCCTTTGGCTATGTTATTAGATTATTTCGTTTTTTTTTTTTTTTGCAGGCAGAAGAGTTGATTGACAAAGTTGTGAAGGAGGCGATAAACAATCCATGGCTGCCCCTTCCTTTGGGGCTAAAGCCTCCCTCCACTGACAGTGTCCTCAATGAGCTTTCTAAACAAGGCATCTCCACCATCCCTCCACGAACCAACGGCTCAGATTTGCGCTGA

>g15212

ATGGTTTGCACTGCTAATGATTTATTGGATTGGAAAGATTTCCCTAAGGGCCTTAGGGTCCTCCTCCTCGATGAGGACACCAATTCTGCTGCTGAGATGAGATCAAAGCTTGAGGAAATGAACTATATAGGTAATCTGATCTACCCATTTCTCAAAGATACTACACTTCTTATCCTTTCTCATATTATTGTCCTTTGAGATGCATCATGGTAGTTTAATGGGATTTACTGTTTTGATAGACAAATAGTGTAGTACCAATAATTTAGATAGTACCAAGTAGGTTGGTTGTCACAAAGGTCAATCCTTTTCTCCTCTGGTAGTTTTAGTATCAAGCGCTTACCACTTACGACTTGATCTTTACTGGCCTAAGGTTGGTTCTGATATCAATTGTTAGGATCAAGCGTTTATCACTTACGTCAAAAGTTATAGCTAGTAGAGAATGCATAACTTTATTTCATTATACTGTCACGCATTTTCGAGACGCTAGGTGCTAGATTTGGCCTTAAGCTCTTTATCGGCCTCCGTCCAACAATCCCGCCATCGGGTGCTAGACTTGGTCTTTACTAACCTCTGTCCAACAGTTTTTAACTTTTGGAGAGTTTTGATCTGCAGGCCGGAGAGAAACTTGATTGGAATAACACTATACATTTAATGCATACATAGTGTATTGGGTTATGCAATACCTCCTCCATAACTATAAGCTGTTGCTTCTTTGGTAAAAGAAAAATCAAATTTACCAACAGAAAAATTTGTAGGTAGGTAAATCAACGAAACCCATAGTATATTTAATTAATTTACCACTTTTAGTAGACAAGTCAATAATATCTGTAGGTATTTCAGTGTTTACTTGTAGTATTTAAAAAAGGTTAGTAATATAATGCCCTTTAAAACTTGTATGTGCCTCAATCATTGCTGCAAAAATGTCCCACTTGATTTGCTATTCTTTTTTTTTTTTTTGGTGAGGCTTGATTTGCTATTCTTTACAGTGGTAAACTTATTTTTTACTGTCCATAAGAGTTCTTGTACAAGCATATATGCATTCAAAAAGCATATATGCATTCATCCATTGTTTGCAAAGAAGTTATGGCCCTGATCTTTCCCTGCAGTTTCTACATTCCAAAAAGAGAATGAAGCTTTATTGGCAATCTCAAACAAATCTGAGGCATTTCATGTTGCAATCATAGAGGTACTACTATACTTTTTCAAGAAATTCCTTTTTTTGTGCTTTTATATAGTTCATGAAAACCAACATGGTTTAAAAAGGTTTTGGTGAATAAAGGAATTGAAATGATAAAGCAGCCCATATTACTTGCCACTCCAGAGCAGGAAAAGTGGCTCCTAAAGTGTGTTGGGTATCTTCTTTTGTTTTTTTGTTCTTTTTCAACTATCTATGGCCTCTGCAAATTCACCCCATTTATCATCTTCAACTTTAGGTTTTTGTCATGGAATCTCAAAAGCACTATTGTAAAAATCGGCCTAGGCGGCCATCTAAGCACCGATTAGGCATTAGGCCCTGATTAGGCCTTAGGCGCCCCTAAGCCGAGGAGATTTTACCTTAGGTGGGCATTAGTCGGCCGACTAGGTGGCCTGGTGTTTTATTTTTTATTTTTTGGTGTGCAATGTGTACATTGCTGTGTTTTTCTTATTATTATTACTCGTCTGCCTAGGCGCTAGGTGCCTCGAGCTCCCGACTAGGGCCTAGCTCCTTCTACCAACATTGCTCAAAAGAGGACACTTTTTTTCCAATTGCAACCATTTTATACATATCATTGATGGTCACAGGTCAAAACCGGAAACAGCGATGAGGCATTCAAATTTCTTGAAACTGCTAAAGATTTACCTACCATAAGTAAGTATTAGACATGATAATTCCATCTTTTGTTCTTATGTGAAGCATTGCTGTGCAGATTTTTGTAGAGTTCCTCGCTCTCTTAGCAAGTCTAGATAGTTCTAATGCTGAAATTTTTTGTTATCCATATTGCAGTGACCTCAAATATCGACTGTCTTAATACCATGATGAAGTGCATTGCGGTGAGAGCTCGAGCCTTGAATTCATTATCCATTGCATTTTCTAGTTCAATGTAAATTTAACGTTTATATTTAGCGTGCTAAATGCACATAGTTAAAAAAATTTATACGCCTTAACTGTCTTGCAGCTCGGTGCAGTTGAGTTTCTTCAGAAACCCCTATCAGATGACAAATTGAGGAATATATGGCAGCATGTTGTTCATAAGGTTTTTACTACTTCTAAATTTGCTCTGTTGCGTTTTCCCGAATTGTTCACTTGTTTTATGGGGAGGAAATCATGAGCTTACTGTCTAAACAGGCATTCAATGCAGGGGAAAAGGATGTGTCCGAGTCGCTTAAGCCTGTCAAAGAATCCATAGTTTCGATGTTACAGCTCGAATCAAGAAATAGCGGTGCAGATGCGCAGAACTCTAATGAAACGATACGAGAAAACAGCCAGGAGTTTTCAGCAGACTCTGATAAATACCCGGCCCCTTCCACTCCACAACTGAAGCAAGGCGCAAGGTCGCTTGATGATTGTGAATGCCTGGATCAAACTAACTTCTTGATGGAACGCGATAGCGTGGAGCGAGATGAGGAATCTAAATCTGTCGAAACTACTTGTTGCAATTCCGGTTCTAGCACTAATCCCGCAATTAGTCCACCCGTGTCACTAGTAGAAGCTAGCATCAAGGGGGAGTGTAAATCGAGCCCTGATCATAAGAGTCGGACCGAAAATTCTACTTCTCTGCAGAGCACCGATGCTCCTCTAAATGTCAGCAATGAATCTGTTGCGCCTAACAAACTGTCTAGAGTGAATAGCTCGAGTGGGACGAAGGTTAATAAAAAGAAATTAAAGGTAAGACACTCATCTATTTGTAAAAGTAAATAATGTAGTGAATGCTTGTTATCTCGAAATACTTATCCGATCCATTGGATATTTGTGGGTTAATTTCTGGTATTTGGTTTTGGCAGGTAGACTGGACACCTGAACTGCACAAAAAGTTTGTACAGGCAGTAGAGCAACTCGGTGTCGATGCAGCCATTCCTTCTCGAATACTAGAAGTGATGAAAGTTGAAGGATTGACCAGACATAACGTGGCGAGCCATCTTCAGGTAAGCCTATAATATCTGCACTGATACGTGCTCGAATTTTGTCAAGGCAAGTGGAGTTGTACAAATAGGGAAAGAAAATCTCTCGTAAATTTTGGTATGCTTTCTTTTCGAAACAGAAATACAGAATGCACCGCAGGCAAATTTTGCCAAAAGAAGATGCGAAAAGATGGCCTAATCCAAGAGATTCAACACAAAGGAGTTGTTTCCCACGCGATCCTATATTGGCCTTCCCGCCATACCATTCTCCGTATTCCATCCCGAGTGATCAATACTATCCCGCCTGGGTACAACCAGGCAGTTATCCAAGCGGTGTCCAGATGTGGGGCTCGCCTTACCACTATCCCGGATGGCAGTCTACAGATAATTGGCACTGGAAACCCCATCCCGGGGTAACTCTCGTTTCCTTCATCTAAGCATAAGAATAATAATAATATTAGCTGAAGTGTTGTACATATGGCGTTTAAAATGTTCTCTGAGATTTATTTTCGCAATGTTTAGGCACACGCCAATGCTTGGGGCTGTCCGGTTATGCCATCGCCTCAGGGGTCATATCCAACATATCCTCAGGTGAACACTTGGAACAATCTTTCGAGTAATGTGGTTTAAACACCGGTCTTGTGCTGCTACCTTATAGCGAGTGATTTAGATGGCCCGGGACTGATATTACTGTGGTTTAAGCATTGCAGAATGCATCTGGATATTACAGGGCTGATGGAGTGCAGAACAGATACCACATGCTGGAAAAGTCATTTGATTTTCAGCCTGTAAGTTTTTTGGCTTTTTCAACTATTTCACCTCAACATTATCTTATACTTGAAGTGAAGAGTCCACCATTAGTCATGGAAAGTTATTGGTTAAGCTTCTCTCGGATTGGGGACTTCTTGTGTGCAGTTAGCAAAAATCTAGTCTCTGTTGATCAGTTGAGTCACCGTGATTACCCCTTTATAATCCTGTCGGGCCGGGGAGTAGGAGCACCTAGGGTGGATGGTTCCACATTTTGTCACAAGTTATTGGTTAAGCTTGGCCTACATAGATAACATCCAACCAATCCTTGCTTGCATAGCTATCATTGCGTATTAGATGTTCTACATTATCAATTATCAACATTTGAGTTGTTTCATCTGGCCTAGTCATCAATCACAAAATCTCCATCCCATTGTATCTTGGAGCAAATTCTACTAAATCATATGAGTTTATGTATAAGATTTTGACCTTTGAGTTTGAGACAACTTGGTGAACGCCACGTCCTTTGTCTTGGCCCGCCATTATAGCCAAAATGGGGCATTCCAAGCATAGGCATAAGATATAAACTTTTGAGTTTGAAACAACTTGTTGAGCACCAAATCCTTAGTCTTGGCCCGCCATTATACTCGAGATGGGACATCCATTTAGTATAAGATTTCAAAAACTTTTGAGCTTGAAACAACTTGCTAAACATCAAATTCTTAGTCTTGGCCCGCCATTATAGGCGAGATGAAACATCCAATCCGACAATAAGTACAAGTAATAGTATTGACTTCAAGTTCAATTTTCACTCTTTCTCGGATTTACACATAGATTTGAATTCTTAACCGCATTGTTACACACACATAAATTTAAATTTTTAGACTCATGTTTAAACGACTTGAACCCATTCTACTTGAACCAACCACTTTTGTTTGATTTTTGTGCAGGCAGAGGAGGTGATTGACAAAGTAGTGAAAGAGGCTATAAACAAGCCATGGCTGCCACTGCCATTGGGACTAAAACCACCCTCCACTGAGTGTGTCCTCAACGAGCTCCTGAAACAAGGCATCTCCACCATCCCTCACAAGATCAACGGCTCACACACGCGCTGA

>g56632

ATGGGAATTAGCAAAAGAATTTGCTTACTGGTCTTCTGTGACGATTATATCTGCCAAAACCTCATCTCCGAGTTGCTCCAGCACTGCACGTATGAGGGTACGCACCTCATGTCATACATATATCATGAATTAATTGTTATGTTTTGGTTTTTGCTTGATCGTCTGTCTTTGATTGATAGATGTTAATTAGTTCTTAATTATTTTAATTTGTTTGTTTCACTGTTTTTGCTTGTTTTTTTGTTCATCAGTGTTGCACATTGGAAAGGCTATGGATGCGTTAAGTGCGATTGGGAAGAGAAAAAATGGGATTAGTGTTGTTTTGACAAACATGAACAGGTTAAAGACAAAGGCGGCTGAGATTATCCACCAAGCCATCCAGGAGGAACTTACAATAGACACGCAGATTGAGTTCTTCCTGTATGGCTTGGTGGATAATCTCAGCCCCCTTTGTGTTTAA

>g56635

ATGGGAATTAGCAAAAGAGTTTGCTTATTCGTCTTCAATGAGGATTATATCTGCCAAAACCTCGTCTCCGAGGTGCTCCAACACTGCTCGTATGAAGGTACGTACCTCGTATCATGAATTAATTCTTACGTTTTGGTTCTTGCTTGATCGTCATTTGTCTCTGCTTGATAGATGTTAATTAGTTCTTAATTTGTTTGTTTCACTGTTTTTTGCTCATCAGTGTTGCACATTGGAAGGGCTATGGATGCGTTAACTGAGATTAGGAAGAGAAAACATGGGATCAGTGTTGTACTGACGAACATGAACAGATTAAAGACAAAGGGGGCTGAGATTATCCAAGCCATTCAGGAGGAACTTAATCTGCGTGTCTGTTGTAAGTTCCTTAATTTCTTCAAAAATTTATTTCTCACATATATTAATTCTAATTAAACTCTGATTATATCATTTCAATTCATTTTTGACAGTGATACTGCCCGGTAACATGGAGTTCAATGACACTAGAGGCCTGGACTGCAATGTTTCAGCCTACATTGTCAATTTTTCAGATACAAATGACATGAAAGAGCTGTGGCAATCAGCTTTTGAGAAGGAGAAAGCCAGAAAAGCAGCAACTACCACTGATAATAATGAACCTGGGAATGATCACCATAACCGGAAGGCGAAAGAGCTGAGAGAGAACCAGAATGAGGAATGTGGTAGTGAAACTAAAAAGAAGCCGAGGTTGAGCTGGAACCCGGATATGCATCAGAGATTTGTGGAGGCAGTCAACAAGTTAGGCTTTGACAGTAAGTCATCATCCTATCCTTTTCTTCATATAAAACATAGTTTCTTGTCCATTATTAACTTTTTGATTCATTATTCTAACGTTGGTTAAATCGTATCCTTCGACAATCAGAGGCAGTTCCGAAGAAAATAGTTGAGTTTATGAACGAGCCAGGGTTGACAAGAGAGCATGTTGCCAGCCATTTGCAGGTCTGTAGTGTTATTCTTTAG

>g56637

ATGGGAATTAGCAAAAGAGTTTGCTTATTCGTCTTCAATGAAGATTATATCTGCCAAAACCTCGTCTCTGAGGTGCTCCAACACTGCTCGTATGAAGGTACGTACCTCGTATCAAACATATATCATGAATTAATTGTTACGTTTTGGTTCTTGCTTGATAGTCTGTCTTTGCTTGATAGACGTTTATTAGTTCTTAATTATGTTAAATTTGTTTGTTTCACTGTTTTTGCTTGTTTTTTTGCTCATCAGTGTTGCACATTGGAAGGGCTATGGATGTGTTAAGTGAGATTGGGAAGAGAAAACATGAGATCAGTGTTGTACTGACGAACATGAACAGATTAAAGAGAAAGGGGGCTGAGATTATCCAAGCCATTCAGGAGGAACTTAATCTGCGTGTCTGTTGTAAGTTCCTTAATTTCTTCAAAAATTTATTTCTCACATATATTAATTCTAATTAAACTCTGATTATATCATTTCAATTCCTTTTTGACAGTGATATTGCCGGGTAACATGGAGTTCGATGACACCAGAGGCCAGGACTGCAATGTTTCAGCATACATTGTCAACTTGTCTGATATGAAGGACATGAAAGAGCTGTGGCAATCAGCTTTTGAGAAGGAGAAAGCCAGAAAAGCAGCAATTAGCAGCCCAGTAGTGGGTGTTGAGACTGGGGAACCTGGGAATGATCACCATAACCGGAAGGCGAAAGAGCTGAGAGAGGAACAGAATGAGGAAAGTGGTGGTGAGACTAAAAAGAAGCCGAGGTTGAACTGGAACCCGGAGATGCATCAGAGATTTGTGGAGGCAGTCAACAAGTTAGGCTATGACAGTAAGTCATCATCCTATCCTTTTCTTCATAAAACATAGTTTCTTGTCCATTATTAATTAACTTTATGATTCATTATTATATTCTAACGTTGGTTAAATCGTATCCTTCACAATCAGAGGCAGTTCCAAAGAAAATAGTTGAGTTTATGAACGAGCCGGGGTTGACAAGAGAGCATGTTGCCAGCCATTTGCAGGTCTGTAGTGTTATTCTTTAG

>g31109

ATGAGGGGAGTTCGGGTGGATGGCAATGGTCCTCCACTTAAAGGGTTAACAGAAATCAATCACAATGGTATGCGGTCTGAGCAGAATGGAGTAAGAGATGGAGTTAATGGTGATGGACATGGGCTCTCTGAGGAAGACGAGTCGAGGATTAATGAAGACACTGAGGACAGAAATGACATGCGCAGAGATTTAATGCAGGTGCAGGCTGCTCTTCATACTCAGCAACAACAGCCTCAAGGCCCTGTGGTTCGCTGGGAGCGTTTTCTTCCTCTTAGATCCCTCAAGGTTCTGCTTGTGGAAAATGATGATTCTACTCGTCATGTAGTCAGTGCACTGCTTCGGAATTGCAGCTACGAAGGTTGGCTTTTTTCTTGCTAAATCCCTTTCTGCTTTTGTGAGTGGTTTATAGTGAGAGTTGATGCCGATATTTTTGCAAATTTATTCTATCCTGTCCTTATTTGGTTAGCAGTATATAGATATCTGCTTAGTTTCCTGGCTGAATAAATATCAGCTAATTGAATTTTCAGGTAAATTCTTGTTGGGTTAGCATGTTCATTTTCTGCCTGGCTTTATGTTAATTGAACTTCTATTGATCTTTTGCCATTTAATGTCATTTTCTGCTAGATGAGATACTAATATTGATATTTGTATCATGTAGAATTAATTACACTTAATTTATAATGTTTTTGGAACATTTTTTACCATAGAAGAAATACTGACCTCCTGATTTCCCAGGTAATAATTGGCTTTATTATTATGATTTATTTTATTTTATTTTTTCTATTGAAGGGTTGACTGGAAGTTATTCCTTTTATCCCAGTAAAGCTCACCCTTACTTTTTGCATCTTTCTTCTCTAGATTTCTGTCACTTCGTTCCTTCTAAGAAAGAGAAGTGAATATAAATTTGGTTGCTCAGGCCTCATCATCTTTTAGAGAGAATTTTTCCATCCTATTGACTGAAATGTTTTCATACAACAGTTATGAAGCATACAGATATACCCATCCTCTCTCAAAACTTTCAGTAGCAATTTCCTGCTTGAGTGGATGTAATTATATCCTAACACAAGAAACTGTGATCTTACTAGAAATGCATCCATCTTGTACTCCTGTCCCTATCTTGTTCCTTTAGGGATTTTTTTTTTTTTAATAATAATAAATTGTGCTTTTGGTCAGGATAAAGGAAAGAAGCATCAGGCAAAATGTGCAATTGATGTTAACTACAAAATTCAGTTCCTATACTTCTTTAGCATTTAAACTTTGCTCTTGGTTCTCCCTATTGCCAATATTTAATTTGTTTAAGTATTCATCTTGTTGATTGTGAAATTTGAGATCTTTATCTGGGCTCTCTGCCAATATTTGTTAGAGCATTTCAAATTCCTCATTCTCAAGGAATATGTTGAATTGGATAACTGCAGTTACAGCTGTTGCAAATGGAGTTGAAGCATGGAAAATTTTAGAAGATTTAACCAATCACATAGATCTTGTTCTAACAGAAGTAGCCATGCCATATATGTCGGGCATTGGTCTGTTATCAAAGGTTATGAACCACAAAACGCGCAAGAATGTCCCTTTGATTAGTGAGTTTTCTTCCTATTTCATGGTTGTGTTTACTACTACTTTTTATGTTTTTAGCTGCTACACCTAATGAGTTTTATTTGCAGTGATGTCATCCAATGATTCTATGGGAGTAGTCTTTAAGTGTTTGTCGAAGGGCGCAGTTGACTTTTTAGTGAAGCCTATTCGAAAGAATGAGCTTAAAAATCTCTGGCAACATGTTTGGAGAAAATGCCACAGTGTAAGTTAATATCATCATCCTATCATGTATTACTTTGAAGCACCATTTCTCAATAATTCTTGATAAAGCAGCCAAGCAGGATGTTGGTGCTTAAATTGAAAAAGAGATTTCTTATCAAAAAACCAAAACTGAAAAGGATATCACTGTGTTTAACTTGGTGGACCTTCTTAATTGTTGCTCCTTTTCTCTCCCTCTCCCTCTCCCTTTCACTTTGTTTTAGTTTTTACCTTATTTTTTCCCCCTTTACACTGTATTTTTTCTAGCAAATTGTGTATTAGCATTCTGTTTTTCATTATTTTGTCATCCTTTTATCAGTCGAGTGGTAGTGGCAGTGAAAGTGGAATACGCACCGAAAAGTCCACAAAATCTAATAAAAGCATTGAAGGGTCGGAGAATAACAGCGACAGCAATGATGAAGGTGAGAATGGAAGCATTGGTTTGAACACCAGGGATGGAAGTGACAACGGAAGTGGGACCCAGGTACTTCTAAAGATCTTACCTATAATAGTCTTTTTCACATATGCATGATACCACACTAACATTCAAACATGTATTTGTTCTTTTTACAAAATATAAGAAAAAGTGCACTTGTAGTCCCTGAGTTATGACTAATTGCAATTTTAGTCCCTAATTGTTTAGCATGAATGTGTATAGTCCCCAAGTTTTTTTATTTTGGTTCTCCTATGTTGCAACTTTAGTCCCTAAGCTATGACTAAATTTCAATTTTGGTCCCCATCTATGAGATGACTAAAATTGCAACGTGGTTAAAATCCTAGTGACTATTTTATGTTCATGCTAAACAATTAAAGACTAAAATCTCTATTCAGTCATAACTTGGGGACTAAAACTTCACTTTCCCTGAAATATAATGTTCCATAATATCTATGTTTGCAAAAATGAATATTTTTTTTTTCAATTTTGTTAATCTTGTTAAATAACCAGTATTGTTAATCTTATTAATTTACATTAATGTGAATCTCGTGGGGCTCTTCTATTGTAGTTACTTTCATGTCTGCTGTCTGCATGCTTTTGTGTAAGCTGTATAACTTGGGTGTCATGGCCCAACTGTATACCATCTTATGCAATCAAGTTTATGTAGGACACTTGGCTGCCTAATTTGTTACTGATTGCCTATAAAATCCTATTGAATAGAAAACAACATAAAGCACCCAACTTATTGAAAAACTTTAAAAACAAACCGAAAAGTATATTGATAGTTACCTGACCAGGTTAAATATTCCTTCCAAAAATCACCAAAAGTAACTAGCAGGAAAAACTAATCTATAAATCAAGAACAAATAAGCATTTGAAAAGAAAGATCTTTAGCTTGGAATGCTTATAATTTAATTTAATTTAATTTTTTTTGGCATGCATACTCCAAAATTTTCCAAATATATTCCACTTACAAATTTTTGGAGGAATAAGAAGCAAGTGTATTAGTTCTAAGATGATTACTCTTGGACGAAATTCCCTATTTTGCTTTTAATTCAAAAACATGGGAAACAGTTTAGGTGCTGGTGTGCCTGCACTTAAGAGTATTTCCACGAGAAGTCCCATAATGATTTTTAAAAATGATGCATTATTTCTCATTTCTTTGTTTTATTTATTTCTTGTTTTCCCCCCAATCTTGTGACGCTTCCAAATGTCAAGTTCAGTCTACCACGCCGCTTGTAGTGCATACCTACCTTATGTTGGAAAAGGGGAGGAGGGGTTGTGAATGACAAATGACAATTTTCACTATAAAGGAGATTGTAGAGGCATTTTCTGCTTAATAAATTTTCTGTGTACCAAATCCAAATCACATCATATATGTAATGTGTTGCAGTAATGAATCAACAATAATTATATCAAAATTCCGGAATGCCATATATTGGAAGGAATGGAAGCCTATGCACTCTTTTCTTAGGTTTGTATATATATACAAAATGATGCAGTGTTCTAATATCATTACAACCCTTCTTTCTCACAGAGTTCATGGTCAAAACGGGCCATTGAGGTGGAAAGCCCACAACCCATGTTGCCTTGGAATGAATTACCTGAACCCCCTGACAGCACTTGTGCTCAGGTTATTCATTCCAGACCAGAAGCACAAAGTGCAAATTGGGTTCCTACAATTGCCACCAGAGAGTACCAAGATGAGGAAGATGATCAGGGTACTATGGTTATTTTGAAAGGGGAATATTGCGTGCTTGCAAACCTAGTTAATTTGCTAGCTCAAGGGCCTAATGACATAATTTGCCTCAATAAAAATGATTTGGAAAATATAATGTCCATACAGCTGAACTCAACTTGATTTGGATCTAGTCCCACTTGAGTTTTGTGTTTGTCCTAAAGTGTTATATCTTTAGCTTCTTTGTTTTCTGATCTGACAAGTTAATGATTTGGTTTGCAAGCAGAAAATGTCCCGATGGGAAAGGATCTACAGATAGGAGTACCTAGGTCTCCGGATTTACAGCTTAATGGTCCAACCAGTAAAGCATTGGATGGTGAAGCGAGTGCTAAGAAGGGCAAACTTGTGAATGTAGACTCCTCAAAAGATGACGAGAAATTGATTGGAAAGCTGGAGCTTAACAAGACTCGAAAGAATGAGTTAAAGGACAAGGATAATGGTCATGTAGCTGCCATCACCATCAAAGATAATGATCTAATGGAAATTACTGGCAATGATGTCCCGACTGATCCATCCAAGATGACTAACACCAAAGAAATAGCCACTTATAACTCCAAGGAGATGCCATCCCTTGAACTCAGTTTGAAGCAACACAGAGAAGTTGGAGAGACTGGAACCACAGTGCAAGAACGCAACGTACTGAGGCATTCGGACCACCTTTCAGCATTCTCAAGGCAAGTAGTAATTTGAACGCACTAAAGTTGAAACACTTACAGGTTACATTATTAGTTAATAAGACGCCATGGGAGGGATCACAAATTATAATTCTCATTTCCATGAACAGGTATGGCACTACTTCAACTGCTAATCAGGCTCCAACTGGAAATGTGGGCAGCTGCTCTCCTGTTAATAATAGCTCTGAAGCAGCAAAAACAGAATCATTGCAGAATTTAAGATCTAATTCAAGCAGTATGCCCAATCAGCGCTCCAATGGCAGTAGTAACAACAATGATATGGGCTCATCAACCAATAACATTTTTGTCAAGGCAGAGGCATTTACTGACAAACCAATCAATAAATCTTCTGCTGTGAATGCACATCCCTGCTCTGCATTTCAGCCAGTTCAGCATGGGCAAAATTCTTCCCTTCCAGGAAAAGCTGATTCTGCCAAGTCAGCATTGGCCCAGGCAAGGGCCATGCAGCAGCAGTTTCAAGTTCAGCATCATCATCACCATTACCATCACCATCATCACCATGTGCATAGCATGCAGCAGCAGCAGCAGCAGCAGCAGCAACAACAACAACAGCAGCAGCAGCTCCTGAATGAAGATTCTTTGCCTTCTAGAAAAACTGTTGCAGATGCTCCACATGGATCCGGACCCTATATGTTAGGTACACTGACAGATGGAAATACGAACTATGGAAGTGCATCGGGAAGTAACAATGCAAGCAATGGGCACAATGGAAGCAGTGGGCAGAATGAAAGCAACACTGCTGTAGTTGCCGAGGACACTAACATGGCTACTGAAGATGGAATAGCTGGAAAATGCACGGTTGGTGGAGAGAGTGGTAGTGGTAGCAGAAGTGGAGTAGACCAATGTCGACAAGCACAAAGGGAGGCTGCCTTGAACAAGTTCAGGCAAAAGCGAAAAGAGAGGAACTTTGAGAAAAAGGTAACTTTTCTTAGATCTTAAAGATCAAACCATTCAGCATATATAATTTCCCCATATTTCCAGCACCACTCTGTTTTGCAGAAAGAATACTTTGTATCTTGATGATACAACAACGGCTTGGCTTAATAAAAGCAGCAGAAAAAACATCTCATTCTTCTCCCTTTCTTCGGGTGTGTCATTTTCAGGTTCGATATCAAAGCAGAAAGAGACTGGCTGAACAGCGGCCACGCATCCGAGGACAATTTGTGAGCCAGTCCTCAGATAAAACCAAAACAAAGGATACAAATTGCTGA

>g30584

ATGAGCACCGCCGCTGCGCGCCCGCTGGTTACCATCCAAGCTTTGGAGAATGACATGGCCACGGACGGAGGCTCCGCCGCCGATTGCCTCCCTCTGCCGGGAGTCATGAAGGCTCCGATCCGACCTGACGTGGTCACCTTCGTCCATTCCAATATCTCCAGGAATTCCCGCCAGCCTTATGCTGTATCGAAGCGCGCTGGTCATCAGACCTCCGCGGAGTCATGGGGACTGGCCGTGCTGTTTCTCGTATCCCTCGTGTGCCGGGAGGTGGTACGCACCGCGCGGGGCAGGGCGCGTTCGGGAATATGTGCCGCGGTGGACGTATGTTCGCTCCGACTAAAATCTGGCGCCGCTGGCACCGGAAGATACCGGTGAATCAGAAACGATATGCGGTTGTTTCCGCTATTGCCGCCTCGGCTGTTCCGGCGCTCGTGCTTGCACGTGGACATCGCGTGGAATCCGTTCCGGAGATTCCGCTCGTGGTTTCGGATTCTGCGGAGGGCATTGAGAAGACCTCGAATGCTATCAAGGCTCTAAAGCAGATCGGAGCCTATCCTGATGCTGAGAAGGCGAAGGACAGTCACGCCATTCGGCCTGGTAAGGGTAAGATGCGTAACCGCCGCTATATTTCACGCAAAGGTCCTCTGATTGTGTATGGAACTGAGGGAGCTAAGCTCGTGAAGGCTTTCCGTAACATTCCGGGCGTTGAAATTTGCCACGTGCACCGTTTAAACCTTCTCAAGCTCGCTCCCGGAGGACACCTTGGCCGTTTCATCATCTGGACTAAATCCGCATTCGAGAAACTTGATGAGGTTTATGGTTCATTTGACAAGCCTTCCGAGAAGAAGAAAGGATATGTATTGCCAAGGCCGAAGATGGTGAACGCTGACCTTGCTAGGATCATCAACTCGGACGAGGTTCAGTCCGTTGTCAGACCGATCAAAAAGGATGTTAAGAGAGCGACTTTGAAGAAGAATCCTCTGAAGAACTTGAATGTGCTTCTCAAGCTCAACCCACATGCTAAGACTGCTAGGAGGATGGCCCTTTTGGCTGAGGCACAACGAGTTAAGGCCAAGTCTGAGAAGCTCGCGAAGAAGAGACATCAAATCTCTAAGGTATGTGCTGTCGCTAATCTACTTCATTGTTTTGACTATTATTGCTTTAGTTTATATTCGTGTCTACTTCATTGTGAGTTACATTCTTTTTCAGTCTGCTTAACTCTATTCTCTTACATTTTATATATGATTGTAACATACTAACATTTACTTCTTCTGTGTATATCTGACTGTGTATATATCCTAGTTTATATTATCATGATGACAATTTCTGTCTTTAATATATCTAAAGAACTAAACTTTTTAATGATAGCTGAAGCTTTTAACATTACCTCACTTCGGCTACATGAATATGAACATATTGTTTTTAAGTGTACAAGACTTTTTTTACTTTATTGGGTATTTCTTTTGAGGTTATATGCATATTATGTTTATGTTCTTGATGTTAAGTGTATCCTTGTTTTAGTTGTTTGGTTTGGTACTATGGTCTAGTGAGGTTTCTGATAACTACTGCACTTAGATGCTCTGTTTTGGAAAGATGTATGAAATTTCTACATTTAATTCTGTTTTTTTGCTAGAATCTCACAGTTTCTCATGTAATTCTTCGTATGATGTGTATAATGTTAATAATGTGGGCTTTATCTGCCAATAAATATTGCATCTTGATTTTGGGTGAGCAGGCAGTTGGGCTTCTGATGTATTTGTGCATTGTTTTCAGAATGCTCTTGTCTCTCTGATGTCTCCCCCAAATATTGAATCAGGATTTAATGCTTGCTCTCCATGTTATATGGGTTTGCAAGTTTGGCTTTTCTGTTACATTTTAAAAATAAAACATACTCCTAATTCATTAGTCATTACTAGTTTACTACTAAAAAACACACTTTCTTCTCATCCATAAAATGGAAAATGTGGTGCTCATTGTTGTTGTTACATCAAATATTGCATCTTGATTTTGGGCGAGCAGGCAGTTGAACTTCTGATGTATTTGTGCATTGTTGTCAGAATGCTCTTGTCTCTTTGATGTCTCCCCCAAACACTGAATCAGGATTTAATGCTTGCTCTCCATGTTATATGGGTTTGCAAGTTTGGCTTTTCTGTAACATTTTAAAAATAGAATTAATTAACCACAATTTAAGAACACTTCTCTACACCCAAAAACTGGAAAATGCATTAGTTTTGTTTGGTGATCTTTTGGTGCTTCTACTTCATTTCCCTGTGCTCATAGATGTTAGTATCAACTTTTTTGTGTTTGAATATCCGGTGTATTGACTCTCATCTCGTATTACAGGAGGAGGCATCTGCAATTGCAGCTGCTGGTAAGTCATGGTACAAGACCATGATATCTGATTCCGACTACACTGAATTCGAGAACTTCTCTAAGTGGTTGGGAGTGTCTCAGTGAATCCATTGCGATTGAGGATACTTAAACTCTACCTTCATTTTGTTGTTTTATTTAGTTTTTAATTTTTTTTCTGCAGCTTTATAATTTTATTGGTGGTTAAGACGTGGGAATGAGAATTACAGTTTTTGAGTTTCATGACTTAGTACCAGTTTCCCCTTTAATCCATATTTTGGTATTGAATTTTGATCTTAGTAACAGTATATGCTATTCAGAGTTTTATACATACAAGTTTCTTTTCCATTGATTGATGGATTCAATAATGAGTTGCCAGCTTTGCATTGTGAATTGTGTATCTTAAAACAAAATATTTGTACCTTTAGTTAACACACTACACATGTACAGAAACTGGTAACTATAAGTATGATTTCCTGGGCCATCACAAACATAATGGTTTCCAATTCCAATACTTTTTGGGTTATTCAATTTTGAACAATTTGAAACCTCAGTACAAACCAAACCAAATTCCACTTGGCCAACTAATCTGGTTGGCTGCCACATTAAATTCCCAGAATTAGCTTTACTAAATAGATCTATTGCACCATATCATTTGGTTAAGGCTTAAAAGGGGAATCAATTCCTTTCTGCAGAACAGGATCATCAACCTATCTTGGCTGCTAATCACCAAGGCATTGAACCATGCATGAATTCATGCCATTGAATAAAAACTAAGGAATGTGCACATGACATGATATAAAGAACTCTCCTATCTTGCTCACTGAAGACAGATATTTAGTTATTTACAGACAAAGTTTCATAATTCTGTAGATATTTTGACCTTAGTAGAGACATGTTAGGTATGCACAGGCATTTGTTCATCAATACACAGTGCTGTTGTTCAGCATGATCTATCTTCACTTTCATTGCCAAGATGCCAAACTCATATACAGTTTGCAGAACCCTTTAGTTCATCCATCCCCACTGTTAACTTTTCCTTGAAACTCATTAATGTAGCTGGCTTGTTGTGATTCCTGTCATCTCCATGCTTCAGAAGAATCAACTGTAAGAACATATGCCTTTCTTGATTTTGCTGGGCTTTTTATGTTCCCATATTCTTTCTGAATCAATTCATCTGTTTCTTCTTATGTGATGTAATTCTACTAGATAGCCTGAATGTTCTCTACTTCTCTCCCGATCTTGCATTGGCTCCACAAGAGATTGCTGAACGGTACTTAAGTTAGAGTTGAAACATCTCCCCGTTTTGATTGTCTGAATTGAGGGTCTTTGGTGGCTCAGTATTAAGCCAACAGTAGAAGAATAACATTCTAATAAACTGCAACCAGGACAAGCAAAACTACATGCCTAAAATGATTGTGTTAAGATATATATGAATTTGTTGTTAGATACCTTACCAATGTTGTTGGGATCAAATGGTTTATTGGTATTTCCAGATCAAGGTAATGGAATTTTCTCGTAAATCAAAGGGATGATAGGATAATTTTGTGAGAAGAATCTAGAAAGTCACATAAATTAGGAAGGTCTTTGTTAGGTCACAAGGGACAACCTAGAAGTATGCTTTTAGTAGGCTTTTGGATAGCTTTTTGTTTCTGGATGTATGATCACTTTGGTATTTGCTGACTAAGACAACAGCCTCATATTCAGTAATTACTTCATTGTCTTTCTGAGTTTCTGTTTGATTGGATAATTCCCACACCTAAACCTTCCGCATTAAAGCCATCATGTTCCAAAAACAACATTGTCATAAGTGGTTATTAACCTTTATGATTTTTTTTTGAAAGCCAACCTTTTATGAGTTAGTTATTAATAGTTGTAACATATATTTGGAAATGTTTAAGATATTGATGGGTGTAGGTACTTTTTGTACCGAGTACAATAATGCATGACATGTCTGGTTGCATTAGGTATTATAGGACAAGTGTTACTCACTCCGGAAAAACATTAGTAATTTTTCATGTTAAGGGCTTGGATCATGCTGAGTAATAATTGGGACCTCCGAATTTCAAGTGGCTTGATCTTTCAAAGAAGAACTTAGGACTTCCGGGTTCAAATGGCTTGATCTTCAACGGGAGGTAAATCATGGAGAGGCTTTTACTTTCAAGATCATGGGACTTTTTATTTTTATAATCATGATCTTTAAATAAGCTTCAAAGCTATTTAGGATGTATCCGAGTGTTTACATAATTTTCCGAGATGAATACATACAAATGAGATGTCCTATTTATAATTGTAAGATAGAAAATTTTGATAAGTTCCAACTCTTTTCTAATTAAACAAATTAGATAATATCAATATATCCAAAATATATCCAAATATTCTAATATTAAAAATAATCAAAATTATCAAATTTTTTATGTAAACAATAATATCAAATTTCTTCTTCTCTTCTCCTTTGTCTTCTCTTTCTCAACCCCAAATCTCTCTTTATTTCCTGCTTTTCACCCTAAGATTGCTGCAGGAATATCCCGCTCCTACATGGCGCCTCCTGGCCCTGTAAATTGTCCACGTCATCGAAACAGAACTAAACTTGCGAAAAAAAATCTTTATCTCATCACTGGTCCTCTGATTTTCCAGTTCTTTTCTCTTTCCACCTAAACGACCTCTCTGTTGCTAAGCTTTCTCTGCAACTCCAAACCAAAATCCGAACTCTACTTTGAACTCTTATCCAATTGATTGTAATCTATAAACTCATTTTTTTTAAATAAATTTTTGATTAACTTTCTAGTGTTCTCCGTGTGTTTATTGGCTTGGATTTATGATTTTTCAAATGTAAATCCAGGTCTGGATTTGATGATTTCGTGGTTTATCGATAAGCTCTGAAGGTAAAGTTGTTCATCTGTATCCTTATCAGTATTCAATCTAGGCAATTTAAGTATGTTTCTGAAAATCTGAAGTTGTTTTTAGTATCTTCAAGATATTTTTGTCCATTAAGTTAATTTTTACGTAGTAATGAGAATATGAGATGGTTGATGATATAGATTTGATTAATCTGCAACTTGAGCAGTAAAACTGTTTTGATTGTTTTGCTGGTTATATGGATATACACGGAGAAGGATAGTGTTTTTTGTGTAGGTTAGGGTTTGAGAGTAAGTGAAAATGAACTATTAGCTAATACTCTGTTGTGTTCAAATGCAGGGTGGGTGAAGAGCGATTGATAGGGCTTGATGGAACTGTTGTGACGGTTTGGTTTAGTTTTGGTTTGAATTTTTAGGTGAATATCCAGTTATGATTTCGGATACAGTTATTATTGTTATTCTATTGCTCTTTAACCTTTGAAATGTTTTGAAAGATTTGTAGTTGAGGAATTTGGATCTTGAAGAGGAAGCTATGTGCTGTTTGTTTCCATTGACAAGGATTTGTCAAAGGTTTTACCCCGCAGTTCGATATCACCAACTCTTCCTTACCATCAGTGTTTTTGAATCTGGTTGGCAGGGAGTGTTTCAATGACCACCATTTCTGAAGGAGAGAAAGATCTGCCAGATGAAGACAGGAAAGTTGAAGATGGAATTGTGTGTGAGGGACAGAATGCTAGTGCGGATGTTGAGTTGAAGGTTGAGAGTGTTAGTAAAGATGTAAATGATGAAGGTAGGAGGGCATTGCAAGCCCAAGGTGCTATACAGGTGCAACAGCAACAGAGTCAAAGTGGAACAATCTGTTGGGAGAGATTTCTTCATGTTACGTCCATTAAGGTGCTGCTGGTAGAGAGTGATGACTCCACAAGACATATTGTCACTGCTTTGCTCCGGAATTGCAATTATGAAGGTTAGTTCTTTCGTTGAGAAACTGATTGAACAAACATGCTGTATATTTGAACTGGAACACTAGATTCATGGTTAAGCTCTTGGCTTATTAAATGGTGAAAGCTGAAATGCAACTTGGTTTATTGTTTATACTGTCATGTATATTTCTGTGTTATTTAATGATTAAATCATCAACTGGTACTAAGGTCTTCCCCAATAAGGGGATTTTACACAGTAGGAGTGTGTGGTGATGTGGAGGAGTGATAAGGGGGAGAGAGAAAAAAATGATATCTCCTTCAAGGACCTGTTTTTGGTCGGAATTGTGAGGCCCATCTTCGAAAGCAGCAGACCGCCTAGTGCACATGCCCCTTGGCGCGTAACTATGTTTATTTTATTTTATTTTTTCAGTTTAATATTTTGTTTTATTTTTTCCTTTTTTTTTTTTTTCCTCCACTCCCATTTTCTCTTTCCTAATCACACCTACAAAAACTCCTCAAAAACCACACCAAAATCTCCACTATTGAGGAAGGCCTAATATCTGTGATTATGGGAATATGCAGTTACTACACATAGGTGGAATGGTGACTAGATGTGTACTGTATATTGATGGCCATTGAGTGCTTAGCCATGAAAGGATCTTTTGACTTGTCACCATTAGACTTGTATTCCAATTTCCAATTGATAGTCTCTGTAGTGTTTATTACAGGATGACTCAGTCATTGTGATTCTATTCTGTAAAACTATCTTCATATATGAAACTGGATCCGTATGAAGTGGCAATCAACTGTTATCTTTCTTTTTCTTGATTAGTATTCTGATTGATGCTTTGCCCTTCATGTTCCTATCACTCAATAATCCACCAAACTTGTGCAGTGATTGAAGCAGCCAATGTATTGCAAGCATGGAGGGTTTTAGAAGATCTAACCAACCATATTGATCTTGTGTTAACTGAGTTAGAAATGCCTTGTGTTTCTGGCATAGTTCTTCTTTGCAAAATCATGAGCCACAAAACACGCAAGAACGTTCCTGTTATTAGTGAGTAATTTCTTTTGGCACTCTTGAACTTCTGATAGTGCAAAGGTTTTATTATTGTGTTTGTCATCGTCAATTTGATTTCATTAACCTCCACAGTTAATACGGGTCATTTGCAGTGATGTCATCTCGAGATTCTATGGGTTTAGTGTTCAAGTGTTTGTCAAAAGGTGCAGTTGACTTTCTGGTAAAACCTATTCGGAAGAATGAGCTTAAAAACCTGTGGCAACATGTATGGAGGAGATGCCACAGTGTAAGTGACTATTTCTGCACTTTCAGCATCAGTATCATAAACTTTGTTTGCCTACAAGCTTGTGAAAAAGGCCATCTTTGATATTCTGGTCTCATTTTATCATTTGTTAAGATTAAAAATAATTGCTTTGTCTACCACAGCCATTTTTTTTTATCTGAAAAAAGGAAAAAAGGAAGGGAAAGAAAGAAGAAGTGAATATAAACTTCTCCTTTTTCCCTCGATAAAATTTAACATTGTACCTTTTCTATATTTTATTTTCTTTGTTCATTAATTAATGTTTGCTTTTCTCAGTCTAGTGGAAGTGGGAGTGAAAGTGGAACACAAACCCAAAACTCTGTAAAATCAAAGAGCATTGAGAAGTGTGGCAATAATAGTGGCAGCAGTGATGGAGAAGACAATGGAAGTGATGGTTTGAATATTGGTGATGGTAGCGATGATGGAAGTGGCGCTCAGGTTCCATAATTTCCTTAGTCTTTATCTTTATAATAATGTGCCAACTCTTTGTTTGAAAATTGATTGGGATGCTTCTTTTAACTTATTAGTGTTCATTTACATACGGAAGAAAAAGAGTGCATTTGGGTTGTAGATTTACCCTTTACAGCCACTTACTCTTAAACAATAGAAATTTTCATTAGCTTAAATCTATACCCTCCATCTGAGATGAAAGCTCCTTTGCTGGGTCTGTGTGCTTCATATCTAGTTATCGACATTGGGCATCAACGTCTCCATATCAAGCCAAATAGTTTTGGCAAGTTAAGGGAGTCAGTTGAATACTTGGGATCGAGAATGATGATCAATAATGGTGTGCTTGGAACCCATATATTGTTTTTGAAGATGGAGCAGAAATTGAAGGCAGAGTTCTCCTTACAATTTTAACATCCATGAACTGCCTGCACTTTTCTCCATGAAACTGATTCTTACATATTACATTAGAGGATCTATAACTAAGTGTATACTTTCTTTTTTGGGGTGAACTGTGTGCTGCGGTTATAGCTGATAAATGTGTGACTTATGGCGAAATGAAATCTGAACATAATATTGACTTATAGATTACATACTTCTAGTATGAAGAAGTGTTACAATTTCCAATGTCTTGAATACTTTGTTGGCTTGGAAGGATGAACAAAATGCTTCATGCCGAGTTATATAGAAAACTCTCGATTGACATTACTATCAGTTTACTATATTTGATAATTGAAATGATGCCATGTTCTAGTGCTGAGTGAGTGCTGAAAGTCTGTTGCTGAACATTTTTGTATGACATCTTTAGTCTTATGTTCTAATCCTTGTCTATCTTTAATATTCTAGTCTCTGCTGTATCTGCAGTGTTTCTTAAATTTGCTGTTGGATTTAAGAAACACTCAGTTCTTTTTATATGTGCCACAGCGCCAGTAACCAAAACCAAATGTATTCATTCCAACCTACCGAAACTGGACCCATACATTTAATCTGTGAAAGAAAGAGTGCAGGACACTTGGTAACATGATTTTAAAATGTAAGCATTCAATTTTGCAGAGTTCATGGACTAAGCAAGCTGCTGAAGTTGATAGCTCTCAAGCAGCATCTCCATGGGATCAGGTAACTGAATGCCCAGACAGCACTTGTGCGCAAGTTATTCGCTCGAATGCTGAAAATTCCGGTAACAGGAAGGTGCATGTTGCTGCAACTAAAGATTGCCAAGAAGAGAAACAACCTGGTACCAATGGATTTTTTTCCTAATTTGTCTGCTGTATTTGTTCTTCCTTGATTTACTGATGTTTACTAAATTATGTGTGGTTCTTTCAGACAACACAAAATGCAAATACCCTGCCATGACCATACCAAAAAAGTTGGAGACACAATGTGAAAATCCCATTGGTGCTCCAATCAATTCTGTGGGTGAAAAACATACGAATATGGTGGAAATAGATCCCAGTGCAAGCAATAATAGAATAGAAAAGGAACAGGTAATTATTGTGCTTGTCTATCAATTATTATTTATTATGTCGTCAATACTAACATGCCTCATCCTTAGATTGACAGGAAAGAATTTGAAGCACAAAAAATGGTTGCAGCTGTATCAGAAATTGAGAATAACACCATACATGAGTCAAGAAAAGCAGTTATTGAGCCAAGTTTAAAGAGGCTGAGAGAAATGAAAGAATCTAGAGAGACCTCTGAAGATGACCGTTGTGTTTTCAGGCGTTCAGAGCAATCAGCATTCACAAGGTTTGGTGAAAATTGATTTAGCTGCTTTCTATACTTTGTCTATATGTCATGCAGGAATATCCTTAGACAAGATTTTCCTTTTCTTGGTTGTCACTTTGCAGGTACAATACATCATCACAATCAAATCCTTTGAGAACTCCTAATGGGTTGACTGGAAACAGTTTGGTGATTGACAGTGGACTAGAAAGTGCAAATAATGTGGTTAGCAATAACATAGATATGGGCTCCACGACTAACAAGCTTGCTACAAAACCACTAACTGTTCAAGATAAATCAGAAGCAACGTGTACAACTAATGGTTTGCATCCCTCCTCAGCATACAAACCTGTGAAAAACGATTTCAGGAACTGCCAATCACTAATTAAGACTAGTGACATGCAAGCTACAACACTGCTGGCTCCAAGCAGTTCTCACACAGACATCCCGGATCAGCACCTTCATCATCACAACAACCACTATCCTCGCAATAGTCATCATTTGCACAACCAAGAACAACAGCCTGCATCAAACCATGATAAATTCTCACTGAAGCAATTGACAGCTAATGCTCTTAATTCTGATTCTTCGAACGTGATGGCTGGGCCTTTTGAAGGCACTCTTGGAAATCATAGCTTGAATAAAAGTGCTTCAGGAAGTAATCATGGAAGCAATGGACAGAATGGGAGCAGCACAGCTGTGAATGTTGGAGGCAACAATGGGAAGACTGAAACTGGGCTAGATGGGAAAGGTGGAAGTGGAAGTGGAGATGCAAGTGGCAGCGGTTCTGGAAGCAGAATGGATCCAAACAAACTTGCACAAAGAGAAGCTGCTTTGTCCAAGTTTCGTCAAAAGAAGAAAAGTAGATGCTTCAAGAACAAGGTAGGTAACATGATTTAACTTCAAACTTGATTTCAGGTTCAATCTTCTTGTTCTAGTAGAATTGATAACACATATGGGACACATCACACGCATACACTTAGTAAGACAACTTGATTTGTTGTCCTTGGTTTAGCTAACAGATGTGTGGCAAGCATAAGAATGCATAAGTAAAATACAGCGAGTATAGTTATCTACTTTTTCTTTTTTCAAGCTCTAAACATAAAGGAAGACATGTAAGAGGGGCAATGCTGACAAATGGTAAAATACTCATGGTTGTTGGAGAGATTTTAATTATATGGGGTGATAAAGAAGGGTAGTGGATTGATGGATAGTCTTTTGGTTAGTGTTGACCATGGCCAGCAATGTGTTGGAAGTTTGAACTACCACATATAAGATTGAAACTTGGGTTACTAGTTTTGAGGAGATTTCTGCAATTTATCAATCTAGTTTCATCTTTTGAATTTGTTTGTTTGGTAAGGTTCGATACCAAAACAGAAAGCGACTAGCAGAGCAGCGGCCACGCATTCGAGGACAGTTTGTGCGGCAGACGGGGCAAAACAACCCTAG

>g20699

ATGAGGTGGGAGAGGTTTTTGCCGAAGATGGTGCTCAGAGTTTTGTTGGTGGAAGCTGACGATTCTACTCGGCAGATCATTGCTGCTCTTCTCAGAAAATGCAGTTACAGAGGTTAGTTTGATGTTTTACTTAGAAAACTGTGAGAAACCTTAATCTTGATCAAGTTGGGGGTTGGATTTGGGGATTACTAAAATGTTTTTGAAATTTAGTTGATGGGAAAGTTGTTGAATTGCAGTTAGTGCCCAGAGGTTATAGATAGAGAATCTATGGAAGGTGAAAATTGGAAGCAAAAGATCACTGTTTTTTTTGCATCTAAAAATCTGATCTTTTTTTCTGGGATTTCGGAAAGTCATTTGTTGTGTTGTGGTGGCAGTTGCTGCAGTTCCTGATGGTCTGAAGGCATGGGAGGTGCTGAAAGAAAGGCCCAGCAATGTAGACCTAATCTTGACAGAAGTTGAGCTGCCATCTATCTCTGGATATGCTCTTCTCACCTTAATCATGGAGCATGAAATCTGCAAAAACATTCCTGTGATAAGTATGGACCATCCTAACCTAGGATTATCTGCAATTCTGCATAAATAATGTTGATTTACACCTAGGAAAAATTGCATTTTGGTTATCAGTTATGACCGTGGTACAGACTTAATCCTGAGTTCTACGTGTGACCTGCATAAAGTGGCGAGTTTAGACTTGAAGGACCAAATCCGCCACTTTGTTAAGTGTCAGAAATGGTCCCAAGGGATTAAAATCGGGGTTTAAGATGGTCACACATATAACTGAAGGACAAAAAATGCAATTTCCCTTACATCATCTATATAACTGCCCTTTTGTGGACTGTGTTTTGACAACTTTGTTCCTGATTATGCAGTGATGTCTTCGAATGATTCGGTTAGTATGGTGTATAAATGCATGTTGAGAGGAGCAGCAGACTTTCTTGTGAAGCCTGTGAGGAAGAATGAGCTGAGGAACTTGTGGCAGCATGTGTGGAGGAGACAAGCTGCAAACAGAATTGATAATCTTAATGGTCCAGTTTCTCCTACTAGGAATGAGGATTGCAATGAAAAAGGGAGTGATGATGAGGTAATAATTTATAATCAATCATGTTTCAAGAAATCTGTGATGTGGGGTGGGATTGTCTATTCACTGGATTTTGTGCAGAACTCGTGCGTTAAGCTCGAGATGGAGATCGGGGAGAAAACACAGAGCACGTCGAGGAATCTGAACAGAGATTTCGGGGCAGTTCTCTTCCTATTCATCCACAGAAACAAGAACAGGAGGATCATAATCGAGTTGGAGGTAAAATTATTCATCTTGAACTCTAAGGTGTTGTTCCCTTGCAGTGTTTGATTGTGACTGTGTTTGGATGTTTTGAATTTTGATAGATGGTGATGATAATGTTAATGTTAATGGTGATGAGGATGATCCTGGTTACCTGAAATCTTCCAAACAAGCCATTGATTTAATTGGAGCGTTTGATAATTCCCCGAAATGTGATTACAGAAGTTCTTGTTCTAAAGATAGTGCAGACAAGGTTGAGTCTTTGCCCCCATTGGATCTTTCCTTGACAAGATATCCCAGTGGCTCTATGAACAGATTAAACCACTCAGATGCGTCAGCTTTTACACGGTGAGGTCTCATTATCGCCTTCCTGTAGGCTTTCTGCAGCGTTTTTAGACGAGTGTGAACTTTGTCCAACACGCGAGGAATGTGTTTCTGCAGGTACATTAACAAAGGCGTGCAGCCCAGAAACTCGATGGCCCCCAAGACTTGCAATCAACACGAGGACTGTGGAACCGATTCTGATAAGCACCTGTCTGTTCACAATCTCGATGGTTCAACGATGAAATTCCACACACTTATGCAACAGGCGAGAGCTGAGCCGGGGAACAACGAAATTGGGCTTCCGATTCCAGTGAGAGGTGTGGGGTTTAAGGGTCTAGGAAATGCCCACAGTTCCATGATGTCTTCTCCTATGCAGAGCCCCGGTTCAGCAGGCTGCCCGGATTCTCAATTTCAGACGCCTTTGTTTCATCTATTAAACCATCAAGCCGTGAGTTTTCAGCAGTCGTGTGGGCTCGTGGATCAGAACACTGATAACGACACCAGTCAGTCCGAGAAAAAAGAAGAGAACACAGATGATCACGGACATTTTTCATCGTTTACTGATCAAAGTGCAAATAGCAGAACCTCGGTTCCATTAATTAAATCTACAGCCGAGTGTGAAAAAGCTCCTCTTGGTCAGGACGGGAGCTATCAACAATCTCAACGAGAAGCAGCTCTTACCAAATTCCGATTGAAAAGGAAAGACAGATGCTTTGAAAAGAAGGTATATTACCTTTTTGCAGTAGTTTCAAGAAATCACCACATCTGCTGTATATATAATCACCAAACACGCGTTTTATTTGCTCACAGGTAAGATACGAAAGCAGGAAAAAGCTTGCAGAGCAGCGCCCTCGAGTGAAGGGACAGTTTGTTCGCCAACAGCCAAAGTGACGCGAGCTGGTTAGTGATTAGAACTTCGATAAAAGCTTTCCAACACTATAACCCGATGTTGGGATGTTTCTTATGACTTGCGGATAGGGAATTCGCTGCATTCCTGTGAGAATGATGCCACCAATGTTTTGTTTGGTCTTATAATATTATGATAAGCTTTTGGAATTTGACCTGTAGGCTAGCCACTAACCAGAACTCCGATTCTTTCATTATAAACGCTTTCATGTCACCGCTTCTTATAGCCATTAAGTACTGTAATTTTAAGGTTCAGAAGTAG

>g871

ATGGGTGAAGTAGTGGTGAGCGGCGACGGCGGCGCCGCCGCAATGGAACTGGAAACAGAGGAGGTGGAAGTGGTGGAGGCGCCGGCTTCCGCCGCCGCCGCATCGGCGGTGAGGTGGGAGAGATTCTTGCCGAAAATGGTGCTCAGAGTGTTGTTGGTGGAAGCGGATGATTCTACAAGGCAGATTATTGCCGCTCTTCTCAGAAAATGCAGCTATAAAGGTTAATTTTTCAATTATTCAGTATTGGAAGATTGTTGTTTATGTTTAAATTAAGTTATTAATTTATTATTATAATAGGAATAGGAATAAGAAATTTAGGTTTCTGGAAAGTTCTTTAATTTGTTATCCAAACTATGTATTTGGAATTGGAAAGATCTGTGTTTTAAAAATCTTGTCTTTATAGGTTCTGAAACAGTTTAAATCTTGTGGTTTTGCAGTTGCTGCAGTTCCTGATGGTTTAAAGGCATGGGAGGTGTTAAAAGGAAGGCCAAGAAATGTAGACCTTATATTGACAGAAGTTGATCTGCCATCAATCTCTGGATATGCTCTTCTTACCCTAATTATGGAGCATGAAATCTGCAAAAACATTCCTGTTATAAGTATGCTTCAACTCTACTTTACTGATTTAATTAGCATTACCAGCAGAAATGGTGTTCATAAACCCTTAACTTAACGAATTCTACCTCGATTTACAGTGATGTCTGCACACGATTCGGTTAGTACAGTCTACAGATGCATGTTGAGAGGTGCAGCAGACTTTCTTGTCAAGCCCGTGAGGAAAAACGAGCTGAGGAACTTGTGGCAGCATGTATGGAGGAGACAAGCTGTAAGCAAATCCATAGCCTGTGATTCCTGTGAAGTGTAAACTTTCCTCCAAAAGTTCCTTTTCGGGGAGGATCTCATTTTCTTTTTTTTTTTTTGGTCTCTCAGACGAGCAAAAGCGGGCAAGGACCTGGTGATGAGAGCATGGCGCAATTGAAGGTTGAAGCCACAGCTGAAAACAATGGTTTTAGTAATCATTCGAGCGGGTATAAGGCTTGTATTGAAAGGAACAGAGAATGCATTGAAAAAGGAAGTGATGCTCAGGTAAGCACATATTCATTGCTATTTCAGTATATTGAAAGTAGTATTCAAGTCATTTTAGCCGTAGGATGTTTCTTTGATTCCTTGATCATGGTCTACTGTTTTACATGTGAGATTGTCTGTTACCGATGTGTGTGCAGAGTTCTTGTACTAAGCCAGAGATGGAGACTGGAGAAGAAAACACGAAACATATCCAGGAATTCGGACAGCCGGATTGGAACAAACCTCGTCCAGCTGATGCAGATATGCAGAAGGAAGAGCAACATCGCGATGCAAGCTCGAAGCTGAGGAATCCCAGCGATCAAGTGGAAGGTAATGTTGTTTGACTGTTTCTCGTGAATCCTATGATGTTCTTTCTGTTTGATAGTGTCTGACTTCTTAGATGTTCTTACAGGCACAGGCTATAATGCTGCTACAGTGGCCAGTGGGGAAGACAGGAGTTCTAATGAGAATTGCTGTCATCTTCAAGTGATTGGTCAAGCTTCTGATGAAGATCCTGCGATCATGAATTCTTGTAAACGCGCCATTGATTTGATTGGGACCTTTGATAATCACGGGATATGCACTTACATTTCTGGTTCGAATATTAGTGCGAACAACAAGGTTGATTCTCCACCCCGCCTCAGCTTTCGTTGACAAGGTATCCTAGTGGCTCTGTCAACCAGTTTCCAGATGAGAAGCACAAGTTGAACCATTCAGATGCATCAGCCTTTACTCGGTGAGTTTTTTGCATTCTTGGCGCGATATATTTGGAACATTGGGAGTTGCCTAACACGCAAGAAACATGTATGCAGGTATGTGAGCAAGGGGGTGCAGCCACGAGACTTGATATCCCAAAAGAACAAAGAAAGCGAAACTGACTCTGATAAGCGCTTGTCTGTTCACCTCGATTGCAATTCTGATACTCATGGTCCAACAGCAAGCTCTCACAGACTTGTTCCACCGACAAATTTTGAATCTGGACGGGCTGAAACTGAACTTCCATCCCCCGGGCAGAGAGTATTGAGTGCCCCGATTCCAGTAAGAGGTGTGAGGTTTGAGGGGCTAAGTAACGCCTACAGCTTCATGACATCTCCGATGCAGAGCCCTGGATCGGCTGGGCATCAGAATTCTCCACGCCAGGCAAACACATTTCATCGCTTGAATCATCAAACTATAAATTCTCAGCAGCGTCACAGTGTAATTGAGCACAATGTCAATACTGTCTCCACTCAGACCGAGTACAAACAAGGTTATCAGTCAGAACCTGACCGGGGACATTTTTCTTCTGCTACTGATCAAAGCGCAAATAGCAGCCTGTGCAACGGTGTTGTCAACTGCCATTACACTGGTGGTGGTGGGAGCAATGGCAGAATCCCGGTTACAATGATCAAATCTACAGCAGAATATAGGAACGACGAAGCTTCTGTTGTTCAAGATGCAAACTCTCAGAGGTCACAAAGAGAAGCTGCACTCAACAAATTTCGCTTGAAGAGGAAAGACAGATGTTATGAGAAAAAGGTATGCTATAGTTTCCAAAAAAGAAAAAGAAAAAACTCACATGCTATTTCTTCATATGTTAGGAAGAAAAGACTGACCAATCTTTGGATTTGGACACAGGTAAGATACGAAAGCAGGAAAAAGCTTGCAGAGCAGCGTCCACGAGTGAAGGGACAGTTTGTTCATCAATTGCCAAGTGAACCACCACCAGGAGACACATAA

>g27243

ATGGAGTTGAACGAAGCTAATGAGGCAGAGAGGAAGAGGGACGACGAAAGTGGTGGTGGCTCTGCGGCGGTGTTCAGGTGGGAGAGGTTTCTGGCGAAGATGGCGGTGAGGGTTTTGCTGGTGGAAGCTGACGATTCTACTCGCCACATCATTAGTGCACTTCTAAGGAAATGCGGTTACAAAGGTTGCTCTATATACCTTCCTCTTTCCATAATTTTCTTCTATGTTGAATCGAGTTAGTCAAAAGTTAATATTGCATTGTGAATCTTGTTCAAAAACTTATGTGTCTGCATTTAATGTATTTTGTACATATAATTAACCATTCATATAATTGTGAAAACACATAACTGAGTAATTATAAACACATAACTTAATAACTACGTACACATAAATTGTTAACTGCACGTATGTTAATTATTTGCAGACACAAAAGATGTTTGTTAACATGTTATGTGTTTGCAGTTAACATATATACTTGCAAATTATCACGTGATACATACTATGTTCAATTTGTTTACAAATACGAAAATAATTAATTGCTGACACGAAATATGAAAGTTATTTTTGAAACAGGGCTATAATACAAGTAAGTCTTAGTAGCTTGATCCATGGTATAATTTGTCAGTTAGGCCCTTGTCGGCCATAATATATAGATATAAATATAAATAATAAAACTACAATTTTAGTATCGGTTACTCGGTTAGCATTGAGTTTTGAAGAACAATATATGTACGTGTGCGTATGTATATATACACAGGAATATGTATAATGTATTGCAGCAAAATATGGCCATGGACAGTGACCAGTGACCACAAGTCTGTGGAAGAACATAAACATTGAACAGTGCCGCAAATGATCAACTGAGATCCATGTTTAACGGTGTAGCTAACGGTTGGATTGAAATTGAATATATGTTTTTTTTTTTTTTTTTTTTCAATTCCATGGACCACATATATCTATCCAAGTTGAAAGTTGTGGATAAGATCTAAAATACATACTTGTGGGACCGAAGTGAATTTAATTCTTAGGAAGAATTGGTGTGAGCTTAATGACGTATTTGGTAGGATGCAGTTGTAATTCAATTACAATTTAATTTTTTGTGGAGCATAACTATATTGTTTAGTTAGAATAAATTATAATTTCAGGTAATTGCAATTCTTATTGCAATTATTTTAGACAAAGATATTTTAATTTTTATATCATTTATTCCTTTTCTAGTAATTCTATTTCAGCCTACCAATATAATTTGAATTCCTACTTTATTCCCACCTTATTTTTTTCTGACGGAATTATAATTCTTTCTCGGTTAAGGATGTTGATGTGTAATGGATTGTGTTATATTAATAAATGATAGATGAAAGAGAAGAAGACATTTTTACAGAGTTAAGAGATAATGTTAATTAACTATTTGTATGTATTCTGGCAGTTGCTGCAGTTTGTGATGGTTTGAAGGCATGGGAGGTGCTGAAAAAAAAGCCCCATAATGTGGACCTGATTTTGGCAGAAGTTGATCTGCCATCAATCTCAGGATATGCCCTTCTCACCTTAATCATGGAACATCAAATCTGCAAAAACATCCCTGTAATAAGTATGCCATCCACACCCCAACTTAGGATTGCTGATATGATTTACTAATTTTACTGAGTCTCGTACCTTAATGCTGTGAACCTGGTTGTCCAGTGATGTCTTCCCAGGATTCGGTTAGTACAGCTTATGGATGCATGTTGAGAGGGGCAGCTGATTTTCTGGTCAAGCCGATTAGGAAGAATGAACTAACAAACCTGTGGCAGCATGTCTGGAGAAGACAAACAGTGAGTATAGTATAGATTCAAATCCTATATATATATATGCATCTCCACTTCTAATAACTGATAATGTGGTGGAATTGGTGTTGTTGTCTCAGTTGAGCAGTGGAGCCATGGTTGAAAATAATGACATTTCTAATGGGGAATGCAGGGAGAAAGGAAGTGAAGATCAGGTAACGCAGTGTTAGGATTAAGTGTTTACTACTATGTCTTTACCCTCCTAGAGAACTGTGACTTAGCTCTAATACTGCTTTTTAAGATCAAGTGCTTATCACTATGCTAAAAGTTGTAGTTCATAGCAAAGACACAACTTTATTTCCTTATATCGCCAATTACATTTTCTAGAGAGGGTCTTTGAACTTCCCTAGCCACCAATTCTGAATTTGACTCTTTACTGACTTCTGTCCAACACACACACATATTTTCAACATCAAATGAGAACCATGCCTTAGAAGAAAAGTTACGAAACCTGCATGAATGCACACAATCTATTCTAGATAGATACTGTGTAGTGTGCACCAGGAGTATTAGATTGTGTGCATTCATGCAATAATTCGAAAATTCTCATTGAACACAAGCCTGTATATATATCTTGGCTTCAATTAAATTCTTGTACATGAAGATGATTGAGATTGTGTGACATGTGCAGAGCTGTTGTTCAAAGCCAGATATAGACAGTGAAAGAGAAATGACAGAACATATCCAGGATCTTTTACAGCCAAATTGGGACAGATCTCTTCCTATAGTTGATCAAGCCTCTGATGAAGATATAGAGTCTTGCAAACAGGGAATTGATTTAATTGGAGCATTTGATGACTACCTCAATTGCAATCACATAAATCCAAGTCCAAATACTAGTCCCAACAAGCAGGTTGATTCTGCTTCACCCGAATTGGATCTTTCCTTGACAAGAACACATCCTACTTCCATGCTGAATCAATTTGTAGACAATCATAGATTAAACCACTCAGATGGATCAGCCTTTACACCGTGAGTTTTCTGTACATACCAAATTGAAGTCATAGGATAAATCGTTTACCACTAAGGCAAAAGTTATAACTGCTTTATTTCCTTTTGGGTGACCATAACATTTAGTGAAGACCCACAAAAATGACCATCACATTTTTCAGAGGCATGGTGCTGAACTTGGCCTCGAATCTGTGACTTGACTCTAATATCAATTTGATAGGATTAAGCTCTTGTGCAACTAGGCCAAAAATCATAGCTAGTAGCAAAGATGCAACTTTATTTCTTTATACCGCTATCACATTTTTGAGAGGCATGGTACTGGAATAGGCCTTCACCAGCCTCCGCCTAACGATTCCCTAGCCACCCATTGATGGACTTGACCCTTTACTGTCTTTGGCCAACAGAAAGATTGTTACTTCTTGACTCTACTAAAAAGTTTAGCTGAATTGAACATTCTTAATGAAACCCTTGGCAGTATTATGAAATTGCATAATTATGAGGAATATGAATGTGCAGATATGTTAACAAGGGGACGCAGAAGCAGCGAGGATTAACAATCCCTATTGGTGTGAGGTTTGAGGGTGCAAGCAGCCCTGTGATATCTCCAAGTGATTCCCCTGCTTCTGGCAATCTAGAATCTCCTCCCGGCCTTGACCCAAATGCAGTTCATCCTATTCCTTCCACAGCTGATGAGTGTAGGAAGGAAGAAGTTTCCCTGGCTCAAGACGGAAACTCTCAACGATCATCTCAAAGAGAAGCAGCTCTCACCAAATTCCGCTTGAAGAGGAAAGACAGATGTTTCGAGAAGAAGGTAATTAATTCAACTAGTGTTTCAACTTTCATGTTTCTTTCTTTCACAGTTTCACATTATTATCATTCATATGATGCGATAGGATCAAATGCTTACCACTAGGCTAAAAGTTATAGTTTGTAGCGAAAGTAGACTTGATCCTTTAAGCCTTTTACTAGCACTCAGGAGTGCTGGATTTGCCTTTATCGGCTTCCAACAATCCTTCCGCTACTAGATGCTAGATTTAGGCTCGCTTGGTAAACATAGTTAGTTTATCAGCCAATTTTGATTATTTGACTACTATTAGCTGTTTGACTTGATTAAACAATTAATATGTGTGTTTGATTAATTAACTTTTTGTAATAGCGTATTGCTCAAAAATGTTAAAATTCAAAAAACTGCTTAGTTTTTTCGATCAGTTTTTTGGAAAACTCATTTTACATGTAATAGCTATCAGCTAACAAGCTAATTACCAAATACTTTTCTGCAACCAGTTAATGTTTTCAACTAGTGAAACCCATTAACCCAATCAACTAATAGTTATTTATTAATAACCCCTTAGCCTTTAACCGCTTCTGCCCAACATGATGGTTTTGATAATAGCTGCAGTGCAAAACAACTTTTATGAGCTAAAGATTGATCTTTCTTTTGGGGGATGTGTTGATAGGTAAGATACGAGAGCAGGAAAAAACTTGCAGAGCAGCGGCCACGGGTGAAGGGACAGTTTGTTCGCAGGCAGCCAATCGGCACGGGTGACAATGAGATGATTAGTGCTGTAAATCAATAG

>g27036

ATGGAGTTGAACGAAGCTAATGAGGCAGAGAGGAAGAGGGACGACGAAAGTGGTGGTGGCTCTGCGGCGGTGTTCAGGTGGGAGAGGTTTCTGGCGAAGATGGCGGTGAGGGTTTTGCTGGTGGAAGCTGACGATTCTACTCGCCACATCATTAGTGCACTTCTAAGGAAATGCGGTTACAAAGGTTGCTCTATATACCTTCCTCTTTCCATAATTTTCTTCTATGTTGAATCGAGTTAGTCAAAAGTTAATATTGCATTGTGAATCTTGTTCAAAAACTTATGTGTCTGCATTTAATGTATTTTGTACATATAATTAACCATTCATATAATTGTGAAAACACATAACTGAGTAATTATAAACACATAACTTAATAACTACGTACACATAAATTGTTAACTGCACGTATGTTAATTATTTGCAGACACAAAAGATGTTTGTTAACATGTTATGTGTTTGCAGTTAACATATATACTTGCAAATTATCACGTGATACATACTATGTTCAATTTGTTTACAAATACGAAAATAATTAATTGCTGACACGAAATATGAAAGTTATTTTTGAAACAGGGCTATAATACAAGTAAGTCTTAGTAGCTTGATCCATGGTATAATTTGTCAGTTAGGCCCTTGTCGGCCATAATATATAGATATAAATATAAATAATAAAACTACAATTTTAGTATCGGTTACTCGGTTAGCATTGAGTTTTGAAGAACAATATATGTACGTGTGCGTATGTATATATACACAGGAATATGTATAATGTATTGCAGCAAAATATGGCCATGGACAGTGACCAGTGACCACAAGTCTGTGGAAGAACATAAACATTGAACAGTGCCGCAAATGATCAACTGAGATCCATGTTTAACGGTGTAGCTAACGGTTGGATTGAAATTGAATATATGTTTTTTTTTTTTTTTTTTTTCAATTCCATGGACCACATATATCTATCCAAGTTGAAAGTTGTGGATAAGATCTAAAATACATACTTGTGGGACCGAAGTGAATTTAATTCTTAGGAAGAATTGGTGTGAGCTTAATGACGTATTTGGTAGGATGCAGTTGTAATTCAATTACAATTTAATTTTTTGTGGAGCATAACTATATTGTTTAGTTAGAATAAATTATAATTTCAGGTAATTGCAATTCTTATTGCAATTATTTTAGACAAAGATATTTTAATTTTTATATCATTTATTCCTTTTCTAGTAATTCTATTTCAGCCTACCAATATAATTTGAATTCCTACTTTATTCCCACCTTATTTTTTTCTGACGGAATTATAATTCTTTCTCGGTTAAGGATGTTGATGTGTAATGGATTGTGTTATATTAATAAATGATAGATGAAAGAGAAGAAGACATTTTTACAGAGTTAAGAGATAATGTTAATTAACTATTTGTATGTATTCTGGCAGTTGCTGCAGTTTGTGATGGTTTGAAGGCATGGGAGGTGCTGAAAAAAAAGCCCCATAATGTGGACCTGATTTTGGCAGAAGTTGATCTGCCATCAATCTCAGGATATGCCCTTCTCACCTTAATCATGGAACATCAAATCTGCAAAAACATCCCTGTAATAAGTATGCCATCCACACCCCAACTTAGGATTGCTGATATGATTTACTAATTTTACTGAGTCTCGTACCTTAATGCTGTGAACCTGGTTGTCCAGTGATGTCTTCCCAGGATTCGGTTAGTACAGCTTATGGATGCATGTTGAGAGGGGCAGCTGATTTTCTGGTCAAGCCGATTAGGAAGAATGAACTAACAAACCTGTGGCAGCATGTCTGGAGAAGACAAACAGTGAGTATAGTATAGATTCAAATCCTATATATATATATGCATCTCCACTTCTAATAACTGATAATGTGGTGGAATTGGTGTTGTTGTCTCAGTTGAGCAGTGGAGCCATGGTTGAAAATAATGACATTTCTAATGGGGAATGCAGGGAGAAAGGAAGTGAAGATCAGGTAACGCAGTGTTAGGATTAAGTGTTTACTACTATGTCTTTACCCTCCTAGAGAACTGTGACTTAGCTCTAATACTGCTTTTTAAGATCAAGTGCTTATCACTATGCTAAAAGTTGTAGTTCATAGCAAAGACACAACTTTATTTCCTTATATCGCCAATTACATTTTCTAGAGAGGGTCTTTGAACTTCCCTAGCCACCAATTCTGAATTTGACTCTTTACTGACTTCTGTCCAACACACACACATATTTTCAACATCAAATGAGAACCATGCCTTAGAAGAAAAGTTACGAAACCTGCATGAATGCACACAATCTATTCTAGATAGATACTGTGTAGTGTGCACCAGGAGTATTAGATTGTGTGCATTCATGCAATAATTCGAAAATTCTCATTGAACACAAGCCTGTATATATATCTTGGCTTCAATTAAATTCTTGTACATGAAGATGATTGAGATTGTGTGACATGTGCAGAGCTGTTGTTCAAAGCCAGATATAGACAGTGAAAGAGAAATGACAGAACATATCCAGGATCTTTTACAGCCAAATTGGGACAGATCTCTTCCTATAGTTGATCAAGCCTCTGATGAAGATATAGAGTCTTGCAAACAGGGAATTGATTTAATTGGAGCATTTGATGACTACCTCAATTGCAATCACATAAATCCAAGTCCAAATACTAGTCCCAACAAGCAGGTTGATTCTGCTTCACCCGAATTGGATCTTTCCTTGACAAGAACACATCCTACTTCCATGCTGAATCAATTTGTAGACAATCATAGATTAAACCACTCAGATGGATCAGCCTTTACACCGTGAGTTTTCTGTACATACCAAATTGAAGTCATAGGATAAATCGTTTACCACTAAGGCAAAAGTTATAACTGCTTTATTTCCTTTTGGGTGACCATAACATTTAGTGAAGACCCACAAAAATGACCATCACATTTTTCAGAGGCATGGTGCTGAACTTGGCCTCGAATCTGTGACTTGACTCTAATATCAATTTGATAGGATTAAGCTCTTGTGCAACTAGGCCAAAAATCATAGCTAGTAGCAAAGATGCAACTTTATTTCTTTATACCGCTATCACATTTTTGAGAGGCATGGTACTGGAATAGGCCTTCACCAGCCTCCGCCTAACGATTCCCTAGCCACCCATTGATGGACTTGACCCTTTACTGTCTTTGGCCAACAGAAAGATTGTTACTTCTTGACTCTACTAAAAAGTTTAGCTGAATTGAACATTCTTAATGAAACCCTTGGCAGTATTATGAAATTGCATAATTATGAGGAATATGAATGTGCAGATATGTTAACAAGGGGACGCAGAAGCAGCGAGGATTAACAATCCCTATTGGTGTGAGGTTTGAGGGTGCAAGCAGCCCTGTGATATCTCCAAGTGATTCCCCTGCTTCTGGCAATCTAGAATCTCCTCCCGGCCTTGACCCAAATGCAGTTCATCCTATTCCTTCCACAGCTGATGAGTGTAGGAAGGAAGAAGTTTCCCTGGCTCAAGACGGAAACTCTCAACGATCATCTCAAAGAGAAGCAGCTCTCACCAAATTCCGCTTGAAGAGGAAAGACAGATGTTTCGAGAAGAAGGTAATTAATTCAACTAGTGTTTCAACTTTCATGTTTCTTTCTTTCACAGTTTCACATTATTATCATTCATATGATGCGATAGGATCAAATGCTTACCACTAGGCTAAAAGTTATAGTTTGTAGCGAAAGTAGACTTGATCCTTTAAGCCTTTTACTAGCACTCAGGAGTGCTGGATTTGCCTTTATCGGCTTCCAACAATCCTTCCGCTACTAGATGCTAGATTTAGGCTCGCTTGGTAAACATAGTTAGTTTATCAGCCAATTTTGATTATTTGACTACTATTAGCTGTTTGACTTGATTAAACAATTAATATGTGTGTTTGATTAATTAACTTTTTGTAATAGCGTATTGCTCAAAAATGTTAAAATTCAAAAAACTGCTTAGTTTTTTCGATCAGTTTTTTGGAAAACTCATTTTACATGTAATAGCTATCAGCTAACAAGCTAATTACCAAATACTTTTCTGCAACCAGTTAATGTTTTCAACTAGTGAAACCCATTAACCCAATCAACTAATAGTTATTTATTAATAACCCCTTAGCCTTTAACCGCTTCTGCCCAACATGATGGTTTTGATAATAGCTGCAGTGCAAAACAACTTTTATGAGCTAAAGATTGATCTTTCTTTTGGGGGATGTGTTGATAGGTAAGATACGAGAGCAGGAAAAAACTTGCAGAGCAGCGGCCACGGGTGAAGGGACAGTTTGTTCGCAGGCAGCCAATCGGCACGGGTGACAATGAGATGATTAGTGCTGTAAATCAATAG

>g53330

ATGATGGAGAAGAATGAGATTGTGAAGACTGGGGATGGGTTCATAGATAGAAGTAAAGTTAGGATTTTACTGTGCGATAATGATTCCAAGAGCTCTGAGGAGGTTTTCACGCTATTGTGCAAATGTTCTTATCAAGGTTATGAAATCTTCTCTGCTATTCAGTTCCGAGATAACTTTGCTACTATGATTGTTGTTGTTGTTATTGCTATTTTTTATTTTTATTATGCCTAAAACTTTTATGTTGCTGCAACATGTTCTGCATATTCTGAATTGGATTGAATTTAATTTTTCTAGTTCAGAGTATGTTTTACGAAGATAGCAGCTTAAAATGTTGTAACTACTGTCATTCTAAATCAATTGTAATTCGATTCATGATTACTGTGATAGATGATGTCTTTGTTGGTTTTTTGGTTTACTGAAATTTAGGTTTGGTTTTGGATGCATTTAGTTAGGTACTGCTTGCCTGAAAATTGGATTCTTTAAGTACAAGTGTTTTAACACATTAAGGAATTTGTTGGTTTGGGGAGAAGTAGGTCTTTTGACATGCTAATGCTCACCAGAAAGCACACCCTGCTGCTGCCTTACCCTAGAAACGGGAAAAAGAAAGAAATATAATATGTGGTTTCAAAGTCTAGGTACTAGGCTAATGAGTGGATAAATACGTGTTGGGAGAGTTATTTTTTATCATTATTTTTCCTTGCTAAATGTTTGTGAGTTGCATTGTAGATTCTTTAGTCTTTACTGAGTCTGCTTTTTAATTTGCAGTGACTTCTGTGAGGTCACCCAGACAGGTGATCGATGCATTGAATGCAGAAGGACCTGATATTGATATTATACTTTCTGAAGTGGACCTTCCGATGTCCAAAGGGCTGAAGTTGTTGAAATATATTATGAGGGATAAGGAGTTAAGGCGAATTCCAGTTATCAGTAAGTCAAACTTCATTATATTCCCATTCCCATTTAATTTAAAATAAAAGTTATTCAACAACTGAAGTAAAAATTTCTTTCTTATTTTCTTTGGTGTAGTGATGTCATCTCAAGATGAGGTTTCTGTTGTCGTTAAGTGCTTAAAATTGGGAGCTGCAGATTACCTTGTGAAGCCCCTCCGCACTAATGAACTATTGAACTTGTGGACTCATATGTGGAGAAGAAGGCGAATGGTATGCACTATTATACCTTCTATGTTTCATTTCCCTCTAGTGTTAGTTTCATGAAATATTAGGGTAACTAGAAAGTAGTTATCCTTATGCTTCTGCAGTAAATAGAAAGGCTATGGAAACCTTAGCCTGAGATTGGATGAGAGATAAATGCATCAGATATTATGTCTTCTATATATCAATATTTAGAGTAGCTACTTTAATTTCTTTTCTGTTATTTTATGTAGTTTTTTTTTTTTAATATTCTTCTTGACATACTTCATATAATCATTTCTGTATCTCATTTTGTTGACAGCTTGGACTAGCAGAGAAGAACATTCTAAATTATGACTTTGATTTGGTAGTATCAGACCCGAGTGATGCTAACACAAACAGCACTACACTCTTCTCAGATGACACAGATGAAAAGTCCCGGAAAAGCATAAATCTGGAAACTGGTCCCTCTACTCAACAGGAAGATGAGGTGAGTTATTGGAACTGAGTGTCGGTATTTGAATCTTAGTGAGTTCGCTTTAAGGCTATTATTGATGTTCTCGACATTCAACAACACCATACAAAGATTAATATTGAACCTAGCGTTGACCTGATTAGGTGGCTTGTCAGGAAATGAATGTGGGTGGGGGAGATTAACTCTGTGTTCTGGAATCAACTTGGTTATTGTTGAGCACTGAAATTGCATTTCTTTCTCAACTGGTTTGCACTTTTCAAATTTTGTTGGAAAAATTTACCAGGCCTGTTCAGTTTCCATTTTCTGGAATAATGGAAAGTAGAGTTTGTTAAAAATGAATATTTCTAGCCTTATTTTTCCAATTAAAATAAATTGAATAACTCCAAAAAGATGTTATTGAAATTGTATTATTGTGGCTTATCAGTGTCCTGAGTTTGCCAAATGGAAAACATGGGTGATCCAAAATTGCCCACCACCCCTGACTGAACAGCATTGCCCACCACCACCAATATTGGTAGTTTTTGTATGAAGACAATTAAGTAAAATTCAAAAGGAAAATGATCAATGGACTCCAAGGGAGTAAAACTCCTGTAGTCCTGCCATGTCTTGATTTTATTGGATGATGATAAAAAGAAAGTAATCGAAACTATGGCCCGCATTTTTTTTTTTGAATGAATCAAGGAATGACATGTAAGCAGGGAGTAGAAAATGGAGTAGAATGTGGGAGTATGTGTAGTATTACTCAATTCAAAATTTTCTGTTTTTTAAAATCTAAATTAGAAAATAATTGCAATTTTACAACTAAAGAACACAGTATAATGGAAAATGAAAGCTTGAAAACTTGAAGTTGAAGAAAAAGCATTCTCTACAACAAAATAGATCCTTAGAATTTACGTGGTTTAAATATTAGTTGCTGGAAGCCACTTAGTGAATCTTTAAAGATGTTTTTATGTTAACAGTAAAGCATTCCTTTCTGATTATAGTAGGTTACATTCTTTCATGCTATAAGATAATAATTTTTCATGAAGATGAAATAGTCATTATTTTTCTTTTTTAATCATTCATGCTGTACTGATTTGCATAGTGTCTTTTTTAAAAAAAAATCAATCATGCCTACAATTCAGACCAATGCCATAACAAATGCTGCCTCCCCAGAGACTCTAGTCATTGGTTCATTTGAGTGTCTGCCGGATGTTCCTGGAAGTAGTGACCGAAAAACAGGTGAGTGGTTCACATGGCTCTCTATTACTGTCCACATGCTAACAGATAGAAATACACATTACCATCAACTTGTGGATTTAGTAACTTATTCTGAAAATTAGAAGTATTTACTCTGCAAGTTCAATATCTATCCATCATATTAATATATTATTATGTTCTACTTTGTTAGAGGTATCATTTTGATGACTGCTTTTAATAGCATACTGAGATTATGGTCACTTTTTTTTTTTTTTTTTTTTTTTTCCTCCTATTATAAAGGACAAAATCGTGTCATGGGCAAGAGAATTTTGGTTTCAGTTTTGTCTCCTAAACTACAATACAAATGAAGATTTTCAGTTTGAACTTTGTTTCTTGAGTAAACTGAAATAGTCATTACTAGCTGGCAAGAACTTTGGATATCCATGTTGTTATTGTCCTCCTTGTGCACTTGAATTTGTTATCTTCTCAAGTGTTCTGCCTTTCAATGAAGCGTGGGTATCATTTTAATCTGTTACACTTGGATTTATCACTTACATTTAATATTGTTTCCAGGAAAAATCTGTTCATTTCCAAAGAAGAGTGAATTGAAAATAGGCGAGTCTTCTGCATTCTTTACATATGTCAAGTCAAGCATGCCCAAGAGCAATGACCAAGTTACTGTCCGTGAAAACGTGACTTATCATTCAAGGATCAATGAGGGTGGGAATGTGGATATTGAATCCAAAGAACGGGCCAATGGTGATGCAATCGAAAATCATTCACAAGGAGATGGCTATCCGAGCAGTAACAGTATTCCCGATTCCCTCTCTATGGAAAGGTCTTGTACTCCACCTTTATCGATGGAATTCCCGCAACAAAGGATGGAGGAGTTCTCCAAGGTGCATATGCATCCAACAAACGAGTCTCACCACGACATTTCAGGTTATCATGCCCATGCCCATGCCCATGCTGCTTATCCACCGTATTACATTCCAAGGATAATGAATCAAGTTATGATGCCCTCTTCACAAATGTATCAAAAGAACTTGCCCGATCTTCACAACCATGCTAATTCCGCTATGCTTCCAACATACAGTCATGTACCGCATTGTCCTCCCCATATGCCTGGAATGGGATCATTTCCCTATTATCCAATGAACATGTGCTTGCAACCAGGCCAAATGCCACCACAACATCCATGGCCATCATACGGGAGCTCCTCGTCTGCTGATGGTAAGATGGGGAAAATAGACCATAGAGAGGCAGCCTTGATGAAATTCAGGCAGAAGAGAAAGGCGCGATGTTTTGACAAGAAGATTCGGTATGTCAACCGCAAACGCCTGGCAGATCGGAGACCTAGGGTTAGGGGACAGTTTGTTAGGAAGCCCAACGGAGTTTTAGTGGATCTTAATGGACACCCTGCCTCTGCAGATGATGATGAAGAGGATGACGAGGACGAGGACGACGAAGACCAAACAACTACTTTGGATTCCTCACCTGAAGATGACACTTCAATTTCTCTACTCTGA

>g8789

ATGAAGAGAAAGGATTTGGAGATGGAGATCCATGCGAAGGAAGAAATGCACGTAACCGGGGACGACGACAAGGAGTTGACGGTTAATAGTGCGAGTGAATTCTGGGAGATGTTTCTTCATGTTACGTCCATTAGGGTGCTGGTGGTGGAGAACGATGACTCCACGAGGCGTGTTATCAGTGCGCTGCTTATGAATTGCAATTATGAAGGTTAGCTCGATTCGATTTAATTTCCTCCAGTGCTTTGATGCAGTGTTTAGGAATCTAGATTGAATATTCATATGCTCGATCGTCAATGGATATGATCTAGTATTGCATCGTCCTTTATCTGACTCGGATCAATAATCCATATTGAACTTTACTGGTTCAGTGCAGTGATCGGAGCTTCAAACGGATTGGAAGCATGGAAGATTTTAGAAGATGAAACCAACCAGATTGATCTTGTGCTGAGTGAGGTAGTAATTCCTTATTTGTCAGGCTTAGATCTTCTCTGCAAAATAAGGAGTCACAAACCGCGCAGTAATATTCCCGTGATTAGTAAGTAACTCCTTTCAATTCTCATCTGTTAAAACTTCTGATAGATAGATAGTGTACAAATTGAATTACTCTCTTTCTGAGATTCCTGAGTTGTTAATCTGTGCAGTGATGTCCTCCCACGATTCGATGAGCTTGGTGTTTAAGTGTTTGTCAAATGGCGCAGTAGACTTCCTGGTAAAACCTGTTAGGAAGAATGAGCTTAAGAACTTGTGGCAGCACGTCTGGCGAAGTAGCCACAATGTTAGTCGAAATCAATGAAGTTGTACCTTCTTTTTTAGACCACACAGAAATTATAAATTTCTGCTTTTCTCACTGTCTTCATTTCTTTGTTCCTCAGTCTAATGGCAGTGCAAGTGGGAGTGGGAAAGAAACCAAAATCTCTGACAACATTGGAGAGGATAGCAGAAGCAGCAGCGACTTGAAAACCGGTGGTGACAATGAATGA

>g25313

ATGGTTGGAAATCACCACGTACTCTCAAAGACTTGTTCTTTCATCCCGCCCGCGAGCCCAGAATTATCTATCTATCTGAATCTGAGGTCGTCTGAACATGAAGCTGTTCCTTACTACTTCTTCTTCTTCTTCTTCATTCTGGGTTTCCTTGAGAAATTCCAAAAAAATCAATTCAAACCTCATCAATTCAGAACCAAGAAGCTGTCCTGTGGGTGTGGAGTTTGGAGTTCAATAATAATATCCCCTCCACCGCCTGACTTCGACTTCAGAACCCAAATTCTGGGCGATTCTCGGGCCACCATAGCCGAAACTCAACCTGAGTTACTAGACTTGGCTGATAATGGAAGCTTGGTTGTGATCAGCAAGAGGCAGTATGGGGCGGTTCCACCTTGGCGCACTGAGTTTGTGGAGCCTGACGCCATTTGGCTGATTGGAACAACACACACTTCTCAGGATTCCGCGCTTGATGTTGAGAGGGTTATTCGTGCTGTCAGGCCTGACAATGTTGTTGTGGAGCTCTGCAGAAGCAGGCAAGCTAAATATATATATATCCAGTGTTTAATTTTCATTTAATTGCCGGTCAATTTGATCTTTTTCATTTTGATGTATGTCTGTTGAATTGAATGTTTTTAAGAGGGAAATTAACTTGTTGAATGATATATATGTATATATATGGCTTAACTTCACTTGTTGCAGAGTTGATTTTCAGAGCCGGGATCATGTACACTGGTGATGTTAATCAGCCCTTGAAATCAAACATGTTTTCTATGAGTGGGAATGGGTTCTTTGGCGCTGTTGGTCGTAGCATAAACTTGGGTATGTAAATTAAAGAATGTATATATGTACATACGCTGTGATTTTAATTTGCTTTTATATATGCTACGGAGTATTATTATTAGTATTAGTATTAGTATTAAAGCAAAAGTGTACTGTGTGGCGTGGCTCATATATATATGCAGGGGGGCAGACTGCTCTAGCATTGCGTCTCTTGTTGGCATTTTTTTCCTCAAAACTCTCATCAAATGTTAATCGTCCCTTTGGTGATGAGGTAATTAATATATATACTCAATATACAAATTAAACCTATTCCAAACTGTATGTATATATAGTCGTAGGTGCTTAATCTAATTAATTAATTTGTGGATCTGAACCATTATTATCTAATGGCAAGTTTGATTGTATGTATGTAGTTTCGTGCTGCTCGAAAGGTTTCTGAGGAAATTGGTGCTCAAATAGTATTGGGAGATCGACCCATAGAGATTACAGTATGTTCATTCCAGGCCAGCAGTGCAGTAGTGCCACTGAGCTCCCTATTTTTTAATTAATTAATATAAGTATACCATTAATATAATAATTGTTAGGTTCTCAAAAAAAAAAATATAATAATTGTTAGGTTTGTTGTGTTGGATGGATCAGCTTGAACGTGCATGGAATTCATTGAAGTTGAAGGAGAAATTTGGATTGGTTTCCTCTGTTGTCCGTGGAATAACTTCTTCATCCTCTGATCTCGACATCAAAACTTTGCAGGTTAATTACTATATATGTACTAATTAATATAATAATTCTCTTTCTTGCTTAATTATATTAAGCAATGCATGGCAGCAGGAATGGAGCTCCAAGAACAACAACACCTTCCACCTTTATGAGCAATTAAGCTTCTCATATCCCTCACTCCTGCAGCCCCTCATACACGAGCGTGATACAGTAAGTATATTATATTATATTATAATATATTTATAACAAATCACTGCACGTGCGTGACTTTTTATGGACCGGGTTATCTCTGAAATCTGTAACAAGTAACTGGACTTTAATACCAATTATTCGGATCAAACACTTACTATGTGGATTTGACCGCCTTACTGGCCTCCGCCCAACCTGCAATATAGTATAATTGTTACATATACATGCATATATGCAGTATCTTGCTTGGTCCTTGAAGAGGAGCAAAGCAGTGAACAGGAGTAAGAAAGTGGTGGGAGTGATAGGCAATGGACACATGAACGGGGTCATATATTCACTCATGTCTGACCAAGGCAACCTGCGCTTTCGCGACCTCGTTGGCGTCCGCACTGCACCTCAGAACCAATACGCTTGGGTTAATACCATTTTCAACAACTTGTTGAGAGACACTGTAATTGGTATCATATTATGGTTCTTCTTTCAGCACTTCAACCTAAAATTATAGTTTTTTTTCCCCCTCAACAAATCTGATGATTGGATTGTGATTATATATAGAAGAATTATAATTGCGTTGGAAATTTATTATTATTTTAAATTGATGGATTTAATTAACGGTCCGAATTAATAAACCCGATAATTAAGAACAACGATAAATGGATATTAGGAGTAAAGTAATAGCAAAGTTAATAATGAGATAGATAGTGGTAAGTTACCGAGACAAATTGCATTGCTAAGTTGTGTAGATGATTACAAGAAATATGTAATGCTATTACAATACTTGTGTAGACAATTGTAAATGAAGATATCTTAATGAGACAATTACAAGGGTTTTATACTAGTCAACTCTCCTAACCGCCACCATAAATAGACAGGAATGGTTGCAACAAACCTTGACCACTAGACCCGGGTTAATTGGCTAATGCTTGGGCCGAGTGCCTTGGCCTAGGCCGTGGCCCAAGCAACTAGGATGGACTTGCTACAGTAGTCCAATTCCTAATATATCCATCATAAATAAAAATAACATAATAAATATGTAGATTAAATTTAATTTTTTGATTATTTGTGGGAAAACCAAGGATATGATGACATCACCCCAAATCCATGGTCGATCTGGTGCAGCACCTGAGCGGATCGCAAGATATCGGATAATCTCAATCTTCCTTAATATCCATTATCATATCTTTTCTTATGGAAAAACAATTAATGTTAGATATGATACTATTTTTGGTCGTTTACAAAAGATCGCTTGTCAAAGATTAGATTTTCTTATTTTCTACTGATTTTTATTTTAATTCATATGTTTTTAGATAGTTAAGTAACATGTTTCAGTGATTTAGTTGGCATAATAAATCAAACTACTCGTATTATAAAGTGAGATTGTAAATTTTATACAATTCTTAGTCATACAACTAATAACTGTACTTTCATTGTTTTTAGTTGTCTAAGGTAGTGCAAGCCCCGTGCGTGCACGTCCAAATTAGATTCTTATTATATTTAAATATAATTTCTCCTGATCAATTACATAATTACTGCAGCATATAACTAAACCAAAAAGTGAAAAATAAAATAAAATAAAATATAGCGAATCCAGGTTTGTAAACAAAATATTTGATCGAATCCACCATGCCCCACACGGATGCAACCCATTGACTCAACGCCATGCAACAAAACGTGTATAAATACGATGCGTATGTTGAGGTGATATTCGTAAAAACACAGTGCATCGTTGAATAATTTGTCATTTGCTTCAAAAATCCCACCCCAGGCCAGGCCAGGCCAGATCAATGATGGACTCCCCTGCTGCTATTGCCATGAAAATTTTGGTTGTGGACGACGATTCCACTTGCCTTGCCGTTGTTGCTGCTCTTCTCAGGAAATGGAATTTCCAAGGTTACGTTAGTTGATTTATTACTCTTCCTTTAACTTCACTCATCGATCACTTGTTTCTTAAGATCTATATATGTATATGCATGCATGGATCCATCCTTTTTTGTTAATATAATTATACACATATATGCGACTTGCAACTTGCATCCATCTTTCTTCTTTCTTCAGTGGATCGGATATATACATTTTGTGTATGCCTCGTAAAACATCTTTAATTTCGTGGGAGATCAACACTTACAAGTTACAAATTAAATCAAATAGTAATTCATTTGAATTATTTTTCAATAAAAAGTATGTTAATTATTAGATCCACAAGTTTTCGAACAAAAAAAAAAATAGATTGAGGGTGTGTTTGGTTCACACATGGGAATCGTAATCAGGATGGTTATCAAATAATTGGTAAAAGTAATGAGTTTTGGTGAAAGTATTTTGCATGTTTGGTAGTAGGGTGGAATGAGAATGATTATTAATAGTTGAGGAAGAAATGAGCGAGGAAGGGAAATGAAACCCTTATTTTATTAGGGTATGAGTTTATCAATTAATGGGGTATTCCAAACCCATAGTAATATTCTAAAAACCTATCAACCAAACAATAATAATCACTTTGATACCCATACCTTATGCCTAAACCCACAAACCAAACACACCCTGACAATTTTTCAATTTTTCAACGGGAATGGTTGATCCTCACCCCAATGTGAACGGGGATTTTCGATTTTGGCCTAACGGAGACGGGAATACCCCTCCCACATACCTAATTAATTTTTATTTTTTATTTTTTATTAAAAATGTACTAATTTGACCATTTGATTATGGGATTGACCCGAGACCAATTACTTTTCAATTAATTCCTCAACGAGGTTAACCCAATGGGGTAGCTCAATTAGCAAATGAGTTCTCTTTGTGGGGAATAATTTCGGGAGAACCCGGATTCGATTCCCACAAGTGACGATTCCCCGAAGAGTAAAACGAATACATTGCTCTATAATAAAACATGTATGGCTGGATGGACAGTTAAATATCTAAAATGTATGGTAGTTAGATCTTTATCCCTTCTTAAATAGATTTGCTTTCTAAAACATGTTTGGCAGGGGACACCACTCAAATTTGATCTATTTATAACTTATTTGAACTATGTATATATCATTAAAACGTATAAATGCTAATAAAATCCTTATTCCTACTTTGTGACTAAATAAATTTGTTGTATGTATTTTTTGAGTACTACTGACTCTCTGCTACTTTCTTACATAGGAGTGTAAACCACTAGACTACAAAGTCTTTGACAAATTTGTTGTATGTATAAAACATGTTTAGCGTGCAAATCTAGTAGGGGAGTAATTAAAAAGCATGTTAGTTAAATCTTTATTTCTTTTTAGATAGATTTTGTTTATTACAAAACATGTCGGCAAGAACCAACATTTGCAGAAATCTATAAATAACTCTTCATTTGAAAATTTTAAACCTTTTAAATTCTTTTGAAAGAATTTATTTACTTGTTATATTTAATGTAGTTAATTAGTGTGACGCTAGCTTTCATTCATTCCTATGCTATATATATTTTTGGTGCATTAAATAAATATATAGAATGGAAGTAGAAAATTTGTTGTTAGTTTTTTTTTTTTTTTTTTTTTTTTTTTTGTTTTATTGTTATTAGTTAGGAAGCGAGTAATTAATTTATATTTTAAATTACTAAAGTTGATGGGGATTTAATTAATGGGGTGTAGTGTTGGGTGCAGTTGTGACAGTGAAAGACCCCAGGGAAGCACTGATTATTGTTGGTGGGGTGGAAGGTGGGTTTGATGTTGTTATTGCAGATGTGCACATGCCTGAGATGAATGGATTTCAATTGCAGCAGCAAATCACCACTGACTTTCAACTGCCTGTTATTTGTGAGTTTTAAGATTTGGACTTTATATAGGATGATGATGAAATATGTAAAAAATGATGAATGTAAATGCGTGCGTGCAGTGATGTCTGTTGACGACAGAGACAACTTGGTGGGAAGGGGAGTGGACTTTGGTGCTACTTTATTCATAAGTAAGCCGGTGTCGGTTAATGATGTCAGAAGATATCTATGCCAAATTAAGAAGGACGATGACCAAAATAGCAATATATATATTATTCCTCCTCCTCATCATCACGGTCAGGCTACTGCTGCTGCTGCACCCACCAGGCGTAGCACAGATAATAATAATAATTCTACTGAAATAACAAGAGAAGGAGAAGTAACATTCAAGACTAATAAGCTCACCGGAAAGAAAAAGCTTGTCTGGACACCCACACTTCACTACAAGTTCTTGGATGCTATCACAATAATTGGACTTAACAGTAAGTTCAGTTGGATCTCTCTCTCTCTCTCTGTGTATATATATATTGGTTAAAATGTAATTAAAGTTGTCTGATTTAATTTGTTTGTCGGCCTGGATCTTTAATTATTGGTTGTGCCTGCTGCGTGCGTGGTGCGTGTATGTGGGCGCAGATGCTGCTCCAAAGAAGATTCTAGATGTCATGGATGTGCCCGGAATAACAAGAGACCACGTTGCCAGTCATTTACAGGTAGCTAGCTAGGTTCCCTCAGCTCCATCGCTCGGAGTGTTTAATTTGTTTTTCTAGTTCTGTATATATATGCAGAAATACCGGATGTTGTTGAGGCGGGTGTCGGAGCTAACGATGCCTGCAAGCCAACGTAATTATAATGTTGATTGGGGGTTTGGCAGCGGGGTGAACTTGAGCCCAAGAATTGCTGCACCCAGAAGGTACGGCGGCGGCATGTTTTCCCTAATTAATAATCTGCAGGCATCATTAGGCGGCGGCGCCGGGCGGTGGAATATAATATCACGGCGCCTATGCCCCCAGCTGGGACCTTCTCATCATCATCGTCCTTTTTATTCTCTGATCCTCCTCCTCCTCCTCGCCTAGCCGTCGCTTCACCTCCCTGCAATCAACCCAGGTGGCGGCCTGGATATGGTACCGGTGGTCGTCAATCAAGCCTGCTGCTTTCGCCCCATCATCATCACCAGCTTTTTAATTTTGAACTACAGTCGTCGTCCCCGTTGATACCTGCAGCTAGCACCAATATGGTGGACCCAATTTATCAGCGAAGCTTCACATCGTTGCCCCCACTGGTAATCCCTGGAAATAATGACGATGCTATAGCTGCAGCCTCACCGCCGGTGCCGACACTGCAATTGCACGGTGGAGGCGGCGGCAGTAGTATGCTACAACCACCTCCTCAAACATACAACAATAATAATGCTACTGGAGGAGATGAAACCAGCTCCCTGAAGCTACCTGCAAGGCTGGGGCTCACTGCTCCTCTCTCCACCGAATTCTCCATGTTTGACATGATAGATGATGATCACCAACTGGATGGCGGCGGCTTGCAGGCTACTCATGATGAAGATCTTATAATACATAATAATGATTGTGAAAATCAAAATTCCAACTTCAGCAATAGTAGTAGTAGTAATGCTGTGAAGAATGTGACGACGACGACACAGTACTACTATACGATCAGGATCTTCCAAGCCTTGATCAGCTGA

>g20841

ATGGGAGAATTCGCAGGTTTTCGTCACAGTTTCCCTCAGGTTACCGATGGGACCAATTCCTCAATCGGTTCACTTGTGTTCCCGCCACGGCGTATGCACGGTGTTCATGTGTTGCTCGTTGAGCGTCGCGCGGATTTCGTCGCAAATGGAACTTCCATGGTAGCAGAGATTCTTAAGCAGTTCTCGTATGAAGGTATAATATTATCAACTTTAATTCGTCTTCTTTTTATTTATCTATGCACACCGTCATACAGTGACTGGATTTTCTGATTATCTAAAAAATATAAATTTATTTTTACACTTTTAATAAAATTTATTTGTATGAAGTGTCGTGCATGCAGCATGAATCTAATTTGTAGAACAAAAACAGTAACCTTTTTAAGTGAAGTGAGAAATAGTTGAAAGCGAAAGACTAAAATATGAAATTTTCTTTTAATTTTTTTAGTAAAAAAAAAAGAGCAGGTACCAAATCTTGACATCCTCCTGATCGAGTAAAGTACTAAATATTGTTTTCTTAATCTTTGCGTTTTTTTCACTCAAAATATCTTTACAAATATTCTTAGGGTTTACATAAATTATTCAAATCAAATTTTTTTTTGCACACATCTCATGATGCGTAACTGGGTACATATTTTATTTTATTTTTAAAAAATCTAAATAGCATTTTTGCAAACTAAACAAGTTTAGGGGAGGATTGATCTTTATTTTATTTTATTTATTTATTTATTTCCCTTATTAATAGGAAAACGTATACCAACTAACAACCATCGTTTACTTGCTTTAATTTATAGTAACAAGTAAATATTAATAATTGTAAGGGATATAAGCATACTGCCACGTGTGAACTATATGGCTGCGAATAATTAGAAGTGGTTTGGAGCTATTAAATAAAAGCCCGGTTTTATGGGAACATATTTAGGTAATAGTTACGAGATTTTTTCTGTTAAAAAAAAGTAATAAATAAACTTATAGGATGTCAAATCAGCATCAAAATGGAAAAAATAAACTATTTAATTCATTATTTACTTTTAGTACGTTACGATTAGTTTTTATATACTGTAGTTGAATTATGCAATATTTTCTCAGCTAAAATAATTTAATTTATGCACACTCTAATAATCTTAATTTGGAATTAATTATTAAATTGAATTGACTTTGGCAGTGACTGTGGTGGAAAGTGCAAGCGCTGCACTGTTAAGCCTCTACCATGGCAAGGAGAAATTTGATGTGTTAATCGCCAACTTTTACCTACCAGACAAGGAAGTTAACGTTAAACTCCTCGAAGAAGCCATTAAAAGGAAACTGCTTGTCGTCCGTAAGTATTTTTTTTTTTACTATTGACTCTGTTAGAACGCAGTATATGTTGTTATATTCATTTTAGAAAGATTAATGTAATCTGGTAAACATTATTGTTTAATCAATAATACTGCAGAGATTAGTGACGAAAAAGATGAAAATGGAGATGAGGTGGCAAGGAGAGCTATAGAACAAGGAGTGTTCCTTTACCTGGAAAAACCTTTTCCGGTAGACATGTTGAAGTATCTGTGGCAACATGTGTATAGGGAAAGAAGACTGATGAATCACAGTACTCATCAGGCTTTGGATATAAGCATGGTGGCAGAGACGTTAATGAATGGACAAAACAACATTGTTTTCACCGACAATCAGACTGCGACAGAATTTGCAACGGATTCAAACAACGTTAATCTTGTGCCAACCAGGAGAAGAGGGGCCAAGTTTAAGTGGACTGAAGAGCTCCATGCCAAATTCATGCACGCCGTCAATCAGCTTGGAGCAGGAAGTACATTCCTTTCTCACTTTTCCACGAGAAAATCCTTCCACGTACCCTTTCGAGTGTGAGTAAACCTTACTTTATGACTTTAGTCCAGCATAAATTATCCATGGTAGATCGCCTCAAACAAAGAGAGTAAGGATTCAAACTCGTGGCCTTAACTGGGGTTAACCCAATTTACCTTTATTATTTTGGGTCTGGCCTAATATTTTTTTTACAGGTCACATGCAACATGTAGAGTTGTAGACATGTTCTAACTTCATTATATTATTATTAGGAAAAAAGTCAAATAAGTCACTAAATTAATTGTTTATGTTCAATTGAGTTACTGAATTTTAAAAAGTGTGCAGTTAAATCAACATTTTTTGTTTGATGGTTTAATTACACACTTTTTTTTAAGTGACTCAATTGCACAAAAATGATAAGTTTATGATCTATTTGACACGTTTTGCTTATGTTTGTAACTTCTGTTTATTTTTTGCGACGAATAGATTGCTATCCGAAGGAAATATCGGAGATGATGAACGTGCCAGGTCTGACAAGAGAAAAAATTGCTAGTCATCTGCAGAGGTGTCGTGACAACAAATGGAGGCCAGTGGAGGAGCATGGAAATCGTCGCAGGTCACGAACAATGCAATCCACCTCTCAACCTAGACGCCCTCGTCACAAAAAGTTCGGGTTGATGCCCACAGTCGAAGAATTGGAAGCCAATAATAATGGAATCATGCCTCCGCAAGAACAAATTGTTGCTGCTGCTCCTGCTGCGAATAATGAAATTTCGTCGCAAAATGGTGGTAATAATAATAATTATGAGTACAGAAGCTTAATGACAGTGTCCACTAATTCCGTCACTTATATTGATACAGGAATACTTCAAGCTGTTCAAAGTTCAGCAGGCATTATTCAAGATCGTCGCCTGTTGGGAGAAGTACAAACTCACCCAATCGTCGCTGATTCCGTCACTAATACTGATGTAGGCGTATTTCAAACTGGTCAAAGTTCAGCAGGTATTATTCATGATCGTCCCCTATTGGAATAAGTAGAAACCCACCCAATCGTCGCTGATTTCGTCACTAATACTGATGTAGGAGTACTTGAAATTGGTCAAAGTTCAGCAGGCACTAGTCATGATCCATATCGTCCCCAGTTGGAAGAACTACAAACCCACCCAGTCGTCACTGATTCTCTGAACATTGACGAAACAGCTTTTGTGAATTCGTCGCCAATCCCCTGGCAGTTCGACGATGGACTGATGTTTGATGACCTGCTGAATATCCCGGAGGGAATGTTACCTCGGATATTCTTCGGCGGACAACTAAATTTTCCTTTTTAA

>g20553

ATGATGAGGTTAAGCATGGAGAGTGAGCGAAGCGTAAAAGTTATTGTGGTTGAGGATGACTCTGACTCTGATTCCACGTGTGTACAAATGCTTAGGAAATTGAAGTTTCAAGGTAGTCATTATAGTAGTATTATATTATATTATATTATATTATATTATATTATATTATTTTTTTTGGTAAATTTACGTGTCTTTCTCTCCGTCCGCTTAACGGTCCGGGTCCACCCATCGCTCAGCCCGAGGCACGGAGTCGCCTGGGGGAATCGCCACTTGTGAGAATTGAACCCGGTTCTCCAAATTCTCCCACAAAGAGAGCTCACTGCCACTGAGCACCCCACTTGGGTTTATATTTTATTATATGATTATGAATTGACGTATGTAGCATTTGTTTGTACTAATTTTGTTCTATTTGCTGCACAGTTGAGGTGGTGAAGCATCCTAAAGGTGCTTTTGACACTCTAGTACGTACTAGGGGAGAATTTGATGTTGTTATTTCAGATGTGAACATGCCTGACATCAACAGATTTCAACGAATGATTGCTCAACAATTTGGGCTTCCTGTACTTTGTGAGATTTTTCTTCTTTTATGTTTCATTATATTTTTATTACTAAATAATCGGAAGTGATCCAGTATTACTTAATTCTCGGAAAATTCTATTTTATTCAAGCTGGCTGAAAGTAATATATTTGAGAGTTGAAAAATGCTTCAAACACTACTCCAAGCACCTTGGAGAAGAAGCAAATAATTTTAAAAATTAAACAATTTGATTTACATGCTACACACCTTGAATTATTAATGATGGAATATAATCCTATTGGTCCCTGGCAAGCAAAAAAAGTCTCAAGAGAATTGAATAGACTTTTTTAACAATTTTAAAACCTTTATGAGTTAGTTCTAAGTGAGTTTGAATTTATAAATCAAAGTTTTCTTGCAATGAAAATATCTTTGACCTTGTCGATGAAAAGATGTAGCCATATTTTATGATTTGAAGTGTTAGTCTGTGTAAGTATGAGGTTAGTTTCACATTAGAGTAATGAAAATGAATGTAAAAAATGATAAAGAATTTTTCTATTCCACCCTAGCTTCCCCTTAACTCCAATGATGTTTCCGCTAAGTATTTTTTGTACATACACAAATACAAAGTGACACGAAGCCTTTAGCTTCTACTAATTGACAATCCTTGAGACAAAGGTCAATCACAAAGTCATCTTGTCTTTCATTGCATGATTAGAGCTAGCTAAGGGTCACATACCAAGTCGTGATGCTAGGTTAGTTGATCTTCGAGGGCTGGACTTTCAAGAATCAAACTGATTCTTTTCTAATCTTCAGCTTAGAAGCTTAGCTTGATTCAACAATATAGCACTTTCATAGTAGCATGAACACTTATCACTATTTTTCAATTATTCACAAAATTTCTTCTCTCTCCTTCTAAGCTTAGGTCGAGGAGAGGTAAACACCAACATAAATTTTGAATTTGTCCATTCTCACTCAGTAAGTGGTAGCCATTCAAAAGCTATCATAATCGCCTTAAATCAGTTGTCATATCTTTAAACTCTTGCAGCTTGATATCTATAGGGTAAATAATGTTTTCCTATTAGTGTATAAGCACGCCAAAGTAAAAAAGATTTGCTTCTACTTATCACCAATTTGTCTTACATGTAAACTTCTAGGTGTACAAATTGCATTTTCCAACGGTTCTTTTTGTCATGTCCGGTGTGAACTTTTTCCGACGATGACCTTCTTTGAAACATTAAATGCCCTTTCATCTTCCATGCATTAAGATCACTTCTGAACTACTTTGATCATCTTCTGATTCCCTTCGCTTCATTTTGAACCCTTTCCTTCTAGTCACCTTCTTCCAAAACCCTTTCTTTCGAAACTGCTTCCTTTCGGTTAGGTGGTTACAAAATTTGTCTAGATTTACAAGAAAAAGAATGAAGGGTCTAATGTGCCCTATACAATATCTTTTACATTATCGATCATTTTATATAAATGTTAAATGCATATATGCAGTGTTATGTGATGAGAAGCAAGAGGCTACTTTTGGGGTGAAAATGCAGAATGTCCTAAGGAAACCGGTGTCGGCTTATGAACTTAAAGATCTATGGCAGATTATTACAGCTCATCAGAAGTGCAAACTCTCAATTGGTATGGAGCTGGGGGAGACGGGTCCGATTATTATTTGTGATAAAAATCTTAAGAGCAAGTTCAATTGGACCACTGAACGCCATTTCAAATTCATGGATGCCATCCAGATTCTAGGCGGCATTAAGAGTTAA

>g59988

ATGGGTAAGGGTAAGGGTAAGAGCATTATGGTGAGTGGTGGAGATGGTGGCAATGCCACTGATCTTCCTGATTGCAGCAGAGTTAGGGTTCTGCTTTGTGACACTAATGCTGACAGTTGTCGCCATGTTTTCCAGCTCCTAACCCAGTGTTCTTACCAGGGTAAGATGGTAATTTTGTGTTTTTCGTTTTACGATGAAGTTATTGGCTAAACTCGACTCATGTGTGCAGTTGCGCTGGTCACCTCGAGAGCGCAATTGTTTGATACGTTGAGATCTGAGGGTCCTTGTATGGACATCATTCTTGCGGAAATCGCGATTCTTATTGCGAATGAATCGAGTATTATGAGGTATATCAAGAGGGACGTTAGACTGAAACATGTTCCCGTGATTAGTAAGTACTTGTTTGATCTAGAATTTATTATCAGAAAAGATTTTTTCTCAATCATTGAAGCCAATCTGTGTTTTGGTTGTTATTTTTGGCAAGTGATGGTGACAATTGAAGAGGTATCCCTTATCCGGAAGGGATTGGGATTTGGAGCAGCAGACTATCTTGTTAAACCACTGAGCATTCATGAAATCAAGGATTTGGGGTTTCACATCAAGAAAAACTAG

>g34928

ATGGGGGTGAAGAGCATGATAGTTGGCGTAACTTCGCACGGTCCATATGCCGTCAGGGATGAGTTTATCGCGGCGGAACTGGACGAGTGCCTGATGAAGCCGTTGGGTCCAGAAGTGGTTTTGCATCTGATTAACCAGCTAGTTGCCTAAAAATGAGTTATTATGAAGAGTATTTTCAATTCCTATTTTACTATTCCTTAGTTACATTCTAAGCTCCAAAATGGGAAAATACTAGTGTATTGTTCTGCTTGTGTAACTGCAATAATAATAATAATAATAATAATAATAATAATAATAATAATAATAATAAATAATGAGATGATGAGGTTGAAAAATATATTATATTATTTTTTAATTTTATTTGAAGTTTTTCTAAGTCTGCACCCCGATGGGAAACATCGCAACGACCACGCACCAAGCCTATGTGCTGGGCATCTCAACCACCTCTTTCCCTGGCCTCATCACGCAAAAAGCATATAACAATTAATGTATTAATTCTAATTATCAAAAGATGTATTTATTTGGATTATTAAAAATTGATTAACCTCATCAAATGGTGATGCATTTGTATATATGACAATTAATCAACTAACACCCTACATGGTCCGGATACAATTTGTCTAAATTGAAATATTAATAAAATTATTGAATAGCTAAAATGCAAAAATCATTTTTAATTTTTATATGTACAAGGGATAAAACAACCTAGTTTGTTTACCAGTCAAAACCAAAATGGTCAATAGATCGTCGCTCAAGTTGTGTTGGATAAATCTCGCATTATGACCATAGCCCGTCATATGCTTAAACTAAAAAGGTCAATAGGTTGCTACCATTTTGTTGGGTAAATCTCACATTGTAACTATAGCCAGGAAAAGATAAGGTAAATGAGACTAAGTTGTCTATACCGACTCAAATCAATAAGACGACTCAGATTGGGAAGGGAACTTGTAACCTAAAAGTTACTCTTAAAACCAACAATCTCTTGATTTGGCTGATTGGCTCGTTGTCCCCCTCTCTTTCCTTATTGGTCCACAAGATGCTGCTGAAGAAATACGGACTGGAAGCTCAGATGGCCAAGAATGGCGAGGAGGTGGTGGTGCTCCACCGCTTCGACCTCTTGCTCATGAACCAGGAAATGCAAAGGATGGCGTCAATGTGAACCCATTCTTCCCTTTCCCTCTTACTCTTATATATCTATTCTAAAAACATTAAGGTGACAATTTGCACAGTCGATGCACGGTTCTGTATCTGTCTTAATTATATGTATATTTGAGAAACGGTAAGTTGACTATTTAATTTATCATGAATACTATTTTTATATATGTATTCAAGAAACGCCAAGGTGACAATTTGCATCCGATACTATTCTGTATATGATATAAATTGGCACGATATTGATCAAACCCTGAAAAGGAAAGCATGTTATGTTGTTTTGTTTTGGCATCGTTGAATTAGGCAACTCGGGATCGGAGCTGTGGGACATGGGGGTGAAGAGCATGATAGTTGGAGTAACTTTGCACGGGCCCGGTGCCATCAAGGATGAGTTTATTGCGGCCGGACTGGATGAGTGCCTGATGAAGCCGTTGGGTCCAGAAGTGATTTTGCTCCTGATTAACCAGCTAGTATCCTAA

>g61529

ATGAATTTCAAGGAAAAACAATGGTGTGAGGAAAGTTTTCACAAATTTACAATTGATTTCATAATTTTTGTGTTTGTAGATTAAGTAGCTAATATAGTTTGAAATACATATATATTGGTGCGTGCGTGCAGTTACCGTGGTGGATTCTGCTAATGCTGCAATTTCAGTCCTTAACCGGAAAGAAGAGAAATTCGATGCCGTGATTGCGAACATTCATTCACCTGATAGGCAAGCGTATAAGCTTCTTCGCGATGCTGTCAGCATGGATTTGCTAGTGATCTGTAAGTAACTAATTTCTTCGAATTTAATTTTTTAATTATTGTTATTTCTTTGAATTGAATAATGTAATGTATATCTGATTATGTAATGTAACAACAACACCACCGCCGGTGACGACTCTTTCGTCACTCCCCAGGCGCCCCGCATCCACCGCAGGCTTCAGTCCGATGAATTCTTCAGCTTCAACGACGTGGACTATGAATATCTGATTCAAGGTTTCTCCGACAACAATGCTCGCCAAGCAGGCGTTGCGCTGCAGGCCCCTACCCACAACAACACATCCTCGTCTGAGTTCAACGACAAAGCAGGTTTCAACCCCGTGCAGGTCCTGAGCGAAACACTTATAATTCTTTACAATACAATATCTGTTTATATATATTTCAATTCAAATTTTAAACTAAGATATAGTGATTAATAGATGCTATATTTGTGGCAGAACCAGGAAGCAAAGGCACAAACTTCGGAGATGGACATTGCGAAGATCAGTCCTTAGCTTGAAGAGGACACATCTGTGGAAGCATCGTGACCAGAAGATGACAATAATAAGGACATGCATGCACGCATGGATAATTTATTTTCTTTGTTTCTATCAGTTTCTTTGTTGTGTAATTTTAATAATTTCTGCTAGACATTACCCAGCAAAAGACAGTTATGTTTTATAATTTGCTGTGGTTTCTGCTTCATTAACATTTCCTTGATTTGATATTATGCAATTTGGTATTAAAAAAAATGCAGTTCTTGCATTTGTTTTATCATTTCTTGTTGTTTGATCTTTTAGTTAAGTTATTTAAGGTTTTTTTTTTTTTTGAAAATAATAAATTGTGAACGGACTTAAGGCCGTTGGTAAGTATGGATATTGTGTTGGATTGAAGTTGGTGAGGAAGACCAATGGGGATTCTGTGTTGGCTACCTCGCCGGTTTCATGAGTCTCTGGCGAACGGCCGGCCAGTTTTGTTAATAATGTCAGGGATGTTGAACTTGTATCAATCGCAAACGCTGTGAGCGTATGGGCCAGCCCAACATATATTATTAGTGTTGGGCCGTGCTGGTGCCATACATTGGAACCGGCCAAGTTAGCCCAATTTGATAAATTATAAATCATGTTCCATGTTGCATTGCGGATCATAATAGTATAAAACGACGTTCAAATTATGTACTTTCAGTTAATATATGTTAAATTGTTAATGTAATATATATTGGTGAGTGCGTGCACTTCCGTGTGCACTCCCGTGCAGTTCCAGTGTTGGATTGTGCTACTGCTGCAATTTCAATTCAGTCCTTTCTCGGAAAGAAGAGAAATTCGATGCGGTGATTGCAAGCATTAATTCACCTGATATGCAAGCGTATAAGCTTCTCCGCGAGGCTGTCAGCATGGATTTGCTAGTGATCTGTAAGTCGTTAATTTCTTCGAATTTAATTTTTTAGTTATTGTTATTTCTTCGAATTGAATCATATTATATATATGTATATGTTGCTGGCCTGGATCATATCCCTGGAATTGTTGAAATGCAGTATTGTGTGATGAGGAAGATGCTGAGATGGCGGTTAGGCTTATAGAGCACGGAGCCTTAGCTTCCTCCTGCACAAGCCAATTTGTGATCAACTGA

**CDS sequences**

>IbHK1a

ATGGCTGTTGGATCCAATACAAGTCCTGTCAGCTCTGAATCCTTATCGCCTTCTATCACACCAAAGGGATCATTCCTTGAGAGAATTTTGTGCAGGATGTTTAGCTCTGGGATGTTTTGCACAAGCAACCAATCTCCTAGTAGCCGGAGAAATTTCAGTAGGGATGTAGAAGAGGAAGAATTTCAGGATGCAAGTACCCTTTGTTTATCTTCGTACTATAGCGTTTTTGTGGTCCGCCTTGCTATCATGGTCATGCTAGCAATTTTGATTGGATTGCTAACCTTACTAACATGGCATTTTACCAGAGTTTACACAACCAGGTCATTGAACACATTGGCATTTGGGCTTCGACATGAGCTACTTCAAAGGCCTATTTTGCGAATGTGGAACATCCTCAATTCTACTGTTGAAATAGCAACTGCTCAGGTTAAATTGTCAGAATATGTAATCAAACGATATAGCAAGCCTGTAAATCAAGCACAGCAAGCTGAGCTATATGAAGTCATGAGGGATGTAACATGGGCATTGTTTGCCAGCCGGAAGGCTCTGAATTCGATAACAATCAGTTACAAAAATGGTTTTGTCCAGGCTTTCCACAGAGACCACAGAAGTAACAATACATTCTACATATACTCTGATCTTTCCAATTATTCAATAAGTGGAACATACGATGTCAGTATGTTGACATCTCGTCAAGGGTGGAACGACCAATCTATACACAACAACACGACAGCAATTTGGTACAGAGAAACTCTGGATCCTCTGACAGGTGTCAGGGTTGGAAGAAAAAGTCAAATTCCACCAGATGAGTTAATCAATATTGCAGGAATTTCGCAAGTACCTGATGGTGCAGCGACATGGCATGTAGCGGTGAGCAAGTTCAGTGATTCGCCGCTGCTTTCTTCTGCACTTCCGGTTTGGGATGCATCCAATGAAAGCATAGTTGCTGTTGTGGGAGTTACTACAGCTCTTTATAGTGTTGGTCAATTCATGAAAGAAATTGTGGAATTCCATAGTGGACATATTTATTTAACATCTCAAGAGGGTTGGTTACTTGCTACTTCCACAAGTACTCCTCTCTTGAGGAACTCAACCACACGGCCCGAGCTGATAATGGCTGTTGATTCTGAAGACCCCGTAATAAAAGCTGGTGCTCAGTGCTTGCAGAAAGAATATGGGAACAAGTTTCCCCCTAGTAATGAAGTTCATATAGAGAATGCCAAGCTTGGAGATCAGATGTACTATATTGACTCTTTTTTCCTGAACTTAAAGAGACTTCCTATGGTGGGAGTTATAATTATTCCAAGAAAGTATATAATGGGAAAGGTTGATGAGAGAGCTTTCAAAACATTTGTGATATTGATATCTGCATCTATATGCATCCTATTCATTGGGTGTGTCTGCATATTCATATTAACAAATGGAGTATCAAAGGAAATGAAACTGAGAGCAGAATTGATAAGACAGTTAGATGCAAGAAGGAAGGCAGAGGCATCAAGCAACTACAAAAGCCAGTTTTTAGCAAACATGAGTCATGAATTACGAACACCTATGGCTGCAGTGATTGGCTTGCTGGACATTCTTATATATGACGATTGCCTAACAAATGAGCAATATGCAACAATTACTCAAATTCGCAAATGTTCCACTGCTTTACTTCGGCTTCTGAACAATATTCTGGACATCAGTAAGGTCGAATCTGGAAAGCTAGTGCTGGAAGAGACGGAATTTGACTTGACTCGTGAACTTGAAGGTCTTATTGACATGTTCTCTGTCCAATGCATTAACCACAATGTGGAGACTGTTCTAGATCTCTCTGATGAGATGCCAAAACTAGTCAAAGGAGACTCGGGAAGGGTTGTTCAAATATTTGCAAACCTAATCAGCAATTCTCTGAAGTTCACTACTTCTGGATATATTGTTCTGCGGGGATGGTGTGAGAGCCTGAATGATCTCACAAACAGCAGGAACTTTTTCTTCAATCAGAAGGATTCTTGGTCTGCACCTAAAGTGAAGTTGAAGCGAGCAGAAAGACGACCCTTCAAGAAAGATAGCAAAACAGTTCTTTGGTTTGAAGTCGAGGACACTGGTTGTGGAATTGATCCAAACAAATGGGAATCTGTGTTTGAAAACTTTGAGCAAGCTGATCCCTCAACAACTAGATTGCATGGTGGCACTGGTCTTGGCCTATGCATAGTACGTTCCCTGGTGAACAAAATGGGCGGTGAGATCAAAGTCGTGAAGAAAAATGGACCGGGAACTCTGATGCGGCTTTACCTGTTACTCAACGCTCCCACTGATGGTGCAGAGCAGCATAGCCCTCCAACTTTAGCAGAGCAGACGACGACTGTGTTGCTTGCACTTAATGGCAGAATGGGTAGGCTAATCATGTCCAAATGGTTAGAGAAAAATGGGCTACATACCTGTGAAGCAGCAGACTGGAATGAGCTAACACAGATGCTTCAGGGGGTTTTCGGATCCAAAAGTAGTCTGCAAGATTCTGGATGTGAACATTTTAGCGATAATAGTTCGACACTTTTGATCGTAGTTATTGACATTGGCCTTCTTAACTTGAGCACGAATATTTGGAAGGAGCAGCTAAATTTTCTGGATAAGTACAGTGAGAGAGCAAAGTTTGCTTGGGTTCTTTACCATGACACTTCCAATTCCATCAAATCCGAGCTGCGAAAAAGAGGGCATCTGATGATGGTAAACAGACCACTCTATAAAGGAAAAATGATTCAGATTTTGGAAGCTGCATTCACAAAAGATAAAAATCTCGAGTTGCAATCTGCAGAAAATACAGCTATACAAGTAAACATGCATGAATGCCATCACGAAATCGATGCCAGCCACTCATGTCTCACTAGCCCTGATGATTCTGACAAGTCAGAAACTGGGAATGTTAGACCTGTGAGGACATTCCTTGCTGAAGAGAAGCCCAACAAGCATTTCCGGAATGTATCTTCTTCCTCGATCTATGCCACACTTAACAACTACTTTGTCGACATCACTCAACCAAATCTGGGAGAAGACGATGCTTCAAGGGAGGATGATAGAAGAGAAAAAAGGAACAGATCAGAAGAACACTCAGGAAGCACTCGTCGTGTAGAATTAAGCACTGTCAGTGTCAGCTCTAGTAAGACAGCGAACGAACAAAAATCCCTGTCAGGGCTGCGAATCCTACTTGCTGAAGATACACCGGTACTTCAGAGAGTCGCAACCATAATGCTGGAAAAAATGGGAGCTACGGTTGTGGTTGTTGGAGATGGACAACAGGCCGTGGATGCTCTCAAATTCTGCAGAAACGGTCCAAACGAATCCTCCCAGGAAGACGACACCTCACCAACCTCACCAACCGAAGGATTCTGCTCTCCACCTTATGACTTGATCCTGATGGATTGCCAAATGCCAAAGATGGATGGCTATGAAGCAACAAAAGCCATTAGAAGATCGGAAATGGAAACTGGAACGCACATTCCTATCGTGGCATTGACAGCTCATGCAATGTCGTCGGATGAAGCAAAGTGCCTGGAGGTAGGAATGGACGCTTATTTAACAAAGCCCATTGACAGCAAGCTAATGGTCTCCACTATCCTTTCATTAACAAAGAGAAAAAACTGA

>IbHK1b

ATGGCGTACAGAGCCAATAGAACTCCCTCTATCAGCTCTGAATCATCATCAACCCCCAACACACCCGTGGGATCACTCCCGGAAAGAATTTTGCATAAAATGTTTGGTTTTGGAAACCTTTACAGACGAAACCAGTCTCCCACTAGAAGAAGAATTTTCCGCCGAGATGTTGAAGAAGAAGAAGAGGAGTTTCAATATGCAAGTACCCTTTGCTTGTCTTCATATTATAGCGTGTTTGTGGTTCGTCTTGCCATCATGGTCATGCTAGCAATTTTGATTGGATTGTTAACCTTACTAACATGGCATTTTACCAGAGTTTACACGAAAAGATCACTTAACACGCTAGCATTCGGTCTTCGCCATGAACTGCTGCAAAGGCCTATACTAAGAATGTGGAACATCCTTAATTCTACTGTCGAAATAGCAACCGCTCAGGTTAAGATGTCAGAGTTTGTAATGAGGCGTTATAGCAAGGCTATAAATCAAGAACAGCAAGTTGAGTTGTATGAAGCTATGAAGGATGTGACGTGGGCGTTGTTTGCCAGCAGGAAAGCTCTCAATTCGTTAACCATCAATTACAGGAATGGCTTTGTCCAGGCTTTCCATAGAGATCACAGGAGTAACAACACGTTCTACATATACTCTGATCTTTCCAATTATTCGATAAGTGGAACATATGATGCTAGTATGTTGTCGTCTCGTGATGGATGGAACGATCAATCTATACATGGCAACACATCTGCTATCTGGTACAGGGAGCCCCTGGATCCTCTTTCTGGTGTAAGGATTGGGAAACAAAGCCAAATCCAACCAGATGAGTTGATCAATATTGCGGGCATTTCTCAAGTGCCTGATGGTGCAGCCTCGTGGCACGTGGCTGTCAGCAAGTACTCCGATTCGCCACTGCTTTCTGCAGCACTTCCAGTTTGGGATCCATCTAATAAAAGTATAGTTGCTGTTGTGGGAGTTACTACAGCTCTTTATAGTGTTGGCCAATTGATGAAAGAAATCGTCGAGTTCCATAGTGGACATATATATTTAACCTCACAAGAGGGCTGGTTACTTGCTACTTCCACGAATACTCCTCTCTTGGTGAACTCCACAACAAGGCCGGAGTTGATCATGGCTATTGAGTCTGAGGACCCTGTGATACAAGCTGGAGCCCAATGCTTGCAGAAAGAGTACGGGAACAAGATTCCTCCCGGTCATGAAGTGGGAGTTATAATCATTCCAAGGAAATATATAATGGGGAAGGTTGATGAGAGGGCATTCAAAACATTGGTGATATTGATATCTGCATCTGTGTGCATCCTGATCATTGGATGTGTCTGCATATTCATATTGACAAATGGCGTTTCAAAGGAAATGAAACTTAGGGCAGAATTGATAAGTCAATTAGACGCAAGAAGGAAAGCAGAGGCATCAAGCAACTACAAAAGTCAGTTTTTAGCAAACATGAGTCATGAATTACGAACACCAATGGCTGCAGTGATTGGCTTGCTGGACATTCTTATATGTGACGATTGTCTCACAAATGAGCAATTTGCAACAATCACTCAGATACGCAAATGTTCAACTGCTTTACTTAGGCTCCTAAACAACATTTTGGATCTCAGTAAGGTAGAATCTGGAAAGTTAGTTCTGGAAGAGACAGAATTTGACTTGAGCCGAGAACTGGAAGGTCTTGTTGACATGTTCTCTGTCCAATGCATTAACCACAATGTGGAAACTGTTTTAGATCTCTCTGATGACATGCCAAAACTAGTTAAAGGCGACTCGGGAAGAGTTGTTCAAATATTTGCGAATCTATTAAGCAATTCTCTGAAGTTCACAAGTTCTGGCTATATCATTCTCCGGGGATGGTGTGAGAACCCAAACACTCTCGCAAACAGCAGGAAGTTTTCTGTCAACCAGAAGGACTCTTGGTCTGCGCCTAAAGTGAAGTTGAAGCCACACGGAAACCATGCCAGAAGACCTTCCAAGAAAGATAACAACAAAACTGTCCTTTGGTTTGAAGTTGATGACACCGGTTGTGGAATCGATACAAGCAAATGGGAATCTGTGTTTGAAAGCTTTGAGCAAGCTGATCCCTCCACTACAAGATTGCATGGCGGCACTGGTCTTGGTCTATGCATAGTTCGTACCCTGGTGAACAAAATGGGCGGTGAGATCAAGGTTGTGAAGAAAAATGGATCGGGCACTCTGATGCAACTTTGCCTCCTACTCAACACTCCTATAGATGTCACAGGACAGCACGGACATCTGAATTTTAGAGAGCAGACAATGACTGTTCTGCTTGCACTAAATGGCAGAATGGGAAGGCTAATTATGTCCCAGTGGTTAGAGAAAAACGGAGTTCATACTTGTGAAGCGTCGGAGTGGAATGAACTGACACAAATGCTTCAGAGGCTTTCTAAAACCAAAACCAACTCTCAAGGTGCAGGCAATGCAAACACCTCGCTTTTTGTCATAGTTATTGACATTGGCCTGCTTGACTTGAGCACAAACATATGGGAAGAACAGCTAAATTTTCTTGATAAATACTGTGGGAAAGCAAAGTTTGCGTGGATTCTTTACCATGACACTGCGAATACCATCAAATCTGAGCTCCGGAGAAGAGGGCATCTGTTGATGGTAAATAGACCGCTTTACAAGGGGAAAATGATTCAGATTCTGGAAGCTATAGTAAAAGAGAACAGCCTTGAGCTGCAATCTGCAGTAAACACAACAGAAGAAAATTTGCACGAATGCCATGAAATTGATGCCAACCACTCTTGTATTGCCAGCCCAGATGATTCTGACAATTCAGAAAATGGGAAGGATAAAGCTGTGAACGCATTCCGTGCTGAAGAAAGGGGGAATGAACATTTCGCCAAAGCCTCTTCCACGTCACAATATGGAACTCTCAACAACTACTTTGTTGACTTCACTCAAACAAATTTGGAAGACAATACATCACCTGAGGATCAACCAAGGCAGGCAAGGAATAGGTCGGTAGAATGCTTGGGTAGCCCTCATCCCAGAGAAAGTACTGTCAGCTATAGTAATGAAACAAACCAGCAAAAATCTCTGGCGGGGCTGACAATACTACTTGCTGAAGATACACCCGTACTTCAGAGAGTTGCAACGATAATGCTGGAAAAACTGGGAGCAAAGGTTGTAGTTGTAGGTGACGGGCAGCAGGCTGTGGATGCTCTCAAGTCAAGGGAAGAAGGCAGCTCAACAACTACCCAAACCGAAGGATCTTGTTCCATGGCTTTCGACTTGATCTTGATGGATTGCCAAATGCCAAAGATGGATGGGTATGAAGCAACAAAAGCGATCAGAAGATCTGAAGTAGCAACTGGTTCACACATACCAATTGTGGCATTGACAGCTCATGCAATGTCTTCAGATCAAGCAAAATGCCTGGAGGTGGGAATGGATGCTTATTTAACAAAGCCCATCGACAGCAAGCTGATGGTCTCCACAATCCTCTCATTAACGAAGAGCCTACAAGCTTGA

>IbHK5

ATGGTATCTGAGATGGAGAATGCTCATACTGAAGAAATGGACATTGAAGTCCTGTCTTCAATGTGGCCCGAAGATATTAATGAAGCGGGAAAACAATTTAATATTGAACAGCCGGGAGCAGACCTAGATATGCTGGAGGAGGTTACAATAAATGAGGAGGCGACGACTATAGTTGATTTCCAACGTCTCATGGAGCTTACGGACTATAGTGACAAAGGCTCTTCTCAGTTGGCATACTTGGTAAAGAATTGGGAGTATAAGCAGGCAAATGCTGTACGGTTGCTGAGAGAAGAGCTTGACTATCTTAGCAAGCAGCAGCAAGAATCCGAGCTCAAAAAATTGGAGATACTGGAGCAACATCGATTCGAGGAAGAAAGATACGGGGGTGATAAGCGTCCTGTTTCCATATTGGATGAGGATTTAAAATACATCTATCAAGATATCCCGAGGAGGAAAAAAGATGTGGTAGTTCAAGCTGAAAAGCTAGAAATAGAGGCCGAGTATGATAGCATCATATACTGGAAGCAGCGGGCCATGCATTTACAAAAACTGTTGGCAGCAAGCATTGAGCGAGAGAATATACTACTTGAGAAACTGCAAGAAAGTATAGAGAAACTTGAGCGGCAGTCCTCCCCTGTTGAAGAACTATCACAGGTTTTAAAGCGAGCAGATAACTATTTGCATTTTGTTCTTCAGACTGCACCTATTGTCATTGGTCACCAGGATAAGGAGCTGCGCTATCGGTTCATCTATAATCATTTCCCAAGTTTGCGTGAGGAGGATATAATAGGCAAGACAGATGTGGAGATTTTTTCCGGGTCTGGTGTAAAGGAGTCCCAAGATTTCAAAAAGGAAGTTTTGGAACGTGGGTTGCCTGCAAAACGGGAGATTACCTTTGAGACAGAACTATTTGGATCGAAAACATTTTTAATATATGTCGAACCAGTATTCAGCAAGGCCGGGGAGACTATTGGTGTAAATTATATGGGGATGGAAGTTCGAAAACGAGAAAAGATGGCAAAGCTTCGCGAGGAGATAGCTGTACAAAAGGCCAAGGAAACCGAACTTAACAGAACAATCCACATAACAGAGGAAACAATGCGGGCAAAACAAATGCTTGCAACCATGTCCCATGAGATAAGATCTCCTTTGTCTGGTGTTGTAAGCATGACCGAAATTCTTGCCACCACTAAACTAGAAAAGGATCAACGCCAACTTGTAAATGTCATGTTGTCTTCGGGTGATTTGGTTCTCCAACTGATAAATGACATCCTTGATCTTTCCAAAGTTGAGTCAGGGGTAATGAAATTGGAAGCTACGAAGTTCAGGCCAAGAGAGGTAGTAAAACATGTGCTGCAAACCGCAGCTGCATCACTGCAGAAACTACTGACCTTAGAAGGTTTTGTCGCAGAGGATGTCCCAACAGAGGTCATTGGAGATGTTCTTAGAATTCGCCAAATTCTCACCAATTTGATCAGCAATGCAATAAAATTCACTCATGAAGGAAAAGTCGGTATAAAACTGTATGTGGTTCCCGAGCCATCTTTGGGCGCAAAACAGGGATCTCATCAGAAGCAATCTTTAGATTCCCTGAAAAGTTCGTCAAACAATTGGAAAGAGGATAGGTGTTTGTCAGCGTCTCATGGGAAGCATGACCGAACGGCCTCTTTTAGTTATAAGGATGGAGAAGGAACTTTCGAAAATCAGATGCATAAAGATGGGTCCAACCATTCAGTAAGCAGTGGGGCTTTGGATGACGATTTAGACGCCCATCCTGATCAAGAGGAAAAAACTGTGTGGATATGCTGCGACGTTTATGATACCGGGATTGGCATACCTGAAAACGCTTTGCCCACTTTGTTTAAAAAGTACATGCAAGTCGGTGCAGATACAGCTCGAAAATATGGCGGGACTGGACTAGGCTTAGCAATCTGCAAGCAGCTGGTTGAGCTCATGGGTGGCCATCTCACGGTGTCCAGCAAAGAACACCACGGTTCTACTTTCACGTTTGTTTTACCGCACAAGGTTTCACCGTTGTGTGAAAGCTCTGATGAGAACGATGAAATGTCTGATATGGGTAGTCATGACACCTCGACTGATGCAAATGAAGACGATGCAAATTCTGGCTTCTTCCAGTTCCAACCGCGTACTTTGGGTTCTTTATTTTCTTCCCACGGTTCTGGAAGAGCCCAAAAGCTGTCACCGAATACTTTTGGGTTTAATACTTTGCAGAGTTGCAATGGATTGCCAAAGAACTCCTACACTTTCCCCGCTAATAGCGTTATGCTGAAAGATATGGGATCGGTAGAAGATGCCTGTTCGGTCATAGATGTTGACATATTGTCTGACCCCGAAAGTTCTTTCAGGCAAAGCTCACATTCTGATAACCCGAGCACATTGGAAAGAGACAAACACGCTCATTCTGGTAGCAATGGTCAATGCCATCACCACTCTTCCTATTCAACCGATTCCACGAGTACAAGGAAAGATGAGGACGTGAAAACAGCTGTTCAAGAAAAAAGACAGCCTGAGGGAAATTCTCCATGCTCCTCTGACAACAACCAGGAGTTGGGGCATCAGATTGACATTGTGAATAATGGAATAGAAGCTGTGCGGGCAGTTCAACGCAGCTGTTATGACCTTATTCTAATGGGGTGGGGCAACTCTAGTCAAGTTGGTCAAACTGTTAACTTGGTAACCACAAGGTTACAAGTTAAATTCCCAGCGGGAGTGACCTATTGGCCTTCTTGGTTTGAGCCGGATGTATGCATGCCTGTAATGGACGGACTTCAAGCCACAAGACTTATCCGATCATTTGAAGAAACGGGTAATTGGGATGCCGCCAGGACTGCTGGAGTCGAGGAGGTGCCTTCTTCAAGCTTGTCACTAAAACGTTCAGATTCCAAATCATCAAATGGAAGAATTCCAATCATTGCAATGACGGCCAATGCATTGTCAGAAAGCGCAGACGAGTGCTTTGCAAACGGCATGGACTCCTTCGTATCAAAGCCGGTCACGTTTCAGAAGTTGAAAGAATGCCTCCAGCAGTATTTGCCGCAGCGCCATCGCCTGTAA

>IbHK2a

ATGTCTATGAACTGTAAAGTCCATGGAATGAAGGGAGGCTTCTCTTCCAAATTCAGGCTCAAGAAGGCAAGAGAATCCCAGCATGGACCAAGTCGATGGAGGAGGCAATTATTGTTTCTTTGGCTCTTTTTTGTTGCCATTGGATTCATTTGGTTGTTGATTAGTTCCTCTTATGGGCGTTTGGGGAGGAAGGTAGAGGCCCCCCCACACTTGGATGGAGATACCACCAACTTTTTGCTTCAACATTTCAATGTTAGCAGGGAAGAAATTCATGCTCTGGCTTCCAATTTTCTTGATACAGATCAGATCTCATTGTTAAAATGTAGCGGAAGTCCCAGATATGAATCAAGCGTTCTCAAATCAGAAAACCAAGTTTATGAGAAAAAATGCAAATTGGGAGAGAAAATAGAGGCTTATGGCCAGTGTCCTGTTTCAGATGAGAACACTTTTAGGAATATTGACTCTGTACTACAACAGACATCTACACCATTTCTTTCACATTGTGCATCATCTTCAATTTCATCAGACCATCAGTTTTGTGAAAAGGAAACATTGCAAGTGAGAGCACTAGGGGATCAGTGTAAGGATATAGCCTTCTGTTTCACCAAGATATTCTGGTGGATCCTTCTTGGCATTGCTGTCAGCTGGAAACTGCGGTGGTTACGTGCAGAATCTGGTAGAAATGAACAGCAGAAATTAGTTTCGCAGCAAGAATTTGGTCAGCAACCTCAGCTACTAGAGCACTTGCAACAACAGCAAGCTCATGTTGCTTCTAGAGTTTCTCGAAAGTTGTGGGAAAAGCTTCTTGTTGCATTTGTATTATCTGGTGTGATAGCATCCATTTGGTTCTTCTGGTACTTGAATGAAGACATCATGTTCTGGAGGAAAGAAACACTGGCAAGCATGTGTGATGAACGGGCACGAATGCTGCAGGATCAATTTAACGTCAGCATGAACCATGTTCATGCCTTGGCTATTCTTGTCTCCACCTTTCACCATGGAAAGCAACCTTCAGCTATAGATCAGAGAACTTTTGAAGAATACACTGAGAGAACAGCTTTTGAGAGGCCACTCACAAGCGGTGTTGCCTATGCTCTAAGGGTTCTCCACTCAGAAAGAGAAAATTTTGAGAGGCAACATGGATGGGCAATTAAGAAAATGGAATCTGAGGATCAATCTTTGGCTCAAGAATATATGCCTGGGAATTTGGATCGTGCTCCTGATAAAGATGAATATGCACCAGTCATATTTTCTCAACAAACTGTCTCCCATATTGTTTCAATTGATATGATGTCTGGAAAGGATGACCGTGAAAACATATTGCGGGCAAGGGCATCTGGGAAGGGAGTCCTGACATCACCTTTTAAGTTATTGAAGTCCAATAACTTGGGTGTAGTACTTACATTTGCTGTATATAACACTCATCTTGCTCCCGATGCTACACCAGATCAACGTATTAATGCTACTGTCGGGTATATTGGTGCATCATACGATGTCCCCTCATTAGTTGAGAAGCTTCTTCACCAACTTGCGAGCAAACACACTATTGTTGTAAATGTTTATGATACAACAAACACACATTCTCCAATTAAAATGTACGGTGCAGATGAGACCGAGACAGAATTATTGCATGTTAGCAGCCTTGACTTTGGAGACCCTGCTCGGAAGCATGAGATGCATTGCAGGTTCAAGCAAAAACTTCCCCCGCCCTGGATAGCAATAGGAGCTTCTATCGGTGTTCTTGTAATCACCTTGCTTGTTGGTCATATTTTTCACGCTGCAATAGCTCGGATTGCCAAATTTGAGCATGACTATCAGAAGATGATGAATCTCAAGCATCGTGCTGAGGCTGCAGATATTGCAAAATCCCAGTTTCTTGCTACAGTTTCTCACGAAATCAGGACCCCAATGAATGGTGTTTTAGGCATGCTTCAGATGCTCATGGATACAAATCTCGATGCTACACAACGGGACTTTGCGCAGACTGCTCATGCTAGTGGGAAGGATTTGATATCTTTGATCAACGAGGTATTGGATCAGGCTAAGATTGACTCAGGACGTCTTGAGTTGGAAGCTGTACCTTTTGATCTGCGAGCTGTACTCGATAATGTCTTATCACTTTCATCTGGAAGATCTCATGAAAAAGGGATTGAGGTCCCAAAAATGGTTGTTGGAGATCCCGGAAGGTTTAGGCAAATAATCGCGAATCTTGTTGGAAACTCAATCAAGTTCACAAAAAACAAAGACGGGCATGTGTTTGTCACGATGCATTTAGCAGATGAAGTGAGGTGCCCGCTTGATGTGAAGGATGAAGTCTTGAGACAGAGCTTATCCCTCGTTGAAGACCAGACGAACAGATCTTTCAACACATTGAGCGGGTTTCCGGTAGTTGACAGATGGAGAAGTTGGCAAAATTTTAAGAAGCTTAGTGAGGAAGAAAGTGACAAGATCAAGTTGTTAGTGACAGTTGAAGATACGGGTGTTGGAATTTCTCTTGAAGCACAAGGCCGCATTTTCACGCCTTTTATGCAGGCCGATAGTTCAACGTCTCGAACATATGGAGGGACGGGAATAGGATTGAGCATCAGCAAGCATTTGGTGGACCTTATGGGCGGGGAGATTGGATTCTTCAGTGAACCAGGCACCGGCAGTACCTTTTCTTTCACCGCAGCCTTTTCGAGAGACCAAAGAGGTTCGGTAGAAGCAAAGTGGCAACAATATGATACAGGTGTTTTAGACTTTCACGGGCTAAGGGCATTGGTGATAGATGGCAAAAGAATTCGAGCCGAAGTCACCAGATACCACCTTCAAAGGCTGGGATTAAACGTGAAGATAACTTCCACGGTAGATCATGCGTGTTCATATCTATCTACTTGTTCAAAGACAAGTGAACCCGAGCATTTGGTCATCATGATTTTCATCGATAAAGACAACTGGGATACGGAGAATTCTTTTGCACTCCGTAACATTGTAAAGGATCTTAGGCCATATGGCTCAACGGCTCTCAATGGAGCCACGCCAAAATTGTTTCTGTTGGCAACAGAAATGAGCTCGACAGAAAGCAATCAGCTTAAATCGGATGGGCTGGTTGATAACGTATTAATAAAACCTATTCGGTTGAGTGTGTTGGCGTCATGCTTGCAAGAAGCTACTGGCTTTACGTATAAGAGGCAAGTGACAATGCCAAAACCGTCGACTCTTGGAAATCTGCTGAAAGAAAAACAAATTTTGGTCGTGGACGATAATATTGTAAATAGAAGAGTGGCAGAAGGTGCCTTGAAGAAGTATGGCGCTATTGTGACCTGTGTTGATGGCGGGAAGGCTGCTTTGGCACTTCTTAAGCCACCTCACAACTTTGACGCTTGCTTTATGGACCTCCAAATGCCCGAAATGGATGGGTTTGAGGCTACTCGACAAATCCGCAAGCTAGAGAGCGAATATAAAGAAACAATAAATTCTGGTGAGATATTGGTTGACGCTCCTGGCAAACTGGCTCATTGGCGTTTGCCAATATTAGCAATGACGGCAGATGTTATTAGGGCATCGAATGAAGAGTGCATGAGATGCGGGATGGATGATTATGTATCAAAACCATTCGACGAAGGGCAGCTATATTCAGCATTGGCGCGCGTTTCTTTGAATCGGGATGATTATCATCGGTCAGACTACACTTGGCCTTTTGTCCCGTGCGGAACGAATGCTAACCCCGCGTTTTTGCAAGGATGTTTCTTGATTTCTACTCGGCCATGCGTAGTGATCCCGTCTTGTTTGGTTCAGAAACTGCACCGCAATGGCACCCGGACCAGACAAACCATTCGGGTCGAGTTCCATTTGCAAGGGTTTGTGTACTTGGAGCAACATGGCAATGAACATTGGTTTATGGCTCTGTGA

>IbHK2b

ATGGGTAAAGCTTTGATGGGATCAGTTGGTGAGAATAGACAACACCAGGAGCAGCAATTTTTTGTGCTTGCTGTGGCCCATGCTCTTGTTGAAGTCACTAATTGGTGGGCTTTGAAGAAAATGTCTTGGAACTGCAAAAACCTTGGCATGAAGGGAAGCCTCTCTTCCAATTTCAGGCTGAGGAAGTTACTAAGTGGTGGGTGGAGATGGAGGAGAAAATATTTGATTTTGTGGCTCATATTTGTTGCCATTGGATTAATTGGGTTGCTGATTAGTTTGAATAATGGGCATATGAGGAGGAAGGTAGAAGCTCCAGACTTGGATGAAGATAGTACTAACCTTTTGCTTGAACATTTCAATGTGAGCAAGGAACGCATTCAGGTGCTAAGTGCAGAGAATGTTGTGTACCAGAAGCAATATGAACTGGCAATCGAGAAATTAGAAGCAAATGGCCAGTGTCCTGTTCCAGATGAAAACACTCTTACGAACCTTGACATTGTAGTGCAACAGATACCTTTACCAATTTCGCATTGTGCATCGTTGGCAACTTCATCAGATCACCAGTTCTGTGAAAAGGAACCGCTTCAAGGGAGGGCACTTGGAGATCAGTGCAAGGATGCAGCCTTCTATTTCACAAAAAAACAAGTTCAGCAGCAAGAATTGCCTCAACAACCTCAGCTACTTCAACACTTGCAGCAGCAACAAGCTCAGGCATCCTCAAGAATTGCTCGGAAGTGGTGGGAAAAACTTCTTGTTATTTCTGTCTCAGTTGGAGTAATGGGATCCATCTGGTTATTCTCACACCTGAATGAAGAATTCACAGTGAGGAGAAAAGAAACAATAGCAAGCATGTGTGATGAAAGAGCCCGAATGCTACAGGATCAATTTAATGTCAGCATGAACCACGTTCATGCATGGGCTTTTCTAGTCTCAACGTTTCACCATGGAAAGCAACCTTCAGCTATAGATCAGAAAACTTTTGAAGAATATGCTGACAGAACAGCTTTTGAGAGGCCACTTACCAGTGGGGTTGCCTATGCTATAAAACTTTGCCACTCAGAAAGGGAAAATTTTGAGAAGCAGCAAGGATGGACCATAAAGAAAATGGAATCCGAGGATCAAAGTTTGGCCCAAGAGTATATATCTGGGAACTTGGATCCTGCTCCAATTCAAGATGAATATGCACCTGTTATATTTTCTCAGCAAACAATCTCCCATATCGTGTCAATTGATATGATGTCTGGAAAGCATTCAAAAGAACTGAATCTTGGACAGGAGGGGTTAGTTTGGTCAGTGAGCATTCCTAACTTACAACCAGTAGGTTTGTATAACACTGATCTTCCTCCTGATACAACACCAGAGGAACGTATTAATGCTACTCTTGGGTATTTTGGTGCTGTGTATGATTTCCCCTCATTAGTTGAAAAGCTTCTCCACCAGCTAGCAAGCAAGCACACTATTGTTGTAAATGTTTATGATACAACAAATGCATCTGCTCCGATTAGAATGTATGGAATGGAAGAAGCTGATTTGGATGAGACTGATAGAGAATTAGTTCATGTTATCAATCTTGATTTTGGAGATCCAGCTAGGAGGCACGAGATGCATTGCAGGTTTAAGCAGAAACGCCCTCCGCCCTGGACAGCAATAGCTGCATCCATAGGAGTCCTTGTAATCACTTTGCTTCTTGGTCATATTTTCCATGCAGCCATAAACCGGATTGCAAAATTTGAGCGTGATTATCAGAAGATGATGGATCTCAAACATCGTGCTGAGGCTGCAGATATTGCAAAATCTCAGTTTCTTGCAACAGTTTCTCATGAAATCAGGACCCCAATGAATGGTGTTTTAGGCATGCTTCAGATGCTCATGGATACAAATCTCGATGCTACACAACTGGAATACGCACAGACTGCCCATGCTAGTGGGAAAGATCTGATATCACTAATCAACGAGGTGTTGGATCAGGCTAAGATTGAATCAGGCAGGCTAGAATTGGAGGCTGTAGCTTTTGACCTACGAGCTGTACTTGATAAAGTTTTATCACTCTGCTCCGGAAGATCTCATGAAAAACGGATTGAGGTCCCAGAAGTTGTTATTGGAGATCCAGGAAGGTTCAGGCAAATAATTACCAATCTTGTTGGAAACTCAATCAAGTTCACAAAGGAAAAGGGGCATGTGTTTGTTTCAGTGCATTTAGCAGATGAAGTGAAGAGCCCAAATGATGTGAAGGATGAAGTCCTGAGACAAAGCTTAACCCTTGTTCAAGACCGGCCAAACACGTCTTTCAATACATTGAGTGGGTTCCCCATAGTTGACAGATGGCGAAGTTGGCAGAACTTTAAGAAGCTCAGTGAGGAAAAAACCGAAAATATCAAGTTGTTAGTGACTGTTGAAGACGCTGGTGTTGGAATTCCTCTCGAAGCACAGGGCCGTATCTTCATGCCATTTATGCAAGCGGATAGTTCAACATCTCGTACGTATGGTGGGACAGGAATAGGACTAAGCATTAGTAAACGCTTGGTGGAGCTTATGGGTGGGGAAATTGGATTCTTCAGTGAACCTGGCACTGGCAGTACCTTTTCTTTCACAGCAGCCTTTGCCCGAGCAGAAGAAGATGATAAAAGCATTAGAGCAGAGGTCACTAGATACCATCTTCAAAGATTGGGATTAAACGTGAAGATAATTTCCAAAATGGATTCTTCGTGCTCACATCTGTCTACTTGTTTAGAAGCAAGTCCATTGGAGCATTTAGCTCTGATTTTCATTGATAAAGATAATTGGGATGACGAGACTTCTATTACACTCTCTAAGATTCTGAAAGAGCTGAGAGCCAACAGCTCCAACGTTGTTTCTGGAGTCATTCCAAAATTTGTTCTGAAACCATCATCTCTTGGAAGTCTGCTGAAAGACAAAAGGATTCTGGTGGTGGACGATAATGTTGTCAATAGAAGAGTAGCAGAAGGGGCCATAAGGAAGTATGGTGCAATTGTGAGCTGTGTAGATAGTGGGAAGGCTGCATTGGCACTACTTAAGCCACCTCACAAGTTTGATGCTTGCTTCATGGACCTCCAAATGCCAGAAATGGATGGGTTTGAGGCTACTCGACAGATCCGCTGTCTAGAAAGCAAATATAACGAGAATATCAATTCAGGAGAGGTGTTGATTGAAATGCATGGAAAAGTGTCTCATTGGCACACACCAATATTAGCAACGACAGCAGACGTTATTCAAGCAACAAACGAGAACAGTGGCGGTTCTTTGAGTCTGGTTGATTTGGTCATTTGGATTGACATTTGGCTGTTCCCACTTCTCAGACCTAGGCTGTTCAGATATGACTCTATTGCTCGGTCATGGACATGGACAAAGTACGCCTATCTTGTCTGTCCTGTGCGTAAAGAAAGTCAATCCAAGAAGCAGAAGTGCAGGTCCGGTGAAAATCACCTGAGAGATGATATGGCTTCTCTACCCCGAGAAGTTTCCCTCGACATAATCTCCAGGCTTCCCATTACATCTCTCGTGCGATTCAGGTCAGTATGCAAATCCTGGCACAACTTGTCTCATGATCATCAGCTTGTTCATTTGCACCTGTCTCGAGCATCAAACGACAATCCATGCCTCATATTTCACTGTAAATATCCCATCAGAAACTGGCTTTACTTTGTTTTGTTGTCTGGTCGTGATGATGATGATGAGCGAGTAGTGAGAAGAATCGATCCCCCTTTTGCAGCAGACTTTAATGTGGTAGGATCATGTGCCGGCCTGTTATGTCTAGCTGATTCTTTGTTCCACTCTTCTCTCTTCATATATAATCCTTTTACTGGGAACCACAAAGAACTCCCAAAATCCATTGTATTTCAGCACCAGGAACAAAAGGTGGTTTCTGGATTCGGATTTCACCCGATTTCTAAGCAGTACAAGGTGATCAAGATTGTCTATTATGCCACCGACCCGAGCTACTGCAGGCCTTCTGGTAGGGTCAGGACTCGCTGCTTCAACCAATCAGATGTTCAAGTGCTCAGCCTTGACAGCAGCAATTGGAGAAGCATTGGAGAAGCCCCTTACTGGCTGGAATTCGGGTCAACCGGGGTGCTGGTGAACGGAAGGCTGCACTGGTTAAGAAGAAATTCCGGGTATTATCTCGATGGGAGCATTGCATCCTTCGATCTAGCTGAGGAGCGGTTTCAGGACATCCCGAAACCTTATTTTGGCGAGATCTGCAGCCTTATGGTTCTTCAAGGTTGTCTTTCTGGCGTGACATTCGATAATAGGTGTTTGAAGATTTGGGTCATGAAAGAAGAGTCCTGGGTGAAACAGTTCACAATTGAGACTTCACTAATCCCCAGTTTTAACTATCCAAAGCTACCTTATAAGCTGTGGAAAGATGTTTTATGGTGCATCCCTGCAGTGAAAGTTCTGTGCCTTATGAAAAATGGTGAGTTGCTGATACAGTGCAAAGGTGTTGGCCTGGTTGCATATAATCCTGAGAGTGGTGTGTTTAGGCATCTAAACTTTCCTGGGCTGCCTAATATCTTTCTCACAATTGTTCATCTTGCTAGCCTTAATTGGATTGATATTGCTATTTGA

>IbHK3

ATGAATTGGTTGAGTAATGGTGGAGTCATGACCACCAAGACTTTGCTTGATGATGGAGAGGAGGTACTGACCAAGTTGTGGGGGAAGATCTCTGAGAACATCTCCAAGATCCAGCATAGTTACTCTCAGTATATTGGGTCCAAGAAAGTAAGGAAAAACTGGTGGGGTCTTTTGGTGATATGGCTAGGTTTTGGGGCAGTTCTAGCTTTTTGTGCTTTCTGGTGTTTGAGTTCTCAAGCTATGGAGAAGAGGAAAGAGACACTTGCAAGCATGTGTGATGAGAGAGCTAGGATGCTACAGGATCAGTTTAATGTCAGCATGAACCATGTCCAAGCCATGTCCATTTTGATCTCGACGTTCCACCATGGCAAAAATCCTTCTGTTATTGATCAGAGGACTTTTTCGAGATATACAGAAAGGACTGCTTTTGAGAGGCCTCTGACAAGTGGTGTTGCATATGCTGTAAGAGTTCTCCACCCTGAACGAGAACAATTTGAAAGGGAGCAGGATTGGACAATTAAAAGAATGGACCCCCAATTTCATGAAAATGAGTATAATGTAGATAACCTGGAGGCATCCCCAATTCAGGAGGAATATGCACCTGTTATCTTTGCTCAGGATACAATTGCTCATGTAATTTCCGTTGATATGCTCTCTGGAAAGGAGGATCGTGAAAATGTACTGCGTGCAAGAGCCTCAGGAAAGGGTGTTCTCACTGCACCCTTCAAGCTACTCAAAACAAACCGACTTGGAGTAATACTGACATTTGCTGTCTATAAAAAGGATCTTCCCTCAAATGCAACTCCTAATGAGAGAATTGAAGCAACTTATGGGTATGCCATGTTACTTCACATGATTGTACCTTGGAGGGTCTTTGATATTGAATCACTTGTAGAGAAGCTCCTTCAACAGCTTGCAAGTAAACAAACCATCCTTGTTAATGTTTATGATACAACTAATCTCTCCGATCCTATAAGTATGTATGGTACAAATGTATCAATTGATGACCTGGAGCATGTTAGTTCCCTAAACTTTGGGGATCCATTCAGAAAGCATGAAATGCATTGCAGATTCAAACAGAAGCCACCATGGCCATGGCTTGCTATTATTACCTCCTTTGGCATCATTACAATCGTGTTGCTCTTAGGGCATATATTTCATGCAACAATAAACCGAATAGCAAAGGTTGAGGACGACTATCATGACATGATGGAGCTCAAAAAGCGTGCTGAGGCAGCTGATGTTGCAAAATCAGAGTTTCTTGCTACTGTTTCCCATGAAATCAGGACCCCAATGAATGGTGTTTTAGGGATGCTTCATATGCTTATGGACACTGAGCTTGATGTAACCCAACAAGATTATGTTAGAACTGCACAGGCTAGTGGTAAAGCTCTAGTTTCACTCATAAATGAGGTTTTGGACCAAGCCAAAATTGAATCTGGAAAACTCGAGCTTGAGGCAGTGTCTTTTGATCCGAGGGCAATTTTGGACGATGTCCTGTCACTTTTTTCTGGGAAATCACAGGAGAAAGGAGTTGAGTTTACTGAGAAGGGGCATATATTTGTTACTGTCCACCTTGCCGAGGAGGTGGTTGTTGAGCATGAATCAAGTTATGCTTTGAGTGGGTTCTCGATTGAGCACGAATCAAGTAGCACTTTGAGTGGATTCCTGGTAGCTGATAGACGACAGAGCTGGAAAAAATTCAAAGCTTTTCAAGAAGGATTCTCTTCTTTCAAGTTGACATCTGACCAGATCAATTTAATTGTCTCGGTTGAAGACACAGGCGTTGGGATTCCGTTTGAAGCTCAATCTCGTGTATTCACCCCCTTTATGCAAGTTGGCCCCTCTATTGCACGGATTCATGGGGGTACTGGTATTGGATTGAGTATAAGCAAATGTTTGGTGCACCTCATGAAAGGTGAAATTGGTTTCGTAAGCTTGCCAAAGACTGGGTCCACATTTACTTTTACAGCTGTTTTTGCTAATGGTTCTTTTAGTTCGAATGAGCTGAAGGGTCAGCACATTAATGACGAGTCCAACTCTGTTTTTTCTGAATTCGAAGGGATGAGAGCCTTAGTTGTGGATCCCAGACCTGTACGAGCCCAGGTCTCAAAGTATCACATTCAACGCCTTGGTATTTACGTTAAGGTGATTCCAGATTTGAATCATGGGTACACTTGTTTAAGCACTGAGAAAACAAATATAAATATTGTACTTGTGGAACAAGAAGTGTGGGATATGGATTCAGGGATGGCCACAGACGTGCTTTGGGGGGTTAACAACCGAGGAAATTACCGGAATGGAGGGCTTTCTGGTGTTCCTCTTTCTGAGCTCCTTCACAAAAGAAAGATACTTGTTGTGGATGACAACCCTGTGAACCTAAGAGTAGCTAATGCTGCCCTGCGTAAGTATGGTGCTGATGTGGTCTGCATAGATAGTGGGGAACAGGCAATCTCACATCTGCGGCCTCCTCATCGCTTTGATGCCTGTTTTATGGATATTCAGATGCCAAAAATGGACGGGTTTGAAGCTACAAAAAGAATCCGTGAACTTGAACGTCAAGCTAATAGTCAAAACGAACATGGTGAACTTCTGGTAAATGCCTCAAACTGGCATGTGCCCATTCTGGCGATGACTGCTGATGTAATTCATGCTACAAATGAACAATGCCTGAAGTGTGGAATGGATGGATACGTCTCGAAACCATTTGAACCAGAACAACTTTACCGCGAAGTTTCAAGATTCCAGAAAATGCATGTCTTTAATCAACACGGAATGTATATACAAGTCGAGAGTTGTTTGCGTGGATGCCATTCAAAGCTCAACCACGAGGGAAGAATCTGA

>IbHK4

ATGGGTCAGAAGATTCATAGCCAAAGTCACCACCACCACACTGTGGCTATGAGGTTGGGTGAGCAATTGAGCAGTAAGAGGAAGTACACATTGATCTGCAGGAATAGGCTCCCACAGTTGTTGGGTTGTTGGATTCTTCTCATATTCTTTGTGAGTAGTTGGATATTCAATAATATGGATGCTACCCACAAGGAGAAGAGGAAGGAGGCTTTGGTGAGCATGTGTGATCAGAGGGCTAGGATGTTGCAAGATCAATTCAGTGTCAGTGTTAACCATGTCCATGCCCTTGCCATCCTTGTCTCCACTTTCCATTACTACAAGTCCCCTTCTGCCATTGATAAGCTTAGGTTTTTAGTTGAGATGGAGCAGATGCTTCAATTTGGTATTAGAGCCAAACTCATGTCAAAGGAAACTTTCGCTGAATACACTGCCAGAACAGCCTTCGAGAGGCCGTTGTTGAGCGGGGTAGCCTATGCGGAGAGAGTTCTTAATTCGCACCGGGGGAGCTTCGAGGATCAGCACGGATGGACTATTCGGACGATGGATAAAGAGCCTTCACCGATCAGGGACGAGTATGCCCCGGTCATACTCGCACAAGAAACTGTTTCTTACCTCGAGTCGCTTGACATGATGTCAGGGGAGGAGGACCGGGAAAATATCTTGAGGGCTAGGGCTACTGGGAAGGCTGTTCTTACAAGTCCCTTTAGGCTTCTAGGCTCTAATCACCTTGGTGTCGTTTTGACATTCCGATACCTTGGTGGGGCCTTTGATGTCGAGTCTCTAGTTGAGAACCTGCTCGGCCAACTTGCTGGGAACCAAGCAATCGTCGTGAATGTTTACGACATTACCAACGCTTCTGATCCTTTAGTCATGTATGGACAACCGGGCGAAGAGGGCGACCTGTCCCTGACACACGTGAGCAAGCTTGATTTTGGCGATCCATTTCGTAAGCACGAGATGATATGCAGGTATCTTCAGAAGGCTCCGACAGCGTGGGCTGCAGTAACCACTGCATTCTTTATCTTCGTGATCGGCTTTTTGGTTGGATATATGATATACGGTGCTGGAATTCACATTATTAAAGTCGAGGATGATTTCCACAAAATGGAGGCATTGAAGGTTAAAGCCGAAGCTGCTGACATTGCAAAATCTCAGTTTTTAGCTACCGTTTCACACGAAATAAGAACTCCCATGAATGGAATCTTAGGAATGCTTGCTCTGCTTCTTGACTCGGATTTGAGTTCGACTCAAAGGGATTATGCTCAAACGGCACAAGCCTGTGGAAAAGCGCTGATAACATTGATAAACGAAGTGCTGGATCGAGCAAAAATTGAAGCGGGGAAATTAGAACTCGAGATCGTCCCGTTTGATCTTCGCTCAATACTCGATGATGTTCTCTCTTTATTCTCCGAGAAGTCTAGGAAGAAAGGTGTCGAGGTTCCTGAAATTGTTTTCGGGGATCCCGGAAGATTCAGACAAGTGATAACAAATTTGGTTGGCAACTCTGTCAAATTCACTGAACGAGGGCATGTATTTGTTCAAGTTAGTCTAGCCGAAGAAGCAAAGGCAAAATCCGAAGCATGCTTGAATGGAGGATCTGAAAGATTTATACCGTCGAGTGGGTATCATTGCGAAACCCTTAGTGGTTATGAAGTTGCCGATAACCGGAATACTTGGGACAATTTTAAGCATGTAATTCCCGATGAACCGTTGTACTATAGAGCTGCGAACAAACTGATGACTGATGATGGGTCTCAGAATGTTACTCTGATGGTATCTGTTGAAGATACTGGGATTGGGATCCCGTTACATGCACAAGACAGAGTTTTCACGCCCTTTATGCAAGCCGACAGTTCCACTTCTAGAAACTATGGAGGAACCGGGATTGGATTGAGCATTAGCAAGTGTCTAGTTGAGCTGATGGGCGGTCAGATAAACTTTATCAGTCGTCCCGATGTAGGAAGCACATTTTCTTTCACGGTTAACTTCCAAAGATACGAGACGAATGGCAGCGTTGATTTGAAAAAGGGTCTTTCCGACGATTTGCCTATGTCGTTTAAAGGACTAAGAGCTATTGTCGTGGATGGGAAGCCAGTTAGGGCTTCCGTAACAAAGTATCATTTGAAGAGACTCGGGATCCTGGTCGAGGTGGTGAATAGCATTAAGAAAGCTGCAGCGGTATTCGGGAAAAACGGTTCCCTGATTTCCAAAGGTCAACTTCAGCCGGATATGATTCTTGTCGAGAAAGATGTGTGGATATCTGAAGATGGCGGGGGGCTGAACTTACAGATTCCGAACTTGAAACCGAATGGGCACACATACAAGGTGCCTAAGATGATTCTTCTTGCGGTGGATATTTCCAGTGCGGAGTTTGAGAAGGCTAAAGCTGCGGGATTTGCGGATACCATCATAATGAAGCCTTTGAGGGCAAGCATGGTGGGGGCGTGCCTTCATCAGGTGTTGGGAATGGGAAAGAAGACTCAAGGAAAAGACGCGTGCAACAAATCAACTCTTCGTGGCCTACTTTGCGGCAAAAGAATCTTGGTGGTCGATGATAACCGGGTAAACCGGAGAGTTGCAGCTGGCGCACTCAAGAAGTTCGGGGCTGATGTCGAGTGTGCGGAGAGCGGGGCAGCTGCTCTTGCATTGCTTCAGCTACCACACAATTTTGACGCTTGCTTCATGGACATTCAGATGCCCGAAATGGACGGATTTGAAGCCACTCGTCGCATTCGTAAAATGGAAAACGAAGCTAATGAGCGGGTGAACGGAGGACTAGAGGGAGAAGGAAGGCACAAGTGGCACGTCCCGATATTGGCCATGACTGCCGATGTCATTCATGCCACACTAGACAAATGCCTCAAAATCGGGATGGATGGATACGTCTCAAAGCCATTCGAGGAAGAGAATCTCTACAAGGCCGTGGCCAACACGGGTCTCCTGAAAAGTGCATCTGAGCAGCAAAGTTTTCCGCGTTCTACTGGTGCGGTGTTGGAAGCCGTGGATGTCCTGACGAAGCGTCGTTCAGATGTGTTCCTGGGGATGATAACAGGTTGCCCCAGGAATGTTATTATGTTCCTTCTCCAGTTCTCCCTCCCAGGTGTCCCTCCTGCTGCTGCTGATGTTGATAACAAGCCTGGAGATGATTGTCCCCCAACTTGGACAATCAATGTGAATGAAGTAAGAACGCTGAAAGTAAGCAATATCTCAGTGTCTGCTTCTGAGGATGATATCAAGGAATTCTTCTCTTATCCTGGGGATCTGCATTTCATTGAAGTGCAGAGGGAAAGTGACACCACTCAATTTGCATATGTCACATACAAGGATCCAAAGGGAGCAGAAACCGCAATGCTTCTTTCGGGAGCTGTCATATCTAATCTTTCCATTTCTATAGCACCAGCTGAGAACTATGATCTGCCTCCTAATGCTCCTCCTCTAACCCTAGGAACACAGCCAGAATCCGATTCTGATGCTGTCAAGAAGGCGGAGGATGTTGTAAGCTCCATGCTTGCCAAAGGCTTCATCCTGGGAAAGGATGCCGTAAACAAGGCAAAATCTTTGGATGAAAGTTACCATGTGACATCAAATGCCTCAGCTACGGTTGCTTCTGTTGACCTTAAAATGGGAATAAGTGAGAAGCTAAGCATAGGGACAGCTGTAGTTAACGAAAGGGTTAAGGAGATGGACCAGAAGTACCAAGTTACAGAAAAGGCAAAATCTGCACTTGCGGCAGCTGAGCAAAAGGCGAAAGCAACAGAGGACGTGAGCACAATGACAAAGGAAAAGGTTGAAAAAACCGAGGAGGAGAGACGGGCTGCAATTGGTAATGATTCTGAAGCTGCCCTTCTTGATCAGTCTTTGGCTGGAGATCCTGCCCCTAATTACTCTACTGATAGCAAAAAACTCGTATAA

>IbERS1

ATGGAGTCTTGTGACTGTGTTGAGATTCTGCTTCCAACTGATGAGCTGTTGGTGAAATACCAGTACATATCAGATTTCTTTATAGCGTTTGCCTACTTCTCAATTCCATTGGAGCTTATTTATTTTGTCCACAAATCAGCCTTCTTCCCCTATAGATGGGTGCTGATGCAATTCGGGGCGTTTATTGTACTTTGTGGAGCAACCCATTTGATAAATCTCTGGACATTCTCTTCGCACTCGAAAACAGTTGCTATACGTGAAAACAAGAGAATTGTTCTTGAAAACGCGCGGGCAGAAGAGCTTGACAGGGAAATGGGGCTTATTATAAAGCAAGAAGAAACTGGGAGACATGTTAGAATGCTGACCCATGAAATTAGAAGCACGCTTGACAGGCATACGATATTAAGAACTACTCTTGTTGAGCTAGGTAGGACCTTGGACTTGGCGGAATGTGCTCTGTGGATGCCAACACAAAGAGGGATGGTTTTACAGCTTTCCCATACTCTTAACAATCTTATACCCGTTGGATCTACTGTGCCTATAAACCTTGGCATCATCAATGATATTTTCAACAGCTCAGGAGCCATACTGATTCCACATTCTTGCGAACTAGCAAAGATGAGGTCTACTAATACTGGGAGACACGTTCCACCTGAAGTTGCTGCTGTTAGGTGTTCCACTTATACATCTCTCAATTTCCAAATAAATGACTGGCCCGAACTCTCTGCAAAAAGTTATGCCGTCATGGTTCTTATCCTCCCAATGAACGGAATTAGAAAATGGCGTGAGCATGAGTTAGAACTTGTTCAAGTTGTTGCTGACCAGGTTGCGGTGGCTCTTTCTCATGCTGCAATTTTGGAGGAGTCAATGCGGGCGCATGATCAACTCATGCAGCAGAATATTGCTTTAGACTTAGCCCGACAAGAAGCAGAGATGGCTATCCATGCTCGCAATGATTTCCTAGCTGTGATGAATCATGAAATGAGAACACCGATGCACTCAGTCATAGCTTTGTGCTCACTGCTTTTAGAAACTGACTTAAATCCCGAACAGAGGGTTATGATGGAAACTATACTTAAGAGTAGCAATCTTCTAGCAACACTGATTAACGATGTCCTAGATCTCTCAAGACTCGAAGATGGCAGTCTCGAATTAGAGAACGTAACATTCAATCTTCATGGAGTGTTTCGGGAGGTCGTTAACATGATTAAGCCTATTGCAGCAGTGAAGAAATTATCTACGACTTTATCTTTAGCTCTTGATGTTCCCATCCATGCTGTTGGAGATGCCAAACGTCTTACGCAAATCATGTTGAATGTTGCTGGGAATGCTGTTAAGTTCACAAAGGAAGGTCAAATTTCTATCGAGGCTTCTGTTGCGAAACCAGACTACATTAGGGGCAGTCGGCAGGGAGAGTTTTATCCACCGTCTACCGAGGGCCACTTTTATCTGCGTATGCAGGTCAAAGATTCGGGGTCTGGTATTAGCCCCCAAGATATTCCACTCATCTTCACCAAGTTTACAGAGGCTCGAAGTGCATCAAATCGAAGCAACAGCGGGGCTGGTCTTGGACTTGCCATTTGCAGAAGGTTTGTACAGCTGATGGGTGGTCATATTTGGATAGAAAGCGAGGGCCTAGGTAAGGGCACCACGGTTACTTTCATTGTTAAACTTGGCTCGTGCAACTATCCAAATGCCCCGGCCATTGTGGCCCCTCGAGTGAGAGCAAATCAAGGCAGCGACGATCTCTTCAAATATAGACAGTACCATAGAGCAGATGGTTCAATGTACGCCCCCGTTCCACGCTATCAAAGGAGTCTTTAG

>IbETR1

ATGGAGTCTTGTAATTGCATTGACCCGCAATGGCCTGCCGACGAGTTACTGATGAAGTATCAGTATATATCAGATTTCTTCATCGCACTTGCTTATTTTTCCATCCCAGTTGAGTTGATATACTTCGTTAAGAAATCTGCAGTTTTCCCTTATAGATGGGTGCTTGTGCAGTTTGGTGCGTTTATTATTCTTTGTGGAGCTACACACTTTATCAACTTATGGACATTTGGCATGCATACAAGAACAGTAGCTATAGTCATGACCACCGCAAAGCTATTGACTGCATTAGCTGCAGAACTTGATCGAGAAATGGGCCTTATCCGCACACAAGAAGAGACAGGTAGACATGTTAGAATGCTTACTCATGAAATTAGAAGCACTCTTGATAGACATACTATTTTAAAGACTACACTTGTGGAGTTGGGAAGGACGCTAGGTTTGGAAGAGTGTGCACTGTGGATGCCTACCCGTACTGGACTAGAGCTTCAGCTTTCATACACTCTTCGGCACCAAAATCCTGTTGGATTTACTGTACCGATTCACCTTCCTGTAATTAGTCAAGTATTCCATACAAATCGTGCAGTAAAAATATCGCCAAATTCTCCAGTAGCAAGGTTACGACCTGCTGGAAAGTACATCCCAGGCGAGGTGGTTGCCATCCGTGTCCCTCTTCTGCATCTCTCAAATTTCCAAATTAATGACTGGCCTGAACTTTCGACAAAACGCTATGCTTTGATGGTCTTGATGCTTCCTTCAGACAGCGAAAGACAATGGCATGTCCATGAGTTGGAGCTTGTTGAAGTTGTGGCTGATCAGGTTGCTGTTGCTCTCTCCCATGCTGCAATTTTAGAAGAGTCAATGAGGGCAAGGGATCTTCTTGTGGAGCAGAACATTGCCCTTGATCTGGCAAGAAGAGAAGCTGAAACAGCTGTTCGTGCTCGTAATGATTTCTTGGCTGTTATGAATCACGAGATGAGAACACCCATGCATGCAATAATAGCGCTTTCTTCCTTATTACAAGAAACCAAGTTGACGCCTGAACAGCGTCTGATGGTGGAAACAATCCTCAAAAGCAGCAATCTTTTGGCAACACTCATCAATGATGTCTTGGATCTTTCAAGACTAGAGGATGGTAGCCTGCAACTCGAGATAGGCACTTTTAATCTTCAGGCTCTCTTTTGGGAGGTCCATAATTTGATCAAGCCAATTGCCTCTGTGAAAAAGCTGTCTGTTACTCTAAGTTTGTCTTCAGATTTGCCCGAGTATGCCATTGGTGATGAAAAACGGCTAATGCAAGTTCTGTTGAATGTTGTTGGCAATGCTGTAAAATTCTCTAAAGAAGGCAGTATATCGGTTTCTGCTTTTGTTGCTAAATCGGAATTTTTAAGAGATCCTCAAGCTCCTGACTTCTTCCCAGTGATAACTGAAAATCACTTTTATTTACGTGTACAGGTTAAAGATACAGGAGTGGGAATTAACCCTCTGGACATTCCCAAAATCTTTAGCAAATTTGCTCAAAACCAATCATTGGCTACTAAAAAACTCTGGTGGTTCTGGGCTTGGCCTTGCTATTTGTTCGTAAATCTTATGGAAGGACATATTTGGATTGAAAGTGAAGGTCTTGGCAAAGGAGCTACTGCTATTTTTATCGTCAAACTTGGAATTCCTGGACTCTCAAATGAATTGAAGCCCACTCTTGTTCCCAAACTTCCAGCTAATCATATCCACACAATTTTTTTAGGACTCAAAGTTTTGCTTATGGATGATAATAGTATGAGCAGGATGGTGACCAAGGGACTGCTGGCGCACCTCGGGTTTGATGTAACAACTGCGAATTCTGGAGACGAGTGCTTGAGAGTGGTTAACCAGGAGCACAAGGAACCACGGACCGAAGCGATGAAAGAAAAATGCTTGCGGGCTGGTATGGATGGAGTAATACTAAAACCCATTTCTGTGGAGAAAATGAGGAATGTTTTGACCGAGCTTTTTGAGCATGGAGTTGTCCTCGACGCTCAATAG

>IbHKL2

ATGTCGAAGATATTAGCATTGAGGGTGTTGGTTTGGGTTTCCCTTGTTGCATTCACAGTTGCTGATAATGGGTTCTTCAGATGCAACTGTGATTATGATGGATTTTGGAGTATCGAGACGATTATGGAGTGGCAAAAAGTGGGCGACTTCTTGATTGCAGTGGCCTATTTTTCCATCCCGATTGAGCTTCTTTATTTCGTTAGCTGCTCCAATGCGCCGTTCAAGTTGATACTTGTTGAGTTTATTGCCTTCATTGTTCTGTGTGGAATGACGCATTTGCTCATGGGCTGGACTTATTATGGTCAACACTCGTTTCATTTGATGCTTGCCCTTACCATTTTCAAAGTTCTCACGGCTTTGGTGTCTTTTGCTACTGCTATTACCCTCGTAACGCTTATTCCCTTGCTGCTCAAGGTGAAGGTACGAGAGTTTATGCTGAAAAAGAAGACTTGGGATCTTGGTCGAGAAGTTGGGATGATAAAGAAACAGAAAGAAGCGGGATGGCATGTCCGGATGCTCACCCGAGAGATACGCAAGTCACTTGATCGACACACAATTTTGTATACGACTCTTATAGAGCTATCGAAGACGCTGGATTTACATAATTGTGCCATATGGATGCCAAATGAGGAGAAAACGGAAATGGACCTCACTCATGAGGTGAGAGGGAGGAGCTTCTTAGATGGGCATAACTTCCCTATTCCGGTCCTCGATCCAGTCGTGCAGGAAATTAAGCAGAGCGTTGAAGTGAAACTACTCAACCCCGATACACCACTTGCTGTTGCAAGTAGCGGAGGGGTTTGTGAGCCAGGAAGTGTGGCTGCGATTCGGATGCCAATGCTGAGGGTCGCAAACTTTAAGGGTGGGACCCCTGAGCTTGTCCCGCAATGTTATGCTATCCTCGTTTTGGTGATTCCTGCTGGACAGGGTAGATGCTGGGGCAACCAGGAAATGGGAATAGTAAAGGTTGTAGCTGATCAAGTTGCTGTGGCAATTTCCCATGCTGCAGTGCTTGAGGAGGTCCAAAATATGCGGGATAAATTAGAAGAGCAAAACCGAGCTCTGCATCAAGCACAGCAAGATGCTTTGAGGGCGAGTCAGGCTAGGAATTCATTCCAGATGGTCATGAGCAATGGCATGAGAAGGCCTATGCACTCAATTTTGGGCCTTCTCTCTGTTTTGCAGGACGAGCAATTAAATTGTGAGCAGAAACTTCTTAGAGATACCTTGGCGAAGACCAGCAATGTCCTCTCGACTTTAATTAACGATGCAATGGATACATCAACAAAGCAAAACAGAAGATTCCAACTTGAGATGCGATCCTTTCAATTACATTCTATGATAAAAGAAGCCATCTGCCTTGCCAAGTGCCTTTGTACTTTTAAGGGTTACGAATTTGTGGTTGAAGTGGACAAATCTCTGCCTAATCATGTTATTGGCAATGAAATTAGGGTTTTTCAGGTAATCCTGCATATGGTTGGAAATCTACTGAAGAGCAATGGTGGAGGGTGCATCAAATTTTCTGTAACCCGTGAAAAGGATGGTCAGGGAGGAAACGATTTAGGATGGAGAACAAAGTCATCTAGTGAGCATGTTCATGTCAGGTTTGAAATTGGGATTGCCGGTAATTGTTCTAAGCCCGAGGGTGTTTACAAAGCCTCACATTGTAGTGAGGCATATGGCCGGAGGGAGGTTGAAGAAGTTTTAAGCTTCACCGTGTGCAAAAAGCTAGTTCAGTTAATGCAAGGAAACATCTCGGTAGTGCCAAACCCTAAGGGCTTTCATCAAAGCATGGCAGTTGTTCTCGGCTTTCAACTCGGGCCATCCACTTCAGGCATGTCTGGGTGCAGCGAATCTTCCAGTCTTACACATCCGAGCTCCCTTTTGGCCGGGCTCAAAGTTCTGTTAGCCGACCATGATGGAATCAACAGGGGAGTAACCCGTAGGCTGCTCGAGAAACTGGGGTGCAATGTTTCTGCAGTTTCAACCGGATACGAATGTCTCGGGGCTCTAGGACCCGCAGCATGCCCATTCCAAGTTGTCCTTTTGGACCTTCACCTGCCTGAACTCGACGGTTTTGAAGTCACCATGAGAATTCGCAAGTTCAGAAGCCGAAGCTGGCCACTGATCATCGCTCTAACTGCAAATGACGATGAAGATGCGAGCGAAAGATGCATTCAGGTCGGGATGAATGGTATTATTCGTAA

>IbHKL5

ATGATGTTGAAGCTGCTAGCATCAGGACTGTTTATTTCGTCGTTCCTAATTGTGCTAGCAGCTGCCGATAATGGCGTTAGATGTAACTGTGATGATATCGAGGGAGTTTGGAGCATCGAGAGCATTTTGGAGTGCCAAAAAGTTAGCGACTTTTTGATTGCCGTGGCCTATTTTTCCATCCCGATTGAGCTTCTTTACTTCATTAGCTGCTCGAACATCCCGCTTAAGTTGGTGCTCTTTGAGTTTATTGCGTTCATCGTTCTGTGTGGGATGACTCATCTGCTTAGTGGTTGGACTTACTACGGTCAACATCCGTTTCAGCTTATGCTTGCCCTCACAGTTTTCAAAGTTCTAACGGCCATGGTCTCGTTTGCTACTGCTATAACGCTTATAACTTTCATCCCCTTGCTGCTGAAGGTGAAGGTGAGGGAAATAATGCTGAAGAAGAAGGCTCAGGATCTTGGGCGAGAAGTTGGAATGATAAAGAAGCAGAAAGAAGCAGGGTGGCATGTCCGGATGCTTACTCAAGAGATTCGAAAGTCACTTGATCGCCACACAATTCTGTATACGACACTGATTGAGCTATCCAAGACGTTGGATTTGTGTAATTGTGCGATTTGGATGCCTAATGTGGGAAAAACAGAAATGAATCTGACTCATGAGGTGAGGGGGAAGGACTTTTCGAATTTATACAACTACTCTATTCCAATCCTTGATCCGGATGTACAAGAAATCAAGAAGAGCGTTGGAGTGAAACTACTTGACCCTAATTCTGCACTTGCTGCTGCTAGTAGTGGAGGGACAAGCGAGCCCGGAGGTGTGGCTGCAATAAGGATGCCGATGCTGAGGGTTGCTAACTTTAAAGGTGGAACTCCCGAGCTTGTGCCTGCATGTTATGCAATTCTGGTGTTGGTGATTCCCGCTGGGCAGGGGAGATGTTGGGGCAACCAGGAGATTGCAATATTAAAGGTTGTAGCTGATCAGGTTGCGGTGGCGATTTCCCATGCTGCAGTGCTCGAAGAGTCTCAGCATATGAGGGAGAAATTAGTGGAGCAAAATCGATCACTACAACAAGCACAGAAGGATGCACTGAGGGCCAATCAGGCAAGAAATGGATTTCAAATGGTCATGAGCAATGGGATGAGAAGACCTATGCACTCGATTTCAGGCTTGCTCTCGATCTTGCAAGACGAGAAATTAAACAGAGAGCAAAAACTCCTTGTAGATGCAATGGCAAAAACCAGCAACGTCCTCTCAAACTTAGTAAATGACGTGATGGATACTTCGACAAAGGACAACGGAAAATTCCCACTAGACTTCAGGTCCTTTCAGCTACACTCTATGATAAAAGAAGCTGCCTGCCTTATCAAGTGCCTTTGTGCTTTTAAGGGTAATGATTTTGCTGTTGAAGTTGACCGATCCCTTCCCAATCGTGTTATGGGTGACGAAAGAAGAGTTTTTCAGGTTATTCTTCATGTGGTCGGGAATCTCTTGAAGATCAGCGGAGGAGGGTGCCTTAAATTTCGCGTAGTACCTGAAAAAGCAAGCCAGGGAGGAAACGATTTCCGATGGAAGACATGGAGATCAAACTCGTCTAGTGAAAATGTTTATATCAGGCTTGAAATTGGGATATGTAGTTATAAGTCTAGGACGGAAGGTGCAACATCGAACGTGTCCAGTCAGAAATATGGCAGTAGGGAGATCGAGGATGGCTTAAGCTTCAGCCTGTGCAGAAAGCTAGTTAAGTTAATGCAAGGAGAGATATGGATGGTCCCAAATTCGAAGGGATTTGATCAGAATGTAGCAATTATTCTTCCGTTTCAACTCAAGCCATCAATCGTTTTAGACATATTCGGGGAGTCCTCTAACTATACAAATCCGTATTTTCTGTTTGAGGGACTCGAAGTCTTGTTAGCCGACTACGATGATCTGAATAGAGCAGTAACTTGCAGGCTGCTCGAGAAACTTGGATGCATCGTTTCCACAGTTTCGTCTGGATACGATTGTCTCGGTGCTCTGGGCAATGGGGTATCCTCGTTTCAGGTAGTCCTTTTGGAGCTTAATTTACCCGATTTGGATGGCTTTGAATTAACCGTGAGAATCCGGAAGTTTCAAAGCCGTGGCTTCCCACTTATCATTGCTCTAACAGCAAGCAGCGATGAAGATGTGATCGGGAGATGCTTGCAAGTTGGAATGAATGGTATTATTCGTAAACCCGTTCTTTTGCAAGGAATTGCTGACGAGCTTCAACGAGTCAAAATGGTATTTGGAACACAAAACAGTTCCACCACCAAGAATCCAACACAGACATGCTGCATAGCACCGCACAGGATCAAATCGAGTCAGGTTATGGGTTCGAGTGACAAACGCTATTATGAGGCTGGGACCACTATGCCAAACGCTATAACCAGTAGCAAAGGTGCACCTTTATTTCCTTATTATGAGGCTAGTGGTGGACTTGGCCCTTCACCCTTCACCCCTAGACTGCCCTAA

>IbHKL4

ATGTCGGTGTTGAAGCTGTTGGTTTTGTCTGTCCACATTGCGATGGCAGCAGCCGATAATGGCTTTGCTCGGTGTAATTGTGAGTACGAGGGGTTTTGGAGCATTGAGAACATTTTGGAGTGCCAAAAAGTTAGCGACTTTTTGATTGCGGTGGCGTATTTTTCCATCCCAATTGAGCTCATTTACTTCATCAGCTGCTCGAATGTTCCGTTCAAATTGGTGCTCTTTGAGTTTATCGCGTTCATTGTGCTCTGCGGGATGACCCATCTGCTCAACGGTTGGACTTATTACGGCCAACACTCGTTTCAGCTTATGCTTGCCCTTACGGTTTTCAAAGTTCTCACTGCCATGGTCTCGTTTGCTACTGCCATAACCCTTATCAGCCTCATCCCGTTGCTGCTCAAGGTGAAGGTGAGAGAGCTTATGCTCAAGAAGAAGGCGTGGGATCTTGGTCGGGAAGTTGGGCTAATAAAGAAGCAGAGAGAAGCGGGATGGCATGTCCGGATGCTCACCCAAGAAATCCGCAAGTCACTTGATCGAGACACAATACTCGAGACGACTCTGAGCGAGCTGTCGAAGACGTTGGGTTTACATAACTGCGCTATTTGGATGCCCAATCAGGACAGAACAGTAATGAACCTGACTCACGAGGTGAGGGAGAGGAACTTTTCGGACATGAATGACTTCTTGATTCCAATACTCGACACCGATGTGCAAGAAATTAAGGCCAGCGATGAAGTGAAACTACTCGAGCCTTCGTCGCCTCTCGCTGCTGCGAGTAGTGGAAGGAGTAGCGAGCCAGGATGCGTGGCTGCGATAAGGATGCCGATGCTGAGGGTTGCTAATTTTAAAGGCGGAACGCCCGAGCTTGTCCCGGCTTGTTATGCTATTCTGGTTTTAGTCCTCCCGTCTGGACAGGGCCGATCTTGGGGCAGCCAGGAGATTGAAATAGTAAAGGTCGTTGCCAATCAGGTTACCGTGGCTATCTCCCACGCTGCCGTGCTCGAGGAGTCTCAGCATATGAGGGACAAATTAGCGGAGCAAAATCGAGAACTGCAGCAAGCGCAGCAGGGCGCACTGAGGGCGAATCAAGCAAGAAACGCGTTTCAGATGGTCATGAGCAACGGGATGAGAAGGCCTATGCACTCGATCTTTGGCCTTCTCTCGATCTTGCAGGAGGACGAGAACTTAAACAGCGAGCAGCATCTTCTTATAAACGCAACGGTGAAAACCAGCAATGTTATCTCGAACCTAATAACCGACGTGATGGATTGCTCAACAAAAGACAACCGAAAGTTCCCACTCGAGACTCGGTGCTTCGAGCTACATTCTATGATAAAAGAGGCGGTCTGTGTTGCCAAGTGCATCTGTGCTTACAAGGGTTACGAATTCTCCGTTGAAGTCGACAAATCCCTGCCTAATCACGTTATGGGAGACGAAAGACGAGCTTTCCAGGTTATTCTCCATGTAGTCGGGAATCTCTTGAAGAACAGTAACGGAGGGTGCCTTAAATTTCACGTAGTGCCCGAAAGGTCTAGTCAGGGAGGAAACGATTTAGGTTGGAGAACTTGGCGATCAAACTCATCCCGTGAGAATGTCTTTGTCAGATTTGAAATCGGGATACATGGAAACAATTCTCAGCCCGATCACACAACATCCAAAGTCTTGAATTCTAATCAGAAATATTGTGGAAAGGATTTCGAGGGAAGCTTGAGCTTCAGTGTCTGCAAAAAGCTAGTTCAGTTAATGCAAGGAGACATCTGGGTATCCCCAAATCCGATGGGGTTTGATCAACAAGTCATGGCTGTTGTTCTAGGATTTCAACTCAGACCCTCGGTCGTCATAGGCATATCGGAATACGCTGACTACGATGATGTGAATAGAGCAGTAACCCGCCGTATGCTAGAGAAACTCGGATGCATCGTTTCTTCGGTTTCATCTGGATACGAATGCTTAGGCTGTCTCGGCACTACAATATCCCCGTTCCAAATCGTGCTTTTGGATCTCCACCTACCCGATCTGGATGGGTTTGAAGTCACCATGAGAATCCGAAAGTTCAAAAGCCGTAATTGGCCACTGATCGTTGCCCTAACGTCAAACAACGATGCCAGCATCCGTGGAAGATGCTTTCAGGTTGGGATGAATGGCGTCATCTGTAAACCGCTATTCTTGCAGGGAATTGCAGACGAGCTTCAGAAAGTTATGTTAATTGCGAGCCGAACCCTATCTTGA

>IbHKL3

ATGTCGAAGATATTAGCATTGAGGGTGTTGGTTTGGGTTTCCCTTGTTGCATTCACAGTTGCTGATAATGGGTTCTTCAGATGCAACTGTGATTATGATGGATTTTGGAGTATCGAGACGATTATGGAGTGGCAAAAAGTGGGCGACTTCTTGATTGCAGTGGCCTATTTTTCCATCCCGATTGAGCTTCTTTATTTCGTTAGCTGCTCCAATGCGCCGTTCAAATTGATACTTGTTGAGTTTATTGCCTTCATTGTTCTGTGTGGAATGACGCATTTGCTCATGGGCTGGACTTATTATGGTCAACACTCGTTTCATTTGATGCTTGCCCTTACCATTTTCAAAGTTCTCACGGCTTTGGTGTCTTTTGCTACTGCTATTACCCTCGTAACGCTTATTCCCTTGCTGCTCAAGGTGAAGGTACGAGAGTTTATGCTGAAAAAGAAGACTTGGGATCTTGGTCGAGAAGTTGGAATGATAAAGAAACAGAAAGAAGCGGGATGGCATGTCCGGATGCTCACCCGAGAGAATACGCAAGAGAAAACGGAAATGGACCTCACTCATGAGGTGAGAGGAAGGAGCTTCTTAGATGGGCATAACTTCCCTATTCCGGTCCTTGATCCAGTCGTGCAGGAAATTAAGCAGAGCGTTGAAGTGAAACTACTCGACCCCGATACACCACTTGCTGTTGCAAGTAGCGGAGGGGTTTGTGAGCCAGGAAGTGTGGCGTCGATTCGGATGCCAATGCTGAGGGTCGCAAACTTTAAGGGTGGGACCCCTGAGCTTGTCCCGCAATGTTATGCTATCCTCGTTTTGGTGATTCCTGCTGGACAGGGTAGATGCTGGGGCAACCAGGAAATGGGAATAGTAAAGGTTGTAGCTGATCAAGTTGCTGTGGCAATTTCCCATGCTGCAGTGCTTGAGGAGGTCCAAAATATGCGGGATAAATTAGAAGAGCAAAACCGAGCTCTGCATCAAGCACAGCAAGATGCTTTGAGGGCCAGTCAGGCTAGGAATTCATTCCAGATGGTGATGAGCAATGGCATGAGAAGGCCTATGCACTCAATTTTGGGCCTTCTCTCTGTTTTGCAGGACGAGCAATTAAATTGTGAGCAGAAACTTCTTAGAGATACCTTGGCGAAGACCAGCAATGTTCTCTCGACTTTAATTAACGATGCAATGGATACATCGACAAAGCAAAACAGAAGATTCCAACTTGAGATGAGATCCTTTCAATTACATTCGATGATAAAAGAAGCCATCTGCCTTGCCAAGTGCCTTTGCACTTTTAAGGGTTACGAATTTGTGGTTGAAGTGGACAAATCTCTGCCTAATCATGTTATTGGCAATGAAATTAGGGTTTTTCAGGTAATTCTGCATATGGTTGGAAATCTACTGAAGAGCAGCGGTGGAGGGTGCATCAAATTTTCTGTAACCCGTGAAAAGGATGGTCAGGGAGGAAATGATTTAGGATGGAGAACAAAGTCATCTAGTGAGCATGTTCATGTCAGGTTTGAAATTGGGATTGTCGGTAATTGTTCTAAGCCCGAGGGTGTTTACAAAGCCGCACATTGTAGTGAGGCATATGGCCGGAGGGAGGTTGAAGAAGTTTTAAGCTTCACCGTGTGCAAAAAGCTCGTTCAGTTAATGCAAGGAAACATCTCGGTAGTGCCAAACCCTAAGGGCTTTCATCAAAGCATGGCAGTTGTTCTCGGCTTTCAACTCGGGCCATCCACTTCAGGCATGTCTGGGTGCAGCAAATCTTCCAGTCTTACACACCCGAGCTCTCTTTTGGCCGGGCTCAAAGTTCTGTTAGCCGACCATGATGGTATCAACAGGGGAGTAACCCGTAGGCTGCTCGAGAAACTGGGGTGCAATGTTTCTGCAGTTTCAACCGGATACGAATGTCTCGGGGCTCTAGGACCCGCAGCATGCCCATTCCAAGTTGTCCTTTTGGACCTTCACCTGCCTGAACTCGACGGTTTTGAAGTCACCATGAGAATTCGCAAGTTCAGAAGCCGAAGCTGGCCACTGATCATCGCTCTAACTGCAAATGACGATGAAGATGCGAGTGAAAGATGCATTCAGGTTGGGATGAATGGTATTATTCGTAAACCGGTTATTTTGCAAGGAATTGCTGATGAACTTACCAGAGTCCTGTTGCTAAAAAGCAGAAACATTGCATGA

>IbHKL1

ATGGGTGCTGCAATGTTGAGGTGGTTGTTTCTTGGGTTGTTAGTTTCTTCGATTTTCTCCGCGGTGTCAGCTATTGATTATCTCTGTTGTGATGACGAGGGATTGTTTAGTGTAAGTAACATTCTATTTATGCAGAAAGTGGGCGACGTCTTGATTGCAGTTGCTTATTTCTCGATTCCTATTGAATTGCTGTACTTCATTAGCTGCTCGAACATACCTTTCAAATGGGTGCTCGTTCAATTCATTGCGTTCATAGTGCTTTGTGGATTGACCCATTTGCTCAATGTGTGGACTATCAACACTCAGCCTTCCTTTCAGATGATAATGTCATTGACAGTCGCAAAAATCCTGACTGCACTCGTGTCCTGTGCAACTGCAATCACCCTTCTCACGCTTATCCCGCTTCTTCTCAAATTTAAGGTGAGGGAACTGTTTTTGAGACAGAATGTGTTGGAGCTGGATCAAGAGGTCGGGATGATGAAGAAACAGAAAGAAGCGAGTATGCATGTCCGAATGCTGACACTAGAAATTAGAAAGTCGCTTGATAAGCATACTATACTCTATACCACTCTGGTCGAGCTTTCTAAAACGTTGAATCTTCAGAACTGTGCAGTGTGGATGCCAAGTGGGAACAGAGCAGAGATGAACTTGACGCACGAGTTGAACCCCTGTTCGGCTAGAGAGCATCATTCCCTTTCAATTAATGACCCGGATGTGCTGGAGATAACAAAGAATGAAGGGGTGAGGTTACTGAAGCAAGATTCAGTTCTTGCAGCTGCGAGCAGTGGTGGGTCTGGTCAACCCGGTGCTGTTGCAGCTATTCGGATGCCATTGCTTCGTGGTTCAAACTTCAAAGGTGGAACGCCAGAGCTTATTGAAACGTGGCCCGTGGCTCTGTCCCATGCAACAGTTCTTGAAGAGTCTCAGTCGATGCAGGAGAAGCTGAAAGAAAGAAACCGTGTATTGCAACAAGCTAAGGAGGATGCTATGAAGGCTAGCCAGGCAAGGAATTCGTTCCAGAAGGTAATGAACAATGGAATGAGGCGACCAATGCACTCGATTTTGGGCTTGCTTTCCATACTCCAAGATGACAATTTGAAACCCGAGCAGAAAATTGTCGTCGACACATTGGTGAAAACCAGCACGGTACTTTCAACCTTAATTAGTGATGCAATGGAGATATCTGCCAAAGATGACGGGAAATTCCCTGTCGAAATGAGGCCCTTTCAACTTCATTCATTGATCAGGGAGGCCTCTTGTCTCGTGAAATGCTTTGCCATTTATAAGGGCTTTGATTTTTCCACAGATGTTCTGAGTTCTTTGCCTAATCAGGTGATGGGTGACGAGAAGAGAACATTTCAGGTTATACTTCATATGGTTGGGCATTTATTCAATGTCAGTGATGGAAACGGCTCCGTCATATTCAGAGTTGCTTCGGAAAGTGGAACTGAGGATGGGAATAATAAAGTTTGGAATACGAGAAAACCGAGCTCGAGTGATGACAATGTAACAATAAAATTTGAAATTGAAGTCACTATTGGAGATTCTCAATCAGGTACCTCGGTTTCAGTCGTTCCTTCTGGCAGGAAAAGGCATAACAGCAAAGATGTGAAGGAGGGCTTGAGCTTCACCATGTGCAAAAAGCTTGTGCAGTTGATGCAAGGAAATATATGGGTCTCCTCAAATTCTCGGGGCCGTGGACAAGGCATGACGCTGATTCTAAGATATCAGAAGCAATCCTCGATTAGAAGACGAATCTTTGAATACAGAAATCCTTCGGAGCAACCGCTTCCAAGCACAATGTTTGAAGGCCTACAAGTTCTTCTGGCTGACGATGACGGCGTAAATAGAATGGTGACTAAGAAGCTGCTCGAGAAGTTACGCTGCCAAGTATCCACGGTTTCGACGGGTTTCGAATGTCTAAGCGCCCTAGGCCCCTCGGCAACGTCCTTCCAAGTCATCATCCTAGATCTCCACATGCCGGAAATGGACGGGTTCGAAGTGGCGATGAGAGTGCGCAAGTTTCGCAGCCGCAACTGGCCGTTGATCATAGCCCTGACCGCGAGCTCGGAAGACCACATGTGGGAACGATGCCTCCAGGTGGGGATGAACGGTCTGATACGAAAACCCGTCCTCCTACAGAGACTCGCGGAAGAACTTCAGAGAGTCCTCCAGCGGGCTGGCACCGAAGTCATGTGA

>IbHKL6

ATGTCAACTTCCAGACCTAGTCAGTCTTCTAGTAATTCAGCGAGATCAAAACATAGTGCTAGGATCATAGCACAAACCTCTATAGATGCAAAGCTCCACGCGGAGTTTGAGGAATCGGGGGATTCCTTTGATTACTCTAGCTCAGTCCGAGTCACGAGTGTGGATGCTGGAGTGCAGAAACCTAGGTCTGACAAAGTAACCACTGCTTATCTCCATCAGATCCAAAAAGCCAAATACATCCAGCCATTTGGCTGTTTGTTAGCGCTGGATGAGAAAACTTTTAAAGTCATAGCATTCAGTGAAAATGCTCCCGAGATGCTTACCATGGTCAGCCATGCTGTTCCAAGTGTTGGAGATCATCCAGTTCTTGGTATTGGGACTGATATTAGAACCATTTTTTACCAGTCCCAGTGCAGCAGCATTACAGAAGGCCTTGGGATTTGGAGAGGTAGCTTGATTGTTGACTTTGAGCCAGTCAAACCATATGAAGTACCCATGACTGCTGCTGGTGCATTGCAATCGTATAAACTTGCAGCAAAAGCTATTGCTCGATTGCAGTCCTTGCCAAGTGGCAGCATGGAGAGGCTCTGTGATACTATGGTCCAAGAGGTGTTCGAACTCACAGGTTATGATAGGGTGATGATTTATAAATTTCATGATGATGACCACGGGGAGGTTGTTTCTGAGATCACTAAGCCTGGCCTTGAACCTTACTTGGGCTTGCATTATCCAGCAACCGATATTCCTCAAGCTGCACGCTTTTTGTTCATGAAGAATAAAGTACGGATGATTTGTGATTGCCGAGCAAAACATGTCAGGGTGGTCCAAGATGAGAAACTTTCGATTGATTTAACATTGTGTGGTTCCACACTTAGGGCTCCTCACAGCTGTCATTTACAGTACATGGAGAATATGAATTCAATAGCGTCACTGGTAATGGCAGTCGTAGTTAATGATGGAGATGACGAAGGGGAGGCCTCAGAATCTGGACGGATACAGAAGAGGAAAAGGCTTTGGGGCCTAGTTGTGTTCGCCATTCATGTCAACAAGGAACTAGAATTAGAAAACCAGATTGTTGAGAAAAACATTCTACGCACTCAAACTCTCTTGTGTGATATGTTAATGCGAGATGCGCCACTGGGTATTGTGTCACAGAGCCCCAATATAATGGATCTTATCAAATGTGATGGCGCTGCTTTGCTGTATAAGAGTAAGGTACACAGATTGGGAATTACCCCAACCGACTTTCAGTTGCATGATATAGTCTCTTGGCTCTCCGAGTACCATATGGATTCCACAGGTCTGAGCACAGATAGCTTGTACGATGCTGGCTTCCAAGGGGCTCTTGCCCTTGGTGATGCAATATGCGGGATGGCTTCTGTCAGAATATCTGACAAGGATTGGCTTTTCTGGTTCAGATCGCACACTGCTGCTGAAGTTCGTTGGGGTGGTGCGAAGCACGAACCTGATGAGAAGGATGATGGTAGGAAAATGCATCCAAGGTCATCGTTTAAAGCGTTTCTTGAAGTTGTCAAGACAAGAAGTTTACCTTGGAAGGACTACGAGATGGATGCGATTCATTCGTTGCAGCTAATACTGAGAAATGCTTTCGGCAAGGAGGCTGATACTATGGATACAAAAGCAAATGCAAATGCTATCCATTCAAAGCTTAATGACCTAAGAATTGACGGTATGCAAGAACTAGAAGCCGTAACAAGTGAAATGGTCCGCCTGATTGAAACGGCCACAGTGCCAATCTTGGCAGTTGATGTGGATGGGCTGGTGAATGGGTGGAACACAAAAATTGCTGAGTTGACTGGTCTAACTGTCGATGAAGCAATTGGGAAGCACTTTCTTACACTCGTGGAAGACTCCTCAGTTCATAATGTGAGAAAGATGTTGAGTTTGGCGTTGCAGGGGAAAGAAGAAAAAAATGTACAGTTTGAGATAAAAACACATGGGCAAAGATCAGAATCTGGTCCAATCAGCTTAATTGTGAATGCTTGTGCAAGCAGGGATGTTCAAGAAAGTGTGGTCGGTGTCTGTTTTATTGCACAGGATATAACTGGACAGAAGACTATTATGGACAAGTTCACACGAATTGAAGGGGATTACAGGGCTATTATACAAAATCCTAACCCTTTGATTCCCCCAATATTTGGAACCGATGAATTTGGGTGGTGTTCTGAGTGGAATTCAGCTATGACAAATTTATCTGGATGGTGCCGTGATGAAGTTATGGACAAGATGCTTCTGGGGGAGGTTTTCGGGACACAGAAAGCCTGCTGTCGTCTCAAGAATCAAGAGGCTTTTGTAAATCTGGGCGTTGTACTGAACAACGCTATAACTGGTCAAGTGTCTGAAAAGACCAGGTTTGGTTTCTTTGCACGAAATGGGAAATATGTGGAGTGCCTACTTTCTGTGAGCAAAAGATTGGACCAAGAGGGAGCCGTCACGGGGCTATTTTGTTTCCTGCAGTTAGCAAGCCAGGAACTGCAACAGGCCCTTCATTTTCAAAAATTGTCCGAGCAAACTGCAATGAAGAGGTTGAAAGTGTTGGCGTACATAAGAAGGCAGGTCAAAAAACCCTCTTTCGGGAATCATGTTCTCTCGAAAGATGCTAGAGGGGACGGAGTTGGGTATCTGGATCTGGAAATGGTTGAATTTAAGCTAGATGAAGTGTTACAAGCTTCAATTAGTCAAGTAATGACAAAGAGCAATGGAAAGAGTTTAAGGATAATTAATGACATAGCTGACAATATTCTTTGTGAAACTTTATATGGAGATAGTCTGAGGCTTCAACAAATCCTCTCTGAATTTTTGTCAGTTGCTGTGAATTTTACCCCGGGCGGAGGCCAGCTTGCTCTTTCATCCAAGTTGACTAAAGATAATTTGGGAGAATCTATTCAGCTTGCCCATTTGGAATTCAGGCTAACGCATACAGGCGGTGGGGTGCCAGAAGAGCTGCTGACCCAAATGTTTGGTAGCGAAGCAGACGCATCAGAGGACGGGATCAGCCTGCTTATAAGCAGAAAGCTGGTGAAGCTCATGAACGGGGACGTTCAGTACCTCAGGGAGGCGGGTCGATCCACCTTCATCATATCTGTCGAACTTGCAGTTGCTTCTAAACCTTCTTCGTGA

>IbHKL9

ATGGCGTCAGGGAGCAGGTCCAAAAACGTGCAGCAAAATCAAGCTCAATCTTCGGGTACAAGTAATGTTAATTATCGCGATTCAGTGAGCAAAGCTGTTGCGCAGTACACTGTGGATGCTAGGTTGCACGCTGTTTTCGAGCAGTCGGGAGAGTCTGGCAAGTCTTTCGATTACTCCCAATCTGTGAAGACTATTACTCAGAACGTTCCCGAGAAGCAAATCACCGCCTACTTGTCCAAAATTCAGAGAGGGGGTCACATTCAACCCTTTGGGTGTATGATTGCGGTGGACGAGCCCAGTTTTCGGGTAATTGGCTATAGCGAGAATGCCCGCGAAATGCTTGGTTTAACGCCCCAGTCAGTTCCCAGCCTCGAGAGGCCTGAAATCCTCGCGATTGGGACGGATGTGAGGACCCTCTTTACGCCCTCCAGCTCCGTTTTGCTTGAACGCGCCTTTGGAGCGCGAGAGATCACCTTGCTCAACCCAATTTGGATTCATTCTAAGAATTCCGGCAAGCCCTTTTACGCCATTTTGCATAGGATTGATGTTGGAATTGTGATTGACTTGGAGCCTGCTAGGACTGAGGACCCTGCATTGTCTATTGCTGGCGCTGTGCAGTCTCAGAAGCTCGCTGTGAGGGCAATTTCCCACTTGCAGTCACTTCCCGGCGGGGATATTAAGCTTTTGTGTGATACTGTGGTTGAGAGTGTGAGGGAGCTAACCGGGTATGATCGAGTCATGGTGTATAAGTTTCATGAGGATGAGCATGGGGAGGTTGTGGCCGAGAGCAAAAGGCCTGATTTAGAGCCCTATATTGGATTACACTATCCTGCCACTGATATTCCTCAAGCTTCCAGGTTTTTGTTTAAACAGAATAGGGTTAGGATGATAGTTGATTGCAATGCCACTCCGGTGCAGGTTATTCAGGATGAATCACTGATGCAACCACTGTGTTTAGTTGGATCAACCCTTCGTGCTCCTCATGGCTGCCATGCACAGTACATGGCCAATATGGGCTCCATTGCTTCGTTAACACTCGCAGTTGTTATTAACGGTAGTGATGAGGAAGCTGTAGGAGGGAGAAACTCAATGAGGCTATGGGGGTTGGTTGTTGGGCATCACACTTCTGCCAGGTGCATCCCTTTCCCCCTCCGTTATGCTTGTGAATTTCTTATGCAGGCGTTTGGTCTGCAGTTGAACATGGAACTGCAATTGGCATCACAATTGTCTGAGAAGCATGTCCTAAGGACGCAAACGCTTTTGTGTGACATGCTTTTGCGAGATGCCCCCATTGGCATTATTACCCAGAGCCCCAGCATTATGGATCTTGTCAAATGTGATGGAGCTGCACTATACTACCAAGGGAAATATTATCCTTTAGGTGTGACACCTAATGAAGCCCAGATAAAGGAAATAGTTGACTGGCTATTGACTTACCATGGTGACTCAACTGGGTTGAGCACAGATAGTTTAGGTGATGCAGGGTATCCTGGTGCAGCTTCACTCGGTGATGCAGTTTGTGGGATGGCAGTTGCTTATATAACTTCAAGAGATTTCTTGTTCTGGTTCCGGTCTCACACTGCAAAAGAGATTAAGTGGGGTGGTGCTAAGCATCATCCAGAGGACAAAGATGATGGTCAGAGGATGCATCCGCGCTCTTCGTTCAAGGCATTTTTGGAAGTTGTTAAGAGTCGCAGTTTGCTTTGGGAGAATGCTGAAATGGATGCAATTCACTCTTTGCAGCTTATCCTACGTGATTCATTTAAGGATGCTGAGGCAAGCAATTCTAAGGCTGTTGTGCGTGCTCCGCCTGGAGGATTGGAGTTGCAAGGAATGGATGAACTGAGTTCTGTTGCTAGAGAAATGGTTAGATTGATAGAGACTGCTACAGCTCCCATATTTGCTGTAGATGTTGAAGGGCGCATAAATGGGTGGAATGCAAAAGTTGCTGAGTTGGTAGGCTTGTCGGTTGAAGAAGCTATGGGAAAGTTGTTGATTCAAGATTTAGTTCACAAGGAATCACAAGAAACTACCGAGAAGCTTCTGTTTAATGCTTTAAGAGGTGAAGAAGATAAGAACGTAGAGATAAAGTTGAGAACATTTGGCACTGAGGAAGATAAGAAGGCTATTTTTTTAGTAGTCAATGCATGTTCTAGCAAGGACTATACAAATAATATTGTTGGTGTCTGCTTTGTTGGTCAAGATGTTACAGGACAGAAAATTGTAATGGACAAATTTATTCACATACAAGGTGATTACAAGGCTATTGTACACAGTCCCAATCCTCTGATCCCTCCCATATTTGCTTCAGATGAGAACACATCCTGCTCTGAGTGGAATACTGCCATGGAAAAGCTTACTGGGTGGAGCAGGGGTGAAACGATTGGCAAGTTGCTAGTTGGTGAGGTTTTTGGAAGCTGCTGCAGGCTCAGGGGTCCAGATGCCATGACTAAATTTATGATCATATTGCATAATGCAATTGGAGGCCAAGACACAGACAGGTTTCCATTTTCATTTTTCGACCGAAATGGAAAGTACGTGCAAGCTCTCTTGACGGCAAATAAGAGAGCAAATATGGATGGACAGATTATTGGAGCCTTCTGCTTCTTGCAGATTGCCAGTCCTGAATTGCAGCAAGCTCTCAAAATCCAGAGGCAGCAGGAAAATAAATGCTTTTCGAGAATGAAAGAGTTGGCTTACATCTGTCAAGAAATTAAAAATCCACTGAATGGCATACGCTTTACAAATTCATTATTGGAGGCAACAGATTTGACAGAAGACCAGAAGCAGTTCCTGGAGACTAGTGCTGCTTGCGAGAAACAAATGTCAAAGATTATAATGGATGTTGATCTGGAAAACATTGAAGATGGTTCACTTGAGCTGGAGAAAGAAGATTTTTTTCTTGGGAGGATAATAGATGCTATTGTTAGCCAAGTAATGTCGTTGCTGAGAGAAAGAGGTCTCCAACTTATCCGGGATATTCCAGAAGAAATTAAGACACTGGCTGTGAATGGCGATCAAGTGAGAATTCAGCAGGTGCTGGCAGATTTTTTGCTAAACATGGCACGCCATGCACCAGTTCCAGGAGGATGGGTAGAAATCCAAGTTCGCCCTAGTTTGAAGCAAGTTTCTGATGGCACAAATGTTGTGCATACTGAATTCAGGATAATGTGCCCGGGCGAAGGTCTTCCTCCTGAATTGGTGCAAGACATGTTCCACAGCAGTCGATGGGTGAGTCAAGAAGGACTAGGGCTGAGCATGTGCAGGAAAGTAGTAAAGCTTATGAACGGGGAAGTCCAATATATCAGAGAATCAGAAAGATGTTACTTCCTGATCATCCTTGAGCTACCAATCCCCCGAAGAGGTTCAAAGAGTATTATTATTGGCTAG

>IbHKL10

ATGGCGGGCTCAGGGACAGGGAGTAGTAGTAAGAGGTTTATTGAGCACCAGAGTTCTTCAGCTCAAATTGCTCAATCTTCAGGTACGAGTAACTCTAATAATCGCTATCCTGTAAGCAAGGCGGTAGCGCAGTACACTGAGGATGCTAGGCTCCACGCGGTGTTTGAGCGGTCCGGCGGGTCGGGGAAGTCGTTTGATTACTCTGAATCAGTGAAGGTCGCGACTCATTTTGTAGCGGAGCAGCAAATCGCCGCGTACCTGTCCAACATTCAGAGGGGAGGTCACATACAGCCTTTTGGGTGTATGATTGGCGTGGAGGAGGGGAGTTTTCGCGTGATTGCGTATAGCGAGAATGCACGCGAGGTGCTTGGTTTAATGCCTCAGTCAGTTCCGAGTCTGGATCGGCCGGATATCCTGGGGATTGGGGTGGATGTGAGGACGCTTTTCAGGCCTTCGAGCTCGGTGTTGCTCCAACGGGCGTTTGGAGCGCAGGAGATCACGTTGCTGAACCCTATTTGGGTTCACTCCAAGAATTCCGGTAAGCCTTTTTACGCGATTTTACATAAGATTGATGTTGGCATTGTGATTGACTTGGAGCCTGCTAGGAGTGAGGACCCTGCCCTGTCCATAGCCGGGGCTGTGCAGTCACAGAAGCTCGCCGTGAGGGGCATTTCGCGCTTGCAGTCGCTTCCCGGTGGGAATATTAAGCATCTTTGTGATGTTGTGGTTGAGTGTGTGAGGGAGTTAACCGGGTATGATCGAGTTATGGTGTATAAGTTTCATGAGGATGAGCATGGGGAGGTTTTGGCTGAGAGCAAAAGACCAGATTTAGAGCCTTATATTGGGCTGCACTATCCAGCTACTGATATTCCTCAAGCCTCCAGGTTTTTGTTTAAACAAAACAGGGTTAGGATGATTGTTGATTGCAATGCCACCCCGGTACGCGTTATTCAGGATGAATCACTAAAGCAGCCGTTGTGTTTAGTTGGTTCGACTCTACGGGCTCCTCATGGTTGCCATGCCCAGTACATGGCCAATATGGGCTCCATTGCCTCGTTAACTCTCGCAGTTATTGTAAATGGGAACGAAGATGAAGGTGTTGGAGGGAGGAATTCGATGAGGCTATGGGGGTTGGTTGTAGGCCATCACACTTCGGCTAGGAGTATTGCGTTCCCCCTTCGTTCTGCCTGTGAGTTTCTTATGCAGGCCTTTGGGCTCCAGTTGAATATGGAATTGCAATTGGCATCACAATTGGCAGAAAAACATGTGTTAAGGACACAAACACTGTTGTGTGACATGCTTCTAAGGGACTCCGCCACTGGGATTGTTACCCAGAGCCCTAGTATAAGGGATCTTGTGAAATGTGATGGGGCTGCATTGTACTATAAGGGTAAATACTATCCTTTAGGCGTGACACCTACTGAAGACCAGATAAAGGATATAGCTGTGTGGTTATTGACTTACCATGGAGACTCGACAGGTTTGAGCACTGATAATTTGGCTGATGCAGGGTACTCTGGTGCAGCTTCACTTGGTGATGCAGTTCGTGGGATGGCTGTTGCCTATATAACACCAAAAGATTTCTTGTTCTGGTTTCGGTCCCACACTGCAAAAGAGATTAAGTGGGGTGGTGCTAAGCATCATCCACAGGATAAAGATGATGGACAAAGGATGCACCCTCGATCTTCATTCAAGGCATTTTTAGAAGTAGTTAAGAGACGTAGTTTGCCATGGGAGAACGCAGAAATGGATGCAATTCACTCTTTGCAGCTTATTCTACGCGATTCTTTTAAGGATGCTGAGGTAAGCAATTCTAAGGCTGTTGTGCATGCTCCGCCAGGAGAGTTGGAATTGCAAGGAATGGATGAGCTGAGCTCTGTTGCCAGAGAAATGGTTAGATTGATAGAAACTGCAACCACTCCGATATTTGCTGTAGATGCTGAAGGACACATAAATGGATGGAATGCGAAAGTTGCTGAGTTGGTGGGGTTGCCAGTTGAAGAAGCAATGGGAAAGTCATTAGTTCATGATCTTGTTCATATGGAATCACAAGAAACCACTGAGAAGCTTTTGTTTAATGCTTTAAGAGGTTCTGAAGACAGGAATGTAGAGATCAAGTTGAAAACATTTGGCACTAAGCAACATACAAAAGCCGTTTTTGTGGTGGTCAATGCTTGCTCTAGCAAAGACTGTACAAATAAAATTGTTGGTGTATGTTTTGTTGGTCAAGATGTGACGGAACAGAAAGTTGTAATGGACAAATTTATTCACATACAAAGTGATTACAAGGCAATTGTACATAGCCCCAATCCTCTGATCCCTCCCATATTTGCTTCAGATGAGAACGCTTGTTGCTCTGAGTGGAACATCGCCATGGAAAAGCTTACTGGGTGGAGCAAAGGGGAAATGATGGGGAAGATGTTAATTGGCGAGCTTTTTGGAGGAGTCTGTCGACTCAAGGGTCCAGATGCTATGATGAAATTCATGATCACATTGCATCATGCGATTGGAGGCAAAGATACAGACAAGTTTCCCTTCTACTTTTTTGACCGAAATGGAAAATATGTGCAAACCCTCTTGACCGCAAATAAGAGAGTGAATATGGATGGTCGGGTTATTGGAGCCTTCTGTTTCTTGCAGATAGCAAGTCCTGAATTGCTGCAAGCCATCAAAATCCAGAGGCAACAAGAAAACAAGTGGCTCACTAAGTCAAAAGTGATGGCATATATCTGCCAGGAAATTAAGAATCCACTGAATGGTATACGCTTTACAAGTTCTTTATTAGAGGCAACAAATTTGACAGAACATCAAAAGCAGTTTCTGGAGACTAGCGCCGCTTGTCAGAAGCAGATGTCGAAGATTTTAAGGGATGCTGGTCTGGAAAACATTGAAGATGGTTCACTGGAGCTAGAGAAAGAAGAATTTCATTTTGGGAGTGTTATAGATGCTATTGTCAGCCAAGTAATGCTATTGCTGAGAGAAAGAGGTCTGCAATTTATGCTGGACATTCCAGACGAAATGAAGACGCTGAAAGTGTATGGTGATCAAGCAAGAATTCAACAGGTGCTGGCAGATTTTTTGCTGAACGTGGTACATCATGCGCCAACTCCAAAAGGATGGGTAAAAATCCATGTTCGGCCTAGTTTGAGGCAAAGTTCTGATGGAATAACCATAGCGCACGTTGAATTCAGGTTTATCTGCCCCGGTGAAGGTCTTCCTTCAGCGCTGGTCCAGGACGTGTTCAACAACAGTGAATGGGAAACTCGGGAAGGATTAGGGCTGAGTATGTGCAGGAAAATAGTGACACTCATGAATGGAGAAGTGCGATATGTAAGAGAAGCAGAAAGGTGTTATTTCCTAGTTATCCTCAAGCTGCCCGTGCCCACAAGAGGCTCAAAAAGCGGTTGA

>IbHKL8

ATGGATTTGCAAAGCCAGGAAAACAAACCACCCACAAGCAAGAAAATGGAGAATCATGCCAAAGCTGCGACCTTTTCTTCGTCTGCTACTAGCAACTTGAACACCGGCAAGGCCATAGCTCAGTACAATGCTGATGCGAAGCTAATGGCTGAGTTTGAGCAGTCTAGGGAGTCTGGTAAGTCCTTTGACTACTCCAGGTCTGTTATTGGTGCTCCACAGAATGTGACTGAAGAAGAAATGACTGCTTATTTATCAAGAATCCAGAGGGGTGGGCTTATCCAACCCTTTGGTTGTATGCTTGCAATTGAAGAACCCAGTTTCAAGATTGTAGGGTTTAGTGAGAATTGCTTTGATTTGTTGGGTTTGAAGAGTGGTGTTGAGCCTCCGGAGAGGATGAGTTTGATTGGGATTGATGCCAGGACTCTTTTCACCCTTTCTTCAAGGGCTTCTTTAGCCAAGGCTGTGGCATCTAGGGAAATTTCTCTTTTGAACCCAATTTGGGTGCATTCTAAGACTAATCAAAAGCCTTTTTATGCTGTACTCCATAGAATTGATGTAGGGATTGTGATTGATTTGGAGCCTGCTAACTCTGCTGATCCTGCACTGTTGCTTGCTGGGGCAGTGCAATCACAGAAACTCGCGGTCCGGGCTATTTCTAGGCTTCAGGCACTCCCTGGGGGAGATATAGGGACATTGTGTGACACAGTAGTGGAGGATGTACAGAAGCTGACCGGGTACGATAGGGTAATGGTTTATAAGTTCCATGATGACAGTCATGGCGAGGTTGTGTCCGAAATTAGGAGGTCAGACTTAGAGCCTTATTTAGGATTGCACTATCCTGCAACAGATATCCCACAAGCGGCGCGTTTCTTGTTTAAACAGAACAGGGTTAGGATGATCTGTGATTGCAATGCACAACCCGTCAAGGTTTTTCAAAGCGAAGAACTAAAACAGCCTCTTTGCTTGGTGAATTCGACTCTTAGATCACCTCATGGCTGCCATACCAAGTATATGGCTAACATGGGGTCTATAGCCTCGTTGGTGATGGCTGTTGTTATTAATAGTAGTGAGTCCATGAAGCTTTGGGGATTGGTAGTGTGCCACCATACTTCTGCTCGCTATGTTCCTTTCCCTCTTCGCTATGCATGTGAGTTCCTTATGCAGGCGTTTAGTCTTCAGCTTTATATGGAGCTTCAATTGGCCTCACAATTGGCTGAAAAGAAAATTCTCCGCACTCAGACCTTACTATGTGACATGCTTCTCCGAGATGCTCCGTTTGGAATTGTGACGCAAACTCCTAGTATAATGGATCTCGTGAGGTGTGATGGGGCTGCACTCTATTATGACGGGAAATGTTGGTTGCTTGGTGTAACACCAACTGAGACACAAGTTAAAGATATTGCAGAGTGGTTGCTACATAATCACGGGGATTCTACAGGTTTGAGTACGGATAGTCTTTCGGATGCTGGCTATCCCGGAGCACCCTTATTAGGTGATGCAGTTTCTGGCATGGCTACTGCAAGAATCACATCCAAGGATTTTCTATTCTGGTTTAGGTCTCACACTGCAAAGGAAGTTAAATGGGGAGGGGCTAAGCATCATCCCGAGGATAAAGATGATGGTGGAAGAATGCACCCCCGATCTTCATTCATTGCCTTTCTAGAAGTGGTGAAAAGCAAAAGTCTGCCTTGGGAGGATTCAGAAATTAATGCTATTCATTCTTTACAGCTCATAATGAGAGATTCTCTTCAAGGGATTGGAGAGAACTATATGAAAAGCGTGTCATCACCCCAACAGACTGATTCCGAAGGGACAAGGTTCTATGAACTTAGTTCAATGGCGTTGGAACTGGTCAGGTTGGTCGAGACAGCAACAGTTCCTATTTTTGGTGTTGATTCATCTGGCTTAATCAATGGATGGAATGCAAAGATTGCAGAACTAACAGGATTGCAAGCTAATGTTGCGATTGGAAAGTATCTCATTGATTACGTTACTCATGAAGATTCACATGAAACTTTCAAAGGTCTTATGTGTCGAGCTTTACAAGGTGAGGAGGACAGAAATGTAGAAGTAAAACTGCTAAAGTTTGGGGAGCATCCAACAAAGGAAGTTGTATACCTTGTCGTTAATGCCTGCACAAGTAGGGACTACAAAAACGATATTATTGGGGTGTGCTTTGTGGGTCAAGACATCACTCCTCAGAAAGCTGTGATGGATAAATTTGTTCGGTTGCAGGGAGATTATGAGGCTATTATACAAAGCCTTAATCCACTAATCCCGCCAATATTTGCTTCTGATGAGAATGCCTGCTGCTCTGAATGGAACGCGGCTATGGAGCGGTTAACTGGTTTGGTGAAATGTGAGGTTATAGGGAAGAGACTTCCGGGTGAAATTTTCGGTGGCTTGTGTCGGCTTAAGGGTCAAGACGCTCTTACCAAATTTATGATTCTCTTGTACCAAGGGATAAGTGGTCATGATACCGAGAAGCTCTCATTTGGATTTTTTGATAGGAAGGGGAACTTTATAGACGTGTTTATAACCGCAAATAAGAGAACTGATGAGCGTGGGAATATAATTGGATGTTTCTGCTTCTTGCAAACGATGGCTGTTGACCCGCAGACATCCGCAAGAGATATAGAAGATGATAGAGAATGTCTCTCGACTCTTAAAGAGTTTGCTTACATCCAACAGCAGATGAAAAATCCCTTGAATGGAATTCGTTTCACCCACAAGCTCCTTGAAGGTACCGTTACTTCAGATCATCAGAAACAGTTTCTCGAGACAAGTGAAGCCTGTGAGAAACAGATACTCTCTATAATCGAGAATATGGATAGTGGAGGCATCGTGGATGGCAACAAAGTGGAGCTAAAGACGGAAGAGTTTGTTATAGGAAATGTCATAGATGCAGTGGTTAGTCAAGTTATGATCCCGCTGAAGGAGAAGAATTTACAGCTACTTCATGATATTCCGGACCAGATCAAATCTCTCCCTATATACGGGGATCAAATTAAGCTTCAGCTTGTCCTATCGGATTTCTTGCTCAGCATAGTGCGCCACGCACCTTCTCCAGATGGTTGGGTGGAAATCAGGGTATCCCCGGGGTTGAAGCTGATTCAAGACGGGAATGAGTTTATCCATATCCAGTTCAGAATGACTCATCCCGGGCAAGGCCTCCCTTTTGCTCTTATCGAAGACATGGTAAGAGGAGGAACTCGATGGACAACACAAGAAGGAATTGTTTTACATTTGTCACAGAAACTCGTTAGAATGATGAATGGTCATGTCCATTACGTCAGAGAACAACAAAAGTGCTACTTCCTAATCGACCTTGATTTCAAAACACAAAAACCAAGGTCGCGGGAGTCAAGTATGGATACGAGCAGAATAACCTGA

>IbHKL7

ATGTCGTCTAGATCGGGTACAATCAGGACAAATTGCTCCATGAGCAGCTCTGCTCGGTCGAGGCATGATGCTCGCGTTGTTGCTCAGACATCTATTGATGCTAAGCTCCATGTGGAGTTTGAGGAGTCTGAGGAACAGTTTGATTATTCTACCTCTGTTAATTTGTCCAATTCAACTAGCAATATCCCCCTCTTCAACTGTGTCTGCTTATCTCCAGAAGATGCAAAGAGGAATGAAAATGCACCAGAAATGCTGGATTTGGCACCACATGCAGTTCCAAGCATTGAACAGCAGGAAGCTCTGACTTTTGGCACCAATGTTAGGACACTGTTTCGGTCAACTGGTGCTGCTGCACTTGAAAAAGCAGCAAGTTTTGAGGAAGTTAGTTTGATTAATCCTATTCTGGTTCACTGCAAAAATTCGGGTAAGCCTTTTTATGCAATTTTACACCGAATTGATGTTGGATTAGTTATAGATTTGGAGCCTGTCAATCCAGCAGATGTTCCAGTGACAGCTGCTGGAGCATTGAAATCATATAAACTAGCAGCTAAAGCCATTTCGAAGTTGCAATCACTGCCAAGTGGAGATATATCATTGTTGTGTGATGTGTTAGTTAGAGAAGTAAGGGATTTGACAGGTTACGACCGAGTTATGGTTTATAAATTCCATGAGGATGAGCATGGGGAAGTTGTTGCAGAATGCCGCAAGCCTGATCTTGAACCTTATCTTGGCTTGCATTACCCTGCTACTGATATACCACAAGCTTCAAGATTTCTTTTCATGAAAAACAAGGTCAGAATGATATGTGATTGCTTAGCTCCATCGGTAAAAGTAATCCAAGACAAGACATTGGCTCAACCATTAAGCCTTTGTGGATCCACATTAAGAGCTCCCCATGGATGTCATGCACAATACATGGCCAATATGGGGTCCATTGCATCTTTGGCAATGTCTGTGACAATCAATGAGGATGATGATGAGATGGATAGTGATCAACAAAAAGGAAGAAAACTCTGGGGATTGGTGATCAATAAGGAAGTGGAGTTGGCAGCTCAACGTCTAGAAAAGCATATATTGCGAACCCAGACTGTACTCTGTGACATGCTTCTCAGAGAATCTCCTGTGGGTATTGTCACCAAGTCTCCTAATATTATGGACCTTGTCAGATGTGATGGAGCTGCACTTTACTATAGGAACAAATTTTGGTTGCTTGGTGCAACGCCAACAGAGCCCCAAATTAGAGATATAGCACAATGGCTTCTTGATTCTCATAGTAGTAGTACAGGGTTAAGCACTGATAGCCTCATGGAAGCTGGCTACCCAAATGCTTCCGTTCTTGGTGATTCAGTCTGTGGAATGGCTGCTGTCAAAATAACTGCAAAAGATTTTCTCTTCTGGTTTCGATCTCACACAGCAAAAGCGATTAAGTGGGGTGGTGCAAAACATGATCCTGGAGACAAGGATGATGGGAGAAAGATGCATCCAAGATCATCTTTCAAGGCTTTCCTGGAGGTGGTTAAGAGGAGTCTGCCTTGGGAAGATGTGGAGATGGATGCAATTCATTCCTTGCAGCTGATACTGAGAGGCTCTTTGCAAGACGAAGTTGTTGATAATTCTAAAATGATTGTGAATGTACCTGCTGTGGACACCAGTATACAGAGGGTTGATGAACTTCGAATTGTGACAACTGAAATGGTTCGCCTTATTGAGACAGCATCTATACCCATTTTGGCTGTTGACACTTCTGGCTGTATCAATGGGTGGAACATTAAAGTGGCTGAGCTAACCGGATTGGTTGTACAAGAAGCTATAGGTGCGCCCTTAGTTGATTTGGTTGTCAGTGAAGCTGTCAGCACCATTAAAAATGTGCTCTCCCTCGCTTTGCAAGGCAAAGAGGAGAAAAATGTTGAAATCAAACTTAAAAAGTTTGGTTCTCCAGAAAACAATGATCCTGTTATTCTGGTAGCTAATGCCTGTTCAAGTGATTATGTTGGAATTTTGCGCAGCCCTTCCGCATTGATTCCTCCAATCTTTTTGATGGATGAGCATGGTAGATGCTTGGAATGGAATGATGCAATGCAAAAGTTGACTGGTCTGAAGAGGGCAGAGGCCATTGATCAAATGATTCTCGGTGAGGACAGGATGCAGAGGATTTGTTATTTGGTTTTTTTTGATAAGCAGAATAAATATGTTGAAGCATTAATATCAGCAAATAAAAGAACTGATGTAGTGGGGCGGATCACTGGGGTCCTCTGCTTTTTACACGTTCCTAGTCCAGAACTTCAGTACGCAATACATGTACAAAAACTATCTGAACAAGCAGCTGCAAATAGCCTTAAAAAGTTGGCTTATGTTCGTCGAGAAGTTAGAAACCCGTTAAATGGTATAAAGTGCATTCAGAATCTGATGAAATCTTCTGACCTAAGTAAGGATCAGATGCAGCTTCTGAAGACCAGTACAATGTGCCAAGAACAGCTGGCTAAGATTATTGATGATACCGATATTGAAAGTATTGAAGAAAGCTATATGGAAATGAACTGTTGCGAGTTCAGTCTTGGTGAGGCTATTAAAGCAGTCGTAAATCAAGCTATGATTCCAAGTCGGGAGCGCCAGGTGCAGATCATGTGCGATTTACCTGTTGAAGCATCATCCTTGTACTTGTTTGGAGACAATTTGAGGATTCAACAAGTGCTCTCAGACTTCTTGACAACTGCTGTACTCTTTACACCTCATTTTGAAGAATCATCCGTTCTATTCAGAATTATTCCTAGAAGGGAGCAGATTGGAGCCAAGATGCATGTAGTGCATCTTGAATTTCGGATCACACATCCAGCCCCGGGGATTCCAGAAGAGCTAATCCAGGAAATGTTCAACTATAGCCAAAGCATGTCGAGGGAAGTATCTTCGAGAGGCAGAGAGGTCATCATTCATAATCTTAGTAGAATTTCCAGCATCTCTACGAAGCGACCATCAATAACTTCAATCATCAGTGTGCTCACCGCCTGCTTGGGGAGAAACTGCACCTTAATTATGGCAGACGAGTACTCAAACATCATGGCTTGCCCGATCTGCCTCGTCCTCTTCAGCAACAACCATAATAAAGAATGA

>IbCKI1

ATGGTCAACTTTGTTCGCTCACTCAAGACTATGTGGCCTGTCTACTTGGCCCTCACTCTCGTAGGTTGGCTGATTTCGGTGTTGGTTGCGATTCAACACCATACTACCCAGGGGGGAGCGAAAGTTGAGAAGAGGGATGTGAAACTGGGGATGATTCTCCTGGGCATAAACATTTGTATTACAGTGACGAGTGTGGTGGTGTTGACATGGTGGAGGAGTAGAGTGATGATGAGGGAGATGTGTGTAAAGGCGGCGCTGATAAAGCAGAAGGAAGCCACAGAAGAAGCGGAGAGGAAGAGCATGAGCAAGAGCGTGGCGGTGGCCAATGCGAGCCATGAGGTGCGGACTGCTCTTGCAGGGATTACTGGTTTGATTCAGATGTGCCGCGCTGATGCTGATGCTTCTGCTGCTCATTCTGAACTCAACGATAATCTGAGGCACATGGAATCATGCACCAATGATCTCTATAGTTTATTGAATTCGATTCTGGATGCTAGCAGAATAGAAGCGGGTAAAATGCAGGTTGAAGAAGATGAATTTGATTTGCAGGAACTGCTAGAAGATGTGGTGGATTTGTACTATCCTGTAGGTATGAAGAAGGGAGTGGATGTAATATTGGATCCGTGTGATGAGTCCGTGGAAAAGTTTAGGCGTGTGAGGGGGGATAGAGGCAAACTCAAACAAGTTTTGTCTAATTTGTTGTTCAACGCCATTAAGTTCACAGATGAAGGCTATGTTGCTCTTCGTGTTTGGGCACGAAAACCTTCTCCTTGTCCTCCTTCTCAATCTCCTCCTAAACCCAAACGACCTTCTTCTTCTTCTTCTCCTATCGCCATTTTAAAGGGCTGTGTAGCAACCTTTTGCAGTGTTCCTGCAAAAACAGGAGGTGGGGAAGAAGAAGTAAATGATTCGGTGTTGGAGAGAAAAGATGGTGGGATAGAGTACATATTTAAGGTGGTGGATACAGGGAAAGGGATTCCCAAGGAGAAAAGGAATAGTGTGTTTGAAAACTATTCTCAGGTGAAAGATATGGGTAGGGGAAAGAAACATCAATTAGGGCATGGATTAGGGCTTGGGATAGCTCAGTCTCTAGTGCGGTTAATGGGCGGGGAGATTGGGATTGAGGATAAGGAAACCGGGGAAAGGGGGACTTGTTTTAAGTTCAACATAGTCTTGGACAACATTGTTATTCTAGAATCATCATCATCGTCTCATAATAACAACATTAATACTTATTCATCGGGTCATCACGTTGTTGTGTTCATGCATTGCGAGGAGAGGGGTAAAATCATAGGGAGATTCTTGGAAAACCGTGGCATTAAGGTTAGTTTGGTTCAGAAAGGACACCAGCAACTGAGCAGGAAATTGAAGAAGATCAAACGTGGGGCATTAAATCTCCCCCGTTCCACAACCACACCCTTACCCTCTTACTATTCTTCTTCTTCGTCCTCAAAGGAGGAGTTGGAAGACGAAACCATGCCTCTCCATACCAACACTTGTACGGTGTTGATAATAATAGACACAAGTGCAGCAGGAGAAGCATTATTCCCAGAAGTGATTAAAGCAGTTAGTGAATTCCATAGGGACCTTCAACCTGGTTGTGTTAGGGTTCTTTGGATAGACACTACTGCTTTAGGTAGAGGTGTGGATAACAACTTTCAGCTCCCTTCAACTGATCTCATCGTGTCCAAGCCCTTGCAAGGCTCCCGTCTGCATAGCGTGTTAGGGCTTCTACCCGACTTTGCATCAAGTTCCCAACTGGGAGAAATACAGGTGGTGATTGAGAAAGATAAGGAAGAAGAAGACGAGGATAATGGTGGTGGGAGCAGCAGTAGTGAAAAGAAGGCATTGACGGGGAAAAGAATCTTGGTGGTTGAAGACAATCCAACGCTGCGCAAAATATGCACCACAGTGGTTTCAAGTCTGGGTGCTCTAACTTACGCCTGTACTAATGGTGAAGAAGCTTTACAGCTCGTGTCTTCGGGTCTTCAGGACCACCATCATCAACCTCCCTTTGATTACATTCTAATGGACTGTGAGATGCCGATAATGGATGGGTTTGAAGCCACAAAGCGCATAAAAGAAGAAGGGAAAGCTATGGGGATATGGATTCCCATTATTGCTCTAACAGCACATACAGGAAAGGAGGATATGGACAAGGTGACTGAAGCTGGAATGGATTACTACTTGTCCAAGCCCATCAATGCTGCTACTCTTCTAACAGCTATTCACTTCTTGGACAAATCCACCACCCATCTCTAA

>IbHP2

ATGGACGTTGTGCCTCAGCTGCAGAAACAATTTGTGGACCTCATAGCTTCTCTATATCGAGAGGGCTTTTTGGATGATCAGTTTCTGCAGCTTCAGAAACTGCAAGATGACAGCAACCCAGACTTTGTGTTTGAGGTTGTTTCACTTTTCTTTGAGGATTCTGAGAAGCTTATTAACAATCTGGCCACAGCTCTTCAGCAGCCAGTTGTAGATTTTAACCAGGTTGATGCCCATGTTCACCAGTTCAAGGGAAGCAGTTCCAGCATAGGTGCACAAAGAGTAAAGAATGCATGTGTTTCTTTCAGAAATTTTTGTGAGGAGAAGAACCTCGATGGGTGTGTGCAATGCCTGCAACTCGTGAAAAATGAATACTTTGTTGTGAAGAACAAACTCGAAACTTTACTCAGACTCGAGCAGCAAATCTTGGCCGCTGGTGGGAAAATTCCTGTTCTGCCATAA

>IbHP1

ATGGAGGTGAGCCAATTGCAGAACAGTTTTCTTGGGTACATGGCAGAATTATCTCGTGAGGGATTCTTGGATGCTCAGTTTAGTCAGCTCCAGCAACTTCAGGATGAAAGCAACCCTACTTTTGTAGCTGAAGTTGTGACTCTCTTCTTTGAAGATTCTGAAAGACTACTCAATGATCTCAACACAACTCTTAATCAGCCAGATGTGGACTTTAAGAAGGTTGATGCTCATGTTCACCAGCTCAAAGGTAGCAGCTCAAGCATAGGTGCTCAGAGAGTGAAGAATGTCTGCGTTGCTTTTCGCAACTTCTGTGAAGAACACAACATTGAAGGGAGCTTGAGATGCTTGCAACAAGTAAAACAAGAGTACTTGCTAGTCAAGAACAAGCTTGAAACTTTATTCAGGGGGGAGGTAAAGCCCCTTACTGCTTATACCTTGGAGACGGGTGGGGGAGATAAGTCACGGGATATGTCTCAAGTGACCTACAAGCTCCTAAATGCTTGTACACTAGTGCCCACAACAAAGTGA

>IbHP3

ATGGAAGTTGTAGGACAGCTGCAGAAACAGTTTGTAGCGTACATGGCTTCCCTGTATCGTGAGGGCTTTTTGGATGATCAGTTTCTGCAGCTTCAGAAACTGCAAGATCAGAGCAACCCAGATTTTGTGGTTGAAGTGGTTTCTCTTTTCTTTGAGGATTCTGAGAAGCTTATCAATAATATGGCCAATGCCTTTCAGCAACAAGTTGTAGATTTTAAGCAGGTTGATGCCCATGTCCATCAGCTCAAAGGTAGCAGTTCCAGCATAGGTGCACAAAGAGTAAAGAACGCTTGTGTTTCTTTCAGAAACCATTGCGAGGAGAGGAGTCTTGATGGGTGA

>IbHP5

ATGGAAAGAAACCACTTGCCTAGGCAGCTTGCCACCATGAGGAAGTCTCTTTTTGATCAGGGGTATCTAGATGATCAATTTGTTCAGTTGGAAGAGCTGCAAGATGATGTTAACCCTAACTTTGCAGAGGAAGTTGTCACTTTATTCTATCGAGATTCTGCTCGCTTAGTGCAAAACATTGAACATGCATTGGAGAGAAGTCCTCTTGACTTTGCTAAGCTGGATGGGTTGATGCACCAGTTCAAGGACAGTTGCTCAAGCATTGGAGCCAGGAAGGTGAAATATGAATGCACGCAGTTCAGGGAGCATTGCAGGGTGGCAAATGCAGAGGGATGCAAGAGAAGTTTCCTGCAGCTAAAGAAAGAATATTCCACACTCCAAAAGAAGCTCAAAGCTTATTTTCAGTTTGCAAGACAAGCTGGGCCTGTTGAGGTGGCATGTCGCCCTAACTGA

>IbHP4

ATGGGATACATTGACGATCAATTCATTCAGCTTGAAGAATTGCAAGATGATGCTAACCCTAATTTTGTTGAGGAAGTTGTCAGATTGTTCTACAACGATTCAACTAGGCAGATTCACAACATAGAATTGGCACTGGGAAGTGGAGCTTGTGATTTTACTAAGCTTGATGATATGATGCATCAGTTCAAGGGAAGCTGCTCAAGTATTGGTGCCAGAAAGGTGAAGAAGGAATGCTCAGAATTTCAGCAATACTGTGATGCTGGAAATGTTGAAGGGAGGGCATTCCAACGATTGAAGCAAGAATATTACACTCTTGAGGCAAAACTTGACACTTACTTTCAGATGGCAAAACAAGATTCTTGA

>IbHP6

ATGTTGGGTTTGGGTGCGGAGCGGTTGCGAGTCGACATGAATCGCTTGCTCGCCCTACTCTTTCACCAGGGAGTGTTGGACGAGCAATTCTTGCAACTACAACAGCTCCAAGATGAAGCTTCTCCCAACTTTGTCTCCGAGGTTGTCAACATTTACTTCCATGAATCCGAGAAGCTCCTCAGGAATCTCAGAGTTTTGCTGATGGAGAGTGAAATATGGGACTACAAGAAAATGGGAATGCATTTGAACCAGTTGATGGGAAGCAGCTCCAGCATTGGAGCCAAACGAGTGAGAAATGTATGCGTGGCCTTTCGTGCTGCCGCCGAACAAAACAACCGTTTTGGGTGTTTGAGAGCCTTAGAGGCACTTGACCATGAATACTGCTATCTCAAGAACAAACTCGTAGAACTATTCCAGCTAGAGCAACAGCGGGTTTTAGCAGCTGGAAGGATTGATGATTACCTTGCCAAAAAGAGTAAGCTTTATGGGGTAAAGGACGCTAGATTGGATAGATGTGTTCACTGCTTGGCTGGAAGAGTTTCGTTCATGAGCCATCCTCCTTCAAAGATGTTAGAGCCACTTGATGAGTTGATACATTTTGATGAAGCTTTGGGTCACACTTTGAAGCACAAAAATGATGTGCTTGGTGTGTTAAAGAATTTCATGCTCTTGCTGAGAGACAGAAAGGTCCACAGTCGTGGATGGAGAATAGAAGGTTCCACATCCAGGCCCACGATTGTGGATGGGAACGTGGCTAGGATAAGTGGCACTGTCCACGAGCGCTCCATGCTCATGGACAGGTTCGTGGACGGGAATCGGCGGAAGCAATTGGAGAATGTGCTTGGAGGGAAGACTATTTAA

>IbHP10

ATGGACGTCGATCTCCTCCAACATCAGCTCATTGCACACATTCAAGCTTTGCAGCGTGAAGGCTATGTTGATGAGTATCTGCAGATATGCTATGGGTTGAAGGAGACTTCTGGCTTAACATTTTTCCTAGAATTGATTGCTACCTTTCTCACAAACTCTGCTGCTACTATACATGATATGACTCAAACTATGGAATACCCGATTCTTGACTATGATAAGATGCAAAGGCTTGCAATCAGGCTCAAGGGAAGTTCATCATGCATTGGGGCATGTCGGATAAGTGCTAGTTGCTCTGAGTTACGCCAGGCAGCTACAAAAAGATCTAAGATAAACTGCAAACGGGTAGTGGAGATGATCAACGGAGAAAAATCCGCATGGGAAATCAAGTTGGAAACTATTATGCAGCTGGAGCGTCAGATCGTTGACAAACAGATGCAGTGA

>IbHP11

CATGGACGTCGATCTCCTCCAACAGCAGCTCATCGCACACATTCAAGCTTTGCAGCGTGAAGGCTACGTTGATGAGTATCTGCAGATATGCTATGGGCTGAAGGAGACTTCTGGCTTAACATTTTTCCTAGAATTGATTGCTACCTTTCTCACAAACTCTGCTGCTACTATACATGATATGACTCAAACTATGGAATACCCGATTCTTGACTATGATAAGATGCAAAGGCTTGCAATCAGGCTCAAGGGAAGCTCAGCATGCATTGGGGCATGTCGGATAAGTGCTAGTTGCTCTGAGTTACGCCAGGCAGCTATAAAAAGATCTAAGATAAACTGCAAACGGGCAGTGGAGATGATCAGCGGAGAAAAATCCGCCTGGGAAATCAAGTTGGAAACTATTATGCAGCTGGAGCGTCAGATCGTTGACAGACAGATGCAGTGA

>IbHP7

ATGGATGATTCAAGCACCTTTCCTCAGGCCGCCTCGGGGTCCGCTAGTGCGAATGGAAAGACCAGAGTGGATGTTCTCAAACAACAAGTGATTGCTCACATTGAACAGCTGCAGCGAGAAGTATCGATCGATTCATTTTATGATGATCAGATATCAGGCTTTGTTGATGAGTATTTCAGGATGAGCTACGGGCTGAAGGACGATACTTCTGAGAAAACATTCTTCATAGATTTGGTTTCTACGTTTCTGATGGAGATTGCCGCCACCATAGATGATATGATTGATAGTCTGGAGTATCCAATCATTGACTATGACAAACTGTATCAGCTTTCCATGAAGTTGAAAGGGAGTTCATCATTCATTGGGGCTTGCCAGCTTTCAACTGGTTGCATCAACTTGATTCATGCCATTGTCAACAAATATATGAATGAGTGCAGGCGGATAGTGGAAAAGCTCAATAAGGACAAAGTTGTCTTGGAAATGAAGTTGGTGGAGGTTATGGAGCTGGAGCATGAGATTGTTGCAGACGGTGAATGA

>IbHP8

ATGGATGCTTCAAGCACCTTTCCTCAGGCCACCTCGGGGTCCGCTAGTGCAAATGGAAAGACCAGAGTGGATGTTCTCAAACAACAAGTGATTGCTCACATTGAACAGCTGCAGCGAGAAGTATCGATCGATTCATTTTATGATGATCAGATATCAGGCTTTGTTGATGAGTATTTCAGGATGAGCTACTGGCTGAAGGACGATACTTCTGAGAAAACATTCTTCATAGATTTGGTTTCTACGTTTGTGATGGAGACTGCCGCCACTATAGATGATATGATTGATAGTCTGGAGTATCCAATCATTGACTATGACAAACTGTATCAGATTTCCATGAAGTTGAAAGGGAGTTCATCATTCATTGGGGCTTGCCAGCTTTCAACTGGTTGCATCAACTTCATTCATGCCATTGTCAACAAATATATGAATGAGTGCATGCGGATAGTGGAAAAGCTCAATAAGGACAAAGTTGCCTTGGAAATGAAGTTGGTGGAGGTTATGAAGCCGGAGCATGAGATTGTTGCAGACAGTGAATGA

>IbHP9

ATGGACCTCGCTCTCCTTCAGCAGCACCTCATTGCACACATTGAAGCTTTGCAACGTGAAGGTTATGTTGATCAGTATCTGCTGATGTGCTATGGGCTGAAAGAGACTTCTGGCATAACATTCTTCCTAGAATTGATTGTTAATTTTCTCAGAGAAGCTGCTGCTGACATATATAATATGACTGCCACTGTTGGATATCCGATTCTGGACTATCATAAGATGCATGAGCTTGCCATCAAGCTCAAGGGAAGTTCTTCATGCATTGGGGCTTGTAGGATAAGCGTTGCTTGCACTCAGTTACTCCAGGAAATTAGTAAAAGATCTGAGATAGACTGCAAACTGGCAGTGGAGATGATCATCACAGAAAAATCCACATTGGAAATCAAGCTGGATACTATTATTAAGCTGGAGCGTGAGATCGTTGACAGACAGTTACAGTGA

>g47182

ATGGCGAGGAACGGGGTGTTTTCGCGGCGGCGGACGGCGGCGGAGATGGAGGATTCCGACGAGGTGGTGCTGTCGTCGGAATCTCACGATGTTCATGTTCTCGCCGTCGACGACAGCCTCGTCGATCGGAAAGTCATTGAAAAGTTGCTGAAAATTACGGCCTGCAAAGTGACGACGGTGGATAGTGGGAGGAGAGCTCTGCAAATTCTGGGACTGGACGAGGAGAAAACCTCTGTCCAATTCGATGGTTTGAAGGTGGATCTGATAATCACAGATTATTGTATGCCTGGGATGACTGGCTATGAATTGCTCAAAAAGATTAAGGGTTCATCGTTTAGGGAAATACCAGTGGTGATCATGTCGTCCGAGAATGTTTTGGCACGAATAGACAGGAAGGATGTCCACGTGTTCGGTCTTAACTGCTTACCTAAAATGTTCTTGTTCTGGTTACTTTTTGCCTTTACTAGTAGGGTTATAGAGCTGTCAATACGGGCCAATCCGTCACGGGTTGGCGAAATACAGGATGGGTTGGGATTTCTCCGACCCAACCCGTCCAAAACACGGGTTAAACGGGCCCAACCATGGCCCGTTTTGACAGCTCTAGTCGGTTATGGACTTACGGCTATCATGATGGCTAGAGTTCGATGTTTGGAAGAAGGCGCCGAAGATTTTCTCTTAAAGCCGGTGAAATTGTCCGACGTTAAACGCTTGAAGAGCTACATGTTTGGCGACGACCGGTTTCATGGCGAAGACGGCGGGACAAACAAGCCGGAAACGCCAGAAATATCCGACGACACATCGTCATCATCGGCGCCATCTCTCGCTCTCGCCATCACCAACGACGTCAATGGATCTCTCTTCATGTCTCTCGCTCTCGCCATCTCCAACATCAACTGA

>g12608

ATGATGTCGTCGTCGTCGTTCAGCGTATCCCGCAAGAACTCCGGCCGCCGGGCGGCAGACACATGGGATCCAGAAACTCTTAGCAGCCAGCCGGCGAGCGGAGGAGACAACGGTGGGTTTACCTCAAAGTTGTCGAGCAAGATGAGCCAACAGTTTGAGGCGATTAAGGTGGGGTTTCGACGGATTAAGGAGAGCGAAAAGTTGGATAAGATTATGGTCTTAAGCTCCAAAGGCGTGGAGAAAGTGAAGGATGGGGCGTGTTCTGGTTTTCAGTGGATAAAGGACAAGTGCCGCAACACTGCTCACAATTCACGAGTCGTCATTACTAAACGGTACGAGTTATTGGGCCTATGGACACTACTGGGCTCAAGAGGAAGAAAATTGATAATGGGCCTACTCACATTGCTGAAGTCTGCTGGATGGAGGAAGTATTTAGCTCATCTACTGGGCCAAAAAACTTGCAATGGCTGGCTCAAGATTAATGTGGATGTTGTCGTAGACACTATCGAGGGACGAATGCGTTTCAATGACTTGAACGGGAAATTCCCATCTCGCCTAATTATCCACGAGAACGTGGATGAGGACTATTTTCCGTTTCCGTGGGAGTATACTCCCATCTGTGTGGGAGAGATGGCGAGGAACGGGGTGTTTTCACGGTGGCGGAGGGCTGAGGGACCCGCCGGATTGTCGCTTCCCTCAGAATCTCACGACGTTCATGTCCTCGCCGTCGACGATAGCCTCGTCGACAGGAAAGTAATCGAGAAATTGCTCAAAATTACATCCTGCAAAGTGACAGCAGTGGATAGTGGGAGTAGAGCTCTGCAATTCTTAGGATTGGACAGGGAAGAGAGTTCTGTTGGATTTGATGGTTTGAAGGTGGATATGATAATCACAGATTATTGCATGCCTGGAATGACTGGCTATGAATTGCTCAAAAAGATTAAGGGATCATCTTTTAGGGAAATTCCTGTTGTCATAATGTCATCTGAGAATGTTTTGGCAAGAATTGACAGATGTCTGGAAGAGGGGGCGGAAGATTTTTTGTTGAAGCCAGTGAAACTGTCGGATTTAAAACGATTGAAGAGTCACATGTTTGGCGAGGACGACAAGAATCCAAGAGAAGACAGCGGGATAAACAAGCGAAAGTTACAAGAAATGTCCGAGGATTCATCGCCTCCCTTGCCTTCACCATCACCATTGCTGTCTCCAAATCCGTCAACCGATCTTTCTTCTTCTTCATCATCATCATCATCATCATCGTCCTCCCCACCCTCCACTTCATCATCACCTTCCTCACCTGAACTACTTGAATCTCCCAAGACAGAGGAATGA

>g12082

ATGGACACTACTGGGCTCAAGAGGAAGAAAATTGATAATGGGCCTACTCACATTGCTGAAGTCTGCTGGATGGAGGAAGTATTTAGCTCATCTACTGGGCCAAAAACTTGCAATGGCTGGCTCAAGATTAATGTGGATGTTGTCGTAGACACTATCGAGGGACGAATGCGTTTCAATGACTTCTGTGTGGGAGAGATGGCGAGGAACGCGGTGTTTTCACGGTGGCGGAGGGCTGAGGGACCCGCCGGATTGTCGCTTCCCTCAGAATCTCACGACGTTCATGTCCTCGCCGTCGACGATAGCCTCGTCGACAGGAAAGTAATCGAGAAATTGCTCAAAATTACATCCTGCAAAGTGACAGCAGTGGATAGTGGGAGTAGAGCTCTGCAATTCTTAGGATTGGACAGGGAAGAGAGTTCTGTTGGATTTGATGGTTTGAAGGTGGATATGATAATCACAGATTATTGCATGCCTGGAATGACTGGCTATGAATTGCTCAAAAAGATTAAGGGATCATCTTTTAGGGAAATTCCTGTTGTCATAATGTCATCTGAGAATGTTTTGGCAAGAATTGACAGATGTCTGGAAGAGGGGGCGGAAGATTTTTTGTTGAAGCCAGTGAAACTGTCGGATTTAAAACGATTGAAGAGTCACATGTTTGGCGAGGACGACAAGAATCCAAGAGAAGACAGCGCGATAAACAAGCGAAAGTTACAAGAAATGTCCGAGGATTCATCGCCTCCCTTGCCTTCACCATCACCATTGCTGTCTCCAAATCCGTCAACCGATCTTTCTTCTTCATCATCATCACCATCATCATCATCCTCCCCACCCTCCACTTCATCATCACCTTCCTCACCTGAACTACTTGAATCTTCCAAGACAGAGGAATGA

>g33858

ATGGTCATTGGGAAGCCGGAGAAAGTCGCCGCCGGTGATGATTGCTGTGCCGGCGGTGTACAGGAGTTGCATGTTCTTGCCGTCGATGATAGCCACGTGGACAGAAAGGTCATTGAGAGGTTGCTCAAAATCTCTGCCTGTAAAGTTACAGCAGTAGAGAGTGGGAGCAGAGCTCTACAATATCTGGGCTTGGATGGAGAGAAAGGCTCTGCTGCAATTGATGGTTTGAAGGTAAATCTGATAATGACAGACTATTCAATGCCTGGGATGACTGGTTATGAACTACTCAAAAAGATTAAGGGTTCTTCAGCACTGAGGGAAATTCCTGTTGTGATAATGTCATCTGAAAACATTTTAGCTCGCATTGATAGATGTCTGGAGGAAGGGGCCGAGGAGTTTCTGATGAAGCCTGTTAAGCTGTCCGATGTAAAGCGTCTCAAAGATTTCGTCCTGAGAGGAGACGGGGAGAGCAAAGAGGGAGCAACAACCCGGAAGAGGAAACCGACAGACGACTCCTTCATAATGCCGCCATTATCTCTTTCCTTGGCGTCGTCTTCGCCCTCCATCCATCCCGAGACCACTACGCCTCTGTCCCCGAGGTGTTCATCAGTTCCTCTCTCAAAACATCCTAGACTGCACCAAGACACTGAGCCACTGAGTCCACCCCACCGTGTATTTTCCTTTCCGGATCCTCAAGCTCTTCTCCACCGACTCAAGTTTGGTGTACTGAAACGAATCAATCCAGAACACGAGCTTCACCATAGCTATGGAGTCCAAGAGAACGGCGCTGCCCAGGAGGATATAAGTGACAGAAATGTCCCGGACATGAATGCTGCGCTTGTTGCGGTAGAAAAAGATGATGGTGACGACTATAAAAGCTGCAGACTTGAAAGCTGGAAATCCTCCATGGATTTAACTCCAATAATCCATCGCCGTCCATTTTCGGCTCACAGAAACTCAACGCCGAGCTCACTACGAGGGAGGCGTTCCTGGCCGATCATCATCAAGGATCTTCCGGCGCTCTGGCTTCCTTCATCGAATCATCATCCGCCGGCGTTCCAGACCTGGACTTCTCCGAATGTTATCCTGAATGTTTACGGAGACGATGCTCAAATCAGTCTTTGCTGTCCTAAGAAGCCATGCCTCCTTCAGGCCATTTGCTTCGTTCTGGAGAAGCATAAAATCGAGGTGGTTTATGCTCAGGTTTCATCGAATCATCACCGCACTAGTATTTCCGGCGCTCACTTTTGTGATTTTGCTTCGTCGTCGTCGTTGTTTTGA

>g58574

ATGGGCATGGCAGCAGTAGAGCCACAGTTCCATGTTCTAGCTGTTGATGACAGCCTCATAGATAGAAAGCTCATTGAGAGACTTTTCAAGACCTCTTCTTGTCAAGTAACTACAGTGGATTCTGGTAGCAAGGCCTTACAATTTCTGGGATTGAATGAAGATGACCAGAAAAATCCAATTCAACCTTCTGTTTCCCCCAACAATCATCAGGAAGTGCAGGTGAATCTGATCATTACAGACTATTGCATGCCTGGGATGACAGGCTATGATTTGCTCAAGAAAATTAAGGAATCTTCATCTCTGCGAAACATACCTGTGGTCATTATGTCATCTGAGAATGTTCCTTCAAGAATCAGCAGATGCTTAGAAGAAGGGGCAGAAGATTTTTTTCTCAAGCCAGTGAGGCTATCAGATGTGAATAAGCTCAGACCCCATATGGTGAAAAACAGGAAGGCAGGAGAACAAGAAATTCAAGAATCATCATCATCAGAGGAGTCATCTGCAGAATCTGGTATGACAGATGTTCAATCACAGGCGAATCGAACGATAATCGTTGTAATAAGAGGAAGGCCTTGGATGAAGGTTTTACACAAGAGAACAAGAACAAGATGCAATAGCCTCACTGCTTTCTCTGATCTATGA

>g53645

ATGAGAACGGAGAAAATCGCCGCCGTTGATGGCTGTTCTTCCACCTTTGTCGGCGGTGGCCGGGAGTTGCATGTCCTTGCCGTCGACGATAGCTACGTGGATAGGAAGGTCATTGAGAAGATGCTCAAGATTTCTTGCTGTAAAGTGACAGTAGTAGATAGTGGTAGCAGGGCTCTGCAGTATTTGGGCTTGGAGGGAGAGGAGAGCTCTGTTGCAACTGATGGTCTGAAGGTGAATCTGATAATGACAGACTATTCAATGCCTGGGATGACAGGATATGAGCTCCTTAAAAAGATCAAGATTTTAAATGCAGATCTTGAAACTGTCATAATTGTAAATAATAATACATTAGTTCTTCTTGATCAGGGTTCGTCAGCATTGAGGCAAATCCCCGTTGTGCTAGGTTTCACCCGCATTCTTGATTCTATGTCATGCGTAGAAGATTCTTTGCTTCTCAGGGCAGAGGAGATAGAATCCCAGTTTTGTCTGGAGGAAGGGGCTAAAGAGTTTCTCATGAAGCCCGTTAAGCTGTCTGACGTGAAGCGTGTGGTAGATTTCATACTGAGAGGCGAGGAGGATGGCAACGAGACAGAATCAACGACGGGTTCCTTTTCATCATCAGCTCCTGATAACACGCCATTATCTCCAGAGTCGTATCTCGCTAACATTGCCAGAATCTCTCTCGACGCATCCAACAGAACATAG

>g24894

ATGGGCATGGCAGCTGCAGATCCACAGTTTCATGTTTTGGCTGTTGATGATAGCCTCCTGGATAGGAAGCTCATTGAGAGGCTCTTCAGAACCTCCTCTTGTCAAGTTACTGCAGTTGATTCTGGTAGTAAGGCCTTAGAATTTCTTGGATTACTAGAACATGGCCAGGATTGCCAAACCCAACCTTCTGTTTTACCCAACCACAATCAGGAAGTGGAGGTCAATCTTATCATCACAGACTACTGCATGCCTGGGATGACAGGCTATGATTTGCTCAAGAAAATCAAGGAATCTTCATCTCTGAGAAACATACCAGTTGTCATCATGTCATCTGAGAATGTTCCTTCTAGAATCAATAGATGCTTAGAGGAAGGGGCAGAAGAGTTTTTCCTGAAGCCAGTGAGGTTGTCAGATGTGGATAAGCTTAAACCTCATATGATGAAAACCAAGGGCAATAAACAGCAGAAAGCAGGGAGTGATGACACCCAAGAACACAAAGAAACATCATCTGAAGAGTCATCATCAGTTGAGTCTGGTGTAACAGATGTTCAATCACAACTGCCACAACTACCATTAGAACAGCCACAATCAGAGACACAACTGCACCAACCGCCACCCGATAATAACAATAATTGTAACAACAAGAGGAAGGCCATGGAAGAAGGCCTTTCACCAGATAGATCAAGAACTAGATACAATGGTCTCACCAGCCTCTAA

>g24753

ATGGAAGGCCTTCCTCTGGAGGGCTATCAAGGATGTTCTCCCTACTACTACAAATCTCATTTTAAAGAGGGTAGAGGTTCTTCCAACATGTCCAATGTGTGTTTATTATCTCTGCTCACGGATGATCAGCTTATATATGCAGCAGCAACACTATATTATATTTGGTCGGCTCGCAACAAGGCGGTGTGGGAGCACTTGCTACCGCGGCCAACCTGGAGAGCGGCTTCGGCGGCGGTGCGAGCCTGGCAGCACGTCCATCATCCGCAGACAGCAAACGCAGCACCTCCACAGCTTGGTTACCACCCGACACCCATTGCAGAACCGTCCAGCCTCATCTGCCATTTCGACGCTGGTTTTCAACCGGCAACGAAAAGGGCAACGGTTGGTGCAATCGTCCGATCTAGCACTGGCGGCTTTGTGGCAGCGTTCAATGGTCTTTTTCGGAGCTGTATGTCGCCTCTAATGGCGGAATCCCTTGCATGTAAGGAGGTACTTTCCTGGATGAAGGATAAAGGCATTGATCGTGTCGTTCTTCACACTGATAATTCGATCCTTCAACGATGGCTTACGGCGGAGAATAATGAGTTCTTTTCTTATATTGCTTTTTCTATTGATGCCTCTAGGGCTATTATGTCGTCATTTTCGCATTGTTCCATTAGGTGGGTGCCAAGAACGGCTAATTTAGGCGGTAGTGGGAGGCCATGGAGGCTTAGGATGATGGTGGGTGTGGATTGGATCGCTTGTGCAAGCATGAATGCTAAGATCTTCTTGACAGACGCTGATTTACCTCTCATGACTTTTAGACATGGATTGGAAAACCGGGGTGGAGATATCTTGGCAAGAAATCTGGTCAGATCTTGCTTTGTGGAAGAATTTGGAGAAATGGGCATGGCAGCTGCAGATCCACAGTTTCATGTTTTGGCTGTTGATGATAGCCTCCTGGATAGGAAGCTCATTGAGAGGCTCTTCAGAACCTCCTCTTGTCAAGTTACTGCAGTTGATTCTGGTAGTAAGGCCTTAGAATTTCTTGGATTACTAGAACATGGCCAGGATTGCCCAACCCAACCTTATGTTTTACCCAACCACAATCAGGAAGTGGAGGTCAATCTTATCATCACAGACTACTGCATGCCTGGGATGACAGGCTATGATTTGCTCAAGAAAATCAAGGAATCTTCATCTCTGAGAAACATACCAGTTGTCATCATGTCATCTGAGAATGTTCCTTCAAGAATCAATAGATGCTTAGAGGAAGGGGCAGAAGAGTTTTTCCTGAAGCCAGTGAGGTTGTCAGATGTGGATAAGCTTAAACCTCATATGATGAAAACCAAGGGCAAGAAAGCAGGGAGTGATGACACCCAAGAACACAAAGAAACATCATCTGAAGAGTCATCATCAGTTGAGTCTGGTGTAACAGATGTTCAATCACAACTGCCACAACTACCATTAGAACAGCCACAATCAGAGACACAACAGCACCAACCGCCACCAGATAATAACAATAATTGTAACAACAAGAGGAAGGCCATGGAAGAAGGCCTTTCACCAGATAGATCAAGAACTAGATACAATGGTCTCACCAGCCTCTAA

>g55375

ATGGGCATGGCAGCAGTAGAGCCACAGTTCCATGTTCTAGCTGTTGATGACAGCCTCATAGATAGAAAGCTCATTGAGAGACTTTTCAAGACCTCTTCTTGTCAAGTAACTACAGTGGATTCTGGTAGCAAGGCCTTACAATTTCTGGGATTGAATGAAGATGACCAGAAAAATCCAATTCAACCTTCTGTTTCCCCCAACAATCATCAGGAAGTGCAGGTGAATCTGATCATTACAGACTATTGCATGCCTGGGATGACAGGCTATGATTTGCTCAAGAAAATTAAGGAATCTTCATCTCTGAGAAACATACCTGTGGTCATTATGTCATCTGAGAATGTTCCTTCAAGAATCAGCAGGGTGCTTATAAAACCTAAACAGATGCTTAGAAGAAGGGGCAGAAGATTTTTTCTCAAGCCAGTGAGGCTATCAGATGTGAATAAGCTCAGACCCCATATGGTGAAAAACAGGAAGGCAGGAGAACAAGAAATTCAAGAATCATCATCATCAGAGGAGTCATCTGCAGAATCTGGTATGACAGATGTTCAATCACAGGCCGAATCGAACGATAATCGTTGTAATAAGAGGAAGGCCTTGGATGAAGGCTTTACACCCAAGAGAACAAGAACAAGATGCAATAGCCTCACTGCTTTCTCTGATCTATGA

>g42973

ATGGGCATGGCAGCTGCAGAGTCACAGTTTCATGTTTTGGCTGTTGATGATAGCCTCATAGATAGGAAGCTCATAGAGAGGCTCTTCAGAACCTCCTCTTGTCAAGTTACTACAGTGGATTCTGGTAGCAAGGCTTTAGAATTTCTGGGTTTACATGAACATGATGATGAGAACAACACAAACCATCATCCATCTGTTCTATCCAACCATCCCCAACCCCAGGAAGTAGAAGTGAACCTTGTAATTACAGACTACTGCATGCCTGGGATGACAGGCTATGATCTACTCAAGAAAATTAAGGAATCTTCATATCTGAGAAACATACCTGTAGTCATTATGTCATCTGAGAATGTTCCTTCAAGAATCAGTAGATGTTTAGAAGAAGGAGCAGAGGAATTCTTCCTCAAGCCTGTGAGGTTATCAGATGTAAATAAGCTTAGACCTCATATGATGAAAACCAAATGCAAGAAGCCCGAAATCGATCCCCGGGACAGCCAAGAACCATCCCCAGAACACCCTCTAATCCAACAAGAATGTGCTGTAGAAGATGTGAAATTGCAGCCCCAAAACCCGCCCCCACCGCAGATAGAAGAGCAACAACCAGTGATGAATAGCGATGAAGACTCGAGGAAGGCCATCGAAGAAAGCCTTTCACCGGGAAGAACAAGAACGAGACAAGAACCACAAGGGCGGGGGGACGAGCCACAGCCACTGGTGAATAATAGTAATGAAAACAAGAGGAAGGCCATGGAAGAAAGGGTTTCACCAGATAGAACAAGACCAAGATACAACAATAATGGTCTCACTCACTGCTGTCTCTAA

>g42966

ATGCTTAATCCAGATTGGAAAATTCTGGGAGATCTTTGGTTGAGATCTGGTCAGACCTTGCCCTTTCTGTGTGTTGTTGTTGTGGATTTTATTTTGGAAGCTCATAGAGGCTCTTCAGAACCTCCTCTTGTCAAGGTAGTTACTACAGTGGATTCTGGTAGCAAGGCTTTAGAATTTCTGGGTTTACATGAACATGATGATGAGAACAACACAAACCATCATCCATCTGTTCTATCCAACCATCCCCAACCCCAGGAAGTAGAAGTGAACCTTGTAATTACAGACTACTGCATGCCTGGGATGACAGGCTATGATCTACTCAAGAAAATTAAGGAATCTTCATATCTGAGAAACATACCTGTAGTCATTATGTCATCTGAGAATGTTCCTTCAAGAATCAGTAGATGTTTAGAAGAAGGGGCAGAGGAATTCTTCCTCAAGCCTGTGAGCCAAGAACCATCACCAGAACACCCTCTAATCCAACAAGAATGTGCTGTGGAAGATGTGAAATTGCAGCCCCAAAACCCGCAACCACAGGCACAGACAGAAGAGCAAAAACCAGTGATGAATAGCGATGAAGACTCGAGGAAGGCCATCGAAGAAAGCCTTTCACCGGGAAGAACAAGAACGAGACAAGAACCACAAGGGCGGGCCGAAGAGCAACCGAGTAATGAAAACAAGAGGAAGGCCATGGAAGAAAGGGTTTCACCAGATAGAACAAGACCAAGATACAACAATAATGGTCTCACTCACTGCTGTCTCTAA

>g30557

ATGGCGACTTCTTCCTGCTCAGATCTTGGCAAGTTTCATGTCTTGGCTGTTGATGATAGCATCATTGATCGAAAGCTCATCGAGAGGCTCCTCAGAACTTGTTCTTATCAAGTGACGGTGGTGGATTCTGGGGTGAAGGCGATGGAGTTTCTGGGCGGGAGGATTGAGGAAGTGAATTTGATCATAACGGATTACAGTATGCCGGGGATGACGGGGTACGAGCTTCTGAGGAAGGTGAAGGGGTGTTCTTCATTGAAAGATATTCCGGTGGTGATCATGTCGTCGGAGGATGTTCCGGCGAGGATCGACCGGTGTTTGGAGGAAGGGGCGGAGGAGTTTTTCCTGAAGCCGGTGCGGCAGGCGGATGTGAACCGCCTGAGTTCCCATTTGCTGAGACCTAAATCTCCAGAACCTTCGCCCTGCCGGAAAAGGAAGGCGGCGCCGGCGGAGGCGCAACCAACCAGGCCGATAAGAAGAAGAAGATTGATCTGA

>g20447

ATGGCAACTTCTTCAAGGAATGGGGGAGATGAATCACCTCATGTACTAGCTGTTGATGACAACCTCGTCGATCGCAAACTCGTTGAAAAGCTGCTCAAGAATTCATCCTGCAGAGTGACTACTGCAGAGAATGGGTTGAGGGCTTTGGAGTATTTGGGGTTGGGAGATGAACAACACAACACTTCAAATGACAATGGATCAAAGGTGAATATGATCATCACAGATTACTGCATGCCAGAAATGACAGGCTATGAGCTGCTCAAGAAAATCAAGGAATCTTCCAACATGAAGGACATCCCTGTTGTGATAATGTCATCTGAGAACATTCCAACTCGGATCAACCAATGCTTGGAGGAAGGAGCCCAGATGTTCATGCTAAAGCCACTCAAACACGCGGATGTGAAGCGATTAAGAGGCGAATTGATGCAGTGCAGAGGCTAA

>g5095

ATGGCGTTGCTTTCATCATCACCATCTTCTTGTTCGTCATCAATGGCGGAGGCGGAAGACGAGATTCCGCATGTGTTAGCTGTGGATGACAGCTCAGTAGACCGCAAGCTCATCGAAAGGCTACTCGCTACCTCTTCCTGCAAAGTGACCACAGCGGAGAATGGGCAGAGGGCTCTGGAATTTTTGGGGCTCGGAGAGGGGCATACCAGTAACAGGCAGTCAAAGGTGAATTTGATCATAACAGACTACTGCATGCCGGGGATGACAGGCTATGACCTGCTTAAGAGAGTCAAGGGATCATCTGACCTGAAAGAGATACCGGTAGTGATAGTGTCATCCGAGAATGTTCCAACAAGAATCAAGAAGTGCCTGGAAGGAGGGGCTCAAGAATTCATGATAAAGCCGTTGAAACAATCAGATGTTAAGAAATTAAGATGCCATATGGCTAAGTTTAAGCAGCCCTGCAGTGGACGGTTATGCATTGGAAGATAA

>g6428

ATGGCGTTGCTTTCATCATCTTCTTGTTCGTCATCAATTGCAGAGCCGGAATACGAGATGCCGCATGTGTTAGCTGTGGATGAAAGCTCAGTGGACCGCAAGCTGATCGAATGGCTACTCGCTAGCTCTTCCTGTAAAGTGACCACAGCGAAGAATGGGCAGAGGGCACTGGAATTTTTGGGGCTGGGAGAGGGGCATCATGCCATGACAAAAACTTCCATAAAGATAAAGAATGAATATTCCAGTGAAAGGGAGAAAGAACATGTAGAAACTTCCATAAATTTTGATCCTCTGTACCAACTTTGTTCTACTGAGATTTTCAAAGTTTACAATTTTTCCTTAATAAAAAAAATCAAGGAATCAGAGTCCAGAAGCTATGGAGTGATCGTCAATAGCGTTTACAAGCTTGAACCCGAGTATGCCCTCCTCTCCTCTTTAACACAGAAATTGAAGAGAAAGCCCTACAGGAAAAAGGAACAAAATTATCACCCTTATCTAGCGAAGAACGACACGAGATCTTGA

>g35948

ATGCCGGAAGGATGCGAGAGGATGGACCAGCTGACCTTCGACGACTTGGTCCCCAAATACTTCAAAGCCACCGCCATGCTTGTGGATGACAGCTCAGTAAACTGCAAGCTCATCGAAAGGCTATTCGCTAGCTCTTCCTGCAAAGTGACCACAACGGAGAATGGGCAGAGGGCACCGAATTTTTGGGAGTGGACGAAAAGAGTTCTAGAAGGTAGAGCTATGGAGTATGGACTGGAGAACATCGCCGGAAGTCTGCCGCCCGCTGCCGTCTTGGCGGAGAACTTCGACGATTCACGCCGATCAACGGTGAATCGAACTGGCCGCTTCGGCAGAAGCGCAGACGCCGCAGGCGCCGCTGACCAACATTGTCGCCGCTACTGGTGA

>g41064

ATGCTTGTGGATGATAGCTCAATAGACCGCAAGCTCATCGAAAGGCTACTCGTTAGCTTGGTTTCATCTCCGACTGAGGCTAAGGAGCGGAGGCTTCGAAGGAAACGACCGGCGGTTGAGTTTCGCCGCGACCAATTTCTTCGCTGTCATCCTCACCGCAAAAATCGGAGACAACAAAGCTTTAACCGGCATCCTAACCGGCGACCTCCCCTGCTCTTAGCGAGCGGTGTTGCAGCGGTAGCTGGTTGCGGAGATGATGTTTCCGTGGAAGCCGCCAGCGAGTGTTCTCTTTCCAGTGAGCAGCTATGGCGTCGGCGAGGTTTGGGGATCGGCGGAGGAATCTCTCCTCTTACTCCCTGCTTTTCTCCGCAAAGTATCAAAGGCGTGACCGGCACGTGGTCCATCTCCGACCATCTCTCTTCTCCCGCGTAA

>g8841

ATGGCAACCTTGGATGATGGTTCGGAGTTTTTCCACGTTCTTGCCGTGGACGACAGCGTGGTGGACCGGAAACTCATTGAGAGGCTGCTGAAGACATCCTCGGAGAACGTAAAAGTGACGGTGGTGGATTCCGGGAGCAAGGCGTTAGGGCTGTTGAGCGAGGTGGAAGTGAATCTGATCATCACGGATTACAGCATGCCGGGGATGACGGGGTACGATCTGCTGAGGAAGATCAAAGGCTGCGCCGCGTTCAAGGATATTCCGGTCGTGATCATGTCGTCCGAGGATGTCCCGTCCAGGATCACCAGGTGTTTGGCGGAAGGCGCGGAAGAGTTCTTCCTTAAACCGGTTCGACAGTCCGACGTCAACCGCCTCAAACCGCATCTGTTAAACGGCTGCAAAGGAAAGGCGGCGTTTTCCCCAGATTCCGCGGCGGCGGTTTGCGGGATCGTTTGA

>g30555

ATGGCGGCAGCGGTGGTGGCGGCTACTCATGCATCAGAATCAAGATTTCACGTTCTGGCCGTCGATGATAACCTCGTGGATAGAAAGTTGATCGAAAGGCTTCTCACAACTTGTTCTTACCAAGTGACTGTAGTGGATTCTGGGAACAAGGCGCTGGAGATTCTTGGGCTGTTGGAAGATTCCGTGACGGCTCTGAACTCCGACCACCACGAAGTGGAAGTGGATTTGATCATCACAGACTACTGGATGCCGGGGATGACGGGGTACGATCTGCTGAGAAAGGTGAAGGAATGCCGGAGAGACATTCCGGTGGTGATAATGTCGTCGGAGAACGAAGCGTCGAGGATCAACATGTGCTTGGAAGAAGGCGCGCAGGAGTTTCTGGTGAAGCCCGTCCGCCAATCTGACGTCACCAATCTGATCAAACCGCGCCCGTTTGTGAAAGGCGGCGACGATAACGGCGTTGTTTCGCCGGTGTATTGTTCAGGCGTCGATGATAATCGACATGTAACGGCGACGGAAACTGTTATCTCCCCGGCGGACCGATAA

>g3932

ATGTCTGCAGTCATGCATGTGCTGGCGGTTGATGACAGTACGGTTGACCGTACCATTGTTGAGCAATTGTTCAAGGCCGCTTCTTGCAAAGTAACAACCGCAGAGAATGGATTGAGAGCCTTGGAGTTCCTGGGGTTATTAGCAGGAGATGACCAAAACAACTCTCCAAACACCAACGTCCCAAAGCTGAATTTGATAATAACAGATTACTCCATGCCTGAAATGAATGGCTATGAGTTTCTTAAGAAAGTCAAGGGCTCAGCCATGTTTAAGGATGTTCCAGTTGTGGTAATGTCATCCGAAGACACTCCAAGTCAGATCAACCAATGCATGGAGGCAGGAGCTAGTGTGTTCATCCTGAAGCCTCTCAAGCAAGCTGATGTGAACCAACTGAAATCTCAGTTAATGCAAGCCTGA

>g3934

ATGTCTGCAGTCATGCATGTGCTGGCGGTTGATGACAGTCTGGTTGACCGTACCATTGTTGAGAAATTGTTCAAGTCCGCTTCTTGCAAAGTAACAACTGCAGAGAATGGATTGAGAGCCTTGGAGTACCTGGGGTTATTAGCAGGAGATGACCAAAACAACTCTCCTAACACCAACGTCCCAAAGGTGAATTTGATAATAACAGATTACTCCATGCCTGAAATGAATGGCTATGAGTTTCTTAAGAAAGTCAAGGGCTCAGCCATGTTTAAGGATGTTCCAGTTGTGGTAATGTCATCCGAAAACACTCCAAGTCAGATCAACCAATGCATGGAGGCAGGAGCTAAGATGTACATCCTGAAACCTCTCAAGCAAGCTGATGTGGACCAACTGAAATCTCAGTTAATGCAATCCTGA

>g3935

ATGGCTTGCCCTTCATCAATGGCAATGGGGGAAATTGGGGAAGATGAAGTCATCCATGTGTTGGCTGCTGATGACGATCCAGTTAACCTTATCATCCTTGAGAAATTGCTCAACTCCTCTTCTTGCAAAGTAACAACTGCAGAAAATGGATTGAGAGCCTTGGAGTACCTGGGGTTATTAGCAGGAGATGACCAACAAAACTCTCCAAACACCAACGTCCCAAAGGTGAATTTGATAATAACAGATTACTCCATGCCTGAAATGAATGGCTATGAGCTTCTTAAGAAAGTCAAGGAATCAGCAATGCTTAAGGATGTCCCAGTTGTGGTAATGTCATCAGAGAATGTTCCAAGTATTATCAACCAATGCTTGGAAGAAGGAGCTCTTATGTTCATGCCAAAGCCTCTCAATCAATCTGATGTGAAGCACTTAATATCTCAGTTGCGCACAGATTGGATAGCCACATTAGAGAACCAAAATAAGCGCCTGAGAGTCAAGGAAGCTCGGAGGCACAACAATGGCGTCCCTTTTGGAGACTCCATTTTCTTCACCTCACTCACTCTTCAACTCTCACTCCTCACTCTTCACACCCACAATCCCACAACCCCAATTCACTGCAATGCTTCCCTGTCAAGCCCTTTCACCTCCCTATTCTCCGCTCTTCCTCCACCACCGGCGGAGCAGTCGACCCTTCGCCGCCGCCGCCGCTGTCGAACCAGGACGAGATTTCGCTTGATGGGTCAAGTAAAGATCGGCGCAAGGTGGTCAAGTTTGCCTGGGAGAAGCTGGTTCGATGGTCTCGCTCATGGCGGTCCAAGGCCAAGACTGACGTTCTTGAACGCACTAACAAGTTCTGAGAATGGTAATGCTAGAACTCAGGTAGGGCATCTCCCTTTAGCTGTAGTGGTGGTTCTTGGAGGTGGATCTTTTGGAACAGCTATGGCTGCCCATGTTGCAAATAGAAAGGCTGAATTGGAAGTTAATATGCTCGTACGAGATCATCGAGTTTGTGAATCCATTAATGAGAATCACTGCAATAGTAAGTATTTCCCAGAGCACAAGCTACCAGAAAACATAATTGCAACAACTGATGCCAAGGCTGCTCTGCTGGGTGCAGATTTTTGTTTCCATGCTGTACCTGTTCAGTTCAGCTCGGTATTCCTTGAGGATATTGCAATGCATGTTGATCCGAGCTTGCCATTTATATCTCTCAGCAAGGGTTTAGAGCTCAATACATTAAGAACAATGTCTCAGATAATCCCCCGAGCTCTAAGAAGTCCTCGGCAGCCCTATGTCGTTCTATCTGGACCTTCTTTCGCACTCGAGTTGATGAACACGTTACCAACAGCAATGGTGGTAGCGTCTAAAGACAAAAAAGTTGCAAATGCAGTTCAGCAACTTCTGGCCTCTAGAAACCTGAGAATCAATACATCAAGCTTCAGTGATGTTACAGGGATTGAGATTGCAGGTGCACTGAAGAATGTACTAGCAATAGCAGCTGGTATTGTGGAAGGATTGAATCTCGGGAACAACTCCATGGCTGCTCTTGTAGCACAAGGGTGCTCTGAAATTCGATGGTTGGCAACAAAGATGGGTGCAAAGTCAACTACATTAACGGGGCTTTCAGGGACCGGGGACATCATGCTCACGTGTTTTGTGAATCTTTCAAGAAACAGAACTGTTGGGGTTCGTCTTGGATCAGGAGAAAAGCTTGAAGACATACTCGGTTCTATGAACCAGGTGGCTGAAGGTATAACAACAGCAGGAGCTGTGATTGCGCTAGCGCAAAAATACAAGGTCAAAATGCCGGTTTTGACAGCAGTTGCCCGGATTATTGACAATGAACTCACTCCAACTAAAGCTGTTTTTGAATTGATGAATCTCCCTCAGGTTGAAGAAGTTTAG

>g34926

ATGGACATGGGTTCGAAGAATGTTGGGAAGGGGCTGAGGGCGCTTGTGGTCGAAGACGACCCCGCTACGCAGATGGTCCACAAGATGCTGCTGAAGAAATACGGAGTGGAAGCTCAGGTGGCCAAGAATGGCGAGGAGGCGGTGGAGCTCCACCGCTCCGGCGCCCGCTTCGACCTCCTGCTCATGGACAAGGACATGCCTGTCAAGGATGGCGTCAATGCAACTCGGGAGCTGCGGGAGCTGGGCGTGAAGAGCATGATAGTTGGTGTAACTTCGCACGGGCCAGGTGCGGTGAGGGATGAGTTTATGGCGGCGGGGCTAGACGAGTGTCTGATGAAGCCGCTAGGTGCAGATATGGTTTTGCGCCTGATTAACCAGCTGGTGGCCAAGAACGGCGATAAGGCGATGGTGCTCCACCACTTCGACCTCCTGCTCATGGAGAAGGAAATGCCTGTCAAGGATGGCGTCAATAATGTTTCAAGGAATGTGATTGCTTTTTTTGGTAATCTAAGATACCTTGATTATTCCAACTTTGCCCATGTTAAGATTAGCCGCCCACTGCTTTCTCATAACCTCGCAAACCTGCGTCCTGCTCCTCCAACCGCCGCCCGCGAACTGCCGACCGACCACCGTAAACAATCCGCTGCTCACCGCCAGCCGCCGACCGCCGACTGCCGGAAACAAGGTCTGCTCCTCCGAAAACAACCGACCGCCCAATTCCCACTCTCCTCTCCACGCTCCGTCGCTGCACACCGCCCACCTACGACCACCGCTGCCAGTGCGCTGCCACAGTGA

>g34929

ATGGACATGGGTTCGATGAATGTTGGGAAGGGGCTGAGGGCGCTTGTGGTCGAAGACGACACCACTACGCAGATGGTCCACAAGATGCTGCTGAAGAAATACGGAGTGGAAGCTCAGGTGGCGAAGAATGGCGAGGAGGCGGTGGAGCTCCACCGCTCTGGCTCCTGCTTCGACCTCCTGCTCATGGATAAGTCAATGCCTGTCAAGGATGGCGTCAATGGCAACTCGGGAGCTGCGGGAGCTGGGCGTGAAGAGCATGATAGTTGGTGTAACTTCGCACGGGCCAGCTGGGCCCCTCTTCCCTTATTTGGTACACAAGATGCTCTGAAGAAATACGGAATGGAAGCTCAGGTGGCCAAGAACGGCGAGAAGGCGATGGTGCTCCACCACTTCGACCTCCTGGTCATGGAGAAGGAAATGCCTATCAAGGATGGCGTCAATGCAACTTGGGAGTTGAGGGAAATGGGCTTAAAAAGCATGATAGTTGGCGTAACTTCGCACGGGCCCGGTGAGGTGAGGGATGAGTTTATGGTGGCGAGGCTGGATGAGTGCCTAATGAAGCCGTTGGGTTCAGATGTTGTTTTGCGCCTGATTAACCAACTTGATGCTGCTGAAGAAATACGAATTGGAAGCTCAGGTGGCCAAGAATGGCAAGGAGGCTGTGGTGCTCCACCGCTTCGACCTCCTACTCATGGACAAGGAAATGCCTGTCAAGGATAG

>g33149

ATGGGTTCAAAGAATGTTGGGAAGGGGTTGAGGGTGCTTGTCGTCGTAGACGACCCCGCTACCCAGATGGTCCATAAGATGCTACTAAAGAAGTGCGGACTGGAAGCTCAAATGGCCAAGAATGGCGAGGAGGCAGTGATGCTTCACCGCTTCGGCGCTCGCTTCAACCTCCTGCTCATGGACAAGGAAATGCCTGTCAAGGATGGCGTCAATGCAACTCGGGAGCTATGGGAGATGGGGTTGAGAAAGACGCACGGTCACGCAGCCGGTGATGAGTTTATTGTATGGACCGACGAGTGCTTAATGAAGCCGGGTCCGTAG

>g33158

ATGGGTTCAAAGAATGTTTGGAAGGGGCTGAGGGCGCTTGTAGTTGTAGACAACCCCGCTACCCAGATGGTACACAAGATGCTGCTGAAGAAGTACGTACTGGAAGCTCAGGTGGCAAAGAACGGCGAGGAGGCAGTAGTGCTCCACTGCTTTGGCGCCCGCTTCGACCTCCTGCTCATGGACAAGGAAATGCATGTGAAGGATAGCGTCAATGTGAGCCCATTCTTCCCTTTCCCTCTTACTCTTATATATCTATTCCAAAGAGTTGGCGTAACTTCGCACGGGCTAGGTGCAGTCAGGGATGGGTTTATTGCGGCGGGACTGGACGAGTGCCTGATGAAGCTGCCGGGTCCAGAAGTGGTTTTGGGCCTGATTAACCAGCTGGTGGTTGCCTAA

>g33222

ATGGGTTCAAAGAATGTTTGGAAGGAGCTGAGGGCGCTTGTAGTTGTAGACAACCCCGCTACCCAGATGGTACACAAGATGTCAAGAAGACGCATGGAAGCTCGTGGCAAAGAACGGCGAGAAGTGCTCCACCGCTTTGGGCCGACCTCGCTCATGGACAAGGAAATGCCTGTCAAGGATGGCGTCAATGCAACTCAGGAGCTACGGGAGATGGGGTTGAAGAGCATGATAGTTGGCGTAACTTCGCACGGGCCAGGTGCAGTCAGGGATGAGTTTATTGCAGCGGCATTGGACGAGTACCTGATGAAGCTGCCGGGTCCAGAAGTGGTTTTGCCCCTGATTAACCAGCTGGTGGTTGCCTAA

>g33229

ATGGGTTCAAAGAATGTTGGGAAGGGGTTGAGGGTGCTTGTCCGACCCCTGAGATGGTCCATAAGATGGAAGAAGTGCGGACTGGAAGCTCAAATGGCCAAGAACGGCGAGGAGGCAGTGGTGCTTCACCGCTTCGGCGCTCGCTTCAACCTCCTGCTCATGGACAAGGAAATGCCTGTCAAGGATGGCGTCAATGTGAGCCCATTCTTCCCTTTCCATCTTACTCTTATATATTTATTCGAAAAAGGCAACTCGGGAGCTATGGGAGATGGGGTTGGAGAGCACGGGTCACGTGCAGTCAGGGATGAGTTTATTGTGGCGGGACTGGACGAGTGCTTAATGAAGCTGCTGGGTCCAGAACTTAAAAAGTTTGGTGTATCGGTTAACGGCAAGGAGGCGGTGGAGCTCCATCGCTCCGGTGCCCGCTTCGACCTCCTGCTCATGGACAAGGAAATGCTTGACAAGGATGAAGTCAATGCGAGCCCATTCTTCCCTTCCCCTCTTTACTCTTATATATCTATTTCAAAAATGTTAAGAGATAATGGCTTTTGCCTTGATATTGAGAATTGCCTGTATATTCCAGAATGTTCTCGAAATCTTGCTTCAGCATCTAGGACAACAGCATACTTACTCAATAGGGGTCCGAGTAAGGTTGTTTCTAAAACACCTTATGAACTGTGGATGGGAAGGAAACCTAGTTTGAGGGATCTTCACATTTGCGGCTATCAAGCTAAAGTGAGGATATATAATTCACATGAAAATAATTTGGATTCCATAACCATTGGTGGTAATTTCATTGGATATCCAGAAAGGTCTAAAGGATATAGGTTTTATTGTCCTAATCATAGTACGAGGATTGTTGAGTCTGGTAATGCTCACTTCATTGAAAATAACACAGTCTGTGGGAGTGTGGGAGCTCGTAATGTTGAGATTAAGGAGTCATTGATGGATCTATGTTCATCAAGTGATCCTTTGTAG

>g13465

ATGAATCTGGGTGGTGGTCAAGTGGGAAAGGGGATGTCGGCTACGTGCTCAAACGCTTCTTGGAAGTCCGGTGATGCGGTCTCCGACAAGTTTCCGGCGGGTCTCCGGGTGCTGGTTGTGGATGATGACCCCACTTGCCTCAAGATCTTGGAGAAGATGCTTAGGACTTGCCTCTATGAAGTAACCAAGTGCAATAGGGCAGAGCTTGCTCTATCGTATCTTCGGGAAAATAAGAATGGCTTCGATATCGTTATAAGTGATGTCCATATGCCAGACATGGACGGTTTCAAACTTCTCGAGCATGTCGGTCTCGAGATGGACCTGCCTGTAATTATGATGTCAGCTGATGATAGCAAGAATGTTGTGATGAAGGGTGTCACGCACGGCGCGTGTGATTATCTGATCAAACCGGTCCGTATCGAGGCACTGAAAAACATATGGCAACATGTGGTTCGCAAAAGGAAGCACGAGTGGAAGGATAAGGATCCCGAGCAATCAGGGAGTGCAGATGAAGGCGATCGGCCTCAAAAACCATCAGACGATGCTGATTACTCGTCTTCAGTAAATGAAGGGAATTGGAAAAACTCCAAGAAAAGAAAGGACGAGGAAGATGAAGCGGAAGAAAGGGATGACACGTCCACGTTGAAGAAGCCACGCGTGGTTTGGTCGGTGGAGCTCCACCAGCAATTTGTCGCAGCAAACTATGATGCTTCACTTTTCAATCTGCTCTCTACTTTCGTTAGTCATGGCTTTCTGAAAGGCTGTCCCAAAAAAATTCTGGAATTGATGAATGTTCCTGGGCTTACGAGAGAGAATGTTGCTAGCCACCTTCAGAAATATCGTCTTTATCTCAGGAGGTTGAGTGGTCAGGGGGGACTGGGTAATTCTTTTATGGGGCATCCAGAATCGCCCTCCGGGTCTATGTCTTCCTCAATGGGCTTGATCTCCAAGCGGCTAGCCGCCTCGGGTCAAATATCTGCACAGAGTCTTGCTACATTCCAGGCGGCGGCACTGGGCAGTTCTGTCACTAAATCAGCAATATCAATGCCCCTTGTAGACCAAAGAAACCTATTTAGCTTTGAAAACCCAAAGTCGAGATTTGGTGATGGACCGCCTCAACTTGGCAACAGTAGTAAGCAAATCGGTTTGCTTCATGGAATCCCGACAACGATGGAGCCAAAGCAGCTTGCAAGCTTGCACCAATCTTCCCCGACTTTCGGTGGTATGAGTATGCAATTGAACTCCCAGCCCATTTTGTCGAGTGCAATGGCGGGTGGAGTCCTCGGAGGGAATAGTATCGTTGACAATTCGTGCAGTGCCATTCACAGTTCTGTTTCCCATGCCCCATCAACAGTGGCTTTCTCTGTAAACCAGGGCACCGAGTTGCAAACTAATAGCTATACTACTAGCAATTCTGGAGTGTCGTCTCTAACATCTATAGGAATGCTCCGAGAACAGGCTAATCCCGATGTCAAAGGATCGAGAGGATTTGTTCCCAGTTATGATATATTTAACGATCTCCATCAGCATAAAGCTCAGGATTGGGGTTTACAAAACGTCGGATCCACTTTTGATCCCCCTCCTCACCATTCAAATTTACAAGGAATCCTCGACCCCCCACCCTCAGTTATGGCTCAACACGGGTTTTCCTCTAACCAAAAGAGTGGACAGAACAGAAACGCACCCATCAACAAGGACGTTTTCTTAAGCTGGGAACAAACCGGGCATGGAAACAACCCGATGCTCGGTCCACAATTCAACTCACTACTCGGAGGAAATCCAGTCACAATCAAGACCGAGAGACTCCCTGATACGAGCTTTCAGAACACGCTTTTCTCCGACCAATATGGCCAGGAAGACCTAATGAGTGCCCTTCTCAAACAGCAGCAAGATAGCCTTGGGCCAGTCGAGAACGAGTTTGGCTTCGATGGATACCAATTGGATAATCTTCCAGTGTGTGATTGTAGGGAGATCGGTTCATATATAAGTGCTCGTGATGAAGTATCAGATTCGGCGCAAGTCTTCTTCGGTGGGTAG

>g6082

ATGTCAGTTCATAGCTCAGTTGCTTCTTGGAAGCCCGTTGATGGGGTCTCCGACCAGTTTCCGGTGGGTCTCCGGGTGCTTGTTGTGGATGATGACCCCACTTGTCTCAGGATCTTAGAGAAGATGCTAAGAAATTGCCGCTATGAAGTAACTACATGCAATATGGCTGAGGTTGCATTATCTATGCTCCGGGAAAATAAAAATGGCTTTGATATTGTTCTAAGCGATGTACACATGCCAGACATGGATGGTTTCAAGCTTCTTGAGTGCATTGGACTTGAGATGGATTTGCCTGTTATTATGATGTCGGCAGATGATAGTAAGAATGTCGTGATGAAGGGTGTTACGCACGGTGCGTATGATTATCTGATAAAACCAGTCCGGATTGAAGCGCTGAAGAACATATGGCAACATGTTGTTCGCAAAAGGAAGCAGGAGTTGAAGGACAAGGATGTTGAACAATCGGGAAGTGTGGAAGAAGGAGACCGACAGCAAAAACCATCTGAAGATGTCGATTACTCATCTTCGGTAAATGAAGGAAATTGGAAATGCTTGAAGAAAAGGAAGGACGAGGAAGATGAAGGCGAAGAGAGGGATGAGACATCTGCACTGAAGAAGCCACGTGTGGTTTGGTCGGTGGAGCTCCATCAACAATTTGTAGCAGCTGTTAATCAATTGGGAATCGACAAGGCTGTTCCAAAGAAAATTCTGGAATTGATGAATGTACCTGGGCTTACTAGAGAAAATGTTGCAAGCCACCTGCAGCTAGTGCCTAGTAGCGAAGGCGTAACTTTATTTCCTTATAGACTACCGTCACATTTTGCAGAGGTAGGGTTTTGGACTTGGCCCTTCACTGGCCTCTGCCCAACAATCTGCCTAGCACCAATTGATGGACTTGGCCATTTACTGAAATATCGCCTTTACCTAAGGAGGCTGAGTGGTGTGTCACAGCATCAAAGTGGACTGAACAGCTCTTTCATGGGGCCACCCGACACGACCTTTGGGGCGATGTCTTCCCTTAATGGACTCGATTTTCAAACCCTAGCGGCCACGGGCCAAATTTCAGCTCAGAGTCTGGCTTCACTCCAGGCAGCAGCACTTGGCAGGTCAGCTACAAAGCCAGCAATATCGATGCCCCTAGTAGATCAAAGAAACCTGTTTAGCTTTGAAAACCCAAAGTTCAGATTTGTTGAAGGCCAGCAGCCACTGAACAATAATAGTAAACAAATTGGTTTGCTTCATGGAATCCCTACGACTATGGAGCCAAAGCAGCTCGCAAGCCTGAACCAATCTTCACAGACTTTCAGGGGTTGGGGTATGCAACCTCCGGTGCACCAAAACAACTCCTTACTAATGCAGCAGATGGGGCCACCCCAGTCACAGGCTCACATGTTAAATGAACCCAATGGCACTCAAGTTTCGAGGGTGACACAGCCCATATTATCTAATGGGATGCCTAGTGAGCTATTGGCACGAAATGGTATTGTTGACAATTCGCGTGGTGCCATATACCAACCCGTGTCCCAGGCCCAACCACTGACCAGGAGGGGCTTTGCTCCTCCCAGTTATGATATATTCAATGACCTCCAACAGCATAAAGCACAAGATGACTGGGGCATGGGAGCAGTTTTCGAGGCCTCTCGTCTCCCAAATGCACAAGGAACCCTAGATGCGTCACAATCAGTGATGGTTCAGCAAGGGTTTTCTTCAAGCCAGAACAGTGCACAAAATGGGCGCGTGTCTATTGGCAAAGCTGTGTTTCCAAGTGGGCAAGAAAGCGGGAACCCAATGGTTGGTCCACAATTGAACTCACTGCTCGGTGGCAATTCTATAACAATCAAAGCTGAAAGACTCCCTGATGCAAGCTATCAGAACACACTTTTCCCCGACCAGCATGGCCAGGACGACCTCATGAGTGCTCTTCTCAAACAGCAAGAAAGCGTAGGACCAGTTGAAAACGAGTTCAGCTTTGATGGATTTCAATTGGGCAATCTTCCAGTCTAA

>g50246

ATGCAGGTTGAATTCATCAATGACATAGCAGCTTGCTGCTCGGTTGCAGCTCTGACAGAGGCGGAGGCTGCTGCGGTTGTGCCGGAGCAGTTCCCGGTTGGGCTCAGGGTGCTAGTAGTTGACGACGACCTTCTTTGCCTCAGAATTATAGAGCAAATGCTTCGCAAGTGCAAGTACAATGGCTGTTTTGACATTGTGATAAGTGATGTCCATATGCCTGACATGGATGGATTCAAACTTCTTGAGCATGTTGGGCTGGAAATGGACCTTCCTGTTATAATGATGTCAGCAGATGGAAGAACTAACCTTGTCATGAGGGGAATTCGACATGGGGCTTGTGATTATCTGATTAAGCCTATACGTGATGAGGAGTTGAAGAATATCTGGCAGCATGTTGTTAGGAAGAAGTGTAATCTAAGTAAAGAAAATGACCATTCTGGCAGCTTTGAAGATAATGATCAGCCTAAACAGGGAGGTGATGATGCTGAGCATGCTTCTTCTGTTATTGAAGGAGCTGATGGAGTCTTGAAAACGGTGAAAAAGAAAAGAGATTTTAAAGATGATGACGACGATGATGATGATGATGAAATAGAAAATGATGACCCAGCTAATGCAAAGAAACCACGTGTGGTTTGGTCAGTGGAACTTCATCAACAGTTTGTTAGTGCTGTTAACCAACTTGGGATTGACAAGGCTGTACCTAAGAGAATCCTAGAATTAATGAATGTTCCTGGTTTAACAAGAGAAAATGTTGCTAGCCACTTGCAGAAATTTAGGCTTTATTTGAAACGGCTGAGTGGAGTTGCTCAACAACAGGGTGGCCTTCCTAATTCCTTTTGTGGACCAATTGAACCAAATCCAAAGCTGGGTTCTTTGGGGAGATATGAAATTCAAGCACTAGCAGCCTCCTGCCAAATTGCTCCACAAACTTTGGCAGCTATTCATGCTGAGCTTTTAGGGCGACCTACAAGTGGTCTAGTTTTGCCAACAATTGACCATCCAGCTCTGCTACAAGCATCTCTGCCAGGCACAAAATACATTCTTGATGATCAAGCTGTGGCTTATGGTCAGCCTCTGATGAACATCAATCTTAGTGGTCTAGGTGCTCAAAATGGTAATATGTTAACCACAATGATGCAACACCATCAACAACAGCAAAAACAGCAACAAATGGAGCAACATCAGAAGTTATCTACTATTCCAGAGTCCTGCCGTCCGGTTAATGTGCGGCCCTCATGCCTAGTGGTTCCATCTCAGTCTTCAGCCAATTTTCAAGTCACAAATAGTCCTGCTTCAATCAGCCAGACCAGCAGTTTTAGCAAAAGTAATGTAATGGATTCTAGAATTCTTTCTCCACAATCAGGTAATTCTTCTTCAGGTGCTGGGGAAGTAGCAAATTGGGAACAGAAACTCCCATGTAGGTCTAATATGTTTTGCGCTACAGGTTCTCTCTCTCCATCTCTCTCATCATGCTCTACGAATGCTGACAACAGTGCTAGCTGGCAAGTTCAGAACTCAGCATGTATCATTGGTGCTTCTAGACATGCGGCAGGTGTTGTGCCTAACATTACTGGCATCCCTGTTCCAGACAATCACAAATCAAATCAATTGCTTGACCAGGGACCGATTAGGAATCTTGGGTTTGCCAGTAGAGGATCATCCATTCCTAGTCGCTTTGCTATTGATGAATCTGAATCACCACCAATAAGTAACATATATCATTCAAGAATCTATAAGGAAAGCAATACATGCAAAGTGAAGCAGGAGCCAGATGTGAATATTGCTGACAATGCTAAAGTTAGTGTCCAAACGCTGCAAAGAATTCCTCCAAATGACTTCATGAGTGTTTTTCAGTGA

>g26505

ATGACTGTTGATGAAAGTAGAAGAAGGGTTGAAAAGGAAAATAATTCTGACAATTTCCCAGTGGGTATGCGTGTTCTTGCTGTTGATGATGACCCAATTTGCTTGAGGTTATTGGAATGTCTGCTTCGGAAATGCCAGTACCATGTAACTGTAACGAATCAAGCAACAACGGCACTGGAGATGTTGAGAGAAAACAAGGACAGATTTGATCTGGTGATCAGTGATGTCTATATGCCTGATATGGATGGTTTTAAACTGCTAGAGCTTGTTGGTCTTGAGATGGACCTTCCTGTAATCATGTTGTCGGCAAACAGTGATACCAGCCTTGTAATGAAGGGAGTTACTCATGGTGCTTGCGACTACTTGGTGAAACCTGTGCGGATCGAGGAGCTGCGCAACATTTGGCAACATGTAATCAGGAGAAAGACGTTCGATTCCAAACACCATAGCAAGTCCGGTGATCAGGACAATGAAGAAGAAGGTAGACAGGGGGACCAATTATCAGGTACTGCAGAGCAGAGCGAAAAACTGAATAAGAGAAGAAAGGATGAGGAAGACGGGAGTGAGAATGAAGACCCGGCAACGCAGAAAAAGCCCCGTGTTGTTTGGTCTGCAGAACTTCACAGGAAGTTTGTTGCTGCTGTTAACCATTTGGGCATCGAAAAAGCTGTTCCTAAAAGGATTCTTGATATGATGAACGTTGAAGGGCTTAGTCGAGAAAATGTGGCAAGCCATCTCCAGAAGTATAGGCTTTACTTGAAAAGGATCAGTTTGGTTTCAACCCCACAGGCCAACATGGCAACGCCATATATGCCGATGGGTTCACTAGGTGGGTTTGGAGATTTGCAAACATTGGCTGGACCTGGACAACTTAACCGTGCTACATTATCACCATATGTCCCAGGCAGCCTGCTCGGTAGGCTAAATAGCTCTGCTGGTGTAAGCCTTCAAAACCGAAACTTATTGGGTTTGCTCCAACCAAGCCATGCCCAATCTTCTGGTAACTCCCTTGATCTGCTCGGGAAGTTGAACTCAAATGCTCCGCCCACAAGCCAGAACCCGAGTTTGTTTCGAGGAATTCCATCGTTAGAGCTTGATCAGTTGCAACACGGTAAATTCCCCAAAATCGAACAGGTCCTCAATCCGATGGACAACTCGAAGCTTCTCACAGCTGCTACTACATTCACTGGTTCGGGATCTGCTTTTGGTAACCCAATCAATGCTACGATCCTTCAAGGGAACTCCCAGCAAGGACAAACCGGGGAAGGGTTTGGAAATCCACATTCACTCAATATGGCTTCTTTAAGGCCTGAGCCTTTGAACACTGGTGTTAGCAGTGCTTCGAACTTTCTTGGCCATGGAGGACTGAATGGGAATTTGGGGAATTCTATTTTGGCATCAAATGTTCAGCCGAATTGTTATCCGTTGATGGAAACCTTTACTCATAGCCAGTTGGATCAGAATCATGTAAGAGGGAATTATTCGCCCGCTGGCCCTCATTTGCAGAGCAGCCCTCTTGGTTATAATTCTACTTTCTCAACCTCTATACCTTATGAAAACTCGAGGGGACAAACACAATACCAAGAAGGCTTCATCGGTGATGCTATTCAGAGTGTGAATCAGGCACCTACCCAATTTTGGGGAGATCATAACTCAAACTTAAATAATGTTTTCAGCAACTCGAGCTCTCAGATTCTTGGTAATGGATTAATGCCTCCCTTAAGCCAGATTGCCGACCAAAATAATGATATCTTCAATATGAAAACGGATACACCTTTGATTGGTCAAGAAAATGGAGGCTCTGTGGTTCTCTTTCCGCACAATGAGAACGCAAATTTCAATCAAGACTCAAGGATGGGATCCAATGAAGACTACATGTTGAACTCCGCAAAACCACAGGGTGCTTATTCTTCTCTGGATGATTTAATGAATGGAGTAATCAAGGGGGAACAAAATGGGCAATTCGGATTTGATGATTACTTATTTGGGTCATGA

>g50506

ATGGAGAGTGTCATGGCCGGCGGGATCTTTCTACCCAGGAGCGAAACTTTTCCGGCCGGTCTCCGGGTTCTCGTCGTCGACGATGATCCAACCTGGTTGAAGATTCTTGAGAAGATGCTCAAGAAGTGCTCATATGAAGCCACTAACCAAGGGTGTGCATTGGGTAAACCTCGCCTTGTGACCTTTGCTGCAAAAGGACCACTAAAAGTAACTACATGTGGTCTTGCAACGGAGGCTATTAGCCTGCTCCGAGAAAGAAGAAATGGATTTGACATTGTAATAAGCGATGTTAACATGCCTGATATGGACGGTTTCAAGCTTCTGGAACTCGTTGGACTTGAGATGGACCTTCCAGTTATAATGATGTCTGTTGATGGAGAAACAAGCAGGGTTATGAAGGGTGTACAGCACGGTGCCTGCGATTATCTTTTAAAGCCTATTAGGATGAAGGAGCTAAGAAACATATGGCAGCATGTAGTTAGGAAGAGGATGCAAGAATCACGAGACATAGAAAATCACGAAGGAGACCAATTCGATGAAGCTTGGATGTTTAATGGAATTGAACTACAATCGGGAAAGAAAAGAAAAGATTTTGATTACAAATTTGACGAGAGAGAAACAAGTGATTCTAGAAGTGGCGACCCTTCCTCTGTGAAGAAGCCTAGGGTAGTTTGGACGGTAGATCTCCATCAGAAATTTGTCAAAGCTGTAAATCACATTGGATTCGATAAGGTTGGTCCGAAGAAGATTTTGGACTTAATGGGTGTACCTTGGTTGACTCGAGAAAATGTTGCTAGCCACTTGCAGAAGTATCGCCTATACTTGACGAGGTTGCAGAAAGAGAATGAGCTTAAAGCCTCGTCCAGTGGGACAAAGCACCCGGATCTCTCTCCAAAAGAATCCTCTTCAAGTGCGTGCCTTCAGAATTTAGTTGACGTGAAACCAAGTAAGTCAACAAATGGAAAGTACGCTTTTCATGGGGAAAAATTTTGTGTGCAAGAGGTTGAGTCCAGAAATTACGAGGGCGAGGTTAAGGCTGCTGCTCCGTTGTCAACAGCGGGCGTGAGCAGGGCTCTAGTAGGCGAAAATTGTGATTCTCAGAAGAGTATCAGTTGTTCAAAAGCGAGCTGGGCTAGTGAAGTTTCCAAAACCGGTTTCAAACATGAATTCAAGCCGCAGATTCAAACAGAGGACAACATTAACCATCTACCTTCACCGAAGCTTCCTCGAAATGTCCATCTCGATCAAGCACAACCTCTCCTCAATCTTGCGCCTCACAAAGACATAAACCCCGGGGAAATAAAGAGTAAGCCTGGCAACATCAATACCGAAAATCCCGGGGTAAGAACAGTATCTCCGTTAGAATGTGCTGTAGACTTGTTGCCTGCTCAGCCTTCTCAGCCTCAGAGTTGCTTGACAAATTTTCAAGCTTTCGAGCAAATTCCCAGTACTACATGGAGCGCGAAGACTCCCCAGATTCTAATAAACGGTTTAGAATCCGTAGAGGGAAACCTTTTCCTTGGAGGGGGATCATGGGACAAGGATTTTAATGCTGCTGCTCTTCAAGGCGAATTTCATTCCCCATGTGTTGTTGGCCCTCAAAGTTTAGAGCTGTTGGACTACAGCAACACAAACCTCACTGGTGAAATACAACCTTACTTTTATGACTATGAGTATGCCATTGATCCTGTGATAGATCATGGTCTGTTTATATTATGA

>g22427

ATGACTGTAGAGCAGAGGAATGATAAGCAAAATGATCAGTTTCCACTGGGGATGAGAGTGTTGGCTGTGGATGACAACCCAACTTGCCTCATGGTGTTGGAAAATCTGCTTCGGAAATGCCAGTACCATGTTGCAGTTCTGCTCGAATCAGCTTCTGTAATAGACACGGTCTTGGTCATTAATTCTCTGTTCCTATTTGCAGTCACGACAACAAACCAGGCCATCCAGGCATTGCAATTATTACGAGAGAACAAAAATCAGTTTGATTTGGTCATTAGTGATGTTGATATGCCAGATATGGATGGTTTCAAGCTGCTAGAGCTTGTAGGGCTGGAGATGGATTTACCTGTCATAATGCTATCAGCATATGGTGATACGAACCTTGTGATGAAGGGTATCACCCACGGAGCTTGTGATTATTTGCTGAAGCCTGTCCGCATTGAAGAGCTAAAGAACATATGGCAACATGTACTAAGGAGAAAGAAGTTTGACTGCGAGGAGCAGAAAATTTCTAACAAACCTGATGGTGAGTCTGGTGAACTAGGCAGAGGGTTCAGAGGGATGGGAGAAACTGATCGGAATGGTAAGCCTACCAGGAAAAGGAAGGACCAGAGTGATGACGAAGATGAGGAGCTTGATGAAAACGGTGGGCGAAATGAGGACCCATCAGCTCAGAAAAAACCTCGTGTTGTTTGGTCAGTAGAATTGCACCAAAAATTTGTTGCTGCAGTTAATCATCTGGGAATTGATAAGGCTATGCCTAAAAGAATTTTAGAGTTGATGAATGTGGAGAAGCTGACCCGAGAAAATAAATACAGAATTTACCTCAAAAGACTTAATTCTGTTGCATCGCAGCATGCCAACATGGTTTCAGTTTTGGGAAGTGCAGATCCTAGCTACTTAAGAATGGGTTCTCTGAATAATATTGGAAATATCCCTTTTATAACTGGCTGTACTCAATTTTCAGATGCCCCGCTCAGATCCATCTCATCTGGTTCAGTGCTTACTAGACTGAACACTCCTTCTGGACTTGGGATGTGTGGGTTTGCCCCTTCTAGCATGATTCAGCTGGCTAATGCACCTAATTCTAGCAGTTCAATCACTAGCGAAATTAACTTTCGGCAGTCCATACAGCCTGGAAACCAAGATATGGATATTCTTGAAGGGATGCCAATGCCATTAGGAACTGATCAAGTGCACAATAATCTCGGAGTTACTCATCTTTATCCTTTTTCTAATGGTATGCCAGAAAGAAAAATAGATGTTGATGGCAGAAGGAACTTGACTATTGGTGTTTCAGATAACTCTATAATTTTGAGGTCCCAAGGTCAATCTGTTCAAAGGAAAGATTTTCTTGACAATCAGTTCCCTGTTATAGCATCTCCAATGAGTTCAGCAAGTTCTCCCTTTCTGAATACTACGAGATGTAATGACAATTGGCCAACAGCTAGTCAATCATCTCTTCTTGAAGCAAATTCTTTTGGTACTAGTGTCTACTCACACCACGCTATGCCGAGGGATTTGGGAAACAACGGATCTACTTTGGAGGTACCTATGTCTTCTAATTTACATAACCCTTTAAACTCAGCGTGTCCTCAAGTACCTGACACTAGAACAGAGATGCAATGCCTGACAACGATAATTGACAATGTTTCTGGAGTGAAGATGAATTTTAGCCCTCGACAAGATTGGCACGACTTTGAACCGGATTCAGCTCATGTTCCAAGCCTTGTATGCAGCTCTGCTCACACATTTCTTCCTCCAGATGGTGGCCAGAGGCAGCAGCAGCATCATGAATTTGAAAACGCGCCTGTAGATATGAAACAAGAATATTTAGAGGAGCAAAAGATGCTGGCAGGGAATAATGCTTATGGCCAGATGGGATAG

>g16498

ATGACTGTGGAAGAAATAAGAGGACACATGGGGGGTGAAAAGGGAAATCATGACAGTTTCCCAGTGGGCATGAGAGTTCTTGCTGTTGATGATGACCCAATTTGCTTGAAGTTATTGGAAAGCCTGCTTAGGAAATGCCAGTATCATGTAACTATAACAAGTCAGGCAAGAATGGCACTGAAGATGTTGAGAGAAAACAGAGAGAGATTTGACCTGGTGATCAGCGATGTTCATATGCCCGATATGGATGGTTTTAAGCTGCTAGAGCTCGTTGGTCTTGAGATGGATCTTCCTGTCATCAATTTGCGAATTGACCCCATTCCTTTGAAATTTTTTTCAGTGTTGTCGGCGAATAGTGATACCAAACTTGTTATGAAGGGAATAACTCACGGTGCTTGTGACTATCTCGTGAAGCCTGTGCGGATTGAGGAGCTGAGGAACATATGGCAGCACGTAATCCGAAGAAAGAAGTTCGACTCCAAGAGCCAAAACAAGTCGGGTGATCAAGACAGATCTCCTAATGGGGGTGGAGAAGGCGGGCAGGGGGGTCCACTGTCGGGTTCCACAGACCAGAACGGGAAGCTCAACAAGAAAAGGAAGGACGAGGAAGACGAGAGCGACGAGAATGGTCACGAGAATGAAGACCCTGCAACGCAGAAGAAGCCTAGGGTTGTTTGGTCTATAGAACTCCACAGGAAGTTCGTTGCAGCCGTTAACCAGTTAGGCATTGAAAAAGCTGTACCTAAAAGGATTCTTGACCTGATGAACGTTGATGGGCTTACCCGAGAAAACGTGGCAAGCCATCTCCAGAAATATCGGCTCTATTTGAAAAGGATCAGTTCAGTTGCAACCCAACAGGCTAACATGGTGGCGGCCTTTGGGGGTAAGGATTCTGCTTATATGCGAATGGGTTCGCTAGATGGGCTTGGAGATTTTCGGACACTGGCTGGATCGGGAAGATTTAGCCATGCTTCTTTATCATCATATACGCCAGGTGGCATGCTCGGTAGACTTAATAGTGCTGCTGGTGTGAGCATCCGAAATCTTACCTCACCATCGTTGATCCAACCAAGTCACGGGCAAAATTTGGGCAAGCCCCTTGTTTCAACAAAGCGAAGGCACCCACACATACGGGGGGACCTCAATCCCTTGGATGATTCAACGCTCCTTGGAGCTGCTAATACATTCACGGACCCTGGATCGGGGATTGGTAGTTCGAGCAATCCTATGATGCTTCACGGGAATTCTCAACAAGGGCTAATGGCGGGAGGGATTGGAAACCAGCATTCCCTCAACATGGCTTCTTTAAATTCCGAACATTTTAATATTGGTGTTGGCGGTTCTTCCAATTTTCTTGACCATGGACGATCTAGCGACAACTGGCAGAACCCCATTCAGGTGACCAACTTTCAGTCGAGTTCTCTTCCATTGACTGAAACTTTCAATCAAGGCCAGATGCAACAAAATTGTGCGAGAGAAAATAATTCTTCGATCGGCCCTCATTTACAGGGCGGCTGTGCAGGTTATTCTTCCCTCGCCTCTACTGCCACGCCTTTCGAAGATTCAAGGGGAGAAATTCAACGACGGGAAAGGTTGGTAGGCGATGCTATCCCGAGTATTAACCAAGTACCTAGCCAACAATGGGGAGAACTTAAACAAAACCCTAACTCAAACGGCGTTTACAGCAACTTAACCGCTCAGGTTCCCGCCAGCAGCATCGTGCCTCCTTTAAGCCAGAGTATGGACCAATGCAATGACACCGGCAACAGAAGAATGGACGCTTCCAGTCAATCAAATCTCGGTTCTTCGGTTCTCTTGCAACAAAACAAGAACGAAAAGTTGACCTCAGAATCAAGGGCGAGATACTATGAAGACTACCTTTTTGAGCCCCCGAAGCCACAAGGCGCTTTTCCTTCTCAAGGTTACGGCTCTCTGGACGATCTGATGAGCTCAGTGATCAAGCGGGAACAAGATGGAGCGACACTAGAAGGGGAATTCGGGTTTGATGCTTACTCGTTCGGGCCGTGTATATGA

>g47091

ATGACTGTGGAGGAAACTATGAGGAATATGGGGGTTGATAGGGAAAATTACCATAATTTCCCACTGGGTATGAGAGTTCTTGCTGTTGATGATGATCCTATTTGCTTGAAATTATTGGAAGGCCTGCTTAGAAAATGCCAGTATCATGTAACTACAACCAGTCAGGCAAGAATGGCACTGAATATGTTGAGAGAAAACAAAGACCGATTCGACCTGGTAATCAGTGATGTTCATATGCCTGATATGGATGGTTTTAAACTGCTTGAACTCGTGGGGCTCGAGATGGACCTTCCAGTCATCATGCTGTCAGCTAATAGTGATACCAAGCTTGTAATGAAGGGAATAACTCATGGTGCTTGTGACTATTTGGTGAAACCTGTGCGAATTGAGGAGCTGAGGAACATCTGGCAACACGTAATCAGGAGAAAAAAGTCGGAATCCAAGGGCCAAAATCAAGACAATGGTTATCGTGGAAATGGAGAAGGTGGGCAGGGGTTCCCACTGACAGGTTCCGCGGAACAGAATGCATTAGTCAACAAGAAAAGGAAGGATGAAGAGGACGAGACTAATGAGAACGAAGACCCATCCTCGCAGAAAAAGCCTCGTGTGGTTTGGTCTATAGAACTTCACAGGAAGTTCGTTGCAGCTGTTAACCAGTTGGGCATTGAAAAAGCTGTACCTAAAAGGATTCTTGAACTGATGAATGTTGAAGGGCTTACTCGTGAAAATGTGGCAAGCCATCTACAGAAATATAGGCTTTACTTGAAAAGGATCAGTTCAGTTGCAACCCAGCAGGCGAACATGGTGGCTGCTTTAAGGGGTAAAGACTCGGCTTTCATGAGAATGGCTTCACTAGATGGGCTTGGAGATTTTCAAGCATTGGGTGGACCTGGAAGATTTAATCATGCTACATTGTCTACATATACACCTGCTGACATGCTTGGTAGACTAAGTAGTGCCACTGGTGTAAGTATTCGCAACCTTAGTGCATCCGCCTTGGTCCAGTCAAATCACGCCCAAAATTTGGACAACTCCCTTGGTTCTGATGGGAATTTGAACCCAAACATTTCGCTTTCAAGCCACAATGCTGCTAGTTTGTTTCAAGGAATTCCATCGCCTTTAGCTGTACAGGAGCTGAACCCTTTGGAGAATTCTCGGGTGCTTACTGCAGCAAGTGCGTTTGCAGATTCTGGATCTGTTATTGGTAGCTCAACGAATCCTATGATGCTTCAAGGAAGCCCCAAGCAAGGGCTAATCGGGGGAGGATTTGGAAACCAACATTCTCTCAACATGGCTTCTTTAAGTTCTGAACTTTATAATACTGGTGTCAATAGTTCCTCCAATTTTCTTGGCCATGGCAAATCTAGTGAGAATTGGCAGACTTCTATTCAAGTTTCAGAATTCCAGTCTGGCTCTTATCCATTACCCGAACCATTCAGTCATAGCCAGTTGCCTCAAAATTGCGAGAGAGAACACGAGTCTTCAGCTGCCACACATTTACATAGTAGCCCTGTTGGCTTCTCTTCCACCACCTCAGCTTCTACAACTTTTGAAGATTCAAGAGAACCCCAAGTACCTAGCCAACTGTGGGGCGATGGTAAACAGAATCAGAACACAAACGACATTTTCAGAAACTTGAGCTCTCATGTTCCACCTTCTTTAAGCCAGGGCATGTACCAAATGAGTGGCAACTTAAATACAAAGATGAATTCATTTTTAATGCGTCGGTCAAATGCAGGATCTTTGGTTCTCTTTCACCAAAATGGGAATGAAATGCCAACCCCAGACCCCAGGACAAGATCCAACGAAGATAACCTTTTGGAGTCCACAAAGACACACGGGGCATTTGTATCTCAAGGCTTTGACGCCCTGGATGATCTAATGAATGCAGTGATTAAGCAGGAACAAGATGGAGGCATATTGGTTGGGGAATTTGGGTTTGATGCTTACCCATTTGGTTCTTGTATGTGA

>g22235

ATGACTGTAGAGCAGAGGAATGATAAGCAAAATGATCAGTTTCCACTGGGGATGAGAGTGTTGGCTGTGGATGACAACCCAACTTGCCTCATGGTGTTGGAAAATCTGCTTCGGAAATGCCAGTACCATGAAGAGAAACTATACCTCCAATCATCACGACAACAAACCAGGCCATCCAGGCATTGCAATTATTACAGAGAACAAAATCAGTTTGATTTGGTCATTAGTGATGTTGATATGCCAGATATGGATGGTTTCAAGCTGCTAGAGCTTGTAGGGCTGGAGATGGATTTACCTGTCATAAGGTATCACCCACAGACTTGTGATTATTTGCTGAAGCCTGTCCGCATTGAAGAGCTAAAGAACATATGGCAACATGTACTAAGGAGAAAGAAGTTTGACTGCGAGGAGCAGAAAATTTCTAACAAACCTGATGGTGAGTCTGGTGAACTAGGCAGAGGGTTCAGAGGGATGGGAGAAACTGATCGGAATGGTAAGCCTACCAGGAAAAGGAAGGACCAGAGTGATGACGAAGATGAGGAGCTTGATGAAAACGGTGGGCGAAATGAGGACCCATCAGCTCAGAAAAACCTCGTGTTGTTTGAGGCTATGCCTAAAAGAATTTTAGAGTTGATGAATGTGGAGAAGCTGACCCGAGAAAATAAATACAGAATTTACCTCAAAAGACTTAATTCTGTTGCATCGCAGCATGCCAACATGGTTTCAGTTTTGGGAAGTGCAGATCCTAGCTACTTAAGAATGGGTTCTCTGAATAATATTGGAAATATCCCTTTTATAACTGGCTGTACTCAATTTTCAGATGCCCCGCTCAGATCCATCTCATCTGGTTCAGTGCTTACTAGACTGAACACTCCTTCTGGACTTGGGATGTGTGGGTTTGCCCCTTCTAGCATGATTCAGCTGGCCAATGCACCTAATTCTAGCAGTTCAATCACTAGCGAAATTAACTTTCGGCAGTCCATACAGCCTGGAAACCAAGATATGGATATTCTTGAAGGGATGCCAATGCCATTAGGAACTGATCAAGTGAACAATAATCTGGGAGTTACTCATCTTTATCCTTTTTCTAATGGTGTGCCAGAAAGAAAAATAGATGTTGATGGCAGAAGGAACTTGACTATTGGTGTTTCAGATAACTCTATAATTTTGAGGTCCCAAGGTCAATGTGTTCAAAGGAAAGATTTTCTTGACAATCAGTTCCCTGTTATAGCATCTCCAATGAGTTCAGCAAGTTCTCCCTTACTGAATACTACGAGATGTAATGACAATTGGCCAACAGCTAGTCAATCATCTCTTCTTGAAGCAAATTCTTTTGGTACTAGTGTCTACTCACACCACGCTATGCCCAGGGATTTGGGAAACAACGGATCTACTTTGGAGGTACCTATGTCTTCTAATTTACATAACCCTTTAAACTCAGCGTGTCCTCAAGTACCTGACACTAGAACAGAGATGCAATGCCTGACAACGATAATTGACAATGTTTCTGGAGTGAAGATGAATTTTAGCCCTCGACAAGATTGGCACGACTTTGAACCGGATTCAGCTCATGTTCCAAGCCTTGTATGCAGCTCCGCTCACACATTTCTTCCTCCAGATGGTGGCCAGAGGCAGCAGCAGCATCATGAATTTGAAAACGCGGCTGTAGATATGAAACAAGAATATTTAGAGGAGCAAAAGATGCTGGCAGGGAATAATGCTTATGGCCAGATGGGATAG

>g49840

ATGAATGATAATGATGTGTGGTGTTCTTTCCCAAGGGTGAATGATACTAATGTGGTGTTAATCCCAAAGAAGGCTACCCCAGTGAAAGTGTCTGATTTACGCCCTATAGCCCTCTGTAATGTAGTCTATAAAATTATGGCTAAAATGATTGCTAATAGGATGAAACCTCTGTTGGGAGATATAATTTCAGAGTCACAGAGTGCTTTTATTCCGAATAGGCTCATTACTGACAATATTCTAATTGCTGCGGAGATATTGGTCAGGTTATCCCCACGCGTGGCATTCGCCAAGGAGACCCCCTATCTCCCTATCTGTTTATTATTTGTGCAGAAGGACTCTCCCTACTACTCCAACAGGCTGAAACAAGGGGTGACTTTCATGGTCAGGGTTGCAAGAGTGCACCTCCCCTCTCATCTTTTTGCGATGATAGCCTCCTGTTCTTTAAAGCGAATCTTCAAGAGGCTGAGGCTATCAAGCAATGTCTTTATTATGAGCACATGTCGCCGGGTCAACTATCATAAATCCGTGTATGTTTTAGTAAAACACGACTTGAGCAGAGTTCATGTGGCTACGGTTTTGGGTGTAGTTCTCGCACCAAATTTTGGTAAATACCTAGGGCTACCATCGTTCGTAGGAAGAAATAAGAAAGTTGTGTTCTCGTACATTGAAGATAAGATCAAGCAAAGGATTGGGTCATGGAATAAGAAACTGCTATCACAGGCAGGAAAAGAAGTTCTACTCAAGAGTGTGGCCCAATCTATGCCTACATTCGCTATGAGTGTATTTTTACTTCCTGACTCAGTTTGTGTTGCTATTGAGAGGGCAATGAACCGTTATTGGAGGAGAGTGGGGAATGGAAATTCAACTCTAATCCGGGGTCATCCGTGGTTACCTGACGAACCTGATCCAATGATACACACTACCATGCCTCATAATTTAGTTGGCTCAGTGGTTTCAGGCTTGATTGACCCGAGCACAGGAACATGGGACCATTCCATCATTCAGGATATTTTTCAACATGATGACGTAGCCCGTATATTGAAGTGTGGGGAAGATTTTGGTGTGTGGTTTGCTAATGTGTTTTCCATATTAACGGAGGAAGATGTTGTGACTGTTGTAGCAACCTTGTACCATATCTGGAAGACGCGGCTTACCTTCACCAAGACGGAGCAGCTAAAACGGACCTTCATGGCGGTACCTTCAACGGTGGTTTTGTTTTTCACGCTCATGGCCGAAGTCGCTAGCATGCAAAGAAGCTCTATCATGGCTAAAGGACCTGGTCTTACTGTGCACATCTACACGCATTGTTCCACGCTCAAGAATTTGTTATCTACATCTTCTGCTGGTGTTAGTGTTCTTCTTGTTGACGATGATGCTACCTGTCTACGAATACTAGAAGCTTTGCTACTCGCATGCGAATACAAAGTGGTGAAATGTAGAGGGGCTATAGATGCTTTGAGAATCCTTCAAGAGGGTAAAGAGGAGATCGACATTGTTTTAAGCGAGCTACACATGTCTCGCGTCAATGGATTCAAGCTTCTCAATCAAATCATTGGACTGCAAATTGACCTGCCTGTTGTGATGATGTCGAGTGACGAGAGAGTAGATGCAATAAAGCAGATAGTGATCCAGGGGGCATGTGGCTACTTACTAAAGCCGGTGAGGAAAGAAGAAATCAAGGTCTTATGGCAGCATGTTGTTCGTCACAAACAGGGGAATTTGGGGAAGGGCATAAGGCCGCCGCAGGCGGCGGCGTTTTGGGATTCCGGTGAGATGCCGCAGCAGAAAAGTGTAGAAAACTGTGGAAGCTCAAACAATAATAGGGATGAAGATACTAATGCTACTGCTACTACGGCCAATGTTAAGAAACCGCGATTGGTGTGGACACCACAGCTTCACCAGCAATTTGTGGCTGTCGTTAACCAAATTGGTCTTAGAAATGCTGTTCCAAAGAAAATATTGGATTTGATGAACGTTCCCCATCTAAGCAGAGAAAATGTTGCCAGTCATCTCCAGAAATATCGGCTTCATCTACAAAGAAATGGGGATCAGAATTCATACAAGAGGTTGAGCATTCATCAACTCCATTATGAAGACATGGTCTTGAACAAGAACAATGAACAACAAGTGATGCCAGCGACCGGTGACCATAACTATGTTAATGTTTACAACTATGGCAGCCTAGGCCGAGGGAGTAATGTTTACAACTATGGCGGCCTAGGCCGAAGAGTAGGAGGGAGTACAACTACCAACTACTTTTCGGCTTTTCCGCCCTATCAACCCCAATCCACCGGGTATACATACAATAATAATAATCCCAACCAGTTGGGAAACACTTCCTCTGCTCCCAGCCAGCGCCAACAAGATGAGACTCAGTACAATGGTGAAGGAGATTTTGGAGTCTCTTTGTACGATTTTTCTGGCACGGATAGGGCGTGTGGCTACTTACTAAAGCCGGTGAGGAAAGAAGAAATCAAGCTCTTATGGCAGCATGTTGTTCGTCACAAACAGGGGAATTGGGGGAAGGGCGACATAATAAGGCGGCCGCAGGCGGCGGCGTTTTGGGATTCCGGCGAGATGCCGCGGCAGCAGAAAAGTGTAGAAAACTGTGGAAGCTCAAACAATAATAGGGATGAAGATACTAATGCTACTGCTACTACGGCCAATGTTAAGAAACCGCGATTGGTGTGGACACCACAGCTTCACCAGCAATTTGTGGCTGTTGTTAACCAAATTGGCCTTAGAAATGCTGTTCCAAAGAAAATATTGGATTTGATGAACGTTCCCATCTAA

>g20548

ATGGAGAGCCTGAAAGTCCTTGTTGTTGACGATGATTCCACCTGCTTAGCCATTGTTGCAGCCTTGCTCGTGAAAATGGAGTTTCAAGGTATGGTTTCATGGCTTTTTCAATTCTCTCCGATCCGTTCAAGTTCCGTGCATGGTCCGTGTACTTACCGTGCTTCTCCGGTCATTTTCTCCGATTTGATTTTGGGTAAACTTGCTTTGATCTTCGGAAATGGTGAATTAGGGTTTTTTCTCCCACCCTCCCTTTCTTGTTTTTCGAATGTAAAAGTCGTCACAATTTTTTGGGGGAGGAATTCCGGTCGGGTTCCGTGCACCTGCCGTGCGATTCGTGGTTCCATTCCTCAATCGGCTGCAAATTTGTTTTCCTCAACTATTTTCAGTCCAATTAGCGTAATCTTAGTTGTGGCTTTGAAGAACGGCAACGATGCTATTGAGGCCTTACGATCTCAGGGAGGCTTTGATGTGGTCATCTCCGACGTGCATATGCCGGGCATGAATGGATTTGAACTCCAACAACTGATTTTTAAACAATTTCGGATTCCTGTAGTTTTGATGTCCGGTGACTGCGAAGAGGGAATTGTGAGACAAGCAATGCAGAACGGAGCTATTTCGTTCATCAGAAAACCGGTTTCACCTAATGATCTGAGAGGGATATGGCAATATATCATTGCCCAGAAGAGGAGCAAAGTCACAACGGAGCAGGTGAATACCGGTGACCAGGATAACGTTGATATTGATGCTACGCCAAAGAATATATTGAAGGCCATGAATGTGCCTGGATTAACAAGAGAAAATGTTGGCAGTCATTTGCAGAAGTATCGACAATTTCTTCGTCGGAATATTCAAGATATTGAGAACAGAAACAATGGTGGTGGTGGCAATCAACCCAGATTTGGATCAAGAAAATTTCGCAGACTTGCCAAGCTTGAAGAAATGTTTGCCCAAAGTGGACTAGGTGCCGCATTATCGTCGCCGACATCGTCGGCGGCGTTGGGCCGGAGCAGTGCCGGCGGAGATGCAAGAACTCGCGGCGGCCGTGGTGGTTCATCGGAAAGAGGTCAGCAAGGTGTGCTTTTGTCGGAGGACGATGAATCAATGTTTTATCGGGGTTTGAGAAGGGGTCCGATGATTACTAGCCCTAATGAGTCTTCGCCGGCGAGTCATGAGGTGAATCCCGGCGGTTCACAAGAGTCCGGCCCGTATCCTACTCTAACGGCGTTAATGCAGAACCGTTTGGATTCCAGCAGCAACGACAACGTTAACGTTTCGGGTTTCGGAAACGTTAACGTTGTTTCAGGGAACGAAGACGTGCAAGTTCCGGGTGAAGGGAACTTTGATATTTATGACGCTATCTTGAACTTCATCGGAGAAACTGAGGACGGCGGCGACGAAAACATATCCATGTATGGAAACATCACCGCCGGACAAGATAATTCAGGCTACAACCAAATCGGAAATCTCTCAAACGATTTCCTCAATCAAGTAAACATTAATCAGACATACACTCCCAATCAGGGTGACGGCGATGATGATGACGACGCCTTTGTGAATTCTTTGTATGGACCAAACCCTTATGGAGGAGCTGCTGGAAATGGTGAAGGCATGTGA

>g61530

ATGTGGAGATTACCTGAATATCATATTGACAACTCTTATGTTAACATTTCAATGGCTTCATATCATAGGGTAACATATGGCATACATGTCCTCCTGGCTGATCATGACCACAAGTTTCTTGCATCAACTGTTGATATGCTCAAGCGACAATTTTACAAAGTTACCGTGGTGGATTCTGCTAATGCTGCAATTTCAGTCCTTAACCGGAAAGAAGAGAAATTCGATGCGGTGATTGCGAACATTCATTCACCTGATAGGCAAGCGTATAGGCTTCTCCGCGAGGCTGTCAGCATGGATTTGCTAGTGATCTTTTTGTGTGATGAGGAAGATGCTGAGATGGCGGTTAGGGTTATAGAGCACGGAGCATTCGCCCTCCTGCAAAAGCCAACGTGTCAGGAGACGTTGAAAAACTTGTGGCAGCATGTTGTGAGGGAAAGGAGTATGCTGAGAGCAAAGCAGATGATATTTATGGAGAAAACTAACAGGGAATTAGCTGTGATCAACAATGCTGTGATCAACAATGGCGTCGTCGGGGGCGGGGGCGATAATAGGGGTAAGGGAGTGATGAGGGTGGAAGAAAATGAGAACTACCAGATGAGCTACAGGGGAAAGGGGAAGAGGAGTCGGGAGCAGAGCTTGAGCGAGGCGACTCGGATGACGACGACGATGAGTCAGGGCATGTCTAGGGTTAAACGTAAGACGTGCACGGAATGGACTGTGGATCTCCATGAGAAGTTTATGAGTGCTGTTCATCAGCTTGGAGATGGAAGGTGTTATCCGAAGGAGATTCTGGAGCTGATGAATGTGCCTGGGCTGACAAGGATGCAAGTGGCAAGCCATTTGCAAAAATGCCGCAATGATAACTGGAGAGCTCCTGAGGAGAGAAGAGCTCCGCCGATGTCCAGCGCGTCGCCGGCCAGTGGATCCGGGTCCAGAAATGAGCAGCGGAGGTTCGGGACGATGCCTAAGTTAACCGTAGCTGCTGCGGCTGCGGCGGCGGCGGCAGGAGGGAACTCCCAACAGCTGGGAAGCATTGCGTCGCCGGAAGTTCAGTCCTCGCCGTCCATCACCGGTGCGGTGACAGGAGACCCCAGCTCCCAGCCCCCCGCGGACCGCCAGTATCTGGCCATCGGAGCCGCCGCCGCCTTTGTCTCCAAGCTGGAATCCTCTTCTCCCCCGAGCGCCGCCGTGCAGCCCGCGGTCGTCGCCACCGCCGTCGGCACCTTCACCGCAACTTCCGGCCAGGCCTCCATTGTTGGGAGCGTTAATTACGGCACCGGAGGAATTCTGCAAGCACTCGGCTGCGGCGTTGGTGGTAGTGGTGGGAATTTGTTCCGCAACAAAGATGCTTTCACTGATGACCCCAACAACAACACCACCGCCGGTGACGACTCTTTCGTCACTCCCCAGCCGCCCCGCATCCACCGCAGGCTTCAGTCGGCCGATGAATTCTTCAGCTTCAACGACGTGGACTATGAATATCTGATTCAAGGTTTCTCCGACAACAATGCTCGCCAGGCAGGCGTTGCGCTGCAGGCCCCTACCCACAACAACACATCCTCCTCTGAGTTCAACGACAAAGCAGGTTTCAACCCCGTGCAGAACCAGGAAGCAAAGGCACAAACTTCGGAGATGGACATTGCGAAGATCAGTCCTTAG

>g38332
[truncated: 212,356 more chars]
