# Supplementary figures and images for: Genome-wide identification and expression analysis of two-component system genes in sweet potato (Ipomoea batatas L.)
Source: Front Plant Sci. 2023 Jan 12;13:1091620. doi: 10.3389/fpls.2022.1091620 (PMC9878860; doi:10.3389/fpls.2022.1091620)

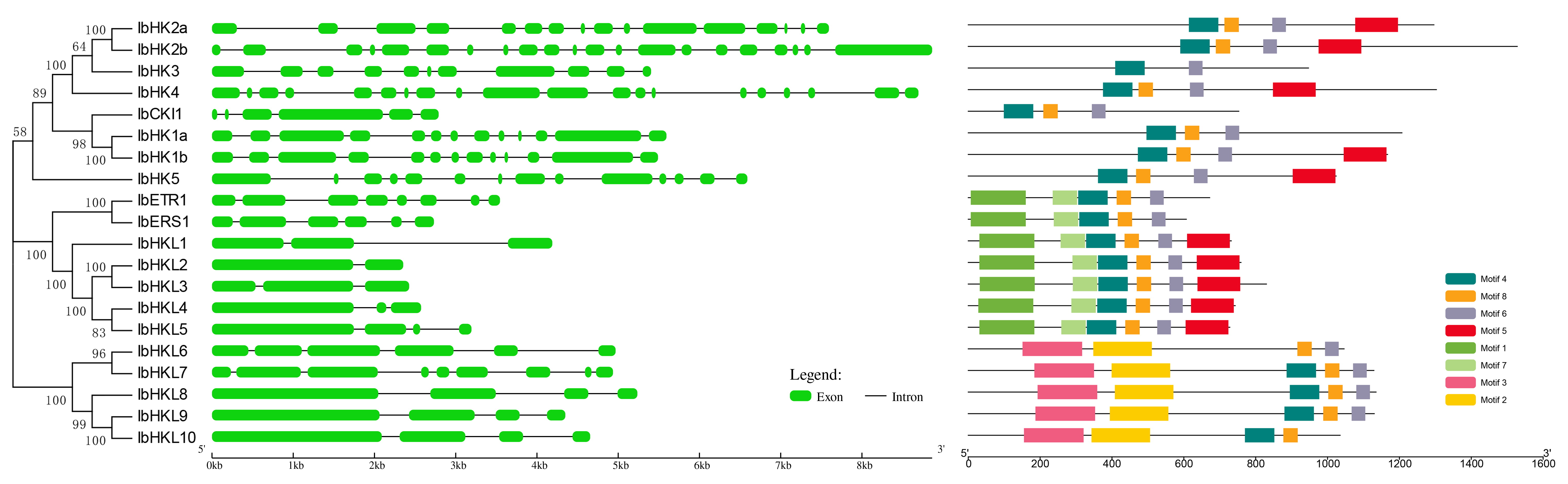

Supplement: Supplementary file 3 [file Image_1.jpeg]

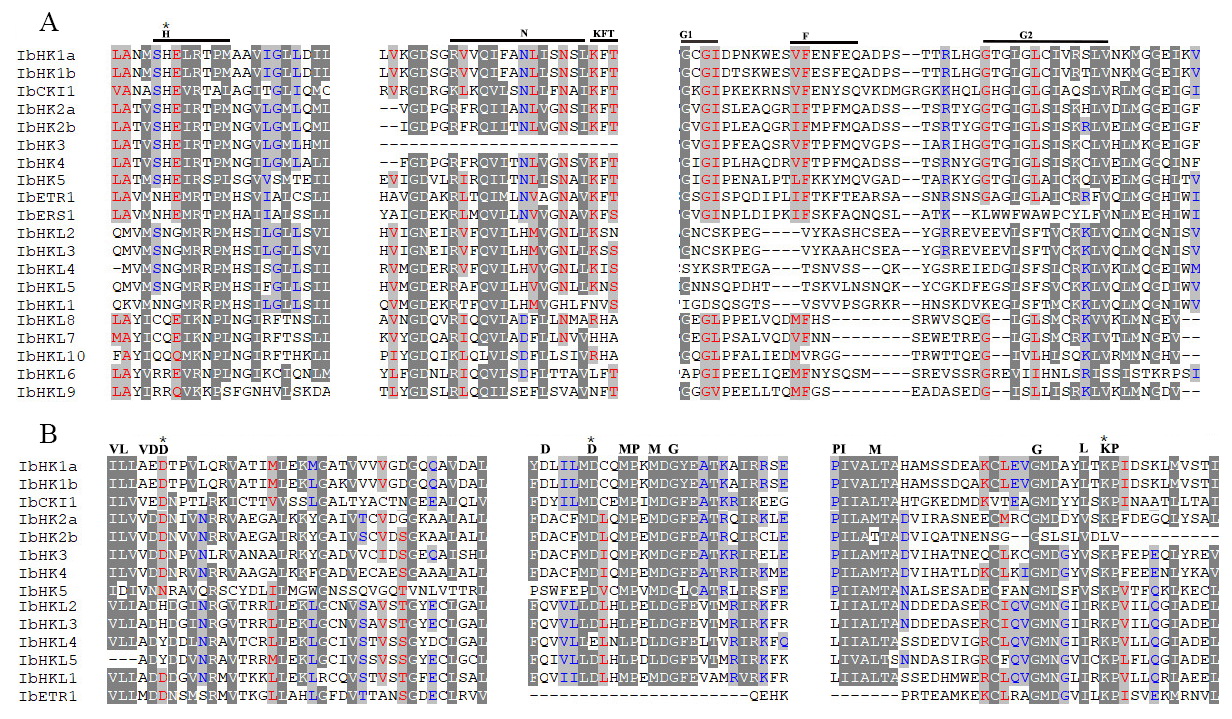

Supplement: Supplementary file 4 [file Image_2.jpeg]

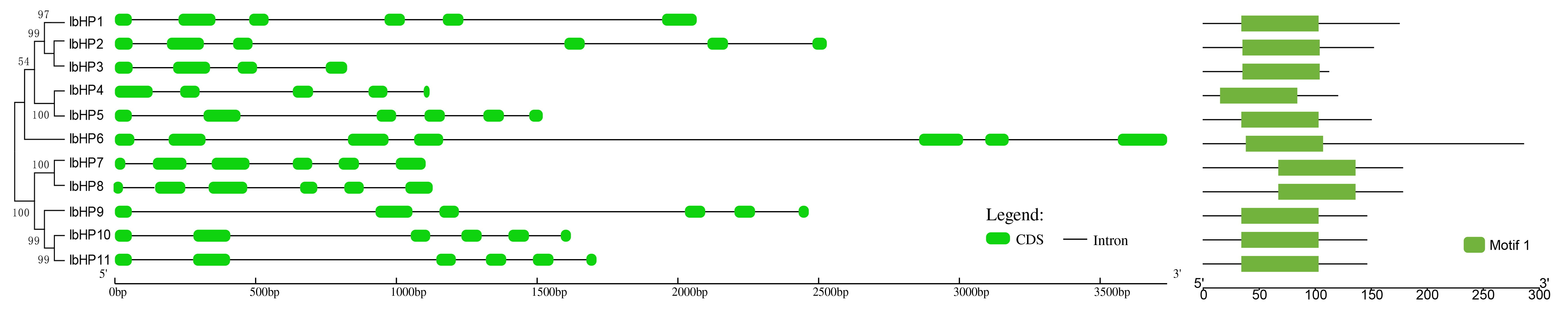

Supplement: Supplementary file 5 [file Image_3.jpeg]

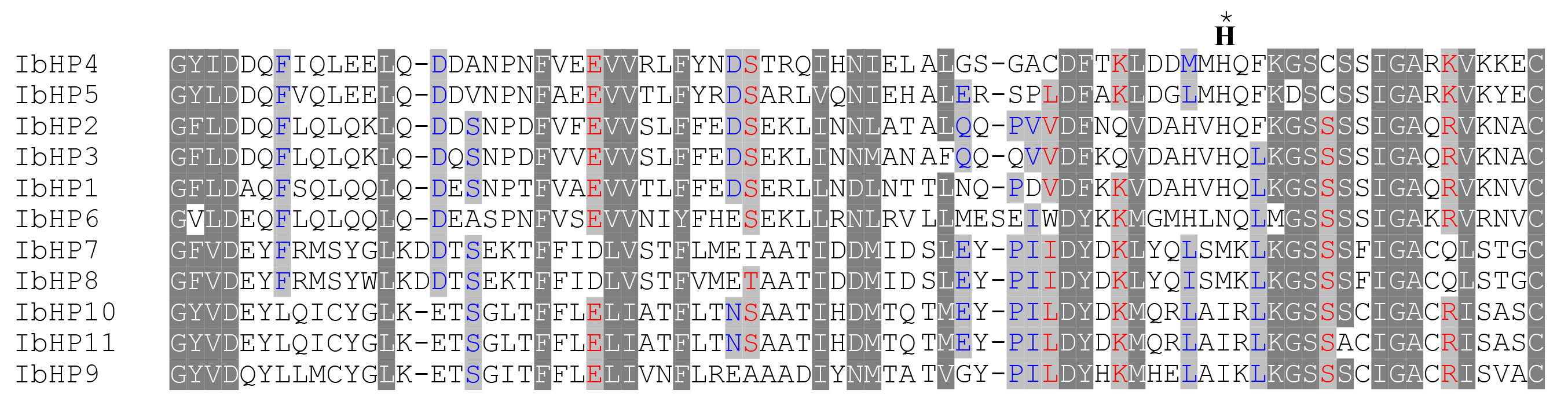

Supplement: Supplementary file 6 [file Image_4.jpeg]

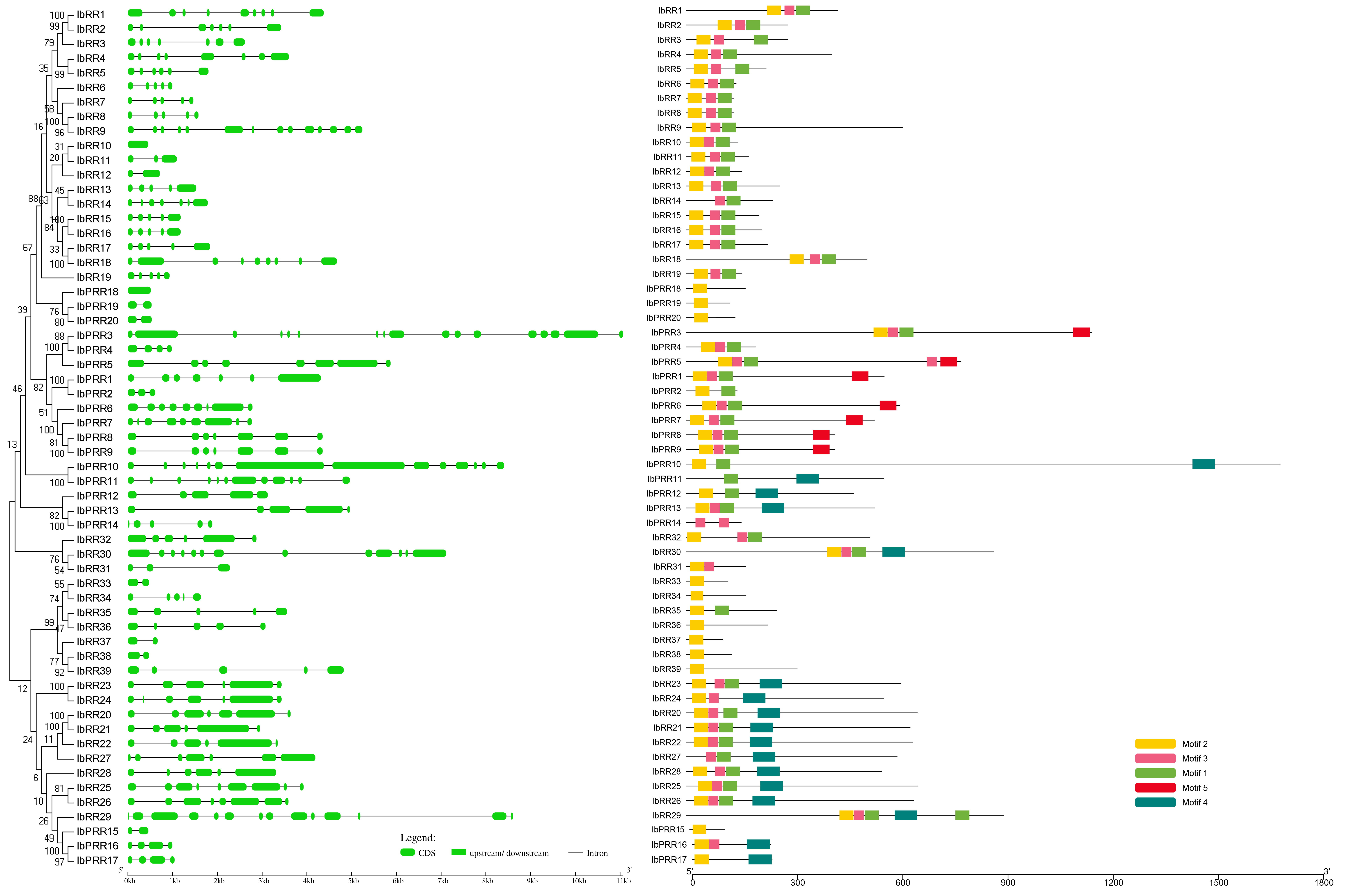

Supplement: Supplementary file 7 [file Image_5.jpeg]

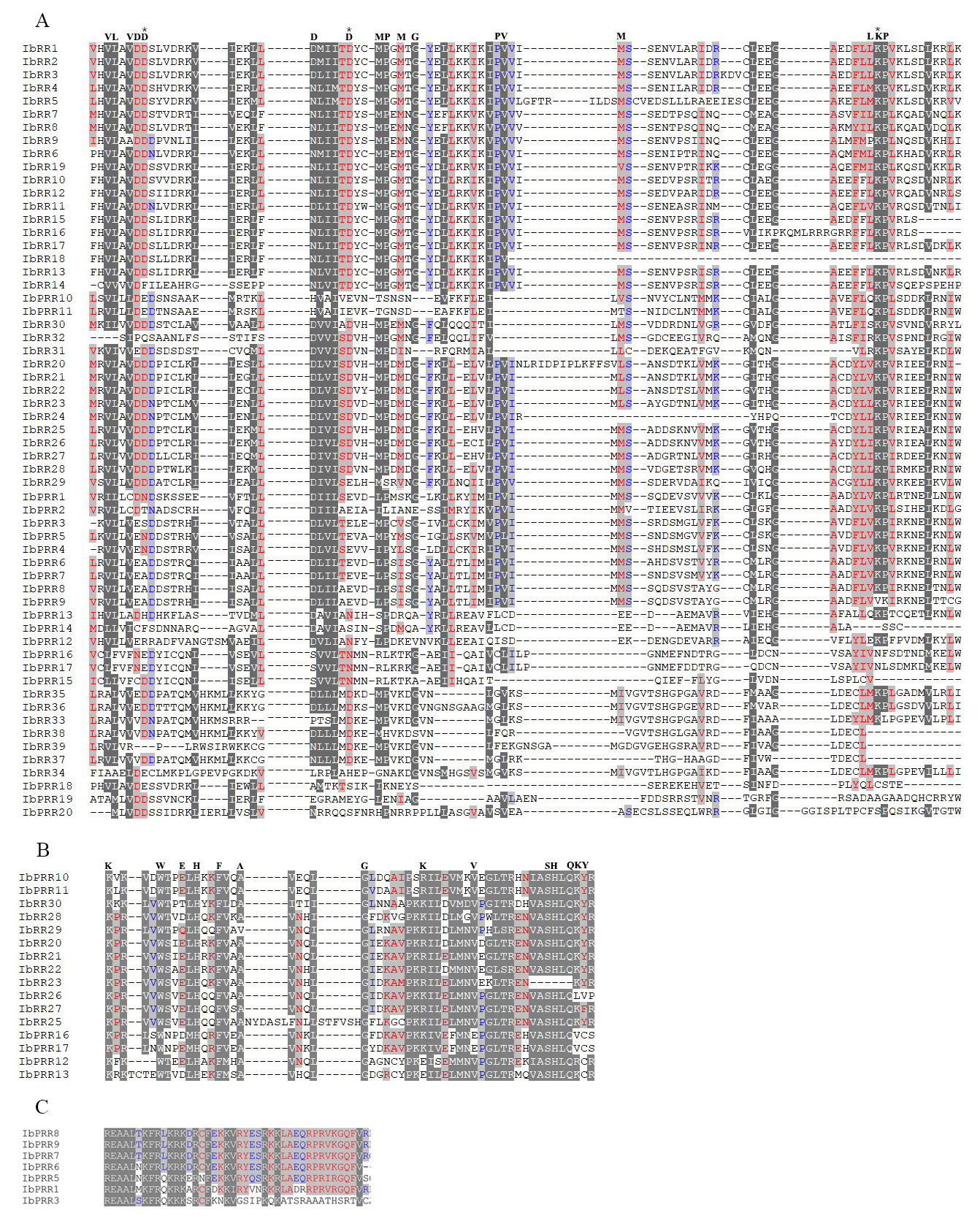

Supplement: Supplementary file 8 [file Image_6.jpeg]

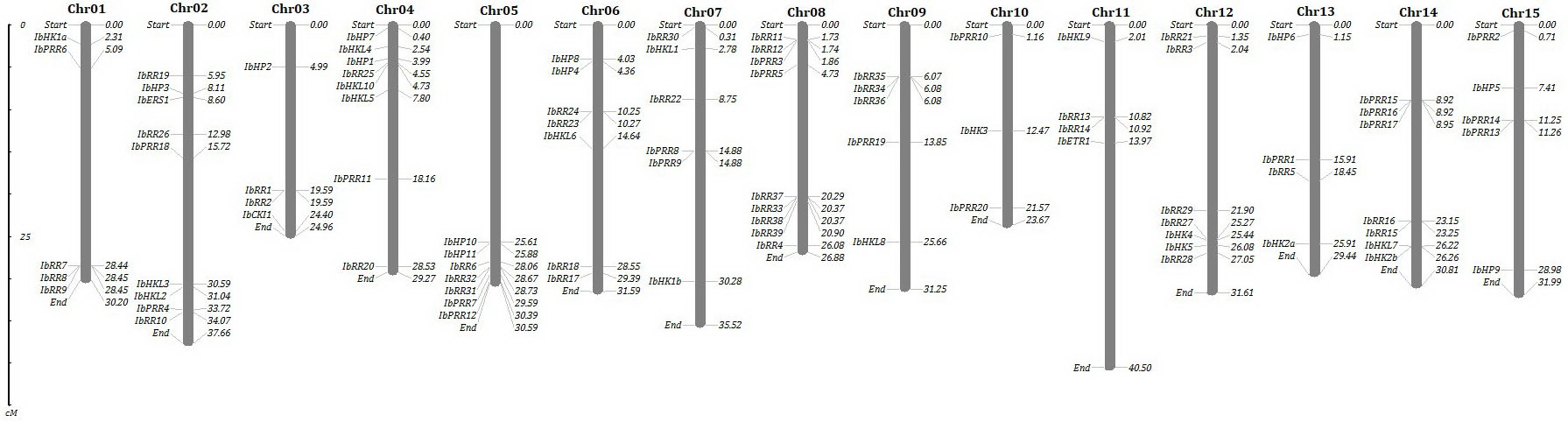

Supplement: Supplementary file 9 [file Image_7.jpeg]

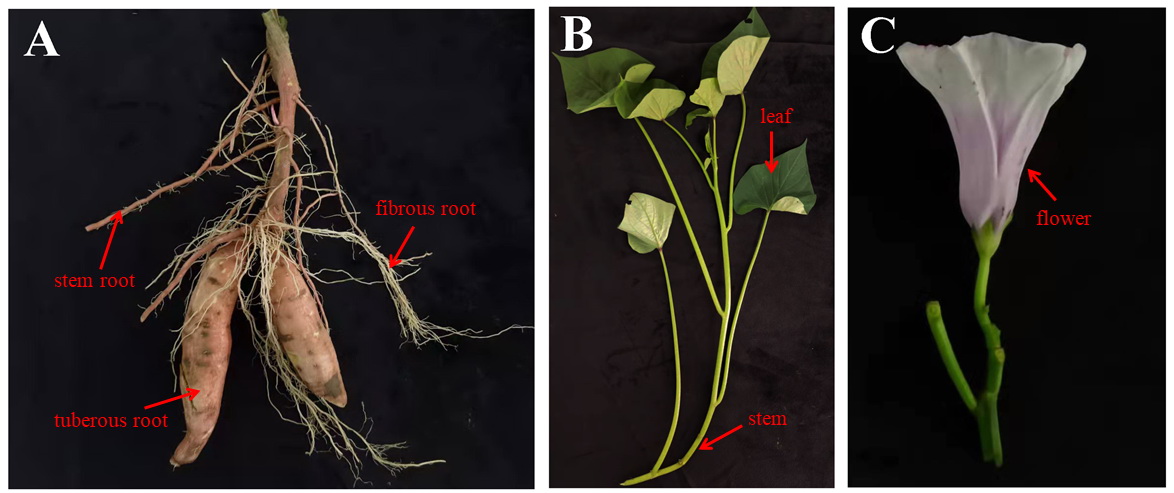

Supplement: Supplementary file 10 [file Image_8.jpeg]
