# Supplementary material for: Genome-wide identification and expression analysis of two-component system genes in sweet potato (Ipomoea batatas L.)
Source: Front Plant Sci. 2023 Jan 12;13:1091620. doi: 10.3389/fpls.2022.1091620 (PMC9878860; doi:10.3389/fpls.2022.1091620)
Supplement: Supplementary file 1 [file DataSheet_1.zip › Supplementary Table S1. Forward and reverse primers used in the qRT-PCR gene expression studies.doc]

Table S1. Forward and reverse primers used in the qRT-PCR gene expression studies.

| Primer name | Forward sequence | Reverse sequence |
| --- | --- | --- |
| *IbARF* | CTTTGCCAAGAAGGAGATGC | CTTGTCCTGACCACCAACA |
| *IbActin* | CTGGTGTTATGGTTGGGATGG | GGGGTGCCTCGGTAAGAAG |
| *IbHK1a* | AGTAAGACAGCGAACGAACAAA | TCAGGATCAAGTCATAAGGTGG |
| *IbHK1b* | AGGCAAGGAATAGGTCGGTAG | TTCGGTTTGGGTAGTTGTTGA |
| *IbHK2a* | GGTTACGTGCAGAATCTGGTAG | GTGGAGAACCCTTAGAGCATAG |
| *IbHK2b* | TATGAACTGGCAATCGAGAAA | AGTAGCTGAGGTTGTTGAGGC |
| *IbHK3* | ATAGCAAAGGTTGAGGACGACT | TTTACCACTAGCCTGTGCAGTT |
| *IbHK4* | CAAGCCGACAGTTCCACTTC | TTCAAATCAACGCTGCCATT |
| *IbHK5* | GAAACCGAACTTAACAGAACAATC | CAAATCACCCGAAGACAACAT |
| *IbCKI1* | TCTCGTAGGTTGGCTGATTTCG | TTCTGTGGCTTCCTTCTGCTTT |
| *IbETR1* | ACAGCGTCTGATGGTGGAA | ATGGCATACTCGGGCAAAT |
| *IbERS1* | GAAACTGACTTAAATCCCGAACA | CGACCTCCCGAAACACTCC |
| *IbHKL1* | TGACGAGGGATTGTTTAGTGTAAG | GATAAGCGTGAGAAGGGTGATT |
| *IbHKL2* | GCTTTCAACTCGGGCCATCCAC | TTCGCTCGCATCTTCATCGTCA |
| *IbHKL3* | GGTGCAGCAAATCTTCCAGTCT | TTCACTCGCATCTTCATCGTCA |
| *IbHKL4* | TAGAGCAGTAACCCGCCGTAT | TGCTGGCATCGTTGTTTGA |
| *IbHKL5* | CGTTTCAACTCAAGCCATCA | CACCGAGACAATCGTATCCAG |
| *IbHP1* | TAATCAGCCAGATGTGGACTT | GTCTCCGAGCTAGTATTATTGTT |
| *IbHP2* | CACCAGTTCAAGGGAAGCAGT | CGAGTCTGAGTAAAGTTTCGAGTT |
| *IbHP3* | AGCGTACATGGCTTCCCTGTA | TGGAAGGCATTGGCCATATT |
| *IbHP4* | AAGTATTGGTGCCAGAAAGGT | TCTTGTTTTGCCATCTGAAAG |
| *IbHP5* | GGCAGCTTGCCACCATGAGGA | TGCGTGCATTCATATTTCACCTT |
| *IbRR20* | ACGACGGGAAAGGTTGGTAGG | CTGGCTTAAAGGAGGCACGAT |
| *IbRR21* | AGCAAGTGCGTTTGCAGATTC | TTCGGGTAATGGATAAGAGCC |
| *IbRR22* | GCAAACATTGGCTGGACCTGG | TGTGGGCGGAGCATTTGAGTT |
| *IbRR23&24* | TAGGCAGAGGGTTCAGAGGGA | CTCGGGTCAGCTTCTCCACAT |
| *IbRR25* | ATGCTCCGAGAACAGGCTAA | GGGTCGAGGATTCCTTGTAAAT |
| *IbRR26* | GCTTTGCTCCTCCCAGTTATG | TCTTGCCCACTTGGAAACAC |
| *IbRR27* | TGCTTGACCAGGGACCGATTA | CTGGCTCCTGCTTCACTTTGC |
| *IbRR28* | AAAGCGAGCTGGGCTAGTGAA | GTGAGGCGCAAGATTGAGGAG |
| *IbRR29* | CAATCTATGCCTACATTCGCTAT | GTCAATCAAGCCTGAAACCAC |
