# Supplementary material for: Genome-wide identification and expression analysis of two-component system genes in sweet potato (Ipomoea batatas L.)
Source: Front Plant Sci. 2023 Jan 12;13:1091620. doi: 10.3389/fpls.2022.1091620 (PMC9878860; doi:10.3389/fpls.2022.1091620)
Supplement: Supplementary file 1 [file DataSheet_1.zip › Supplementary Table S2. Forward and reverse primers used in yeast-two hybrid assays.doc]

Table S2. Forward and reverse primers used in yeast-two hybrid assays. Overlap sequences with the *pGADT7* or *pGBKT7* vectors were marked with red font.

| Primer name | Primer sequence |
| --- | --- |
| *IbHK1a-pGADT7-F* | CCATGGAGGCCAGTGAATTCATGGCTGTTGGATCCAATACAA |
| *IbHK1a-pGADT7-R* | AGCTCGAGCTCGATGGATCCTCAGTTTTTTCTCTTTGTTAAT |
| *IbHK1b-pGADT7-F* | CCATGGAGGCCAGTGAATTCATGGCGTACAGAGCCAATAGAA |
| *IbHK1b-pGADT7-R* | AGCTCGAGCTCGATGGATCCTCAAGCTTGTAGGCTCTTCGTT |
| *IbHK5-pGADT7-F* | CCATGGAGGCCAGTGAATTCATGGTATCTGAGATGGAGAATG |
| *IbHK5-pGADT7-R* | AGCTCGAGCTCGATGGATCCTTACAGGCGATGGCGCTGCGGC |
| *IbHP1-pGBKT7-F* | TGGCCATGGAGGCCGAATTCATGGAGGTGAGCCAATTGCAGA |
| *IbHP1-pGBKT7-R* | CGCTGCAGGTCGACGGATCCTCACTTTGTTGTGGGCACTAGT |
| *IbHP2-pGADT7-F* | TGGCCATGGAGGCCGAATTCATGGACGTTGTGCCTCAGCTGC |
| *IbHP2-pGBKT7-R* | CGCTGCAGGTCGACGGATCCTTATGGCAGAACAGGAATTTTC |
| *IbHP3-pGBKT7-F* | TGGCCATGGAGGCCGAATTCATGGAAGTTGTAGGACAGCTGC |
| *IbHP3-pGBKT7-R* | CGCTGCAGGTCGACGGATCCTCACCCATCAAGACTCCTCTCC |
| *IbHP4-pGBKT7-F* | TGGCCATGGAGGCCGAATTCATGGGATACATTGACGATCAAT |
| *IbHP4-pGBKT7-R* | CGCTGCAGGTCGACGGATCCTCAAGAATCTTGTTTTGCCATC |
| *IbHP5-pGBKT7-F* | TGGCCATGGAGGCCGAATTCATGGAAAGAAACCACTTGCCTA |
| *IbHP5-pGBKT7-R* | CGCTGCAGGTCGACGGATCCTCAGTTAGGGCGACATGCCACC |
| *IbRR20-pGADT7-F* | CCATGGAGGCCAGTGAATTCATGACTGTGGAAGAAATAAGAGGAC |
| *IbRR20-pGADT7-R* | AGCTCGAGCTCGATGGATCCTCATATACACGGCCCGAACGAGTAA |
| *IbRR21-pGADT7-F* | CCATGGAGGCCAGTGAATTCATGACTGTGGAGGAAACTATGAGGA |
| *IbRR21-pGADT7-R* | AGCTCGAGCTCGATGGATCCTCACATACAAGAACCAAATGGGTAA |
| *IbRR22-pGADT7-F* | CCATGGAGGCCAGTGAATTCATGACTGTTGATGAAAGTAGAAGAA |
| *IbRR22-pGADT7-R* | AGCTCGAGCTCGATGGATCCTCATGACCCAAATAAGTAATCATCA |
| *IbRR23-pGADT7-F* | CCATGGAGGCCAGTGAATTCATGACTGTAGAGCAGAGGAATGATA |
| *IbRR23-pGADT7-R* | AGCTCGAGCTCGATGGATCCCTATCCCATCTGGCCATAAGCATTA |
| *IbRR24-pGADT7-F* | CCATGGAGGCCAGTGAATTCATGACTGTAGAGCAGAGGAATGATA |
| *IbRR24-pGADT7-R* | AGCTCGAGCTCGATGGATCCCTATCCCATCTGGCCATAAGCATTA |
| *IbRR25-pGADT7-R* | CCATGGAGGCCAGTGAATTCATGAATCTGGGTGGTGGTCAAGTGG |
| *IbRR25-pGADT7-R* | AGCTCGAGCTCGATGGATCCCTACCCACCGAAGAAGACTTGCGCC |
| *IbRR26-pGADT7-F* | CCATGGAGGCCAGTGAATTCATGTCAGTTCATAGCTCAGTTGCTT |
| *IbRR26-pGADT7-R* | AGCTCGAGCTCGATGGATCCTTAGACTGGAAGATTGCCCAATTGA |
| *IbRR27-pGADT7-F* | CCATGGAGGCCAGTGAATTCATGCAGGTTGAATTCATCAATGACA |
| *IbRR27-pGADT7-R* | AGCTCGAGCTCGATGGATCCTCACTGAAAAACACTCATGAAGTCA |
| *IbRR28-pGADT7-F* | CCATGGAGGCCAGTGAATTCATGGAGAGTGTCATGGCCGGCGGGA |
| *IbRR28-pGADT7-R* | AGCTCGAGCTCGATGGATCCTCATAATATAAACAGACCATGATCT |
